# Convergent Deboronative and Decarboxylative Phosphonylation Enabled by the Phosphite Radical Trap “BecaP”

**DOI:** 10.1021/jacs.3c06524

**Published:** 2023-08-08

**Authors:** Santosh K. Pagire, Chao Shu, Dominik Reich, Adam Noble, Varinder K. Aggarwal

**Affiliations:** † School of Chemistry, University of Bristol, Cantock’s Close, Bristol BS8 1TS, U.K.; ‡ National Key Laboratory of Green Pesticide, College of Chemistry, Central China Normal University (CCNU), 152 Luoyu Road, Wuhan, Hubei 430079, China

## Abstract

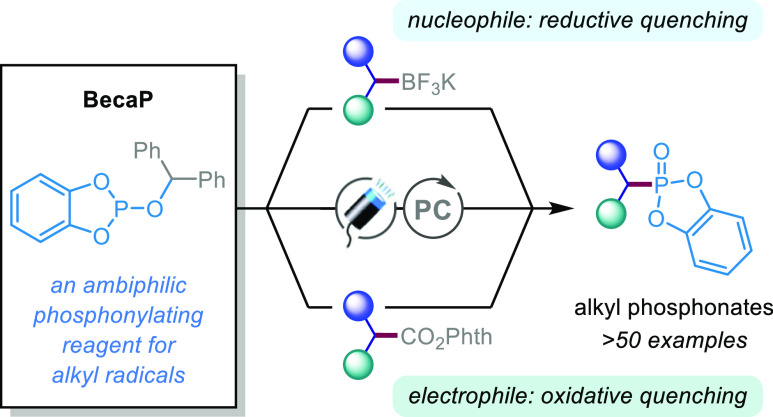

Carbon–phosphorus bond formation is significant in synthetic chemistry because phosphorus-containing compounds offer numerous indispensable biochemical roles. While there is a plethora of methods to access organophosphorus compounds, phosphonylations of readily accessible alkyl radicals to form aliphatic phosphonates are rare and not commonly used in synthesis. Herein, we introduce a novel phosphorus radical trap “BecaP” that enables facile and efficient phosphonylation of alkyl radicals under visible light photocatalytic conditions. Importantly, the ambiphilic nature of BecaP allows redox neutral reactions with both nucleophilic (activated by single-electron oxidation) and electrophilic (activated by single-electron reduction) alkyl radical precursors. Thus, a broad scope of feedstock alkyl potassium trifluoroborate salts and redox active carboxylate esters could be employed, with each class of substrate proceeding through a distinct mechanistic pathway. The mild conditions are applicable to the late-stage installation of phosphonate motifs into medicinal agents and natural products, which is showcased by the straightforward conversion of baclofen (muscle relaxant) to phaclofen (GABA_B_ antagonist).

## Introduction

**Scheme 1:**
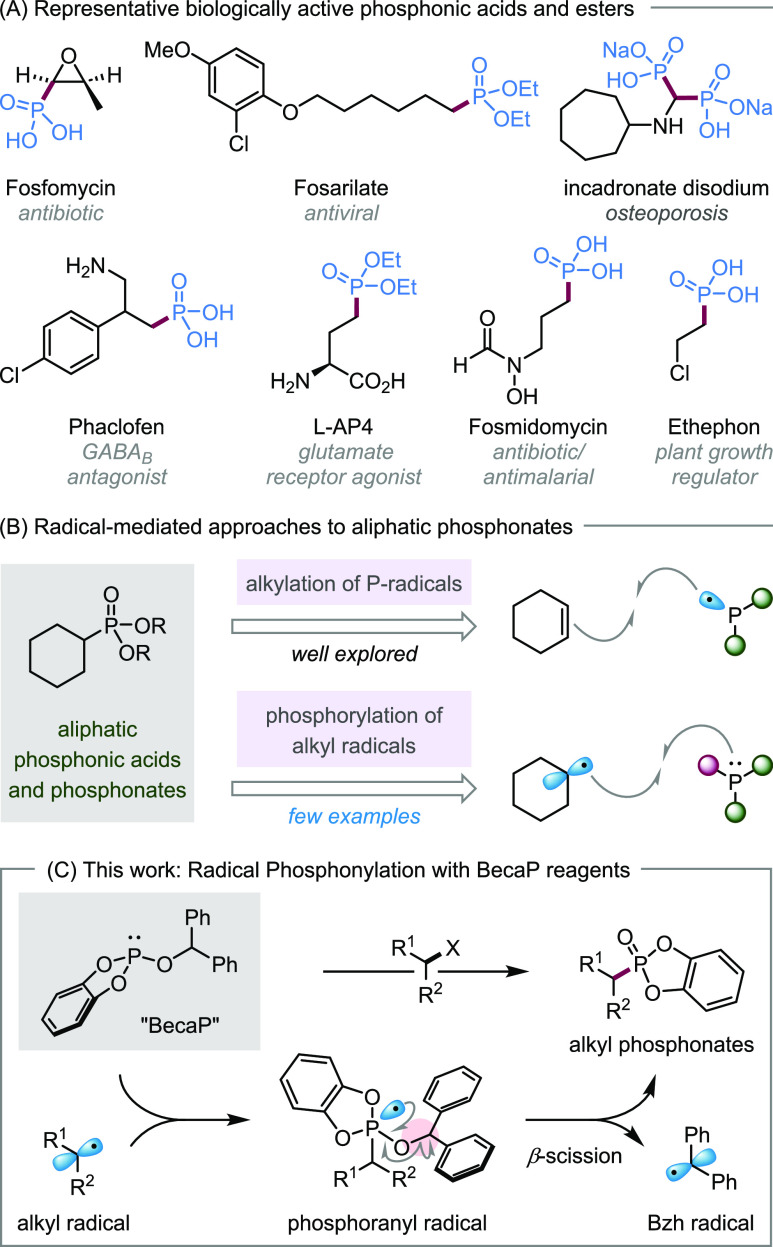
Biologically Relevant Phosphonates and Radical-Mediated Approaches to Aliphatic Phosphonates

Organophosphorus compounds have a rich history, with applications spanning organic synthesis, materials science, agrochemistry, and medicinal chemistry.^1^ In particular, due to their profound biological activity, phosphonic acids and esters are found in numerous pharmaceuticals and agrochemicals (Scheme 1A).^2,3^ Methods to access organophosphonates commonly involve two electron processes,^4^ where C–P bond formation is achieved through the reaction of nucleophilic phosphites or H-phosphonates with electrophilic halides^5^ or C=X bonds,^6^ or reactions of electrophilic phosphorus(V) reagents with organometallics.^7^ One electron processes are also known;^8^ however, their application to the synthesis of alkyl phosphonates typically involves the reaction of phosphorus-centered radicals with alkenes (Scheme 1B, top).^9,10^ In contrast, C–P bond formation by addition of alkyl radicals to phosphorus reagents is rather underdeveloped (Scheme 1B, bottom).^11^ While highly reactive aryl radicals add rapidly to trialkyl phosphites, yielding aryl phosphonates after β-scission of the intermediate phosphoranyl radicals,^12^ the lower reactivity of alkyl radicals means that they either do not react or undergo unproductive reversible addition.^11a^ As a result, methods for C–P bond formation from alkyl radicals rely on the use of highly reactive phosphorus reagents,^13^ which mainly limits their utility to the synthesis of phosphines,^14^ whereas phosphonylations with phosphites to access alkyl phosphonates are limited to highly reactive alkyl radicals, such as bicyclo[1.1.1]butyl radicals.^15^

We reasoned that finding a general solution to alkyl radical phosphonylation could open vast opportunities for radical-mediated synthesis of alkyl phosphonates. In particular, we sought a phosphite reagent that would act as an efficient trap for alkyl radicals under mild conditions and thus allow us to exploit visible light-mediated photoredox catalysis for the generation of alkyl radicals from diverse functional groups.^16^ This would enable the transformation of feedstock chemicals, such as boronic acids and carboxylic acids, into their phosphonic acid counterparts—a potentially highly useful process which could be applied to peptides, drugs, and natural products.^17^

When considering potential phosphite reagents that could efficiently phosphonylate alkyl radicals, we aimed to address the challenges associated with (1) the low reactivity of alkyl radicals toward common trialkyl phosphites to form phosphoranyl radicals and (2) the unproductive α-scission of the resulting phosphoranyl radical outcompeting the desired β-scission pathway. We hypothesized that these challenges could be overcome by designing a phosphite that contained (1) an aromatic diol ligand, which could stabilize the phosphoranyl radical intermediate to facilitate radical addition to phosphorus^18^ and (2) an efficient radical leaving group to promote subsequent β-scission. Herein, we report the phosphonylation of a diverse range of alkyl radicals with benzhydryl-catechol-phosphite (BecaP), which incorporates a phosphoranyl radical-stabilizing catechol ligand and a benzhydryl (Bzh) radical-leaving group (Scheme 1C). These key structural features enable phosphonylation to occur under mild photocatalytic conditions and in the absence of additional transition-metal catalysts.^19^ Notably, the ease of both single-electron oxidation and reduction of the Bzh radical during the photocatalytic cycle allows BecaP to undergo redox neutral reactions with both nucleophilic and electrophilic alkyl radical precursors, thus making it a highly versatile reagent for photoredox-catalyzed phosphonylations.

## Results and Discussion

**Scheme 2:**
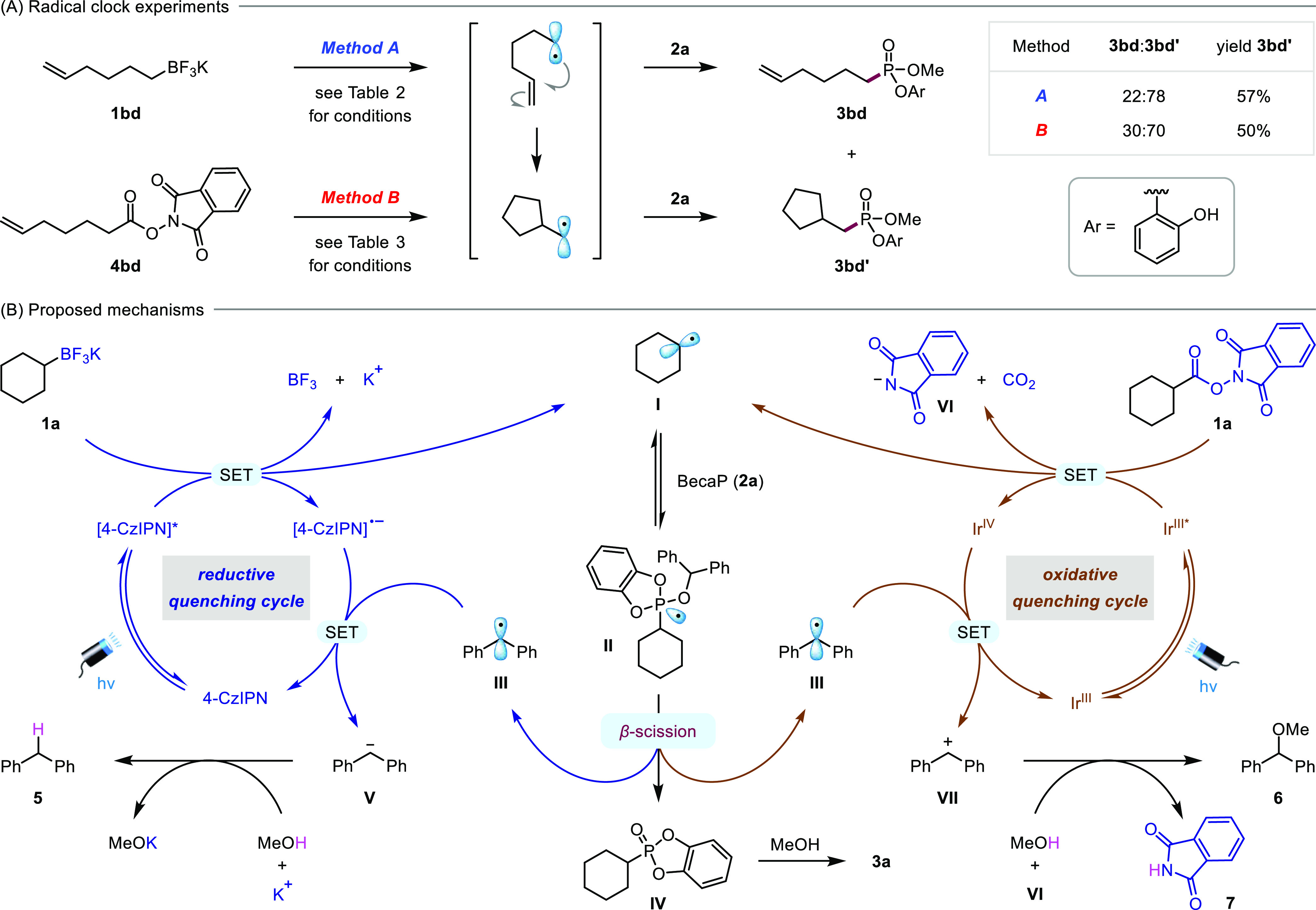
Mechanistic Studies and Proposed Mechanisms for the Deboronative and Decarboxylative Phosphonylations

We began our studies by investigating the deboronative phosphonylation of alkyl BF_3_K salts **1** with a range of potential phosphite radical-trapping agents (Table 1).^20^ After extensive optimization, we found that blue light irradiation of a mixture of cyclohexyl potassium trifluoroborate (**1a**) and BecaP (**2a**) in the presence of the organic photocatalyst 4CzIPN and MeOH in 1,4-dioxane gave phosphonate **3a** in 85% yield (entry 1). It should be noted that a mixture of phosphonate products was obtained due to partial ring-opening of the initially formed cyclic catecholate phosphonate (see **IV** in Scheme 2) by the reaction with methanol to form **3a**; therefore, an excess of methanol was added after irradiation to give **3a** as a single product. We subsequently confirmed the crucial roles of both the catechol ligand and the benzhydryl group on **2a**, since phosphites devoid of either were ineffective phosphonylating reagents (entries 2–3 and Table S1). The optimum reagent, BecaP (**2a**), is a free flowing crystalline solid, which can easily be accessed on a multigram scale from commercially available precursors in a single step, without chromatographic purification, and can be stored under an inert atmosphere at lower temperature for longer use. In the absence of MeOH, only trace **3a** was detected (entry 4), whereas lower yields were obtained when the amount of MeOH was decreased (entry 5 and Table S3; see Table S2 for alternative additives investigated). Increasing the MeOH loading beyond 5 equiv also caused a decrease in the yield (entry 6), which was found to result from a competing reaction with **2a** (see Supporting Information, Section S5.3). Lowering the loading of **2a** from 2.0 to 1.5 equivalents decreased the yield (entry 7). Alternative solvents and photocatalysts were also investigated, but all were found to be less effective compared to the standard conditions (entries 8–9 and Tables S4 and S5). Control experiments showed that light and photocatalyst were both essential for the reaction (entries 10–11).

**Table 1:**
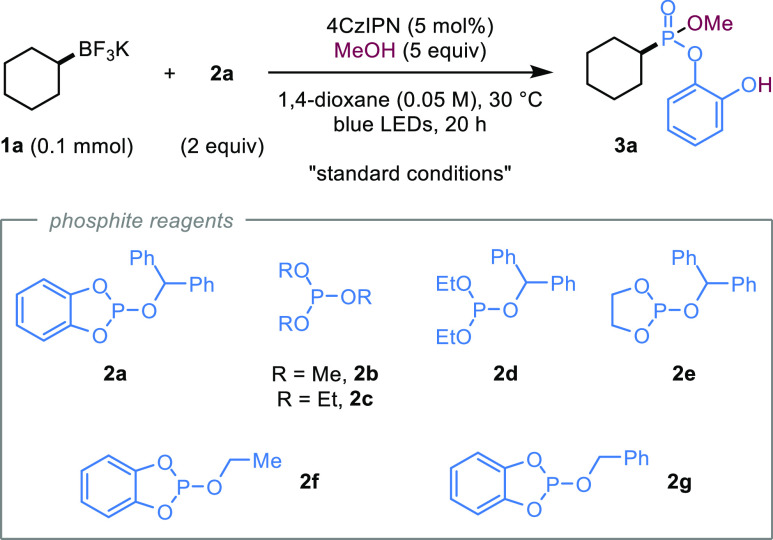
Optimization of the Deboronative Phosphonylation

entry^a^	Variation from “standard conditions”	**3a** (%)^b^
1	no change	85 (81)^c^
2	**2b–2f** instead of **2a**	≤1^d^
3	**2g** instead of **2a**	18
4	no MeOH	2
5	2 equiv of MeOH	57
6	10 equiv of MeOH	65
7	1.5 equiv of **2a**	77
8	MeCN as the solvent	73
9	Ir[(ppy)_2_(dtbbpy)]PF_6_ as the photocatalyst	75
10	in the dark	0
11	no 4CzIPN	0

a Reaction conditions: **1a** (0.10 mmol), **2a** (0.20 mmol), MeOH, 5 mol % 4CzIPN, solvent (0.05 M, 2.0 mL), 40 W blue Kessil LED lamps (×2), N_2_, r.t., 20 h, then MeOH (0.3 mL), 1 h.

b Yields were determined by ^1^H NMR analysis using diethyl phthalate as an internal standard.

c Yield of the isolated product after column chromatography.

d For phosphites **2b–2e**, the corresponding products are dimethyl, diethyl, or ethylene glycolato phosphonate esters instead of catecholato phosphonate ester **3a**.

With optimal conditions in hand, the scope of the deboronative phosphonylation reaction was probed (Table 2). Primary alkyl-BF_3_K salts (**1b–1l**) were smoothly transformed into the desired alkyl phosphonates (**3b–3l**). A range of functional groups were tolerated, including esters (**3g**), acetals (**3i**), halides (**3j**), nitriles (**3k**), and carbazoles (**3l**). Cyclic secondary alkyl-BF_3_K salts (**1n–1p**) were also effectively phosphonylated, delivering the corresponding phosphonates **3n–3p** in good yields. However, lower yields were obtained with more hindered acyclic substrates, with *sec*-butyl phosphonate **3m** formed in low yield. Unfortunately, tertiary alkyl-BF_3_K salts (**1q**) proved unreactive, likely due to the low reactivity of the sterically hindered tertiary alkyl radicals with **2a**. Notably, derivatives of drug molecules and natural products, such as ibuprofen (**3r**), 3-indole acetic acid (**3s**), camphanic acid (**3t**), and dehydrocholic acid (**3u**), could be formed in synthetically useful yields, indicating that the method can be used for late-stage installation of phosphonate groups.

**Table 2:**
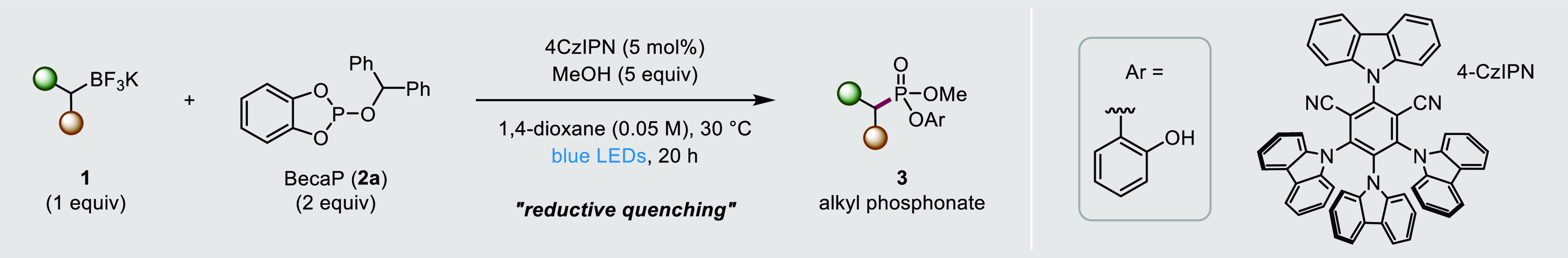

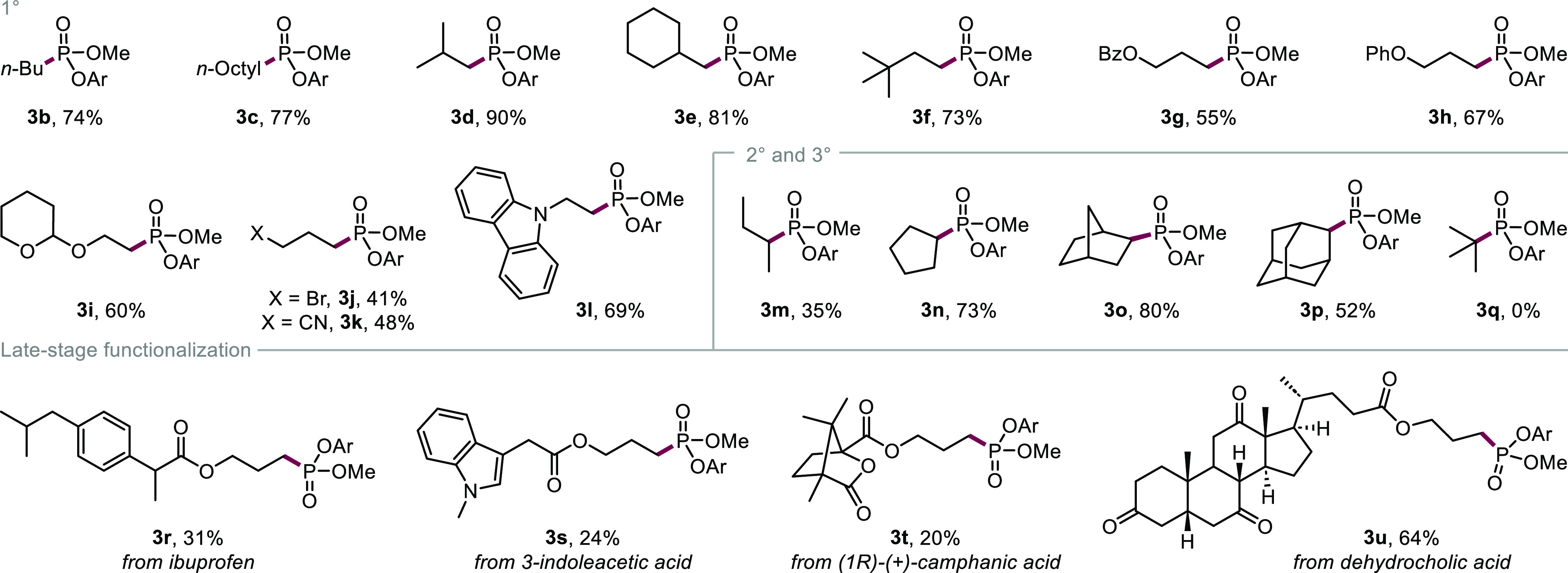
Substrate Scope for the Deboronative Phosphonylation with BecaP^a^

a Reaction conditions: **1** (0.30 mmol, 1 equiv), **2a** (0.60 mmol, 2.0 equiv), MeOH (1.5 mmol, 5 equiv), 4CzIPN (5 mol %), solvent (0.05 M, 6.0 mL), blue LEDs, N_2_, r.t., 20 h, then MeOH (1 mL), 1 h.

We subsequently investigated the versatility of BecaP by applying it to decarboxylative phosphonylations.^13,17a,21^ Since carboxylic acids are readily available feedstock chemicals, and a common source of alkyl radicals in visible light photocatalysis, their use would significantly increase the reach of this chemistry.^22^ We recently reported a photoredox-catalyzed decarboxylative phosphonylation of *N*-hydroxyphthalimide (NHP) esters derived from α-amino acids.^23^ This reaction proceeded through a radical–polar crossover pathway, where C–P bond formation occurred via the reaction of trimethyl phosphite with an intermediate iminium ion, which limited its application to the formation of α-amino phosphonate esters.^21^ We reasoned that using BecaP in place of trimethyl phosphite would enable a radical-mediated C–P bond formation and therefore significantly expand the scope of decarboxylative phosphonylations beyond α-amino acids.^19^ Gratifyingly, after minor modifications to the reaction conditions (see Tables S6–S8 for optimization studies), we found that phosphonate **3a** could be formed in 81% yield from the corresponding cyclohexyl NHP ester **4a**. This result is notable because NHP esters are formal electrophiles, with decarboxylation triggered by single-electron reduction by the excited state photocatalyst (oxidative quenching);^22b^ whereas trifluoroborate salts are nucleophilic reagents, with deboronation promoted by single-electron oxidation (reductive quenching).^20^ Given that both the deboronative and decarboxylative phosphonylation reactions proceed efficiently in the absence of stoichiometric oxidants or reductants, the BecaP reagent must act as an ambiphilic radical trap, where it is able to function either as a formal electrophile or nucleophile to enable redox neutral photocatalytic reactions with nucleophilic and electrophilic radical precursors, respectively. This makes BecaP a versatile radical trap in photoredox-catalyzed phosphonylations since the success of the reaction is not dependant on the electronics of the alkyl radical precursor.

This decarboxylative phosphonylation of NHP esters **4** was found to be applicable to a broad range of aliphatic carboxylic acids (Table 3). Primary carboxylic acids (**4v–4ab** and **4af–4aq**) bearing distinct functional groups, such as alkenes (**3an**, **3ap**), alkynes (**3ab**), esters (**3af**, **3ag**), tertiary amines (**3ai**), halides (**3v**, **3w**, **3aa**, **3ah**, **3ai**), heteroaromatics (**3z**), carbamates (**3af**-**3ah**, **3ak**), and ketones (**3aj**, **3aq**), were converted into the corresponding primary phosphonate esters in good to high yields. In addition to cyclohexyl (**1a**), other cyclic secondary carboxylic acids, including cyclopropyl (**3ac**)^14c,18a^ and indanyl (**3ad**), could be phosphonylated in high yields. However, tertiary carboxylic acids were unsuccessful (**3ae**), mirroring the deboronative phosphonylation and observations made by Wang, Zhu and Li in their recently reported photoredox and copper-catalyzed decarboxylative phosphonylation.^19^ Pleasingly, derivatives of proteinogenic amino acids bearing carboxylic acid side chains, including Fmoc-protected aspartic acid and Boc-protected glutamic acid, delivered the β-amino and γ-amino phosphonate esters **3af** and **3ag**, respectively, in good yields. The potential for the application of this methodology in late-stage decarboxylative phosphonylations was also demonstrated by the successful formation of phosphonate derivatives of a range of natural products and medicinally relevant carboxylic acids (**3ah–3aq**). While the optimized conditions were found to be suitable for the majority of carboxylic acids investigated, for several products (**3ad**, **3ah**, and **3al**), dramatically enhanced yields were achieved when a mixed solvent system of MeCN/tetrahydrofuran (THF) was used due to the low solubility of the NHP esters in MeCN. In addition, increasing the amount of BecaP to 3.5 equiv improved the yield in the phosphonylation of *cis*-pinonic acid (**3aj**), which was attributed to competing ring-opening of the cyclobutane by β-scission of the alkyl radical intermediate.

**Table 3:**
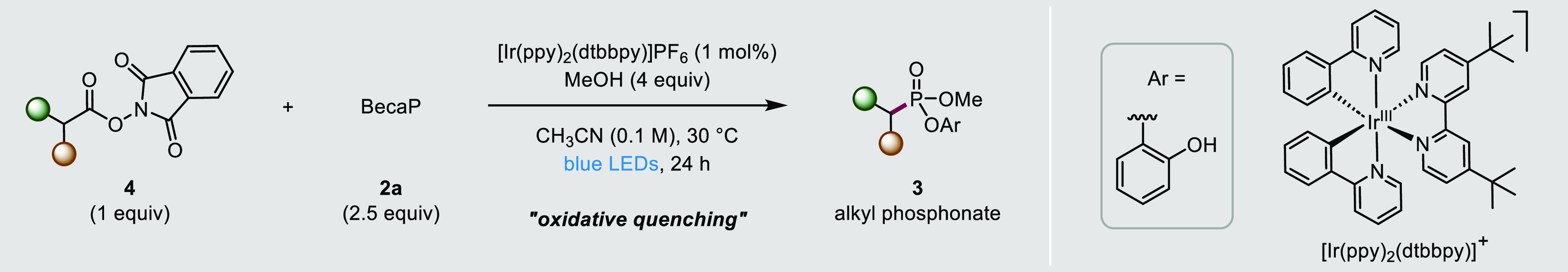

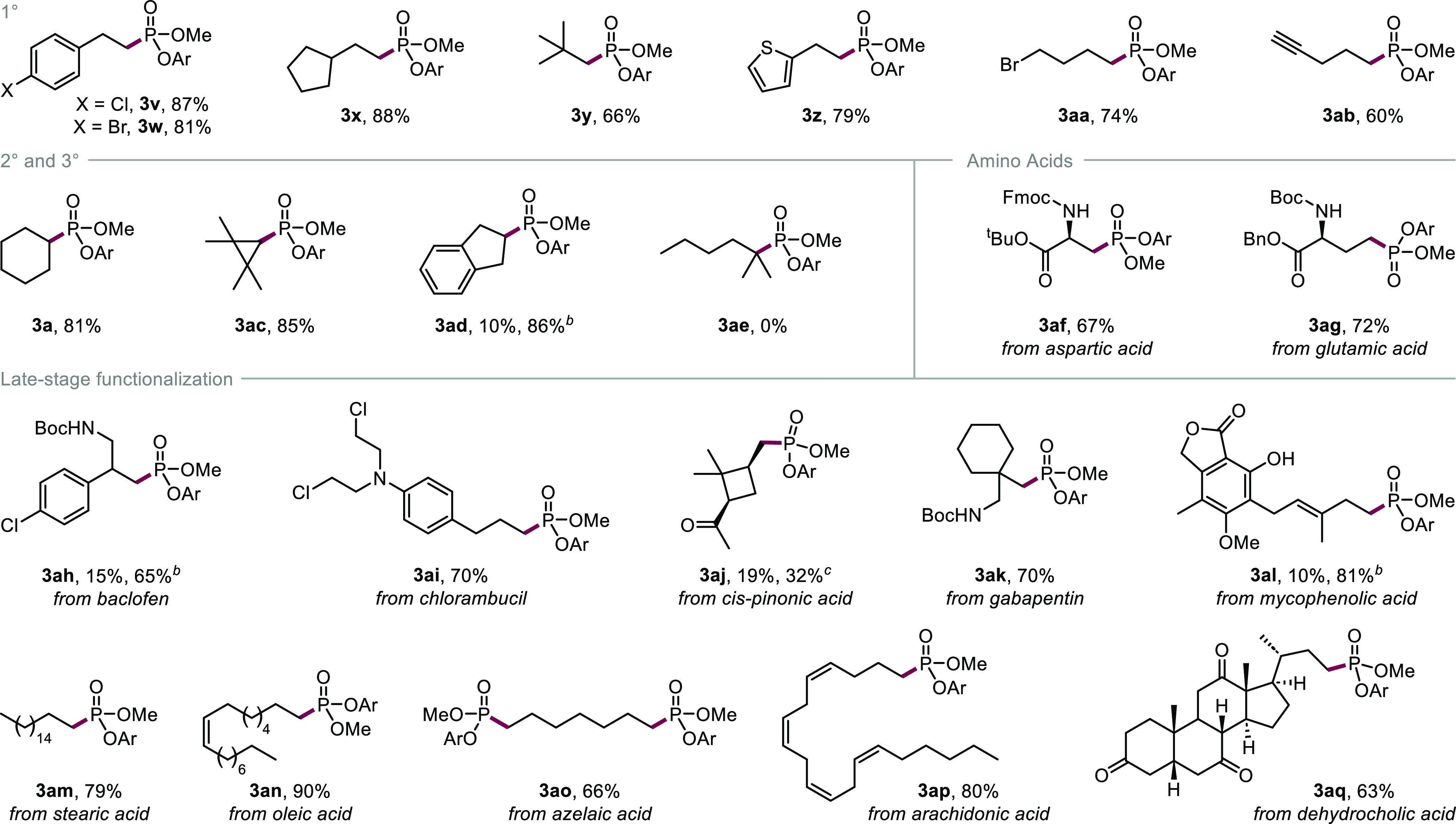
Substrate Scope for the Decarboxylative Phosphonylation with BecaP

a Reaction conditions: **4** (0.20 mmol, 1 equiv), **2a** (0.50 mmol, 2.5 equiv), MeOH (0.8 mmol, 4 equiv), [Ir(ppy)_2_dtbbpy]PF_6_ (1 mol %), solvent (0.1 M, 2.0 mL), blue LEDs, N_2_, r.t., 24 h, then MeOH (1 mL), 1 h.

b MeCN/THF (2:1) as a solvent mixture (0.1 M).

c 3.5 equiv of **2a** was used.

Next, we directly compared the deboronative (method A) and decarboxylative (method B) phosphonylations over a range of substrates (Table 4). We were pleased to find that both methods provided comparable yields, although some exceptions were noted, with the decarboxylative protocol giving significantly higher yields for phenethyl (**3ar**), butenyl (**3at**), and isopropyl (**3az**) products, whereas the deboronative process provides an enhanced yield of methyl carboxylate ester **3av**. These results highlight the versatility of BecaP as a radical phosphonylating reagent under photoredox catalysis since it enables phosphonylations of electronically opposing substrates and, as a result, provides the opportunity to maximize product yields through judicious choice of the alkyl radical precursor. Finally, we found that methanol could be replaced with other alcohols under the optimized conditions for both the deboronative and decarboxylative reactions, which gave ethyl (**3aw**), isopropyl (**3ax**), and benzyl (**3ay**) phosphonates in good yields.

**Table 4:**
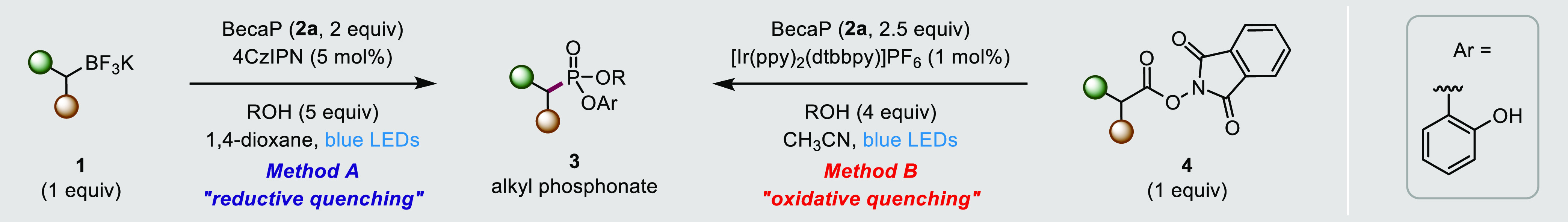

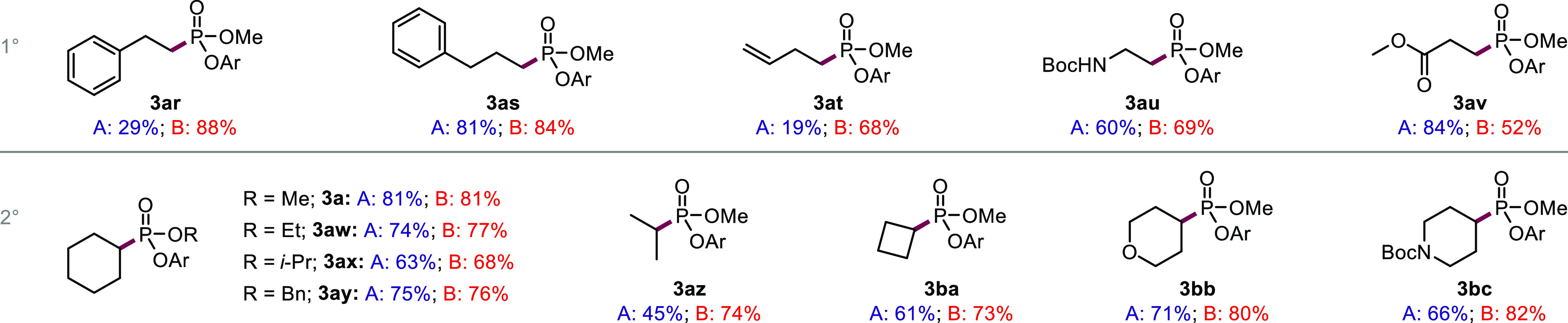
Comparative Substrate Scope for the Deboronative and Decarboxylative Phosphonylation with BecaP^a^

a See Tables 2 and 3 for reaction conditions for methods A and B, respectively.

Mechanistic studies were conducted to provide evidence for the proposed radical pathway. The phosphonylations of alkyl BF_3_K salt **1a** and NHP ester **4a** with BecaP were both inhibited by TEMPO, and the formation for the intermediate cyclohexyl radical was confirmed by the observation of the corresponding TEMPO adduct by mass spectrometry (see Supporting Information, Section S5.2). Radical clock experiments with substrates **1bd** and **4bd** gave the cyclic phosphonate **3bd′** selectively over the acyclic product **3bd**, which also confirmed the radical nature of the reactions (Scheme 2A).^24^ The change in the ratio of **3bd**:**3bd′** between methods A and B reflects the different concentrations of **2a** under the two conditions; the lower concentration of **2a** under the deboronative conditions (method A, [**2a**] = 0.1 M) leads to more cyclic product due to a slower rate of phosphonylation of the radical intermediates compared to the decarboxylative conditions (method B, [**2a**] = 0.25 M). By using the ratio of cyclized (**3bd′**) and uncyclized (**3bd**) products (method A = 3.5; method B = 2.3) as an approximation for the relative rates of 5-*exo*-trig cyclization and phosphonylation, and the known rate constant for cyclization of the 5-hexenyl radical (2.7 × 10^5^ s^–1^ at 30 °C),^25^ the rate constant for phosphonylation of primary alkyl radicals can be estimated to be 7.7 × 10^5^ M^–1^ s^–1^ in 1,4-dioxane and 4.7 × 10^5^ M^–1^ s^–1^ in MeCN.^26^

From the above studies, the following mechanism is proposed for the deboronative phosphonylation reaction (Scheme 2B, left). Reductive quenching of the excited-state photocatalyst (*E*_1/2_ [4CzIPN*/4CzIPN^–•^] = +1.35 V vs SCE)^27^ by single-electron transfer (SET) with potassium trifluoroborate **1a** (*E*_p_ = +1.5 V vs SCE)^28^ results in deboronation to generate alkyl radical **I** and BF_3_. Addition of **I** to the phosphorus center of BecaP (**2a**) gives the transient phosphoranyl radical **II**, which is stabilized by delocalization of the unpaired electron onto the catecholate ligand.^18b^ Subsequent rapid β-scission occurs due to the presence of the benzhydryl group, generating the highly stabilized benzhydryl radical **III** and cyclic catecholate phosphonate **IV**, which undergoes ring-opening upon reaction with MeOH to form **3a**. Turnover of the photocatalyst is enabled by reduction of benzhydryl radical **III** (*E*_1/2_ = −1.14 V vs SCE)^29^ to anion **V** by the reduced state of the catalyst (*E*_1/2_ [4CzIPN/4CzIPN^–•^] = −1.21 V vs SCE).^27^ Finally, protonation of **V** by methanol gives diphenylmethane (**5**), which was detected by mass spectrometry.

For the decarboxylative phosphonylation, single-electron reduction of NHP ester **4a** by the excited state iridium photocatalyst (Ir^III^*, where Ir^III^ = [Ir(ppy)_2_(dtbbpy)]PF_6_) generates the same alkyl radical **I**, along with CO_2_ and phthalimide anion **VI** (Scheme 2B, right).^22a,22b^ Given that this oxidative quenching of the photocatalyst (*E*_1/2_ [Ir^IV^/Ir^III^*] = −0.96 V vs SCE)^30^ by **4a** (*E*_p_ = −1.23 V vs SCE)^31^ is endergonic based on the reduction potentials, we believe that this SET process is facilitated by hydrogen-bonding activation of **4a** by MeOH.^32^ Following addition of **I** to **2a** and β-scission to form phosphonate **IV**, turnover of the photocatalyst is possible because the benzhydryl radical **III** (*E*_1/2_ = +0.35 V vs SCE)^29^ can be oxidized to cation **VII** by the oxidized state of the catalyst (*E*_1/2_ [Ir^IV^/Ir^III^] = +1.21 V vs SCE].^30^ Finally, **VII** is trapped by MeOH to form benzhydryl methyl ether **6**, and proton transfer to **VI** forms phthalimide (**7**). The formation of byproducts **6** and **7** was confirmed by LC–MS and nuclear magnetic resonance (NMR) analyses of the crude reaction mixture (see Supporting Information, Section S5.3).

The above mechanisms highlight the versatility of BecaP as a radical phosphonylating agent, able to undergo productive redox neutral photocatalytic reactions via two mechanistically distinct pathways (reductive and oxidative quenching). This is possible because of the ability of benzhydryl radical **III** to act as both an oxidant and a reductant to achieve photocatalyst turnover. This makes BecaP an ambiphilic radical trap that reacts with similar efficiency with nucleophilic and electrophilic radical precursors. In addition, the dual role of MeOH as a proton source for anion **V** and a nucleophilic trap for cation **VII** means that a single set of reaction conditions could be applicable to photoredox-catalyzed phosphonylations of a broad range of radical precursors.

**Scheme 3:**
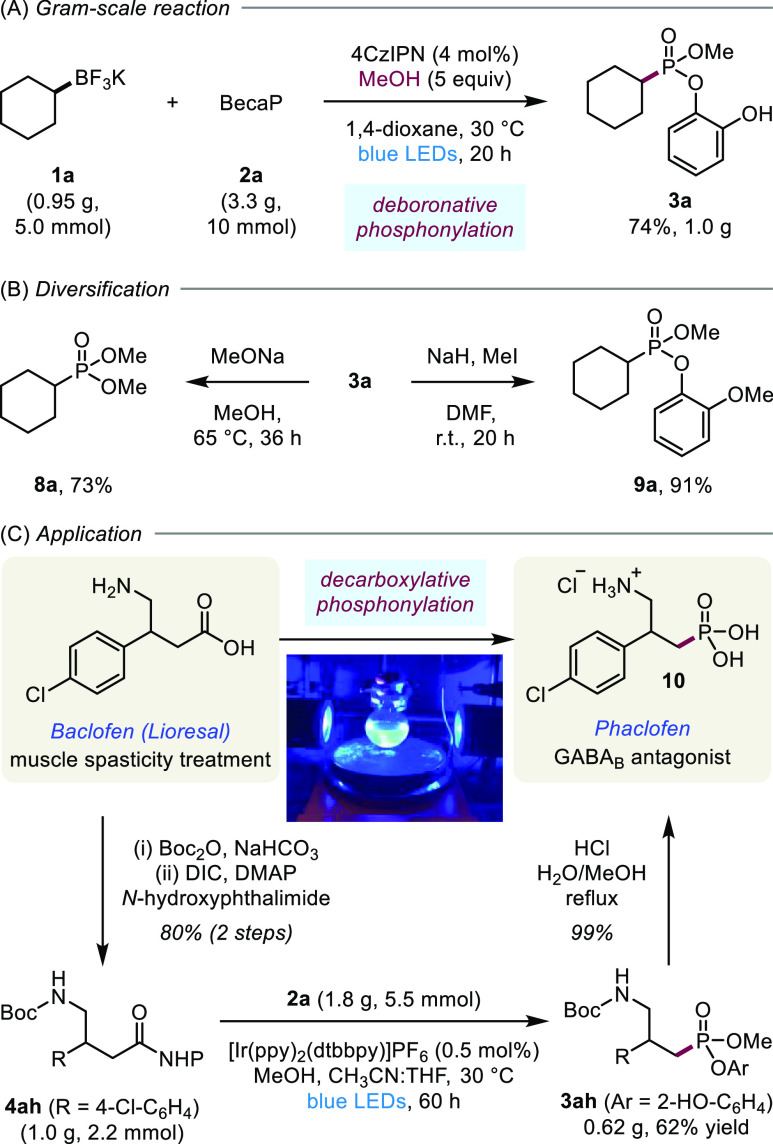
Gram-Scale Reactions, Product Diversification, and Application to the Synthesis of Phaclofen

To highlight the synthetic utility of the new phosphonylation methods, we investigated their scale up and diversification of the products (Scheme 3). The scalability of the deboronative reaction was shown through the phosphonylation of **1a** on gram scale in comparable yield to the small-scale reaction (Scheme 3A). As the pendant catechol is retained in the phosphonate product **3**, we investigated diversification of this phosphonate ester. Pleasingly, reaction with sodium methoxide in methanol delivers dimethyl phosphonate **8a** in good yield (Scheme 3B). Furthermore, reaction with methyl iodide and base resulted in the formation of the methoxy catechol derivative **9a** in excellent yield. We also demonstrated the scalability of the decarboxylative phosphonylation through the transformation of **4ah** into **3ah**, which was performed with a reduced photocatalyst loading of 0.5 mol % while maintaining similar yields (Scheme 3C). Importantly, product **3ah** could be transformed to the biologically active phaclofen **10** in excellent yield by simultaneous Boc-deprotection and hydrolysis of the phosphonate ester.^33^ Overall, the decarboxylative phosphonylation method could be employed to convert one biologically active molecule, baclofen,^34^ to another bioactive molecule, phaclofen,^35^ in 49% yield over four steps.

## Conclusions

In conclusion, we have developed a novel phosphorus radical trap, BecaP, that allows efficient phosphonylation of alkyl radicals under mild photoredox-catalyzed conditions. The synthetic utility of BecaP was demonstrated through the successful conversion of readily available alkyl potassium trifluoroborates and NHP esters of alkyl carboxylic acids into the corresponding phosphonate esters. BecaP can be easily accessed on a multigram scale, and the resulting photocatalytic reactions are scalable and provide moderate to excellent yields across a broad spectrum of primary and secondary alkyl substrates. Furthermore, the deboronative and decarboxylative protocols were both found to be suitable for the late-stage modification of complex natural products and drug molecules.

Notably, BecaP could be used for phosphonylations of electronically opposed alkyl radical precursors without additional stoichiometric oxidants or reductants. This was possible because of the ability of the benzhydryl radical leaving group to function as either a single-electron oxidant or reductant to turnover the photocatalytic cycles. Thus, the phosphonylations could proceed through two distinct reaction mechanisms, including a reductive quenching pathway with the nucleophilic trifluoroborates and an oxidative quenching pathway with the electrophilic NHP esters. We anticipate that this ambiphilic reactivity will enable BecaP to be used in a wide range of radical-mediated phosphonylations.

## Supporting Information Available

Experimental procedures, characterization data, X-ray crystallography details, and spectra (PDF)

## Dedication

This paper is dedicated to Professor Burkhard König (University of Regensburg, Germany) on the occasion of his 60th birthday.
