# Supplementary material for: Convergent Deboronative and Decarboxylative Phosphonylation Enabled by the Phosphite Radical Trap “BecaP”
Source: J Am Chem Soc. 2023 Aug 8;145(33):18649–57. doi: 10.1021/jacs.3c06524 (PMC10450818; doi:10.1021/jacs.3c06524)

SUPPORTING INFORMATION

Convergent Deboronative and Decarboxylative Phosphonylation Enabled by the Phosphite Radical Trap 'BecaP'

Santosh K. Pagire,[a] Chao Shu,[a,b] Dominik Reich,[a] Adam Noble,[a]* and Varinder K. Aggarwal[a]*

[a] School of Chemistry, University of Bristol, Cantock's Close, Bristol BS8 1TS, United Kingdom

[b] National Key Laboratory of Green Pesticide, College of Chemistry, Central China Normal University (CCNU), 152 Luoyu Road, Wuhan, Hubei 430079, China

*e-mail(s): a.noble@bristol.ac.uk; v.aggarwal@bristol.ac.uk

CONTENTS

1. MATERIALS AND GENERAL METHODS 2
1.1. Glassware, Solvents and Reagents 2
1.2. Chromatography and Instrumentation 2
1.3. Naming of Compounds 3
2. EXPERIMENTAL DATA 4
2.1. General Procedure A: Synthesis of alkyl potassium trifluoroborates 4
2.2. General Procedure B: Synthesis of alkyl boronic acid pinacol esters from carboxylic acids 4
2.3. General Procedure C: Synthesis of *N*-Hydroxyphthalimide (NHP) Esters 11
2.4. General Procedure D: Synthesis of phosphites 23
3. PHOTOCATALYSIS 28
3.1. Photochemical Equipment and Reaction Setup 28
3.2. Optimization of the Deboronative Phosphonylation reaction 29
3.2.1. Table S1: Optimization of phosphite sources 29
3.2.2. Table S2: Optimization of additives 30
3.2.3. Table S3: Optimization of MeOH loading 31
3.2.4. Table S4: Optimization of photocatalysts 32
3.2.5. Table S5: Optimization of Solvents 33
3.3. Optimization of the Decarboxylative Phosphonylation 34
3.3.1. Table S6: Optimization of phosphite equivalents 34
3.3.2. Table S7: Optimization of solvents 34
3.3.3. Table S8: Optimization of photocatalysts 35
3.4. General Procedure E: Deboronative Phosphonylation 36
3.5. General Procedure F: Decarboxylative Phosphonylation 36
4. PRODUCT CHARACTERIZATION 38
5. MECHANISTIC STUDY 70
5.1. Radical Clock Experiment 70
5.2. TEMPO Trapping Experiments 72
5.3. Confirmation of the Side Products 74
6. PRODUCT TRANSFORMATIONS 76
6.1. Gram Scale Conditions 76
6.2. Representative Transformations of 3a 77
6.3. Hydrolysis with HCl 79
7. REFERENCES 80
8. X-RAY CRYSTALLOGRAPHIC ANALYSIS 81
3l (CCDC number: 2216343) 81
9. NMR DATA 82

1. MATERIALS AND GENERAL METHODS

1.1. Glassware, Solvents and Reagents

All reactions were conducted under an inert atmosphere of nitrogen using standard Schlenk manifold techniques unless mentioned otherwise. All glassware was oven- and/or flame-dried prior to use.

All anhydrous solvents (acetone, CH_3CN, 1,4-dioxane, MeOH, DMF, DMSO, etc.) were commercially supplied (ACROS) or dried using an Anhydrous Engineering alumina column drying system (THF, Toluene, Et_2O, CH_2Cl_2) and stored over 4 Å mol sieves. All reagents were purchased from commercial sources [Sigma Aldrich (Merck), Across, Fischer, Fluorochem Ltd, TCI, etc.] and used as received. Irradiation of reaction mixtures was achieved using a 40 W Kessil A160WE LED – Tuna Blue light (setup: max blue, max intensity). Glass vials (10 mL) with PTFE/silicon septum lined caps were used as the standard reaction vessel for the photoreaction. De-ionised water was used for aq. workups. Brine refers to a saturated aqueous solution of NaCl. 4-CzIPN was prepared following the method of Zhang and co-workers.[1] [Ir(ppy)$_2$dtb-bpy]PF$_6$ was directly purchased from Sigma-Aldrich (Merck).

1.2. Chromatography and Instrumentation

Thin layer chromatography (TLC) was performed to monitor reactions when practical using Merck Kieselgel 60 F254 fluorescent treated silica, which was visualised under UV light, or by staining with aqueous basic potassium permanganate followed by heating.

Flash column chromatography (FCC) was carried out using Sigma-Aldrich silica gel (60 Å, 230–400 mesh, 40–63 μm) or a Biotage IsoleraTM flash purification system.

NMR spectra were recorded at various field strengths, as indicated, using Varian VNMR 400 MHz, Varian VNMR 500 MHz, or Bruker Cryo 500 MHz for ^{1}H, ^{13}C, ^{31}P, and ^{19}F acquisitions. All NMR spectra were recorded at ~25 °C in $CDCl_3$ unless otherwise stated. Chemical shifts (δ) are reported in parts per million (ppm) and referenced to $CDCl_3$ (^{1}H: 7.26 ppm; ^{13}C: 77.16 ppm). Coupling constants (*J*) are given in Hertz (Hz) and refer to corresponding multiplicities (bs = broad singlet/signal, s = singlet, d = doublet, t = triplet, q = quartet, quin = quintet, sex = sextet, h = heptet, m = multiplet, dd = doublet of doublets, etc.). The ^{1}H NMR spectra are reported as follows: chemical shift (multiplicity, coupling constants, number of protons, assignment). NMR assignments were made according to spin systems, using two-dimensional NMR spectroscopy (COSY, HSQC, HMBC) to assist the characterization. Where an assignment could not be made unambiguously, no assignments are given. NMR yields were determined by ^{1}H NMR analysis using 1,1,2,2-Tetrachloroethane (TCE) or diethyl phthalate as an internal standard.

High resolution mass spectra (HRMS) were recorded on a Bruker Daltonics MicrOTOF II by Electrospray Ionisation (ESI) or a Thermo Scientific Orbitrap Elite by ESI or Atmospheric Pressure Chemical Ionisation (APCI).

IR spectra were recorded neat as a thin film on a Perkin Elmer Spectrum One FT-IR. Selected absorption maxima (ν_{max}) are reported in wavenumbers (cm^{-1}).

Melting points were recorded in degrees Celsius (°C), using a Kofler hot-stage microscope apparatus and are reported uncorrected.

1.3. Naming of Compounds

Compound names are generated by ChemDraw Professional 20.0 software (PerkinElmer), following the IUPAC nomenclature.

2. EXPERIMENTAL DATA

2.1. General Procedure A: Synthesis of alkyl potassium trifluoroborates

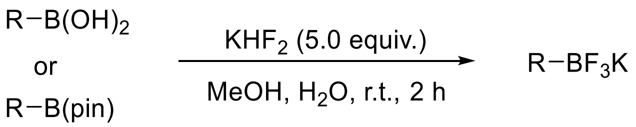

Alkyl trifluoroborates were prepared following a modified literature procedure.[2] To the solution of alkyl boronic acid or pinacol ester (1.0 equiv.) in methanol (0.5 M) was added saturated aqueous KHF_2 (5.0 equiv.) with ice/water bath. The resulting suspension was stirred for 2 h and then concentrated to dryness. The residue, a white solid, was extracted with hot acetone (3 ×30 mL), and the combined filtered extracts were concentrated to approximately 3 mL. Ether was added and the resultant precipitate was collected and dried to afford the potassium trifluoroborate as a white solid.

These trifluoroborates were purchased from commercial suppliers.

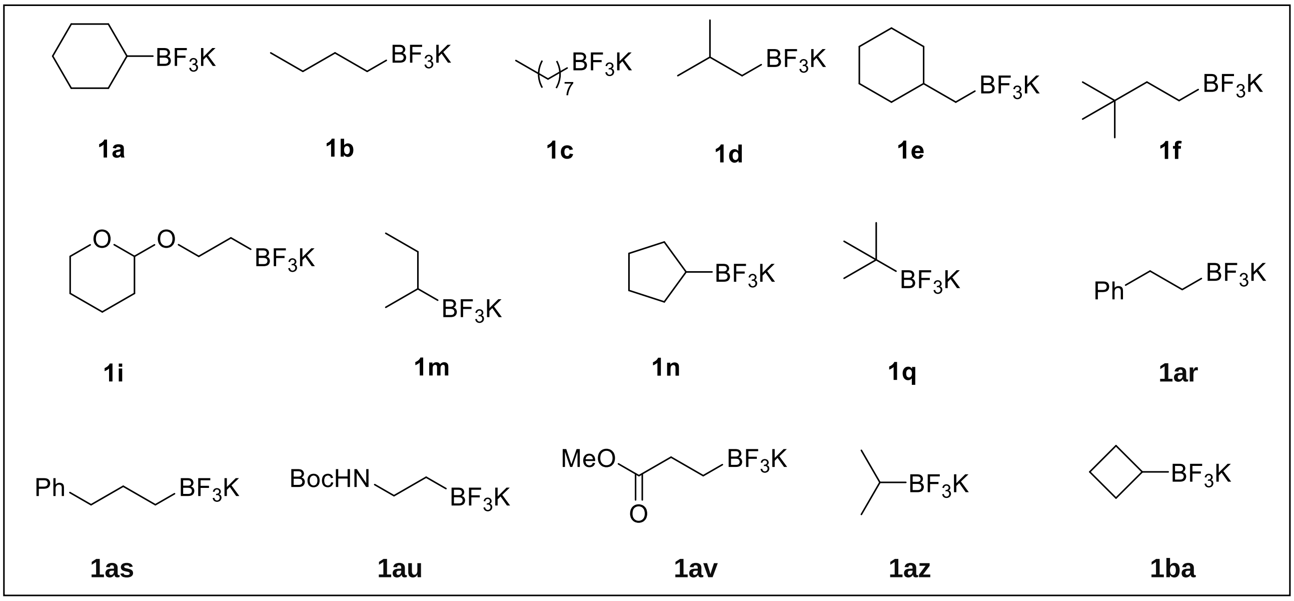

2.2. General Procedure B: Synthesis of alkyl boronic acid pinacol esters from carboxylic acids

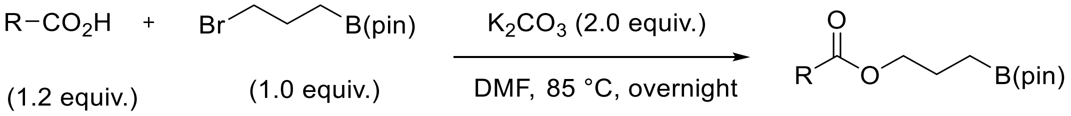

Alkyl boronic acid pinacol esters were prepared following a modified literature procedure.[3] To the solution of carboxylic acid (1.2 equiv.) in dry DMF (0.5 M) was added 3-bromopropylboronic acid pinacol ester (1.0 equiv.) and K_2CO_3 (2.0 equiv.). The reaction mixture was stirred vigorously for overnight at 85 ˚C. EtOAc (30 mL) was then added and allowed to stir for 10 minutes and filtered. The organic layer was washed with saturated aqueous bicarbonate, brine, dried over $MgSO_4$, and concentrated. Purification by silica-gel chromatography afforded pinacol boronic ester as a yellow oil or solid.

Potassium 3-(Trifluoroborato)propyl benzoate (1g)

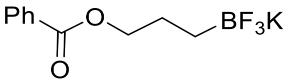

Following the **general procedure A**, the reaction of 3-(4,4,5,5-tetramethyl-1,3,2-dioxaborolan-2-yl)propyl benzoate (870.6 mg, 3.0 mmol) afforded trifluoroborate (**1g**) (730.0 mg, 2.7 mmol, 90%) as a white solid. All recorded spectroscopic data matched with those previously reported in the literature.[4]

^{1}H NMR (400 MHz, acetone-*d*$_6$): δ_H 8.04 – 8.00 (m, 2H), 7.62 – 7.57 (m, 1H), 7.52 – 7.46 (m, 2H), 4.23 (t, *J* = 7.3 Hz, 2H), 1.76 – 1.65 (m, 2H), 0.27 – 0.14 (m, 2H) ppm.

^{13}C NMR (101 MHz, acetone-*d*$_6$): δ_C 167.0, 133.5, 132.2, 130.2, 129.3, 69.3, 26.1 (d, *J* = 2.5 Hz) ppm. Note: *The carbon attached to boron could not be observed due to quadrupolar relaxation*.

Potassium (3-phenoxypropyl)trifluoroborate (1h)

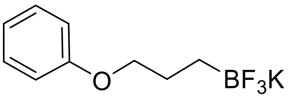

Following the **general procedure A**, the reaction of pinacol boronic ester (655.5 mg, 2.50 mmol) afforded trifluoroborate (**1h**) (508.4 mg, 2.10 mmol, 84%) as a white solid.

^{1}H NMR (400 MHz, dmso-*d*$_6$): δ_H 7.24 (t, *J* = 7.8 Hz, 2H), 6.92 – 6.83 (m, 3H), 3.84 (t, *J* = 7.4 Hz, 2H), 1.66 – 1.53 (m, 2H), 0.14 – -0.03 (m, 2H) ppm.

^{13}C NMR (101 MHz, dmso-*d*$_6$): δ_C 159.5, 129.8, 120.3, 114.8, 71.2, 25.8 ppm. *The carbon attached to boron could not be observed due to quadrupolar relaxation*.

^{19}F NMR (377 MHz, dmso-*d*$_6$): δ_F -137.1 ppm.

^{11}B NMR (128 MHz, dmso-*d*$_6$): δ_B 5.2 ppm.

M.p.: 286-288 ˚C

HRMS (ESI): calcd. for $C_9H_{11}BOF_3$ (M-K) 203.0855, found 203.0856.

Potassium 3-bromopropyltrifluoroborate (1j)

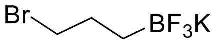

Following the **general procedure A**, the reaction of 3-bromopropylboronic acid pinacol ester (1.24 g, 5.0 mmol) afforded trifluoroborate (**1j**) (915 mg, 4.0 mmol, 80%) as a white solid. All recorded spectroscopic data matched those previously reported in the literature.[5]

^{1}H NMR (400 MHz, DMSO-*d*$_6$): δ_H 3.36 (t, *J* = 7.6 Hz, 2H), 1.70 – 1.57 (m, 2H), 0.04 – -0.07 (m, 2H) ppm.

^{13}C NMR (101 MHz, acetone-*d*$_6$): δ_C 38.4, 30.4 (q, *J* = 2.5 Hz) ppm. *The carbon attached to boron could not be observed due to quadrupolar relaxation*.

Potassium 3-nitrile trifluoroborate (1k)

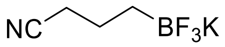

Following the **general procedure A**, the reaction of pinacol boronic ester (585.0 mg, 3.0 mmol) afforded trifluoroborate (**1k**) (315.0 mg, 1.80 mmol, 60%) as a white solid.

^{1}H NMR (400 MHz, acetone-*d*$_6$): δ$_H$ 2.25 (t, *J* = 7.3 Hz, 2H), 1.56 – 1.47 (m, 2H), 0.27 – 0.14 (m, 2H) ppm.

^{13}C NMR (101 MHz, acetone-*d*$_6$): δ$_C$ 120.7, 22.1, 18.6 ppm. *The carbon attached to boron could not be observed due to quadrupolar relaxation*.

^{19}F NMR (377 MHz, acetone-*d*$_6$): δ$_F$ -141.4 ppm.

^{11}B NMR (128 MHz, acetone-*d*$_6$): δ$_B$ 5.0 ppm.

M.p.: 147-148 ˚C

HRMS (ESI): calcd. for $C_4H_6BNF_3$ (M-K) 136.0545, found 136.0543.

Potassium 2-(9*H*-carbazol-9-yl)ethyltrifluoroborate (1l)

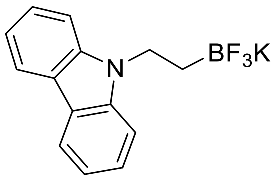

Following the **general procedure A**, the reaction of 2-(9-carbazolyl)ethylboronic acid pinacol ester (642.4 mg, 2.0 mmol) afforded trifluoroborate (**1o**) (271.0 mg, 1.8 mmol, 90%) as a white solid. All recorded spectroscopic data matched those previously reported in the literature.[6]

^{1}H NMR (400 MHz, acetone-*d*$_6$): δ$_H$ 8.09 (dt, *J* = 7.8, 1.0 Hz, 2H), 7.49 (d, *J* = 8.3 Hz, 2H), 7.41 – 7.36 (m, 2H), 7.16 – 7.10 (m, 2H), 4.41 – 4.33 (m, 2H), 0.84 – 0.72 (m, 2H) ppm.

^{13}C NMR (101 MHz, acetone-*d*$_6$): δ$_C$ 139.9, 124.7, 122.2, 119.5, 117.5, 108.7, 40.9 (d, *J* = 3.1 Hz) ppm. *The carbon attached to boron could not be observed due to quadrupolar relaxation*.

Potassium (bicyclo[2.2.1]heptan-2-yl)trifluoroborate (1o)

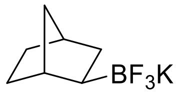

Following the **general procedure A**, the reaction of corresponding boronic acid pinacol ester (444.2 mg, 2.0 mmol) afforded trifluoroborate (**1o**) (320.0 mg, 1.58 mmol, 79%) as a white solid. All recorded spectroscopic data matched those previously reported in the literature.[7]

^{1}H NMR (400 MHz, dmso-*d*$_6$): δ$_H$ 1.92 (s, 1H), 1.88 (s, 1H), 1.35 – 1.23 (m, 3H), 1.25 – 1.14 (m, 1H), 1.02 – 0.85 (m, 3H), 0.76 – 0.69 (m, 1H), 0.06 – -0.06 (m, 1H) ppm.

^{13}C NMR (101 MHz, dmso-*d*$_6$): δ$_C$ 38.6, 37.1, 36.7, 33.8, 33.6, 30.0 ppm. *The carbon attached to boron could not be observed due to quadrupolar relaxation*.

Potassium 2-adamantane trifluoroborate (1p)

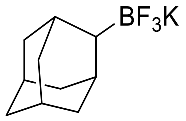

Following the **general procedure A**, the reaction of pinacol boronic ester (262.2 mg, 1.0 mmol) afforded trifluoroborate (**1p**) (181.6 mg, 0.75 mmol, 75%) as a white solid.

^{1}H NMR (400 MHz, acetone-*d*$_6$): δ_H 1.73 – 1.66 (m, 3H), 1.65 – 1.59 (m, 6H), 1.47 – 1.43 (m, 6H) ppm.

^{13}C NMR (101 MHz, acetone-*d*$_6$): δ_C 39.1, 38.8, 28.5 ppm. *The carbon attached to boron could not be observed due to quadrupolar relaxation*.

^{19}F NMR (377 MHz, acetone-*d*$_6$): δ_F -153.0 ppm.

^{11}B NMR (128 MHz, acetone-*d*$_6$): δ_B 4.2 ppm.

HRMS (ESI): calcd. for $C_{10}H_{15}BF_3$ (M-K) 203.1219, found 203.1221.

Potassium propyl 2-(4-isobutylphenyl)propanoate 3-trifluoroborate (1r)

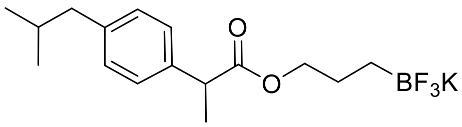

Following the **general procedure A**, the reaction of pinacol boronic ester (748.6 mg, 2.0 mmol) afforded trifluoroborate (**1r**) (496.0 mg, 1.40 mmol, 70%) as a white solid.

^{1}H NMR (400 MHz, acetone-*d*$_6$): δ_H 7.19 – 7.15 (m, 2H), 7.11 – 7.07 (m, 2H), 3.94 – 3.81 (m, 2H), 3.68 (q, *J* = 7.1 Hz, 1H), 2.41 (d, *J* = 7.1 Hz, 2H), 1.87 – 1.75 (m, 1H), 1.43 – 1.32 (m, 5H), 0.85 (d, *J* = 6.6 Hz, 6H), -0.04 – -0.16 (m, 2H) ppm.

^{13}C NMR (101 MHz, acetone-*d*$_6$): δ_C 174.5, 140.1, 138.7, 129.5, 127.5, 68.3, 44.8, 44.7, 30.0, 25.3, 22.7, 19.1 ppm. *The carbon attached to boron could not be observed due to quadrupolar relaxation*.

^{19}F NMR (377 MHz, acetone-*d*$_6$): δ_F -137.3 ppm.

^{11}B NMR (128 MHz, acetone-*d*$_6$): δ_B 4.9 ppm.

M.p.: 87-88 ˚C

HRMS (ESI): calcd. for $C_{16}H_{23}O_2BNF_3$ (M-K) 315.1743, found 315.1749.

Potassium 2-(1-methyl-1*H*-indol-3-yl)acetate trifluoroborate (1s)

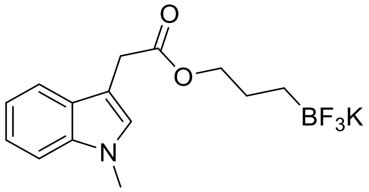

Following the **general procedure B**, (1-methyl-2,3-dihydro-1*H*-indol-3-yl)acetic acid (0.42 g, 3.0 mmol) afforded

the corresponding pinacol boronic ester as a yellow oil (0.50 g, 1.40 mmol, 70%).

Following the **general procedure A**, the reaction of pinacol boronic ester (357.3 mg, 1.0 mmol) afforded trifluoroborate (**1s**) (240.0 mg, 0.71 mmol, 71%) as a white solid.

^{1}H NMR (400 MHz, dmso-d_6): δ_H 7.51 (d, *J* = 7.9 Hz, 1H), 7.39 (d, *J* = 8.2 Hz, 1H), 7.22 (s, 1H), 7.15 (t, *J* = 7.6 Hz, 1H), 7.03 (t, *J* = 7.4 Hz, 1H), 3.92 (t, *J* = 7.6 Hz, 2H), 3.74 (s, 3H), 3.69 (s, 2H), 1.51 – 1.40 (m, 2H), 0.03 – -0.11 (m, 2H) ppm.

^{13}C NMR (101 MHz, dmso-d_6): δ_C 172.0, 137.0, 128.7, 127.9, 121.6, 119.2, 119.1, 110.1, 107.1, 68.3, 32.7, 31.2, 25.4 ppm. *The carbon attached to boron could not be observed due to quadrupolar relaxation*.

^{19}F NMR (377 MHz, acetone-d_6): δ_F -137.3 ppm.

^{11}B NMR (128 MHz, acetone-d_6): δ_B 4.9 ppm.

M.p.: 94-95 ˚C

HRMS (ESI): calcd. for $C_{14}H_{16}NO_2BF_3$ (M-K) 298.1226, found 298.1216.

Potassium (1*R*,4*S*)-4,7,7-trimethyl-3-oxo-2-oxabicyclo[2.2.1]heptane-1-carboxylatetrifluoroborate (1t)

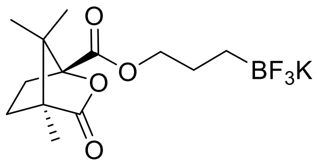

Following the **general procedure A**, the reaction of pinacol boronic ester (439.5 mg, 1.20 mmol) afforded trifluoroborate (**1t**) (200.0 mg, 0.58 mmol, 48%) as a white solid.

^{1}H NMR (400 MHz, acetone-d_6): δ_H 4.16 – 4.01 (m, 2H), 2.46 – 2.34 (m, 1H), 1.98 – 1.84 (m, 2H), 1.62 – 1.49 (m, 3H), 1.03 (s, 3H), 1.00 (s, 3H), 0.86 (s, 3H), 0.16 – 0.03 (m, 2H) ppm.

^{13}C NMR (101 MHz, acetone-d_6): δ_C 177.4, 166.8, 90.7, 68.4, 54.1, 53.2, 30.0, 28.4, 24.7, 24.0, 15.8, 15.8, 8.8 ppm. *The carbon attached to boron could not be observed due to quadrupolar relaxation*.

^{19}F NMR (377 MHz, acetone-d_6): δ_F -141.4 ppm.

^{11}B NMR (128 MHz, acetone-d_6): δ_B 5.3 ppm.

M.p.: 201-203 ˚C

HRMS (ESI): calcd. for $C_{13}H_{19}BO_4F_3$ (M-K) 307.1328, found 307.1329.

Potassium 3-(trifluoroborato)propyl (4*R*)-4-((8*R*,9*S*,10*S*,13*R*,14*S*,17*R*)-10,13-dimethyl-3,7,12-trioxohexadecahydro-1*H*-cyclopenta[a]phenanthren-17-yl)pentanoate (1u)

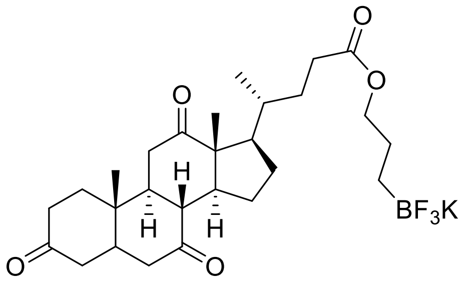

Following **general procedure B**, dehydrocholic acid (1.21 g, 3.0 mmol) afforded corresponding pinacol boronic ester as a yellow oil (1.11 g, 1.94 mmol, 78%).

Following the **general procedure A**, the reaction of pinacol boronic ester (570.6 mg, 1.0 mmol) afforded trifluoroborate (**1u**) (467.0 mg, 0.85 mmol, 85%) as a white solid.

^{1}H NMR (400 MHz, dmso-*d*$_6$): δ_H 3.86 (t, *J* = 7.6 Hz, 2H), 3.10 – 2.94 (m, 2H), 2.84 (t, *J* = 12.7 Hz, 1H), 2.50 (p, *J* = 1.9 Hz, 2H), 2.45 (dd, *J* = 12.6, 5.0 Hz, 1H), 2.38 – 2.07 (m, 6H), 2.01 – 1.90 (m, 3H), 1.89 – 1.79 (m, 4H), 1.80 – 1.64 (m, 2H), 1.49 (td, *J* = 14.5, 4.1 Hz, 1H), 1.46 – 1.34 (m, 2H), 1.33 (s, 3H), 1.32 – 1.14 (m, 4H), 1.01 (s, 3H), 0.76 (d, *J* = 5.9 Hz, 3H), -0.02 – -0.16 (m, 2H) ppm.

^{13}C NMR (101 MHz, dmso-*d*$_6$): δ_C 212.4, 210.1, 210.0, 173.7, 67.8, 56.7, 51.6, 48.4, 46.5, 45.9, 45.0, 44.5, 43.0, 38.8, 36.6, 36.1, 35.4, 35.1, 31.7, 30.9, 27.7, 25.5, 25.4, 25.1, 21.6, 19.1, 11.9 ppm. *The carbon attached to boron could not be observed due to quadrupolar relaxation*.

^{19}F NMR (377 MHz, dmso-*d*$_6$): δ_F -137.3 ppm.

^{11}B NMR (128 MHz, dmso-*d*$_6$): δ_B 4.5 ppm.

M.p.: 227-228 ˚C

HRMS (ESI): calcd. for $C_{27}H_{39}BO_5F_3$ (M-K) 511.2843, found 511.2847.

Potassium 1-Propenyltrifluoroborate (1at)

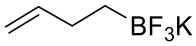

Following the **general procedure A**, the reaction of corresponding boronic acid (219.7 mg, 3.0 mmol) afforded trifluoroborate (**1at**) (374.2 mg, 2.31 mmol, 77%) as a white solid. All recorded spectroscopic data matched those previously reported in the literature.[8]

^{1}H NMR (400 MHz, acetone-*d*$_6$): δ_H 6.02 – 5.89 (m, 1H), 4.95 – 4.86 (m, 1H), 4.78 – 4.71 (m, 1H), 2.07 – 1.98 (m, 2H), 0.35 – 0.22 (m, 2H) ppm.

^{13}C NMR (101 MHz, acetone-*d*$_6$): δ_C 144.2, 109.6, 29.5 ppm. *The carbon attached to boron could not be observed due to quadrupolar relaxation*.

Potassium 4-(Trifluoroborato)tetrahydropyran (1bb)

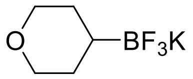

Following the **general procedure A**, the reaction of tetrahydropyran-4-boronic acid pinacol ester (636.3 mg, 3.0 mmol) afforded trifluoroborate (**1bb**) (524.2 mg, 2.73 mmol, 91%) as a white solid. All recorded spectroscopic data matched those previously reported in the literature.[9]

^{1}H NMR (400 MHz, dmso-*d*$_6$): δ_H 3.77 – 3.71 (m, 2H), 3.12 (td, *J* = 10.9, 3.3 Hz, 2H), 1.31 – 1.14 (m, 4H), 0.25 – 0.13 (m, 1H) ppm.

^{13}C NMR (101 MHz, dmso-*d*$_6$): δ_C 69.8, 29.4 (d, *J* = 2.0 Hz) ppm. *The carbon attached to boron could not be observed due to quadrupolar relaxation*.

Potassium *N*-Boc-4-(trifluoroborato)piperidine (1bc)

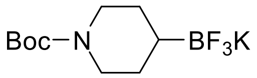

Following the **general procedure A**, the reaction of *N*-Boc-piperidine-4-boronic acid pinacol ester (0.62 g, 2.0 mmol) afforded trifluoroborate (**1bc**) (524.0 mg, 1.80 mmol, 90%) as a white solid. All recorded spectroscopic data matched those previously reported in the literature.[9]

^{1}H NMR (400 MHz, DMSO-*d*$_6$): δ_H 3.84 (d, *J* = 12.4 Hz, 2H), 2.57 – 2.36 (m, 2H), 1.37 (s, 9H), 1.08 – 0.93 (m, 2H), 0.17 – 0.04 (m, 1H) ppm.

^{13}C NMR (101 MHz, DMSO-*d*$_6$): δ_C 154.5, 78.1, 28.7, 28.5 ppm. *The carbon attached to boron could not be observed due to quadrupolar relaxation*.

Potassium 5-hexenyl-1-trifluoroborate (1bd)

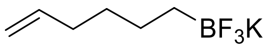

Following the **general procedure A**, the reaction of corresponding boronic acid (384.0 mg, 3.0 mmol) afforded trifluoroborate (**1bd**) (410.0 mg, 2.16 mmol, 72%) as a white solid. All recorded spectroscopic data matched those previously reported in the literature.[10]

^{1}H NMR (400 MHz, MeOD): δ_H 5.87 – 5.75 (m, 1H), 4.98 – 4.83 (m, 2H), 2.09 – 1.97 (m, 2H), 1.41 – 1.22 (m, 4H), 0.28 – 0.15 (m, 2H) ppm.

^{13}C NMR (101 MHz, MeOD): δ_C 141.0, 113.9, 35.3, 34.0, 26.0 ppm. *The carbon attached to boron could not be observed due to quadrupolar relaxation*.

2.3. General Procedure C: Synthesis of *N*-Hydroxyphthalimide (NHP) Esters

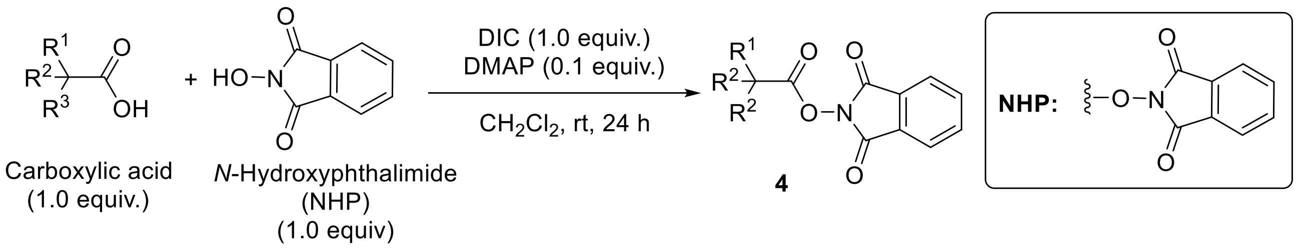

The NHP esters were prepared according to a literature known procedure[11]. *N*-Hydroxyphthalimide (1.0 eq.), DMAP (0.1 eq.) and, if solid, carboxylic acid (1.0 eq.) was added to a round-bottomed flask. Dichloromethane (0.1 M) and, if liquid, carboxylic acid (1.0 eq) was then added, followed by *N*,*N'*-Diisopropylcarbodiimide (DIC) (1.0 eq.). The reaction mixture was allowed to stir at room temperature for 24 h. Saturated $NaHCO_3$ was then added to the reaction mixture and extracted with dichloromethane (30 mL x 3) before concentrating under reduced pressure. The crude residue was directly purified by flash-column chromatography (SiO_2; typically, DCM with either pentane or Et_2O) to yield pure NHP ester. If solid and not pure, the NHP ester can be recrystallized from DCM/MeOH or EtOAc or DCM/hexane to give pure material.

Notes: Unless stated, no precautions were taken throughout the syntheses of the NHP esters. No attempts were made to optimize for yield as the procedure (GP-C) provide the RAEs in good to excellent yields (typically >80%). Ethyl acetate with hexane/pentane/petroleum ether or DCM with pentane/Et_2O as co-solvent, was found to be the best eluent for purification by flash-column chromatography. Sometimes impurities co-elute with the product when using DCM, but this can be avoided by using pentane/hexane instead. All reagents were commercially bought and used without purification. Ethanol-free DCM should be used to prevent the formation of the ethyl ester.

The data of the obtained RAEs (**4**) is in accordance with the literature data.[11]

1,3-Dioxoisoindolin-2-yl cyclohexanecarboxylate (4a)

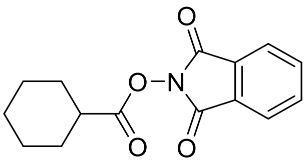

^{1}H NMR (400 MHz, $CDCl_3$): δ_H 7.87 (dd, *J* = 5.5, 3.1 Hz, 2H), 7.77 (dd, *J* = 5.5, 3.1 Hz, 2H), 2.73 (tt, *J* = 10.9, 3.7 Hz, 1H), 2.16 – 2.04 (m, 2H), 1.90 – 1.78 (m, 2H), 1.72 – 1.58 (m, 3H), 1.48 – 1.24 (m, 3H) ppm.

^{13}C NMR (101 MHz, $CDCl_3$): δ_C 171.9, 162.2, 134.8, 129.1, 124.0, 40.6, 28.9, 25.6, 25.1 ppm.

1,3-Dioxoisoindolin-2-yl 3-(4-chlorophenyl)propanoate (4v)

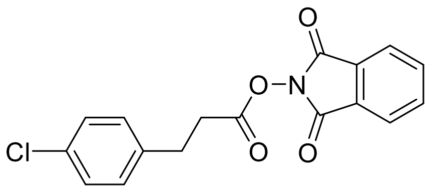

^{1}H NMR (400 MHz, $CDCl_3$): δ_H 7.96 – 7.89 (m, 2H), 7.87 – 7.80 (m, 2H), 7.38 – 7.31 (m, 2H), 7.28 – 7.22 (m, 2H), 3.16 – 3.09 (m, 2H), 3.02 (ddd, *J* = 8.6, 7.0, 1.2 Hz, 2H) ppm.

^{13}C NMR (101 MHz, $CDCl_3$): δ_C 168.8, 161.9, 137.6, 134.9, 132.6, 129.8, 128.9, 128.9, 124.0, 32.6, 29.9 ppm.

1,3-Dioxoisoindolin-2-yl 3-(4-bromophenyl)propanoate (4w)

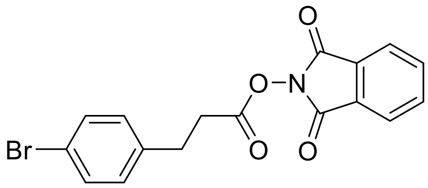

^{1}H NMR (400 MHz, $CDCl_3$): δ_H 7.89 (dd, *J* = 5.5, 3.1 Hz, 2H), 7.84 – 7.75 (m, 2H), 7.48 – 7.42 (m, 2H), 7.18 – 7.12 (m, 2H), 3.06 (dd, *J* = 8.0, 6.1 Hz, 2H), 3.00 – 2.93 (m, 2H) ppm.

^{13}C NMR (101 MHz, $CDCl_3$): δ_C 168.8, 162.0, 138.2, 134.9, 131.9, 130.2, 129.0, 124.1, 120.8, 32.6, 30.1 ppm.

1,3-Dioxoisoindolin-2-yl 3-cyclopentylpropanoate (4x)

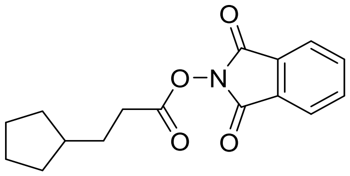

^{1}H NMR (400 MHz, $CDCl_3$): δ_H 7.92 – 7.85 (m, 2H), 7.82 – 7.76 (m, 2H), 2.72 – 2.63 (m, 2H), 1.96 – 1.77 (m, 5H), 1.70 – 1.50 (m, 4H), 1.22 – 1.06 (m, 2H) ppm.

^{13}C NMR (101 MHz, $CDCl_3$): δ_C 169.9, 162.1, 134.8, 129.1, 124.1, 39.6, 32.5, 30.9, 30.5, 25.3 ppm.

1,3-dioxoisoindolin-2-yl 3,3-dimethylbutanoate (4y)

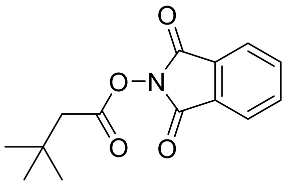

^{1}H NMR (500 MHz, $CDCl_3$): δ_H 7.88 (dd, *J* = 5.4, 3.1 Hz, 2H), 7.78 (dd, *J* = 5.5, 3.1 Hz, 2H), 2.52 (s, 2H), 1.16 (s, 9H) ppm.

^{13}C NMR (126 MHz, $CDCl_3$): δ_C 168.0, 162.2, 134.8, 129.1, 124.0, 44.7, 31.4, 29.6 ppm.

1,3-Dioxoisoindolin-2-yl 3-(thiophen-2-yl)propanoate (4z)

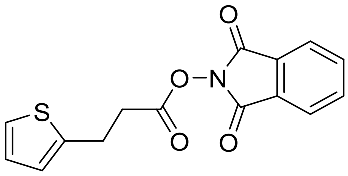

^{1}H NMR (400 MHz, $CDCl_3$): δ_H 7.93 – 7.85 (m, 2H), 7.82 – 7.76 (m, 2H), 7.17 (dd, *J* = 5.1, 1.2 Hz, 1H), 6.96 (dd, *J* = 5.1, 3.4 Hz, 1H), 6.92 (dq, *J* = 3.4, 1.0 Hz, 1H), 3.35 – 3.27 (m, 2H), 3.08 – 3.03 (m, 2H) ppm.

^{13}C NMR (101 MHz, $CDCl_3$): δ_C 168.6, 162.0, 141.5, 134.9, 129.0, 127.2, 125.3, 124.1, 33.1, 24.9 ppm.

1,3-Dioxoisoindolin-2-yl 5-bromopentanoate (4aa)

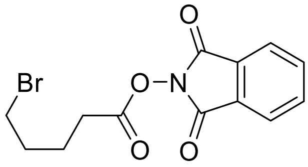

^{1}H NMR (400 MHz, $CDCl_3$): δ_H 7.93 – 7.85 (m, 2H), 7.83 – 7.76 (m, 2H), 3.46 (t, *J* = 6.3 Hz, 2H), 2.72 (t, *J* = 6.9 Hz, 2H), 2.10 – 1.90 (m, 4H) ppm.

^{13}C NMR (101 MHz, $CDCl_3$): δ_C 169.2, 162.1, 134.9, 129.1, 124.1, 32.7, 31.5, 30.2, 23.4 ppm.

1,3-Dioxoisoindolin-2-yl hex-5-ynoate (4ab)

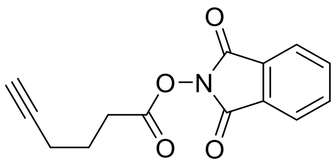

^{1}H NMR (400 MHz, $CDCl_3$): δ_H 7.89 (dd, *J* = 5.5, 3.1 Hz, 2H), 7.82 – 7.76 (m, 2H), 2.83 (t, *J* = 7.4 Hz, 2H), 2.38 (td, *J* = 6.9, 2.7 Hz, 2H), 2.09 – 1.92 (m, 3H) ppm.

^{13}C NMR (101 MHz, $CDCl_3$): δ_C 169.3, 162.0, 134.9, 129.1, 124.1, 82.6, 69.9, 29.8, 23.5, 17.8 ppm.

1,3-Dioxoisoindolin-2-yl 2,2,3,3-tetramethylcyclopropane-1-carboxylate (4ac)

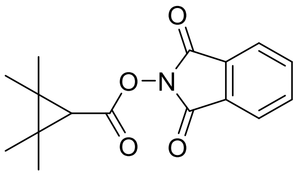

^{1}H NMR (400 MHz, $CDCl_3$): δ_H 7.86 (dd, *J* = 5.5, 3.1 Hz, 2H), 7.79 – 7.73 (m, 2H), 1.52 (s, 1H), 1.29 (s, 6H), 1.26 (s, 7H) ppm.

^{13}C NMR (101 MHz, $CDCl_3$): δ_C 167.8, 162.6, 134.7, 129.2, 123.9, 33.3, 32.4, 23.4, 16.5 ppm.

1,3-Dioxoisoindolin-2-yl 2,3-dihydro-1H-indene-2-carboxylate (4ad)

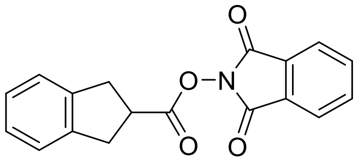

^{1}H NMR (400 MHz, $CDCl_3$): δ_H 7.90 (dd, *J* = 5.5, 3.1 Hz, 2H), 7.79 (dd, *J* = 5.5, 3.1 Hz, 2H), 7.25 (d, *J* = 5.7 Hz, 2H), 7.22 – 7.17 (m, 2H), 3.78 – 3.66 (m, 1H), 3.54 – 3.37 (m, 4H) ppm.

^{13}C NMR (101 MHz, $CDCl_3$): δ_C 171.8, 162.1, 140.7, 134.9, 129.1, 127.1, 124.6, 124.1, 40.6, 36.4 ppm.

1,3-Dioxoisoindolin-2-yl 2,2-dimethylhexanoate (4ae)

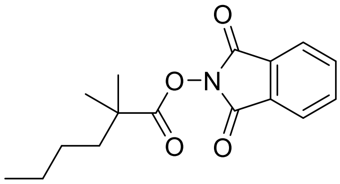

^{1}H NMR (400 MHz, $CDCl_3$): δ_H 7.88 (dd, *J* = 5.5, 3.1 Hz, 2H), 7.81 – 7.75 (m, 2H), 1.76 – 1.68 (m, 2H), 1.56 (q, *J* = 2.2 Hz, 1H), 1.48 – 1.31 (m, 9H), 0.95 (t, *J* = 7.1 Hz, 3H) ppm.

^{13}C NMR (101 MHz, $CDCl_3$): δ_C 174.1, 162.3, 134.8, 129.2, 124.0, 42.3, 40.5, 27.0, 25.2, 23.2, 14.0 ppm.

1-(*tert*-Butyl) 4-(1,3-dioxoisoindolin-2-yl) (((9H-fluoren-9-yl)methoxy)carbonyl)-L-aspartate (4af)

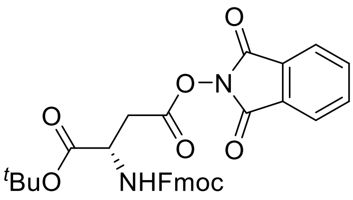

^{1}H NMR (400 MHz, $CDCl_3$): δ_H 7.90 (qd, *J* = 4.5, 2.8 Hz, 2H), 7.81 (td, *J* = 5.3, 2.1 Hz, 2H), 7.76 (d, *J* = 7.5 Hz, 2H), 7.64 (d, *J* = 7.5 Hz, 2H), 7.39 (t, *J* = 7.5 Hz, 2H), 7.32 (tt, *J* = 7.3, 1.4 Hz, 2H), 5.92 (d, *J* = 7.9 Hz, 1H), 4.69 (dt, *J* = 8.4, 4.5 Hz, 1H), 4.40 (qd, *J* = 10.5, 7.4 Hz, 2H), 4.27 (t, *J* = 7.3 Hz, 1H), 3.33 (qd, *J* = 17.0, 4.6 Hz, 2H), 1.51 (s, 9H) ppm.

^{13}C NMR (101 MHz, $CDCl_3$): δ_C 168.5, 167.4, 161.7, 156.0, 143.9, 143.9, 141.4, 135.0, 129.0, 127.8, 127.2, 127.2, 125.4, 125.4, 124.2, 120.1, 83.8, 67.6, 50.8, 47.2, 34.4, 27.9 ppm.

1-Benzyl 5-(1,3-dioxoisoindolin-2-yl) (*tert*-butoxycarbonyl)-L-glutamate (4ag)

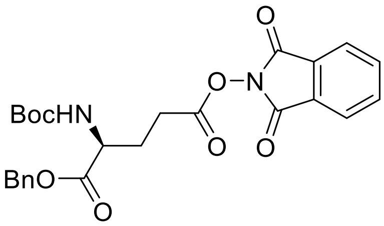

^{1}H NMR (400 MHz, $CDCl_3$): δ_H 7.91 – 7.85 (m, 2H), 7.82 – 7.76 (m, 2H), 7.41 – 7.30 (m, 5H), 5.20 (s, 2H), 4.45 (bs, 1H), 2.74 (qdd, *J* = 17.0, 9.3, 6.2 Hz, 2H), 2.43 – 2.03 (m, 2H), 1.44 (s, 9H) ppm.

^{13}C NMR (101 MHz, $CDCl_3$): δ_C 171.6, 168.9, 161.9, 155.5, 135.2, 134.9, 129.0, 128.8, 128.7, 128.5, 124.1, 80.4, 67.6, 52.8, 28.4, 27.8, 27.4 ppm.

1,3-Dioxoisoindolin-2-yl 4-((*tert*-butoxycarbonyl)amino)-3-(4-chlorophenyl)butanoate (4ah)

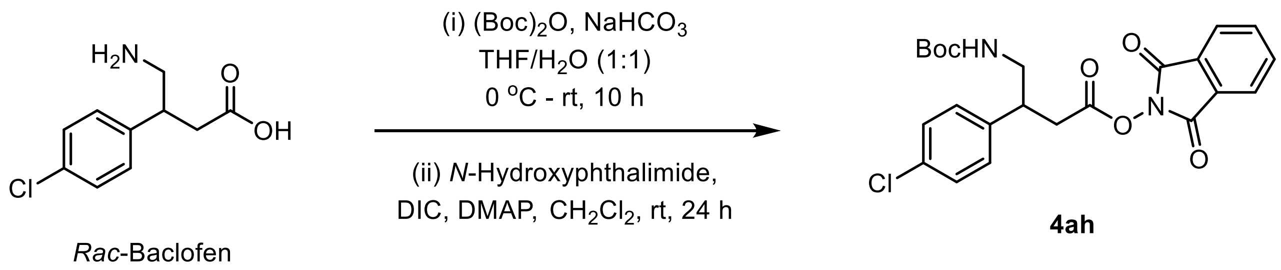

Rac-Baclofen (2.5 g, 11.73 mmol, 1.0 eq.), $(Boc)_2O$ (4.0 g, 17.6 mmol, 1.5 eq.), and K_2CO_3 (5.0 g, 35.2 mmol, 3.0 eq.) was added to a round-bottomed flask. THF (15 mL) and water (15 mL) was then added to the reaction mixture at 0 °C. The reaction mixture was then allowed to stir at room temperature for 10 h. After TLC analysis, the crude reaction mixture extracted with ethyl acetate (~30 mL x 3) before concentrating under reduced pressure. The crude residue was characterized by ^{1}H-NMR and LC-MS and directly subjected under **GP-C**

conditions to yield crude **4ah**, which was purified by EtOAc/pentane column followed by DCM/hexane recrystallization technique to afford pure **4ah** (4.3 g, 9.3 mmol, 80% yield after two steps).

^{1}H NMR (400 MHz, $CDCl_3$): δ_H δ 7.86 (dd, *J* = 5.5, 3.1 Hz, 2H), 7.77 (dd, *J* = 5.5, 3.1 Hz, 2H), 7.36 – 7.31 (m, 2H), 7.22 (d, *J* = 8.3 Hz, 2H), 4.71 (bs, 1H), 3.69 – 3.26 (m, 3H), 3.12 – 2.88 (m, 2H), 1.41 (s, 9H) ppm.

^{13}C NMR (101 MHz, $CDCl_3$): δ_C 167.9, 162.5, 161.8, 155.9, 151.8, 138.6, 136.3, 134.9, 134.8, 133.4, 131.4, 129.2, 129.1, 124.1, 116.8 d, *J* = 2.8 Hz), 79.8, 45.5, 42.0, 35.1, 28.4 ppm.

1,3-Dioxoisoindolin-2-yl 4-(4-(bis(2-chloroethyl)amino)phenyl)butanoate (4ai)

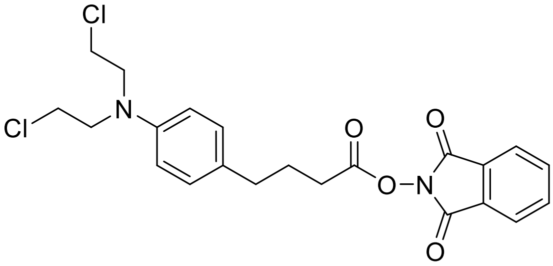

^{1}H NMR (400 MHz, $CDCl_3$): δ_H 7.91 – 7.85 (m, 2H), 7.82 – 7.75 (m, 2H), 7.15 – 7.09 (m, 2H), 6.68 – 6.62 (m, 2H), 3.71 (ddd, *J* = 8.2, 6.8, 1.9 Hz, 4H), 3.63 (ddd, *J* = 8.2, 6.8, 1.9 Hz, 4H), 2.71 – 2.62 (m, 4H), 2.12 – 2.00 (m, 2H) ppm.

^{13}C NMR (101 MHz, $CDCl_3$): δ_C 169.6, 162.1, 144.6, 134.8, 129.9, 129.9, 129.0, 124.0, 112.4, 53.7, 40.6, 33.5, 30.2, 26.6 ppm.

1,3-Dioxoisoindolin-2-yl 2-((1*R*,3*R*)-3-acetyl-2,2-dimethylcyclobutyl)acetate (4aj)

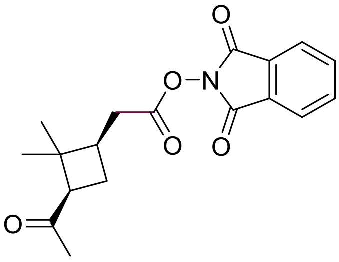

^{1}H NMR (400 MHz, $CDCl_3$): δ_H 7.88 – 7.84 (m, 2H), 7.79 – 7.76 (m, 2H), 2.92 (dd, *J* = 10.1, 7.6 Hz, 1H), 2.72 – 2.57 (m, 2H), 2.50 (dtd, *J* = 10.3, 8.4, 6.8 Hz, 1H), 2.14 – 1.98 (m, 5H), 1.37 (s, 3H), 0.95 (s, 3H) ppm.

^{13}C NMR (101 MHz, $CDCl_3$): δ_C 207.2, 168.8, 162.0, 134.9, 129.0, 124.1, 54.1, 43.5, 38.0, 32.0, 30.3, 30.2, 23.0, 17.40 ppm.

1,3-Dioxoisoindolin-2-yl 2-(1-(((tert-butoxycarbonyl)amino)methyl)cyclohexyl)acetate (4ak)

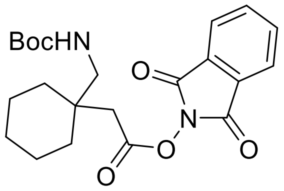

^{1}H NMR (400 MHz, $CDCl_3$): δ_H δ 7.86 (dd, J = 5.5, 3.1 Hz, 2H), 7.78 (dd, J = 5.5, 3.1 Hz, 2H), 4.95 (bs, 1H), 3.26 (d, J = 6.9 Hz, 2H), 2.61 (s, 2H), 1.65 – 1.56 (m, 2H), 1.55 – 1.44 (m, 8H), 1.41 (s, 9H) ppm.

^{13}C NMR (101 MHz, $CDCl_3$): δ_C δ 168.3, 162.1, 156.5, 135.0, 134.9, 129.0, 124.1, 79.2, 46.8, 39.0, 37.8, 33.8, 28.5, 25.9, 21.6 ppm.

1,3-Dioxoisoindolin-2-yl (*E*)-6-(4-hydroxy-6-methoxy-7-methyl-3-oxo-1,3-dihydroisobenzofuran-5-yl)-4-methylhex-4-enoate (4al)

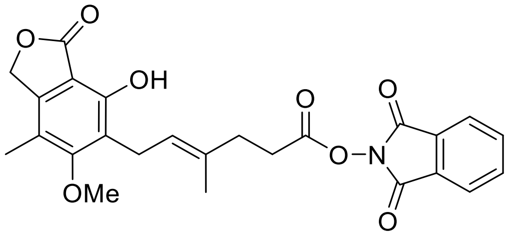

^{1}H NMR (400 MHz, $CDCl_3$): δ_H 7.91 – 7.84 (m, 2H), 7.82 – 7.74 (m, 2H), 7.69 (s, 1H), 5.34 (tq, J = 7.0, 1.3 Hz, 1H), 5.19 (s, 2H), 3.78 (s, 3H), 3.46 – 3.39 (m, 2H), 2.79 – 2.71 (m, 2H), 2.45 (t, J = 7.8 Hz, 2H), 2.15 (s, 3H), 1.85 (d, J = 1.3 Hz, 3H) ppm.

^{13}C NMR (101 MHz, $CDCl_3$): δ_C 173.0, 169.3, 163.8, 162.0, 153.8, 144.2, 134.8, 133.1, 129.1, 124.1, 123.9, 122.1, 116.8, 106.5, 70.1, 61.2, 34.1, 29.9, 22.8, 16.2, 11.7 ppm.

1,3-Dioxoisoindolin-2-yl stearate (4am)

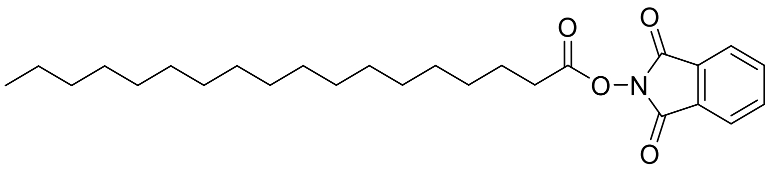

^{1}H NMR (400 MHz, $CDCl_3$): δ_H 7.91 – 7.86 (m, 2H), 7.81 – 7.77 (m, 2H), 2.66 (t, J = 7.5 Hz, 2H), 1.78 (p, J = 7.5 Hz, 2H), 1.53 – 1.38 (m, 2H), 1.37 – 1.21 (m, 26H), 0.92 – 0.84 (m, 3H) ppm.

^{13}C NMR (101 MHz, $CDCl_3$): δ_C 169.8, 162.1, 134.8, 129.1, 124.1, 32.1, 31.1, 29.8, 29.8, 29.8, 29.7, 29.5, 29.5, 29.3, 29.0, 24.8, 22.8, 14.2 ppm.

1,3-Dioxoisoindolin-2-yl oleate (4an)

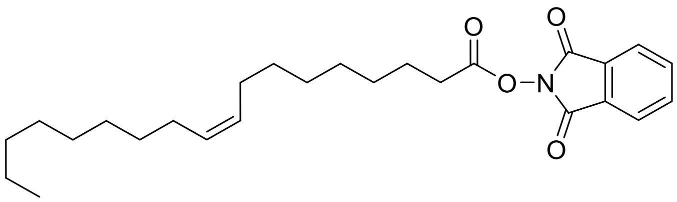

^{1}H NMR (400 MHz, $CDCl_3$): δ_H 7.91 – 7.86 (m, 2H), 7.78 (dd, *J* = 5.5, 3.1 Hz, 2H), 5.45 – 5.27 (m, 2H), 2.66 (t, *J* = 7.5 Hz, 2H), 2.10 – 1.93 (m, 4H), 1.79 (p, *J* = 7.5 Hz, 2H), 1.49 – 1.19 (m, 20H), 0.88 (t, *J* = 6.8 Hz, 3H) ppm.

^{13}C NMR (101 MHz, $CDCl_3$): δ_C 169.7, 162.1, 134.8, 130.2, 129.8, 129.1, 124.1, 32.0, 31.1, 29.9, 29.8, 29.7, 29.5, 29.4, 29.2, 28.9, 27.4, 27.3, 24.8, 22.8, 14.2 ppm.

Bis(1,3-dioxoisoindolin-2-yl) nonanedioate (4ao)

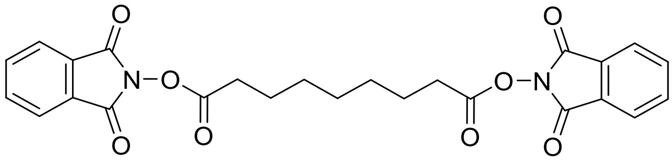

^{1}H NMR (400 MHz, $CDCl_3$): 7.89 (dd, *J* = 5.5, 3.1 Hz, 4H), 7.78 (dd, *J* = 5.5, 3.1 Hz, 4H), 2.68 (t, *J* = 7.4 Hz, 4H), 1.81 (p, *J* = 7.4 Hz, 4H), 1.46 (dddd, *J* = 21.7, 10.5, 8.6, 5.8 Hz, 6H) ppm.

^{13}C NMR (101 MHz, $CDCl_3$): δ_C 169.7, 162.1, 134.8, 129.1, 124.1, 31.1, 28.6, 24.7 ppm.

1,3-Dioxoisoindolin-2-yl (5Z,8Z,11Z,14Z)-icosa-5,8,11,14-tetraenoate (4ap)

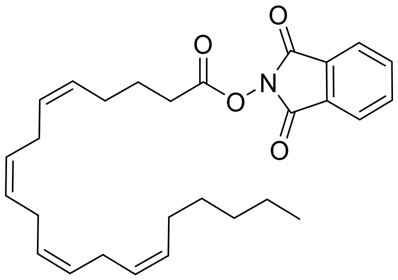

^{1}H NMR (400 MHz, $CDCl_3$): δ_H 7.92 – 7.85 (m, 2H), 7.81 – 7.76 (m, 2H), 5.54 – 5.26 (m, 8H), 2.95 – 2.75 (m, 6H), 2.68 (t, *J* = 7.4 Hz, 2H), 2.31 – 2.19 (m, 2H), 2.12 – 1.97 (m, 2H), 1.87 (p, *J* = 7.4 Hz, 2H), 1.40 – 1.20 (m, 6H), 0.93 – 0.81 (m, 3H) ppm.

^{13}C NMR (101 MHz, $CDCl_3$): δ_C 169.6, 162.1, 134.8, 130.6, 129.8, 129.1, 128.7, 128.5, 128.3, 128.2, 128.0, 127.7, 124.1, 31.6, 30.5, 29.4, 27.3, 26.3, 25.8, 24.7, 22.7, 14.2 ppm.

1,3-Dioxoisoindolin-2-yl (4*R*)-4-((5*S*,8*R*,10*S*,13*R*,14*S*,17*R*)-10,13-dimethyl-3,7,12-trioxohexadecahydro-1H-cyclopenta[a]phenanthren-17-yl)pentanoate (4aq)

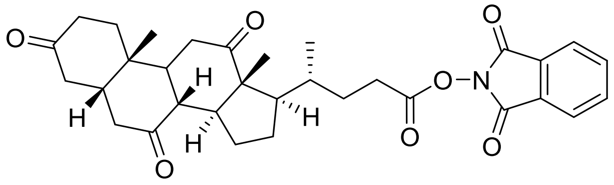

^{1}H NMR (400 MHz, $CDCl_3$): δ_H 7.90 – 7.85 (m, 2H), 7.82 – 7.75 (m, 2H), 2.97 – 2.81 (m, 3H), 2.76 (ddd, *J* = 16.0, 8.5, 5.1 Hz, 1H), 2.64 (dt, *J* = 16.1, 8.1 Hz, 1H), 2.42 – 2.28 (m, 2H), 2.25 (q, *J* = 4.7 Hz, 1H), 2.23 – 2.11 (m, 3H), 2.11 – 1.92 (m, 4H), 1.87 (td, *J* = 11.5, 7.1 Hz, 1H), 1.69 – 1.49 (m, 2H), 1.47 – 1.19 (m, 6H), 1.12 (s, 3H), 0.92 (d, *J* = 6.6 Hz, 3H) ppm.

^{13}C NMR (101 MHz, $CDCl_3$): δ_C 212.0, 209.1, 208.8, 170.0, 162.1, 134.9, 129.1, 124.1, 57.1, 51.9, 49.1, 47.0, 45.8, 45.7, 45.1, 42.9, 38.8, 36.6, 36.1, 35.4, 30.5, 28.6, 27.8, 25.3, 22.0, 18.7, 12.0 ppm.

1,3-Dioxoisoindolin-2-yl 3-phenylpropanoate (**4ar**)

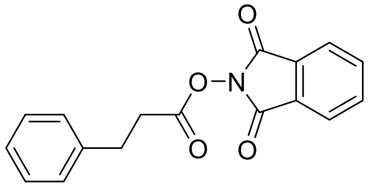

^{1}H NMR (400 MHz, $CDCl_3$): δ_H 7.87 (dd, *J* = 5.5, 3.1 Hz, 2H), 7.77 (dd, *J* = 5.5, 3.1 Hz, 2H), 7.35 – 7.29 (m, 2H), 7.27 – 7.21 (m, 3H), 3.12 – 3.06 (m, 2H), 2.98 (ddd, *J* = 8.3, 7.0, 1.1 Hz, 2H) ppm.

^{13}C NMR (101 MHz, $CDCl_3$): δ_C 169.0, 162.0, 139.3, 134.9, 129.0, 128.8, 128.4, 126.8, 124.1, 32.8, 30.6 ppm.

1,3-Dioxoisoindolin-2-yl 4-phenylbutanoate (4as)

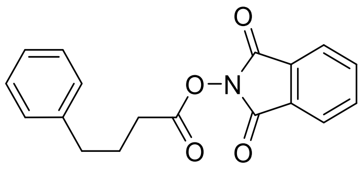

^{1}H NMR (400 MHz, $CDCl_3$): δ_H δ 7.91 (dt, *J* = 7.4, 3.7 Hz, 2H), 7.81 (dd, *J* = 5.5, 3.1 Hz, 2H), 7.34 (t, *J* = 7.5 Hz, 2H), 7.26 (t, *J* = 7.7 Hz, 3H), 2.80 (t, *J* = 7.5 Hz, 2H), 2.69 (t, *J* = 7.4 Hz, 2H), 2.14 (p, *J* = 7.5 Hz, 2H) ppm.

^{13}C NMR (101 MHz, $CDCl_3$): δ_C 169.5, 162.1, 140.8, 134.9, 129.0, 128.7, 128.6, 126.3, 124.1, 34.7, 30.3, 26.4 ppm.

1,3-Dioxoisoindolin-2-yl pent-4-enoate (4at)

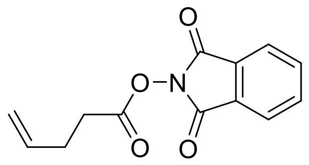

^{1}H NMR (400 MHz, $CDCl_3$): δ_H 7.89 – 7.83 (m, 2H), 7.80 – 7.74 (m, 2H), 5.87 (ddt, *J* = 16.8, 10.2, 6.4 Hz, 1H), 5.19 – 5.04 (m, 2H), 2.81 – 2.68 (m, 2H), 2.51 (dtt, *J* = 8.5, 7.1, 1.4 Hz, 2H) ppm.

^{13}C NMR (101 MHz, $CDCl_3$): δ_C 169.1, 162.0, 135.3, 134.8, 129.0, 124.0, 116.7, 30.4, 28.5 ppm.

1,3-Dioxoisoindolin-2-yl 3-((*tert*-butoxycarbonyl)amino)propanoate (4au)

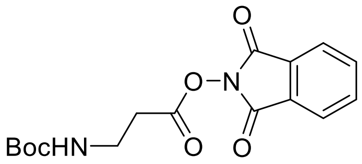

^{1}H NMR (400 MHz, $CDCl_3$): 7.89 (dd, *J* = 5.5, 3.1 Hz, 2H), 7.84 – 7.77 (m, 2H), 5.14 (bs, 1H), 3.56 (q, *J* = 6.2 Hz, 2H), 2.90 (t, *J* = 6.0 Hz, 2H), 1.45 (s, 9H) ppm.

^{13}C NMR (101 MHz, $CDCl_3$): δ_C 168.7, 162.0, 155.9, 135.0, 129.0, 124.2, 80.0, 36.3, 32.2, 28.5 ppm.

1,3-Dioxoisoindolin-2-yl methyl succinate (4av)

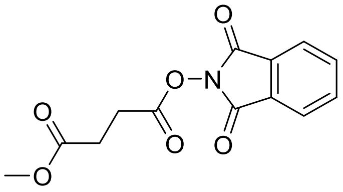

^{1}H NMR (400 MHz, $CDCl_3$): δ_H 7.89 (dd, *J* = 5.5, 3.1 Hz, 2H), 7.83 – 7.77 (m, 2H), 3.75 (s, 3H), 3.02 (td, *J* = 7.1, 0.5 Hz, 2H), 2.79 (t, *J* = 7.0 Hz, 2H) ppm.

^{13}C NMR (101 MHz, $CDCl_3$): δ_C 171.6, 168.8, 161.9, 134.9, 129.0, 124.1, 52.3, 28.8, 26.5 ppm.

1,3-Dioxoisoindolin-2-yl isobutyrate (4az)

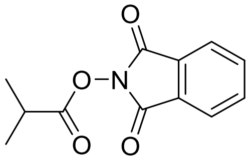

^{1}H NMR (400 MHz, $CDCl_3$): δ_H δ 7.91 – 7.85 (m, 2H), 7.81 – 7.75 (m, 2H), 2.96 (hept, *J* = 7.0 Hz, 1H), 1.38 (d, *J* = 7.0 Hz, 6H) ppm.

^{13}C NMR (101 MHz, $CDCl_3$): δ_C 173.2, 162.2, 134.8, 129.1, 124.0, 31.9, 19.0 ppm.

1,3-Dioxoisoindolin-2-yl cyclobutanecarboxylate (**4ba**)

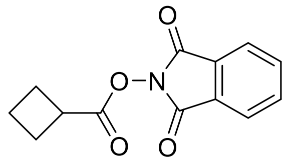

^{1}H NMR (400 MHz, $CDCl_3$): δ_H 7.92 – 7.85 (m, 2H), 7.82 – 7.75 (m, 2H), 3.56 – 3.46 (m, 1H), 2.57 – 2.46 (m, 2H), 2.46 – 2.36 (m, 2H), 2.18 – 1.98 (m, 2H) ppm.

^{13}C NMR (101 MHz, $CDCl_3$): δ_C 171.5, 162.2, 134.8, 129.2, 124.1, 35.2, 25.5, 18.9 ppm.

1,3-Dioxoisoindolin-2-yl tetrahydro-2H-pyran-4-carboxylate (4bb)

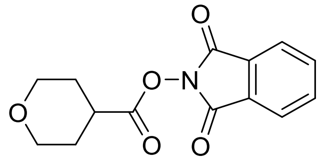

^{1}H NMR (400 MHz, $CDCl_3$): δ_H 7.90 – 7.85 (m, 2H), 7.81 – 7.77 (m, 2H), 4.02 (dt, *J* = 11.7, 3.8 Hz, 2H), 3.53 (ddd, *J* = 11.8, 9.9, 3.3 Hz, 2H), 3.00 (tt, *J* = 9.7, 4.7 Hz, 1H), 2.09 – 1.92 (m, 4H) ppm.

^{13}C NMR (101 MHz, $CDCl_3$): δ_C 170.7, 162.0, 134.9, 129.1, 124.1, 66.7, 37.7, 28.4 ppm.

1-(*tert*-Butyl) 4-(1,3-dioxoisoindolin-2-yl) piperidine-1,4-dicarboxylate (4bc)

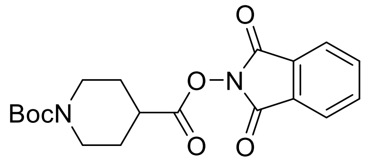

^{1}H NMR (400 MHz, $CDCl_3$): δ_H 7.88 (ddd, *J* = 5.5, 3.1, 1.0 Hz, 2H), 7.79 (ddd, *J* = 5.8, 3.0, 0.9 Hz, 2H), 4.03 (dt, *J* = 14.0, 4.4 Hz, 2H), 3.01 (ddd, *J* = 13.7, 10.7, 3.1 Hz, 2H), 2.91 (tt, *J* = 10.3, 4.0 Hz, 1H), 2.17 – 2.01 (m, 2H), 1.85 (dtd, *J* = 14.3, 10.5, 4.1 Hz, 2H), 1.46 (s, 9H) ppm.

^{13}C NMR (101 MHz, $CDCl_3$): δ_C 170.8, 162.0, 154.7, 134.9, 129.1, 124.1, 80.0, 42.7, 38.7, 28.5, 27.9 ppm.

1,3-Dioxoisoindolin-2-yl hept-6-enoate (4bd)

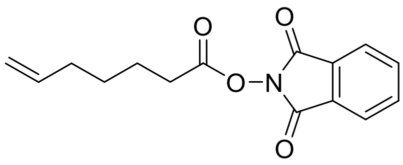

^{1}H NMR (400 MHz, $CDCl_3$): δ_H 7.91 – 7.84 (m, 2H), 7.81 – 7.74 (m, 2H), 5.81 (ddt, *J* = 16.9, 10.2, 6.6 Hz, 1H), 5.08 – 4.94 (m, 2H), 2.67 (t, *J* = 7.4 Hz, 2H), 2.19 – 2.05 (m, 2H), 1.86 – 1.75 (m, 2H), 1.62 – 1.49 (m, 2H) ppm.

^{13}C NMR (101 MHz, $CDCl_3$): δ_C 169.6, 162.1, 138.1, 134.8, 129.1, 124.1, 115.2, 33.3, 30.9, 28.1, 24.2 ppm.

2.4. General Procedure D: Synthesis of phosphites

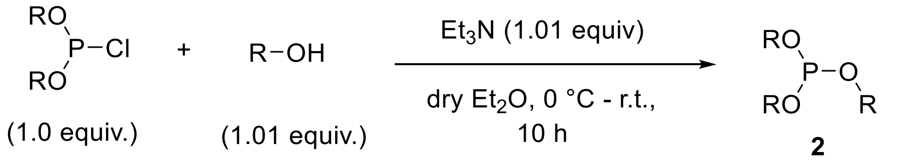

Using a modified literature procedure.[12] Under a N_2 atmosphere, triethylamine (1.0 equiv.) was added to a stirred and cooled (0°C) solution of chlorophosphite (1.0 equiv.) in 0.15 M of dry diethyl ether under N_2 and the mixture was stirred over additional 5 min. Then the solution of 1.01 equiv. of the corresponding alcohol in 10 mL of diethyl ether was added. The mixture was allowed to warm up to ambient temperature. After 10 h the Et_3N hydrochloride was filtered off through Celite pad and rinsed well with ether. Solvent was removed from the combined filtrate under reduced pressure to give of the phosphites (some phosphites were directly used without further purification). Some of them needed further distillation to get pure phosphites.

The following phosphites **2b-2d** and **2h** are purchased from the commercial sources.

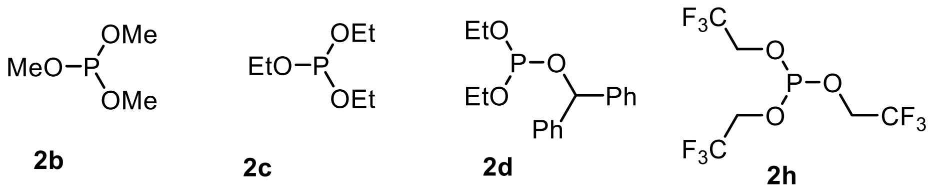

2b **2c** **2d** **2h**

2-(Benzhydryloxy)benzo[*d*][1,3,2]dioxaphosphole (**2a**)

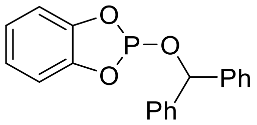

Prepared following the above **General Procedure D** with diphenylmethanol (1.84 g, 10.0 mmol).

The title compound (2.28 g, 7.10 mmol, 71%) was used without further purification as a colorless solid.

^{1}H NMR (400 MHz, $CDCl_3$): δ_H 7.27 – 7.18 (m, 6H), 7.13 – 7.06 (m, 4H), 6.97 – 6.89 (m, 4H), 5.80 (d, J = 8.7 Hz, 1H) ppm.

^{13}C NMR (101 MHz, $CDCl_3$): δ_C 145.7 (d, J = 7.7 Hz), 141.0 (d, J = 2.0 Hz), 128.4, 127.9, 126.7, 122.8, 112.2, 78.7 (d, J = 1.2 Hz) ppm.

^{31}P NMR (162 MHz, $CDCl_3$): δ_P 126.9 (s) ppm.

IR (film) ν_{max}: 3053, 1475, 1332, 1254, 1230, 976, 824, 731 cm^{-1}.

M.p.: 52-54 ˚C.

HRMS (EI$^+$): calcd. for $C_{19}H_{15}O_3P$ $[M]^+$ 322.0753, found 322.0754.

2-(Benzhydryloxy)-1,3,2-dioxaphospholane (2e)

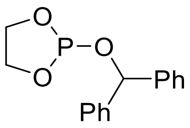

Prepared following the above **General Procedure D** with diphenyl methanol (920 mg, 5.0 mmol) gave the title compound (1164 mg, 4.2 mmol, 85%) as a colorless solid. **Note**: This compound used directly without further purification because it is unstable on silica gel.

^{1}H NMR (400 MHz, $CDCl_3$): δ_H 7.33 – 7.15 (m, 10H), 6.06 (d, *J* = 8.9 Hz, 1H), 4.08 – 3.97 (m, 2H), 3.90 – 3.78 (m, 2H) ppm.

^{13}C NMR (101 MHz, $CDCl_3$): δ_C δ 142.2 (d, *J* = 3.2 Hz), 128.4, 127.7, 126.8, 76.6 (d, *J* = 15.3 Hz), 64.0 (d, *J* = 8.6 Hz) ppm.

^{31}P NMR (162 MHz, $CDCl_3$): δ_P 134.9 (s) ppm.

IR (film) ν_{max}: 2973, 1493, 1453, 989, 916, 814, 790, 732, 694, 623, 600, 532, 451 cm^{-1}.

HRMS (EI^+): calcd. for $C_{15}H_{15}O_3P$ $[M]^+$ 274.0284, found 274.0283.

2-Ethoxybenzo[*d*][1,3,2]dioxaphosphole (**2f**)

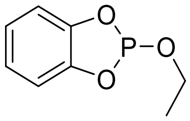

Prepared following the above **General Procedure D** with ethanol (505.6 mg, 5.0 mmol). Purification by distillation gave the title compound (736.0 mg, 4.0 mmol, 80%) as a colorless oil.

^{1}H NMR (400 MHz, $CDCl_3$): δ_H 7.11 – 7.06 (m, 2H), 7.00 – 6.95 (m, 2H), 3.64 (p, *J* = 7.0 Hz, 2H), 1.17 (t, *J* = 7.1 Hz, 3H) ppm.

^{13}C NMR (101 MHz, $CDCl_3$): δ_C 145.9 (d, *J* = 7.5 Hz), 122.7, 111.9, 60.2, 16.6 (d, *J* = 3.3 Hz) ppm.

^{31}P NMR (162 MHz, $CDCl_3$): δ_P 127.6 (s) ppm.

IR (film) ν_{max}: 1476, 1372, 1231, 1025, 905, 725 cm^{-1}.

HRMS (EI^+) calcd. for $C_8H_9O_3P$ $[M]^+$ 184.0284, found 184.0283.

2-(Benzyloxy)benzo[d][1,3,2]dioxaphosphole (2g)

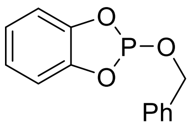

Prepared following the above **General Procedure D** with benzyl alcohol (540 mg, 5.0 mmol) gave the title compound (615 mg, 2.5 mmol, 50%) as a colorless oil. **Note**: This compound quickly passed through the silica gel column.

^{1}H NMR (400 MHz, $CDCl_3$): δ_H 7.41 – 7.27 (m, 3H), 7.21 (dd, *J* = 7.6, 2.0 Hz, 2H), 7.16 – 7.08 (m, 2H), 7.02 (dd, *J* = 5.9, 3.4 Hz, 2H), 4.61 (d, *J* = 6.9 Hz, 2H) ppm.

^{13}C NMR (101 MHz, $CDCl_3$): δ_C 145.9 (d, *J* = 7.7 Hz), 136.5 (d, *J* = 3.1 Hz), 128.6, 128.2, 127.6, 122.9 (d, *J* = 1.0 Hz), 112.1 (d, *J* = 1.4 Hz), 65.8 (d, *J* = 1.5 Hz) ppm.

^{31}P NMR (162 MHz, $CDCl_3$): δ_P 127.5 (s) ppm.

IR (film) ν_{max}: 2949, 1475, 1228, 1032, 1007, 979, 824, 729, 693, 625, 525, 459 cm^{-1}.

HRMS (EI^+): calcd. for $C_{13}H_{11}O_3P$ $[M]^+$ 246.0440, found 246.0437.

2-(Benzhydryloxy)-5,5-dimethyl-1,3,2-dioxaphosphinane (2i)

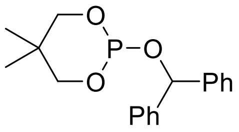

Prepared following the above **General Procedure D** with diphenyl methanol (920 mg, 5.0 mmol) gave the title compound (1295 mg, 4.1 mmol, 82%) as a colorless solid. **Note**: This compound was directly used without further purification because it was unstable to purify.

^{1}H NMR (400 MHz, $CDCl_3$): δ_H 7.39 (dt, *J* = 8.0, 1.8 Hz, 4H), 7.37 – 7.32 (m, 4H), 7.30 – 7.27 (m, 2H), 6.09 (d, *J* = 8.5 Hz, 1H), 4.09 – 3.99 (m, 2H), 3.27 (tt, *J* = 10.7, 1.5 Hz, 2H), 1.24 (s, 3H), 0.67 (s, *J* = 1.1 Hz, 3H) ppm.

^{13}C NMR (101 MHz, $CDCl_3$): δ_C 142.6 (d, *J* = 3.1 Hz), 128.5, 127.7, 127.0, 76.7 (d, *J* = 19.2 Hz), 22.9 (d, *J* = 1.2 Hz), 22.6 ppm.

^{31}P NMR (162 MHz, $CDCl_3$): δ_P 122.1 (s) ppm.

IR (film) ν_{max}: 2958, 1494, 1472, 1454, 1265, 1291, 1057, 1032, 993, 809, 787, 762, 734, 695, 623, 602, 549 cm^{-1}.

HRMS (EI^+): calcd. for $C_{18}H_{21}O_3P$ $[M]^+$ 316.1223, found 316.1220.

2-Isopropoxybenzo[*d*][1,3,2]dioxaphosphole (2j)

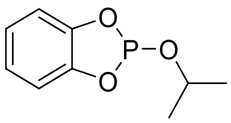

Prepared following the above **General Procedure D** with isopropyl alcohol (300.0 mg, 5.0 mmol). Purification by distillation gave the title compound (872.0 mg, 4.4 mmol, 88%) as a colorless oil.

^{1}H NMR (400 MHz, $CDCl_3$): δ_H 7.11 – 7.05 (m, 2H), 7.00 – 6.95 (m, 2H), 4.23 – 4.10 (m, 1H), 1.17 (d, *J* = 6.2 Hz, 6H) ppm.

^{13}C NMR (101 MHz, $CDCl_3$): δ_C 145.9 (d, *J* = 7.7 Hz), 122.6, 112.1, 69.6, 24.6 (d, *J* = 2.4 Hz) ppm.

^{31}P NMR (162 MHz, $CDCl_3$): δ_P 129.7 (s) ppm.

IR (film) ν_{max}: 2981, 1476, 1374, 1232, 1009, 978, 905, 825, 725 cm^{-1}.

HRMS (EI^+): calcd. for $C_9H_{11}O_3P$ $[M]^+$ 198.0440, found 198.0438.

2-((1,3-Difluoropropan-2-yl)oxy)benzo[*d*][1,3,2]dioxaphosphole (2k)

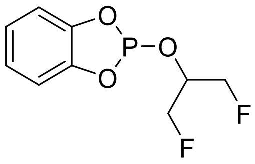

Prepared following the above **General Procedure D** with isopropyl alcohol (480.5 mg, 5.0 mmol). Purification by distillation gave the title compound (721.0 mg, 3.50 mmol, 70%) as a colorless oil.

^{1}H NMR (400 MHz, $CDCl_3$): δ_H 7.16 – 7.10 (m, 2H), 7.05 – 6.99 (m, 2H), 4.52 – 4.43 (m, 2H), 4.40 – 4.33 (m, 2H), 4.32 – 4.18 (m, 1H) ppm.

^{13}C NMR (101 MHz, $CDCl_3$): δ_C 145.2 (d, *J* = 7.5 Hz), 123.2, 112.5, 82.2 (dd, *J* = 6.7, 2.1 Hz), 80.4 (dd, *J* = 6.7, 2.1 Hz), 72.0 (td, *J* = 21.1, 2.7 Hz) ppm.

^{19}F NMR (377 MHz, $CDCl_3$): δ_F -230.9 – -231.3 (m) ppm.

^{31}P NMR (162 MHz, $CDCl_3$): δ_P 131.1 (s) ppm.

IR (film) ν_{max}: 2969, 1475, 1332, 1230, 1095, 1030, 906, 828, 727 cm^{-1}.

HRMS (EI^+): calcd. for $C_9H_9F_2O_3P$ $[M]^+$ 234.0252, found 234.0252.

2-((1,1,1,3,3,3-Hexafluoropropan-2-yl)oxy)benzo[d][1,3,2]dioxaphosphole (**2l**)

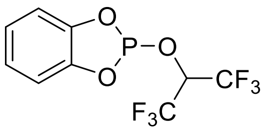

Prepared following the above **General Procedure D** with 1,1,1,3,3,3-hexafluoropropan-2-ol (1.68g, 10.0 mmol). Purification by distillation gave the title compound (2.63 g, 8.60 mmol, 86%) as a colorless oil.

^{1}H NMR (400 MHz, $CDCl_3$): δ_H 7.22 – 7.16 (m, 2H), 7.11 – 7.06 (m, 2H), 4.54 – 4.43 (m, 1H) ppm.

^{13}C NMR (101 MHz, $CDCl_3$): δ_C 144.4 (d, *J* = 7.5 Hz), 123.9, 120.4 (d, *J* = 283.3 Hz), 113.0 (d, *J* = 1.7 Hz), 70.5 – 69.02 (m) ppm.

^{19}F NMR (377 MHz, $CDCl_3$): δ_F -74.43 (t, *J* = 5.2 Hz) ppm.

^{31}P NMR (162 MHz, $CDCl_3$): δ_P 133.42 – 133.16 (m) ppm.

IR (film) ν_{max}: 1475, 1372, 1294, 1230, 903, 724 cm^{-1}.

HRMS (EI^+): calcd. for $C_9H_5F_6O_3P$ $[M]^+$ 305.9875, found 305.9873.

2-((9*H*-Fluoren-9-yl)oxy)benzo[d][1,3,2]dioxaphosphole (**2m**)

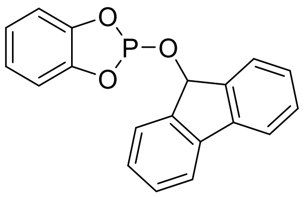

Prepared following the above **General Procedure D** with 9*H*-fluoren-9-ol (911.0 mg, 5.0 mmol). The title compound (1.44 g, 4.5 mmol, 90%) was used without further purification as a colorless solid.

^{1}H NMR (400 MHz, $CDCl_3$): δ_H 7.66 – 7.56 (m, 4H), 7.40 – 7.30 (m, 4H), 7.16 – 7.11 (m, 2H), 6.99 – 6.93 (m, 2H), 5.68 (d, *J* = 10.4 Hz, 1H) ppm.

^{13}C NMR (101 MHz, $CDCl_3$): δ_C 145.7 (d, *J* = 7.8 Hz), 142.2 (d, *J* = 2.1 Hz), 140.4, 129.6, 128.0, 125.8, 123.0, 120.1, 112.3, 76.8 (d, *J* = 1.0 Hz) ppm.

^{31}P NMR (162 MHz, $CDCl_3$): δ_P 130.6 (s) ppm.

IR (film) ν_{max}: 1476, 1330, 1231, 993, 903, 825, 728 cm^{-1}.

M.p:132-134 ˚C.

HRMS (EI^+): calcd. for $C_{19}H_{13}O_3P$ $[M]^+$ 320.0597, found 320.0595.

3. PHOTOCATALYSIS

3.1. Photochemical Equipment and Reaction Setup

The blue LED lamps were either 40 W Kessil A160WE Tuna Blue LED Aquarium Lights (used with the color dial turned fully anticlockwise and the intensity dial turned fully clockwise) or 40 W Kessil PR160-427 nm LED Photoredox Lights (used with the intensity dial set to 100). All photoredox reactions were carried out at room temperature (r.t.) ~30 °C. Fan assisted cooling was used to maintain this temperature. The reaction flasks were positioned ~5 cm from a single 40 W Kessil LED lamp (Figure S1).

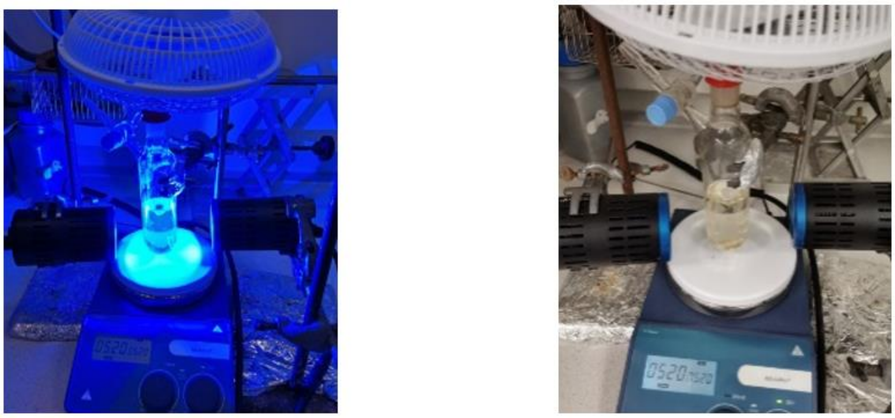

Figure S1. Photocatalysis reaction setup.

3.2. Optimization of the Deboronative Phosphonylation reaction

3.2.1. Table S1: Optimization of phosphite sources

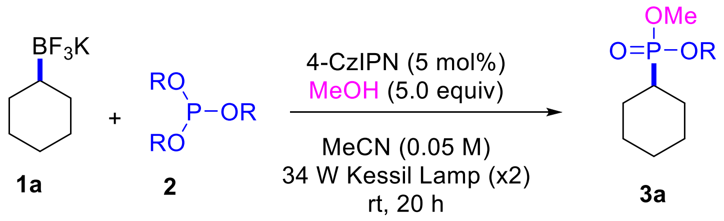

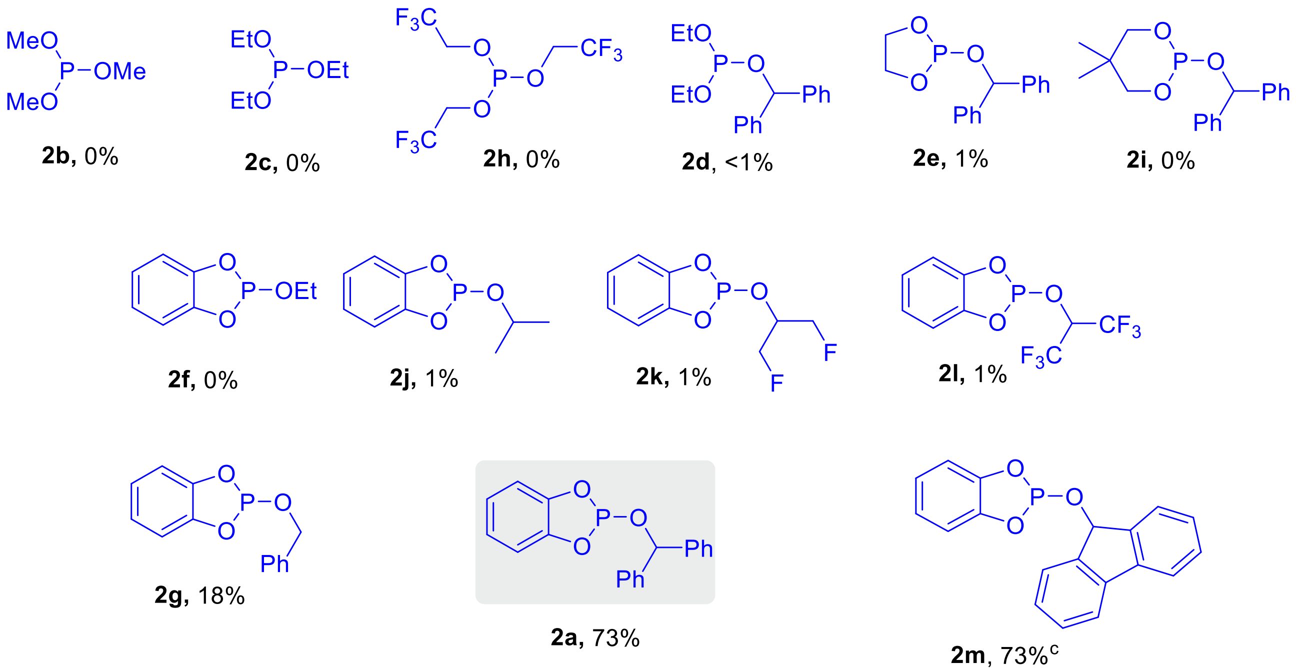

^{a}Reaction conditions: **1a** (0.10 mmol), **2** (0.15 mmol), MeCN (0.05 M, 2.0 mL), 5 mol% 4-CzIPN, MeOH (0.5 mmol), 40 W Kessil Lamp, N_2, r.t., 20 h.
^{b}Measured by 1H-NMR of crude reaction mixture using diethyl phthalate as an internal standard.
^{c}2.0 equiv. of **2m**.

3.2.2. Table S2: Optimization of additives

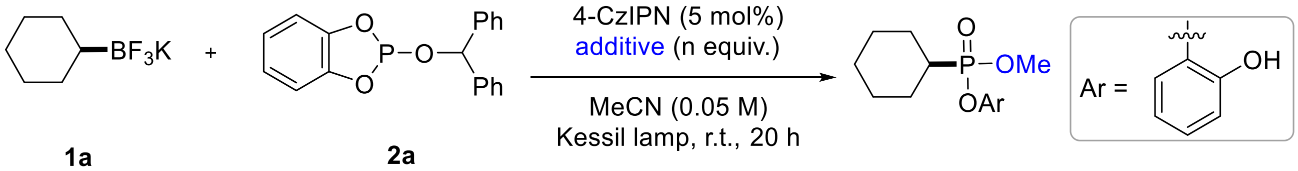

1a (1.0 equiv.) **2a** (1.5 equiv.) **3a**

**Entry**	Additive (n equiv.)	NMR yield (**3a**)[a,b]
1	none	2%
2	MeOH (2.0)	57%
3	MeOH (5.0)	73%
4	NaH_2PO_4 (2.0)	26%
5	KH_2PO_4 (2.0)	29%
6	H_2O (2.0)	1%
7	PPTS (2.0)	19%
8	catechol (2.0)	33%
9	Et_3N•HCl (2.0)	29%

[a]Reaction conditions: **1a** (0.10 mmol), **2a** (0.15 mmol), MeCN (0.05 M, 2.0 mL), 5 mol% 4-CzIPN, MeOH (0.5 mmol), 40 W Kessil Lamp, N_2, r.t., 20 h.
[b]Measured by ^{1}H-NMR of crude reaction mixture using diethyl phthalate as an internal standard.

3.2.3. Table S3: Optimization of MeOH loading

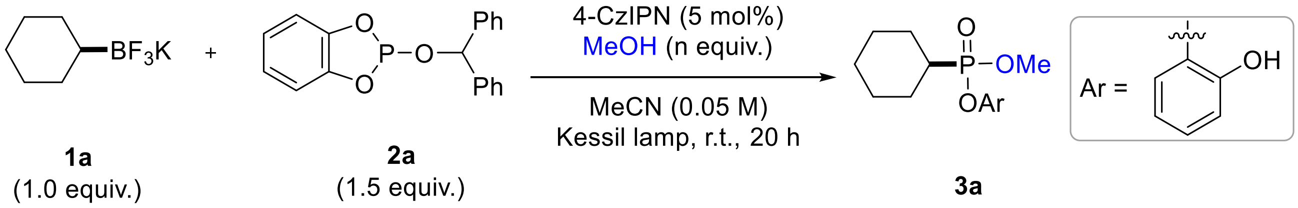

Entry	MeOH equivalents	NMR yield (**3a**)[a,b]
1	0	2%
2	2	57%
3	3	65%
4	5	73%
5[c]	6	60%
6[c]	10	65%
7[c]	40	60%

[a]Reaction conditions: **1a** (0.10 mmol), **2a** (0.15 mmol), MeCN (0.05 M, 2.0 mL), 5 mol% 4-CzIPN, MeOH (n mmol), 40 W Kessil Lamp, N_2, r.t., 20 h.
[b]Measured by ^{1}H-NMR of crude reaction mixture using diethyl phthalate as an internal standard.
[c]Excess of MeOH (or pure MeOH as a solvent) decomposes the BecaP reagent **2a** to form **12** (***vide infra*).**

3.2.4. Table S4: Optimization of photocatalysts

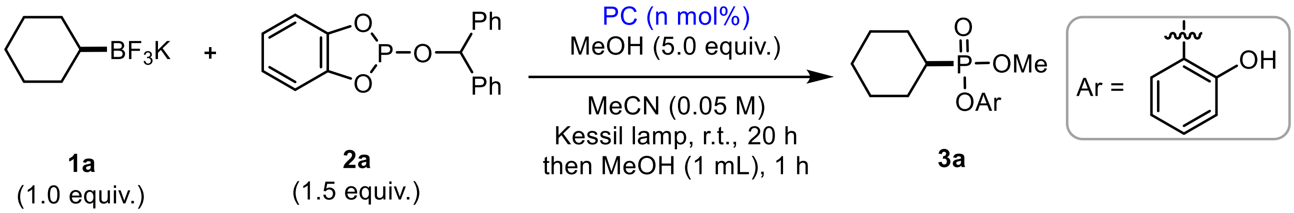

Entry	[PC]	NMR Yield (**3a**)[a,b]
1	5 mol% 4CzIPN	73%
2	1 mol% 4CzIPN	66%
3[c]	5 mol% 4CzIPN	20%
4	2% Ir[dF(CF$_3$)ppy]$_2$(dtbpy)PF$_6$	64%
5	2% Ir[(dtbbpy)(ppy)$_2$]PF$_6$	75%
6	5 mol% Ir(ppy)$_3$	17%
7	w/o photocatalyst	0%
8[d]	5 mol% 4CzIPN	0%

[a]Reaction conditions: **1a** (0.10 mmol), **2a** (0.15 mmol), MeCN (0.05 M, 2.0 mL), 5 mol% 4-CzIPN, MeOH (0.5 mmol), 40 W Kessil Lamp, N_2, r.t., 20 h.
[b]Measured by ^{1}H-NMR of crude reaction mixture using diethyl phthalate as an internal standard.
[c]Open to air.
[d]In the dark.

3.2.5. Table S5: Optimization of Solvents

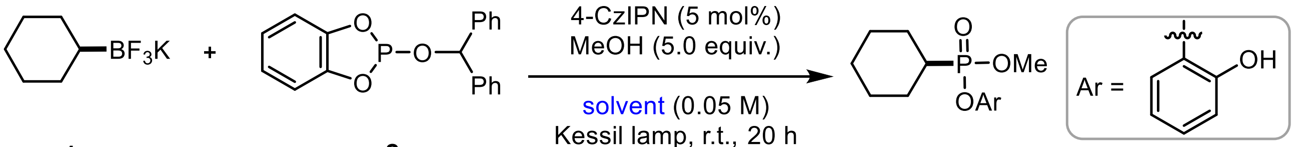

1a (1.0 equiv.) **2a** (1.5 equiv.) **3a**

Entry	Solvent	NMR yield (**3a**)[a,b]
1	MeCN	73%
2	DMF	31%
3	toluene	12%
4	EtOAc	14%
5	acetone	58%
6	THF	56%
7	1,4-dioxane	77%
8[c]	1,4-dioxane	85%

[a]Reaction conditions: **1a** (0.10 mmol), **2a** (0.15 mmol), solvent (0.05 M, 2.0 mL), 5 mol% 4-CzIPN, MeOH (0.5 mmol), 40 W Kessil Lamp, N_2, r.t., 20 h.
[b]Measured by ^{1}H-NMR of crude reaction mixture using diethyl phthalate as an internal standard.
[c]With 2.0 equiv. of **2a**.

3.3. Optimization of the Decarboxylative Phosphonylation

3.3.1. Table S6: Optimization of phosphite equivalents

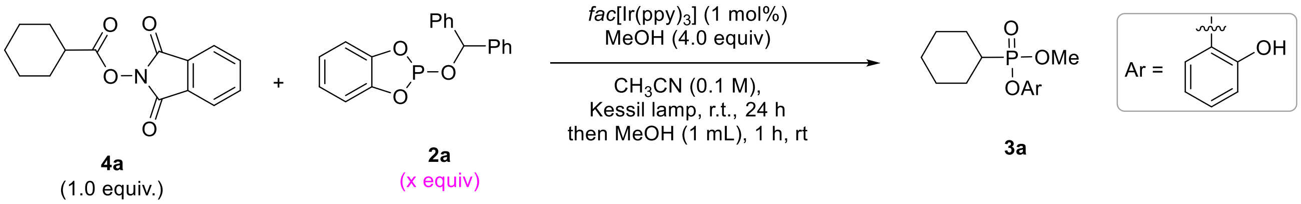

1	1.1	47
2	1.5	60
3	2.0	69
4	2.5	78
5	3.0	69

[a]Reaction conditions: **4a** (0.10 mmol), **2a** (x mmol),CH_3CN (0.1 M, 1.0 mL), 1 mol% *fac*[Ir(ppy)$_3$], MeOH (0.4 mmol), 40 W Kessil Lamp, N_2, r.t., 20 h.
[b]Measured by ^{1}H-NMR of crude reaction mixture using diethyl phthalate as an internal standard.

3.3.2. Table S7: Optimization of solvents

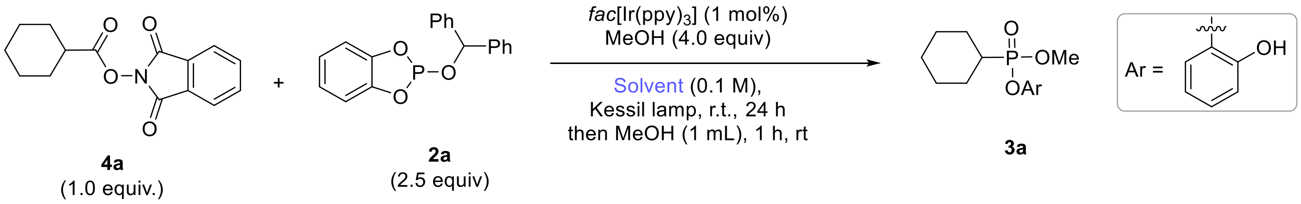

**Entry**	Solvent	Yield (**3a**)[a,b]
1	1,4-Dioxane	11
2	CH_3NO_2	13
3	CH_2Cl_2	59
4	EtOAc	31
5	THF	72
6	HFIP	0
7	CH_3CN	75

[a]Reaction conditions: **4a** (0.10 mmol), **2a** (0.25 mmol), solvent (0.1 M, 1.0 mL), 1 mol% *fac*[Ir(ppy)$_3$], MeOH (0.4 mmol), 40 W Kessil Lamp, N_2, r.t., 20 h.
[b]Measured by ^{1}H-NMR of crude reaction mixture using diethyl phthalate as an internal standard.

3.3.3. Table S8: Optimization of photocatalysts

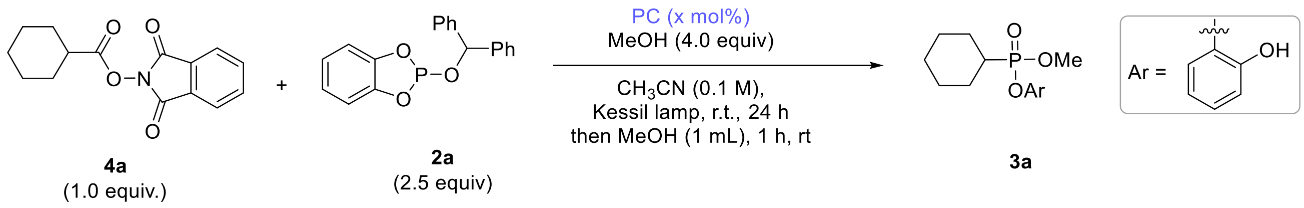

Entry	PC (x mol%)	Yield (**3a**)[a,b]
1	4-CzIPN (5)	32
2	$[Ir(dF\{CF_3\}ppy)_2(dtbbpy)]PF_6$ (1)	58
3	$[Ir(ppy)_2(dtbbpy)]PF_6$ (1)	81
4[c]	$[Ir(dF\{CF_3\}ppy)_2(dtbbpy)]PF_6$ (1)	0
5	w/o photocatalyst	0

[a]Reaction conditions: **4a** (0.10 mmol), **2a** (0.25 mmol), CH_3CN (0.1 M, 1.0 mL), 1 mol% PC, MeOH (0.4 mmol), 40 W Kessil Lamp, N_2, r.t., 20 h.
[b]Measured by ^{1}H-NMR of crude reaction mixture using diethyl phthalate as an internal standard.
[c]In the dark.

3.4. General Procedure E: Deboronative Phosphonylation

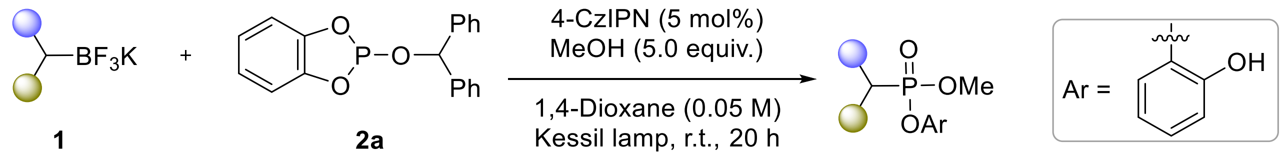

1 (1.0 equiv.) **2a** (1.5 equiv.) **3**

A degassed solution of potassium trifluoroborate **1** (0.30 mmol, 1.0 equiv.), phosphite **2a** (0.60 mmol, 2.0 equiv.), MeOH (1.5 mmol, 5 equiv.) and 4-CzIPN (5.0 mol %) in 1,4-dioxane (6.0 mL) was added under irradiation with a 40 W Kessil LED lamp with fan cooling. The N_2 inlet was removed, and the flask sealed with parafilm. The reaction mixture was stirred vigorously for 20 h under constant irradiation. Then, the lights are switched off and 1 mL MeOH was added to the reaction mixture, and the reaction was allowed to stir for an additional hour. The reaction mixture was diluted with DCM (60 mL) and the solution washed with saturated aqueous NH_4Cl (~30 mL), water (~30 mL) and brine (~30 mL). The resulting organic phase was dried ($MgSO_4$), filtered and concentrated *in vacuo*. The crude product was then purified by flash column chromatography. The crude residue was purified by flash column chromatography on silica gel to yield the corresponding phosphonate **3**.

3.5. General Procedure F: Decarboxylative Phosphonylation

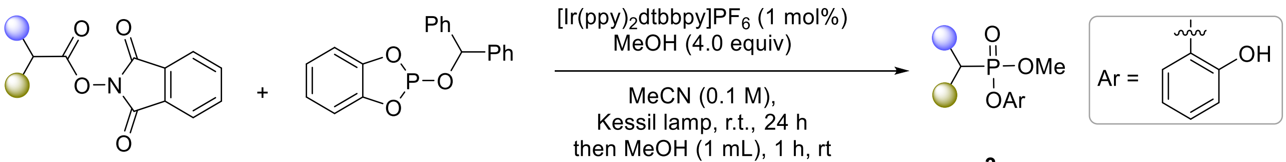

4 **2a** **3**

Condition A *(Unless otherwise stated, condition A was used)*: A degassed solution of redox active ester **4** (0.20 mmol, 1.0 equiv.), phosphite **2a** (0.5 mmol, 2.5 equiv.), MeOH (0.8 mmol, 4.0 equiv.) and $[Ir(ppy)_2dtbbpy]PF_6$ (1.0 mol%) in dry CH_3CN (2.0 mL) was added under irradiation with a 40 W Kessil LED lamp with fan cooling. The N_2 inlet was removed, and the flask was sealed with parafilm. The reaction mixture was stirred vigorously (~1000 rpm) for 24 h under constant irradiation. After 24 h, the lights are switched off and 1 mL MeOH was added to the reaction mixture, and the reaction was allowed to stir for an additional hour. After that the resulting reaction mixture was diluted with DCM (~60 mL) and the solution was washed with saturated aqueous NH_4Cl (~30 mL), water (~30 mL) and brine (~30 mL). The resulting organic phase was dried ($MgSO_4$), filtered and concentrated *in vacuo*. The crude product **3** was then purified by flash column chromatography on silica gel to yield the pure phosphonate **3**.

Note: In the most cases, the second column in different solvent system (from EtOAc/hexane to either acetone/pentane or THF/pentane) were required to remove the phthalimide side product and/or other inseparable impurities obtained from first EtOAc/hexane column.

.

Condition B: MeCN:THF (2:1, v/v) instead of MeCN: Some compounds were insoluble in the MeCN itself (see Table S9).

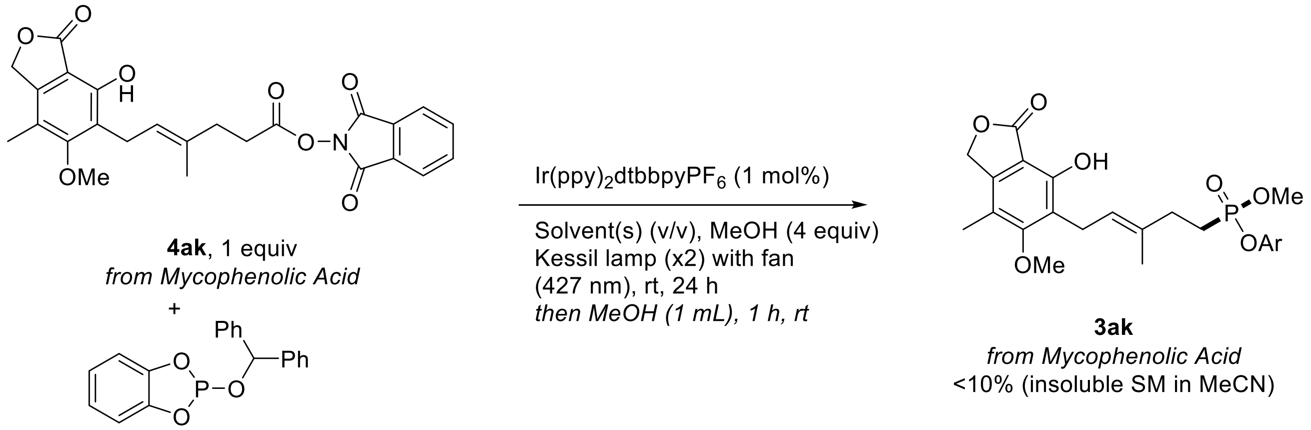

4ak, 1 equiv
from Mycophenolic Acid
+
2a
(2.5 equiv)

3ak
from Mycophenolic Acid
<10% (insoluble SM in MeCN)

Entry	Solvent(s) (v/v)	Yield[a,b]
1	MeCN	<10
2	MeCN:THF (1:1)	58
3	MeCN:THF (2:1)	**81**
4	MeCN:THF (1:2)	57
5	THF	48

Table S9: [a]Reaction conditions: **4** (0.20 mmol), **2a** (0.5 mmol), solvent(s) (3.0 mL), 1 mol% *fac*[Ir(ppy)$_3$], MeOH (0.8 mmol), 40 W Kessil Lamp, N_2, r.t., 20 h.
[b]Isolated yield.

A few more examples: Left picture the substrate is in MeCN; right picture: the substrate is in MeCN:THF

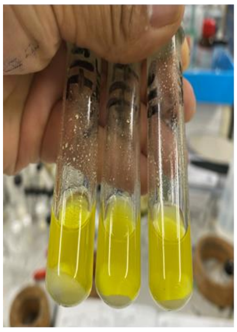

	Yield (%) in MeCN	Yield (%) in MeCN:THF (2:1)
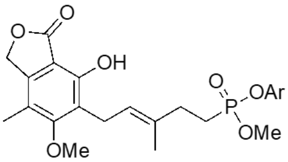 *from mycophenolic acid*	<10%	81%
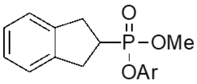 *from benzocyclopentyl*	<10%	86%
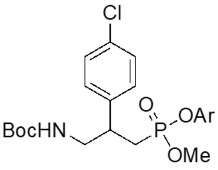 *from baclofen*	<15%	65%

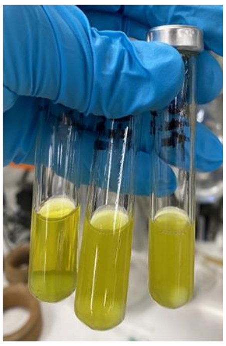

4. PRODUCT CHARACTERIZATION

2-hydroxyphenyl methyl butylphosphonate (**3b**)

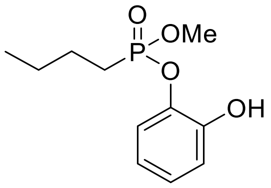

Prepared following **General Procedure E**. Purification by flash column chromatography (50% EtOAc/hexane) gave the title compound (54.0 mg, 0.22 mmol, 74%) as a colorless oil.

TLC: R_f = 0.30 (50% EtOAc/hexane, $KMnO_4$ stain).

^{1}H NMR (400 MHz, $CDCl_3$): δ_H 8.54 (bs, 1H), 7.11 – 6.98 (m, 3H), 6.88 – 6.79 (m, 1H), 3.78 (d, *J* = 11.0 Hz, 3H), 2.02 – 1.90 (m, 2H), 1.74 – 1.61 (m, 2H), 1.49 – 1.38 (m, 2H), 0.93 (t, *J* = 7.3 Hz, 3H) ppm.

^{13}C NMR (101 MHz, $CDCl_3$): δ_C 147.9 (d, *J* = 2.9 Hz), 138.9 (d, *J* = 9.5 Hz), 126.6 (d, *J* = 1.4 Hz), 121.8 (d, *J* = 4.4 Hz), 120.8, 119.3 (d, *J* = 1.3 Hz), 53.5 (d, *J* = 7.5 Hz), 24.4 (d, *J* = 138.8 Hz), 24.2 (d, *J* = 5.7 Hz), 23.7 (d, *J* = 17.5 Hz), 13.6 ppm.

^{31}P NMR (162 MHz, $CDCl_3$): δ_P 36.7 (s) ppm.

IR (film) ν_{max}: 2962, 1595, 1495, 1465, 1370, 1097, 985, 940, 830, 703 cm^{-1}.

HRMS (ESI$^+$): calcd. for $C_{11}H_{18}O_4P$ $[M+H]^+$ 245.0937, found 245.0944.

2-hydroxyphenyl methyl octylphosphonate (**3c**)

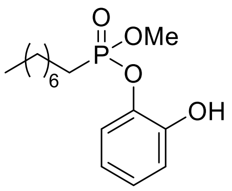

Prepared following **General Procedure E**. Purification by flash column chromatography (50% EtOAc/hexane) gave the title compound (69.3 mg, 0.23 mmol, 77%) as a colorless oil.

TLC: R_f = 0.30 (50% EtOAc/hexane, $KMnO_4$ stain).

^{1}H NMR (400 MHz, $CDCl_3$): δ_H 8.45 (bs, 1H), 7.12 – 6.99 (m, 3H), 6.88 – 6.79 (m, 1H), 3.78 (d, *J* = 11.0 Hz, 3H), 2.00 – 1.90 (m, 2H), 1.75 – 1.63 (m, 2H), 1.45 – 1.35 (m, 2H), 1.35 – 1.20 (m, 8H), 0.88 (t, *J* = 7.1 Hz, 3H) ppm.

^{13}C NMR (101 MHz, $CDCl_3$): δ_C 147.8 (d, *J* = 3.1 Hz), 138.9 (d, *J* = 9.5 Hz), 126.6, 121.8 (d, *J* = 4.2 Hz), 120.8, 119.8, 53.5 (d, *J* = 7.5 Hz), 31.8, 30.5 (d, *J* = 17.0 Hz), 29.0, 24.6 (d, *J* = 138.8 Hz), 22.7, 22.2 (d, *J* = 5.7 Hz), 14.2 ppm.

^{31}P NMR (162 MHz, $CDCl_3$): δ 36.6 (s) ppm.

IR (film) ν_{max}: 2928, 1598, 1495, 1465, 1365, 1172, 1098, 939, 830, 703 cm^{-1}.

HRMS (ESI$^+$): calcd. for $C_{15}H_{25}NaO_4P$ [M+Na]$^+$ 323.1383, found 323.1391.

2-hydroxyphenyl methyl isobutylphosphonate (**3d**)

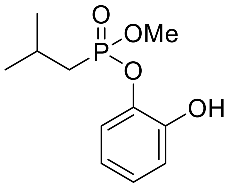

Prepared following **General Procedure E**. Purification by flash column chromatography (50% EtOAc/hexane) gave the title compound (66.0 mg, 0.27 mmol, 90%) as a colorless oil.

TLC: R_f = 0.30 (50% EtOAc/hexane, $KMnO_4$ stain).

^{1}H NMR (400 MHz, $CDCl_3$): δ_H 7.77 (bs, 1H), 7.09 – 6.99 (m, 3H), 6.86 – 6.80 (m, 1H), 3.77 (d, *J* = 11.0 Hz, 3H), 2.25 – 2.10 (m, 1H), 1.99 – 1.82 (m, 2H), 1.11 – 1.05 (m, 6H) ppm.

^{13}C NMR (101 MHz, $CDCl_3$): δ_C 147.9 (d, *J* = 2.9 Hz), 138.9 (d, *J* = 9.6 Hz), 126.6 (d, *J* = 1.5 Hz), 121.8 (d, *J* = 4.3 Hz), 120.8 (d, *J* = 1.2 Hz), 119.8 (d, *J* = 1.4 Hz), 53.3 (d, *J* = 7.5 Hz), 33.4 (d, *J* = 136.7 Hz), 24.1 (d, *J* = 12.2 Hz), 23.9 (d, *J* = 11.0 Hz), 23.8 (d, *J* = 4.6 Hz) ppm.

^{31}P NMR (162 MHz, $CDCl_3$): δ_P 35.6 (s) ppm.

IR (film) ν_{max}: 2963, 1595, 1495, 1464, 1371, 1169, 10998, 1033, 937, 828, 702 cm^{-1}.

HRMS (ESI$^+$): calcd. for $C_{11}H_{18}O_4P$ [M+H]$^+$ 245.0937, found 245.0938.

2-hydroxyphenyl methyl (cyclohexylmethyl)phosphonate (**3e**)

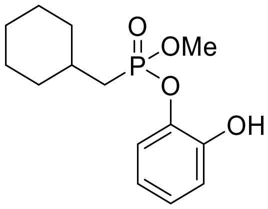

Prepared following **General Procedure E**. Purification by flash column chromatography (50% EtOAc/hexane) gave the title compound (69.1 mg, 0.24 mmol, 81%) as a colorless oil.

TLC: R_f = 0.30 (50% EtOAc/hexane, $KMnO_4$ stain).

^{1}H NMR (400 MHz, $CDCl_3$): δ_H 8.58 (bs, 1H), 7.09 – 6.98 (m, 3H), 6.86 – 6.81 (m, 1H), 3.77 (d, *J* = 11.1 Hz, 3H), 1.95 – 1.78 (m, 5H), 1.75 – 1.60 (m, 3H), 1.35 – 1.22 (m, 2H), 1.20 – 1.11 (m, 1H), 1.11 – 0.98 (m, 2H) ppm.

^{13}C NMR (101 MHz, $CDCl_3$): δ_C 147.9 (d, *J* = 3.1 Hz), 138.9 (d, *J* = 9.7 Hz), 126.6, 121.9 (d, *J* = 4.3 Hz), 120.8, 119.9, 53.4 (d, *J* = 7.5 Hz), 34.5 (d, *J* = 11.8 Hz), 34.4 (d, *J* = 10.6 Hz), 32.6 (d, *J* = 4.5 Hz), 32.1 (d, *J* = 136.3

Hz), 26.1, 25.9 (d, J = 14.4 Hz) ppm.

^{31}P NMR (162 MHz, $CDCl_3$): δ_P 36.2 (s) ppm.

IR (film) ν_{max}: 2972, 1595, 1494, 1449, 1263, 1097, 1042, 904, 831, 725 cm^{-1}.

HRMS (ESI$^+$): calcd. for $C_{14}H_{22}O_4P$ [M+H]$^+$ 285.1250, found 285.1262.

2-hydroxyphenyl methyl (3,3-dimethylbutyl)phosphonate (**3f**)

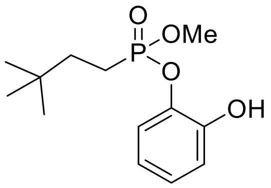

Prepared following **General Procedure E**. Purification by flash column chromatography (50% EtOAc/hexane) gave the title compound (60.0 mg, 0.22 mmol, 73%) as a yellow oil.

TLC: R_f = 0.2 (50% EtOAc/hexane, $KMnO_4$ stain).

^{1}H NMR (400 MHz, $CDCl_3$): δ_H 8.55 (bs, 1H), 7.12 – 7.00 (m, 3H), 6.88 – 6.81 (m, 1H), 3.78 (d, J = 11.0 Hz, 3H), 1.98 – 1.85 (m, 2H), 1.64 – 1.53 (m, 2H), 0.90 (s, 9H) ppm.

^{13}C NMR (101 MHz, $CDCl_3$): δ_C 147.8 (d, J = 2.9 Hz), 138.8 (d, J = 9.5 Hz), 126.6 (d, J = 1.5 Hz), 121.7 (d, J = 4.3 Hz), 120.8, 119.8 (d, J = 1.4 Hz), 53.5 (d, J = 7.5 Hz), 35.5 (d, J = 5.7 Hz), 30.4 (d, J = 17.8 Hz), 28.6, 20.2 (d, J = 140.1 Hz) ppm.

^{31}P NMR (162 MHz, $CDCl_3$): δ_P 38.0 (s) ppm.

IR (film) ν_{max}: 2958, 1597, 1495, 1458, 1366, 1172, 986, 939, 831, 703 cm^{-1}.

HRMS (ESI$^+$): calcd. for $C_{13}H_{22}O_4P$ [M+H]$^+$ 273.1250, found 273.1246.

3-((2-hydroxyphenoxy)(methoxy)phosphoryl)propyl benzoate (**3g**)

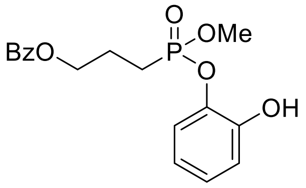

Prepared following **General Procedure E**. Purification by flash column chromatography (50% EtOAc/hexane) gave the title compound (57.4 mg, 0.16 mmol, 55%) as a colorless oil.

TLC: R_f = 0.30 (50% EtOAc/hexane, $KMnO_4$ stain).

^{1}H NMR (400 MHz, $CDCl_3$): δ_H 8.04 – 8.00 (m, 2H), 7.60 – 7.54 (m, 1H), 7.47 – 7.42 (m, 2H), 7.11 – 7.01 (m, 3H), 6.86 – 6.81 (m, 1H), 4.45 – 4.35 (m, 2H), 3.81 (d, J = 11.0 Hz, 3H), 2.25 – 2.08 (m, 4H) ppm.

^{13}C NMR (101 MHz, $CDCl_3$): δ_C 166.5, 147.7 (d, J = 3.0 Hz), 138.7 (d, J = 9.5 Hz), 133.2, 130.0, 129.7, 128.5, 126.8, 121.7 (d, J = 4.5 Hz), 121.0, 120.0, 64.2 (d, J = 18.0 Hz), 53.7 (d, J = 7.5 Hz), 22.1 (d, J = 5.0 Hz), 21.6 (d, J = 142.4 Hz) ppm.

^{31}P NMR (162 MHz, $CDCl_3$): δ_P 35.0 (s) ppm.

IR (film) v_{max}: 2970, 1718(s), 1601, 1495, 1452, 1365, 1216, 1115, 981, 828, 711 cm^{-1}.

HRMS (ESI$^+$): calcd. for $C_{17}H_{19}NaO_6P$ [M+Na]$^+$ 373.0811, found 373.0812.

2-hydroxyphenyl methyl (3-phenoxypropyl)phosphonate (**3h**)

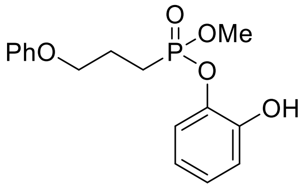

Prepared following **General Procedure E**. Purification by flash column chromatography (50% EtOAc/hexane) gave the title compound (65.0 mg, 0.20 mmol, 67%) as a yellow oil.

TLC: R_f = 0.30 (50% EtOAc/hexane, $KMnO_4$ stain).

^{1}H NMR (400 MHz, $CDCl_3$): δ_H 8.19 (bs, 1H), 7.32 – 7.24 (m, 2H), 7.10 – 7.01 (m, 3H), 6.98 – 6.93 (m, 1H), 6.91 – 6.81 (m, 3H), 4.08 – 3.97 (m, 2H), 3.81 (d, J = 11.1 Hz, 3H), 2.25 – 2.11 (m, 4H) ppm.

^{13}C NMR (101 MHz, $CDCl_3$): δ_C 158.6, 147.8 (d, J = 3.0 Hz), 138.7 (d, J = 9.5 Hz), 129.6, 126.7 (d, J = 1.7 Hz), 121.8 (d, J = 4.2 Hz), 121.1, 120.9, 119.7 (d, J = 1.5 Hz), 114.6, 66.9 (d, J = 17.0 Hz), 53.6 (d, J = 7.4 Hz), 22.3 (d, J = 5.0 Hz), 21.4 (d, J = 141.8 Hz) ppm.

^{31}P NMR (162 MHz, $CDCl_3$): δ_P 35.7 (s) ppm.

IR (film) v_{max}: 3054, 1599, 1495, 1470, 1364, 1241, 1171, 984, 939, 829, 691 cm^{-1}.

HRMS (ESI$^+$) calcd. for $C_{16}H_{20}O_5P$ [M+H]$^+$ 323.1043, found 323.1037.

2-hydroxyphenyl methyl (2-((tetrahydro-2H-pyran-2-yl)oxy)ethyl)phosphonate (**3i**)

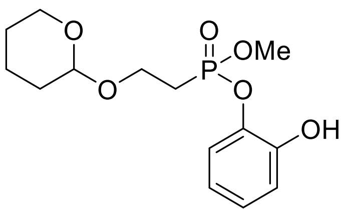

Prepared following **General Procedure E**. Purification by flash column chromatography (80% EtOAc/hexane) gave the title compound (57.0 mg, 0.18 mmol, 60%) as a yellow oil. The product was formed as a 1.2:1 mixture of diastereomers with respect to phosphorus chiral center (as determined by ^{31}P NMR analysis).

TLC: R_f = 0.2 (80% EtOAc/hexane, $KMnO_4$ stain).

^{1}H NMR (400 MHz, $CDCl_3$): δ_H 7.14 – 7.01 (m, 2H), 7.04 – 6.97 (m, 1H), 6.87 – 6.78 (m, 1H), 4.69 – 4.62 (m, 1H), 4.17 – 4.02 (m, 1H), 3.91 – 3.71 (m, 5H), 3.57 – 3.49 (m, 1H), 2.40 – 2.25 (m, 2H), 1.86 – 1.67 (m, 2H), 1.65 – 1.47 (m, 4H) ppm.

^{13}C NMR (101 MHz, $CDCl_3$) (~1:1 mixture of diastereomers): δ_C 147.8 (d, *J* = 3.7 Hz), 147.8 (d, *J* = 3.7 Hz), 138.3 (d, *J* = 9.2 Hz), 138.3 (d, *J* = 9.2 Hz), 126.7 (d, *J* = 1.7 Hz), 126.6 (d, *J* = 1.9 Hz), 121.9 (d, *J* = 3.7 Hz), 121.9 (d, *J* = 3.7 Hz), 120.6 (d, *J* = 1.6 Hz), 120.6 (d, *J* = 1.7 Hz), 119.0 (d, *J* = 10.0 Hz), 118.9 (dd, *J* = 7.4 Hz), 99.1 (d, *J* = 11.7 Hz), 99.1 (d, *J* = 11.7 Hz), 62.5 (d, *J* = 13.3 Hz), 62.5 (d, *J* = 13.3 Hz), 61.1 (d, *J* = 5.0 Hz), 61.0 (d, *J* = 4.9 Hz), 53.5 (d, *J* = 7.1 Hz), 53.4 (d, *J* = 7.1 Hz), 30.3 (d, *J* = 10.3 Hz), 30.3 (d, *J* = 10.3 Hz), 26.7 (d, *J* = 8.3 Hz), 26.7 (d, *J* = 8.3 Hz), 26.0 (d, *J* = 139.9 Hz), 26.0 (d, *J* = 139.9 Hz), 25.4 (d, *J* = 5.8 Hz), 25.3 (d, *J* = 2.6 Hz), 19.4 (d, *J* = 3.1 Hz), 19.4 (d, *J* = 3.1 Hz) ppm.

^{31}P NMR (162 MHz, $CDCl_3$): δ_P 31.4/31.2 (2s).

IR (film) ν_{max}: 2947, 1601, 1497, 1459, 1369, 1175, 1100, 936, 829, 702 cm^{-1}.

HRMS (ESI$^+$): calcd. for $C_{14}H_{21}NaO_6P$ $[M+Na]^+$ 339.0968, found 339.0975.

2-hydroxyphenyl methyl (3-bromopropyl)phosphonate (**3j**)

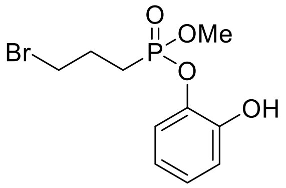

Prepared following **General Procedure E**. Purification by flash column chromatography (50% EtOAc/hexane) gave the title compound (38.0 mg, 0.12 mmol, 41%) as a yellow oil.

TLC: R_f = 0.2 (50% EtOAc/hexane, $KMnO_4$ stain).

^{1}H NMR (400 MHz, $CDCl_3$): δ_H 7.12 – 7.01 (m, 3H), 6.89 – 6.82 (m, 1H), 6.50 (s, 1H), 3.81 (d, *J* = 11.1 Hz, 3H), 3.54 – 3.44 (m, 2H), 2.32 – 2.09 (m, 4H) ppm.

^{13}C NMR (101 MHz, $CDCl_3$): δ_C 147.7 (d, *J* = 2.9 Hz), 138.7 (d, *J* = 9.4 Hz), 126.9 (d, *J* = 1.6 Hz), 121.7 (d, *J* = 4.2 Hz), 121.0 (d, *J* = 1.4 Hz), 119.9 (d, *J* = 1.2 Hz), 53.8 (d, *J* = 7.4 Hz), 33.0 (d, *J* = 19.3 Hz), 25.6 (d, *J* = 4.2 Hz), 23.5 (d, *J* = 142.2 Hz) ppm.

^{31}P NMR (162 MHz, $CDCl_3$): δ_P 34.5 (s) ppm.

IR (film) ν_{max}: 3054, 1597, 1495, 1459, 1361, 1172, 1098, 994, 939, 826, 703 cm^{-1}.

HRMS (ESI$^+$): calcd. for $C_{10}H_{15}BrO_4P$ $[M+H]^+$ 308.9886, found 308.9892.

2-hydroxyphenyl methyl (3-cyanopropyl)phosphonate (**3k**)

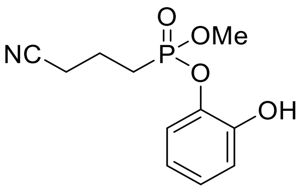

Prepared following **General Procedure E**. Purification by flash column chromatography (100% EtOAc) gave the title compound (37.0 mg, 0.14 mmol, 48%) as a yellow oil.

TLC: R_f = 0.2 (100% EtOAc, $KMnO_4$ stain).

^{1}H NMR (400 MHz, $CDCl_3$): δ_H 7.12 – 7.01 (m, 3H), 6.89 – 6.82 (m, 1H), 6.50 (s, 1H), 3.81 (d, *J* = 11.1 Hz, 3H), 3.55 – 3.43 (m, 2H), 2.32 – 2.10 (m, 4H) ppm.

^{13}C NMR (101 MHz, $CDCl_3$): δ_C 147.6 (d, *J* = 2.9 Hz), 138.6 (d, *J* = 9.2 Hz), 126.8 (d, *J* = 1.6 Hz), 121.6 (d, *J* = 4.2 Hz), 120.9 (d, *J* = 1.4 Hz), 119.8 (d, *J* = 1.2 Hz), 53.7 (d, *J* = 7.4 Hz), 32.9 (d, *J* = 19.3 Hz), 25.5 (d, *J* = 4.2 Hz), 23.4 (d, *J* = 142.2 Hz) ppm.

^{31}P NMR (162 MHz, $CDCl_3$): δ_P 34.5 (s) ppm.

IR (film) ν_{max}: 3054, 1736(s) 1560, 1495, 1422, 1372, 988, 940, 830, 703 cm^{-1}.

HRMS (ESI$^+$): calcd. for $C_{11}H_{24}NNaO_4P$ [M+Na]$^+$ 278.0553, found 278.0561.

2-hydroxyphenyl methyl (2-(9*H*-carbazol-9-yl)ethyl)phosphonate (**3l**)

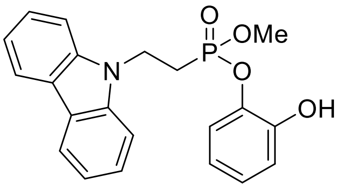

Prepared following **General Procedure E**. Purification by flash column chromatography (80% EtOAc/hexane) gave the title compound (78.6 mg, 0.21 mmol, 69%) as a colorless oil.

TLC: R_f = 0.20 (80% EtOAc/hexane, $KMnO_4$ stain).

^{1}H NMR (400 MHz, $CDCl_3$): δ_H 8.25 (bs, 1H), 7.99 (d, *J* = 7.7 Hz, 2H), 7.43 – 7.34 (m, 2H), 7.30 (d, *J* = 8.3 Hz, 2H), 7.19 – 7.14 (m, 2H), 7.02 – 6.92 (m, 2H), 6.88 (dt, *J* = 8.1, 1.5 Hz, 1H), 6.78 – 6.72 (m, 1H), 4.69 – 4.49 (m, 2H), 3.65 (d, *J* = 11.2 Hz, 3H), 2.52 – 2.30 (m, 2H) ppm.

^{13}C NMR (101 MHz, $CDCl_3$): δ_C 147.7 (d, *J* = 3.0 Hz), 139.6, 138.4 (d, *J* = 9.2 Hz), 127.0 (d, *J* = 1.6 Hz), 126.1, 123.3, 121.7 (d, *J* = 4.3 Hz), 121.0, 120.6, 119.8 (d, *J* = 1.4 Hz), 119.6, 108.5, 53.7 (d, *J* = 7.5 Hz), 36.6, 24.3 (d, *J* = 136.4 Hz) ppm.

^{31}P NMR (162 MHz, $CDCl_3$): δ_P 31.1 (s) ppm.

IR (film) ν_{max}: 3051, 1697, 1595, 1495, 1485, 1453, 1326, 1234, 1174, 1001, 937, 830, 725 cm^{-1}.

HRMS (ESI+): calcd. for $C_{21}H_{21}NO_4P$ [M+H]+ 382.1207, found 382.1209.

2-hydroxyphenyl methyl sec-butylphosphonate (**3m**)

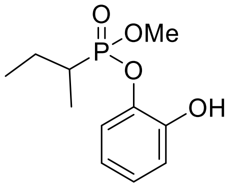

Prepared following **General Procedure E**. Purification by flash column chromatography (50% EtOAc/hexane) gave the title compound (25.6 mg, 0.10 mmol, 35%) as a yellow oil. The product was formed as a 1.2:1 mixture of diastereomers with respect to phosphorus chiral center (as determined by ^{31}P NMR analysis).

TLC: R_f = 0.2 (50% EtOAc/hexane, $KMnO_4$ stain).

^{1}H NMR (400 MHz, $CDCl_3$): δ_H 8.74 (s, 1H), 7.11 – 6.99 (m, 3H), 6.87 – 6.81 (m, 1H), 3.75 (dd, *J* = 10.7, 3.3 Hz, 3H), 2.09 – 1.87 (m, 2H), 1.61 – 1.42 (m, 1H), 1.30 (dd, *J* = 19.7, 7.5 Hz, 3H), 1.08 – 1.01 (m, 3H) ppm.

^{13}C NMR (101 MHz, $CDCl_3$) (~1:1 mixture of diastereomers): δ_C 147.8 (d, *J* = 2.6 Hz), 147.8 (d, *J* = 2.9 Hz), 139.2 (d, *J* = 5.7 Hz), 139.1 (d, *J* = 5.2 Hz), 126.6 (d, *J* = 1.5 Hz), 126.5 (d, *J* = 1.6 Hz), 121.9 (d, *J* = 5.7 Hz), 121.8 (d, *J* = 3.4 Hz), 120.8 (d, *J* = 1.2 Hz), 120.8 (d, *J* = 1.2 Hz), 120.0 (d, *J* = 1.6 Hz), 120.0 (d, *J* = 1.7 Hz), 53.8 (d, *J* = 7.9 Hz), 53.7 (d, *J* = 7.9 Hz), 32.1 (d, *J* = 137.5 Hz), 31.9 (d, *J* = 137.7 Hz), 23.3 (d, *J* = 11.6 Hz), 23.2 (d, *J* = 12.0 Hz), 12.8 (d, *J* = 7.0 Hz), 12.8 (d, *J* = 3.4 Hz), 12.1 (d, *J* = 11.9 Hz), 11.9 (d, *J* = 11.6 Hz) ppm.

^{31}P NMR (162 MHz, $CDCl_3$): δ_P 39.0/39.1 (2s) ppm.

IR (film) ν_{max}: 3131, 1593, 1495, 1453, 1172, 989, 938, 895, 830, 703 cm^{-1}.

HRMS (ESI+): calcd. for $C_{11}H_{18}O_4P$ [M+H]+ 245.0937, found 245.0936.

2-hydroxyphenyl methyl cyclopentylphosphonate (**3n**)

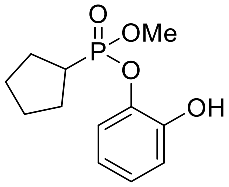

Prepared following **General Procedure E**. Purification by flash column chromatography (50% EtOAc/hexane) gave the title compound (56.0 mg, 0.22 mmol, 73%) as a yellow oil.

TLC: R_f = 0.3 (50% EtOAc/hexane, $KMnO_4$ stain).

^{1}H NMR (400 MHz, $CDCl_3$): δ_H 8.67 (bs, 1H), 7.08 – 6.98 (m, 3H), 6.85 – 6.79 (m, 1H), 3.77 (d, *J* = 10.8 Hz, 3H), 2.44 – 2.28 (m, 1H), 2.05 – 1.80 (m, 4H), 1.79 – 1.68 (m, 2H), 1.67 – 1.55 (m, 2H) ppm.

^{13}C NMR (101 MHz, $CDCl_3$): δ_C 147.9 (d, *J* = 2.7 Hz), 139.1 (d, *J* = 9.9 Hz), 126.4 (d, *J* = 1.5 Hz), 121.8 (d, *J* =

4.5 Hz), 120.7 (d, *J* = 1.2 Hz), 119.8 (d, *J* = 1.3 Hz), 53.6 (d, *J* = 7.9 Hz), 34.2 (d, *J* = 143.5 Hz), 27.2 (d, *J* = 4.6 Hz), 27.1 (d, *J* = 4.2 Hz), 26.5 (d, *J* = 12.1 Hz), 26.4 (d, *J* = 12.2 Hz) ppm.

^{31}P NMR (162 MHz, $CDCl_3$): δ_P 39.5 (s) ppm.

IR (film) ν_{max}: 2960, 1594, 1494, 1372, 1172, 1033, 938, 827 cm^{-1}.

HRMS (ESI$^+$): calcd. for $C_{12}H_{18}O_4P$ [M+H]$^+$ 257.0937, found 257.0946.

2-hydroxyphenyl methyl bicyclo[2.2.1]heptan-2-ylphosphonate (**3o**)

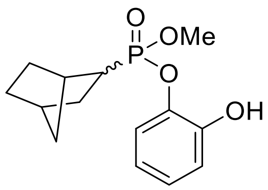

Prepared following **General Procedure E**. Purification by flash column chromatography (50% EtOAc/hexane) gave the title compound (67.8 mg, 0.24 mmol, 80%) as a colorless oil. The product was formed as a 1.3:1 mixture of diastereomers with respect to phosphorus chiral center (as determined by ^{31}P NMR analysis).

TLC: R_f = 0.30 (50% EtOAc/hexane, $KMnO_4$ stain).

^{1}H NMR (400 MHz, $CDCl_3$): δ_H 8.70 (d, *J* = 3.3 Hz, 1H), 7.11 – 6.97 (m, 3H), 6.88 – 6.78 (m, 1H), 3.81 – 3.71 (m, 3H), 2.69 – 2.61 (m, 1H), 2.40 – 2.33 (m, 1H), 2.03 – 1.77 (m, 2H), 1.72 – 1.67 (m, 1H), 1.66 – 1.51 (m, 3H), 1.35 – 1.19 (m, 3H) ppm.

^{13}C NMR (101 MHz, $CDCl_3$) (~1:1 mixture of diastereomers): δ_C 147.93 (d, *J* = 2.9 Hz), 147.85 (d, *J* = 3.0 Hz), 139.3 (d, *J* = 10.0 Hz), 139.2 (d, *J* = 10.3 Hz), 126.54 (d, *J* = 1.6 Hz), 126.52 (d, *J* = 1.6 Hz), 121.92 (d, *J* = 4.6 Hz), 121.88 (d, *J* = 4.6 Hz), 120.83 (d, *J* = 1.5 Hz), 120.82 (d, *J* = 1.5 Hz), 119.93 (d, *J* = 1.8 Hz), 119.91 (d, *J* = 1.8 Hz), 53.8 (d, *J* = 7.9 Hz), 53.7 (d, *J* = 7.8 Hz), 38.29 (d, *J* = 20.5 Hz), 38.27 (d, *J* = 20.3 Hz), 37.47 (d, *J* = 139.7 Hz), 37.45 (d, *J* = 139.7 Hz), 37.2 (d, *J* = 1.7 Hz, 2 overlapping signals), 36.31 (d, *J* = 4.1 Hz), 36.29 (d, *J* = 4.2 Hz), 32.1 (d, *J* = 6.5 Hz), 32.0 (d, *J* = 6.0 Hz), 31.71 (d, *J* = 19.0 Hz), 31.69 (d, *J* = 19.0 Hz), 28.40 (d, *J* = 2.1 Hz), 28.35 (d, *J* = 2.1 Hz) ppm.

^{31}P NMR (162 MHz, $CDCl_3$): δ_P 37.4/37.2 (2s) ppm.

IR (film) ν_{max}: 2951, 1593, 1495, 1453, 1097, 1049, 938, 831, 703 cm^{-1}.

HRMS (ESI$^+$): calcd. for $C_{14}H_{20}O_4P$ [M+H]$^+$ 283.1094, found 283.1104.

2-Hydroxyphenyl methyl ((1r,3r,5r,7r)-adamantan-2-yl)phosphonate (3p)

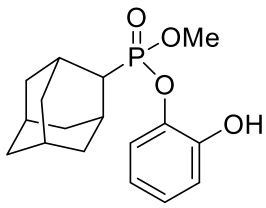

Prepared following **General Procedure E**. Purification by flash column chromatography (40% EtOAc/hexane) gave the title compound (50.0 mg, 0.16 mmol, 52%) as a yellow oil.

TLC: R_f = 0.3 (40% EtOAc/hexane, $KMnO_4$ stain).

^{1}H NMR (400 MHz, $CDCl_3$): δ_H 8.80 (s, 1H), 7.12 – 6.98 (m, 3H), 6.88 – 6.79 (m, 1H), 3.75 (d, *J* = 10.8 Hz, 3H), 2.47 (d, *J* = 21.9 Hz, 1H), 2.42 – 2.29 (m, 4H), 2.00 – 1.85 (m, 4H), 1.83 – 1.75 (m, 4H), 1.71 – 1.63 (m, 2H) ppm.

^{13}C NMR (101 MHz, $CDCl_3$): δ_C 147.7 (d, *J* = 2.7 Hz), 139.3 (d, *J* = 10.5 Hz), 126.4 (d, *J* = 1.2 Hz), 121.8 (d, *J* = 4.5 Hz), 120.7, 119.9 (d, *J* = 1.5 Hz), 53.5 (d, *J* = 7.9 Hz), 42.8 (d, *J* = 135.7 Hz), 39.3 (d, *J* = 7.7 Hz), 39.1 (d, *J* = 7.8 Hz), 37.3, 32.8 (d, *J* = 5.7 Hz), 27.9 (d, *J* = 3.2 Hz), 27.9 (d, *J* = 2.9 Hz), 27.68 (d, *J* = 2.0 Hz), 27.1 ppm.

^{31}P NMR (162 MHz, $CDCl_3$): δ_P 35.8 (s) ppm.

IR (film) ν_{max}: 2913, 1593, 1494, 1453, 1372, 1098, 1050, 937, 832, 703 cm^{-1}.

HRMS (ESI$^+$): calcd. for $C_{17}H_{23}NaO_4P$ $[M+Na]^+$ 345.1226, found 345.1241.

3-((2-hydroxyphenoxy)(methoxy)phosphoryl)propyl 2-(4-isobutylphenyl)propanoate (3r)

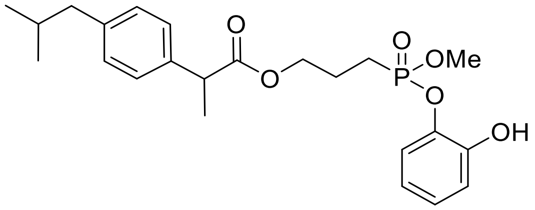

Prepared following **General Procedure E**. Purification by flash column chromatography (80% EtOAc/hexane) gave the title compound (41.0 mg, 0.09 mmol, 31%) as a yellow oil. The diastereomeric ratio of the product with respect to phosphorus chiral center could not be determined by NMR analysis.

TLC: R_f = 0.2 (80% EtOAc/hexane, $KMnO_4$ stain).

^{1}H NMR (400 MHz, $CDCl_3$): δ_H 7.21 – 7.14 (m, 2H), 7.12 – 6.95 (m, 5H), 6.89 – 6.80 (m, 1H), 4.19 – 4.06 (m, 2H), 3.75 (dd, *J* = 11.0, 1.3 Hz, 3H), 3.69 (q, *J* = 7.1 Hz, 1H), 2.44 (d, *J* = 7.2 Hz, 2H), 2.06 – 1.77 (m, 5H), 1.48 (d, *J* = 7.1 Hz, 3H), 0.89 (d, *J* = 6.6 Hz, 6H) ppm.

^{13}C NMR (101 MHz, $CDCl_3$): δ_C 174.7, 147.8 (d, *J* = 3.1 Hz), 140.8, 138.7 (d, *J* = 9.4 Hz), 137.6 (d, *J* = 2.0 Hz), 129.4, 127.2, 126.8 (d, *J* = 1.4 Hz), 121.7 (d, *J* = 4.2 Hz), 120.9, 119.9, 63.7 (d, *J* = 17.8 Hz), 53.6 (d, *J* = 7.4 Hz), 45.1 (d, *J* = 2.2 Hz), 45.1, 30.2, 22.2, 21.9, 20.5 (d, *J* = 138.9 Hz), 18.4 (d, *J* = 3.3 Hz) ppm.

^{31}P NMR (162 MHz, $CDCl_3$): δ_P 35.1 (s) ppm.

IR (film) v_{max}: 2956, 1732(s), 1595, 1495, 1459, 1157, 1034, 983, 939, 829, 702 cm^{-1}.

HRMS (ESI$^+$): calcd. for $C_{23}H_{31}NaO_6P$ [M+Na]$^+$ 457.1750, found 457.1757.

3-((2-hydroxyphenoxy)(methoxy)phosphoryl)propyl 2-(1-methyl-1H-indol-3-yl)acetate (**3s**)

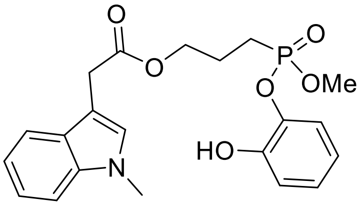

Prepared following **General Procedure A**. Purification by flash column chromatography (80% EtOAc/hexane) gave the title compound (30.0 mg, 0.07 mmol, 24%) as a colorless oil.

TLC: R_f = 0.20 (80% EtOAc/hexane, $KMnO_4$ stain).

^{1}H NMR (400 MHz, $CDCl_3$): δ_H 7.51 (d, J = 7.9 Hz, 1H), 7.21 (d, J = 8.2 Hz, 1H), 7.18 – 7.11 (m, 1H), 7.08 – 6.88 (m, 5H), 6.78 – 6.72 (m, 1H), 4.14 – 4.01 (m, 2H), 3.71 – 3.61 (m, 8H), 1.99 – 1.73 (m, 4H) ppm.

^{13}C NMR (101 MHz, $CDCl_3$): δ_C 172.1, 147.8 (d, J = 3.1 Hz), 138.6 (d, J = 9.5 Hz), 137.0, 127.8, 126.7 (d, J = 1.5 Hz), 121.8, 121.7 (d, J = 4.3 Hz), 120.9 (d, J = 1.0 Hz), 119.7 (d, J = 1.3 Hz), 119.8, 118.9, 109.4, 106.7, 63.8 (d, J = 17.9 Hz), 53.6 (d, J = 7.4 Hz), 32.7, 31.3, 21.9 (d, J = 5.0 Hz), 21.6 (d, J = 142.1 Hz) ppm.

^{31}P NMR (162 MHz, $CDCl_3$): δ_P 35.0 (s) ppm.

IR (film) v_{max}: 3054, 1736(s), 1602, 1496, 1472, 1374, 1332, 1167, 987, 939, 829, 702 cm^{-1}.

HRMS (ESI$^+$): calcd. for $C_{21}H_{24}NaNO_6P$ [M+Na]$^+$ 440.1233, found 440.1246.

3-((2-hydroxyphenoxy)(methoxy)phosphoryl)propyl (1*R*,4*S*)-4,7,7-trimethyl-3-oxo-2-oxabicyclo[2.2.1]heptane-1-carboxylate (**3t**)

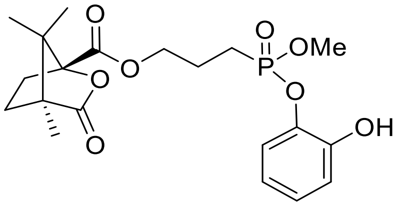

Prepared following **General Procedure E**. Purification by flash column chromatography (80% EtOAc/hexane) gave the title compound (25.0 mg, 0.06 mmol, 20%) as a yellow oil. The product was formed as a 1.1:1 mixture of diastereomers with respect to phosphorus chiral center (as determined by ^{31}P NMR analysis).

TLC: R_f = 0.20 (80% EtOAc/hexane, $KMnO_4$ stain).

^{1}H NMR (400 MHz, $CDCl_3$): δ_H 7.13 – 6.99 (m, 3H), 6.90 – 6.81 (m, 2H), 4.88 (s, 1H), 4.38 – 4.22 (m, 3H), 3.81

(d, *J* = 11.0 Hz, 2H), 2.47 – 2.35 (m, 1H), 2.19 – 1.96 (m, 5H), 1.98 – 1.86 (m, 1H), 1.75 – 1.64 (m, 1H), 1.12 (s, 3H), 1.04 (s, 3H), 0.95 (d, *J* = 9.3 Hz, 3H) ppm.

^{13}C NMR (101 MHz, $CDCl_3$): δ_C 178.2, 167.5, 147.7 (d, *J* = 3.2 Hz), 138.7 (d, *J* = 9.2 Hz), 126.9 (d, *J* = 1.4 Hz), 121.6 (d, *J* = 4.2 Hz), 121.0 (d, *J* = 1.2 Hz), 119.8 (d, *J* = 1.4 Hz), 91.1, 64.7 (d, *J* = 17.7 Hz), 54.6 (d, *J* = 56.5 Hz), 53.8 (d, *J* = 7.6 Hz), 30.8, 29.0, 22.01 (d, *J* = 143.1 Hz), 21.9, 21.9, 20.7, 16.9, 9.8 ppm.

^{31}P NMR (162 MHz, $CDCl_3$): δ_P 34.47/34.46 (2s) ppm.

IR (film) v_{max}: 3051, 1788(s), 1593, 1495, 1450, 1170, 1104, 934, 895, 703 cm^{-1}.

HRMS (ESI^+): calcd. for $C_{20}H_{27}NaO_8P$ $[M+Na]^+$ 449.1335, found 449.1328.

3-((2-hydroxyphenoxy)(methoxy)phosphoryl)propyl (4*R*)-4-((8*R*,9*S*,10*S*,13*R*,14*S*,17*R*)-10,13-dimethyl-3,7,12-trioxohexadecahydro-1*H*-cyclopenta[a]phenanthren-17-yl)pentanoate (**3u**)

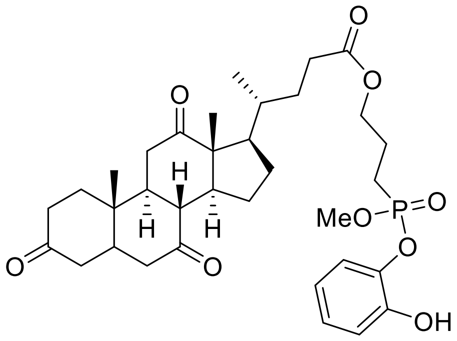

Prepared following **General Procedure E**. Purification by flash column chromatography (90% EtOAc/hexane) gave the title compound (121.5 mg, 0.19 mmol, 64%) as a colorless oil. The product was formed as a 1:1 mixture of diastereomers with respect to phosphorus chiral center (as determined by ^{31}P NMR analysis).

TLC: R_f = 0.20 (100% EtOAc, $KMnO_4$ stain).

^{1}H NMR (400 MHz, $CDCl_3$): δ_H 8.28 (s, 1H), 7.11 – 6.97 (m, 3H), 6.87 – 6.78 (m, 1H), 4.16 – 4.08 (m, 2H), 3.78 (dd, *J* = 11.1, 1.7 Hz, 3H), 2.94 – 2.77 (m, 3H), 2.42 – 2.09 (m, 10H), 2.09 – 1.89 (m, 9H), 1.87 – 1.76 (m, 2H), 1.59 (td, *J* = 14.4, 4.8 Hz, 1H), 1.44 – 1.18 (m, 4H), 1.37 (s, 3H), 1.04 (d, *J* = 1.1 Hz, 3H), 0.83 (dd, *J* = 6.6, 2.7 Hz, 3H) ppm.

^{13}C NMR (101 MHz, $CDCl_3$): δ_C 212.1 (d, *J* = 4.3 Hz), 209.1 (d, *J* = 2.9 Hz), 208.7, 173.9, 147.7 (d, *J* = 3.2 Hz), 138.6 (d, *J* = 9.4 Hz), 126.7, 121.7 (dd, *J* = 4.3, 1.8 Hz), 120.9, 119.7, 63.5 (d, *J* = 18.0 Hz), 56.9, 51.8, 49.0, 46.9, 45.6 (d, *J* = 7.0 Hz), 45.6 (d, *J* = 1.6 Hz), 45.0, 42.8, 38.7, 36.1, 36.0, 35.4 (d, *J* = 1.3 Hz), 35.3, 31.3 (d, *J* = 2.7 Hz), 30.4, 27.7, 25.2, 21.8 (d, *J* = 5.0 Hz), 21.5 (d, *J* = 142.5 Hz), 18.7, 11.9 ppm.

^{31}P NMR (162 MHz, $CDCl_3$): δ_P 34.87/34.81 (2s) ppm.

IR (film) v_{max}: 3055, 1712(s), 1595, 1496, 1465, 1173, 987, 895, 702 cm^{-1}.

HRMS (ESI^+): calcd. for $C_{34}H_{47}NaO_9P$ $[M+Na]^+$ 653.2850, found 653.2819.

2-hydroxyphenyl methyl (4-chlorophenethyl)phosphonate (**3v**)

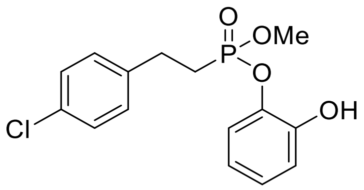

Prepared following **General Procedure F**. Purification by flash column chromatography (30% EtOAc/hexane) gave the title compound (57.0 mg, 0.17 mmol, 87%) as a colorless oil.

TLC: R_f = 0.20 (40% EtOAc/hexane, $KMnO_4$ stain).

^{1}H NMR (400 MHz, $CDCl_3$): δ_H 7.27 (d, *J* = 6.5 Hz, 2H), 7.16 – 7.10 (m, 2H), 7.08 (ddd, *J* = 7.0, 1.6, 0.9 Hz, 1H), 7.04 (dd, *J* = 8.1, 1.9 Hz, 1H), 7.00 (dt, *J* = 8.1, 1.5 Hz, 1H), 6.85 (dddd, *J* = 7.9, 7.0, 1.9, 0.8 Hz, 1H), 3.78 (d, *J* = 11.1 Hz, 3H), 2.98 (ddd, *J* = 11.7, 10.0, 7.7 Hz, 2H), 2.33 – 2.18 (m, 2H) ppm.

^{13}C NMR (101 MHz, $CDCl_3$): δ_C 147.8 (d, *J* = 3.4 Hz), 138.7 (d, *J* = 9.2 Hz), 138.5 (d, *J* = 17.2 Hz), 132.7, 129.6, 129.0, 126.9 (d, *J* = 1.8 Hz), 121.7 (d, *J* = 4.5 Hz), 121.0 (d, *J* = 1.4 Hz), 119.9 (d, *J* = 1.7 Hz), 53.7 (d, *J* = 7.8 Hz), 27.8 (d, *J* = 4.9 Hz), 26.5 (d, *J* = 138.4 Hz) ppm.

^{31}P NMR (162 MHz, $CDCl_3$): δ_P 34.1 (s) ppm.

IR (film) ν_{max}: 3155, 2955, 1594, 1513, 1492, 1459, 1407, 1373, 1292, 1260, 1234, 1172, 1093, 1034, 937, 827, 750, 654 cm^{-1}.

HRMS (ESI^+): calcd. for $C_{15}H_{16}O_4PCl$ $[M+H]^+$ 327.0547, found 327.0539.

2-hydroxyphenyl methyl (4-bromophenethyl)phosphonate (**3w**)

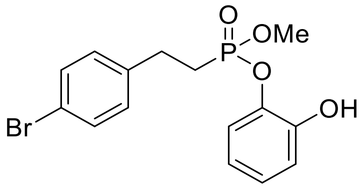

Prepared following **General Procedure F**. Purification by flash column chromatography (30% EtOAc/hexane) gave the title compound (60.0 mg, 0.16 mmol, 81%) as a colorless oil.

TLC: R_f = 0.20 (40% EtOAc/hexane, $KMnO_4$ stain).

^{1}H NMR (400 MHz, $CDCl_3$): δ_H 7.45 – 7.37 (m, 2H), 7.12 – 7.01 (m, 4H), 6.98 (dt, *J* = 8.1, 1.4 Hz, 1H), 6.84 (dddd, *J* = 8.0, 7.0, 1.9, 0.9 Hz, 1H), 3.75 (d, *J* = 11.0 Hz, 3H), 3.00 – 2.88 (m, 2H), 2.30 – 2.16 (m, 2H) ppm.

^{13}C NMR (101 MHz, $CDCl_3$): δ_C 147.7 (d, *J* = 3.2 Hz), 139.0 (d, *J* = 17.4 Hz), 131.9, 131.5, 129.9, 126.9, 121.8, 121.1 (d, *J* = 1.5 Hz), 120.7, 119.9, 53.7 (d, *J* = 7.7 Hz), 27.9 (d, *J* = 4.8 Hz), 26.4 (d, *J* = 138.7 Hz) ppm.

^{31}P NMR (162 MHz, $CDCl_3$): δ_P 34.6 (s) ppm.

IR (film) ν_{max}: 2922, 2853, 1595, 1488, 1459, 1494, 1229, 1178, 1056, 1033, 1010, 985, 935, 822, 800, 751,

628 cm^{-1}.

HRMS (ESI$^+$): calcd. for $C_{15}H_{16}O_4PBr$ [M+H]$^+$ 371.0042, found 371.0054.

2-hydroxyphenyl methyl (2-cyclopentylethyl)phosphonate (**3x**)

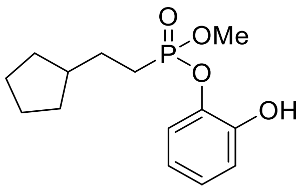

Prepared following **General Procedure F**. Purification by flash column chromatography (25% EtOAc/hexane) gave the title compound (50.0 mg, 0.17 mmol, 88%) as a colorless oil.

TLC: R_f = 0.35 (50% EtOAc/hexane, $KMnO_4$ stain).

^{1}H NMR (400 MHz, $CDCl_3$): δ_H 8.60 (bs, 1H) 7.13 – 7.00 (m, 3H), 6.84 (dddd, *J* = 7.9, 7.0, 2.0, 0.8 Hz, 1H), 3.78 (d, *J* = 11.0 Hz, 3H), 2.04 – 1.91 (m, 2H), 1.87 – 1.73 (m, 3H), 1.73 – 1.63 (m, 2H), 1.61 (dtt, *J* = 7.6, 4.8, 1.7 Hz, 2H), 1.56 – 1.47 (m, 2H), 1.15 – 1.03 (m, 2H) ppm.

^{13}C NMR (101 MHz, $CDCl_3$): δ_C 147.9 (d, *J* = 2.9 Hz), 138.9 (d, *J* = 9.6 Hz), 126.7 (d, *J* = 1.9 Hz), 121.8 (d, *J* = 4.4 Hz), 120.9 (d, *J* = 1.5 Hz), 119.9 (d, *J* = 2.0 Hz), 53.6 (d, *J* = 7.5 Hz), 40.8 (d, *J* = 17.4 Hz), 32.3 (d, *J* = 1.9 Hz), 28.3 (d, *J* = 5.7 Hz), 25.2, 23.9 (d, *J* = 138.7 Hz) ppm.

^{31}P NMR (162 MHz, $CDCl_3$): δ_P 37.5 (s) ppm.

IR (film) ν_{max}: 2947, 2865, 1596, 1495, 1458, 1261, 1169, 1097, 986, 933, 827, 749, 699, 457 cm^{-1}.

HRMS (ESI$^+$): calcd. for $C_{14}H_{21}O_4P$ [M+H]$^+$ 285.1250, found 285.1245.

2-hydroxyphenyl methyl neopentylphosphonate (**3y**)

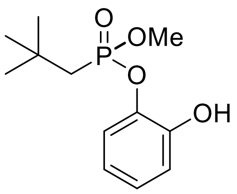

Prepared following **General Procedure F**. Purification by flash column chromatography (20% acetone/hexane) gave the title compound (34.0 mg, 0.13 mmol, 66%) as a colorless oil.

TLC: R_f = 0.30 (20% acetone/hexane, $KMnO_4$ stain).

^{1}H NMR (500 MHz, $CDCl_3$): δ_H 8.66 (bs, 1H), 7.13 – 6.93 (m, 3H), 6.84 (tdd, *J* = 7.1, 2.2, 0.8 Hz, 1H), 3.75 (d, *J* = 11.1 Hz, 3H), 1.99 (dd, *J* = 18.8, 2.6 Hz, 2H), 1.15 (d, *J* = 1.1 Hz, 9H) ppm.

^{13}C NMR (126 MHz, $CDCl_3$): δ_C 147.9 (d, *J* = 2.9 Hz), 139.0 (d, *J* = 10.0 Hz), 126.6 (d, *J* = 1.6 Hz), 122.0 (d, *J* = 4.7 Hz), 120.9 (d, *J* = 1.2 Hz), 120.0 (d, *J* = 1.6 Hz), 53.3 (d, *J* = 7.7 Hz), 38.3 (d, *J* = 134.9 Hz), 31.0 (d, *J* =

9.7 Hz), 30.2 (d, *J* = 3.9 Hz) ppm.

^{31}P NMR (162 MHz, $CDCl_3$): δ_P 35.6 (s) ppm.

IR (film) ν_{max}: 3159, 2955, 2904, 1593, 1514, 1495, 1459, 1369, 1291, 1228, 1174, 1130, 1100, 1039, 934, 828, 751, 702, 596, 569, 479, 456, 431 cm^{-1}.

HRMS (ESI$^+$): calcd. for $C_{12}H_{19}O_4P$ $[M+H]^+$ 259.1094, found 259.1096.

2-hydroxyphenyl methyl (2-(thiophen-2-yl)ethyl)phosphonate (**3z**)

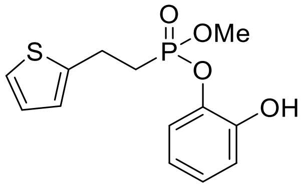

Prepared following **General Procedure F**. Purification by flash column chromatography (30% EtOAc/hexane) gave the title compound (47.0 mg, 0.16 mmol, 79%) as a yellow oil.

TLC: R_f = 0.25 (50% EtOAc/hexane, $KMnO_4$ stain).

^{1}H NMR (400 MHz, $CDCl_3$): δ_H 7.16 (dt, *J* = 5.1, 1.1 Hz, 1H), 7.09 (tdt, *J* = 7.0, 1.7, 0.9 Hz, 1H), 7.06 – 6.99 (m, 2H), 6.93 (dd, *J* = 5.1, 3.5 Hz, 1H), 6.89 – 6.82 (m, 2H), 3.79 (d, *J* = 11.0 Hz, 2H), 3.31 – 3.14 (m, 2H), 2.46 – 2.31 (m, 2H) ppm.

^{13}C NMR (101 MHz, $CDCl_3$): δ_C ^{13}C NMR 147.8, 142.6 (d, *J* = 20.2 Hz), 138.7 (d, *J* = 9.2 Hz), 127.1, 124.9 (d, *J* = 10.1 Hz), 124.0, 121.6 (d, *J* = 4.3 Hz), 121.0, 119.8 (d, *J* = 1.4 Hz), 118.6, 53.7, 27.1 (d, *J* = 138.3 Hz), 22.9 (d, *J* = 4.0 Hz) ppm.

^{31}P NMR (162 MHz, $CDCl_3$): δ_P 34.1 (s) ppm.

IR (film) ν_{max}: 2922, 2853, 1596, 1494, 1459, 1260, 1230, 1169, 1032, 985, 925, 827, 750, 694, 458 cm^{-1}.

HRMS (ESI$^+$): calcd. for $C_{13}H_{16}O_4P$ $[M+H]^+$ 299.1886, found 299.1892.

2-hydroxyphenyl methyl (4-bromobutyl)phosphonate (**3aa**)

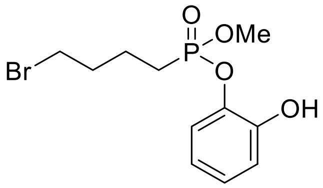

Prepared following **General Procedure F**. Purification by flash column chromatography (30% EtOAc/hexane) gave the title compound (47.0 mg, 0.15 mmol, 74%) as a colorless oil.

TLC: R_f = 0.33 (40% EtOAc/hexane, $KMnO_4$ stain).

^{1}H NMR (400 MHz, $CDCl_3$): δ_H 7.12 – 6.99 (m, 3H), 6.85 (dddd, *J* = 8.0, 7.1, 1.9, 0.8 Hz, 1H), 6.10 (bs, 2H),

3.80 (d, J = 11.0 Hz, 3H), 3.41 (t, J = 6.3 Hz, 2H), 2.05 – 1.94 (m, 4H), 1.94 – 1.81 (m, 2H) ppm.

^{13}C NMR (101 MHz, $CDCl_3$): δ_C 147.8 (d, J = 3.0 Hz), 138.8 (d, J = 9.5 Hz), 126.8 (d, J = 1.4 Hz), 121.8 (d, J = 4.4 Hz), 121.0, 119.9, 53.7 (d, J = 7.5 Hz), 33.0 (d, J = 16.2 Hz), 32.5 (d, J = 1.6 Hz), 23.8 (d, J = 140.3 Hz), 21.0 (d, J = 5.3 Hz) ppm.

^{31}P NMR (162 MHz, $CDCl_3$): δ_P 35.2 (s) ppm.

IR (film) ν_{max}: 3055, 1599, 1493, 1459, 1362, 1172, 1098, 995, 939, 827, 704 cm^{-1}.

HRMS (ESI$^+$): calcd. for $C_{11}H_{17}BrO_4P$ [M+H]$^+$ 322.9881, found 322.9882.

2-hydroxyphenyl methyl pent-4-yn-1-ylphosphonate (**3ab**)

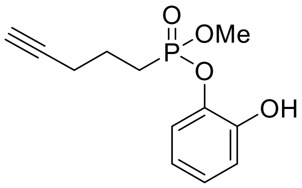

Prepared following **General Procedure F**. Purification by flash column chromatography (50% EtOAc/hexane) gave the title compound (31.0 mg, 0.12 mmol, 60%) as a colorless oil.

TLC: R_f = 0.22 (50% EtOAc/hexane, $KMnO_4$ stain).

^{1}H NMR (400 MHz, $CDCl_3$): δ_H 8.48 (bs, 1H), 7.14 – 7.00 (m, 3H), 6.85 (dddd, J = 7.9, 7.0, 2.0, 0.8 Hz, 1H), 3.80 (d, J = 11.0 Hz, 3H), 2.35 (tdt, J = 6.6, 2.5, 1.3 Hz, 2H), 2.18 – 2.08 (m, 2H), 2.02 (t, J = 2.6 Hz, 1H), 1.99 – 1.87 (m, 2H) ppm.

^{13}C NMR (101 MHz, $CDCl_3$): δ_C δ 147.8, 138.8 (d, J = 9.7 Hz), 126.8 (d, J = 1.4 Hz), 121.9, 121.0, 120.0 (d, J = 1.4 Hz), 82.5, 70.1, 53.7 (d, J = 7.7 Hz), 23.5 (d, J = 141.1 Hz), 21.4 (d, J = 4.4 Hz), 19.2 (d, J = 18.3 Hz) ppm.

^{31}P NMR (162 MHz, $CDCl_3$): δ_P 36.3 (s) ppm.

IR (film) ν_{max}: 3289, 2955, 2117, 1593, 1513, 1495, 1459, 1230, 1172, 1033, 936, 929, 751, 640, 507 cm^{-1}.

HRMS (ESI$^+$): calcd. for $C_{12}H_{15}O_4P$ [M+H]$^+$ 255.0781, found 255.0778.

2-hydroxyphenyl methyl (2,2,3,3-tetramethylcyclopropyl)phosphonate (**3ac**)

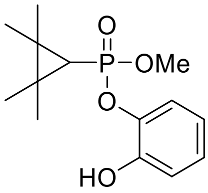

Prepared following **General Procedure F**. Purification by flash column chromatography (30% EtOAc/hexane) gave the title compound (48 mg, 0.17 mmol, 85%) as a colorless oil.

TLC: R_f = 0.31 (40% EtOAc/hexane, $KMnO_4$ stain).

^{1}H NMR (400 MHz, $CDCl_3$): δ_H 8.61 (bs, 1H), 7.11 – 7.02 (m, 2H), 6.99 (dt, J = 7.9, 1.5 Hz, 1H), 6.86 (dddd, J = 7.9, 6.9, 2.0, 0.8 Hz, 1H), 3.84 (d, J = 11.2 Hz, 3H), 1.34 (d, J = 0.7 Hz, 3H), 1.22 (d, J = 2.2 Hz, 3H), 1.02 (d, J = 2.3 Hz, 3H), 0.99 (s, 3H), 0.46 (s, 1H) ppm.

^{13}C NMR (126 MHz, $CDCl_3$): δ_C 148.5 (d, J = 2.8 Hz), 138.6 (d, J = 7.7 Hz), 126.6 (d, J = 1.5 Hz), 122.2 (d, J = 4.2 Hz), 120.7 (d, J = 1.1 Hz), 119.8 (d, J = 1.4 Hz), 52.9 (d, J = 6.8 Hz), δ 29.1 (d, J = 5.0 Hz), 28.9 (d, J = 5.4 Hz), 27.1 (d, J = 183.4 Hz), 23.83 (d, J = 16.0 Hz), 23.80 (d, J = 16.4 Hz), 18.3 (d, J = 5.9 Hz), 17.9 (d, J = 5.2 Hz).

^{31}P NMR (162 MHz, $CDCl_3$): δ_P 35.2 (s) ppm.

IR (film) ν_{max}: 3058, 2949, 1712, 1593, 1514, 1494, 1453, 1291, 1264, 1222, 1175, 1042, 934, 920, 813, 831, 734, 702, 714 cm^{-1}.

HRMS (ESI$^+$): calcd. for $C_{14}H_{21}O_4P$ [M+H]$^+$ 285.1250, found 285.1247.

2-hydroxyphenyl methyl (2,3-dihydro-1H-inden-2-yl)phosphonate (**3ad**)

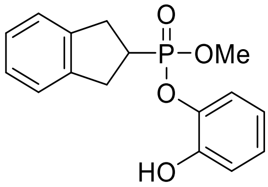

Prepared following **General Procedure F and Condition B**. Purification by flash column chromatography (50% EtOAc/hexane) gave the title compound (52.0 mg, 0.17 mmol, 86%) as a colorless oil.

TLC: R_f = 0.2 (50% EtOAc/hexane, $KMnO_4$ stain).

^{1}H NMR (400 MHz, $CDCl_3$): δ_H 8.51 (bs, 1H), 7.25 – 7.16 (m, 4H), 7.11 – 7.03 (m, 2H), 6.99 (dt, J = 8.1, 1.4 Hz, 1H), 6.83 (dddd, J = 7.9, 6.9, 2.2, 0.8 Hz, 1H), 3.82 (d, J = 10.8 Hz, 3H), 3.46 – 3.19 (m, 4H), 2.99 (dp, J = 16.1, 9.2 Hz, 1H) ppm.

^{13}C NMR (101 MHz, $CDCl_3$): δ_C 147.7 (d, J = 2.9 Hz), 141.0 (dd, J = 12.5, 6.9 Hz), 139.0 (d, J = 9.6 Hz), 127.1, 126.7 (d, J = 1.3 Hz), 124.5, 121.7 (d, J = 4.4 Hz), 120.9, 120.0 (d, J = 1.3 Hz), 53.9 (d, J = 7.6 Hz), 34.1 (d, J = 146.4 Hz), 33.9 (d, J = 7.0 Hz), 33.8 (d, J = 7.0 Hz) ppm.

^{31}P NMR (162 MHz, $CDCl_3$): δ_P 37.1 (s) ppm.

IR (film) ν_{max}: 2956, 1722, 1593, 1513, 1494, 1459, 1293, 1263, 1033, 1047, 938, 831, 732, 701 cm^{-1}.

HRMS (ESI$^+$): calcd. for $C_{14}H_{22}O_4P$ [M+H]$^+$ 285.2937, found 285.2946.

***tert*-butyl (*2R*)-2-((((9H-fluoren-9-yl)methoxy)carbonyl)amino)-3-((2-hydroxyphenoxy)(methoxy)phosphoryl)propanoate** (**3af**)

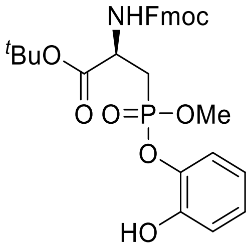

Prepared following **General Procedure F**. Purification by flash column chromatography (80% EtOAc/hexane) gave the title compound (74.0 mg, 0.13 mmol, 67%) as a colorless oil. The product was formed as a 1.1:1 mixture of diastereomers with respect to phosphorus chiral center (as determined by ^{31}P NMR analysis).

TLC: R_f = 0.20 (80% EtOAc/hexane, $KMnO_4$ stain).

^{1}H NMR (600 MHz, $CDCl_3$) (mixture of diastereomers): δ_H 7.77 – 7.72 (m, 2H), 7.66 (s, 1H), 7.62 – 7.56 (m, 2H), 7.42 – 7.35 (m, 2H), 7.31 – 7.24 (m, 2H), 7.16 – 7.11 (m, 1H), 7.09 – 6.73 (m, 4H), 6.02 (dd, *J* = 32.6, 8.1 Hz, 1H), 4.73 – 4.61 (m, 1H), 4.44 – 4.30 (m, 2H), 4.25 – 4.18 (m, 1H), 3.76 – 3.69 (m, 3H), 2.76 – 2.64 (m, 1H), 2.62 – 2.50 (m, 1H), 1.50 – 1.42 (m, 9H).

^{13}C NMR (151 MHz, $CDCl_3$) (~1:1 mixture of diastereomers): δ_C 169.6 (d, J = 10.6 Hz), 169.4 (d, J = 11.0 Hz), 156.1, 156.0, 147.84 (d, J = 3.9 Hz), 147.77 (d, J = 4.1 Hz), 143.84, 143.81, 143.77, 141.45 – 141.23 (m), 138.0 (d, J = 9.9 Hz), 127.9, 127.8, 127.2 (d, J = 3.5 Hz), 126.8, 125.36 – 125.10 (m), 121.86 – 121.67 (m), 120.7, 120.1, 120.0, 119.99, 119.14, 119.08, 83.7, 83.6, 67.5, 53.9 (d, J = 7.4 Hz), 53.8 (d, J = 7.4 Hz), 49.98 (d, *J* = 7.5 Hz), 49.91 (d, *J* = 7.7 Hz), 47.1, 28.0, 27.91 (d, *J* = 144.3 Hz), 27.88, 27.85 (d, *J* = 143.7 Hz) ppm.

^{31}P NMR (162 MHz, $CDCl_3$): δ_P 29.38/29.11 (2s) ppm.

IR (film) ν_{max}: 3317, 2980, 1720, 1595, 1513, 1497, 1369, 1339, 1259, 1230, 1150, 1033, 939, 832, 754, 736, 701 cm^{-1}.

HRMS (ESI$^+$): calcd. for $C_{29}H_{32}NO_8P$ [M+H]$^+$ 554.1938, found 554.1933.

Benzyl (2*S*)-2-((*tert*-butoxycarbonyl)amino)-4-((2-hydroxyphenoxy)(methoxy)phosphoryl)butanoate (**3ag**)

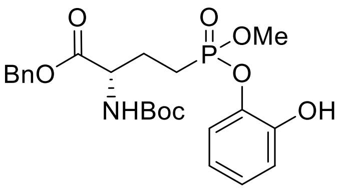

Prepared following **General Procedure F**. Purification by flash column chromatography (80% EtOAc/hexane) gave the title compound (69.0 mg, 0.14 mmol, 72%) as a colorless oil. The product was formed as a 2.1:1 mixture of diastereomers with respect to phosphorus chiral center (as determined by ^{31}P NMR analysis).

TLC: R_f = 0.20 (80% EtOAc/hexane, $KMnO_4$ stain).

^{1}H NMR (400 MHz, $CDCl_3$) (mixture of diastereomers): δ_H 8.30 (d, *J* = 5.6 Hz, 1H), 7.39 – 7.30 (m, 5H), 7.11 – 6.96 (m, 3H), 6.82 (tdt, *J* = 7.1, 2.0, 1.1 Hz, 1H), 5.17 (m, 3H), 4.42 (bs, 1H), 3.76 (dd, *J* = 11.0, 1.0 Hz, 3H), 2.39 – 2.19 (m, 1H), 2.11 – 1.84 (m, 3H), 1.43 (d, *J* = 1.9 Hz, 9H) ppm.

^{13}C NMR (101 MHz, $CDCl_3$) (mixture of diastereomers): δ_C 171.5, 147.8, 138.7 (d, J = 9.5 Hz), 135.2, 128.84, 128.81, 128.8, 128.6, 126.8, 121.8 (t, J = 4.1 Hz), 121.0 (d, J = 3.8 Hz), 119.9 (d, J = 7.6 Hz), 80.5, 67.7, 53.82 (d, J = 7.4 Hz), 53.79 (d, J = 7.5 Hz), 29.4, 28.4, 26.0, 20.9 (d, *J* = 142.6 Hz), 20.8 (d, *J* = 142.6 Hz) ppm.

^{31}P NMR (162 MHz, $CDCl_3$): δ_P 34.5/34.4 (2s) ppm.

IR (film) ν_{max}: 2973, 1709, 1594, 1496, 1456, 1366, 1238, 1159, 1047, 1033, 939, 829, 750, 697 cm^{-1}.

HRMS (ESI^+): calcd. for $C_{23}H_{30}NaNO_8P$ $[M+Na]^+$ 502.1601, found 502.1593.

***tert*-Butyl (2-(4-chlorophenyl)-3-((2-hydroxyphenoxy)(methoxy)phosphoryl)propyl)carbamate** (**3ah**)

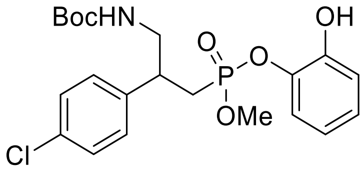

Prepared following **General Procedure F and Condition B**. Purification by flash column chromatography (80% EtOAc/hexane) gave the title compound (59.0 mg, 0.13 mmol, 65%) as a yellow oil. The diastereomeric ratio of the product could not be determined by NMR analysis.

TLC: R_f = 0.20 (80% EtOAc/hexane, $KMnO_4$ stain).

^{1}H NMR (400 MHz, $CDCl_3$): δ_H 7.41 – 7.33 (m, 2H), 7.33 – 7.21 (m, 2H), 7.13 – 6.98 (m, 2H), 6.92 – 6.87 (m, 1H), 6.83 – 6.76 (m, 1H), 4.70 (bs, 1H), 3.60 – 3.54 (m, 1H), 3.50 (d, *J* = 11.1 Hz, 3H), 3.38 – 3.25 (m, 2H), 2.47 (ddd, *J* = 18.9, 15.7, 5.4 Hz, 1H), 2.33 (ddd, *J* = 18.5, 15.5, 8.2 Hz, 1H), 1.42 (s, 9H) ppm.

^{13}C NMR (101 MHz, $CDCl_3$): δ_C 156.1, 147.7 (d, *J* = 3.4 Hz), 141.0 (d, *J* = 7.2 Hz), 138.5 (d, *J* = 9.5 Hz), 129.0, 127.8, 127.6, 126.5, 121.7 (d, *J* = 4.1 Hz), 120.7, 119.3, 79.7, 53.6 (d, *J* = 7.2 Hz), 46.7 (d, *J* = 16.7 Hz), 40.8 (d, *J* = 3.5 Hz), 29.0 (d, *J* = 146.8 Hz), 28.4 ppm.

^{31}P NMR (162 MHz, $CDCl_3$): δ_P 33.2 (s) ppm.

IR (film) ν_{max}: 3282, 2977, 1696, 1594, 1494, 1455, 1366, 1241, 1167, 1043, 937, 908, 829, 727, 698 cm^{-1}.

HRMS (ESI^+): calcd. for $C_{21}H_{28}NaNO_6P$ $[M+Na]^+$ 444.1546, found 444.1536 (*dechlorinated product mass was observed on HR-MS*).[14]

2-Hydroxyphenyl methyl (3-(4-(bis(2-chloroethyl)amino)phenyl)propyl)phosphonate (**3ai**)

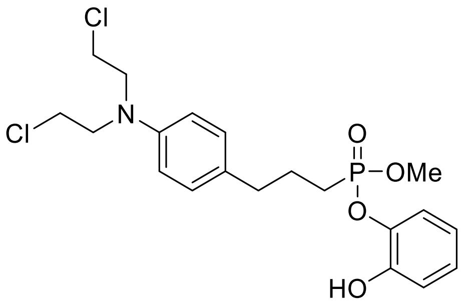

Prepared following **General Procedure F**. Purification by flash column chromatography (25% EtOAc/hexane) gave the title compound (62.0 mg, 0.14 mmol, 70%) as a yellow oil.

TLC: R_f = 0.30 (40% EtOAc/hexane, $KMnO_4$ stain).

^{1}H NMR (400 MHz, $CDCl_3$): δ_H 8.49 (bs, 1H), 7.13 – 7.02 (m, 4H), 6.99 (dt, *J* = 8.0, 1.4 Hz, 1H), 6.86 – 6.81 (m, 1H), 6.67 – 6.61 (m, 2H), 3.77 (d, *J* = 11.0 Hz, 3H), 3.74 – 3.60 (m, 8H), 2.68 – 2.60 (m, 2H), 2.05 – 1.87 (m, 4H) ppm.

^{13}C NMR (101 MHz, $CDCl_3$): δ_C 147.8 (d, *J* = 3.0 Hz), 144.7, 138.9 (d, *J* = 9.4 Hz), 129.8, 129.6, 126.7 (d, *J* = 1.4 Hz), 121.8 (d, *J* = 4.4 Hz), 120.9 (d, *J* = 1.0 Hz), 119.9 (d, *J* = 1.4 Hz), 112.3, 53.7, 53.6 (d, *J* = 7.4 Hz), 40.6, 35.2 (d, *J* = 17.5 Hz), 24.5 (d, *J* = 139.4 Hz), 24.2 (d, *J* = 5.0 Hz) ppm.

^{31}P NMR (162 MHz, $CDCl_3$): δ_P 36.5 (s) ppm.

IR (film) ν_{max}: 2957, 1614, 1518, 1494, 1293, 1263, 1178, 1047, 1033, 938, 831, 732, 701 cm^{-1}.

HRMS (ESI^+): calcd. for $C_{20}H_{26}NO_4PCl_2$ $[M+H]^+$ 446.1049, found 446.1043.

2-Hydroxyphenyl methyl (((1S,3R)-3-acetyl-2,2-dimethylcyclobutyl)methyl)phosphonate (3aj)

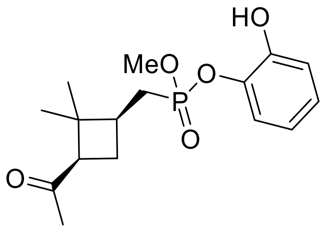

Prepared following **General Procedure F**. Purification by flash column chromatography (20% THF/hexane) gave the title compound (21.0 mg, 0.06 mmol, 32%) as a colorless oil. The product was formed as a 1.1:1 mixture of diastereomers with respect to phosphorus chiral center (as determined by ^{31}P NMR analysis).

TLC: R_f = 0.20 (50% EtOAc/hexane, $KMnO_4$ stain).

^{1}H NMR (400 MHz, $CDCl_3$) (~1:1 mixture of diastereomers): δ_H 8.52 (bs, 1H), 7.12 – 6.97 (m, 3H), 6.88 – 6.82 (m, 1H), 3.75 (d, *J* = 11.1 Hz, 3H), 2.94 – 2.87 (m, 1H), 2.46 – 2.33 (m, 1H), 2.17 – 1.83 (m, 4H), 1.43 (s, 3H), 1.33/1.32 (2×s, 3H), 0.89/0.88 (2×s, 3H) ppm.

^{13}C NMR (101 MHz, $CDCl_3$) (~1:1 mixture of diastereomers): δ_C 207.2 (d, *J* = 2.8 Hz), 147.81 (d, *J* = 3.2 Hz), 147.80 (d, *J* = 3.2 Hz), 138.9 (d, *J* = 10.0 Hz), 138.8 (d, *J* = 10.1 Hz), 126.8, 125.7, 121.91 (d, *J* = 4.7 Hz), 121.90 (d, *J* = 4.5 Hz), 121.01 (d, *J* = 1.2 Hz), 121.98 (d, *J* = 1.1 Hz), 120.08 (d, *J* = 1.5 Hz), 120.05 (d, *J* = 1.7 Hz), 54.4, 54.3, 53.7 (d, *J* = 8.1 Hz), 53.6 (d, *J* = 8.2 Hz), 43.9 (d, *J* = 9.3 Hz), 43.8 (d, *J* = 8.5 Hz), 35.8 (d, *J* = 6.6 Hz), 35.7 (d, *J* = 6.4 Hz), 30.5, 30.3, 29.88, 29.86, 25.3 (d, *J* = 137.3 Hz), 25.2 (d, *J* = 137.3 Hz), 24.4 (d, *J* = 9.3 Hz), 24.2 (d, *J* = 8.9 Hz), 17.39, 17.37 ppm.

^{31}P NMR (162 MHz, $CDCl_3$): δ_P 35.4/35.3 (2s) ppm.

IR (film) ν_{max}: 2954, 1703, 1594, 1514, 1595, 1459, 1357, 1261, 1227, 1177, 1034, 936, 831, 752 cm^{-1}.

HRMS (ESI$^+$): calcd. for $C_{16}H_{23}O_5P$ $[M+H]^+$ 327.1356, found 327.1351.

***tert*-Butyl ((1-(((2-hydroxyphenoxy)(methoxy)phosphoryl)methyl)cyclohexyl)methyl)carbamate** (**3ak**)

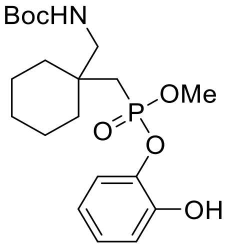

Prepared following **General Procedure F**. Purification by flash column chromatography (40% EtOAc/hexane) gave the title compound (58.0 mg, 0.14 mmol, 70%) as a colorless oil.

TLC: R_f = 0.20 (50% EtOAc/hexane, $KMnO_4$ stain).

^{1}H NMR (400 MHz, $CDCl_3$): δ_H 8.44 (bs, 1H), 7.13 – 7.00 (m, 3H), 6.84 (td, *J* = 7.6, 2.0 Hz, 1H), 5.19 (t, *J* = 6.9 Hz, 1H), 3.74 (d, *J* = 11.1 Hz, 3H), 3.46 – 3.18 (m, 2H), 2.04 (d, *J* = 19.5 Hz, 2H), 1.61 – 1.45 (m, 10H), 1.44 (s, 9H) ppm.

^{13}C NMR (101 MHz, $CDCl_3$): δ_C 156.7, 147.8 (d, *J* = 3.2 Hz), 138.7 (d, *J* = 10.1 Hz), 126.7, 121.8 (d, *J* = 4.1 Hz), 120.8, 119.6, 79.3, 53.6 (d, *J* = 7.6 Hz), 47.5, 37.5 (d, *J* = 3.0 Hz), 35.4 (d, *J* = 12.2 Hz), 34.9 (d, *J* = 10.0 Hz), 31.7 (d, *J* = 134.0 Hz), 28.5, 25.9, 21.6 ppm.

^{31}P NMR (162 MHz, $CDCl_3$): δ 35.2 (s) ppm.

IR (film) ν_{max}: 2980, 2933, 1705, 1510, 1496, 1455, 1366, 1264, 1167, 1044, 1033, 937, 831, 733, 702 cm^{-1}.

HRMS (ESI$^+$): calcd. for $C_{20}H_{32}NO_6P$ $[M+H]^+$ 414.2040, found 414.2039.

2-Hydroxyphenyl methyl (E)-(5-(4-hydroxy-6-methoxy-7-methyl-3-oxo-1,3-dihydroisobenzofuran-5-yl)-3-methylpent-3-en-1-yl)phosphonate (**3al**)

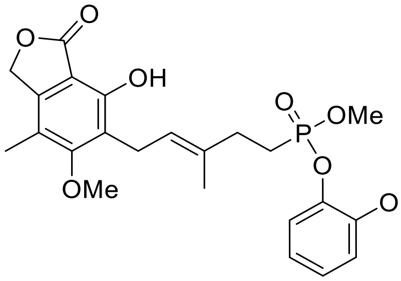

Prepared following **General Procedure F and Condition B**. Purification by flash column chromatography (50% EtOAc/hexane) gave the title compound (75.0 mg, 0.16 mmol, 81%) as a colorless oil.

TLC: R_f = 0.20 (80% EtOAc/hexane, $KMnO_4$ stain).

^{1}H NMR (500 MHz, $CDCl_3$): δ_H 7.68 (bs, 1H), 7.05 (dddd, *J* = 8.1, 7.2, 1.5, 0.9 Hz, 1H), 7.02 – 6.97 (m, 2H), 6.81 (dddd, *J* = 8.0, 7.2, 1.8, 0.7 Hz, 1H), 5.29 (tq, *J* = 7.0, 1.3 Hz, 1H), 5.18 (s, 2H), 3.76 (s, 3H), 3.74 (s, 3H), 3.38 (d, *J* = 7.0 Hz, 2H), 2.35 (td, *J* = 10.6, 6.8 Hz, 2H), 2.14 (s, 3H), 2.10 – 1.96 (m, 2H), 1.80 (s, 3H) ppm.

^{13}C NMR (126 MHz, $CDCl_3$): δ_C 173.0, 163.7, 153.7, 147.8 (d, *J* = 3.0 Hz), 144.2, 138.8 (d, *J* = 9.5 Hz), 133.8 (d, *J* = 17.2 Hz), 126.7 (d, *J* = 1.4 Hz), 123.3, 121.9, 121.8 (d, *J* = 4.3 Hz), 120.9, 119.8 (d, *J* = 1.4 Hz), 116.9, 106.5, 70.2, 61.1, 53.6 (d, *J* = 7.4 Hz), 31.7 (d, *J* = 4.7 Hz), 23.4 (d, *J* = 138.5 Hz), 22.7, 16.0, 11.7 ppm.

^{31}P NMR (162 MHz, $CDCl_3$): δ 35.9 (s) ppm.

IR (film) ν_{max}: 2939, 1730, 1620, 1495, 1456, 1329, 1292, 1274, 1262, 1229, 1190, 1032, 941, 829, 734, 752, 701 cm^{-1}.

HRMS (ESI^+): calcd. for $C_{23}H_{27}O_8P$ $[M+H]^+$ 463.1516, found 463.1512.

2-Hydroxyphenyl methyl heptadecylphosphonate (**3am**)

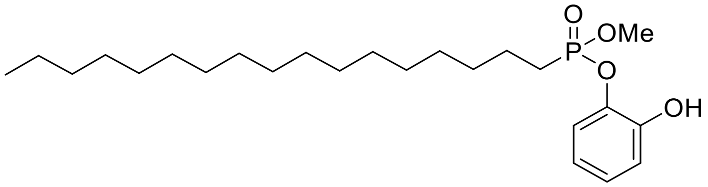

Prepared following **General Procedure F**. Purification by flash column chromatography (20% EtOAc/hexane) gave the title compound (67.0 mg, 0.16 mmol, 79%) as a colorless oil.

TLC: R_f = 0.30 (40% EtOAc/hexane, $KMnO_4$ stain).

^{1}H NMR (400 MHz, $CDCl_3$): δ 7.12 – 6.99 (m, 3H), 6.84 (tdd, *J* = 6.9, 2.1, 0.7 Hz, 1H), 3.78 (d, *J* = 11.0 Hz, 3H), 2.02 – 1.87 (m, 2H), 1.76 – 1.61 (m, 2H), 1.39 (dq, *J* = 10.4, 5.2 Hz, 2H), 1.32 – 1.16 (m, 26H), 0.91 – 0.83 (m, 3H) ppm.

^{13}C NMR (101 MHz, $CDCl_3$): δ_C 147.9 (d, *J* = 2.9 Hz), 138.9 (d, *J* = 9.6 Hz), 126.7 (d, *J* = 1.4 Hz), 121.8 (d, *J* =

4.1 Hz), 120.9, 119.9 (d, *J* = 1.4 Hz), 53.5 (d, *J* = 7.7 Hz), 32.1, 30.5 (d, *J* = 16.9 Hz), 29.83 (3C), 29.81, 29.80 (2C), 29.75, 29.7, 29.5, 29.4, 29.1 (d, *J* = 1.5 Hz), 24.6 (d, *J* = 138.3 Hz), 22.8, 22.2 (d, *J* = 5.7 Hz), 14.3 ppm.

^{31}P NMR (162 MHz, $CDCl_3$): δ 37.4 (s) ppm.

IR (film) ν_{max}: 2919, 2849, 1598, 1496, 1465, 1376, 1293, 1263, 1174, 1098, 1054, 993, 1032, 937, 829, 747, 699, 597 cm^{-1}.

HRMS (ESI$^+$): calcd. for $C_{24}H_{43}O_4P$ [M+H]$^+$ 427.2972, found 427.2968.

2-Hydroxyphenyl methyl (*Z*)-heptadec-8-en-1-ylphosphonate (**3an**)

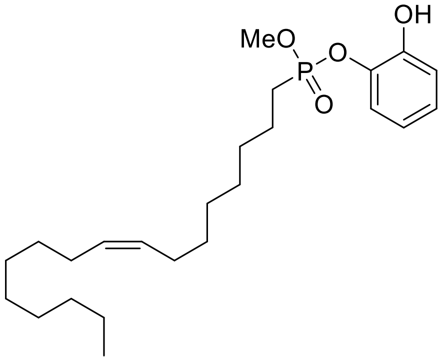

Prepared following **General Procedure F**. Purification by flash column chromatography (20% EtOAc/hexane) gave the title compound (76.0 mg, 0.18 mmol, 90%) as a colorless oil.

TLC: R_f = 0.33 (40% EtOAc/hexane, $KMnO_4$ stain).

^{1}H NMR (600 MHz, $CDCl_3$): δ_H 7.47 (bs, 1H), 7.10 – 6.95 (m, 3H), 6.86 – 6.78 (m, 1H), 5.40 – 5.29 (m, 2H), 3.78 (d, *J* = 11.0 Hz, 3H), 2.03 – 1.98 (m, 4H), 1.98 – 1.91 (m, 2H), 1.73 – 1.64 (m, 2H), 1.43 – 1.36 (m, 2H), 1.35 – 1.24 (m, 18H), 0.88 (t, *J* = 6.9 Hz, 3H) ppm.

^{13}C NMR (151 MHz, $CDCl_3$): δ_C 147.9 (d, *J* = 3.5 Hz), 138.9 (d, *J* = 9.8 Hz), 130.2, 129.8, 126.7 (d, *J* = 1.9 Hz), 121.8 (d, *J* = 4.9 Hz), 120.8, 119.9 (d, *J* = 2.0 Hz), 53.5 (d, *J* = 7.6 Hz), 32.0, 30.5 (d, *J* = 17.0 Hz), 29.9, 29.8, 29.7, 29.5, 29.5, 29.1, 29.0 (d, *J* = 1.5 Hz), 27.4, 27.3, 24.6 (d, *J* = 138.8 Hz), 22.8, 22.2 (d, *J* = 5.5 Hz), 14.2 ppm.

^{31}P NMR (162 MHz, $CDCl_3$): δ 36.5 (s) ppm.

IR (film) ν_{max}: 2922, 2853, 1596, 1512, 1496, 1460, 1871, 1293, 1274, 1236, 1259, 1174, 1097, 1045, 1033, 938, 830, 747, 699 cm^{-1}.

HRMS (ESI$^+$): calcd. for $C_{24}H_{41}NaO_4P$ [M+H]$^+$ 425.2815, found 425.2810.

Heptane-1,7-di(hydroxyphenyl methyl phosphonate (3ao)

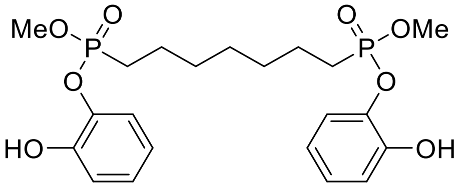

Prepared following **General Procedure F**. Purification by flash column chromatography (80% EtOAc/hexane) gave the title compound (62.0 mg, 0.13 mmol, 66%) as a yellow oil. The product was formed as a 1:1 mixture of diastereomers with respect to the two phosphorus chiral centers (as determined by ^{31}P NMR analysis).

TLC: R_f = 0.2 (80% EtOAc/hexane, $KMnO_4$ stain).

^{1}H NMR (600 MHz, $CDCl_3$): δ_H δ 7.10 – 7.04 (m, 3H), 7.04 – 7.00 (m, 3H), 6.87 – 6.81 (m, 2H), 3.78 (dd, *J* = 11.0, 2.2 Hz, 6H), 2.02 – 1.89 (m, 4H), 1.74 – 1.61 (m, 4H), 1.46 – 1.37 (m, 4H), 1.37 – 1.29 (m, 2H) ppm.

^{13}C NMR (151 MHz, $CDCl_3$) (1:1 mixture of diastereomers): δ_C 147.9 (d, *J* = 3.1 Hz), 147.8 (d, *J* = 3.1 Hz), 138.88 (d, *J* = 9.5 Hz), 138.87 (d, *J* = 9.5 Hz), 126.73 (d, *J* = 1.4 Hz), 126.71 (d, *J* = 1.5 Hz), 121.82 (d, *J* = 4.7 Hz), 121.81 (d, J = 4.5 Hz), 120.93, 120.88, 119.9 (d, *J* = 1.8 Hz), 119.8 (d, *J* = 1.8 Hz), 53.6 (d, *J* = 7.8 Hz), 53.5 (d, *J* = 7.5 Hz), 30.1 (d, *J* = 16.9 Hz), 30.0 (d, *J* = 16.9 Hz), 28.5, 28.4, 24.6 (d, *J* = 138.7 Hz), 22.12 (d, *J* = 6.1 Hz), 22.11 (d, *J* = 6.1 Hz) ppm.

^{31}P NMR (162 MHz, $CDCl_3$): δ 36.21/36.23 (2s) ppm.

IR (film) ν_{max}: 3154, 2924, 1691, 1593, 1497, 1458, 1373, 1292, 1231, 1172, 1100, 1033, 934, 828, 750 cm^{-1}.

HRMS (ESI^+): calcd. for $C_{21}H_{30}O_8P_2$ $[M+H]^+$ 417.1429, found 473.1483.

2-Hydroxyphenyl methyl ((4Z,7Z,10Z,13Z)-nonadeca-4,7,10,13-tetraen-1-yl)phosphonate (3ap)

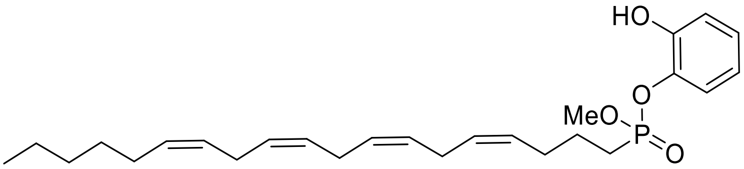

Prepared following **General Procedure F**. Purification by flash column chromatography (30% EtOAc/hexane) gave the title compound (71.0 mg, 0.16 mmol, 80%) as a colorless oil.

TLC: R_f = 0.20 (40% EtOAc/hexane, $KMnO_4$ stain).

^{1}H NMR (400 MHz, $CDCl_3$): δ_H 8.55 (bs, 1H), 7.13 – 6.98 (m, 3H), 6.85 (dddd, *J* = 8.0, 7.1, 1.9, 0.8 Hz, 1H), 5.49 – 5.28 (m, 8H), 3.78 (d, *J* = 11.0 Hz, 3H), 2.87 – 2.75 (m, 5H), 2.24 – 2.17 (m, 1H), 2.16 – 2.09 (m, 1H), 2.08 – 2.02 (m, 2H), 2.01 – 1.90 (m, 2H), 1.84 – 1.73 (m, 2H), 1.41 – 1.23 (m, 7H), 0.89 (t, *J* = 6.9 Hz, 3H) ppm.

^{13}C NMR (101 MHz, $CDCl_3$): δ_C 147.9 (d, J = 2.9 Hz), 138.9 (d, J = 9.6 Hz), 130.7, 129.8, 128.8, 128.5, 128.2, 128.1, 127.9, 127.6, 126.8 (d, J = 1.6 Hz), 121.9 (d, J = 4.5 Hz), 121.0, 120.1 (d, J = 1.4 Hz), 53.6 (d, J = 7.7 Hz), 31.7, 29.5, 27.8 (d, J = 17.9 Hz), 27.4, 24.1 (d, J = 138.9 Hz), 25.8, 22.7, 22.2 (d, J = 5.3 Hz), 14.2 ppm.

^{31}P NMR (162 MHz, $CDCl_3$): δ 36.1 (s) ppm.

IR (film) ν_{max}: 3010, 2955, 2924, 1593, 1514, 1494, 1459, 1260, 1236, 1174, 1100, 1045, 1033, 937, 831, 750 cm^{-1}.

HRMS (ESI^+): calcd. for $C_{26}H_{39}O_4P$ $[M+H]^+$ 447.2659, found 447.2655.

2-Hydroxyphenyl methyl ((*3R*)-3-((*5S,8R,10S,13R,14S,17R*)-10,13-dimethyl-3,7,12-trioxohexadecahydro-1H-cyclopenta[a]phenanthren-17-yl)butyl)phosphonate (**3aq**)

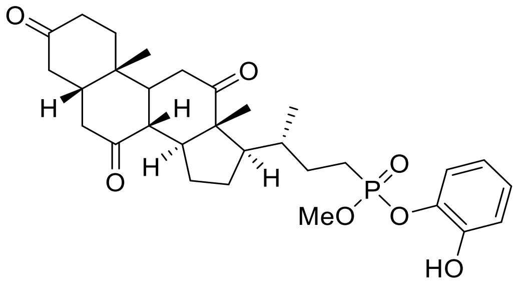

Prepared following **General Procedure F**. Purification by flash column chromatography (30% EtOAc/hexane) gave the title compound (68.0 mg, 0.12 mmol, 63%) as a colorless oil. The product was formed as a 1.5:1 mixture of diastereomers with respect to phosphorus chiral center (as determined by ^{31}P NMR analysis).

TLC: R_f = 0.30 (50% EtOAc/hexane, $KMnO_4$ stain).

^{1}H NMR (400 MHz, $CDCl_3$): δ 8.49 (bs, 1 H), 7.11 – 6.97 (m, 3H), 6.83 (tdd, *J* = 7.0, 2.1, 0.7 Hz, 1H), 3.77 (ddd, *J* = 11.0, 4.6, 0.7 Hz, 3H), 3.11 (d, *J* = 14.6 Hz, 3H), 2.95 – 2.63 (m, 3H), 2.41 – 2.09 (m, 3H), 2.13 – 1.88 (m, 6H), 1.84 (tt, *J* = 9.0, 5.1 Hz, 2H), 1.75 (ddt, *J* = 12.8, 7.1, 3.6 Hz, 2H), 1.51 (dtd, *J* = 12.7, 9.6, 4.2 Hz, 1H), 1.42 – 1.16 (m, 6H), 1.13 (d, *J* = 6.5 Hz, 1H), 1.05 (d, *J* = 4.8 Hz, 1H), 1.01 (d, *J* = 4.4 Hz, 2H), 0.84 (td, *J* = 6.4, 4.8 Hz, 3H) ppm.

^{13}C NMR (101 MHz, $CDCl_3$): δ_C 212.0 (d, *J* = 4.3 Hz), 209.0 (d, *J* = 2.9 Hz), 208.6, 173.8 (d, *J* = 1.6 Hz), 147.6 (d, *J* = 3.2 Hz), 138.5 (d, J = 9.4 Hz), 126.6 (d, J = 1.5 Hz), 121.6 (dd, J = 4.3, 1.8 Hz), 120.8 (t, J = 1.1 Hz), 119.6 (t, J = 1.6 Hz), 63.4 (d, J = 17.9 Hz), 56.8, 53.5 (dd, J = 7.5, 1.2 Hz), 51.7, 48.9, 46.8, 45.5 (dd, J = 4.3, 2.7 Hz), 44.9, 42.7, 38.5, 35.9, 35.4 (d, J = 1.3 Hz), 35.2, 31.2 (d, J = 2.7 Hz), 30.3, 27.5, 25.0, 22.0, 21.5 (d, *J* = 139.3 Hz), 21.0 (d, *J* = 2.9 Hz), 20.7, 18.6, 11.8 ppm.

^{31}P NMR (162 MHz, $CDCl_3$): δ 37.4/37.3 (2s) ppm.

IR (film) ν_{max}: 2965, 1708, 1594, 1513, 1494, 1264, 1099, 1048, 1033, 939, 830, 731, 701 cm^{-1}.

HRMS (ESI^+): calcd. for $C_{30}H_{41}O_7P$ $[M+H]^+$ 545.2663, found 545.2665.

Common examples: Deboronative and Decarboxylative

2-Hydroxyphenyl methyl phenethylphosphonate (**3ar**)

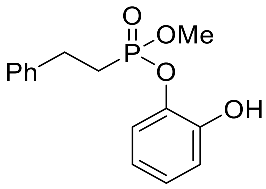

Prepared following **General Procedures E and F**. Purification by flash column chromatography (50% EtOAc/hexane) gave the title compound (25.0 mg, 0.09 mmol, 29% by GP-E) and (51.0 mg, 0.17 mmol, 88% by GP-F) as a colorless oil.

TLC: R_f = 0.30 (50% EtOAc/hexane, $KMnO_4$ stain).

^{1}H NMR (400 MHz, $CDCl_3$): δ_H 8.39 (bs, 1H), 7.35 – 7.28 (m, 2H), 7.27 – 7.18 (m, 3H), 7.12 – 6.98 (m, 3H), 6.88 – 6.81 (m, 1H), 3.79 (d, *J* = 11.0 Hz, 3H), 3.07 – 2.94 (m, 2H), 2.37 – 2.21 (m, 2H) ppm.

^{13}C NMR (101 MHz, $CDCl_3$): δ_C 147.8 (d, *J* = 2.9 Hz), 140.1 (d, *J* = 17.4 Hz), 138.7 (d, *J* = 9.6 Hz), 128.8, 128.1, 126.8, 126.8 (d, *J* = 1.6 Hz), 121.7 (d, *J* = 4.2 Hz), 120.9 (d, *J* = 1.2 Hz), 119.8 (d, *J* = 1.5 Hz), 53.6 (d, *J* = 7.5 Hz), 28.4 (d, *J* = 4.8 Hz), 26.6 (d, *J* = 137.7 Hz) ppm.

^{31}P NMR (162 MHz, $CDCl_3$): δ 34.6 (s) ppm.

IR (film) ν_{max}: 3055, 1598, 1495, 1455, 1371, 1172, 1098, 988, 938, 824, 699 cm^{-1}.

HRMS (ESI^+): calcd. for $C_{15}H_{17}NaO_4P$ $[M+Na]^+$ 315.0757, found 315.0769.

2-Hydroxyphenyl methyl (3-phenylpropyl)phosphonate (**3as**)

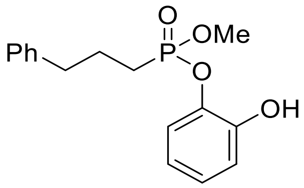

Prepared following **General Procedures E and F**. Purification by flash column chromatography (50% EtOAc/hexane) gave the title compound (74.0 mg, 0.24 mmol, 81% by GP-E) and (51.0 mg, 0.17 mmol, 84% by GP-F) as a yellow oil.

TLC: R_f = 0.3 (50% EtOAc/hexane, $KMnO_4$ stain).

^{1}H NMR (400 MHz, $CDCl_3$): δ_H 8.50 (bs, 1H), 7.35 – 7.26 (m, 2H), 7.26 – 7.13 (m, 3H), 7.12 – 6.96 (m, 3H), 6.88 – 6.79 (m, 1H), 3.77 (d, *J* = 11.0 Hz, 3H), 2.79 – 2.68 (m, 2H), 2.12 – 1.91 (m, 4H) ppm.

^{13}C NMR (101 MHz, $CDCl_3$): δ_C 147.8 (d, *J* = 3.1 Hz), 140.5, 138.8 (d, *J* = 9.5 Hz), 128.6, 128.5, 126.6 (d, *J* = 1.5 Hz), 126.3, 121.7 (d, *J* = 4.4 Hz), 120.8, 119.7 (d, *J* = 1.3 Hz), 53.5 (d, *J* = 7.5 Hz), 36.3 (d, *J* = 17.3 Hz), 24.0 (d, *J* = 140.2 Hz), 23.9 (d, *J* = 5.0 Hz) ppm.

^{31}P NMR (162 MHz, $CDCl_3$): δ 36.0 (s) ppm.

IR (film) v_{max}: 3055, 1596, 1495, 1454, 1371, 1172, 1098, 938, 827, 699 cm^{-1}.

HRMS (ESI$^+$): calcd. for $C_{16}H_{20}O_4P$ [M+H]$^+$ 307.1094, found 307.1103.

2-Hydroxyphenyl methyl but-3-en-1-ylphosphonate (**3at**)

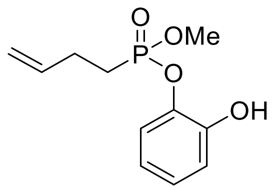

Prepared following **General Procedures E and F**. Purification by flash column chromatography (50% EtOAc/hexane) gave the title compound (14.0 mg, 0.09 mmol, 19% by GP-E) and (33.0 mg, 0.13 mmol, 68% by GP-F) as a yellow oil.

TLC: R_f = 0.3 (50% EtOAc/hexane, $KMnO_4$ stain).

^{1}H NMR (400 MHz, $CDCl_3$): δ_H 8.48 (bs, 1H), 7.13 – 6.99 (m, 3H), 6.89 – 6.80 (m, 1H), 5.91 – 5.79 (m, 1H), 5.15 – 5.02 (m, 2H), 3.79 (d, *J* = 11.1 Hz, 3H), 2.51 – 2.40 (m, 2H), 2.13 – 2.01 (m, 2H) ppm.

^{13}C NMR (101 MHz, $CDCl_3$): δ_C 147.8 (d, *J* = 3.0 Hz), 138.8 (d, *J* = 9.2 Hz), 136.4 (d, *J* = 17.0 Hz), 126.7 (d, *J* = 1.6 Hz), 121.8 (d, *J* = 4.5 Hz), 120.9 (d, *J* = 1.1 Hz), 119.9 (d, *J* = 1.3 Hz), 116.0 (d, *J* = 1.2 Hz), 53.6 (d, *J* = 7.4 Hz), 26.3 (d, *J* = 4.9 Hz), 24.1 (d, *J* = 139.7 Hz) ppm.

^{31}P NMR (162 MHz, $CDCl_3$): δ 35.4 (s) ppm.

IR (film) v_{max}: 2989, 2253, 1593, 1495, 1453, 1092, 989, 831, 702 cm^{-1}.

HRMS (ESI$^+$): calcd. for $C_{11}H_{15}NaO_4P$ [M+Na]$^+$ 265.0600, found 265.0604.

***tert*-Butyl (2-((2-hydroxyphenoxy)(methoxy)phosphoryl)ethyl)carbamate** (**3au**)

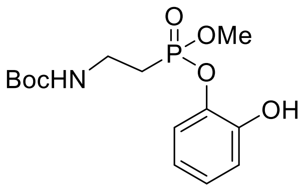

Prepared following **General Procedures E and F**. Purification by flash column chromatography (100% EtOAc) gave the title compound (60.0 mg, 0.18 mmol, 60% by GP-E) and (45.0 mg, 0.14 mmol, 69% by GP-F) as a yellow oil.

TLC: R_f = 0.2 (80% EtOAc/hexane, $KMnO_4$ stain).

^{1}H NMR (400 MHz, $CDCl_3$): δ_H 7.63 (bs, 1H), 7.10 – 6.97 (m, 3H), 6.85 – 6.79 (m, 1H), 5.14 (bs, 1H), 3.79 (d, *J* = 11.1 Hz, 3H), 3.58 – 3.43 (m, 2H), 2.33 – 2.14 (m, 2H), 1.43 (s, 9H) ppm.

^{13}C NMR (101 MHz, $CDCl_3$): δ_C 155.9, 147.8 (d, *J* = 3.4 Hz), 138.4 (d, *J* = 9.4 Hz), 126.6 (d, *J* = 1.5 Hz), 121.7 (d, *J* = 3.8 Hz), 120.7 (d, *J* = 1.2 Hz), 119.3 (d, *J* = 1.2 Hz), 80.0, 53.6 (d, *J* = 7.4 Hz), 34.6, 28.4, 25.9 (d, *J* = 138.9 Hz) ppm.

^{31}P NMR (162 MHz, $CDCl_3$): δ 32.2 (s) ppm.

IR (film) ν_{max}: 3054, 1704(s), 1593, 1495, 1453, 1372, 1169, 989, 909, 830, 703 cm^{-1}.

HRMS (ESI^+): calcd. for $C_{14}H_{23}NO_6P$ $[M+H]^+$ 332.1258, found 332.1244.

Methyl 3-((2-hydroxyphenoxy)(methoxy)phosphoryl)propanoate (**3av**)

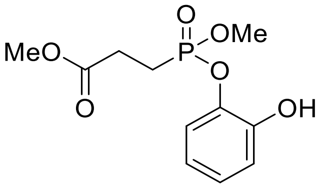

Prepared following **General Procedures E and F**. Purification by flash column chromatography (80% EtOAc/hexane) gave the title compound (69.0 mg, 0.25 mmol, 84% by GP-E) and (28.0 mg, 0.10 mmol, 52% by GP-F) as a colorless oil.

TLC: R_f = 0.20 (80% EtOAc/hexane, $KMnO_4$ stain).

^{1}H NMR (400 MHz, $CDCl_3$): δ_H 8.00 (bs, 1H), 7.10 – 6.99 (m, 3H), 6.87 – 6.81 (m, 1H), 3.80 (d, *J* = 11.1 Hz, 3H), 3.71 (s, 3H), 2.77 – 2.67 (m, 2H), 2.37 – 2.27 (m, 2H) ppm.

^{13}C NMR (101 MHz, $CDCl_3$): δ_C 172.2 (d, *J* = 17.0 Hz), 147.7 (d, *J* = 3.3 Hz), 138.5 (d, *J* = 9.4 Hz), 126.8 (d, *J* = 1.5 Hz), 121.6 (d, *J* = 4.1 Hz), 120.9, 119.6, 53.8 (d, *J* = 7.3 Hz), 52.3, 27.2 (d, *J* = 4.2 Hz), 20.3 (d, *J* = 144.5 Hz) ppm.

^{31}P NMR (162 MHz, $CDCl_3$): δ 33.3 (s) ppm.

IR (film) ν_{max}: 3055, 1736(s), 1602, 1497, 1439, 1368, 1175, 1098, 991, 939, 830, 702 cm^{-1}.

HRMS (ESI^+): calcd. for $C_{11}H_{16}O_6P$ $[M+H]^+$ 275.0682, found 275.0679.

2-Hydroxyphenyl methyl cyclohexylphosphonate (**3a**)

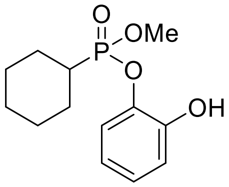

Prepared following **General Procedures E and F**. Purification by flash column chromatography (50% EtOAc/hexane) gave the title compound (65.5 mg, 0.24 mmol, 81% by GP-E) and (43.0 mg, 0.16 mmol, 81% by GP-F) as a colorless oil.

TLC: R_f = 0.30 (50% EtOAc/hexane, $KMnO_4$ stain).

^{1}H NMR (400 MHz, $CDCl_3$): δ_H 8.69 (s, 1H), 7.10 – 7.00 (m, 3H), 6.88 – 6.81 (m, 1H), 3.75 (d, *J* = 10.8 Hz, 3H), 2.13 – 1.94 (m, 3H), 1.90 – 1.80 (m, 2H), 1.75 – 1.69 (m, 1H), 1.57 – 1.43 (m, 2H), 1.34 – 1.21 (m, 3H) ppm.

^{13}C NMR (101 MHz, $CDCl_3$): δ_C 147.8 (d, *J* = 2.8 Hz), 139.3 (d, *J* = 10.3 Hz), 126.5, 121.8 (d, *J* = 4.5 Hz), 120.8, 119.9, 53.7 (d, *J* = 7.8 Hz), 35.1 (d, *J* = 139.7 Hz), 26.04 (d, *J* = 17.0 Hz), 26.00 (d, *J* = 17.0 Hz), 25.80 (d, *J* = 4.6 Hz), 25.77 (d, *J* = 2.0 Hz), 25.76 (d, *J* = 4.6 Hz) ppm.

^{31}P NMR (162 MHz, $CDCl_3$): δ 34.1 (s) ppm.

IR (film) ν_{max}: 2934, 1593, 1494, 1374, 1237, 1172, 1035, 936, 830 cm^{-1}.

HRMS (ESI$^+$): calcd. for $C_{13}H_{19}NaO_4P$ [M+Na]$^+$ 293.0913, found 293.0977.

ethyl (2-hydroxyphenyl) cyclohexylphosphonate (**3aw**)

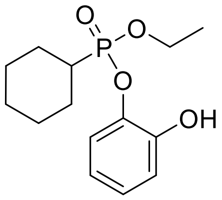

Prepared following modified **General Procedures E and F**, using ethanol instead of methanol. Purification by flash column chromatography (30% acetone/hexane) gave the title compound (63.1 mg, 0.23 mmol, 74% by GP-E) and (43.7 mg, 0.15 mmol, 77% by GP-F) as a colorless oil.

TLC: R_f = 0.30 (30% acetone/hexane, $KMnO_4$ stain).

^{1}H NMR (500 MHz, $CDCl_3$): δ_H 8.79 (s, 1H), 7.12 – 6.96 (m, 3H), 6.83 (dddd, *J* = 7.9, 7.0, 1.9, 0.8 Hz, 1H), 4.17 (ddq, *J* = 10.2, 9.3, 7.1 Hz, 1H), 4.06 (ddq, *J* = 10.2, 7.8, 7.0 Hz, 1H), 2.13 – 2.02 (m, 2H), 2.03 – 1.92 (m, 1H), 1.90 – 1.79 (m, 2H), 1.76 – 1.66 (m, 1H), 1.55 – 1.43 (m, 2H), 1.33 – 1.24 (m, 3H), 1.21 (t, *J* = 7.0 Hz, 3H) ppm.

^{13}C NMR (126 MHz, $CDCl_3$): δ_C 147.9 (d, *J* = 3.1 Hz), 139.2 (d, *J* = 10.4 Hz), 126.5 (d, *J* = 1.6 Hz), 122.0 (d, *J* = 4.6 Hz), 120.8 (d, *J* = 1.1 Hz), 119.9 (d, *J* = 1.6 Hz), 63.5 (d, *J* = 8.0 Hz), 35.3 (d, *J* = 140.1 Hz), 26.03 (d, *J* = 16.7 Hz), 25.99 (d, *J* = 16.8 Hz), 25.79 (d, *J* = 4.9 Hz), 25.75 (d, *J* = 1.9 Hz), 25.7 (d, *J* = 5.0 Hz), 16.4 (d, *J* = 5.7 Hz) ppm.

^{31}P NMR (162 MHz, $CDCl_3$): δ 36.3 (s) ppm.

IR (film) ν_{max}: 3150, 2931, 2855, 1593, 1514, 1494, 1458, 1378, 1292, 1236, 1174, 1100, 1030, 933, 893, 859, 827, 750, 706, 750, 572, 502, 474 cm^{-1}.

HRMS (ESI$^+$): calcd. for $C_{14}H_{21}O_4P$ [M+H]$^+$ 285.1250, found 285.1273.

2-hydroxyphenyl isopropyl cyclohexylphosphonate (**3ax**)

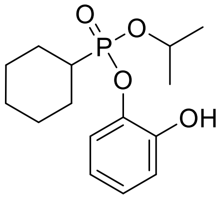

Prepared following modified **General Procedures E and F**, using isopropanol instead of methanol. Purification by flash column chromatography (20% acetone/hexane) gave the title compound (56.5 mg, 0.19 mmol, 63% by GP-E) and (40.0 mg, 0.13 mmol, 68% by GP-F) as a colorless oil.

TLC: R_f = 0.30 (20% acetone/hexane, $KMnO_4$ stain).

^{1}H NMR (500 MHz, $CDCl_3$): δ_H 8.88 (s, 1H), 7.13 – 6.98 (m, 3H), 6.94 – 6.75 (m, 1H), 4.75 (dhept, *J* = 7.6, 6.2 Hz, 1H), 2.15 – 2.05 (m, 1H), 1.97 (dtt, *J* = 18.8, 12.4, 3.2 Hz, 1H), 1.90 – 1.82 (m, 2H), 1.79 – 1.71 (m, 1H), 1.57 – 1.43 (m, 2H), 1.33 (d, *J* = 6.2 Hz, 3H), 1.36 – 1.23 (m, 3H), 1.11 (d, *J* = 6.2 Hz, 3H) ppm.

^{13}C NMR (126 MHz, $CDCl_3$): δ_C 148.0 (d, *J* = 3.1 Hz), 139.2 (d, *J* = 10.6 Hz), 126.5 (d, *J* = 1.6 Hz), 122.2 (d, *J* = 4.7 Hz), 120.7 (d, *J* = 1.2 Hz), 119.9 (d, *J* = 1.6 Hz), 72.5 (d, *J* = 8.2 Hz), 35.5 (d, *J* = 141.0 Hz), 26.1 (d, *J* = 16.8 Hz), 26.0 (d, *J* = 16.9 Hz), 25.82 (d, *J* = 4.9 Hz), 25.78 (d, *J* = 2.0 Hz), 25.7 (d, *J* = 4.8 Hz), 24.1 (d, *J* = 5.2 Hz), 23.8 (d, *J* = 4.0 Hz) ppm.

^{31}P NMR (162 MHz, $CDCl_3$): δ 35.5 (s) ppm.

IR (film) ν_{max}: 3154, 2979, 2931, 2855, 1593, 1514, 1494, 1459, 1385, 1293, 1260, 1238, 1174, 1100, 1012, 991, 934, 899, 832, 824, 750, 705, 605, 573, 556, 509, 436, 409 cm^{-1}.

HRMS (ESI^+): calcd. for $C_{15}H_{23}O_4P$ $[M+H]^+$ 299.1407, found 299.1410.

benzyl (2-hydroxyphenyl) cyclohexylphosphonate (**3ay**)

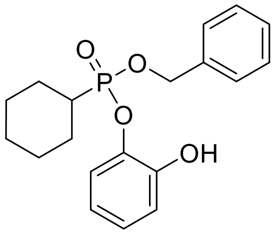

Prepared following modified **General Procedures E and F**, using benzyl alcohol instead of methanol. Purification by flash column chromatography (15% acetone/hexane) gave the title compound (78.0 mg, 0.22 mmol, 75% by GP-E) and (53.0 mg, 0.15 mmol, 76% by GP-F) as a colorless oil.

TLC: R_f = 0.27 (15% acetone/hexane, $KMnO_4$ stain).

^{1}H NMR (500 MHz, $CDCl_3$): δ_H 7.33 – 7.24 (m, 3H), 7.23 – 7.19 (m, 2H), 7.04 – 6.97 (m, 2H), 6.92 (dt, *J* = 8.0, 1.4 Hz, 1H), 6.75 (dddd, *J* = 7.8, 7.0, 2.1, 0.7 Hz, 1H), 5.08 (dd, *J* = 11.7, 8.5 Hz, 1H), 4.92 (dd, *J* = 11.7, 7.7

Hz, 1H), 2.08 – 1.99 (m, 2H), 1.98 – 1.89 (m, 1H), 1.82 – 1.72 (m, 2H), 1.69 – 1.62 (m, 1H), 1.51 – 1.38 (m, 2H), 1.27 – 1.13 (m, 3H) ppm.

^{13}C NMR (126 MHz, $CDCl_3$): δ_C 147.8 (d, *J* = 3.1 Hz), 139.1 (d, *J* = 10.7 Hz), 135.8 (d, *J* = 6.2 Hz), 128.70, 128.69, 128.1, 126.5 (d, *J* = 1.6 Hz), 122.0 (d, *J* = 4.6 Hz), 120.8 (d, *J* = 1.2 Hz), 119.9 (d, *J* = 1.6 Hz), 68.6 (d, *J* = 7.7 Hz), 35.4 (d, *J* = 139.2 Hz), 25.99 (d, *J* = 16.9 Hz), 25.95 (d, *J* = 16.8 Hz), 25.74 (d, *J* = 4.8 Hz), 25.69 (d, *J* = 4.8 Hz), 25.68 (d, *J* = 1.7 Hz) ppm.

^{31}P NMR (162 MHz, $CDCl_3$): δ_P 35.6 (s) ppm.

IR (film) ν_{max}: 3156, 2931, 2854, 1593, 1514, 1494, 1457, 1377, 1284, 1236, 1173, 1122, 1100, 1079, 1033, 1013, 998, 935, 895, 858, 832, 695, 747, 695, 604, 569, 555, 493, 465 cm^{-1}.

HRMS (ESI$^+$): calcd. for $C_{19}H_{23}O_4P$ [M+H]$^+$ 347.1407, found 347.1417.

2-Hydroxyphenyl methyl isopropylphosphonate (**3az**)

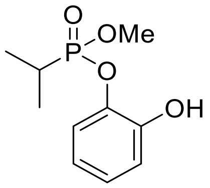

Prepared following **General Procedures E and F**. Purification by flash column chromatography (50% EtOAc/hexane) gave the title compound (31.2 mg, 0.14 mmol, 45% by GP-E) and (34.0 mg, 0.15 mmol, 74% by GP-F) as a colorless oil.

TLC: R_f = 0.30 (50% EtOAc/hexane, $KMnO_4$ stain).

^{1}H NMR (400 MHz, $CDCl_3$): δ_H 8.26 (bs, 1H), 7.10 – 6.99 (m, 3H), 6.88 – 6.80 (m, 1H), 3.77 (d, *J* = 10.8 Hz, 3H), 2.30 – 2.13 (m, 1H), 1.31 (dd, *J* = 19.4, 7.2 Hz, 6H) ppm.

^{13}C NMR (101 MHz, $CDCl_3$): δ_C 147.8 (d, *J* = 2.6 Hz), 139.2 (d, *J* = 10.1 Hz), 126.5, 121.8 (d, *J* = 4.5 Hz), 120.8, 119.9, 53.8 (d, *J* = 7.9 Hz), 25.4 (d, *J* = 140.1 Hz), 16.0 (d, *J* = 5.1 Hz), 15.9 (d, *J* = 5.1 Hz) ppm.

^{31}P NMR (162 MHz, $CDCl_3$): δ_P39.3 (s) ppm.

IR (film) ν_{max}: 2973, 1595, 1495, 1460, 1371, 1174, 1099, 978, 927, 825, 734 cm^{-1}.

HRMS (ESI$^+$) calcd. for $C_{10}H_{16}O_4P$ [M+H]$^+$ 231.0708, found 231.0781.

2-Hydroxyphenyl methyl cyclobutylphosphonate (**3ba**)

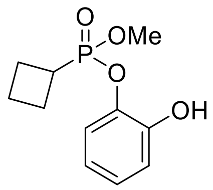

Prepared following **General Procedures E and F**. Purification by flash column chromatography (50% EtOAc/hexane) gave the title compound (44.3 mg, 0.18 mmol, 61% by GP-E) and (35.0 mg, 0.14 mmol, 73% by GP-F) as a colorless oil.

TLC: R_f = 0.30 (50% EtOAc/hexane, $KMnO_4$ stain).

^{1}H NMR (400 MHz, $CDCl_3$): δ_H 8.61 (s, 1H), 7.11 – 6.97 (m, 3H), 6.87 – 6.79 (m, 1H), 3.80 (d, *J* = 10.8 Hz, 3H), 3.01 – 2.89 (m, 1H), 2.55 – 2.35 (m, 2H), 2.35 – 2.03 (m, 4H) ppm.

^{13}C NMR (101 MHz, $CDCl_3$): δ_C 147.8 (d, *J* = 2.7 Hz), 139.0 (d, *J* = 9.5 Hz), 126.6, 121.8 (d, *J* = 4.2 Hz), 120.8, 119.9, 53.7 (d, *J* = 7.6 Hz), 28.8 (d, *J* = 143.4 Hz), 22.6 (d, *J* = 6.7 Hz), 20.4 (d, *J* = 19.4 Hz) ppm.

^{31}P NMR (162 MHz, $CDCl_3$): δ 35.7 (s) ppm.

IR (film) ν_{max}: 3054, 1590, 1496, 1458, 1172, 1098, 980, 938, 825 cm^{-1}.

HRMS (ESI$^+$): calcd. for $C_{11}H_{15}NaO_4P$ $[M+Na]^+$ 265.0600, found 265.0612.

2-Hydroxyphenyl methyl (tetrahydro-2H-pyran-4-yl)phosphonate (**3bb**)

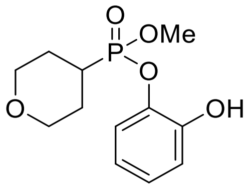

Prepared following **General Procedures E and F**. Purification by flash column chromatography (80% EtOAc/hexane) gave the title compound (57.9 mg, 0.21 mmol, 71%) and (43.0 mg, 0.16 mmol, 80% by GP-F) as a colorless oil.

TLC: R_f = 0.20 (80% EtOAc/hexane, $KMnO_4$ stain).

^{1}H NMR (400 MHz, $CDCl_3$): δ_H 8.42 (bs, 1H), 7.11 – 7.00 (m, 3H), 6.87 – 6.81 (m, 1H), 4.09 – 4.00 (m, 2H), 3.78 (d, *J* = 10.8 Hz, 3H), 3.46 – 3.34 (m, 2H), 2.36 – 2.18 (m, 1H), 1.98 – 1.82 (m, 4H) ppm.

^{13}C NMR (101 MHz, $CDCl_3$): δ_C 147.6 (d, *J* = 2.9 Hz), 139.0 (d, *J* = 10.0 Hz), 126.7, 121.7 (d, *J* = 4.5 Hz), 120.9, 119.9, 67.5 (d, *J* = 1.4 Hz), 67.3 (d, *J* = 1.5 Hz), 53.9 (d, *J* = 7.6 Hz), 32.5 (d, *J* = 146.1 Hz), 25.6 (d, *J* = 2.1 Hz), 25.5 (d, *J* = 2.0 Hz) ppm.

^{31}P NMR (162 MHz, $CDCl_3$) δ 33.6 (s) ppm.

IR (film) ν_{max}: 3057, 1495, 1443, 1422, 1184, 1030, 987, 941, 830, 700 cm^{-1}.

HRMS (ESI^+) calcd. for $C_{12}H_{17}NaO_5P$ $[M+Na]^+$ 295.0706, found 295.0709.

***tert*-Butyl 4-((2-hydroxyphenoxy)(methoxy)phosphoryl)piperidine-1-carboxylate** (**3bc**)

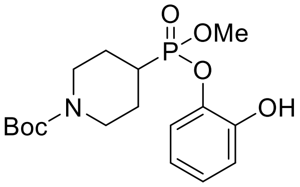

Prepared following **General Procedures E and F**. Purification by flash column chromatography (50% EtOAc/hexane) gave the title compound (74.0 mg, 0.20 mmol, 66%) and (61.0 mg, 0.16 mmol, 82% by GP-F) as a colorless oil.

TLC: R_f = 0.20 (50% EtOAc/hexane, $KMnO_4$ stain).

^{1}H NMR (400 MHz, $CDCl_3$): δ_H 8.46 (s, 1H), 7.10 – 6.99 (m, 3H), 6.86 – 6.81 (m, 1H), 4.32 – 4.12 (m, 2H), 3.76 (d, *J* = 10.8 Hz, 3H), 2.71 (t, *J* = 12.8 Hz, 2H), 2.21 – 2.07 (m, 1H), 2.04 – 1.94 (m, 2H), 1.77 – 1.63 (m, 2H), 1.45 (s, 9H) ppm.

^{13}C NMR (101 MHz, $CDCl_3$): δ_C 154.5, 147.5 (d, *J* = 2.9 Hz), 138.8 (d, *J* = 10.1 Hz), 126.6, 121.6 (d, *J* = 4.5 Hz), 120.8, 119.8, 79.9, 53.8 (d, *J* = 7.6 Hz), 43.5, 33.4 (d, *J* = 145.3 Hz), 28.4, 25.0 ppm.

^{31}P NMR (162 MHz, $CDCl_3$): δ 34.1 (s) ppm.

IR (film) ν_{max}: 2979, 1681(s), 1495, 1425, 1355, 1164, 1100, 907, 829 cm^{-1}.

HRMS (ESI^+): calcd. for $C_{17}H_{26}NNaO_6P$ $[M+Na]^+$ 394.1390, found 394.1384.

5. MECHANISTIC STUDY

5.1. Radical Clock Experiment

First, the radical clock reaction was conducted, the use of **1bd** and **4bd** as radical precursor reacted with **2a** under standard deboronative (general procedure-E) and decarboxylative (general procedure-F) conditions, respectively; over 50% isolated yield of *5-exo-trig* cyclization product **3bd'** was obtained in both the cases. The crude ^{1}H- and ^{31}P-NMR analyses showed that the ratio of cyclized and linear products (**3bd'**:**3bd** 70:30, decarboxylative) and (**3bd'**:**3bd** = 78:22, deboronative).

Note: The major (**3bd'**) and minor (**3bd**) products were isolated and characterized in decarboxylative phosphonylation case (GP-F). Whereas, in deboronative case (GP-E), only the major product (**3bd'**) was isolated.

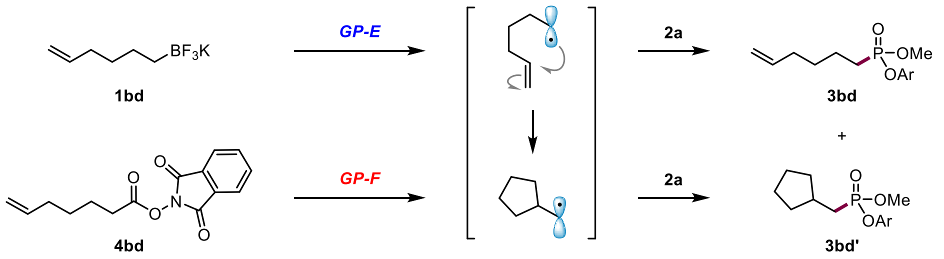

Procedure	3bd:3bd'	yield 3bd'
E	22:78	57%
F	30:70	50%

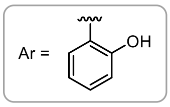

2-Hydroxyphenyl methyl (cyclopentylmethyl)phosphonate (3bd')

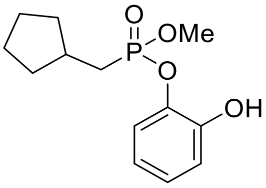

Prepared following **General Procedures E and F**. Purification by flash column chromatography (50% EtOAc/hexane) gave the cyclic compound (46.2 mg, 0.17 mmol, 57% by GP-E) and (40.5 mg, 0.15 mmol, 50% by GP-F) as a yellow oil.

TLC: R_f = 0.2 (80% EtOAc/hexane, $KMnO_4$ stain).

^{1}H NMR (400 MHz, $CDCl_3$): δ_H 7.12 – 6.98 (m, 3H), 6.88 – 6.79 (m, 1H), 3.77 (d, *J* = 11.0 Hz, 3H), 2.30 – 2.15 (m, 1H), 2.08 – 1.90 (m, 4H), 1.72 – 1.62 (m, 2H), 1.62 – 1.49 (m, 2H), 1.31 – 1.18 (m, 2H). ppm.

^{13}C NMR (101 MHz, $CDCl_3$): δ_C 147.9 (d, *J* = 3.0 Hz), 139.0 (d, *J* = 9.8 Hz), 126.6, 121.9 (d, *J* = 4.2 Hz), 120.8, 119.9 (d, *J* = 1.5 Hz), 53.4 (d, *J* = 7.7 Hz), 34.3 (d, *J* = 5.2 Hz), 34.1 (d, *J* = 12.0 Hz), 33.9 (d, *J* = 10.8 Hz), 30.4 (d, *J* = 137.3 Hz), 24.9, 24.8 ppm.

^{31}P NMR (162 MHz, $CDCl_3$): δ 35.9 (s) ppm.

IR (film) ν_{max}: 2954, 1597, 1495, 1453, 1172, 1097, 985, 938, 830, 703 cm^{-1}.

HRMS (ESI$^+$): calcd. for $C_{13}H_{20}O_4P$ $[M+H]^+$ 271.1094, found 271.1098.

2-Hydroxyphenyl methyl hex-5-en-1-ylphosphonate (3bd)

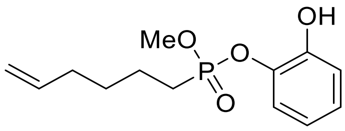

Prepared following **General Procedure F**. Purification by flash column chromatography (50% EtOAc/hexane) gave the cyclic compound (18.6 mg, 0.06 mmol, 23% by GP-F) as a yellow oil.

TLC: R_f = 0.3 (80% EtOAc/hexane, $KMnO_4$ stain).

^{1}H NMR (500 MHz, $CDCl_3$): δ_H 8.55 (bs, 1H), 7.13 – 6.98 (m, 3H), 6.84 (dddd, *J* = 7.9, 7.0, 2.0, 0.8 Hz, 1H), 5.77 (ddt, *J* = 16.9, 10.2, 6.7 Hz, 1H), 5.06 – 4.94 (m, 2H), 3.78 (d, *J* = 11.0 Hz, 3H), 2.12 – 2.04 (m, 2H), 2.02 – 1.92 (m, 2H), 1.78 – 1.64 (m, 2H), 1.57 – 1.46 (m, 2H) ppm.

^{13}C NMR (126 MHz, $CDCl_3$): δ_C 147.9 (d, *J* = 2.9 Hz), 138.9 (d, *J* = 9.5 Hz), 138.0, 126.73 (d, *J* = 1.4 Hz), 121.8 (d, *J* = 4.4 Hz), 120.9 (d, *J* = 1.0 Hz), 119.9 (d, *J* = 1.4 Hz), 115.3, 53.6 (d, *J* = 7.5 Hz), 33.2 (d, *J* = 1.4 Hz), 29.7 (d, *J* = 17.0 Hz), 24.5 (d, *J* = 139.1 Hz), 21.7 (d, *J* = 5.5 Hz) ppm.

^{31}P NMR (162 MHz, $CDCl_3$): δ 36.4 (s) ppm.

IR (film) ν_{max}: 3154, 3076, 2936, 1640, 1593, 1495, 1513, 1459, 1293, 1260, 1223, 1173, 1033, 936, 829, 749 cm^{-1}.

HRMS (ESI^+): calcd. for $C_{13}H_{20}O_4P$ $[M+H]^+$ 271.1094, found 271.1090.

5.2. TEMPO Trapping Experiments

Treatment of **1a**/**4a** and **2a** in the presence of 2,2,6,6-tetramethyl-1-piperidinyloxy (TEMPO) (1.5 equiv.) was investigated under the standard reaction conditions (GP-E and GP-F). The LC-MS and HRESI-MS of the crude reaction mixtures showed that intermediate **11a** occurred in both the reactions (see below). The results therefore further displayed that free-radical intermediate **I** was involved in both the reactions. The mass of the desired product **3a** can only be seen in traces.

(a) Deboronative phosphonylation:

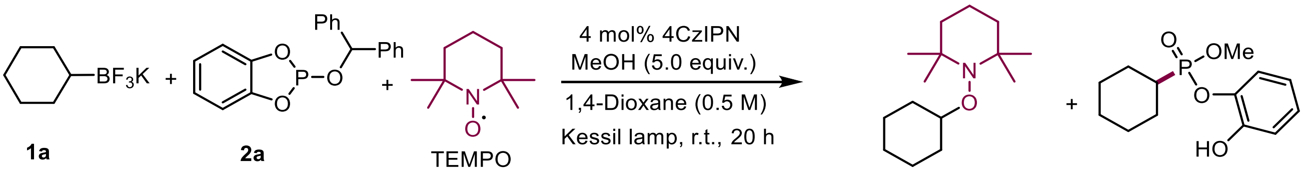

1a **2a** TEMPO

11a, m/z: 240.2 (M+H)$^+$ *detected in LC-MS and HRMS*

3a (traces)

HRMS (ESI$^+$): calcd. for $C_{15}H_{29}NO$ [M+H]$^+$ 240.2322, found 240.2323.

LC chromatogram: The major peak of **11a** is visible at 8.7 min. The mass of the peak at 8.7 min is corresponding to **240.2** [M+H]$^+$

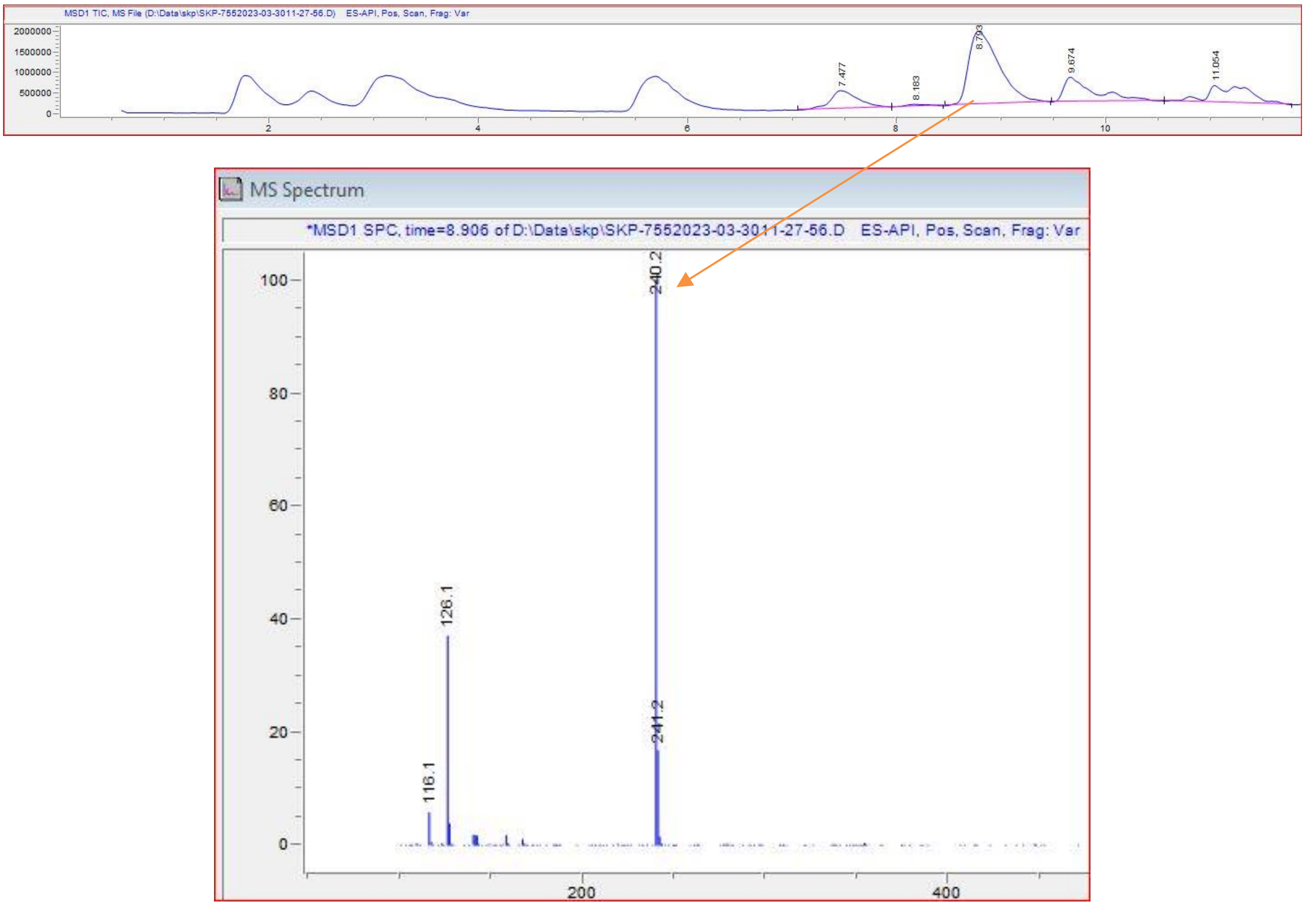

(b) Decarboxylative phosphonylation:

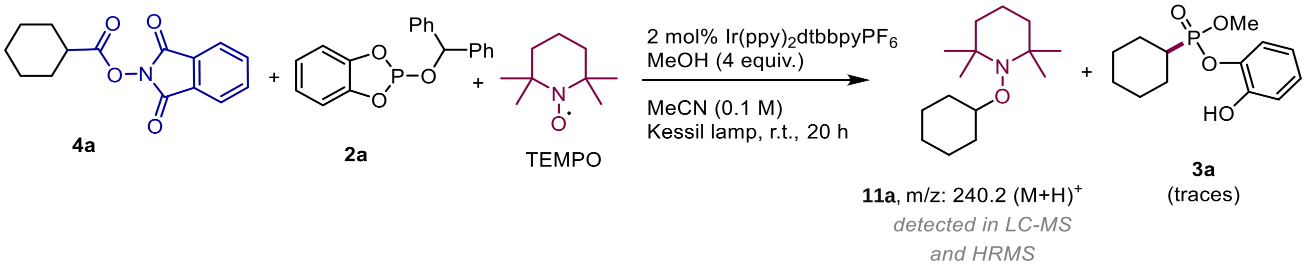

HRMS (ESI+): calcd. for $C_{15}H_{29}NO$ $[M+H]^+$ 240.2322, found 240.2324.

LC chromatogram: The major peak of **11a** is visible at 8.7 min. The mass of the peak at 8.7 min is corresponding to 240.2 $[M+H]^+$

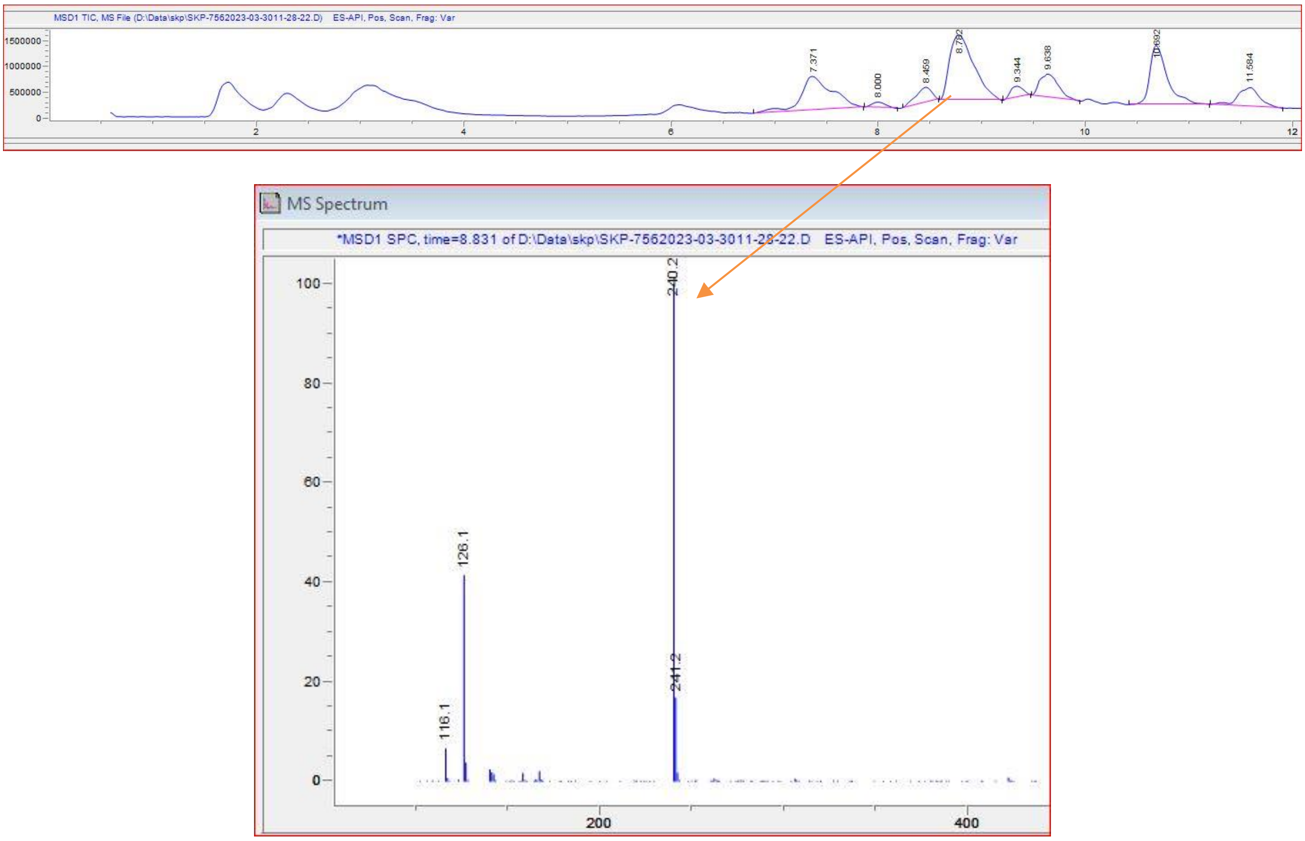

5.3. Confirmation of the Side Products

Diphenylmethane (5)

Byproduct **5** was confirmed by LC-MS as well as by HR-MS analysis upon submitting a crude sample for mass analysis.

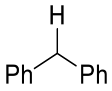

HRMS (ESI$^+$) calcd. for $C_{13}H_{12}$ $[M-H]^-$ 167.2390, found 167.0851.

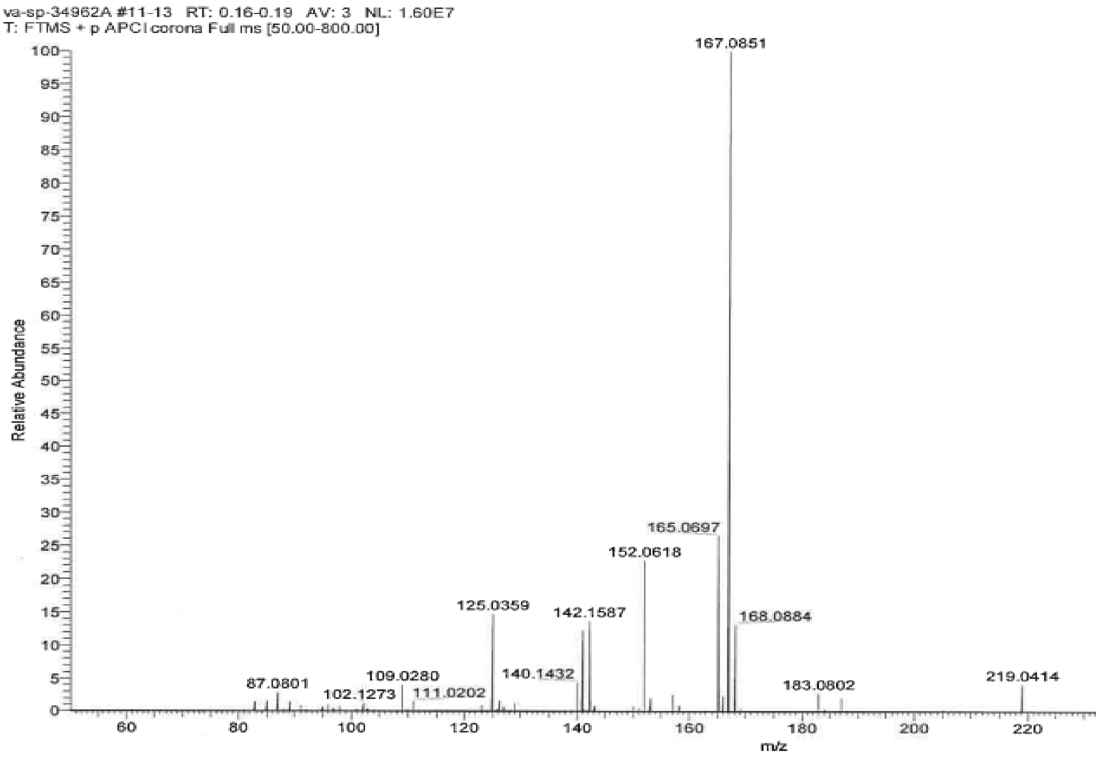

(Methoxymethylene)dibenzene (6)

This product was purified from the reaction mixture and characterized while isolating the phaclofen derivative **3ah**.

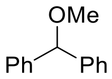

TLC: R_f = 0.4 (10% EtOAc/hexane, $KMnO_4$ stain).

^{1}H NMR (400 MHz, $CDCl_3$): δ_H δ 7.35 – 7.15 (m, 10H), 5.19 (s, 1H), 3.32 (s, 3H) ppm.

^{13}C NMR (101 MHz, $CDCl_3$): δ_C 142.2, 128.5, 127.5, 127.0, 85.5, 57.1 ppm.

The obtained data is in accordance with the literature data.[15]

2-Hydroxyphenyl dimethyl phosphate (12)

This product was observed in variable amounts as a colorless oil, when a large excess of MeOH (>5 equiv.) and/or excess **2a** (>2.0 equiv.) was used under the standard conditions.

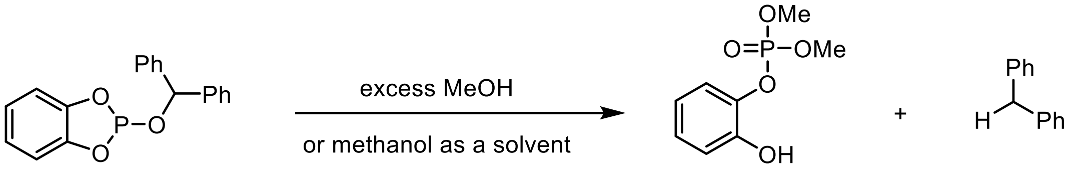

2a **12** (isolated as a major product) **5**, m/z: 167.08 (M-1)$^-$ *detected in LC-MS and HR-MS*

2-Hydroxyphenyl dimethyl phosphate (12)

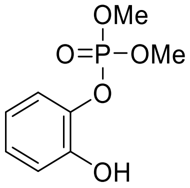

TLC: R_f = 0.33 (50% EtOAc/hexane, $KMnO_4$ stain).

^{1}H NMR (400 MHz, $CDCl_3$): δ_H 7.13 – 7.07 (m, 2H), 7.05 – 7.02 (m, 1H), 6.86 (tdd, *J* = 7.7, 1.8, 0.8 Hz, 1H), 3.91 (s, 3H), 3.88 (s, 2H) ppm.

^{13}C NMR (101 MHz, $CDCl_3$): δ_C 147.6 (d, *J* = 3.7 Hz), 138.47 (d, *J* = 7.8 Hz), 126.95 (d, *J* = 1.8 Hz), 121.48 (d, *J* = 4.4 Hz), 121.04 (d, *J* = 1.3 Hz), 119.52 (d, *J* = 1.6 Hz), 55.78, 55.72 ppm.

^{31}P NMR (162 MHz, $CDCl_3$): δ –1.15 (s) ppm.

IR (film) ν_{max}: 3192, 2959, 1595, 1513, 1459, 1497, 1260, 1178, 1236, 1178, 1035, 956, 836, 752, 556 cm^{-1}.

HRMS (ESI$^+$): calcd. for $C_8H_{12}O_5P$ [M+H]$^+$ 218.0937, found 218.0944.

6. PRODUCT TRANSFORMATIONS

6.1. Gram Scale Conditions

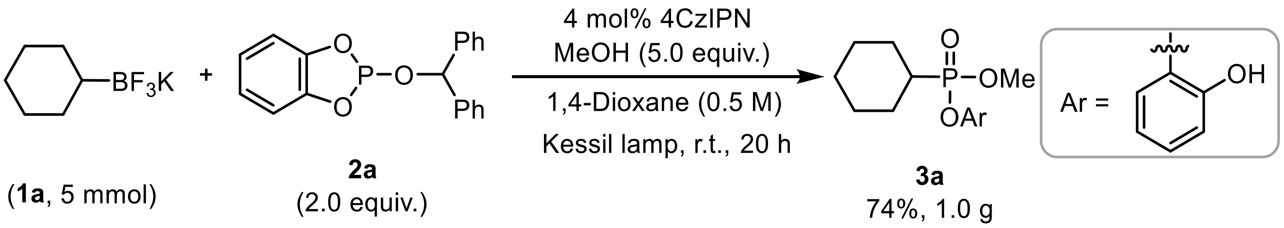

[a] Deboronative Phosphonylation: Prepared following modified **General Procedure E** with **1a** (5.00 mmol) and 4CzIPN (4 mol%) in 1,4-dioxane (0.5 M). Purification by flash column chromatography (50% EtOAc/hexane) gave the **3a** compound (1.0 g, 3.70 mmol, 74%) as a yellow oil.

[b] Decarboxylative Phosphonylation: Prepared following modified **General Procedure F** with **4ah** (1.0 g, 2.2 mmol) and $[Ir(ppy)_2(dtbbpy)]PF_6$ (0.5 mol%) in CH_3CN:THF (12:3, v/v). Purification by flash column chromatography (80% EtOAc/hexane) gave the **3ah** compound (0.62 g, 1.35 mmol, 62%) as a colorless oil.

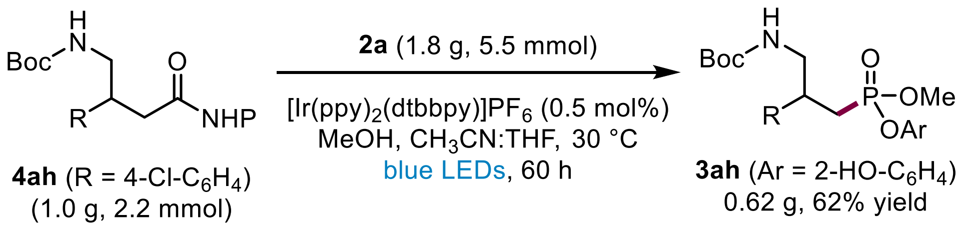

6.2. Representative Transformations of 3a

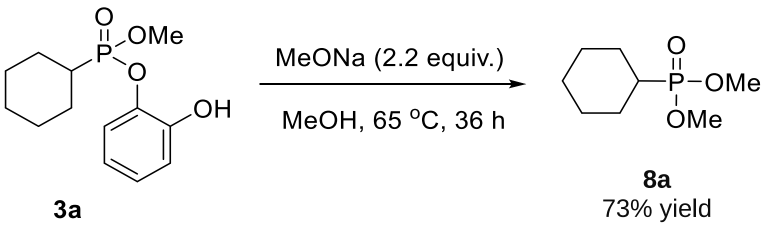

To the solution of **3a** (162.0 mg, 0.6 mmol, 1.0 equiv.) in dry MeOH (0.05 M) was added MeONa (2.2 equiv.). The reaction mixture was stirred vigorously for 36 h at 65 ˚C. NH_4Cl (aq) and EtOAc was then added and allowed to stir for 10 minutes. The organic layer was washed with saturated aqueous bicarbonate, brine, dried over $MgSO_4$, and concentrated. Purification by flash column chromatography (80% EtOAc/hexane) gave **8a** (84.2 mg, 0.44 mmol, 73%) as a yellow oil. All recorded spectroscopic data matched those previously reported in the literature.[12]

Dimethyl cyclohexylphosphonate (**8a**)

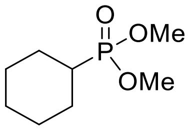

^{1}H NMR (400 MHz, $CDCl_3$): δ_H 3.69 (d, *J* = 10.5 Hz, 6H), 1.95 – 1.85 (m, 2H), 1.80 – 1.68 (m, 3H), 1.68 – 1.62 (m, 1H), 1.41 – 1.27 (m, 2H), 1.25 – 1.11 (m, 3H) ppm.

^{13}C NMR (101 MHz, $CDCl_3$): δ_C 52.4 (d, *J* = 6.9 Hz), 35.3 (d, *J* = 142.2 Hz), 26.1 (d, *J* = 16.3 Hz), 25.8 (d, *J* = 5.0 Hz), 25.7 ppm.

The obtained data is in accordance with the literature data.[16]

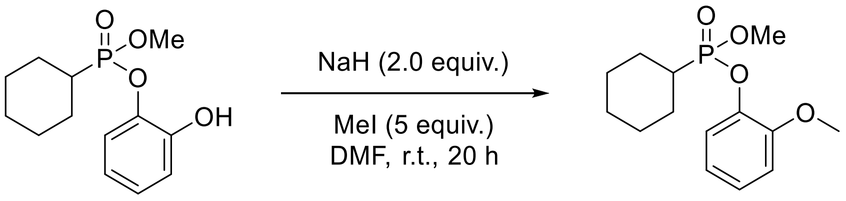

3a **9a** 91% yield

2-Methoxyphenyl methyl cyclohexylphosphonate (9a)

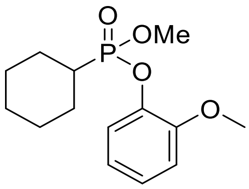

To the solution of **3a** (270.3 mg, 1.0 mmol, 1.0 equiv.) in dry DMF (0.05 M) was added NaH (2.0 equiv.) at 0 ˚C under N_2. The reaction mixture was stirred vigorously for 1 hour at room temperature, then MeI (5.0 equiv.) was added and stirred for further 20 h. NH_4Cl (aq) and EtOAc was then added and allowed to stir for 10 minutes. The organic layer was washed with saturated aqueous bicarbonate, brine, dried over $MgSO_4$, and concentrated. Purification by flash column chromatography (80% EtOAc/hexane) gave the **9a** (258.7 mg, 0.91 mmol, 91%) as a yellow oil.

TLC: R_f = 0.3 (50% EtOAc/hexane, $KMnO_4$ stain).

^{1}H NMR (400 MHz, $CDCl_3$): δ_H 7.24 (dt, J = 8.0, 1.5 Hz, 1H), 7.12 – 7.03 (m, 1H), 6.95 – 6.83 (m, 2H), 3.84 (s, 3H), 3.78 (d, J = 10.9 Hz, 3H), 2.14 – 2.03 (m, 2H), 2.04 – 1.87 (m, 1H), 1.88 – 1.76 (m, 2H), 1.74 – 1.65 (m, 1H), 1.58 – 1.40 (m, 2H), 1.33 – 1.15 (m, 3H) ppm.

^{13}C NMR (101 MHz, $CDCl_3$): δ_C 150.9 (d, J = 4.2 Hz), 140.0 (d, J = 9.1 Hz), 125.4 (d, J = 1.5 Hz), 122.0 (d, J = 2.9 Hz), 120.9 (d, J = 1.5 Hz), 112.7, 55.9, 52.6 (d, J = 7.6 Hz), 36.1 (d, J = 142.3 Hz), 26.2 (d, J = 3.3 Hz), 26.1 (d, J = 3.3 Hz), 25.88 (d, J = 4.5 Hz), 25.86 (d, J = 1.7 Hz), 25.8 ppm.

^{31}P NMR (162 MHz, $CDCl_3$): δ 31.5 (s) ppm.

IR (film) ν_{max}: 2935, 1500, 1502, 1454, 1204, 1174, 1040, 918, 804, 790 cm^{-1}.

HRMS (ESI$^+$): calcd. for $C_{14}H_{22}O_4P$ $[M+H]^+$ 285.1250, found 285.1264.

6.3. Hydrolysis with HCl

According to the literature known procedure,[17] a mixture of **3ah** (615 mg, 1.35 mmol) in 36% aq. HCl (8 mL) and MeOH (1 mL) was refluxed (110 °C) for a period of 11 h. The mixture allowed to cool, and co-evaporated several times with water. The crude residue is dissolved in water (25 mL), washed with ether (25 mL x 4) and the aqueous extract evaporated to give **10** as a sticky solid**.** Yield: 382 mg, 99%.

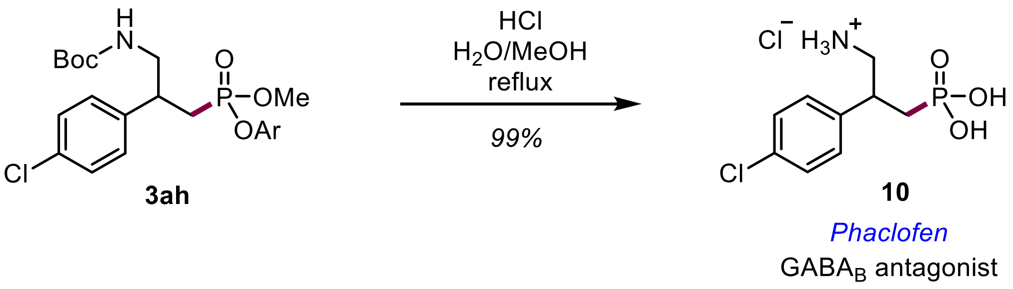

2-(4-Chlorophenyl)-3-phosphonopropan-1-aminium chloride (10)

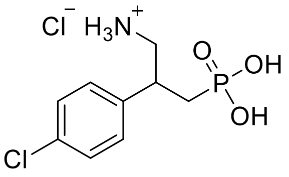

^{1}H NMR (400 MHz, D_2O): δ_H δ 7.35 – 7.26 (m, 2H), 7.24 (q, *J* = 3.0 Hz, 2H), 4.64 (bs, mixture of NH/OH peaks), 3.32 (dd, *J* = 11.7, 3.8 Hz, 1H), 3.21 – 3.07 (m, 2H), 2.07 (dd, *J* = 18.4, 6.6 Hz, 2H) ppm.

^{13}C NMR (101 MHz, D_2O): δ_C 139.1 (d, *J* = 7.5 Hz), 129.3, 128.2, 127.9, 45.0 (d, *J* = 16.3 Hz), 38.8 (d, *J* = 2.9 Hz), 31.5 (d, *J* = 136.0 Hz) ppm.

^{31}P NMR (162 MHz, D_2O): δ_P 25.61 (s) ppm.

The obtained data is in accordance with the literature data.[17]

7. REFERENCES

[1] Luo, J.; Zhang, J. *ACS Catal.* **2016**, *6*, 873.

[2] (a) Ding, S.; Xu, L.; Li, P. *ACS Catal.* **2016**, *6*, 1329. (b) Huang, H.; Zhang, G.; Gong, L.; Zhang, S.; Chen, Y. *J. Am. Chem. Soc.* **2014**, *136*, 2280.

[3] Huang, H.; Zhang, G.; Gong, L.; Zhang, S.; Chen, Y. *J. Am. Chem. Soc.* **2014**, *136*, 2280.

[4] Molander, G. A.; Yun, C.-S.; Ribagorda, M.; Biolatto, B. *J. Org. Chem.* **2003**, *68*, 5534.

[5] Burke, S. J.; Gamrat, J. M.; Santhouse, J. R.; Tomares, D. T.; Tomsho, J. W. *Tetrahedron Lett.* **2015**, *56*, 5500.

[6] Molander, G. A.; Vargas, F. *Org. Lett.* **2007**, *9*, 203.

[7] (a) Ding, S.; Xu, L.; Li, P. *ACS Catal.* **2016**, *6*, 1329. (b) Huang, H.; Zhang, G.; Gong, L.; Zhang, S.; Chen, Y. *J. Am. Chem. Soc.* **2014**, *136*, 2280.

[8] Molander, G. A.; Ham, J. *Org. Lett.* **2006**, *8*, 2031.

[9] Presset, M.; Fleury-Brégot, N.; Oehlrich, D.; Rombouts, F.; Molander, G. A. *J. Org. Chem.* **2013**, *78*, 4615.

[10] Sorin, G.; Malloquin, R. M.; Contie, Y.; Baralle, A.; Malacria, M.; Goddard, J.-P.; Fensterbank, L. *Angew. Chem., Int. Ed.* **2010**, *49*, 8721.

[11] The synthesis of redox active esters (RAEs) and their characterization, see: (a) A. Fawcett, J. Pradeilles, Y. Wang, T. Mutsuga, E. L. Myers and V. K. Aggarwal, *Science* **2017**, 357, 283-286. (b) C. Li, J. Wang, L. M. Barton, S. Yu, M. Tian, D. S. Peters, M. Kumar, A. W. Yu, K. A. Johnson, A. K. Chatterjee, M. Yan, P. S. Baran. *Science* **2017***, 356,* eaam7355.

[12] (a) Artyushin, O.; Odinets, I.; Goryunov, E.; Fedyanin, I.; Lyssenko, K.; Mastryukova, T.; Röschenthaler, G.-V.; Kégl, T.; Keglevich, G.; Kollár, L. *J. Organomet. Chem.* **2006**, *691*, 5547. (b) Ould, D. M. C.; Tran, T. T. P.; Rawson, J. M.; Melen, R. L. *Dalton Trans.* **2019**, *48*, 16922.

[13] Geant, P.-Y.; Mohamed, B. S.; Perigaud, C.; Peyrottes, S.; Uttaro, J.-P.; Mathe, C. *New J. Chem.* **2016**, *40*, 5318.

[14] Tang, Caiming & Tan, Jianhua. (2017). Observation of Concerted and Stepwise Multiple Dechlorination Reactions of Perchlorethylene in Electron Ionization Mass Spectrometry According to Measured Chlorine Isotope Effects. https://arxiv.org/ftp/arxiv/papers/1709/1709.01739.pdf

[15] W. Muramatsu, K. Nakano, *Org. Lett.* **2014**, *16*, 2042.

[16] (a) P.-Y. Geant, B. S. Mohamed, C. Périgaud, S. Peyrottes, J.-P. Uttaro, C. Mathé, *New Journal of Chemistry.* **2016**, *00*, 1. (b) Y. Wang, Z. Chang, Y. Hu, X. Lin, X. Dou *Org. Lett.* **2021**, *23*, 1910.

[17] (a) R. G. Hall, Synthesis **1989**, *21*, 442. (b) R. Błaszczyk, T. Gajda, *Tetrahedron Lett.* **2007** *48*, 5859–5863 (c) J. Chiefari, S. Galanopoulos, W. Janowski, D. Kerr and R. Prager, *Aust. J. Chem*., **1987**, *40*, 1511. (d) Z- C. Duan, X- P. Hu, C. Zhang, Z. Zheng, *J. Org. Chem.* **2010**, *75*, 8319.

8. X-RAY CRYSTALLOGRAPHIC ANALYSIS

3l (CCDC number: 2216343)

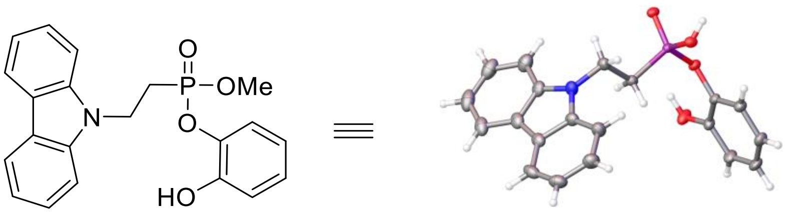

Crystal structure of **3l** (refined by Olex2)

X-ray diffraction experiments on **3l** were carried out at 100(2) K on a Bruker D8 Venture diffractometer. Data collections were performed using a Bruker CPAD detector. The structure was solved using using Olex2. The crystal structure and refinement data are given in the Table below. Crystallographic data for compound **3l** has been deposited with the Cambridge Crystallographic Data Centre as supplementary publications CCDC 2216343. Copies of the data can be obtained free of charge on application to CCDC, 12 Union Road, Cambridge CB2 1EZ, UK [fax(+44) 1223 336033, e-mail: deposit@ccdc.cam.ac.uk].

Bond precision: C-C = 0.0022 A Wavelength=1.54178

Cell:	a=11.4305(4)	b=6.4194(2)	c=24.3820(7)
	alpha=90	beta=100.093(1)	gamma=90
Temperature:	100 K		

	Calculated	Reported
Volume	1761.39(10)	1761.39(10)
Space group	P 21/n	P 1 21/n 1
Hall group	-P 2yn	-P 2yn
Moiety formula	C20 H18 N O4 P	C20 H18 N O4 P
Sum formula	C20 H18 N O4 P	C20 H18 N O4 P
Mr	367.32	367.32
Dx,g cm-3	1.385	1.385
Z	4	4
Mu (mm-1)	1.608	1.608
F000	768.0	768.0
F000'	771.45	
h,k,lmax	13,7,29	13,7,29
Nref	3333	3333
Tmin,Tmax	0.496,0.953	0.322,0.754
Tmin'	0.450	

Correction method= # Reported T Limits: Tmin=0.322 Tmax=0.754
AbsCorr = MULTI-SCAN

Data completeness= 1.000 Theta(max)= 70.031

R(reflections)= 0.0326(3140) wR2(reflections)= 0.0884(3333)

S = 1.044 Npar= 243

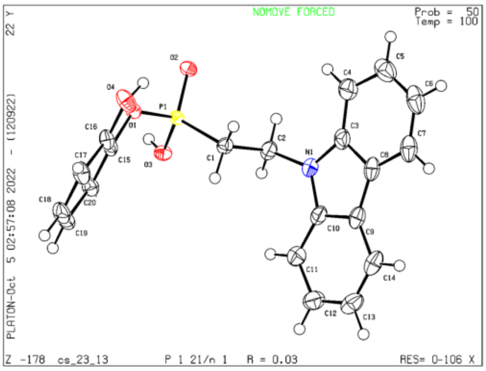

9. NMR DATA

^{1}**H NMR** (400 MHz, acetone-d_6): **1g**

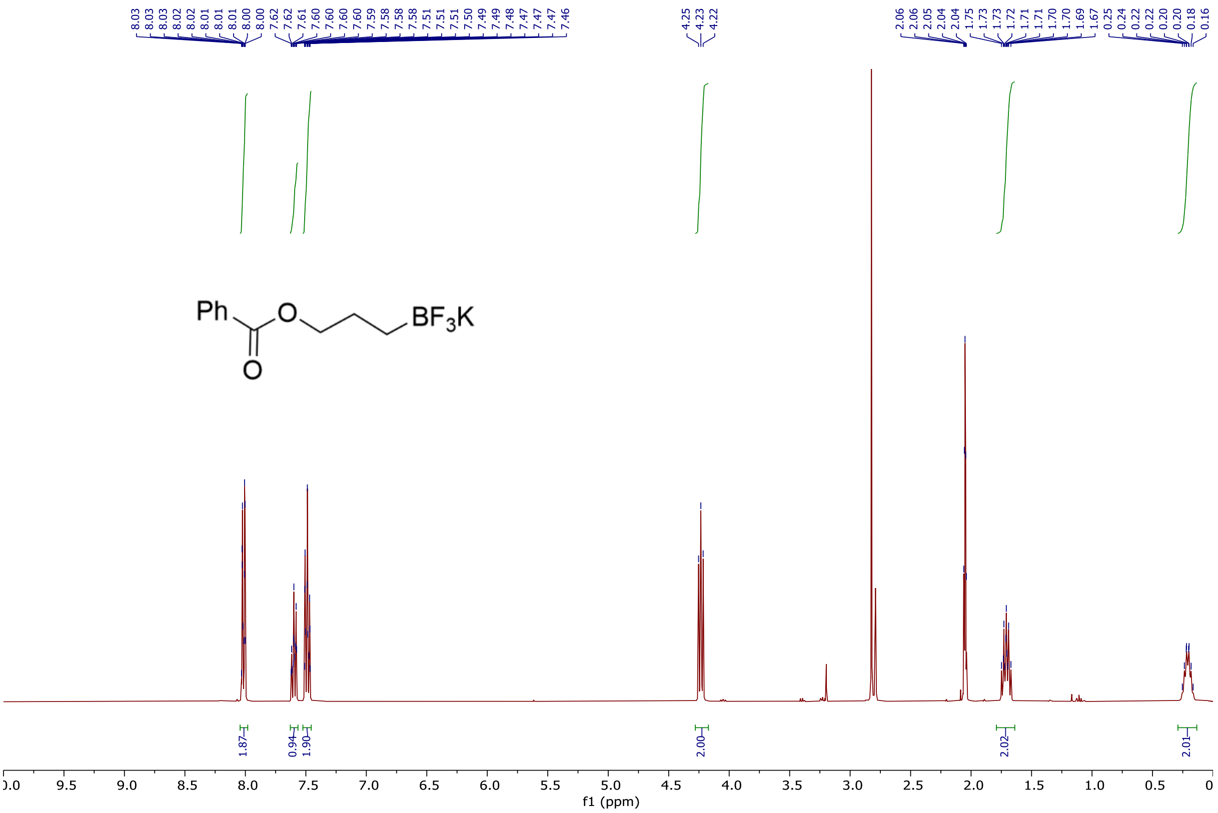

^{13}**C NMR** (101 MHz, acetone-d_6): **1g**

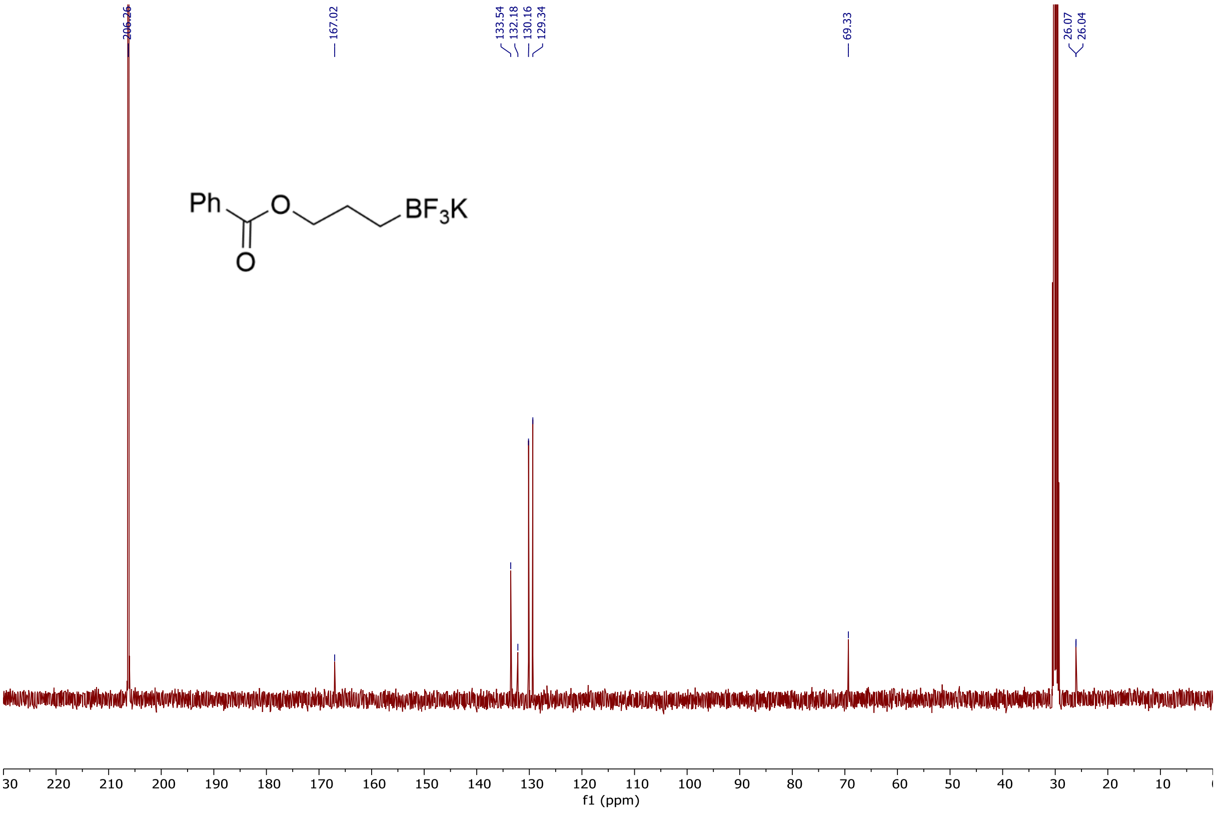

^{1}H NMR (400 MHz, dmso-d_6): **1h**

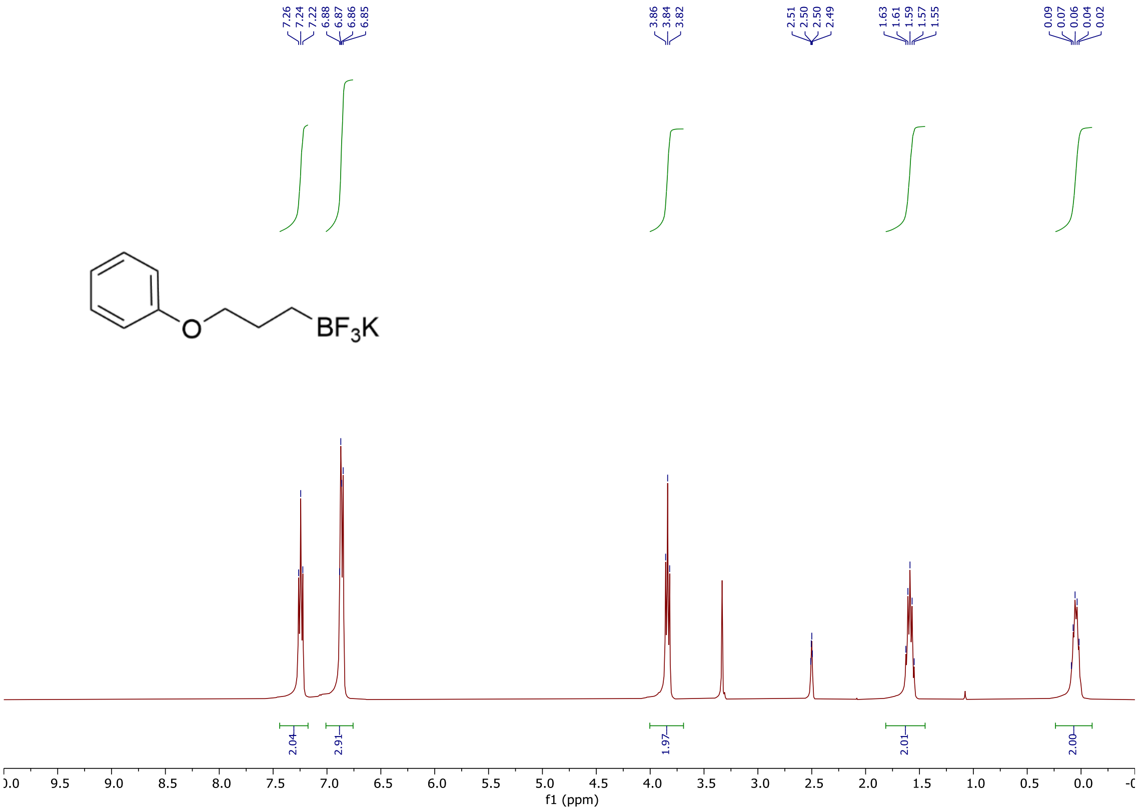

^{13}C NMR (101 MHz, dmso-d_6): **1h**

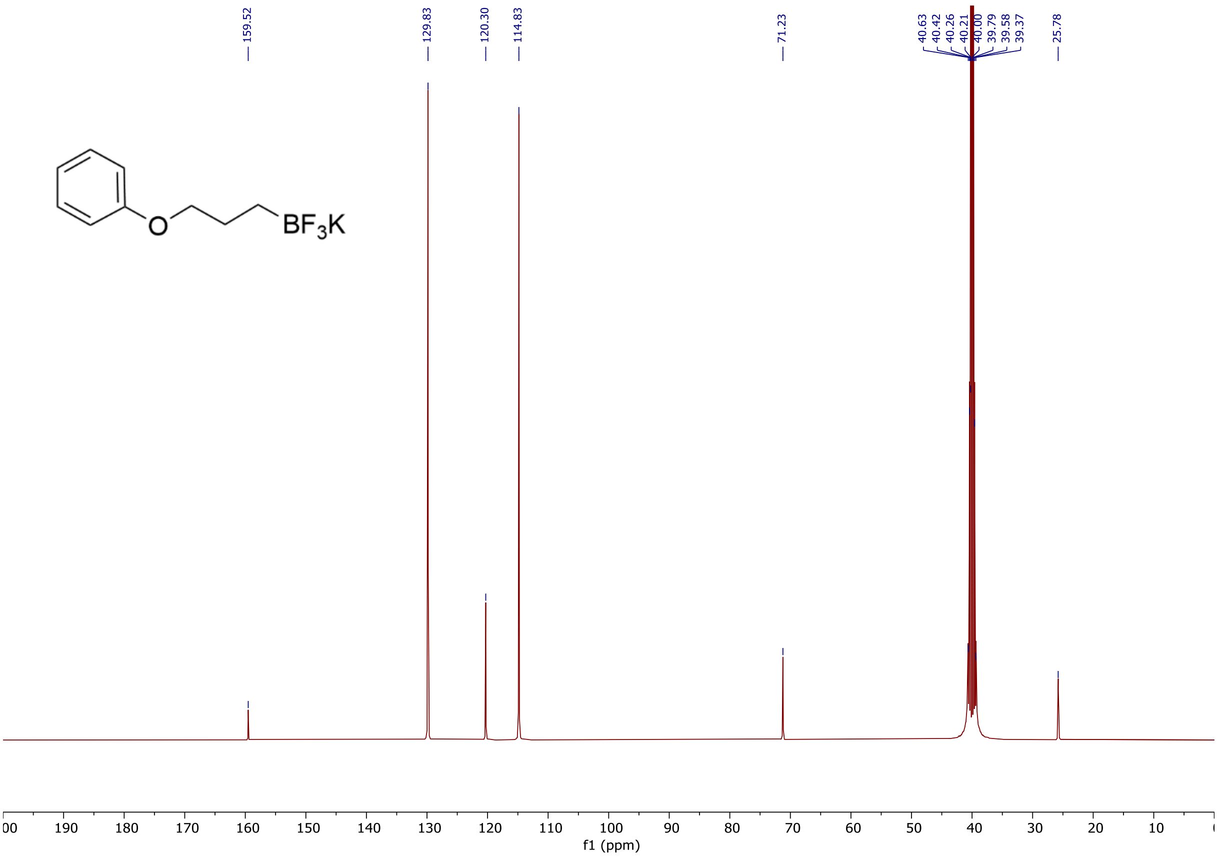

^{19}F NMR (377 MHz, dmso-d_6): **1h**

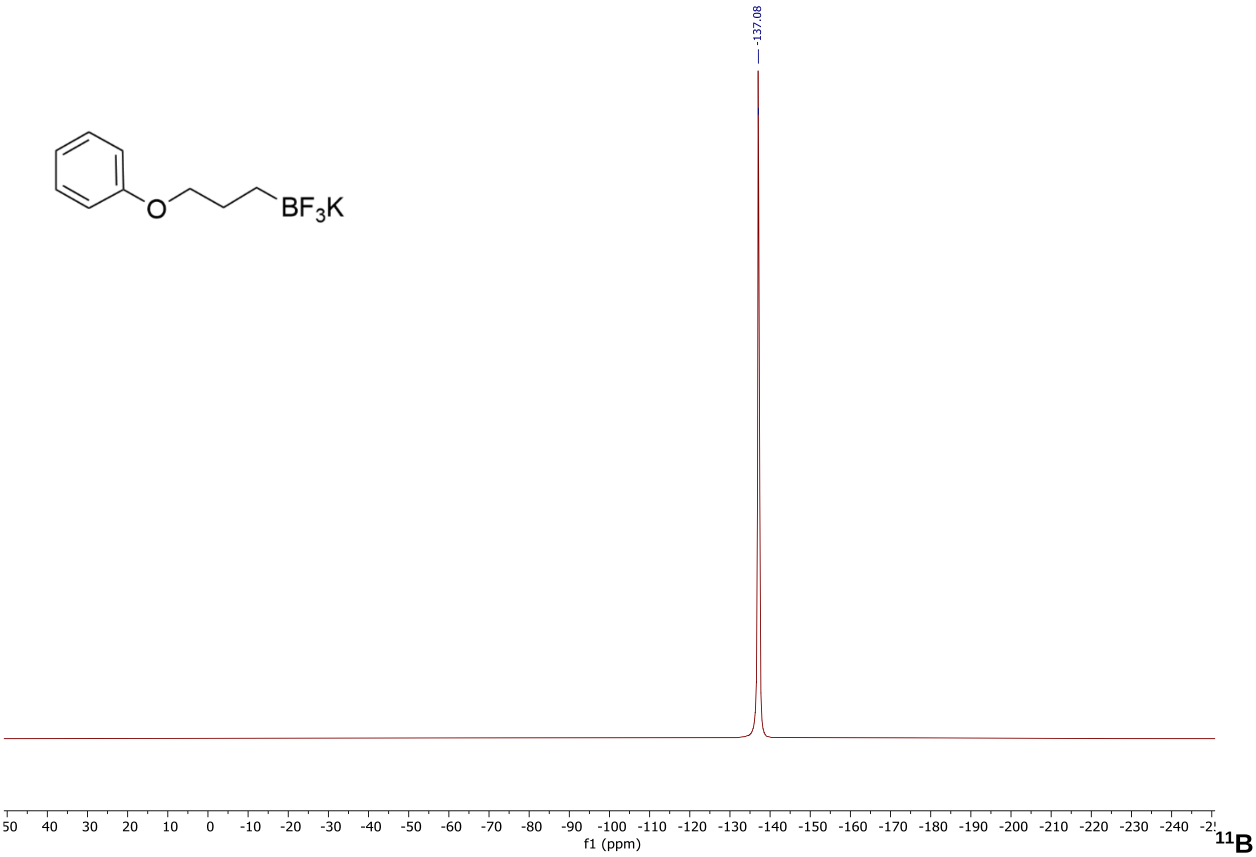

^{11}B NMR (128 MHz, dmso-d_6): **1h**

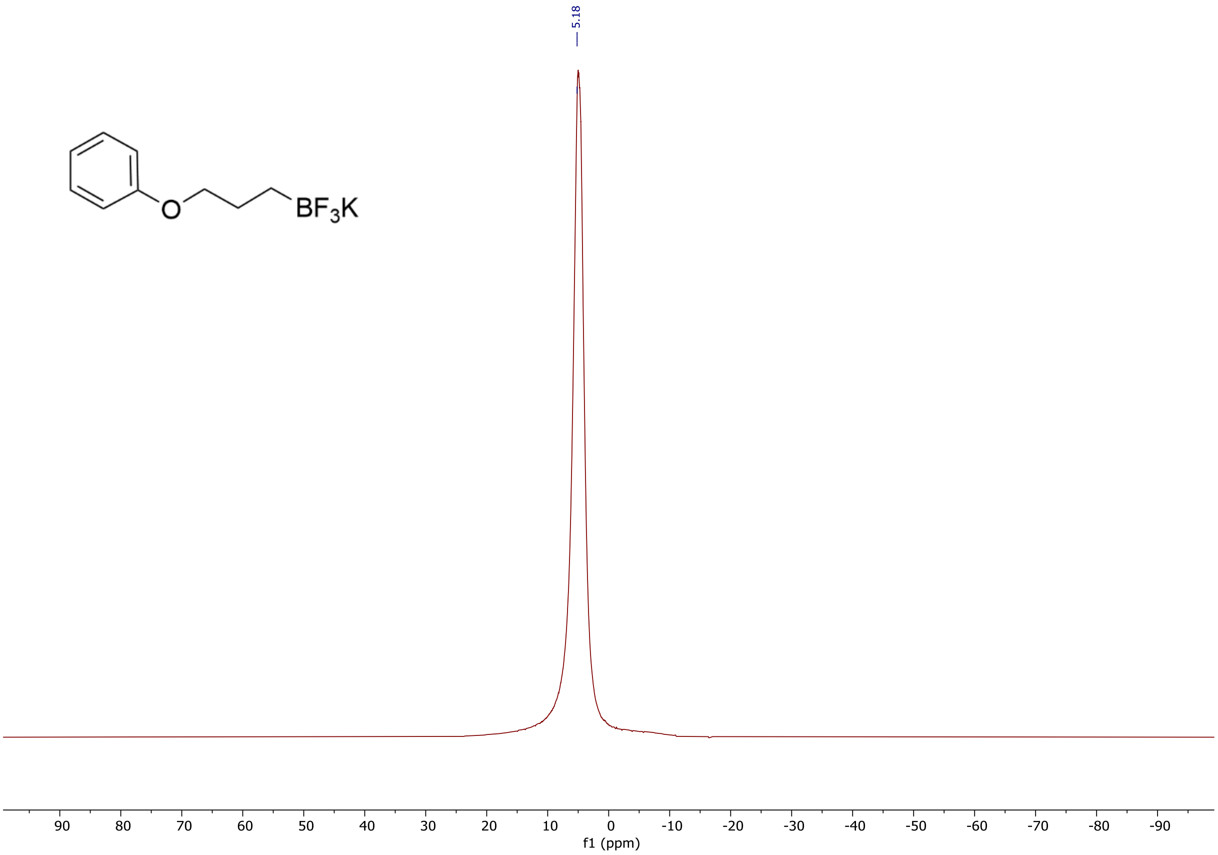

^{1}H NMR (400 MHz, DMSO-*d*6): **1j**

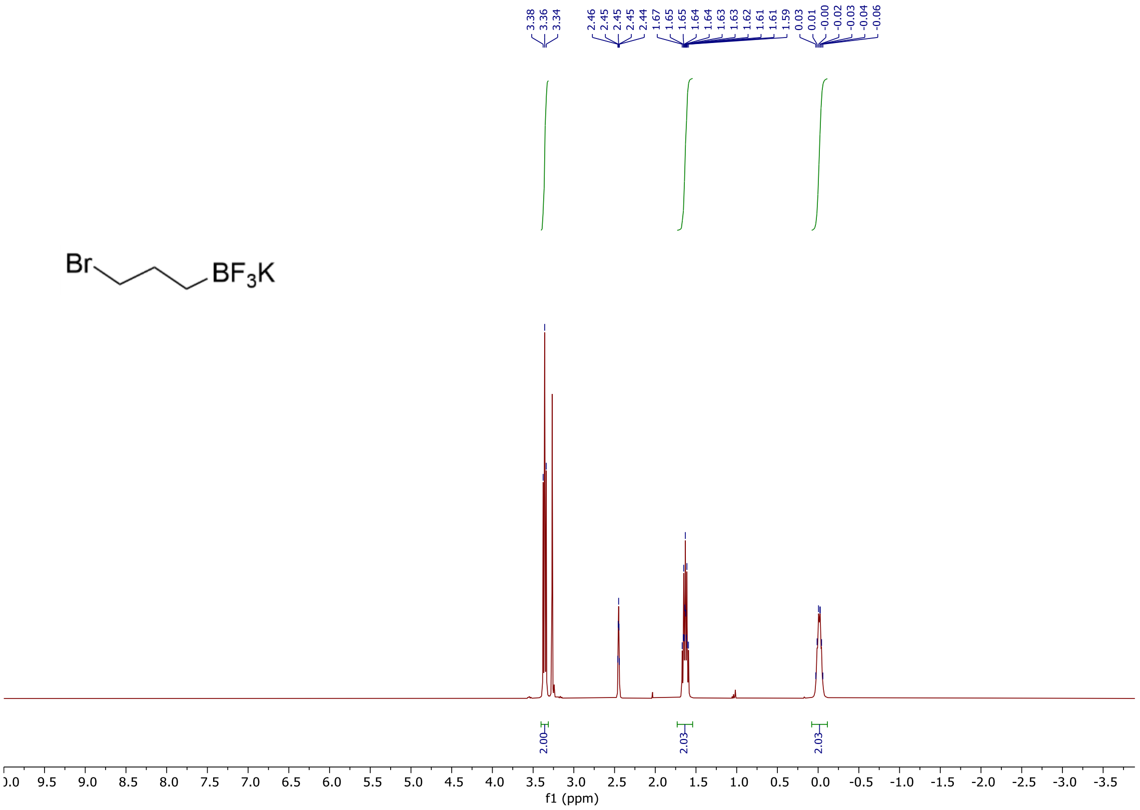

^{13}C NMR (101 MHz, acetone-*d*6): **1j**

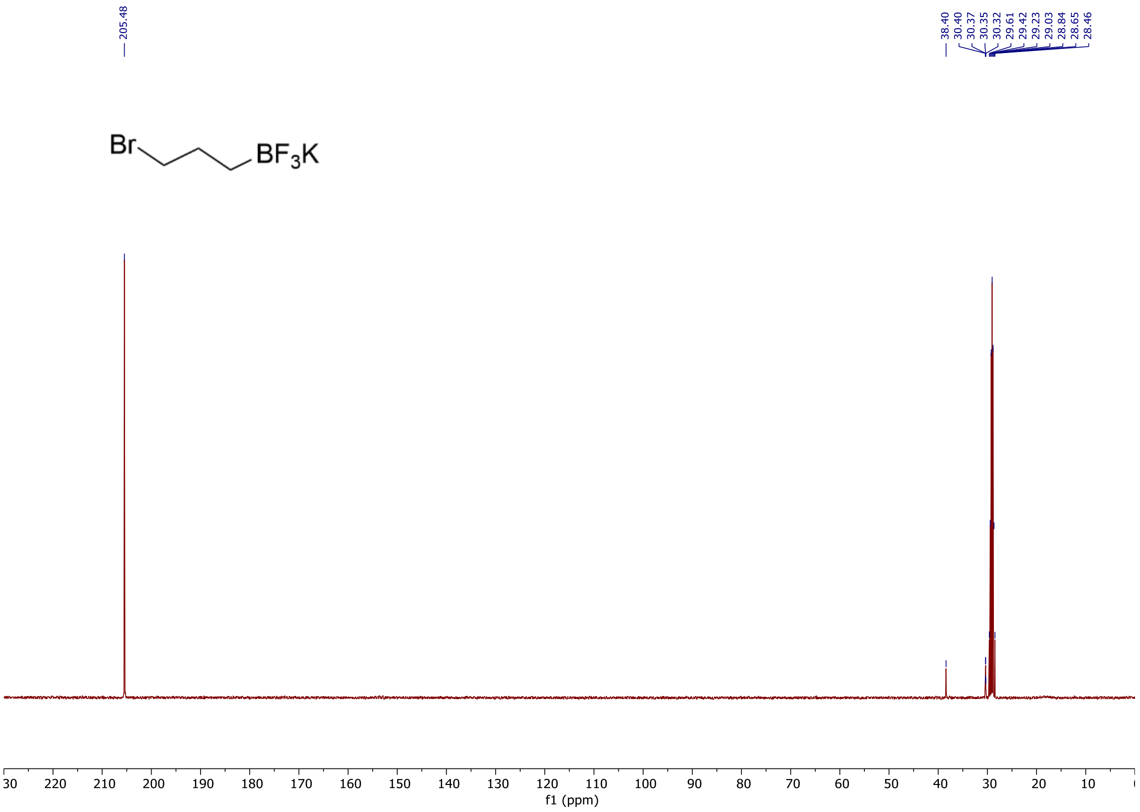

^{1}H NMR (400 MHz, acetone-d_6): **1k**

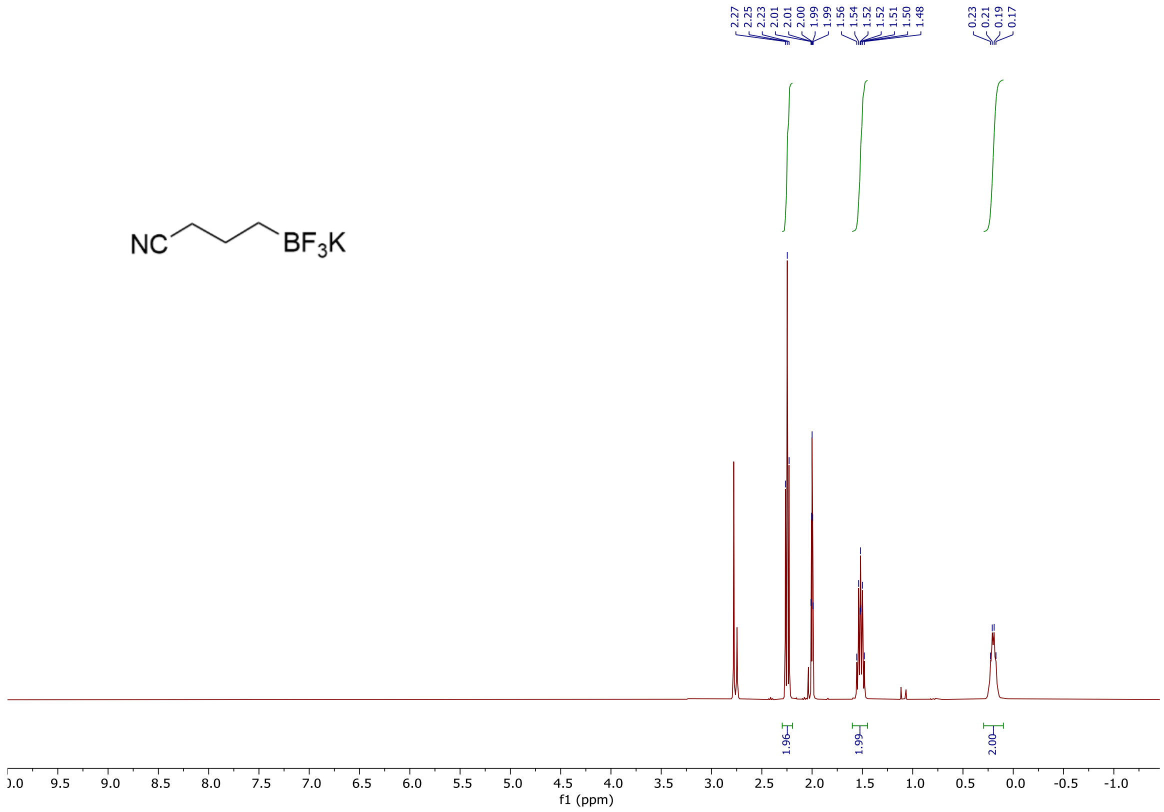

^{13}C NMR (101 MHz, acetone-d_6): **1k**

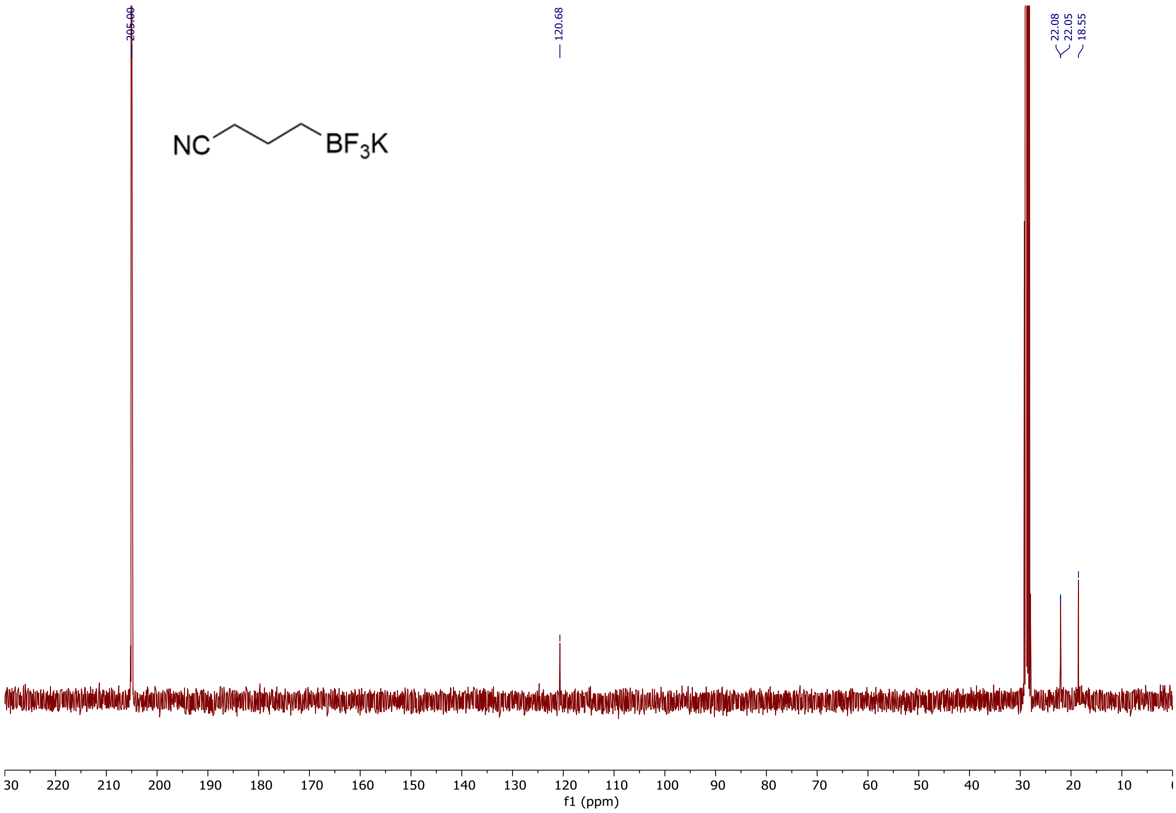

^{19}F NMR (377 MHz, acetone-d_6): **1k**

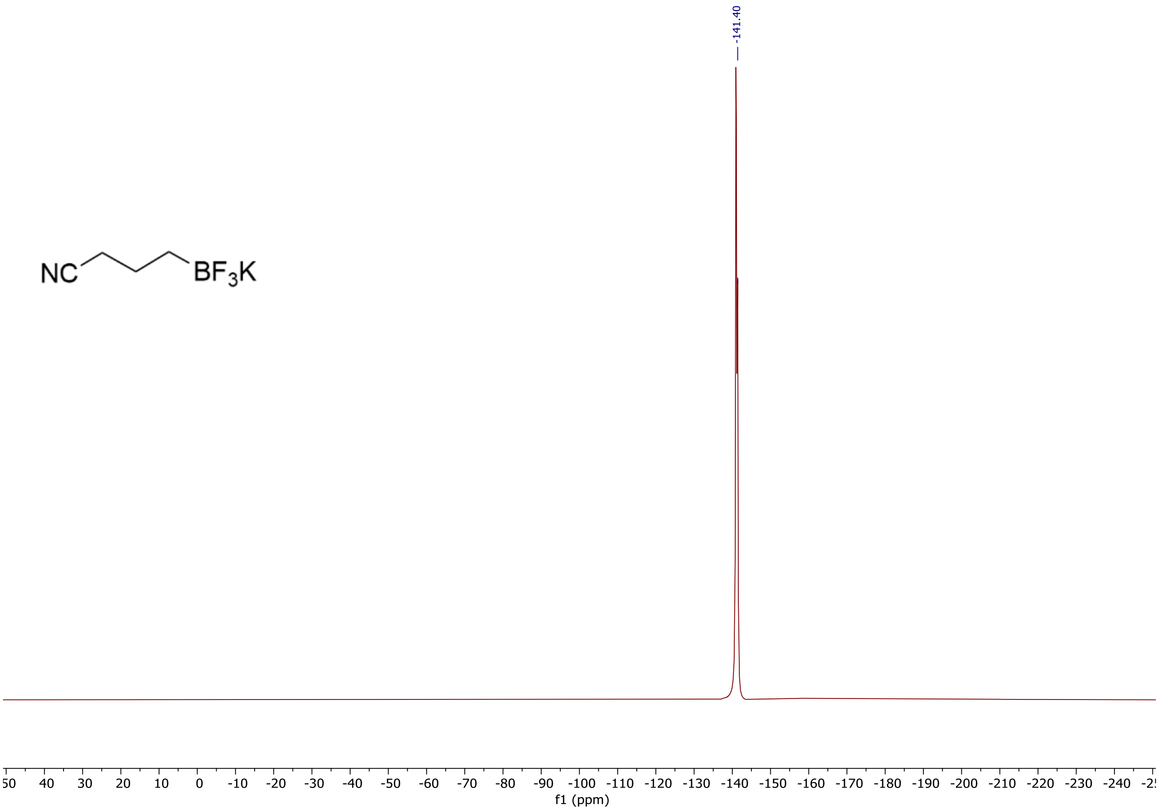

^{11}B NMR (128 MHz, acetone-d_6): **1k**

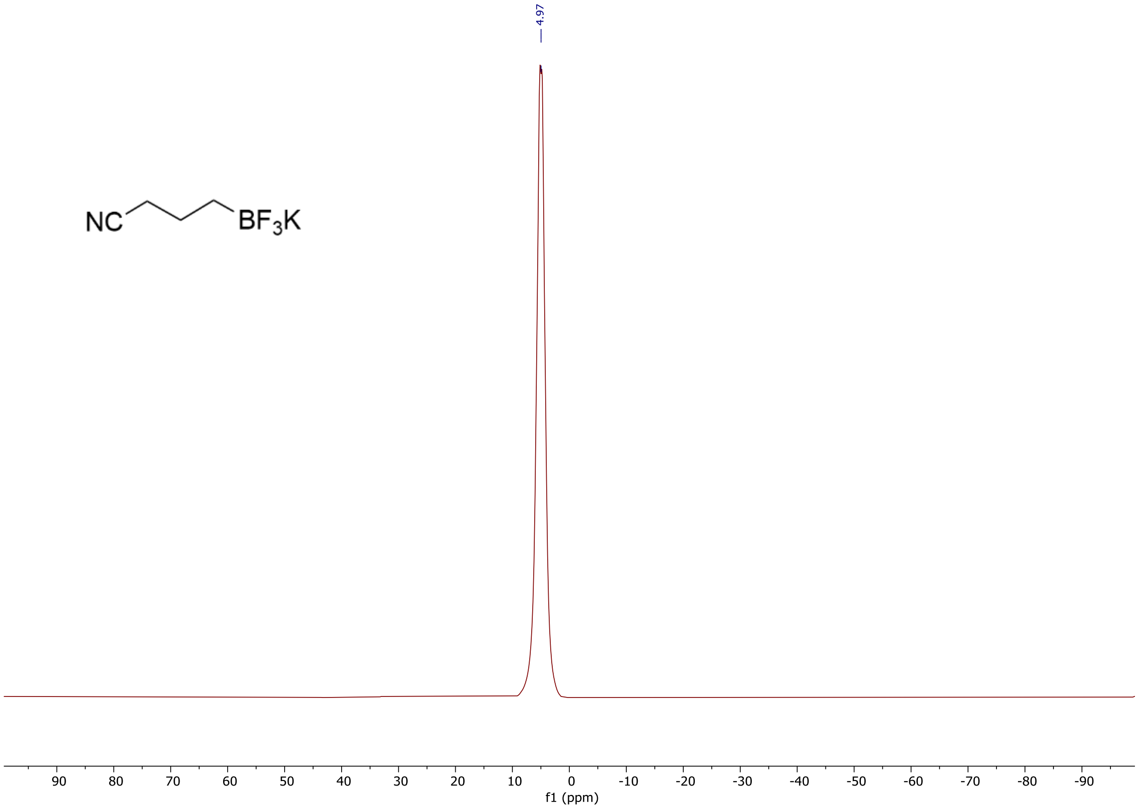

^{1}H NMR (400 MHz, acetone-d_6): **1l**

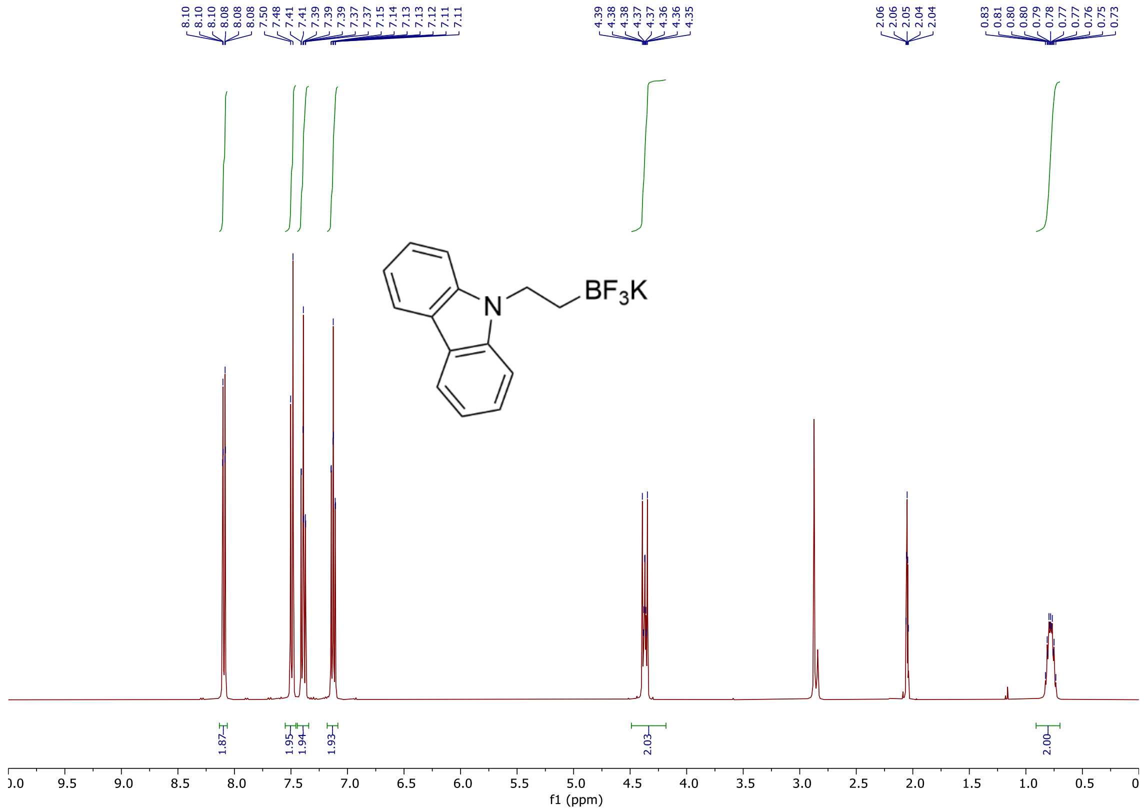

^{13}C NMR (101 MHz, acetone-d_6): **1l**

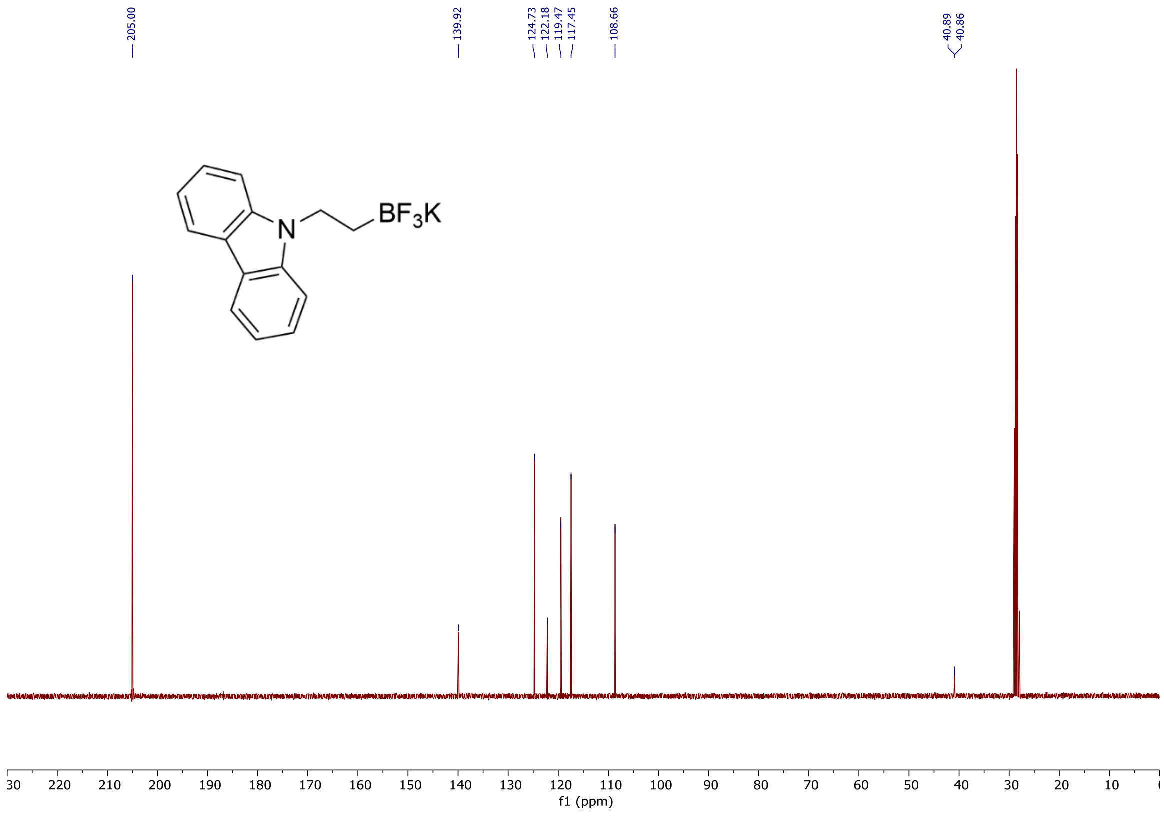

¹H NMR (400 MHz, dmso-*d*6): **1o**

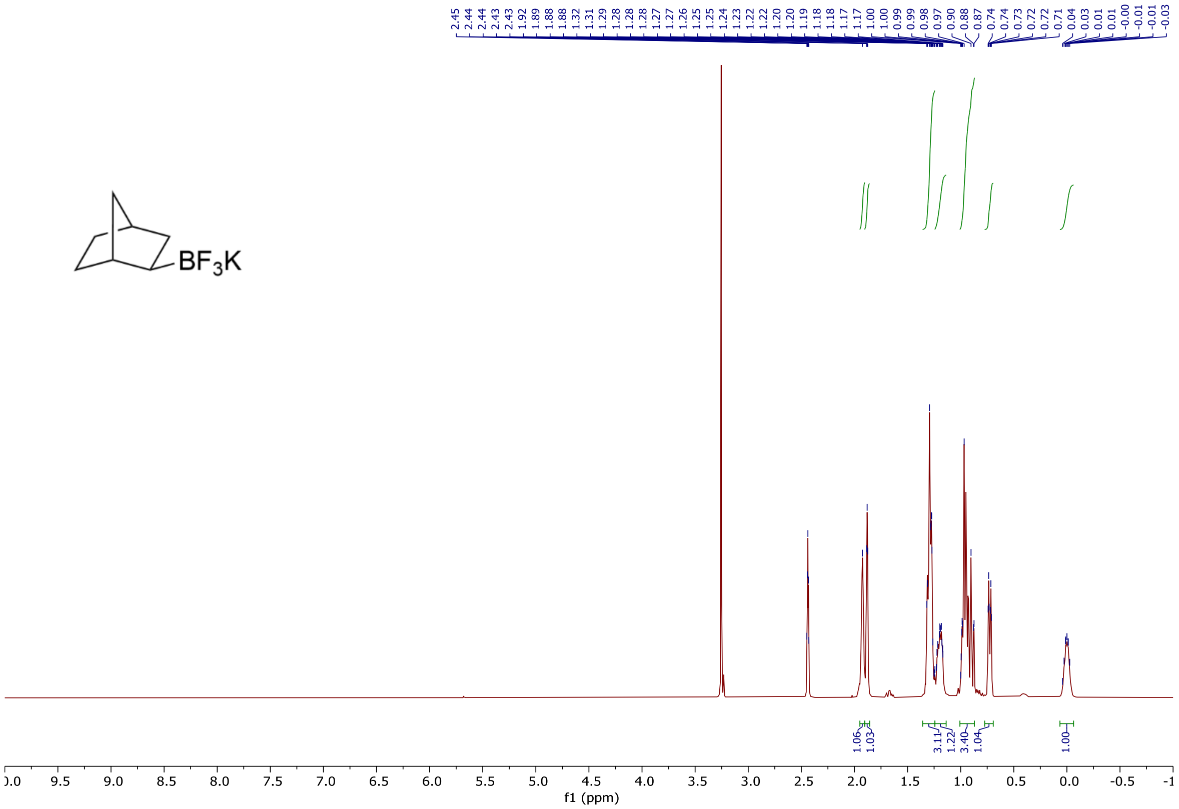

¹³C NMR (101 MHz, dmso-*d*6): **1o**

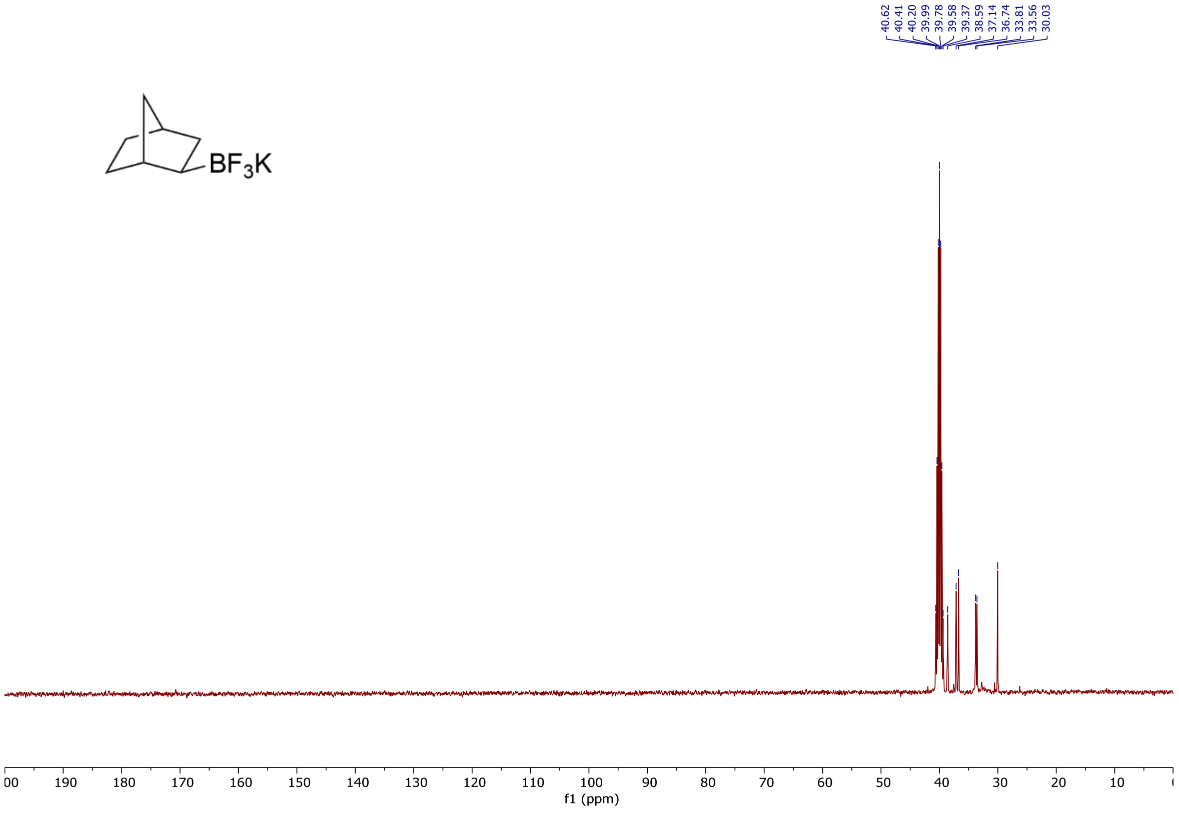

^{1}H NMR (400 MHz, dmso-d_6): **1p**

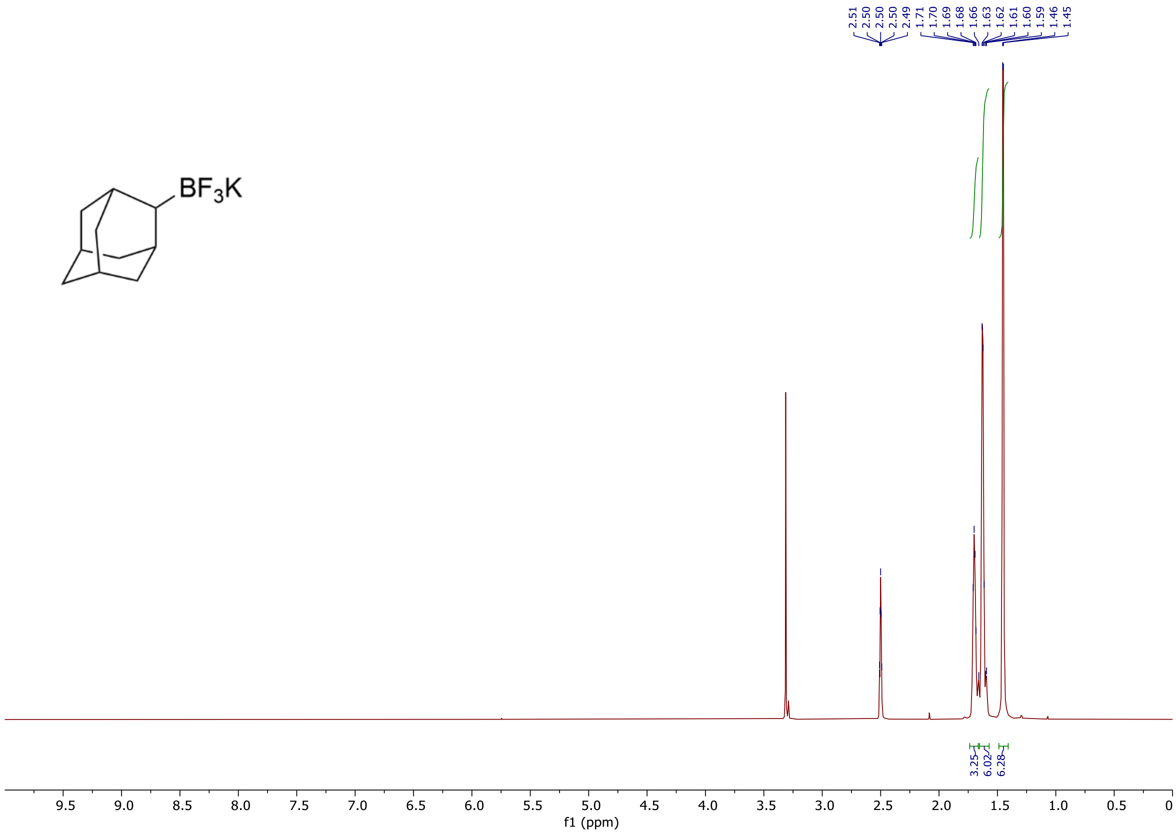

^{13}C NMR (101 MHz, dmso-d_6): **1p**

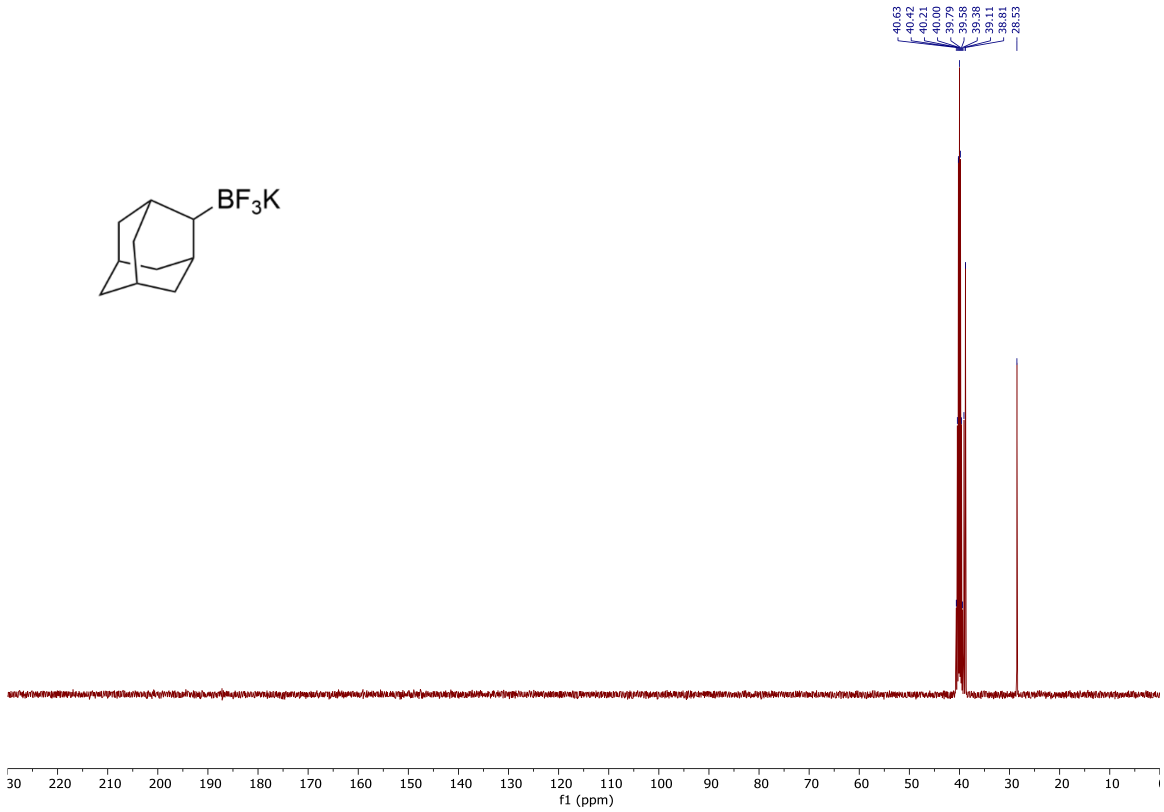

^{19}F NMR (377 MHz, dmso-*d*6): **1p**

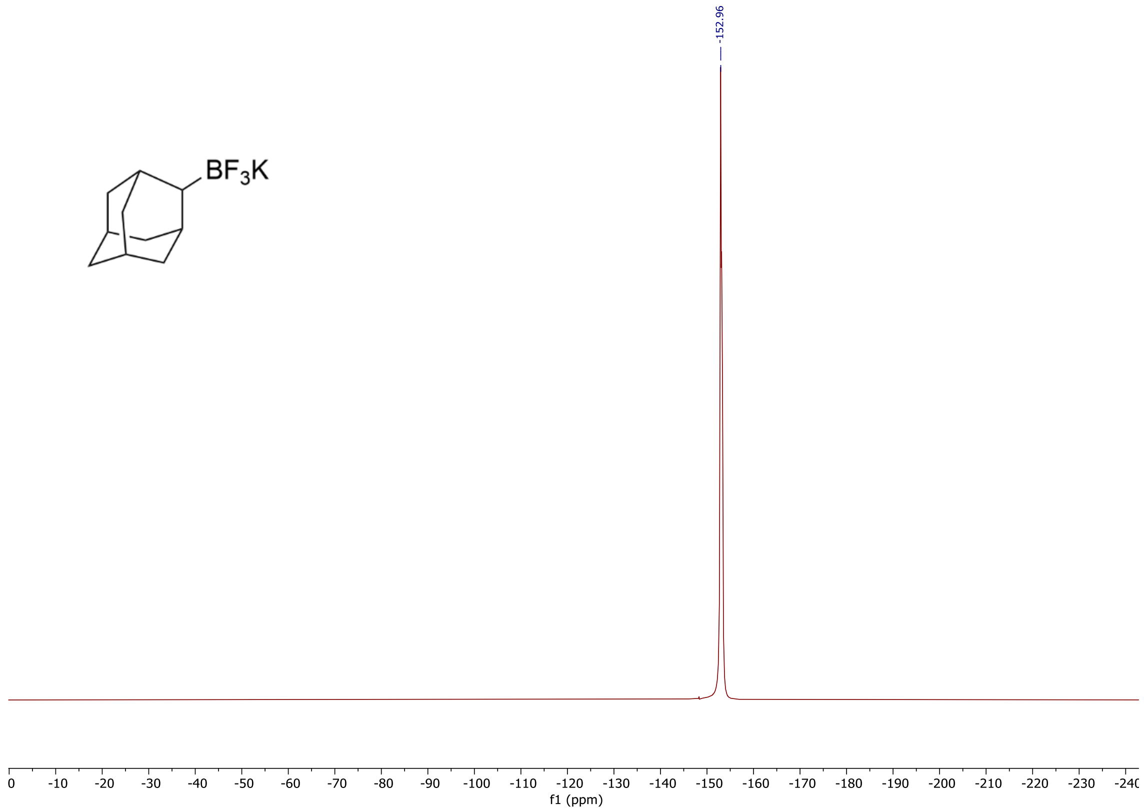

^{11}B NMR (128 MHz, dmso-*d*6): **1p**

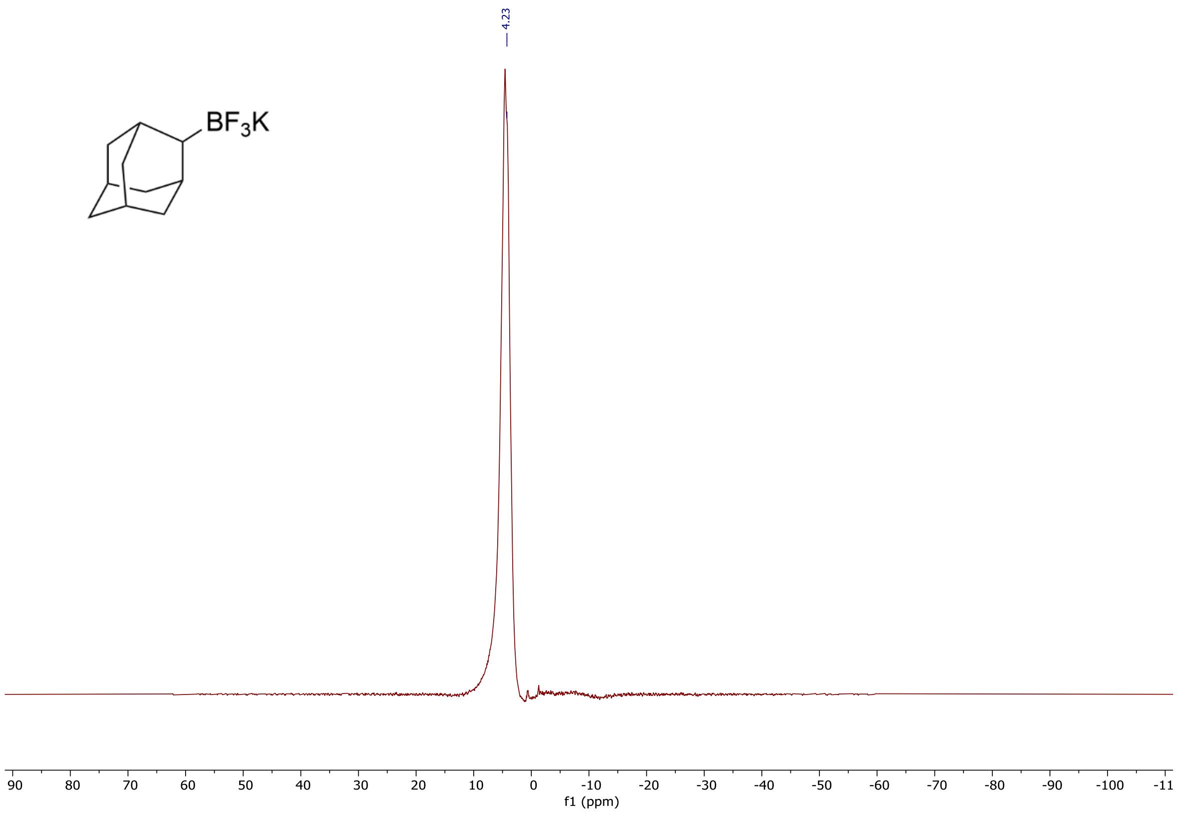

^{1}H NMR (400 MHz, dmso-d_6): **1r**

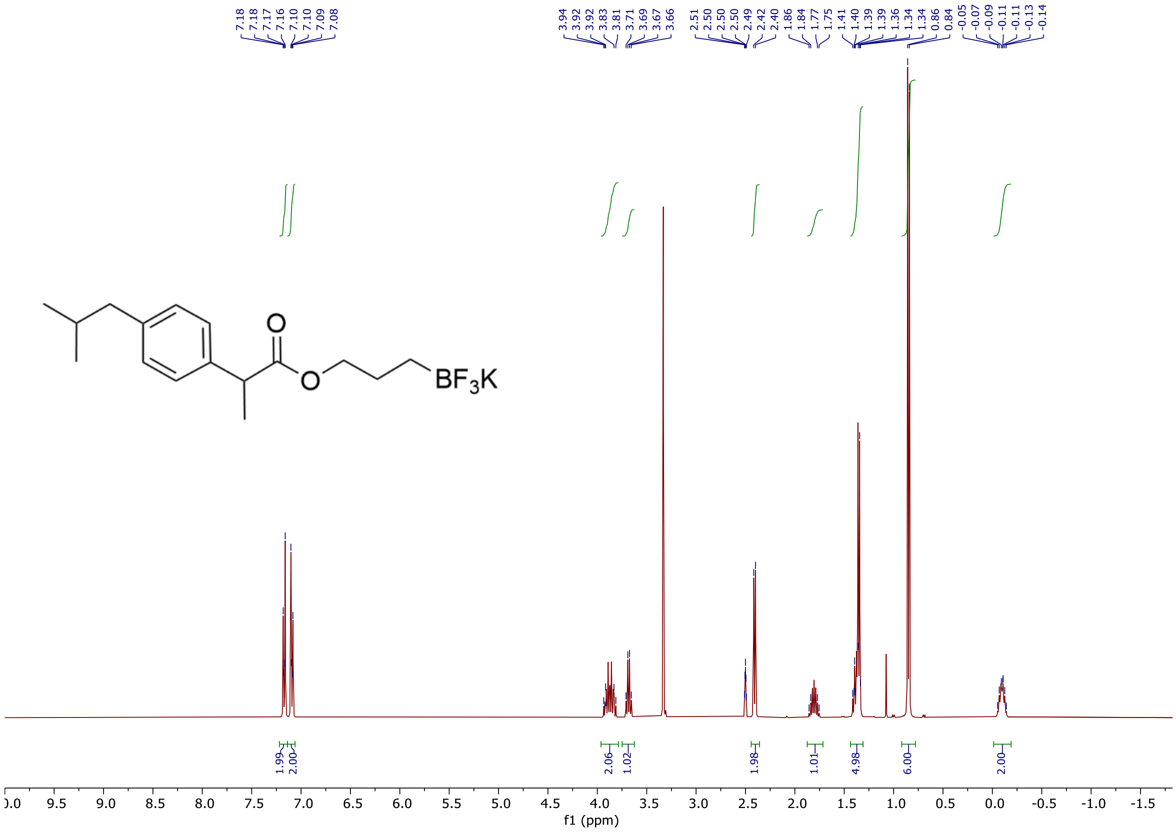

^{13}C NMR (101 MHz, dmso-d_6): **1r**

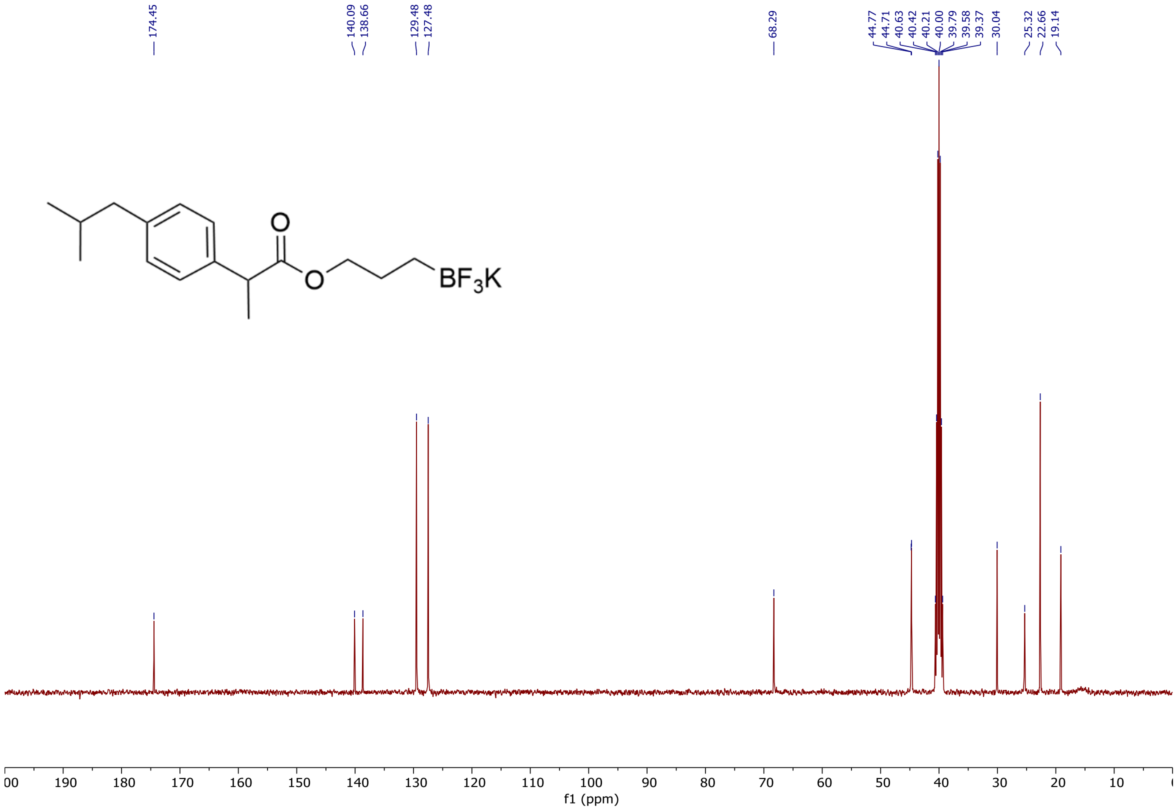

^{19}F NMR (377 MHz, dmso-d_6): **1r**

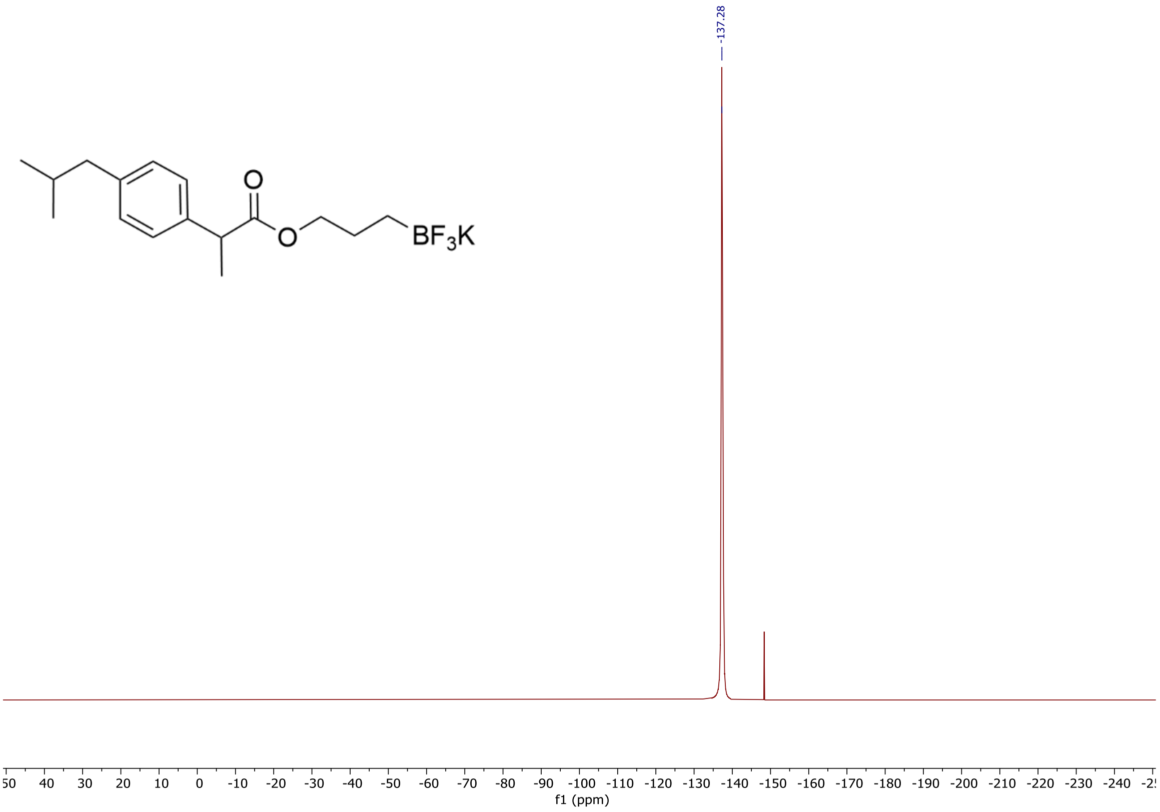

^{11}B NMR (128 MHz, dmso-d_6): **1r**

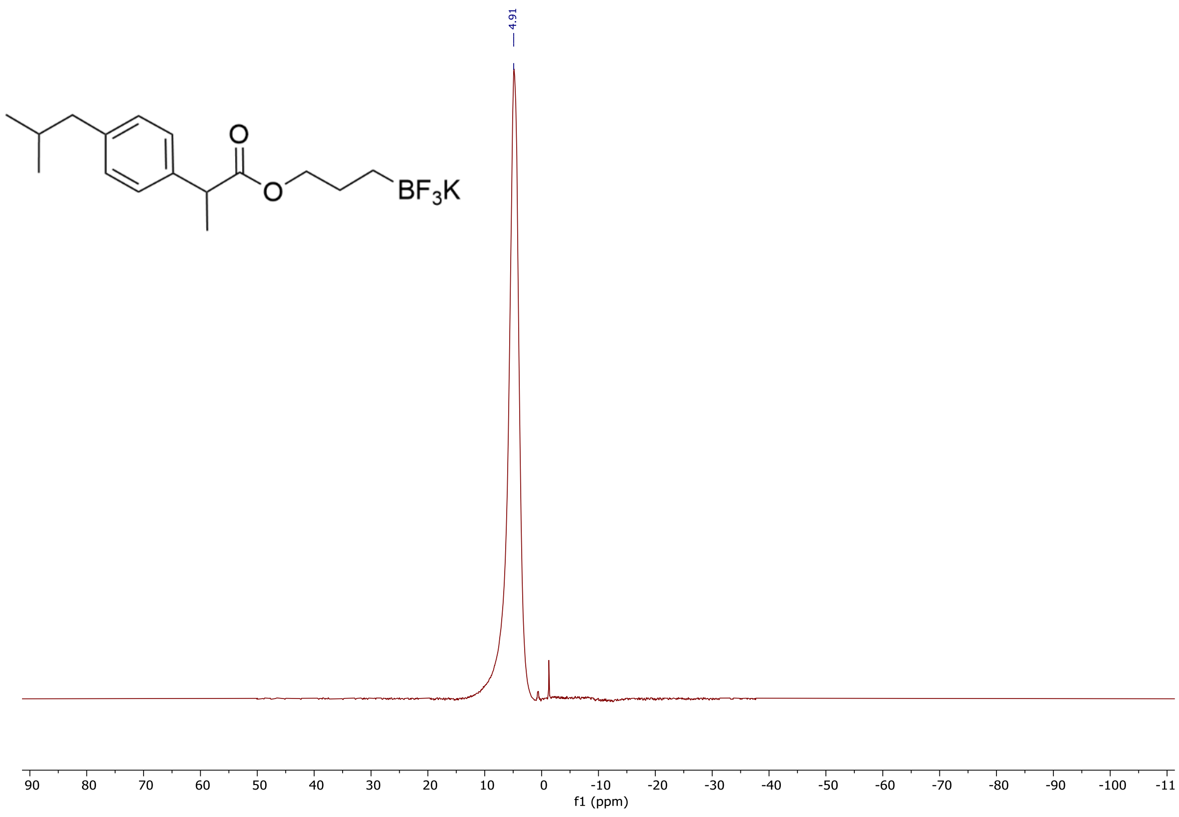

^{1}**H NMR** (400 MHz, dmso-d_6): **1s**

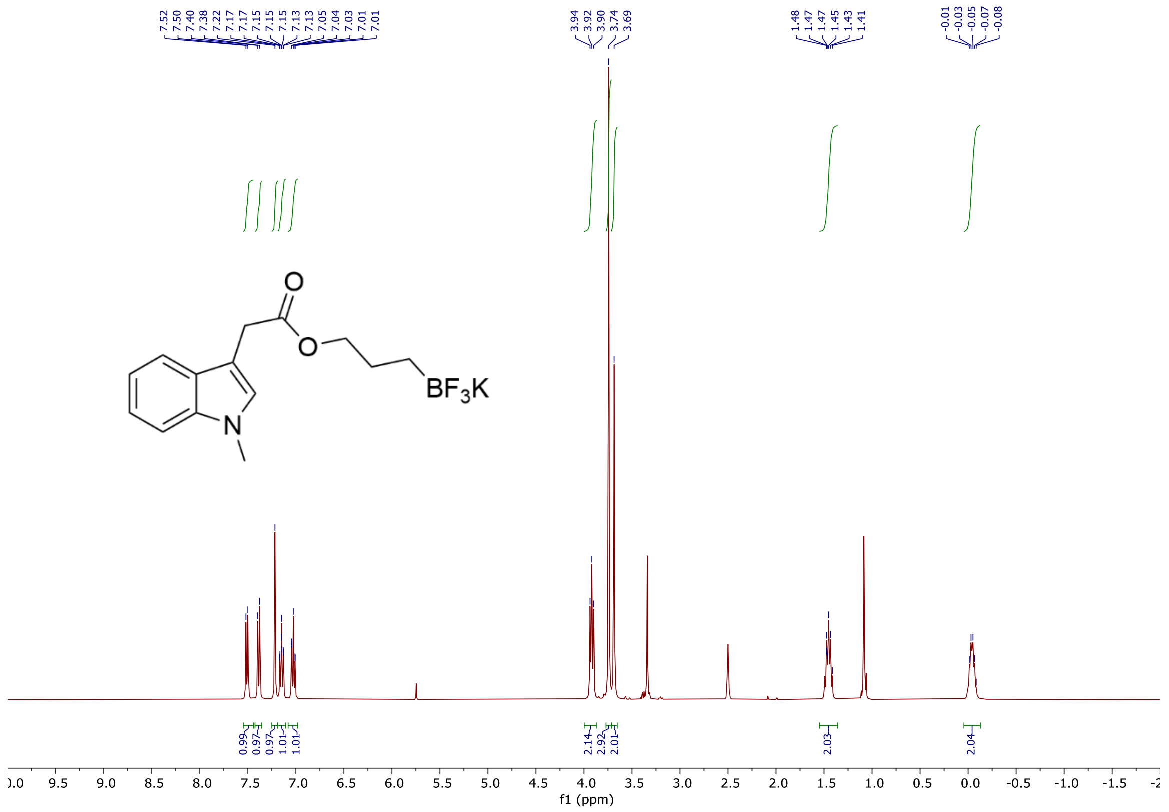

^{13}**C NMR** (101 MHz, dmso-d_6): **1s**

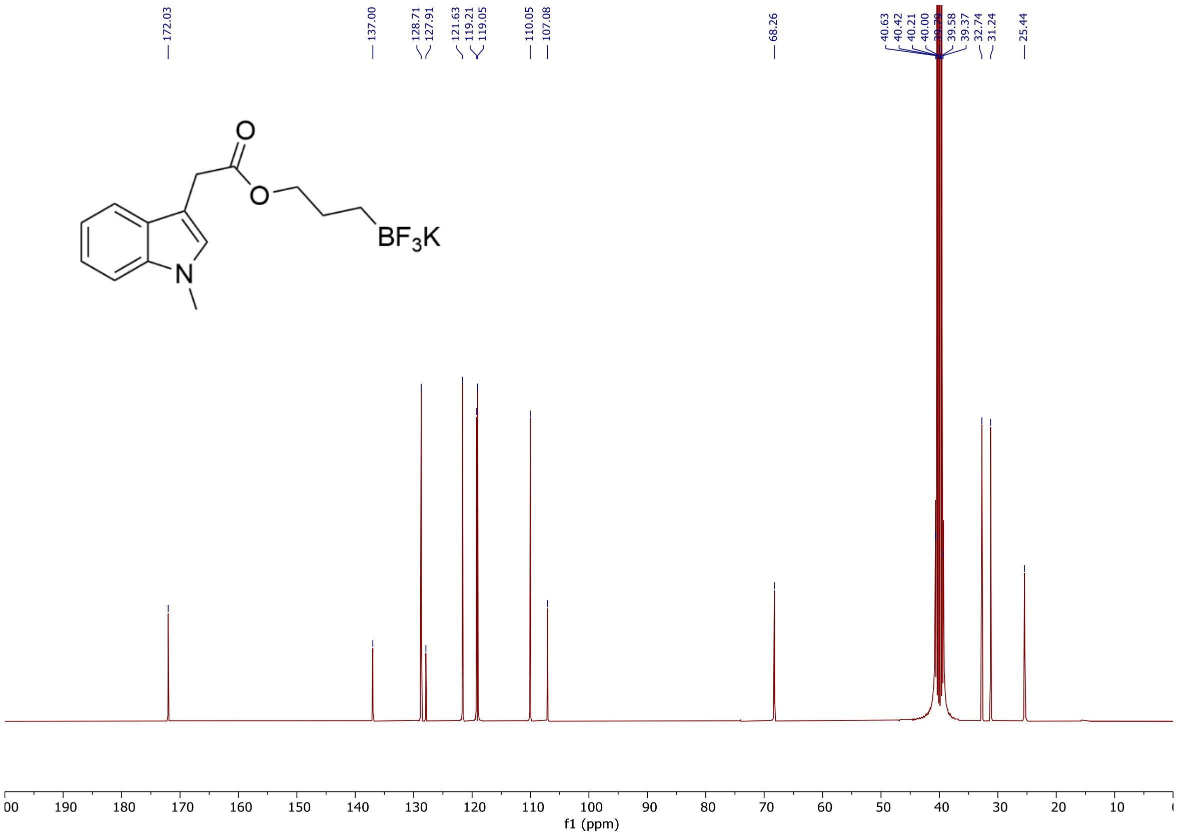

^{19}F NMR (377 MHz, dmso-d_6): **1s**

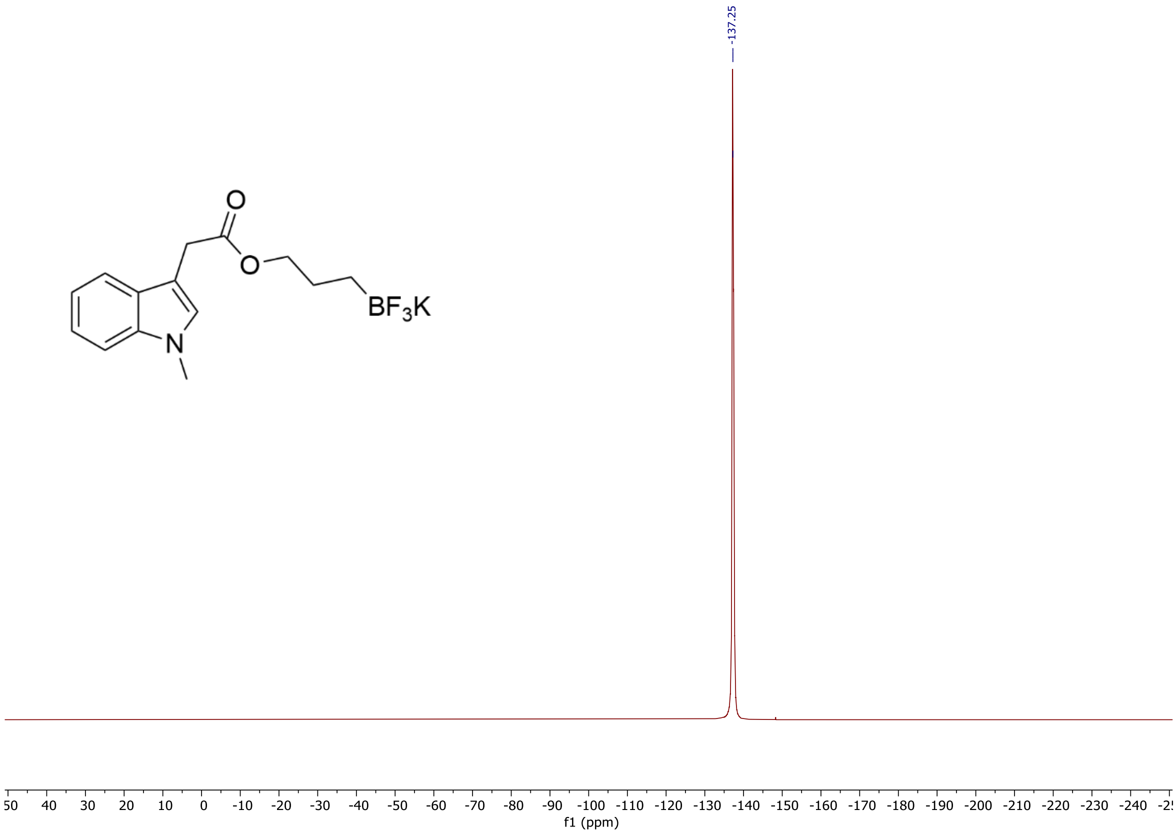

^{11}B NMR (128 MHz, dmso-d_6): **1s**

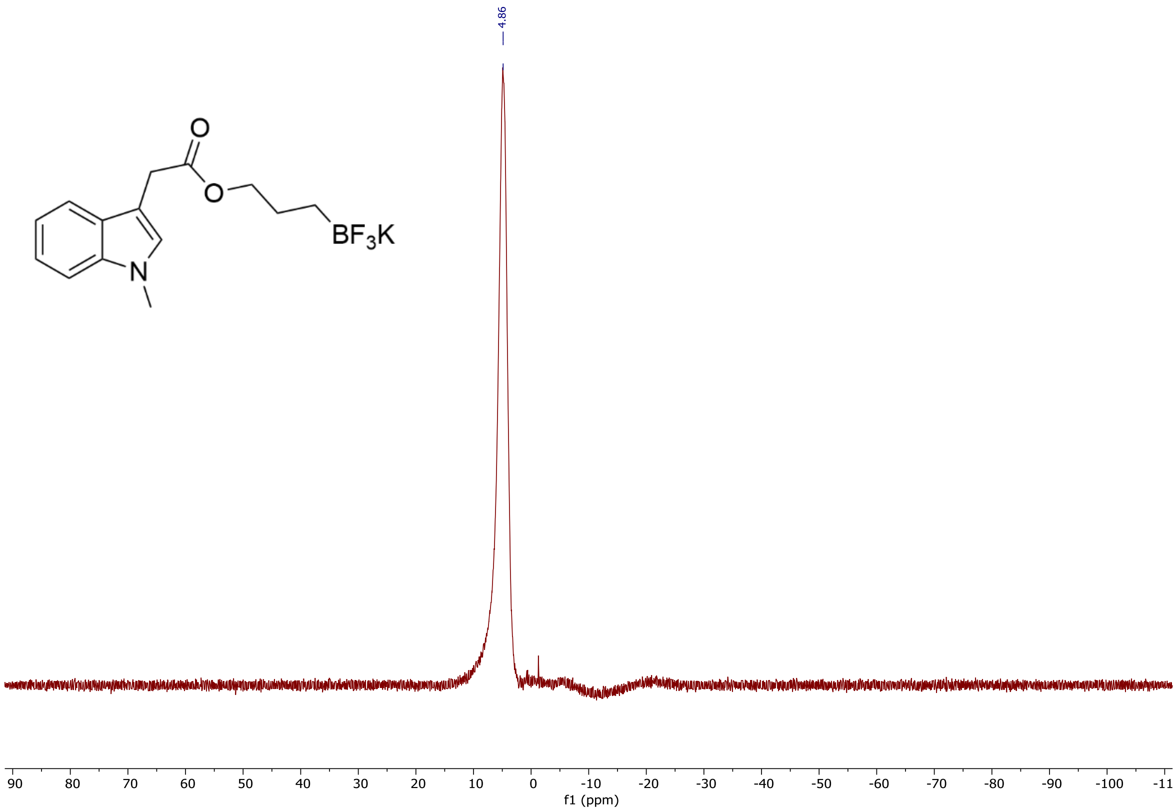

^{1}**H NMR** (400 MHz, acetone-d_6): **1t**

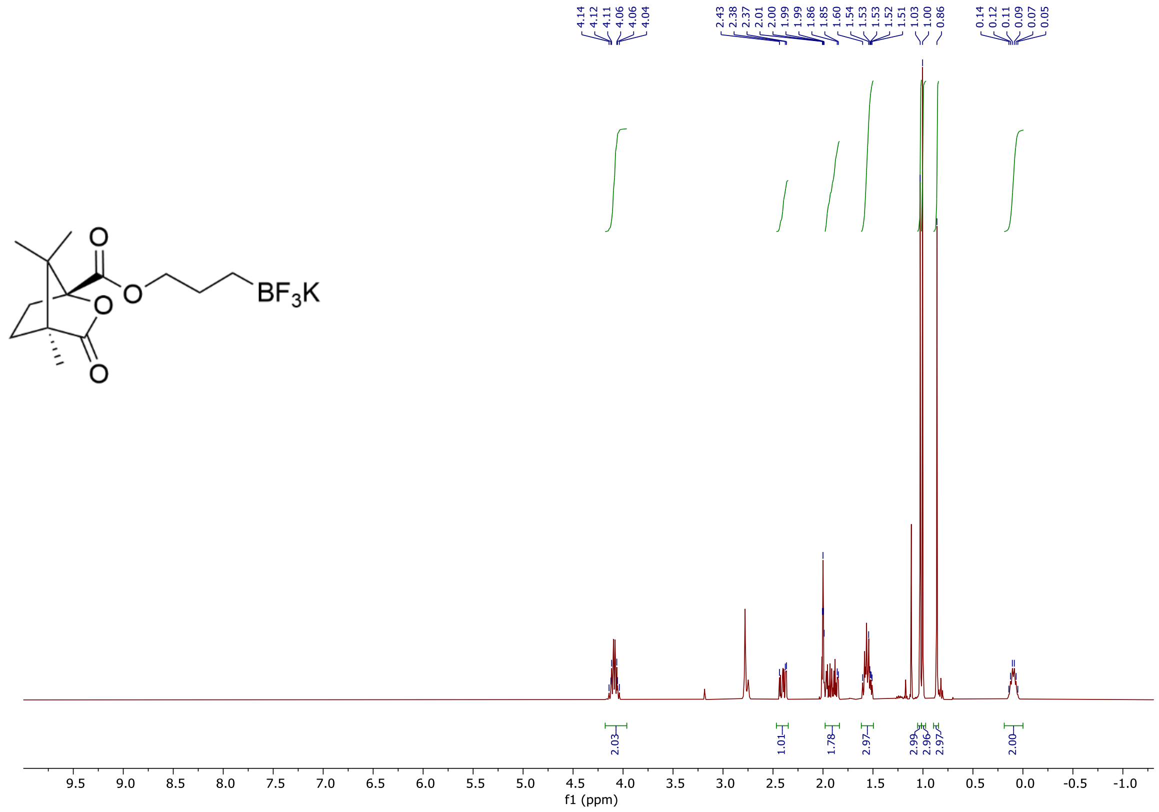

^{13}**C NMR** (101 MHz, acetone-d_6): **1t**

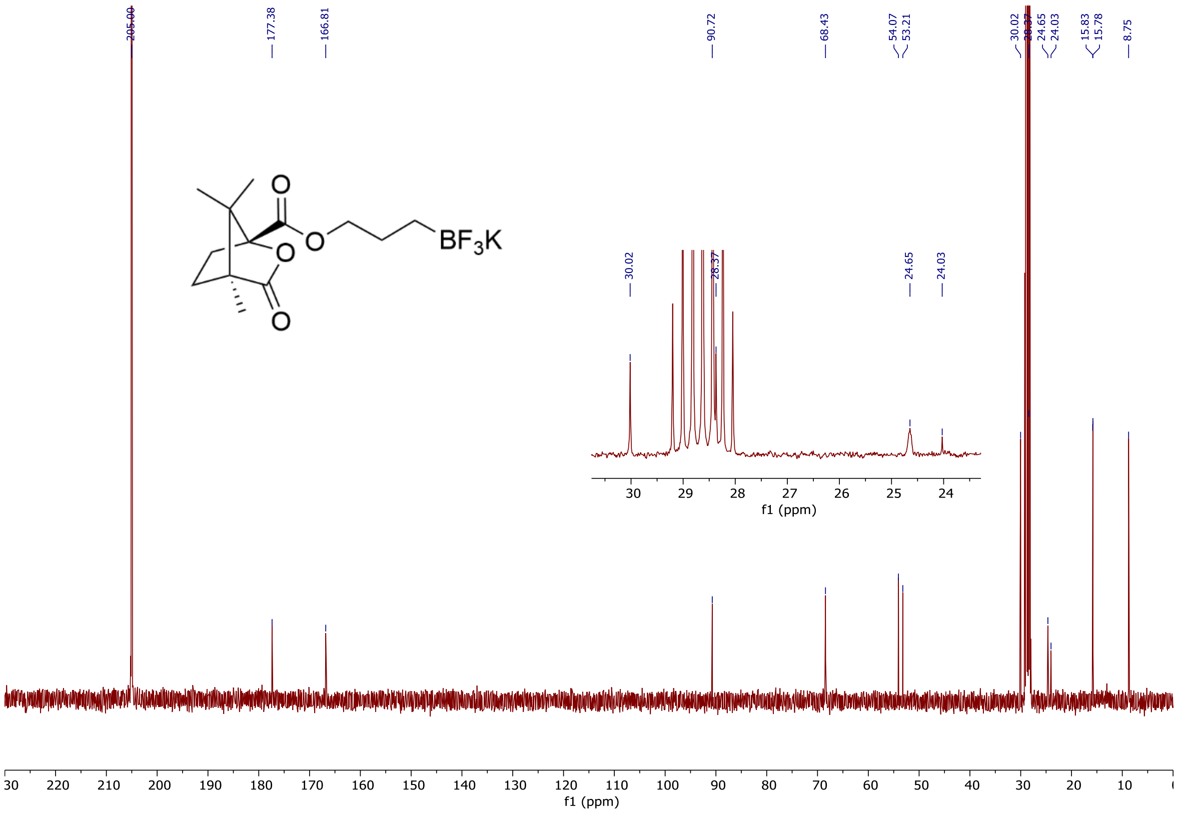

^{19}F NMR (377 MHz, acetone-d_6): **1t**

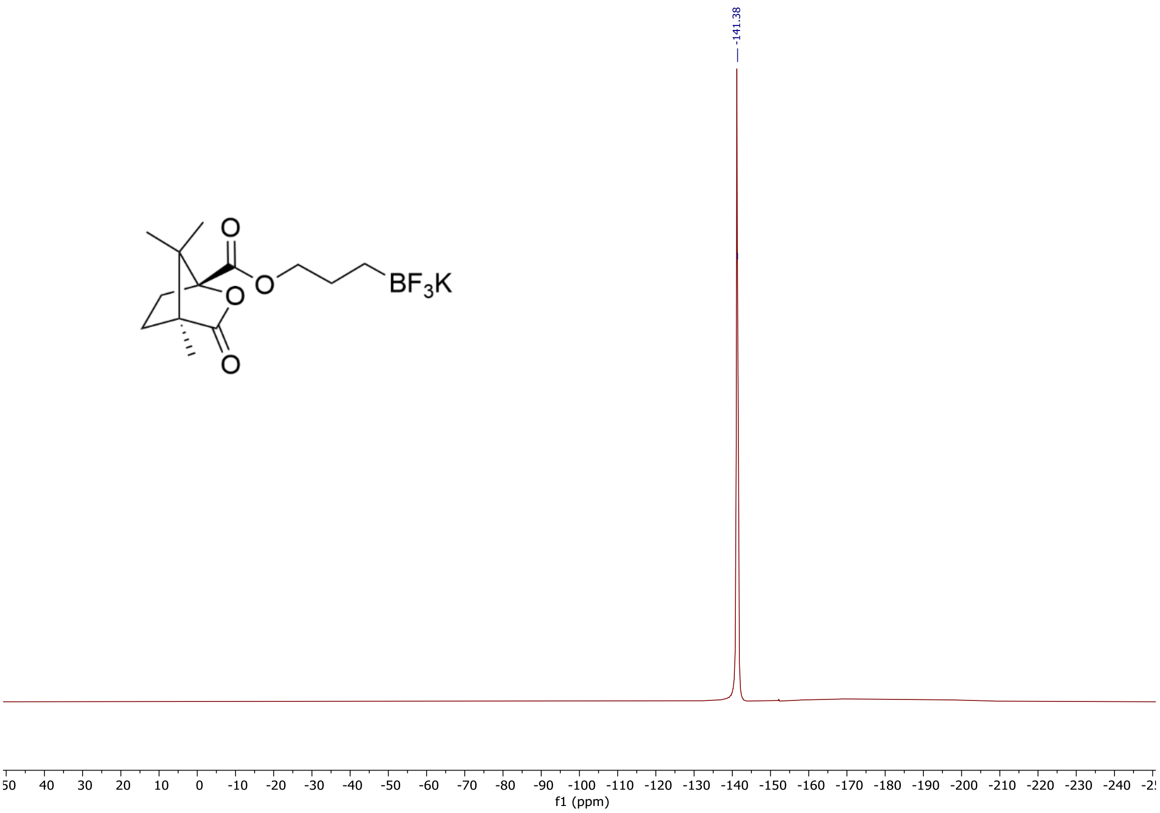

^{11}B NMR (128 MHz, acetone-d_6): **1t**

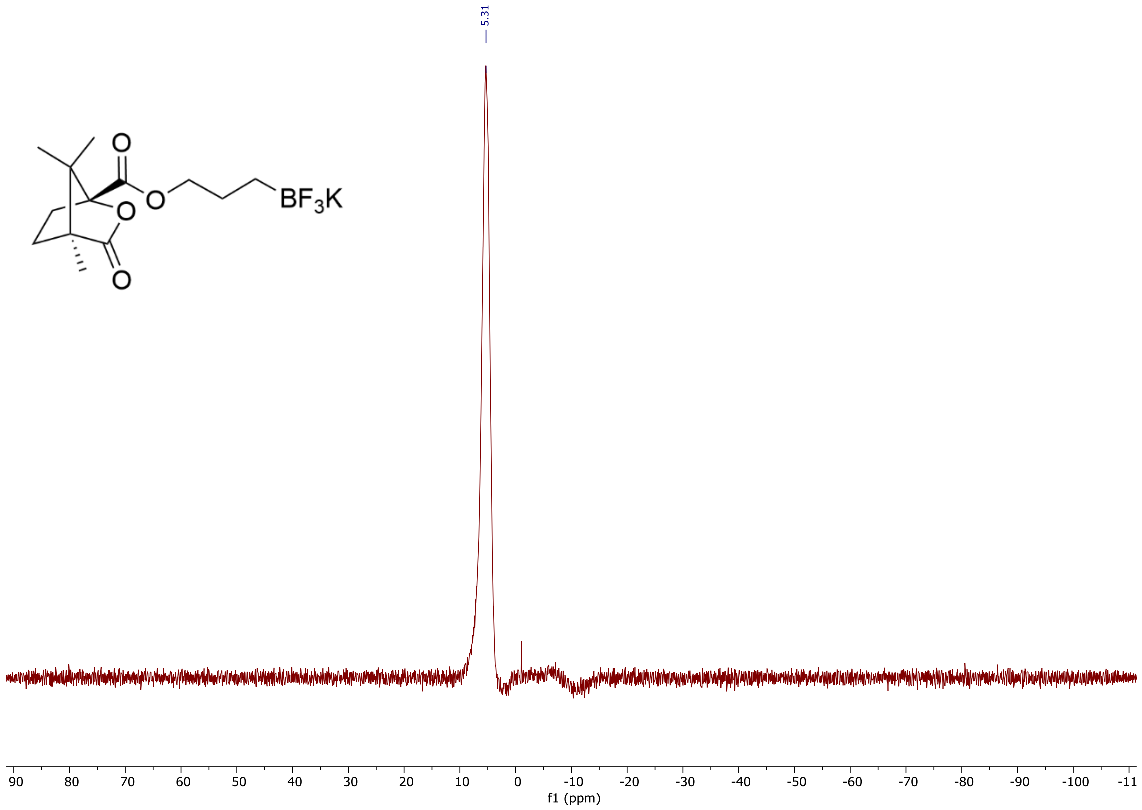

^{1}H NMR (400 MHz, dmso-d_6): **1u**

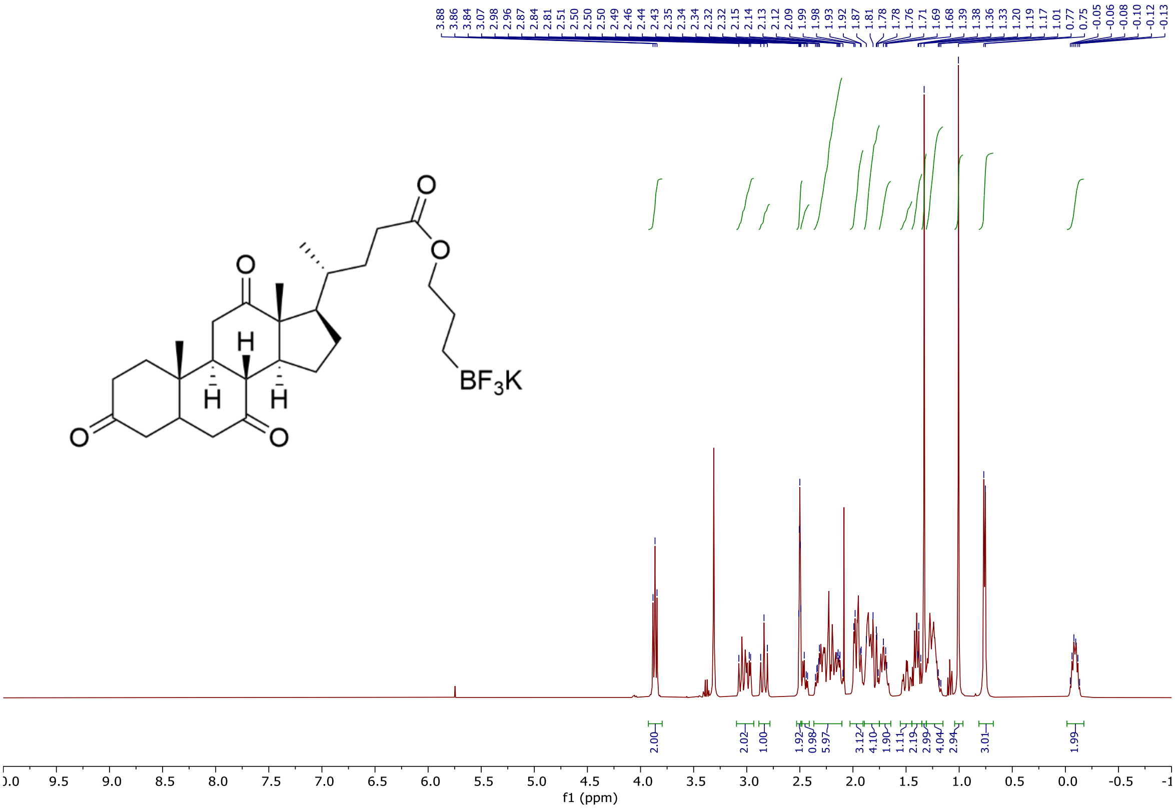

^{13}C NMR (101 MHz, dmso-d_6): **1u**

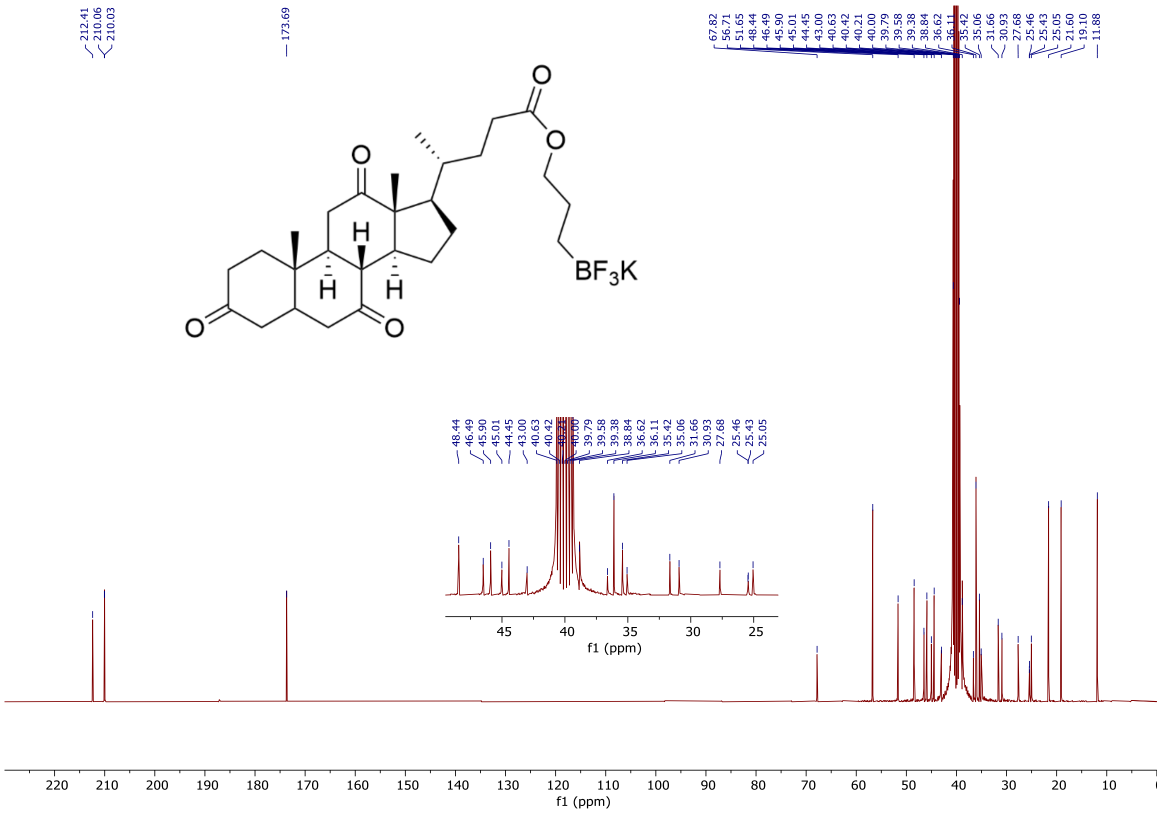

^{19}F NMR (377 MHz, dmso-d_6): **1u**

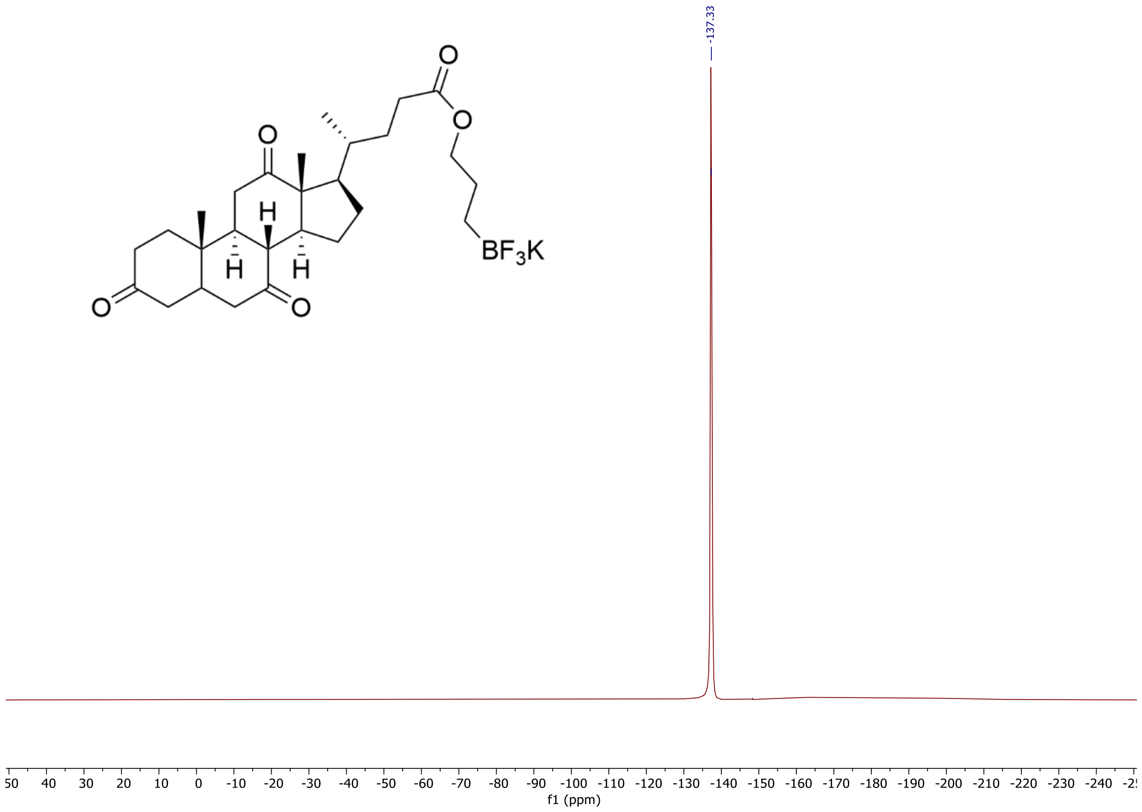

^{11}B NMR (128 MHz, dmso-d_6): **1u**

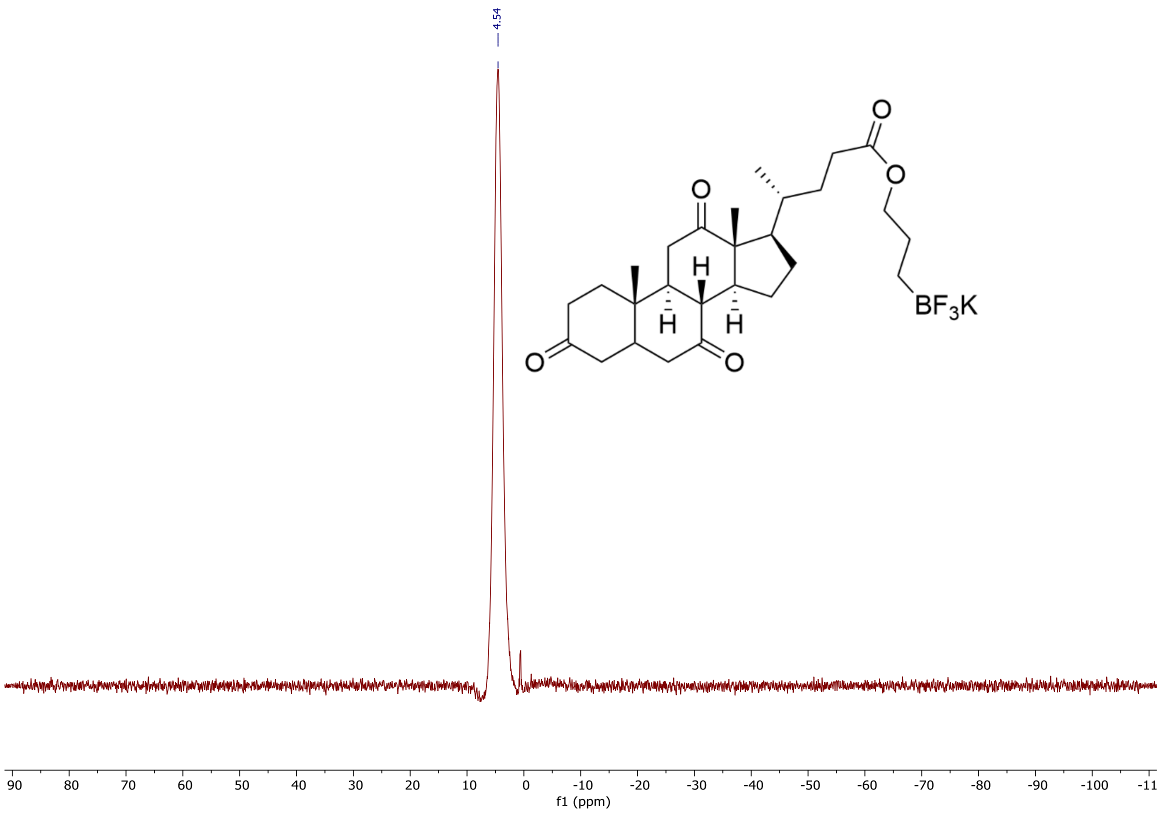

^{1}H NMR (400 MHz, acetone-d_6): **1at**

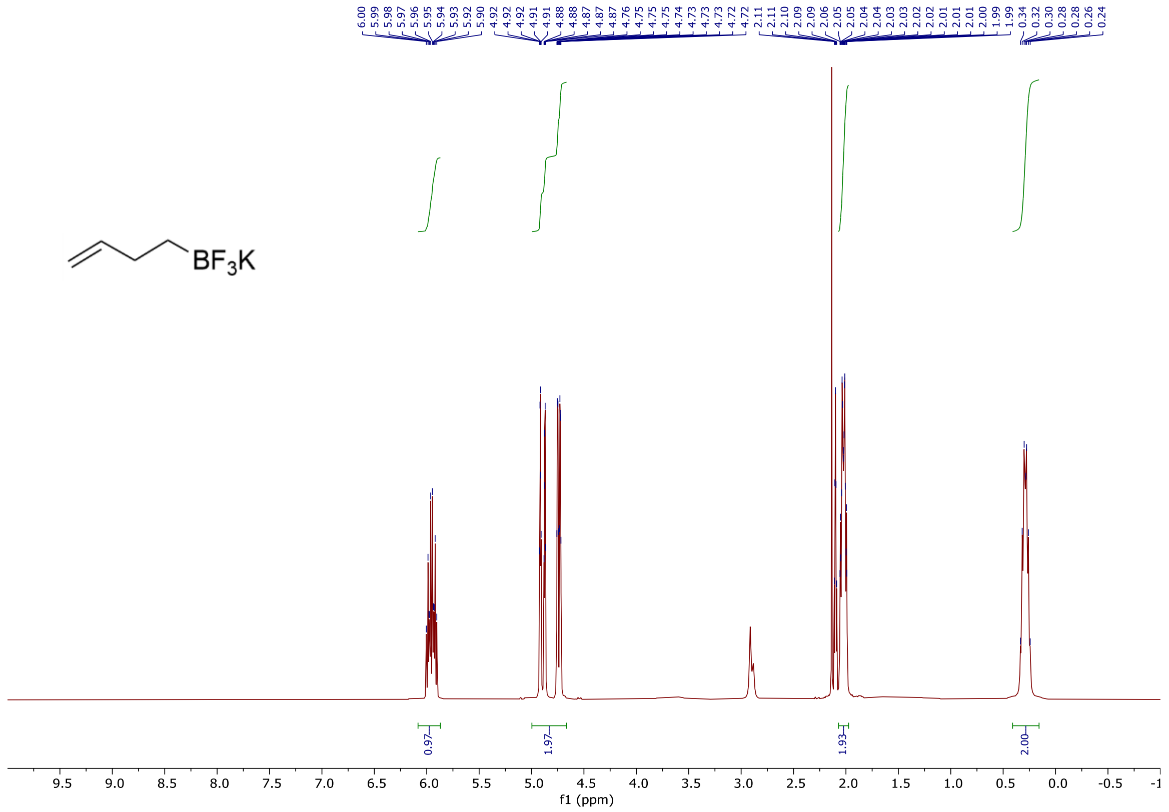

^{13}C NMR (101 MHz, acetone-d_6): **1at**

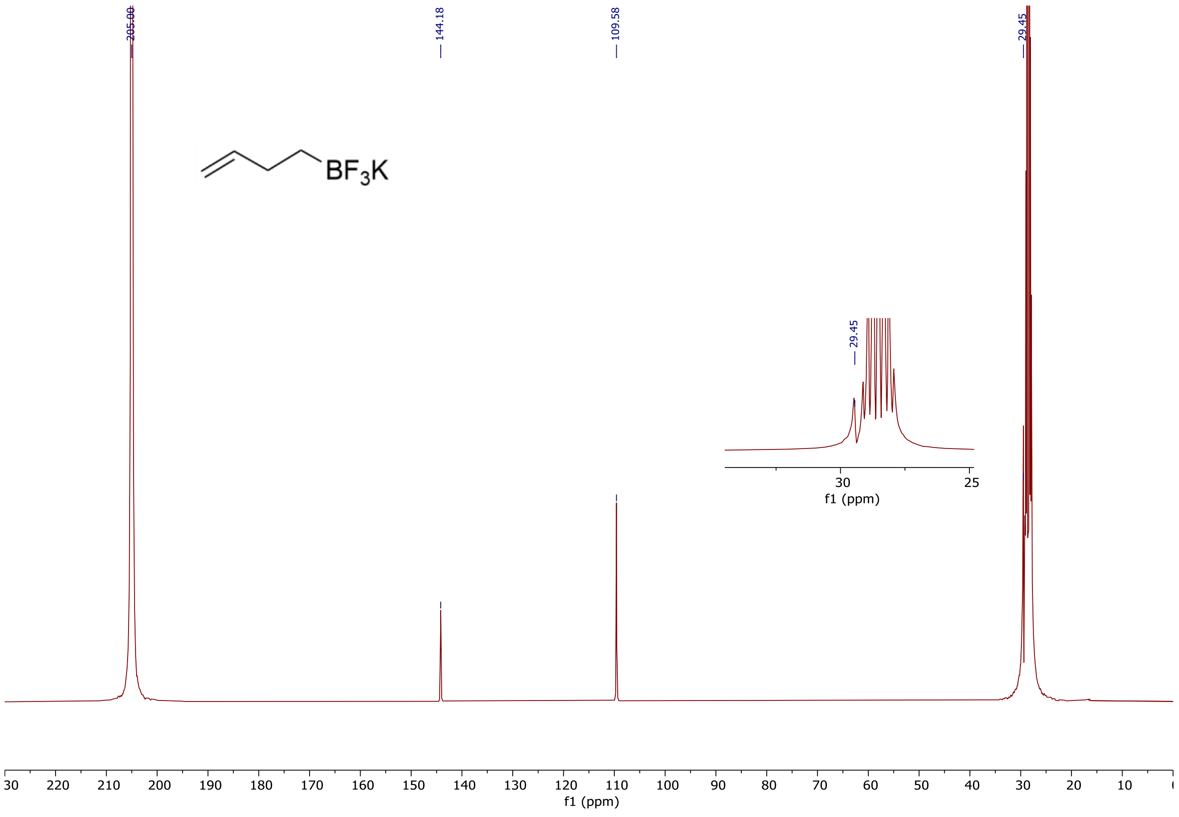

^{1}H NMR (400 MHz, dmso-d_6): **1bb**

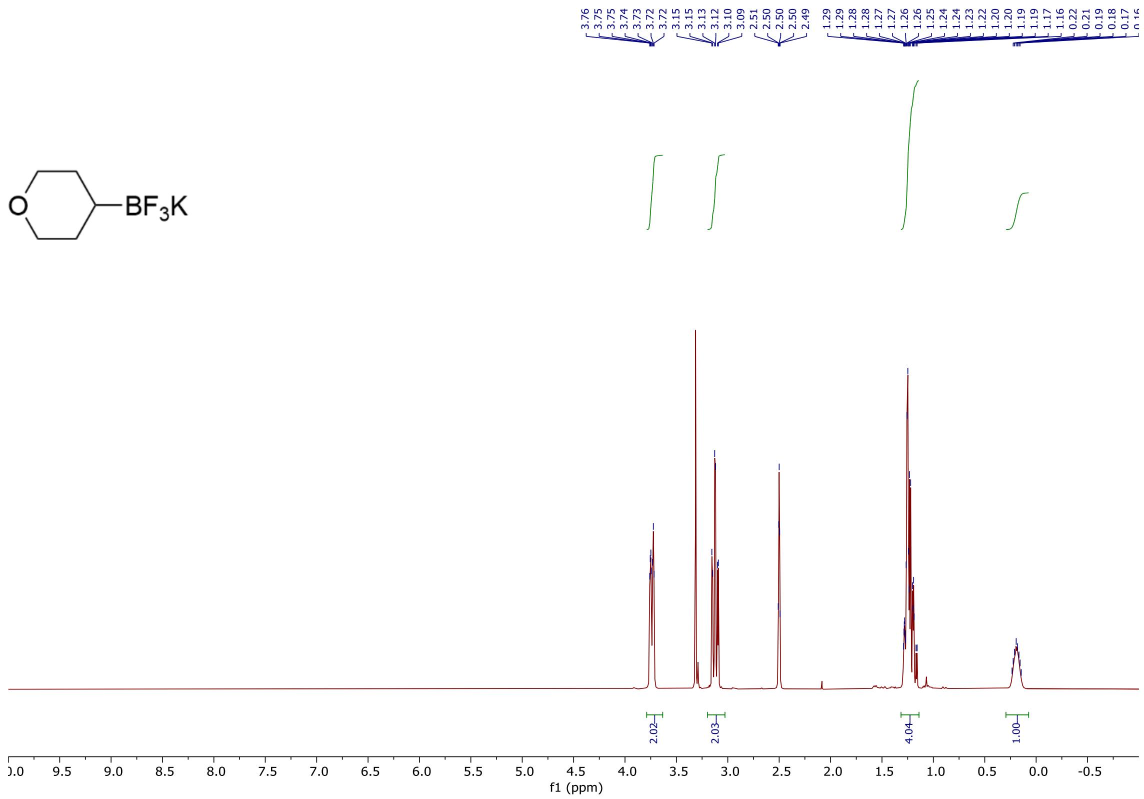

^{13}C NMR (101 MHz, dmso-d_6): **1bb**

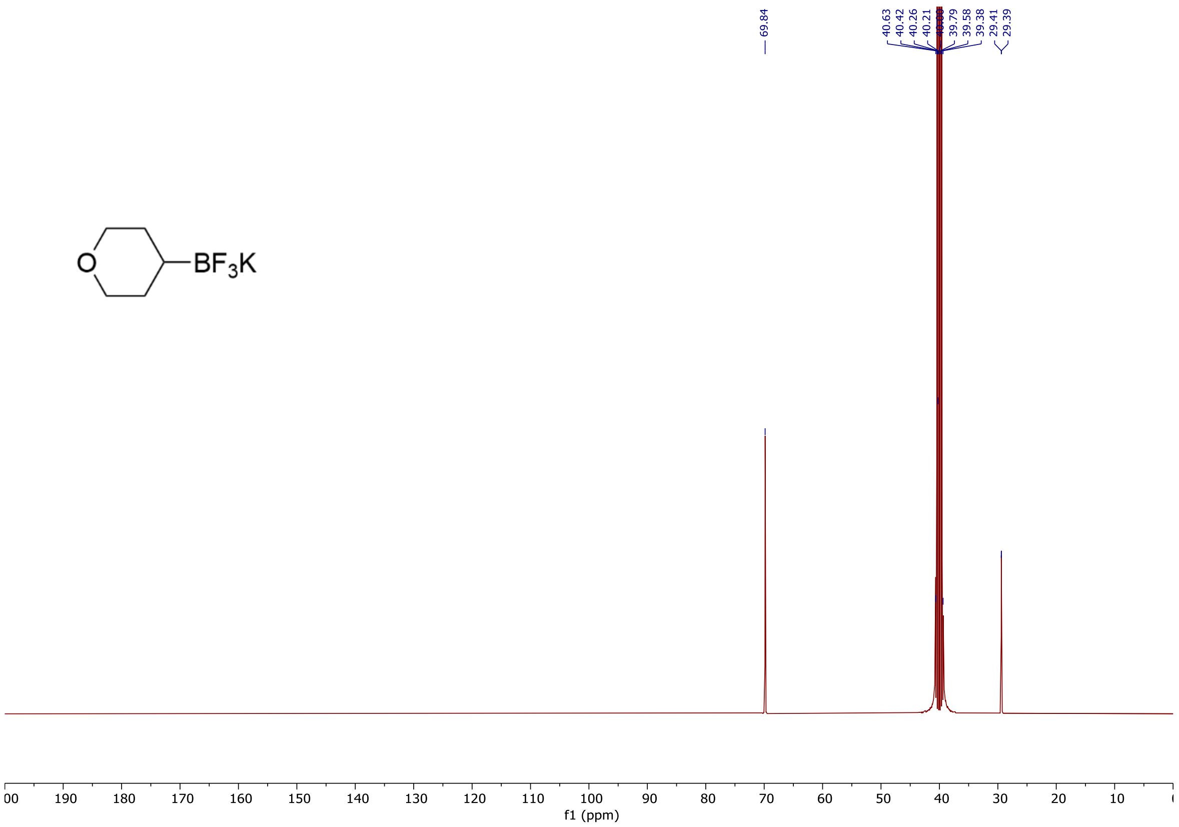

^{1}**H NMR** (400 MHz, dmso-d_6): **1bc**

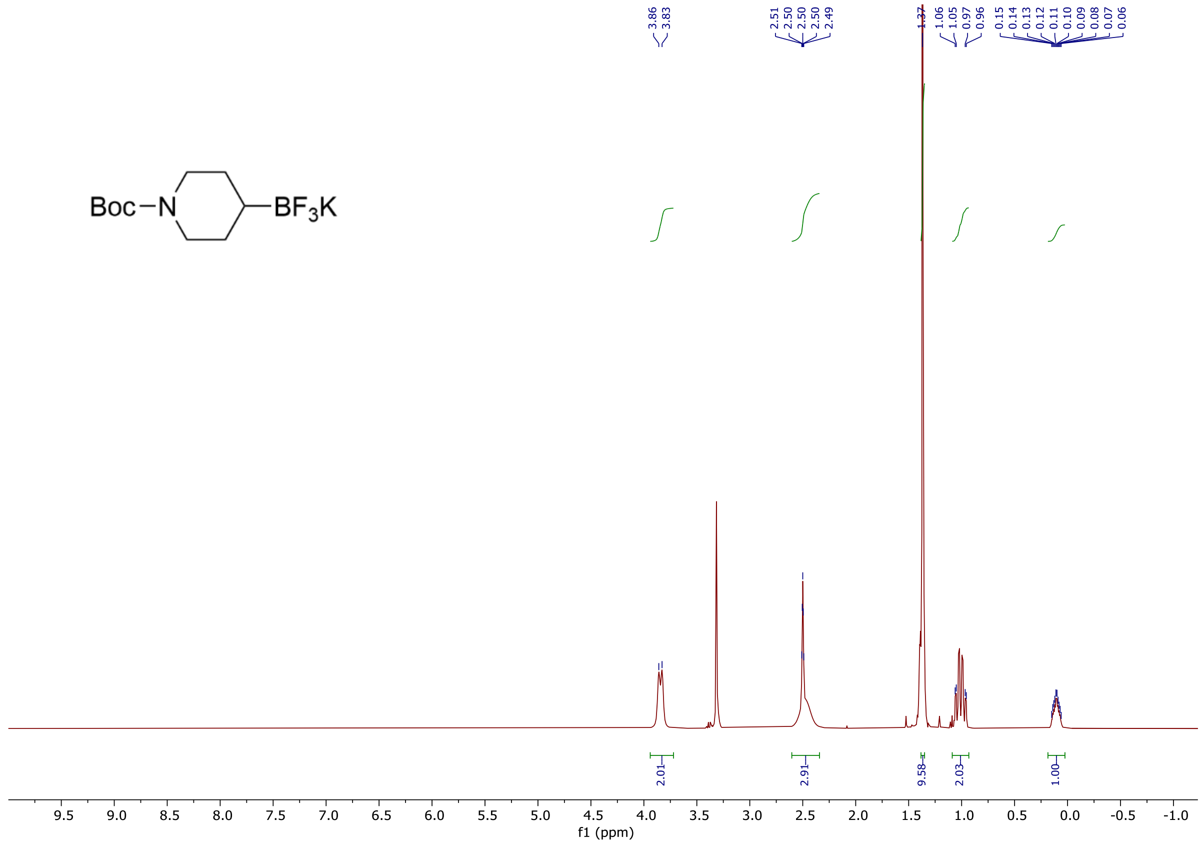

^{13}**C NMR** (101 MHz, dmso-d_6): **1bc**

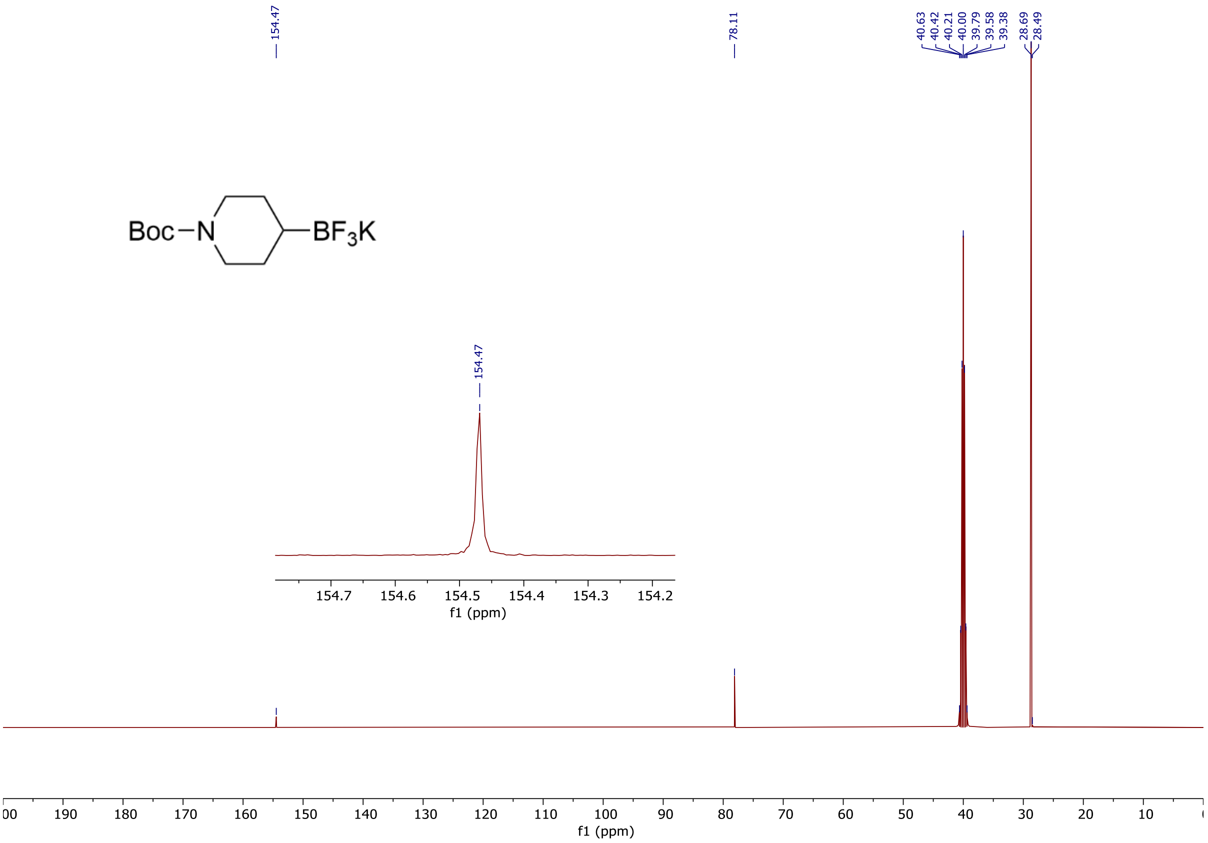

^{1}H NMR (400 MHz, MeOD): **1bd**

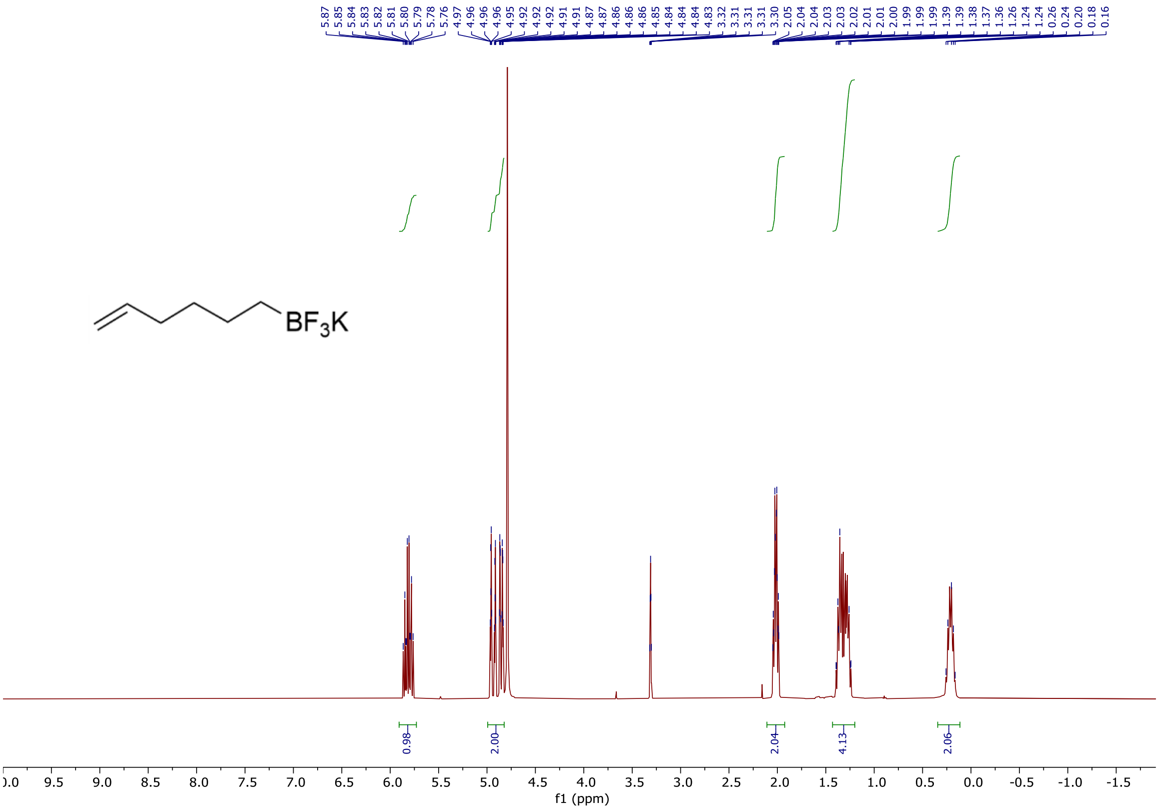

^{13}C NMR (101 MHz, MeOD): **1bd**

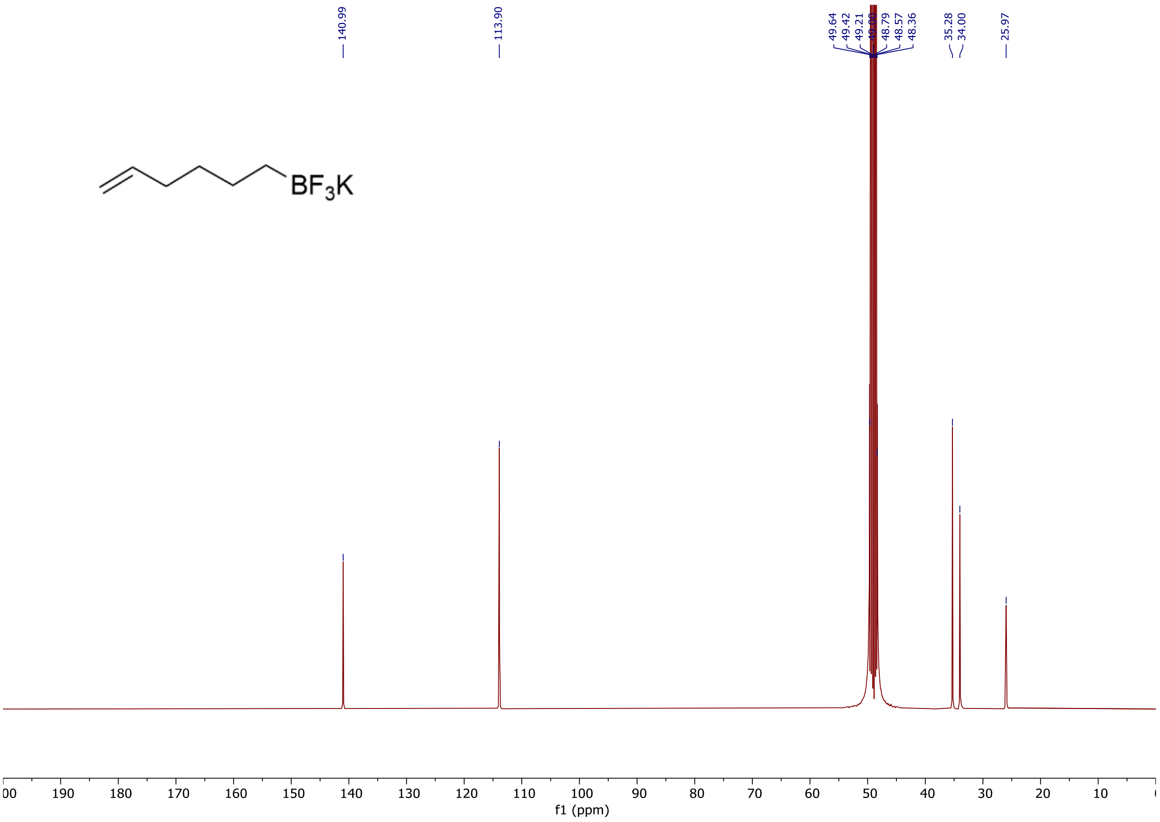

^{1}H NMR (400 MHz, $CDCl_3$): **4a**

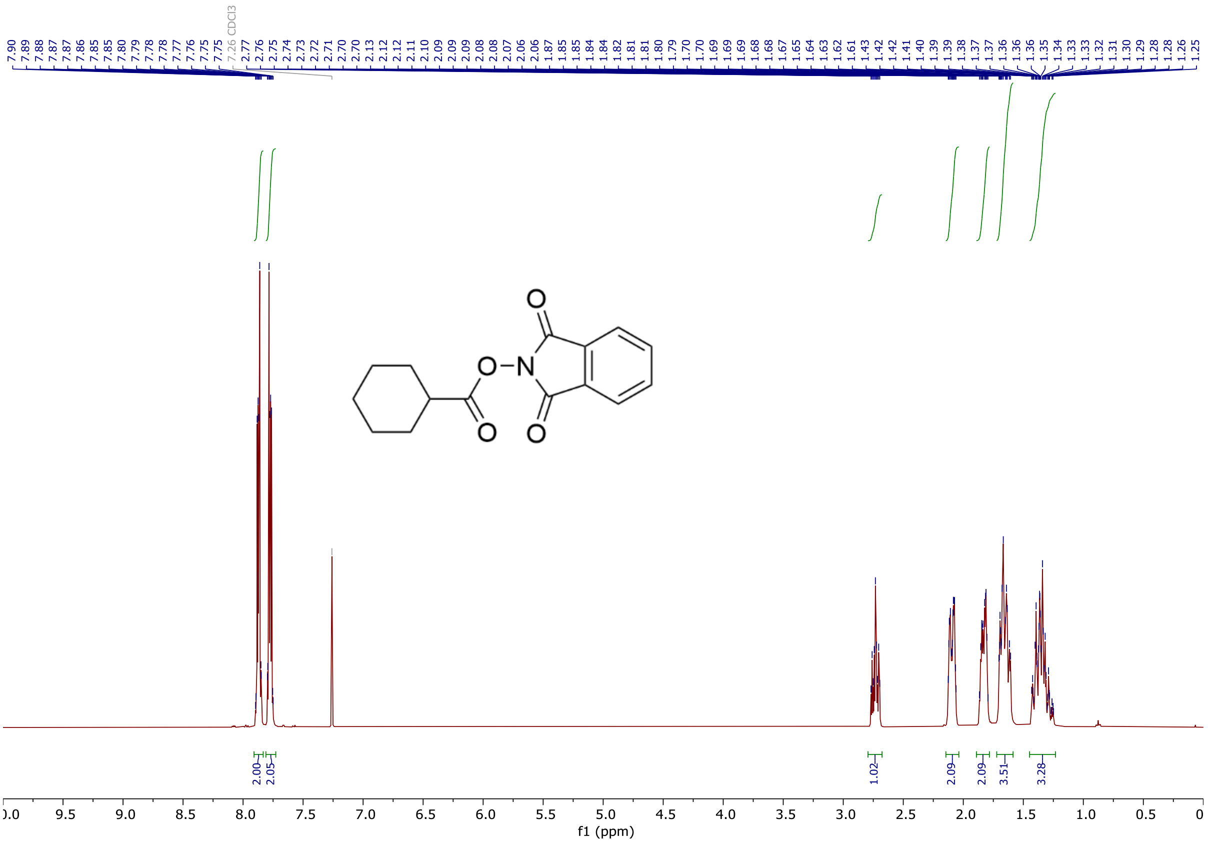

^{13}C NMR (101 MHz, $CDCl_3$): **4a**

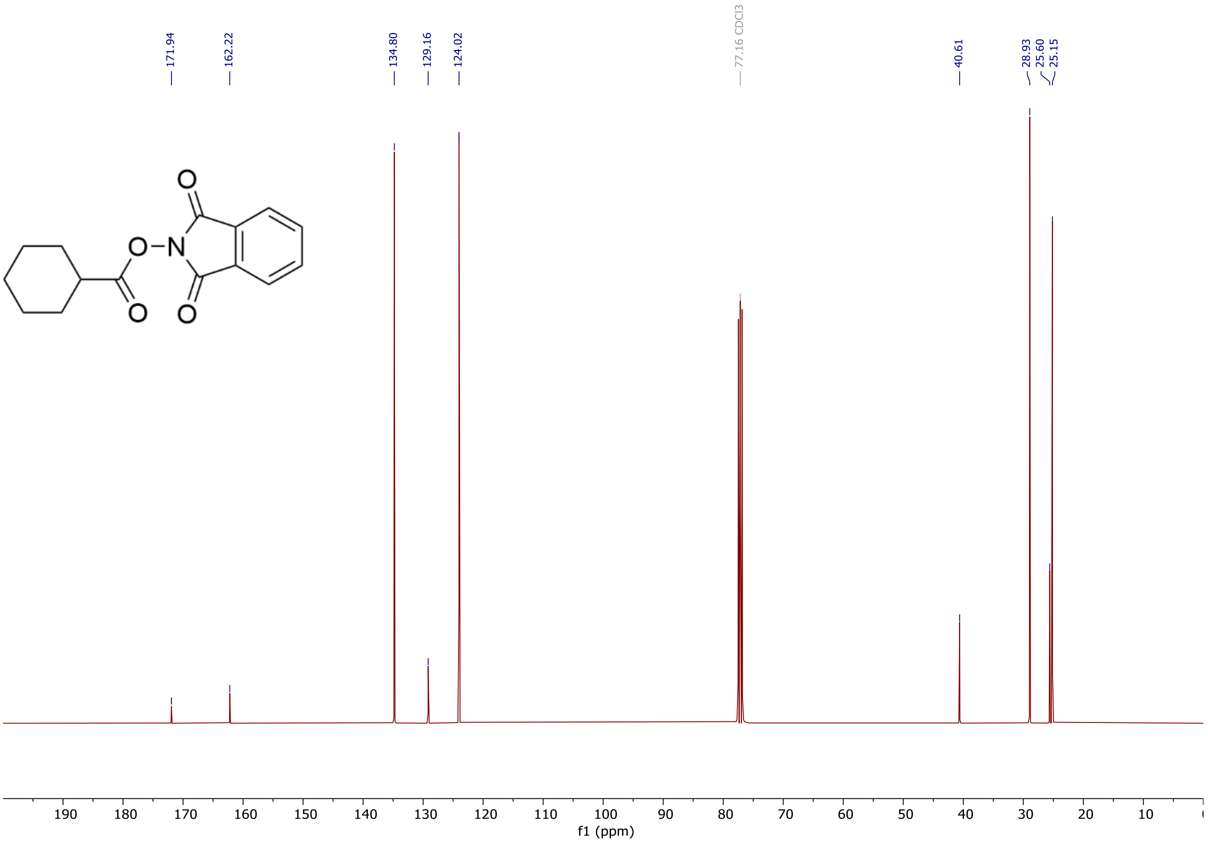

^{1}H NMR (400 MHz, $CDCl_3$): **4v**

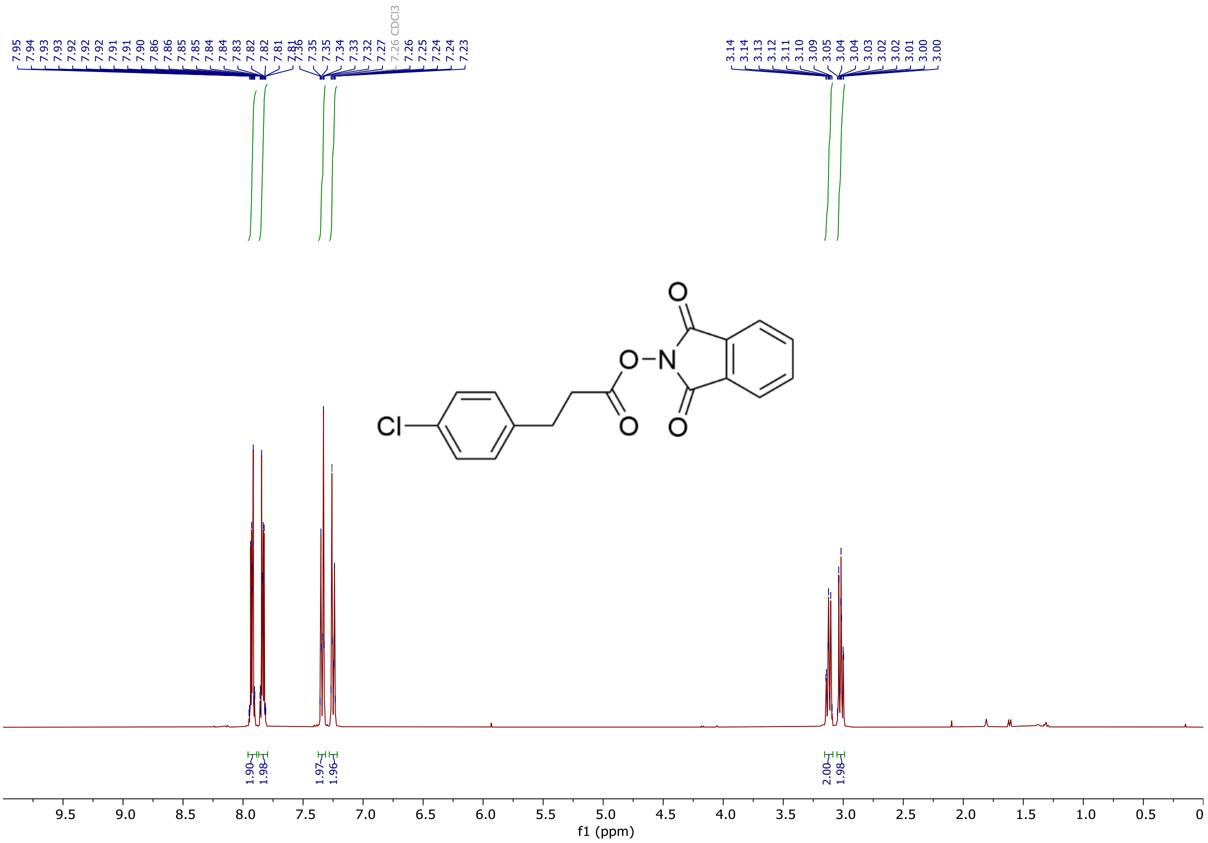

^{13}C NMR (101 MHz, $CDCl_3$): **4v**

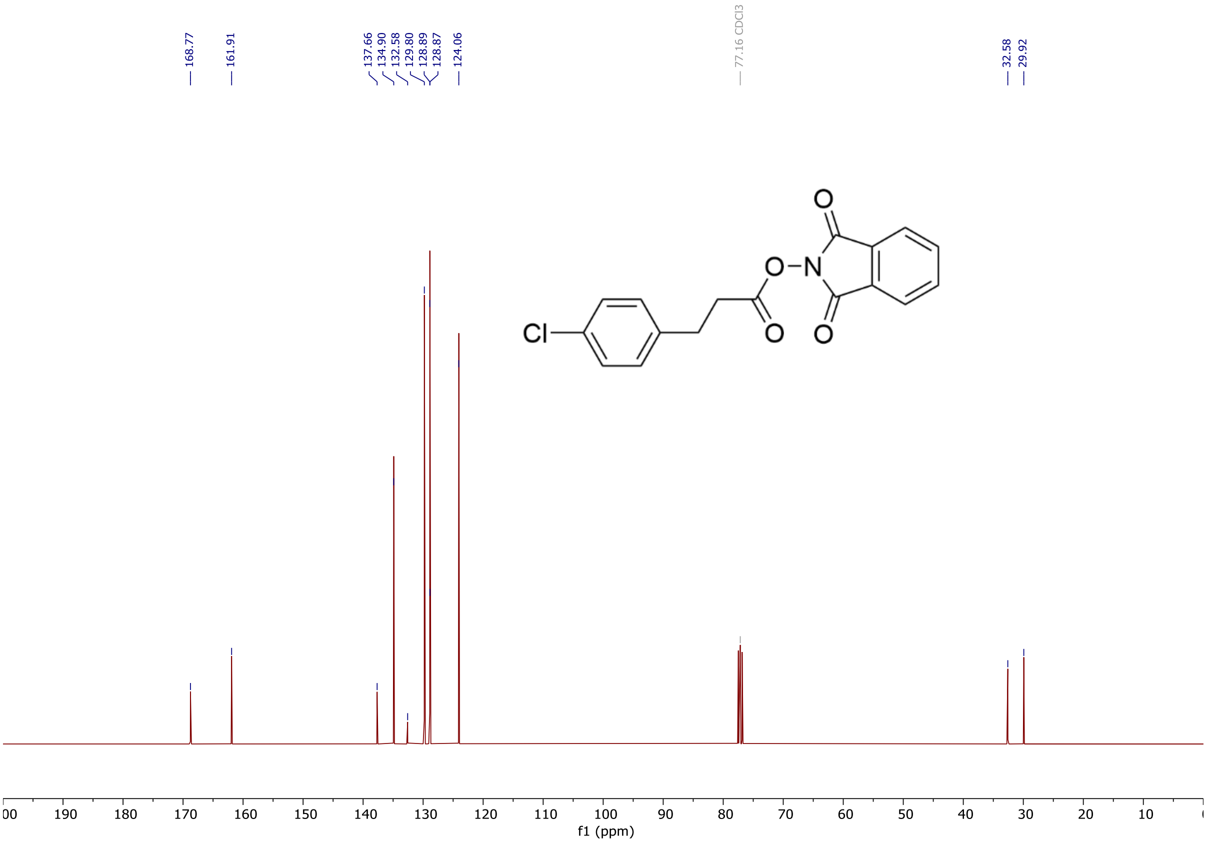

^{1}H NMR (400 MHz, $CDCl_3$): **4w**

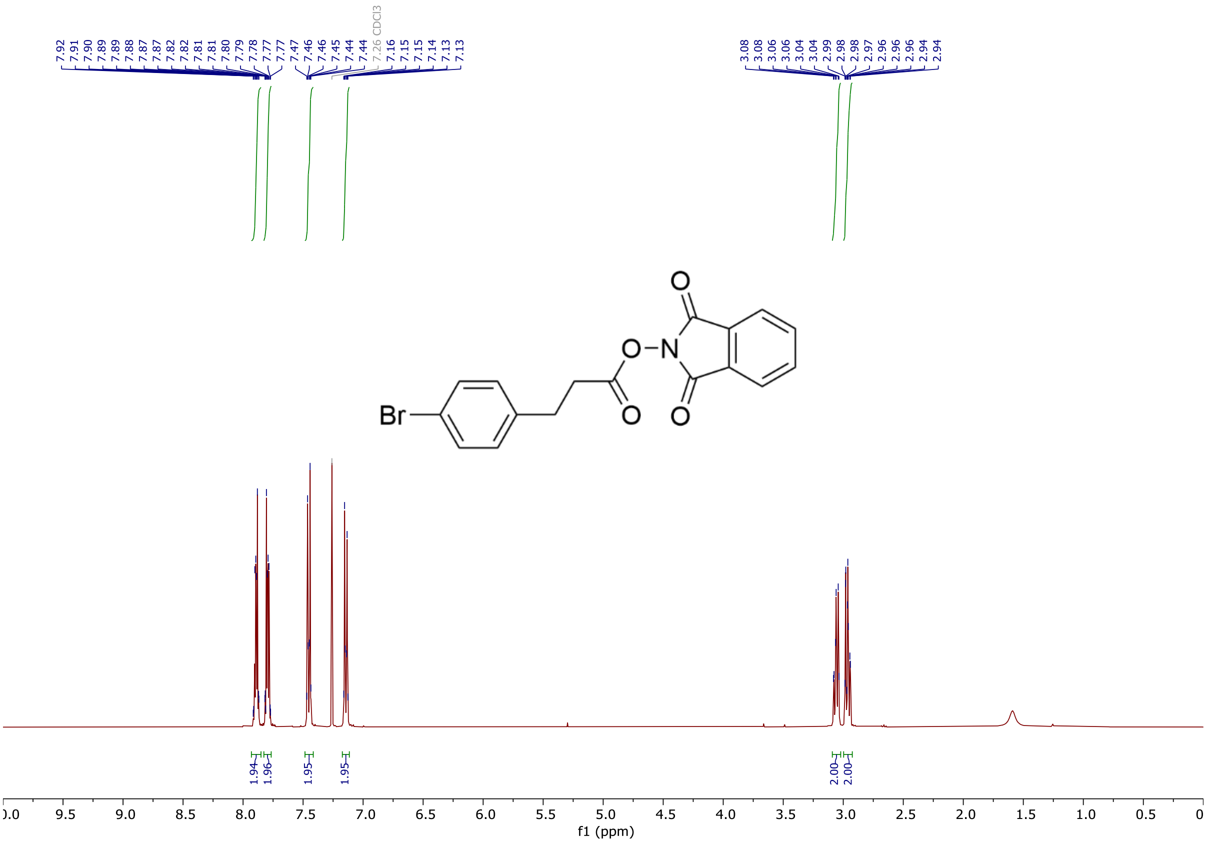

^{13}C NMR (101 MHz, $CDCl_3$): **4w**

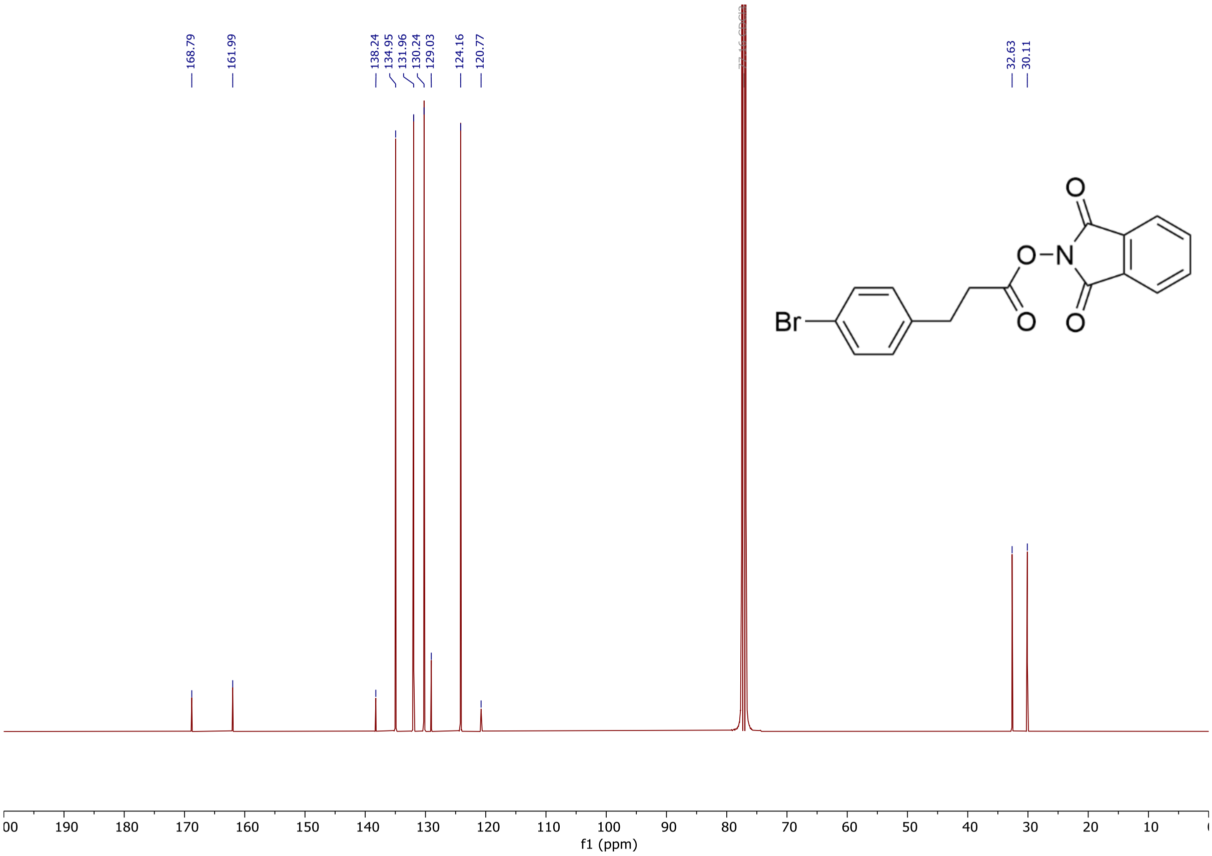

^{1}H NMR (400 MHz, $CDCl_3$): **4x**

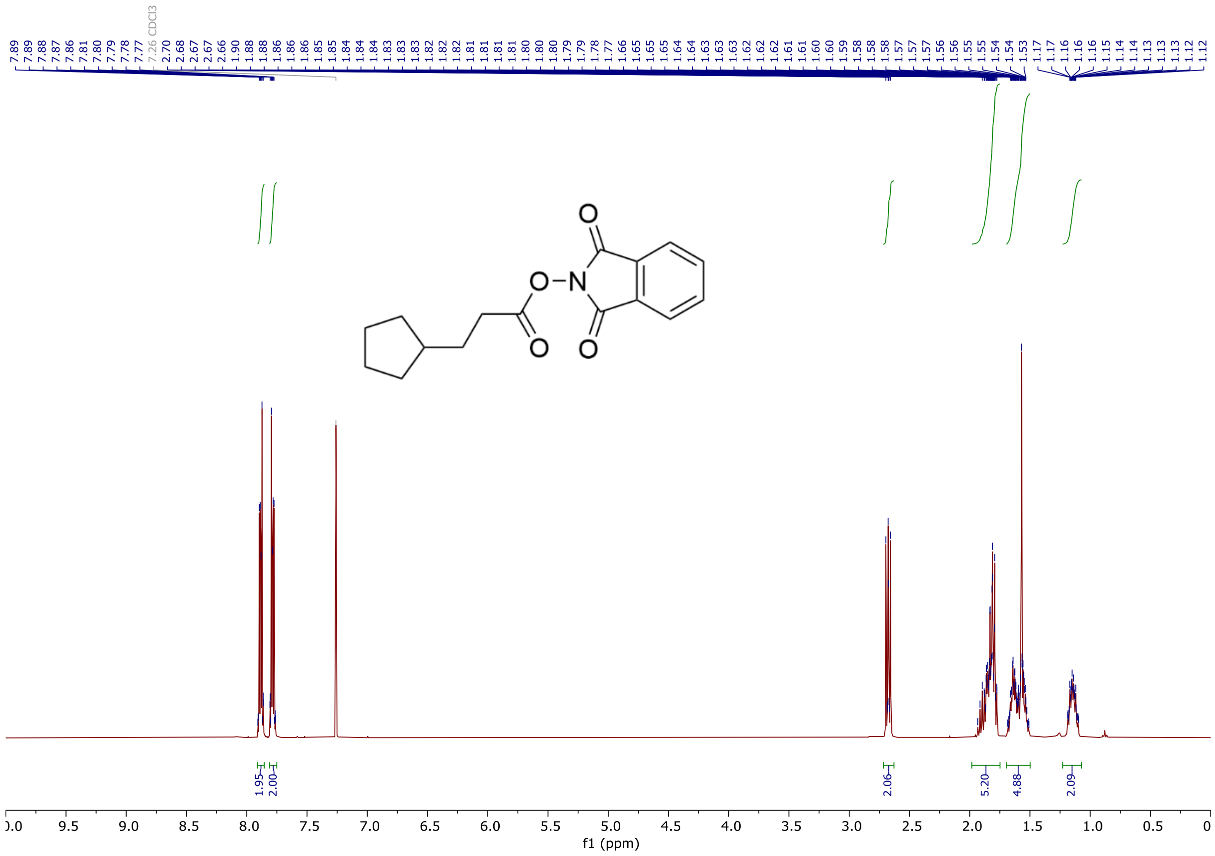

^{13}C NMR (101 MHz, $CDCl_3$): **4x**

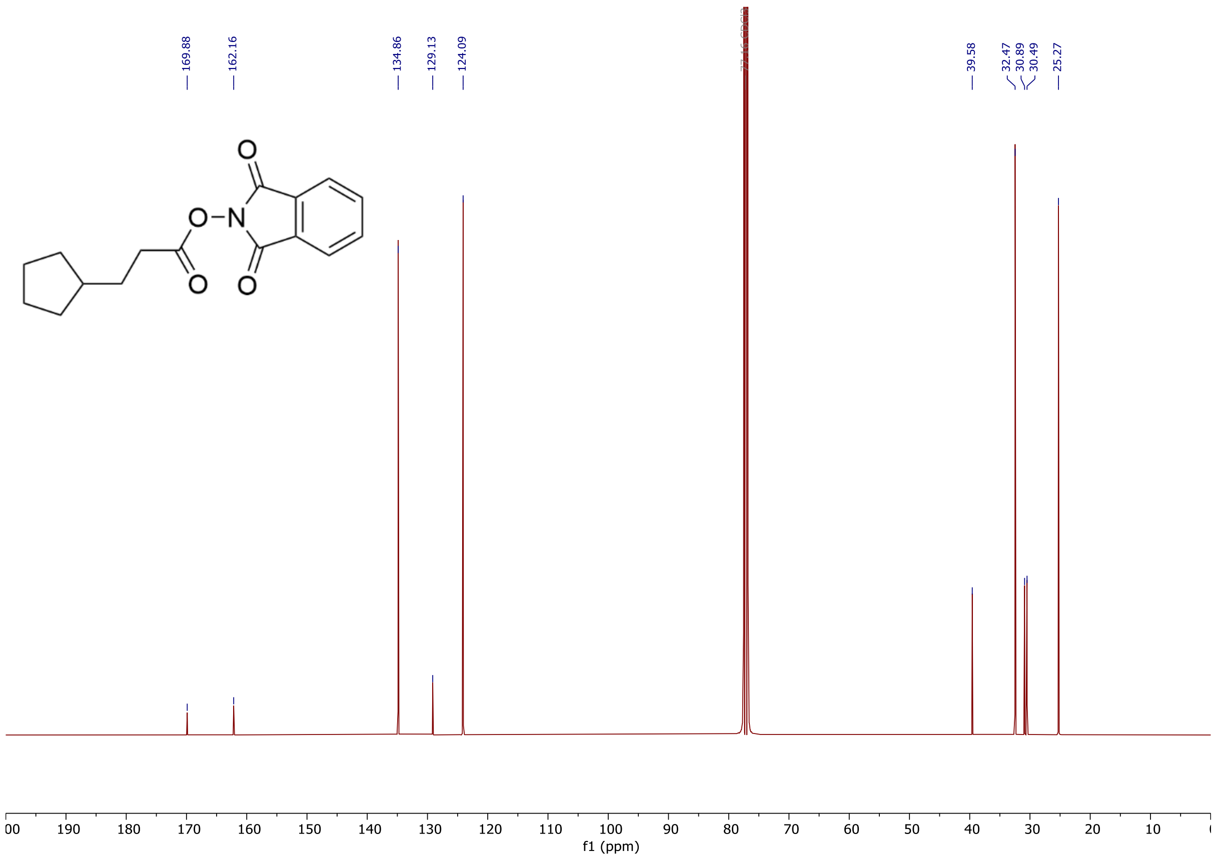

^{1}H NMR (500 MHz, CDCl$_3$): **4y**

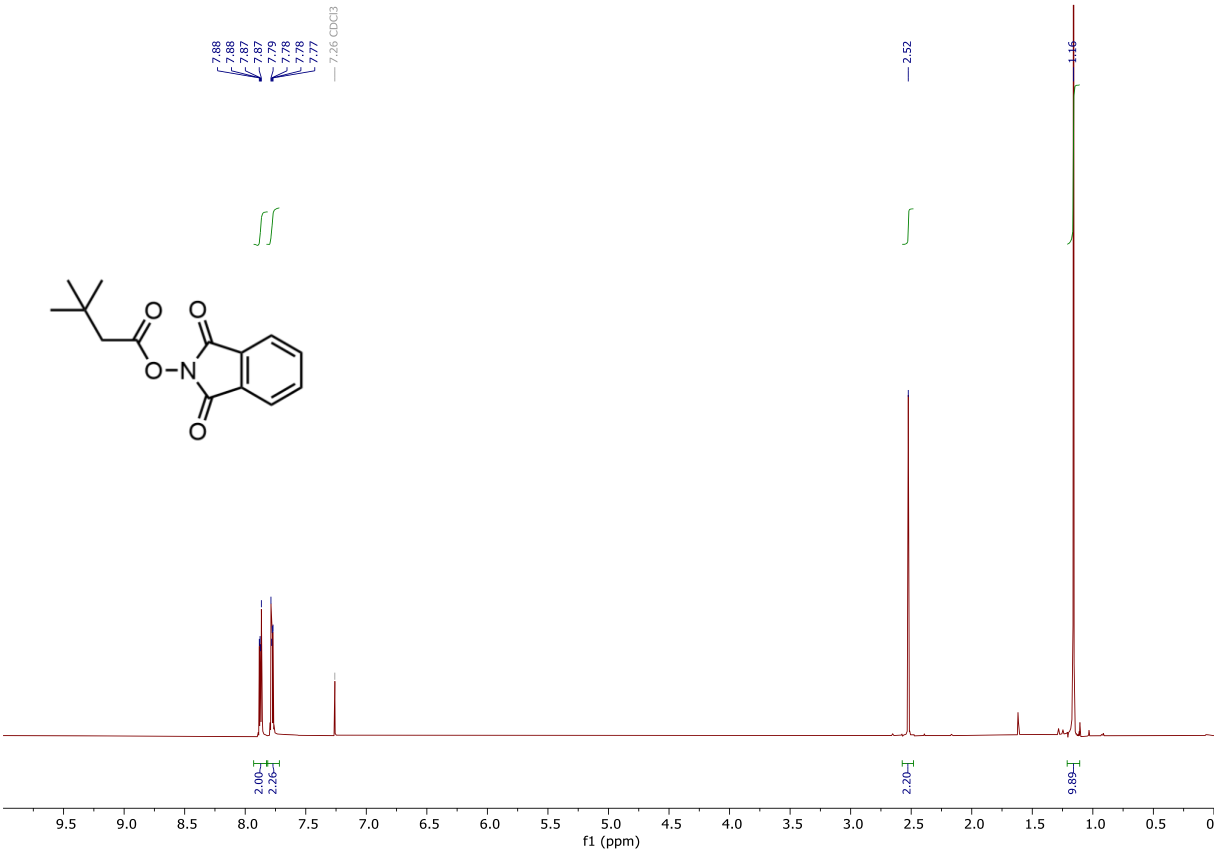

^{13}C NMR (126 MHz, CDCl$_3$): **4y**

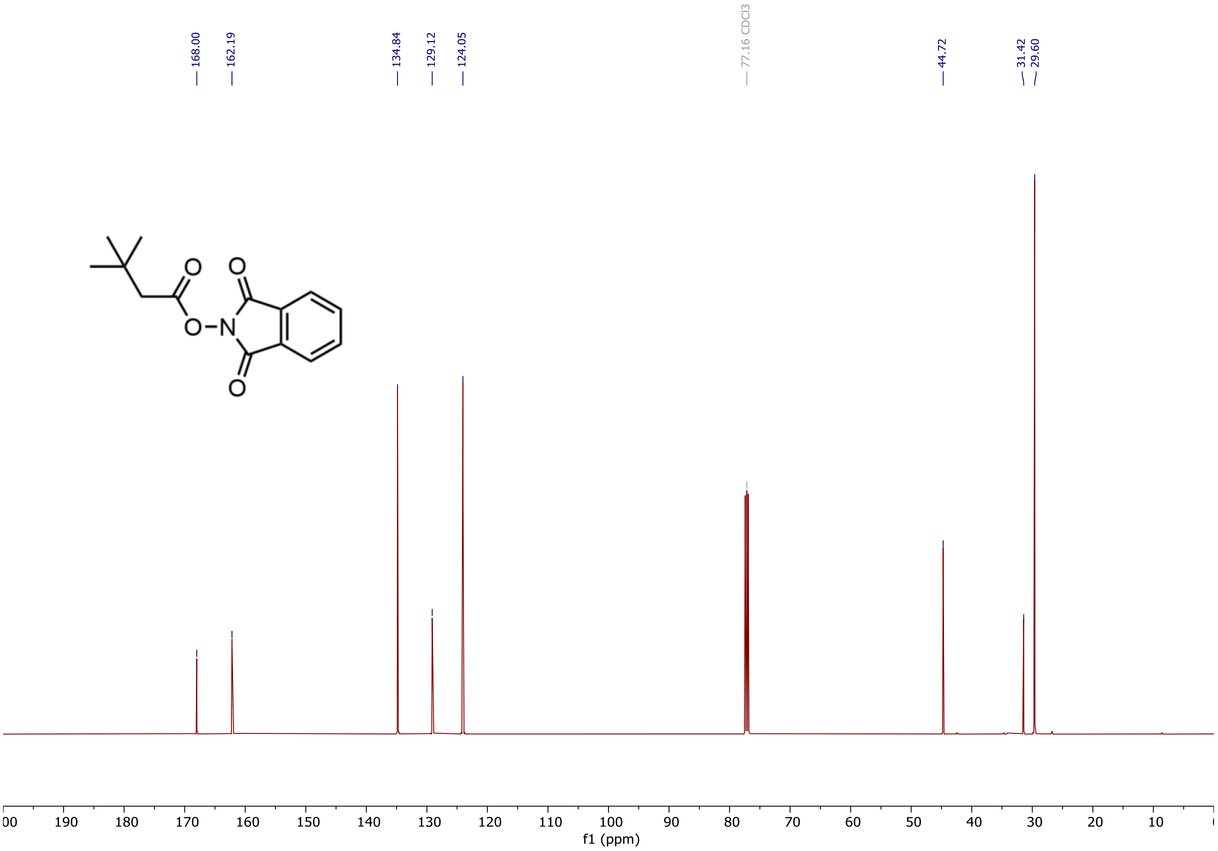

^{1}H NMR (400 MHz, $CDCl_3$): **4z**

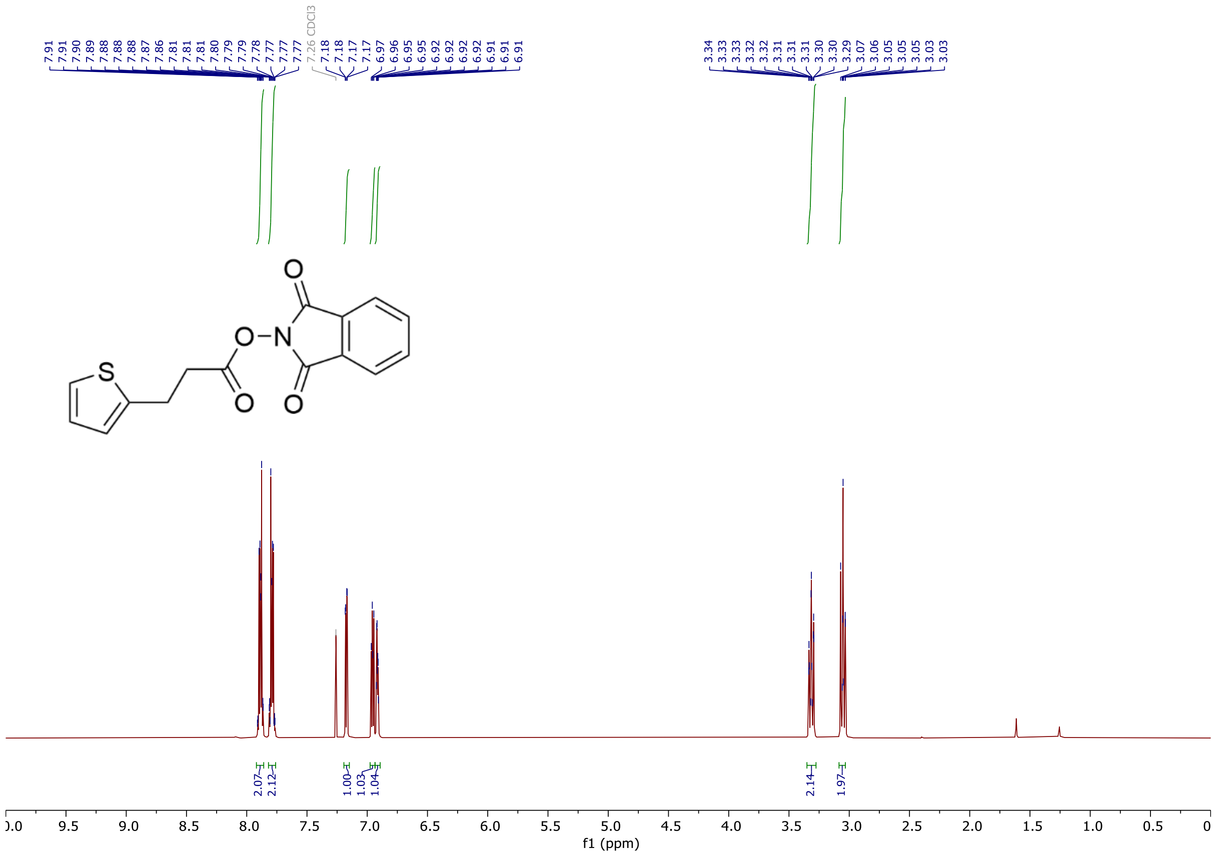

^{13}C NMR (101 MHz, $CDCl_3$): **4z**

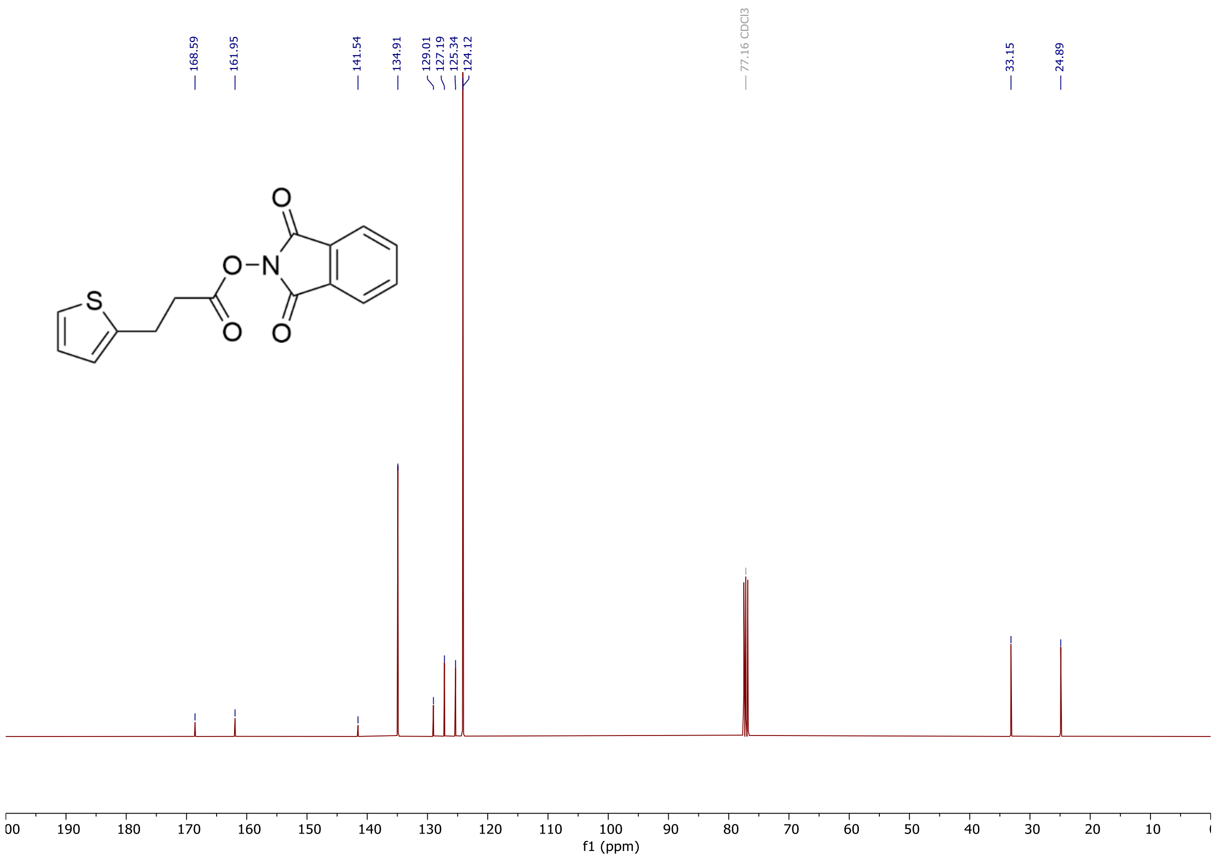

^{1}H NMR (400 MHz, $CDCl_3$): **4aa**

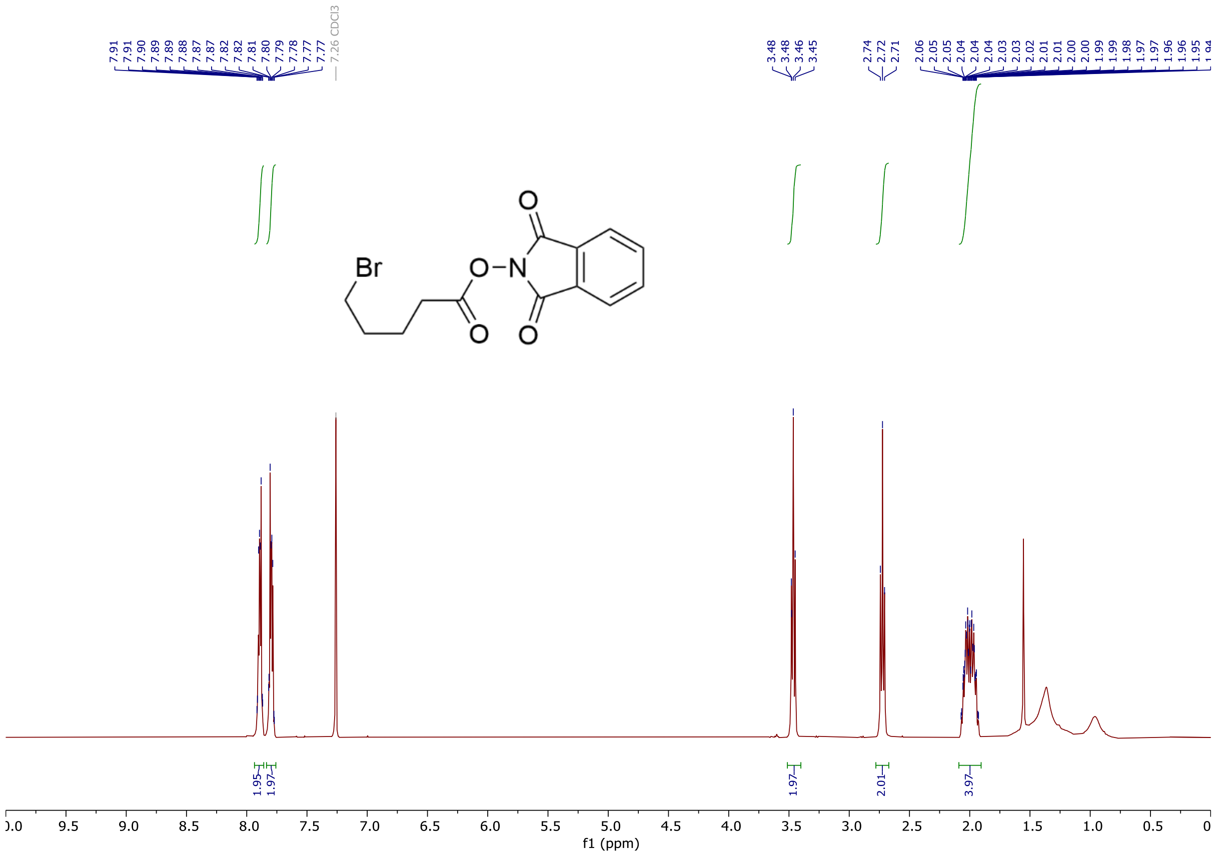

^{13}C NMR (101 MHz, $CDCl_3$): **4aa**

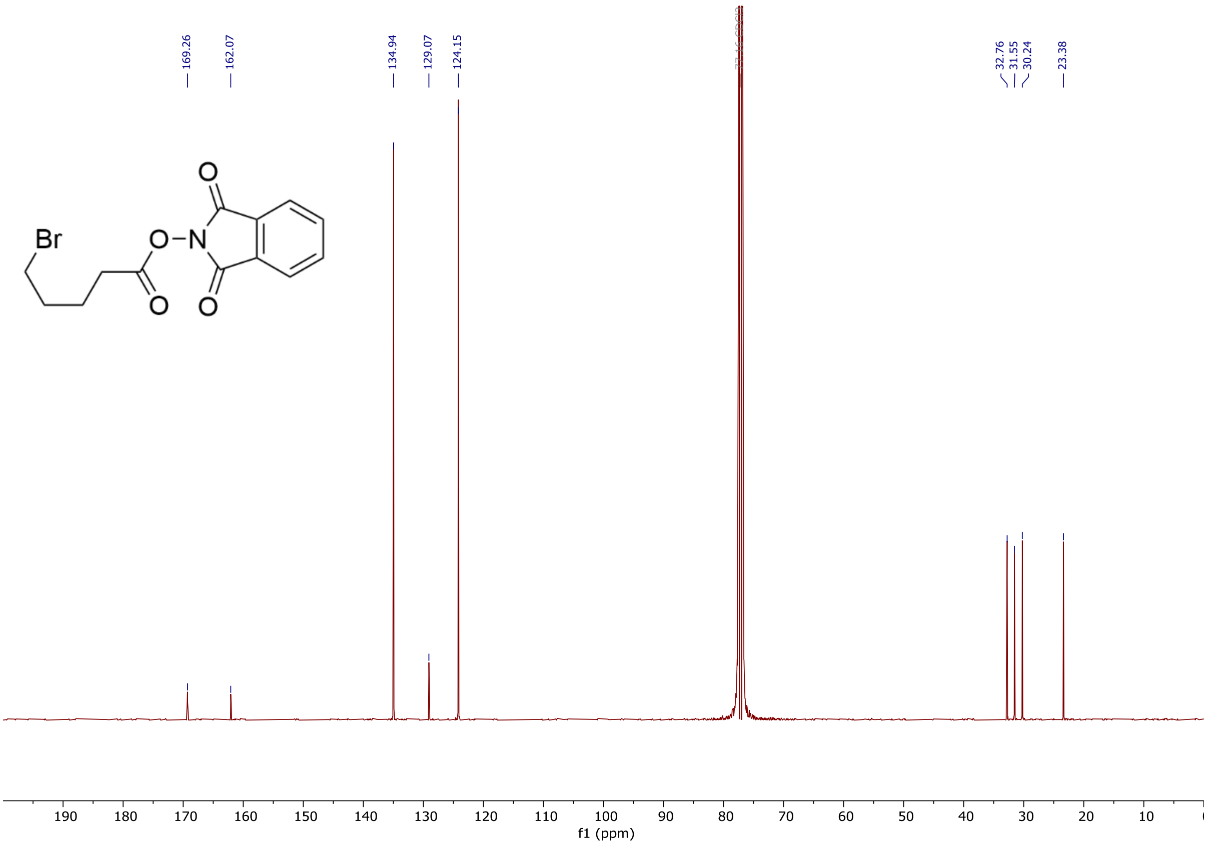

¹H NMR (400 MHz, $CDCl_3$): **4ab**

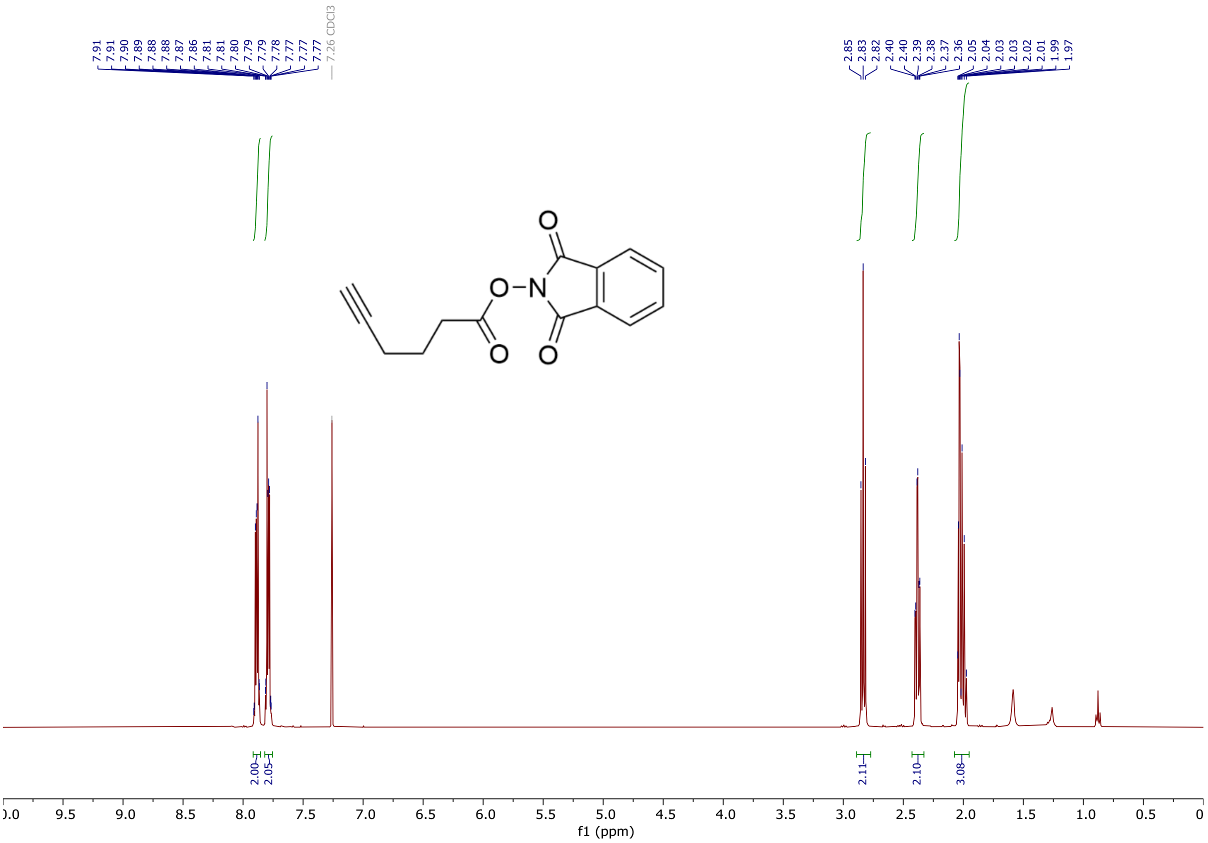

¹³C NMR (101 MHz, $CDCl_3$): **4ab**

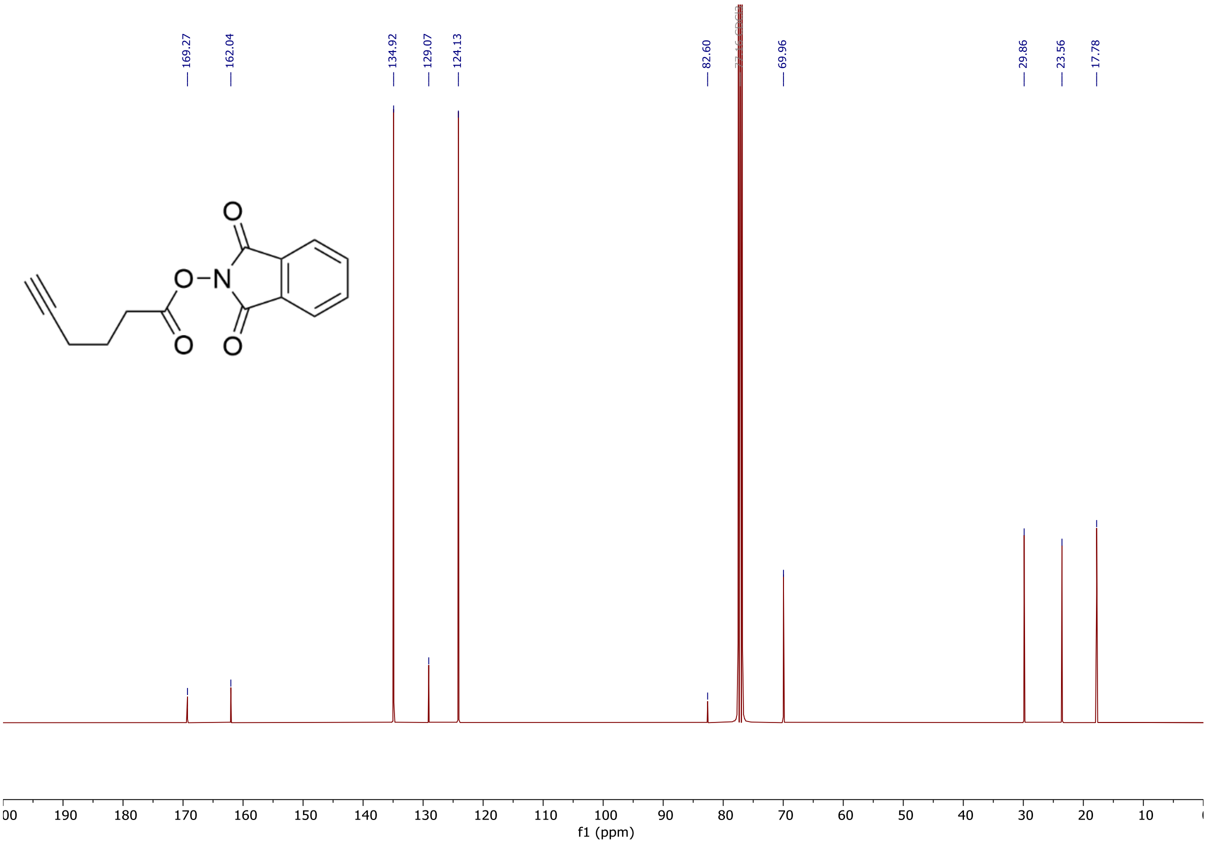

^{1}H NMR (400 MHz, $CDCl_3$): **4ac**

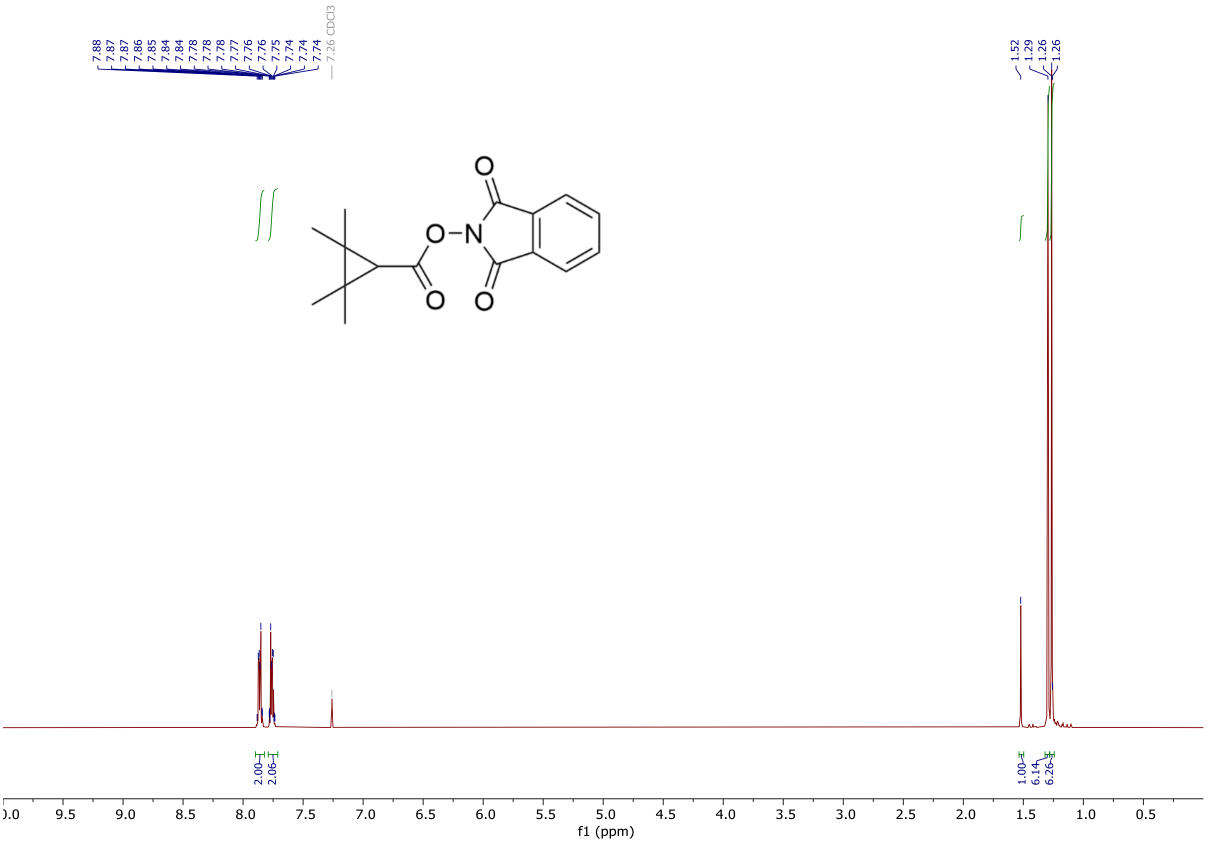

^{13}C NMR (101 MHz, $CDCl_3$): **4ac**

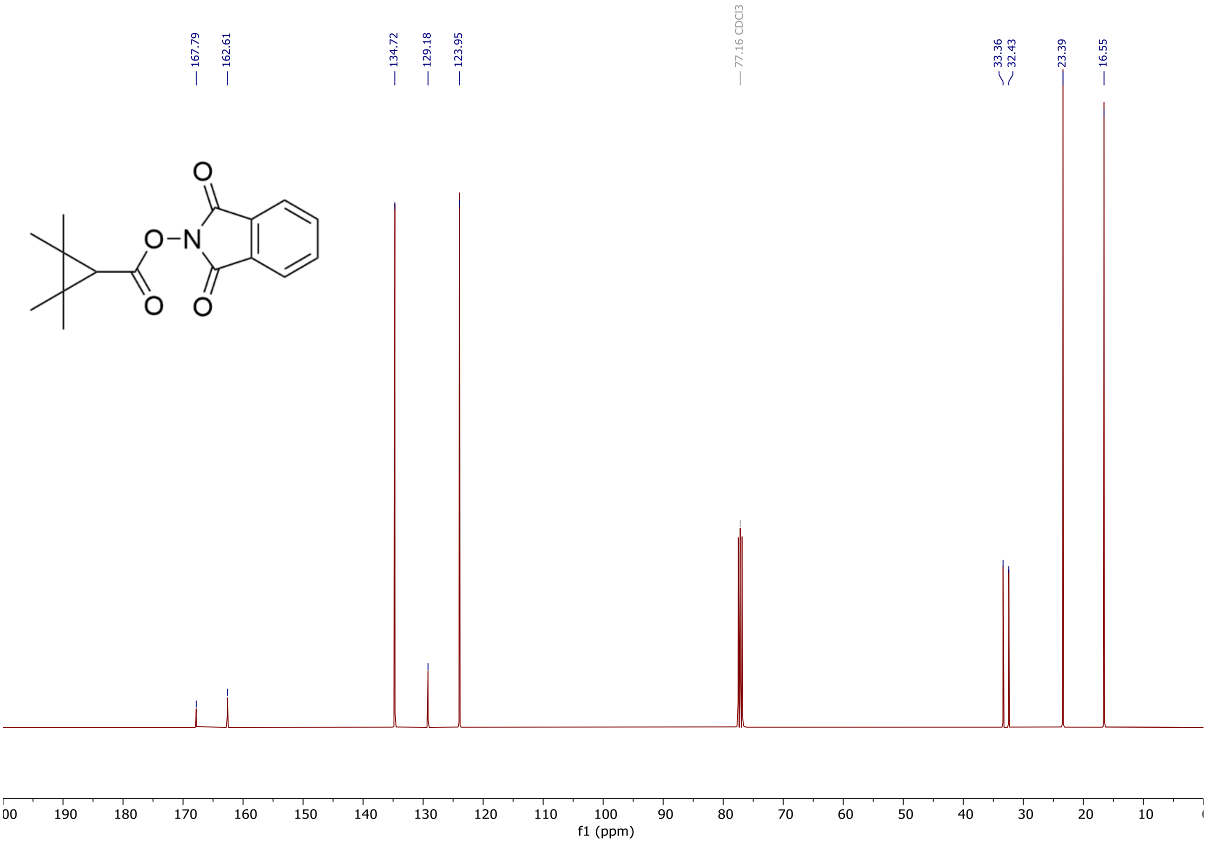

^{1}H NMR (400 MHz, $CDCl_3$): **4ad**

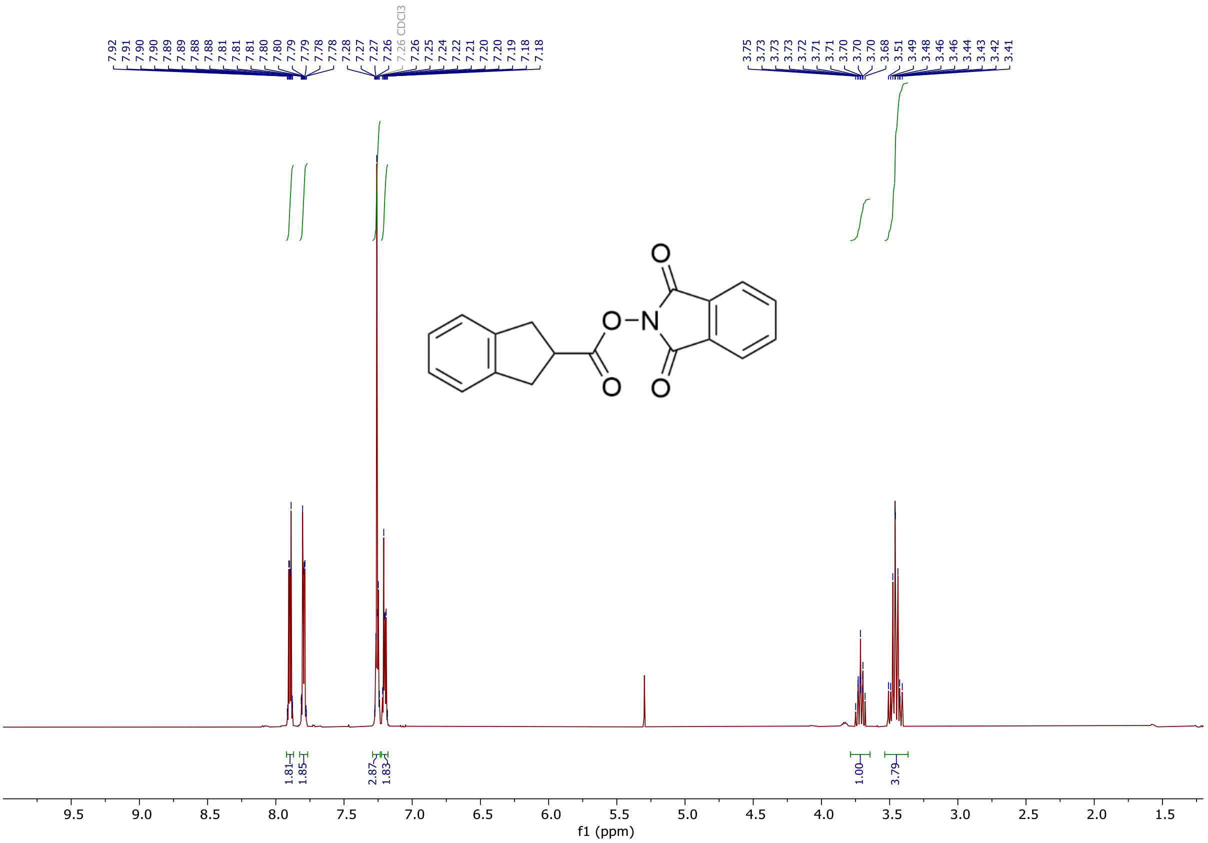

^{13}C NMR (101 MHz, $CDCl_3$): **4ad**

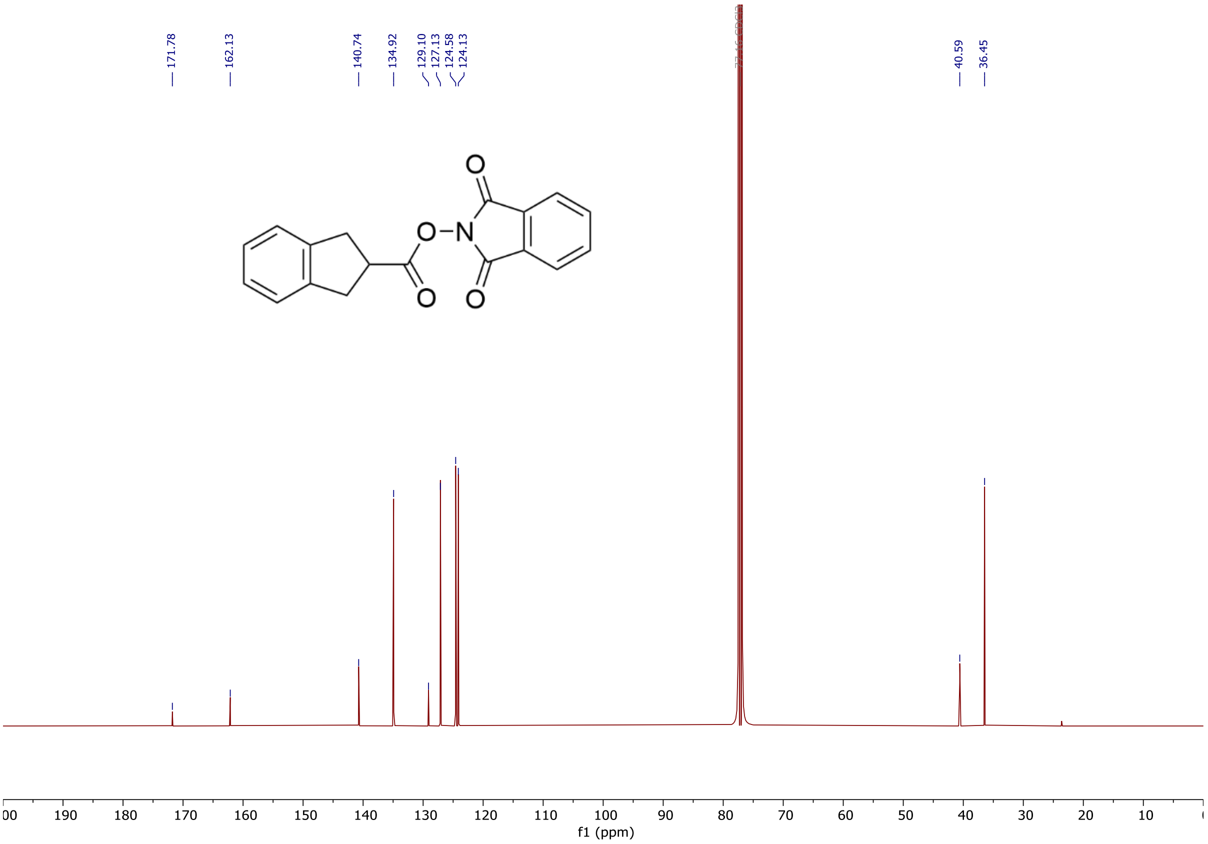

^{1}H NMR (400 MHz, $CDCl_3$): **4ae**

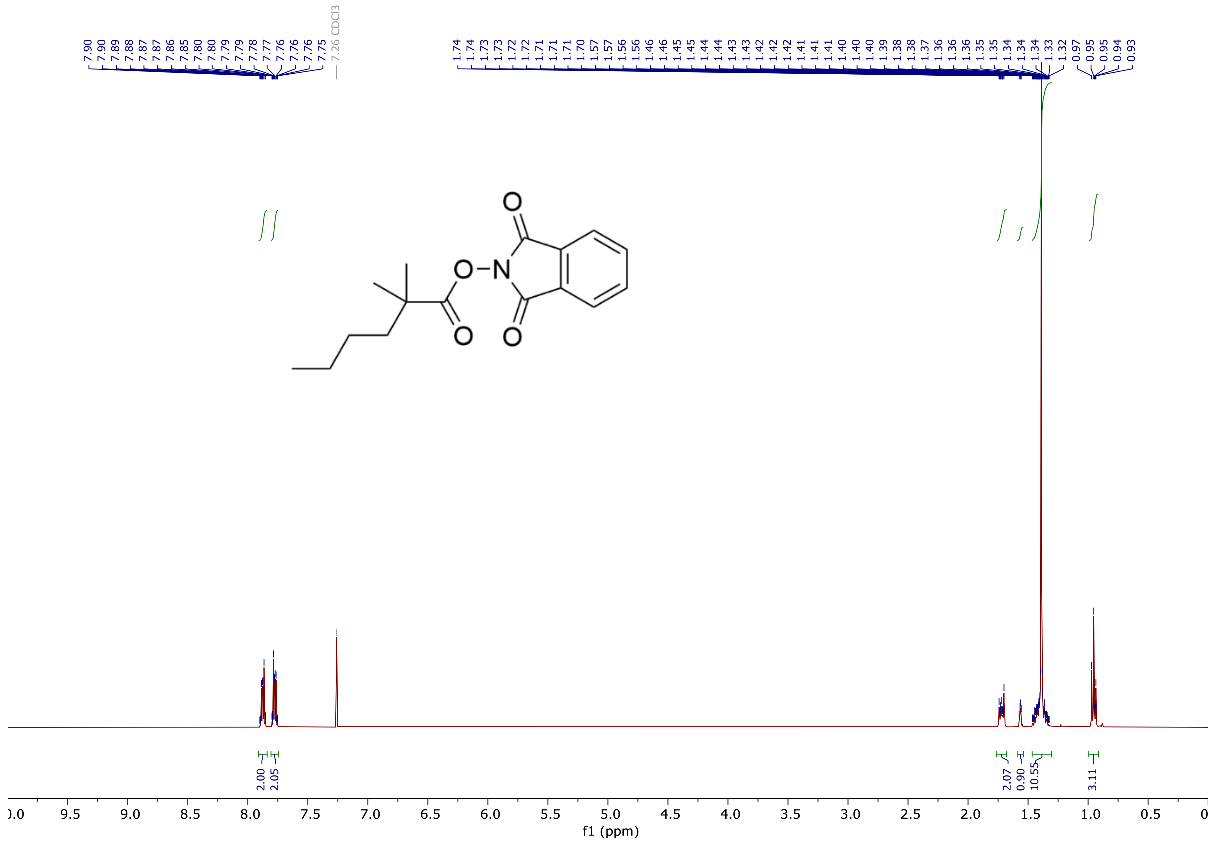

^{13}C NMR (101 MHz, $CDCl_3$): **4ae**

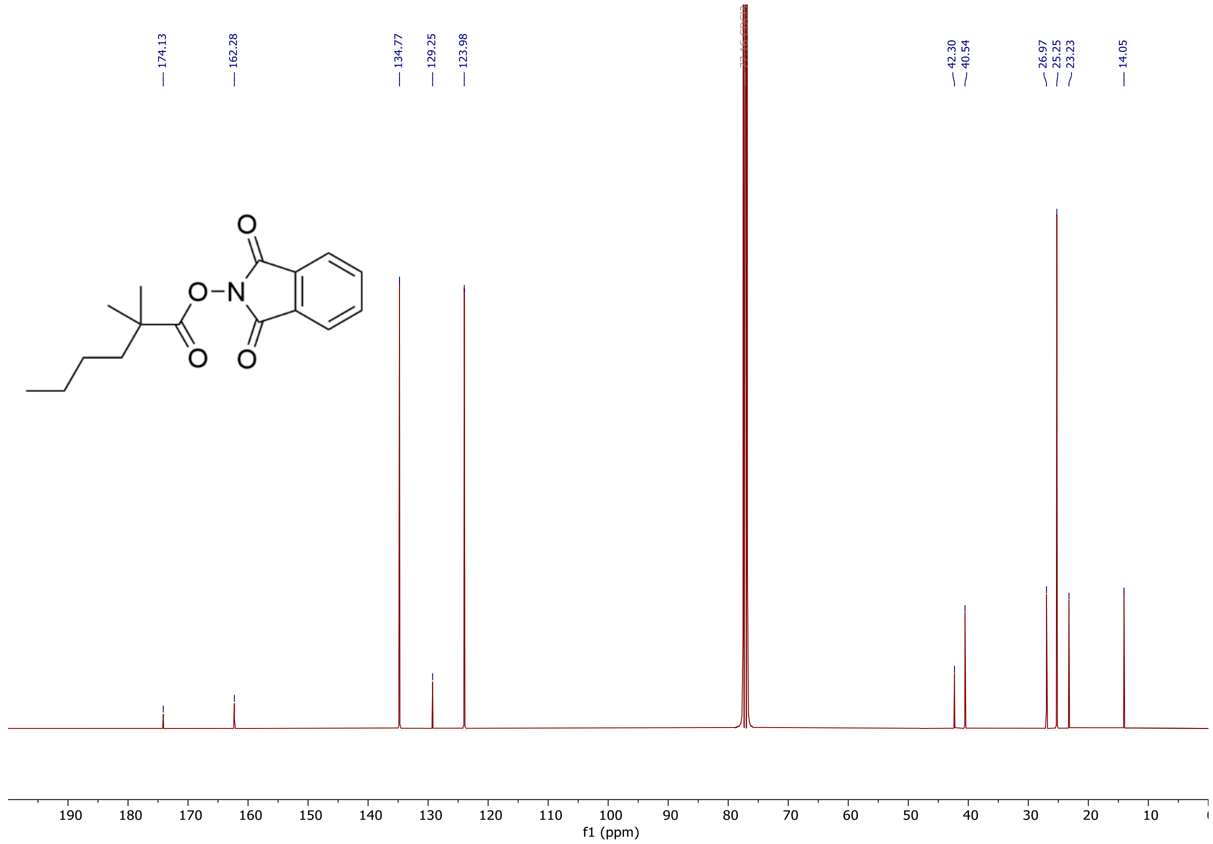

^{1}H NMR (400 MHz, $CDCl_3$): **4af**

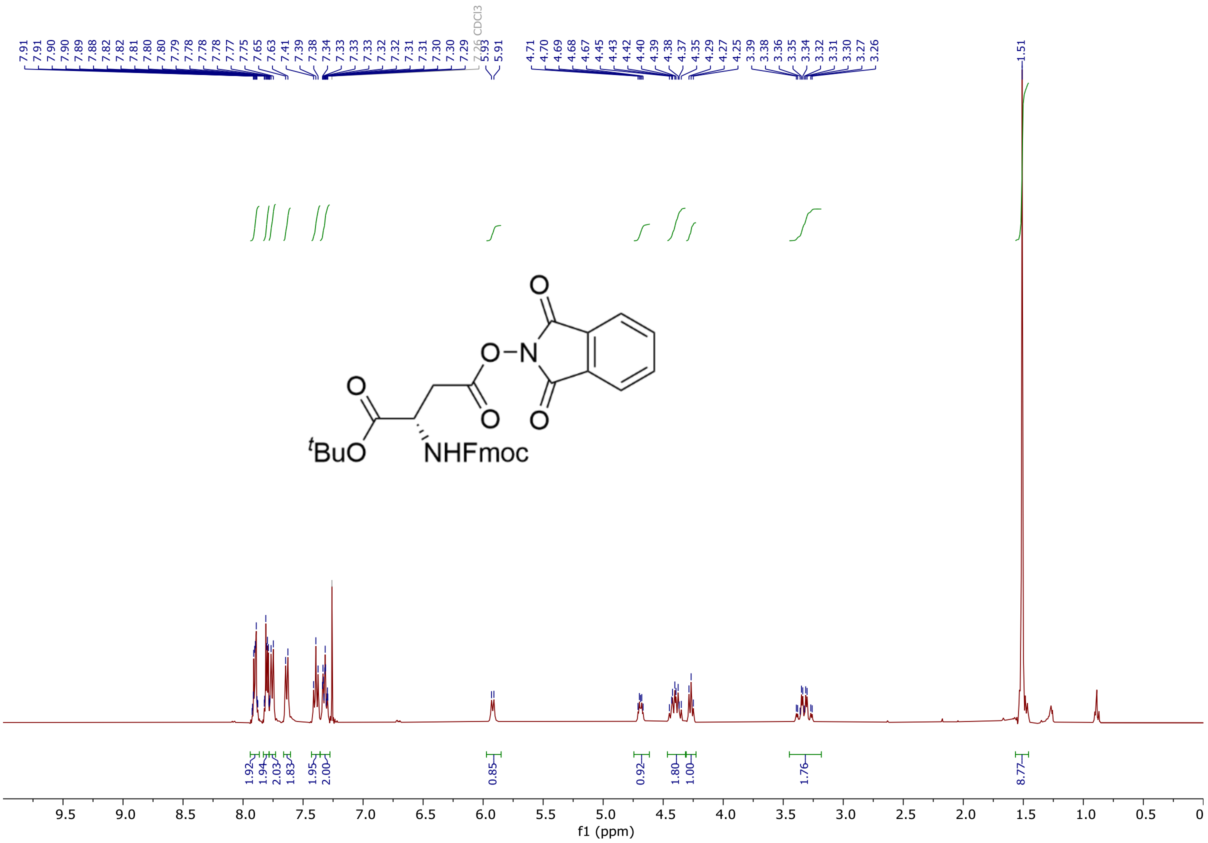

^{13}C NMR (101 MHz, $CDCl_3$): **4af**

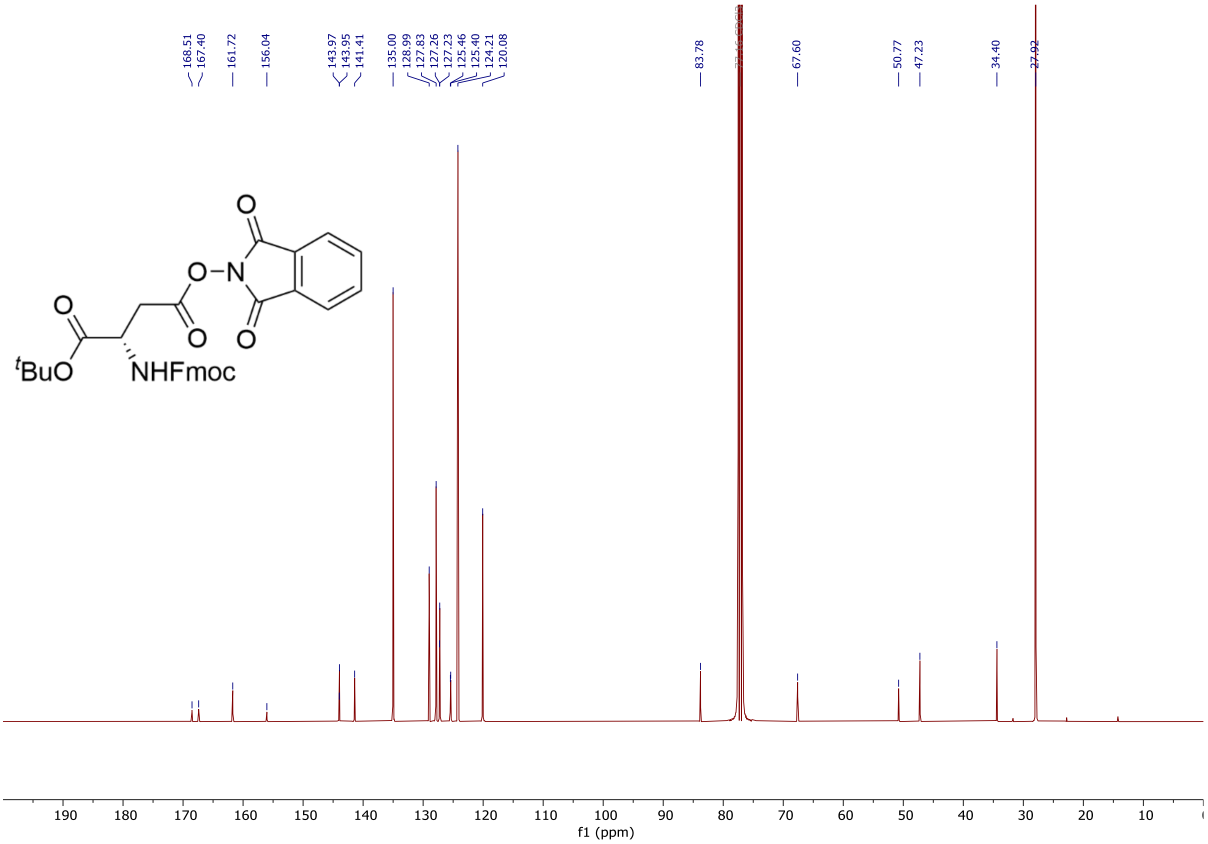

¹H NMR (400 MHz, CDCl₃): **4ag**

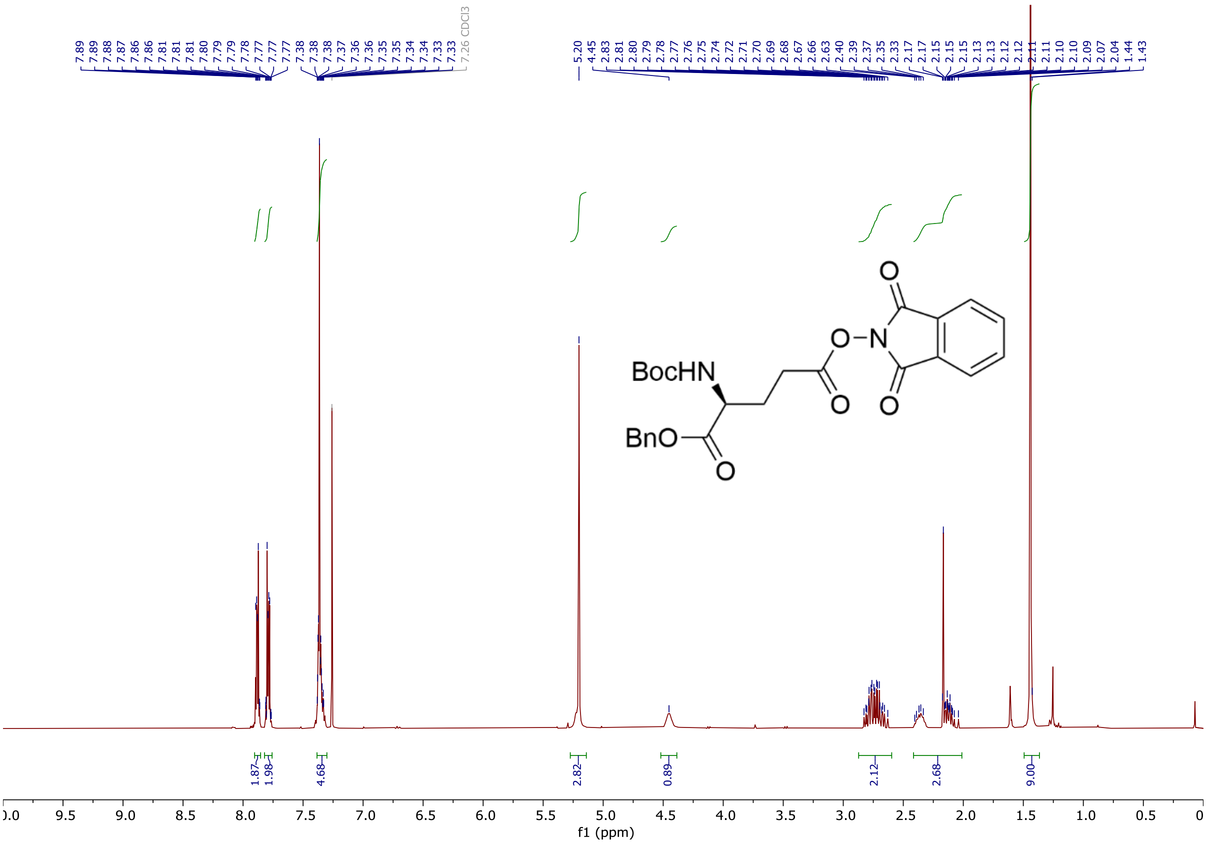

¹³C NMR (101 MHz, CDCl₃): **4ag**

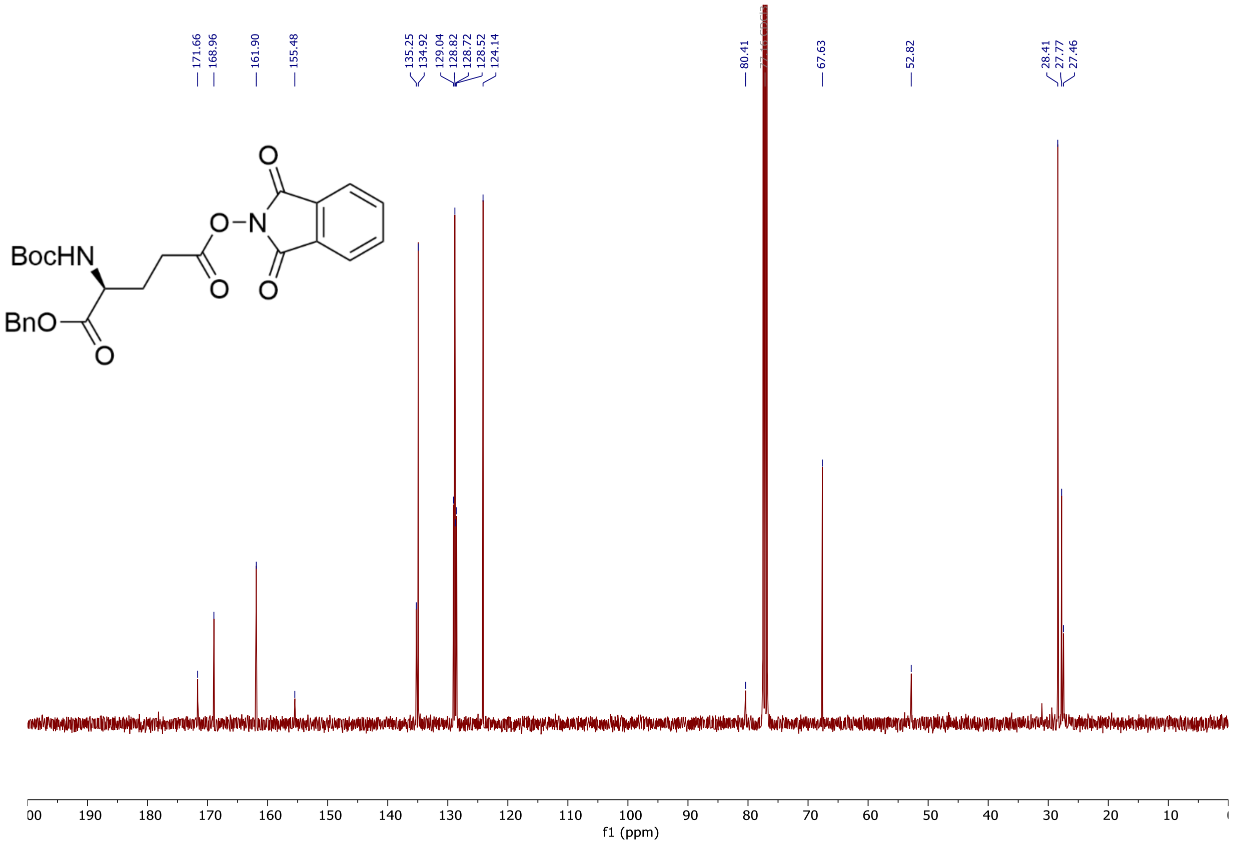

^{1}H NMR (400 MHz, $CDCl_3$): **4ah**

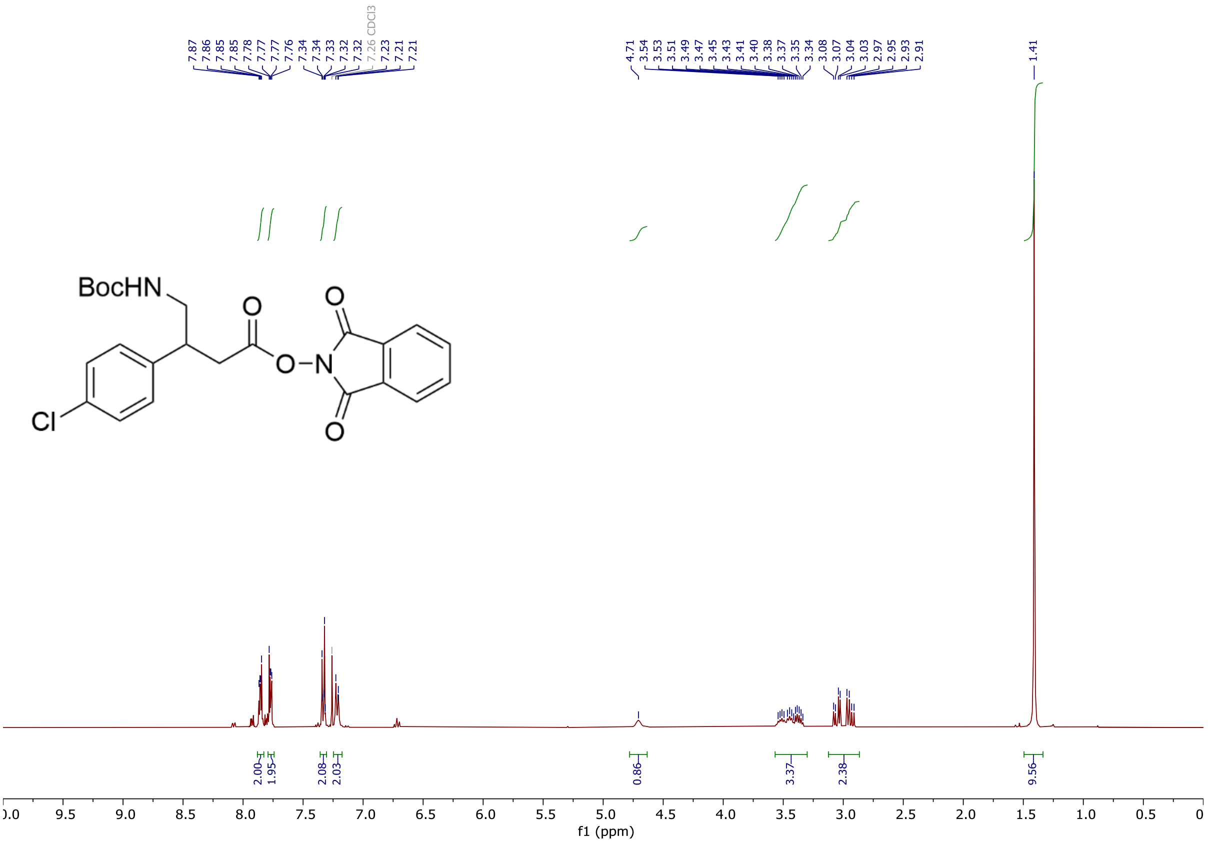

^{13}C NMR (101 MHz, $CDCl_3$): **4ah**

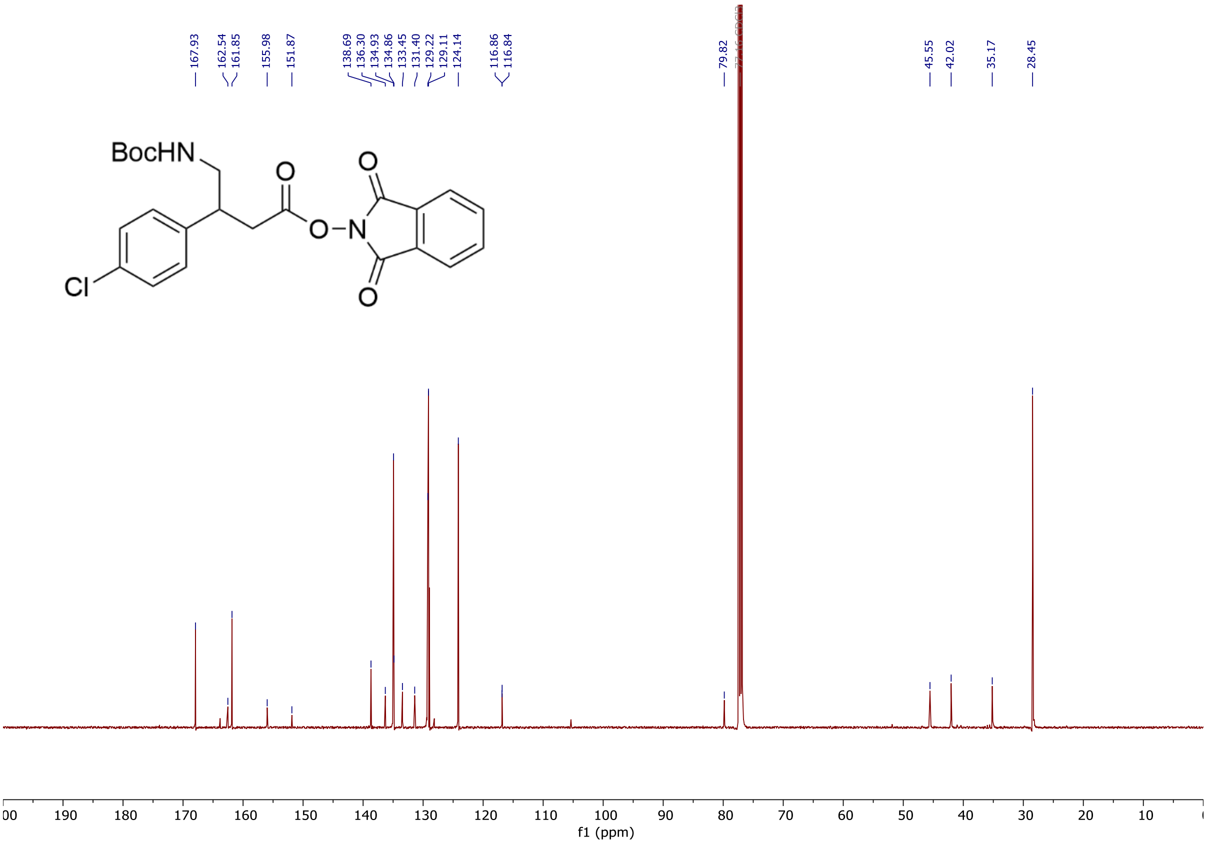

^{1}H NMR (400 MHz, $CDCl_3$): **4ai**

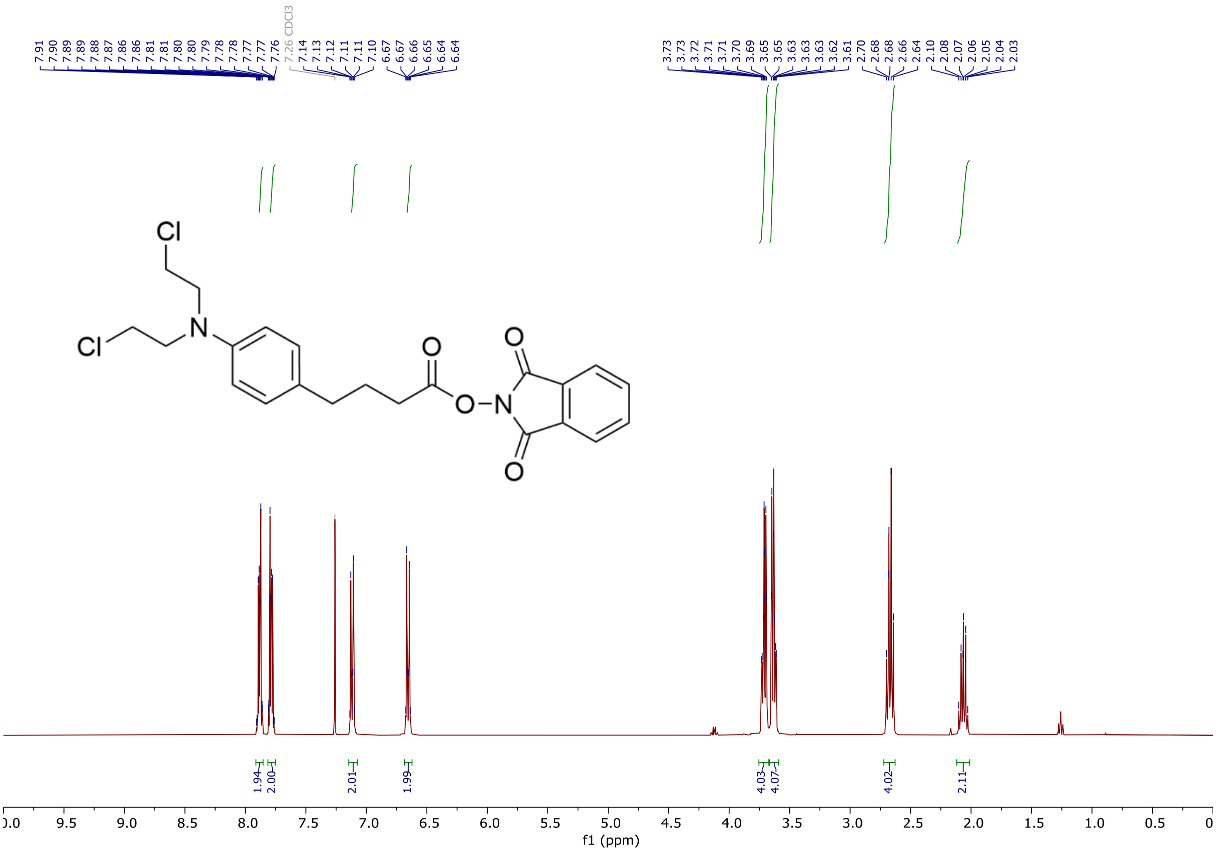

^{13}C NMR (101 MHz, $CDCl_3$): **4ai**

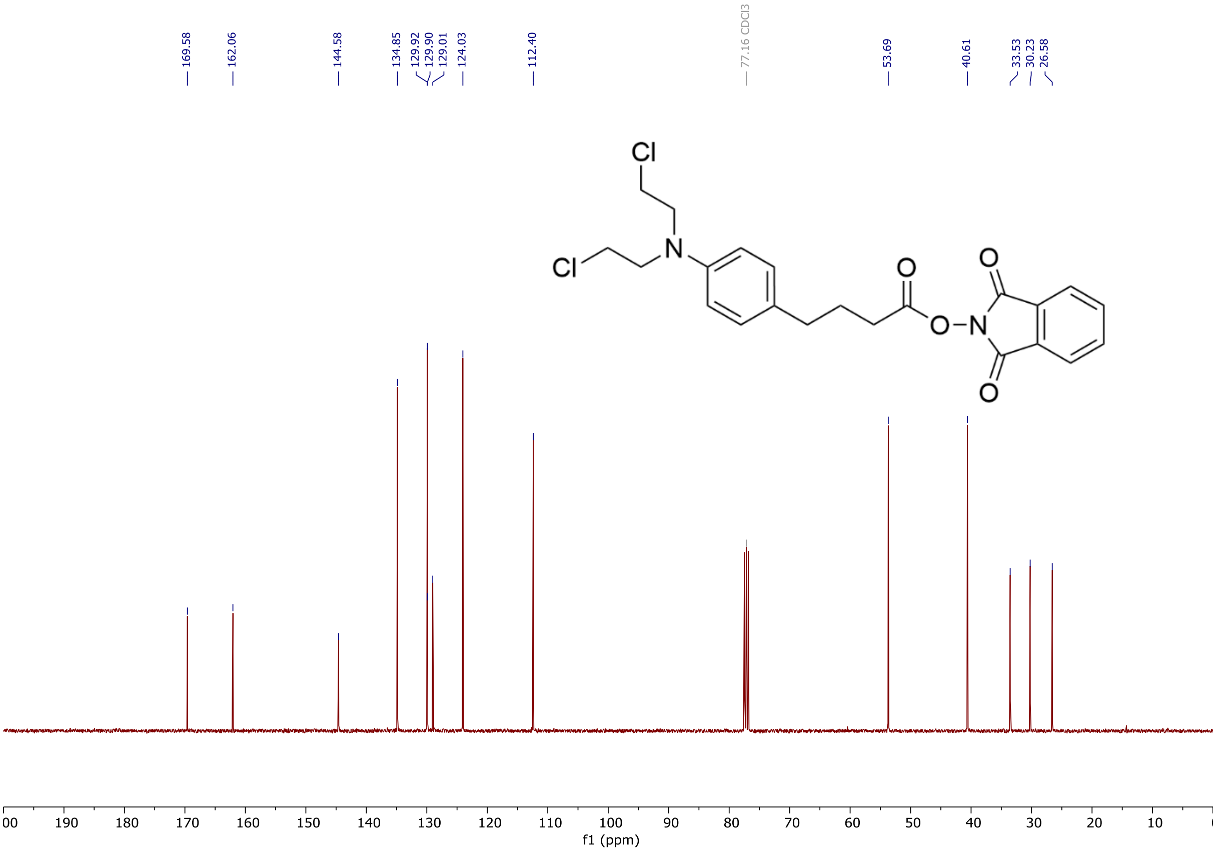

^{1}H NMR (400 MHz, CDCl$_3$): **4aj**

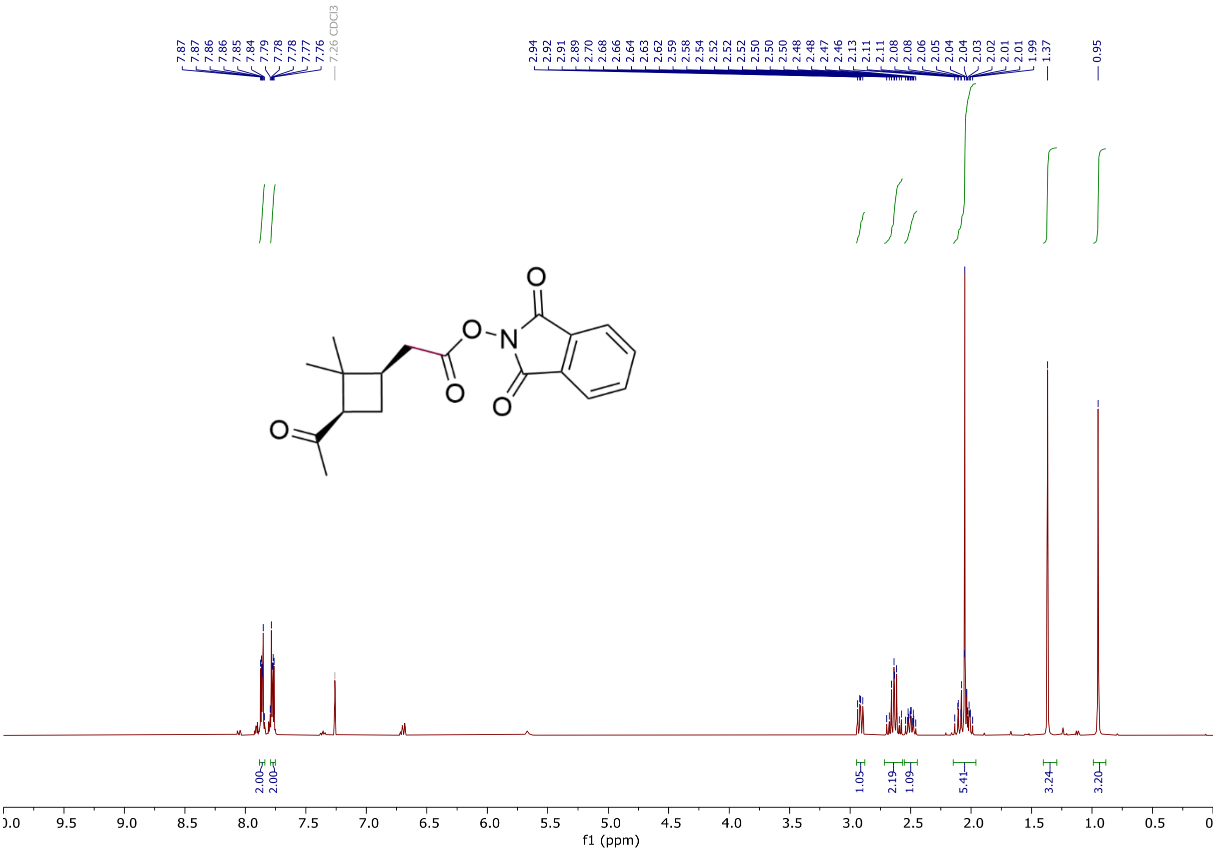

^{13}C NMR (101 MHz, CDCl$_3$): **4aj**

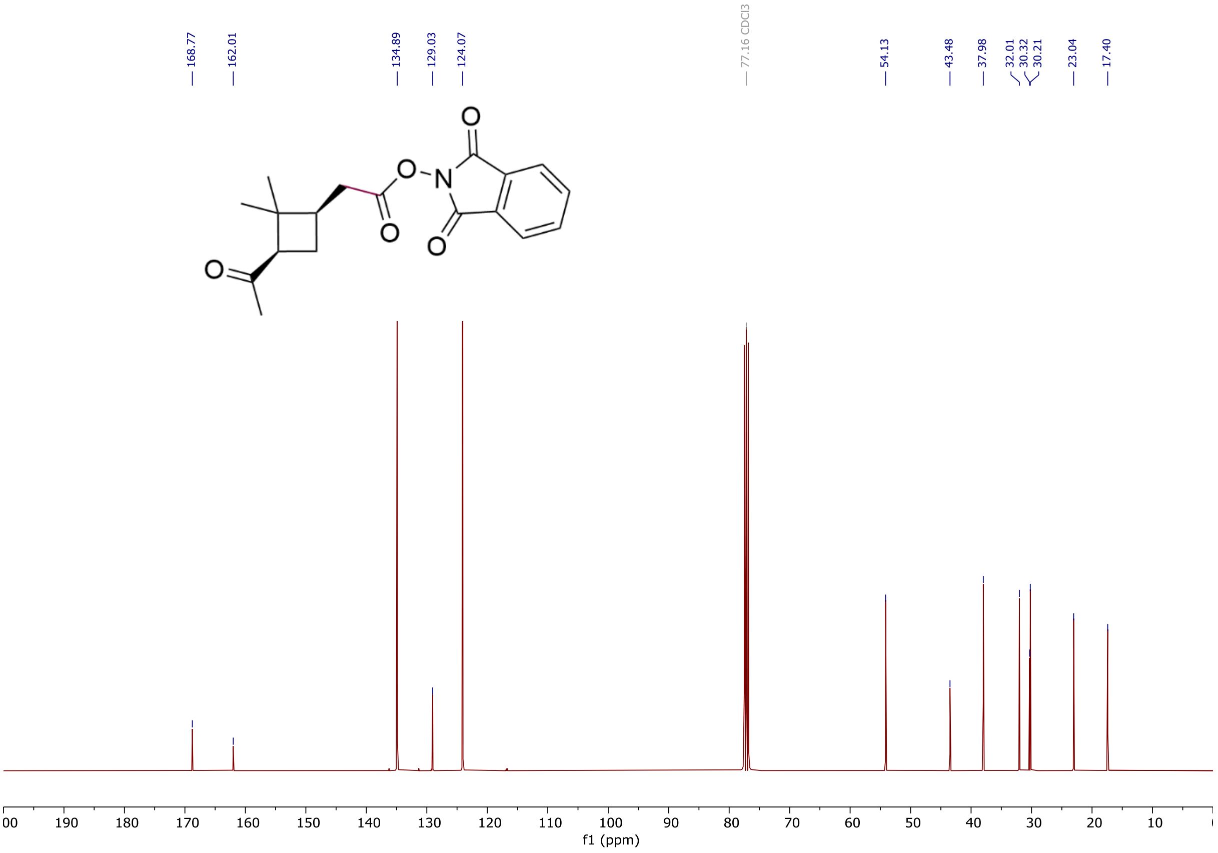

^{1}H NMR (400 MHz, $CDCl_3$): **4ak**

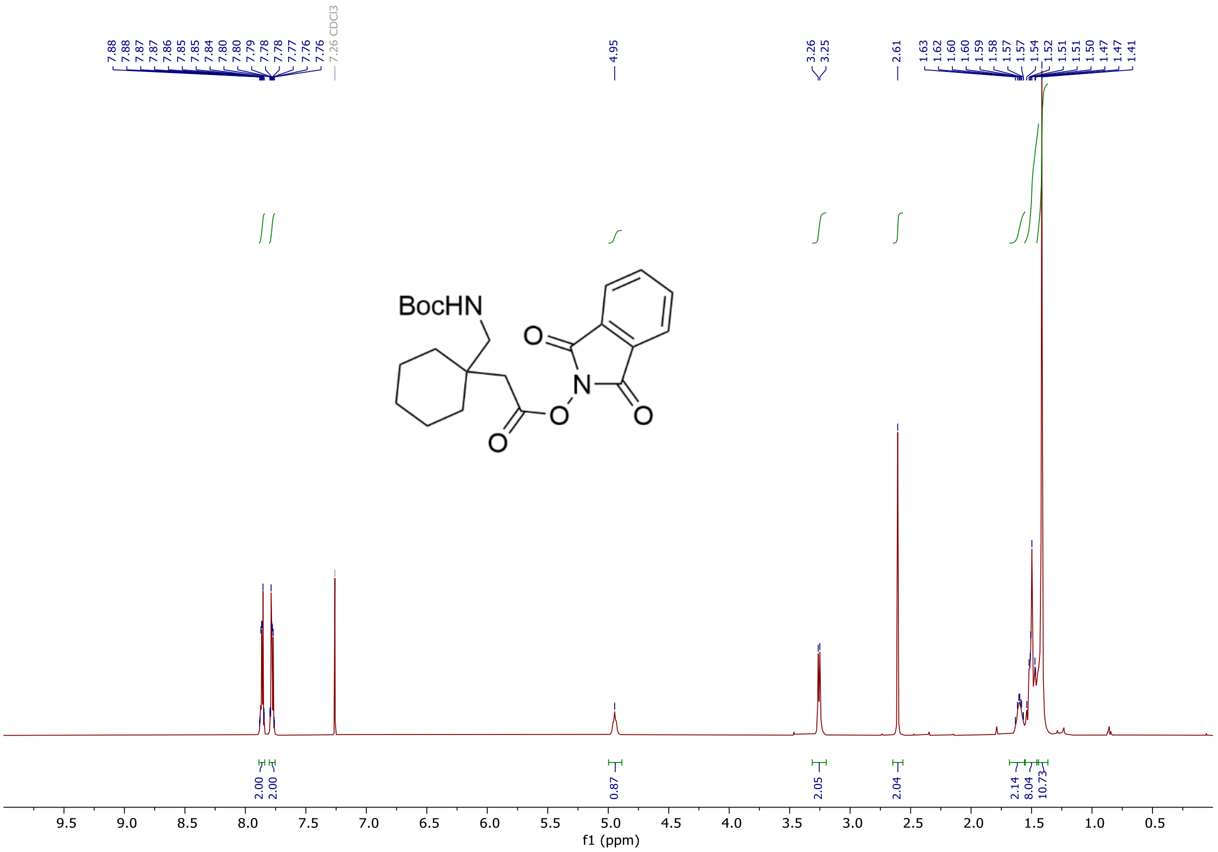

^{13}C NMR (101 MHz, $CDCl_3$): **4ak**

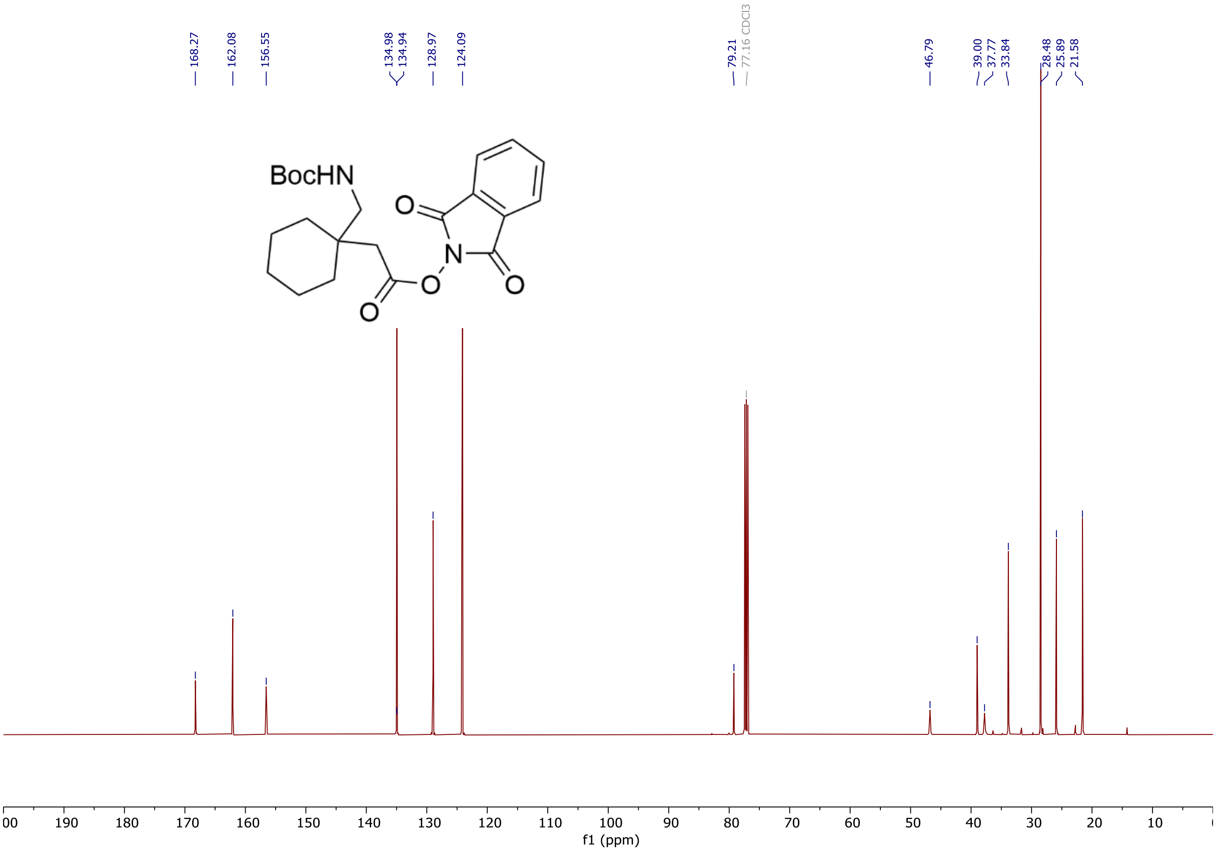

^{1}H NMR (400 MHz, CDCl3): **4al**

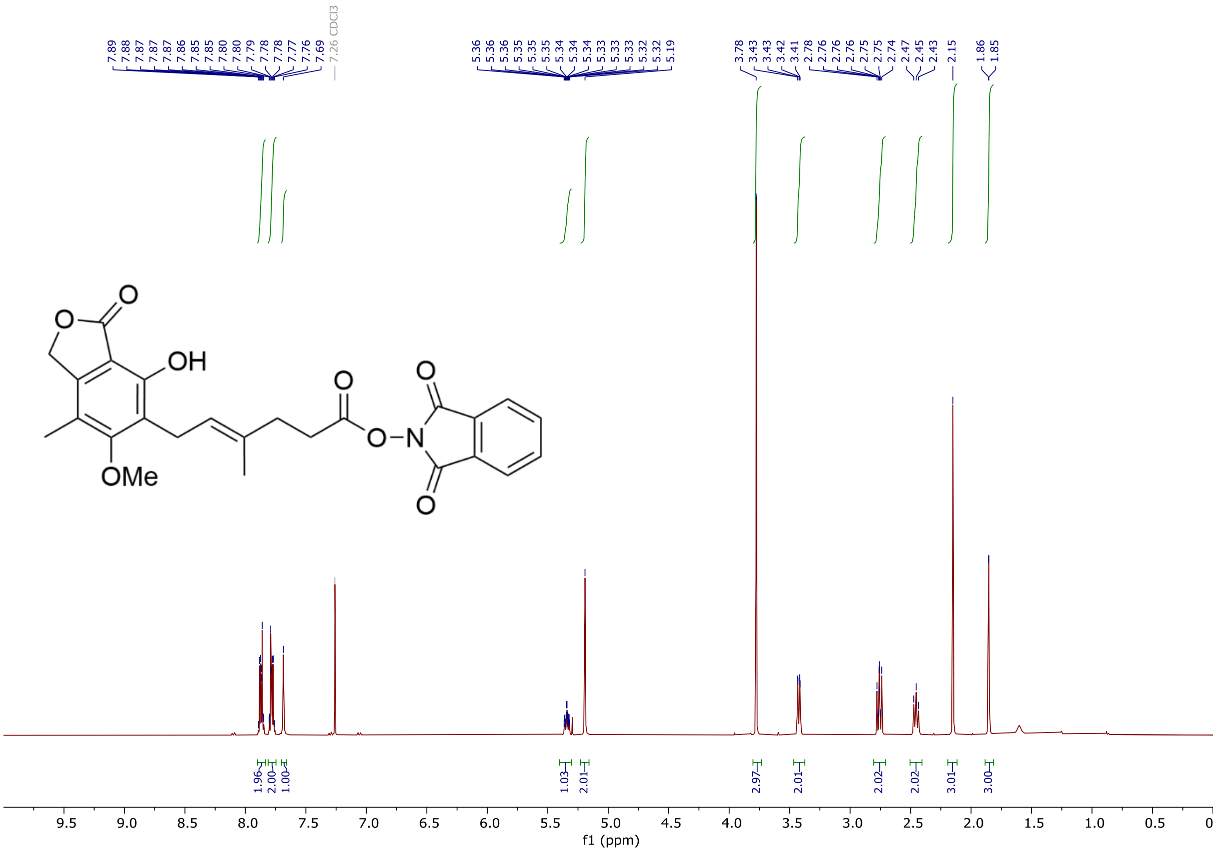

^{13}C NMR (101 MHz, CDCl3): **4al**

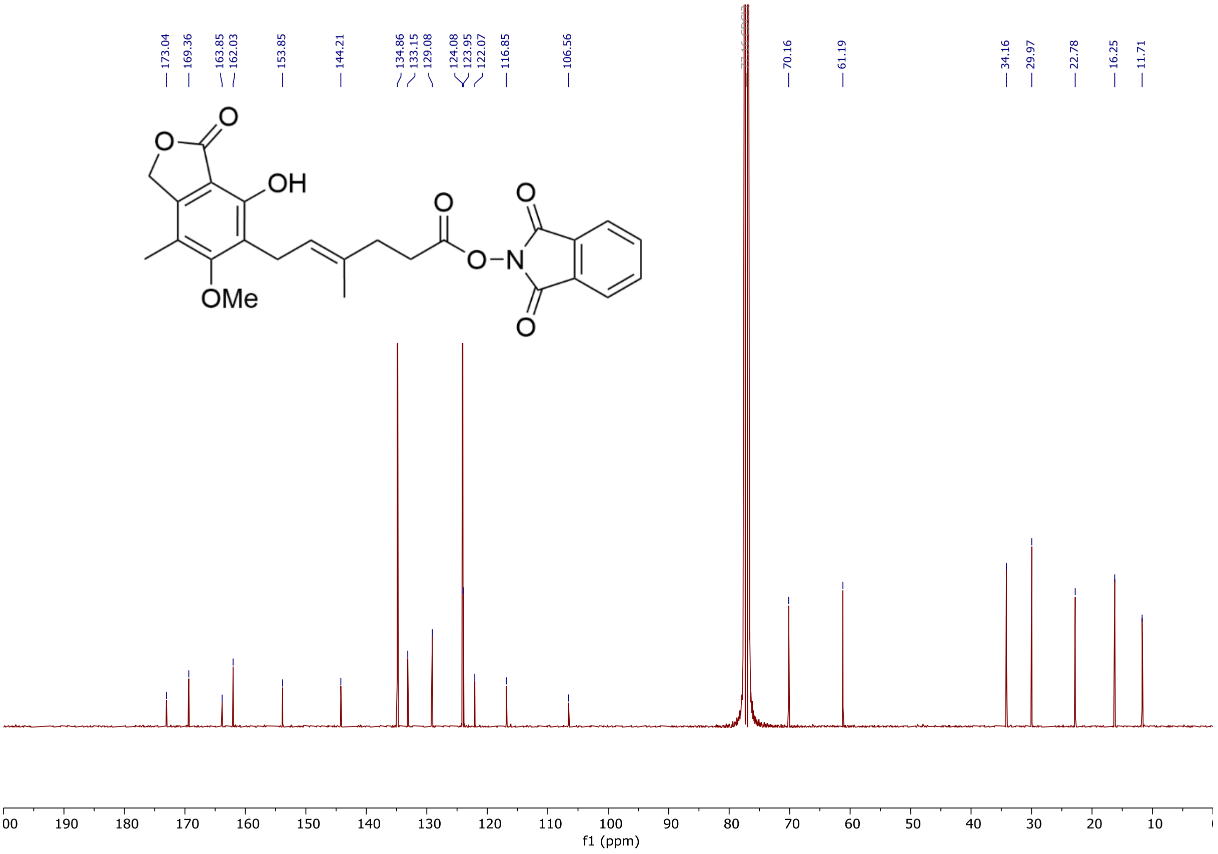

^{1}H NMR (400 MHz, $CDCl_3$): **4am**

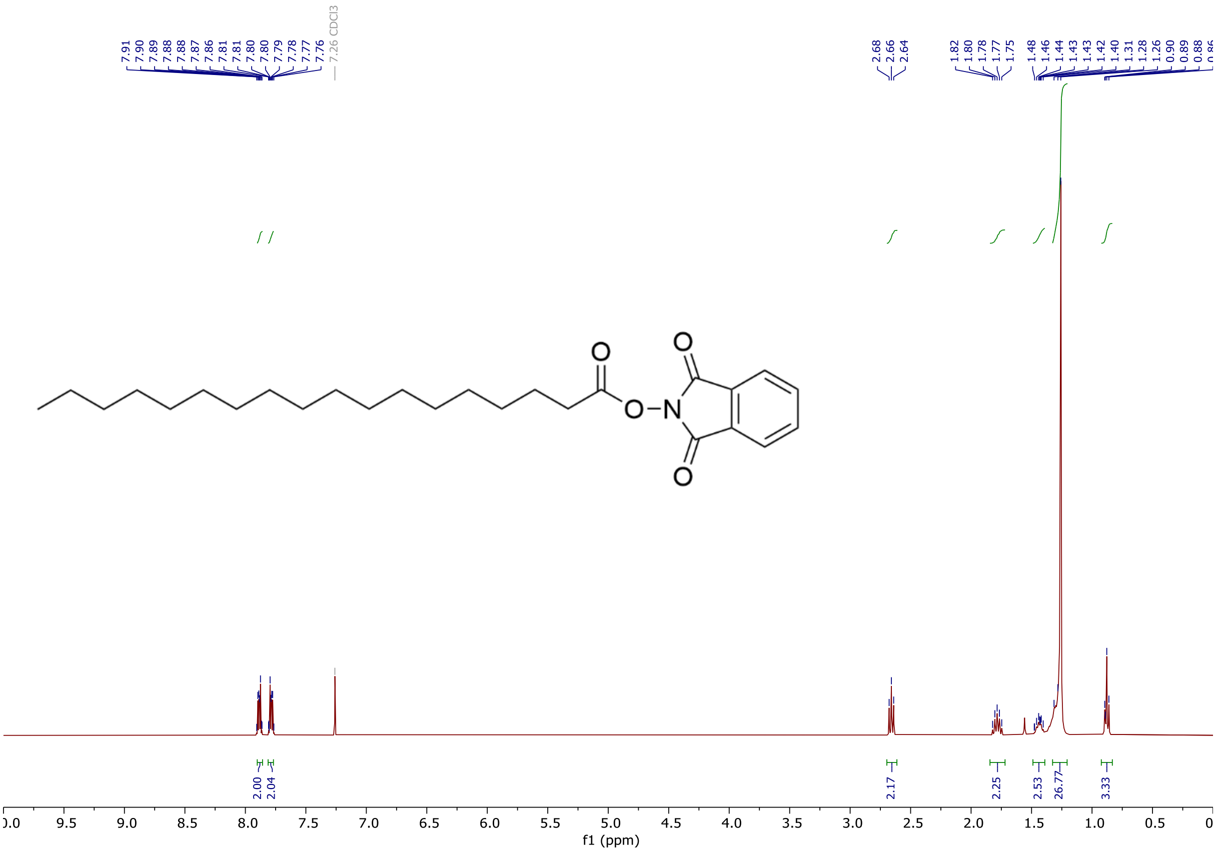

^{13}C NMR (101 MHz, $CDCl_3$): **4am**

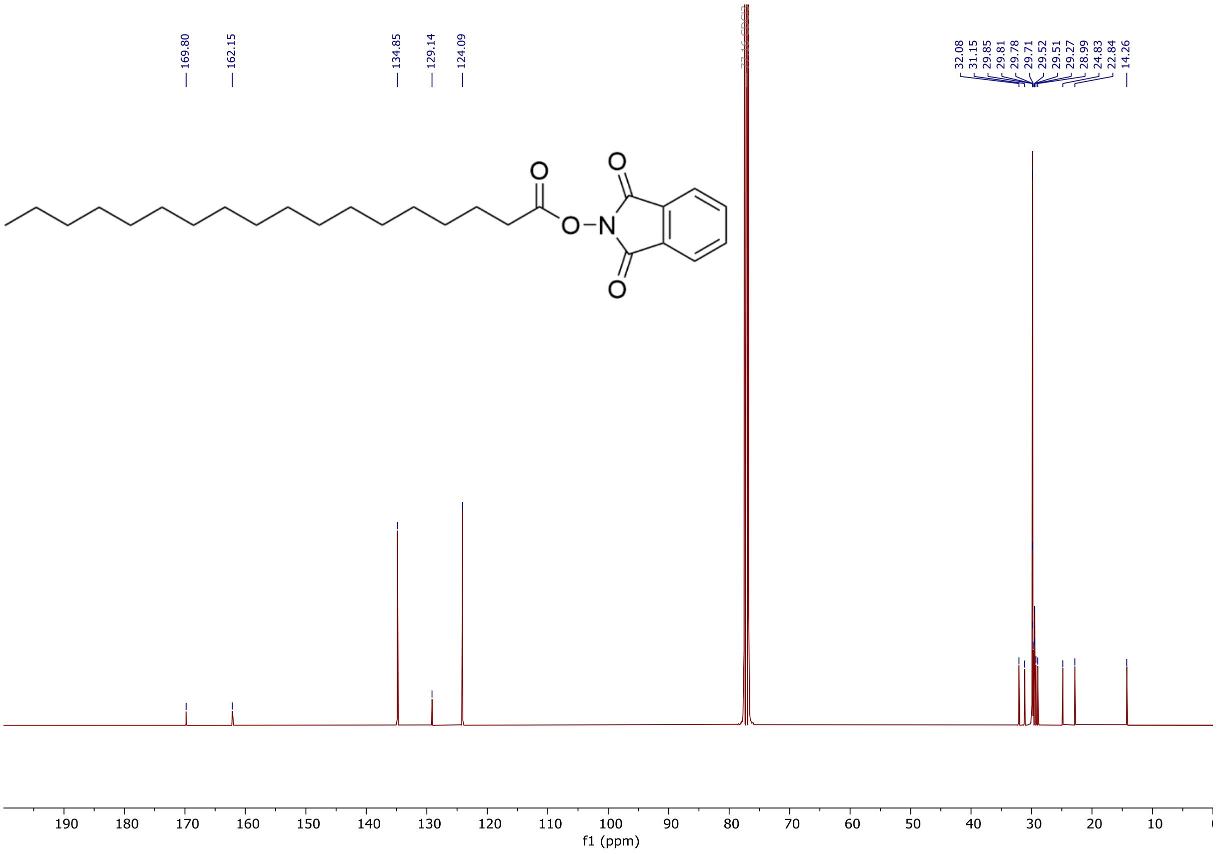

^{1}H NMR (400 MHz, $CDCl_3$): **4an**

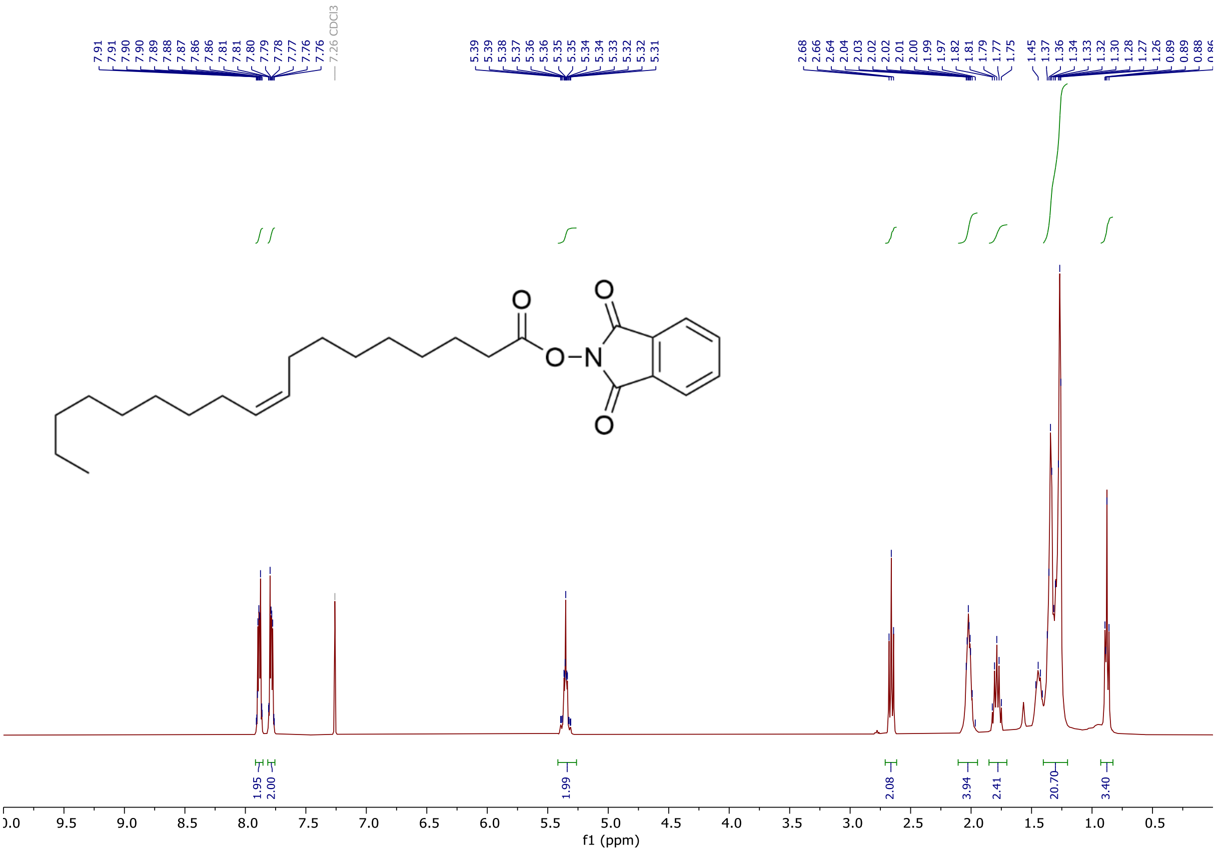

^{13}C NMR (101 MHz, $CDCl_3$): **4an**

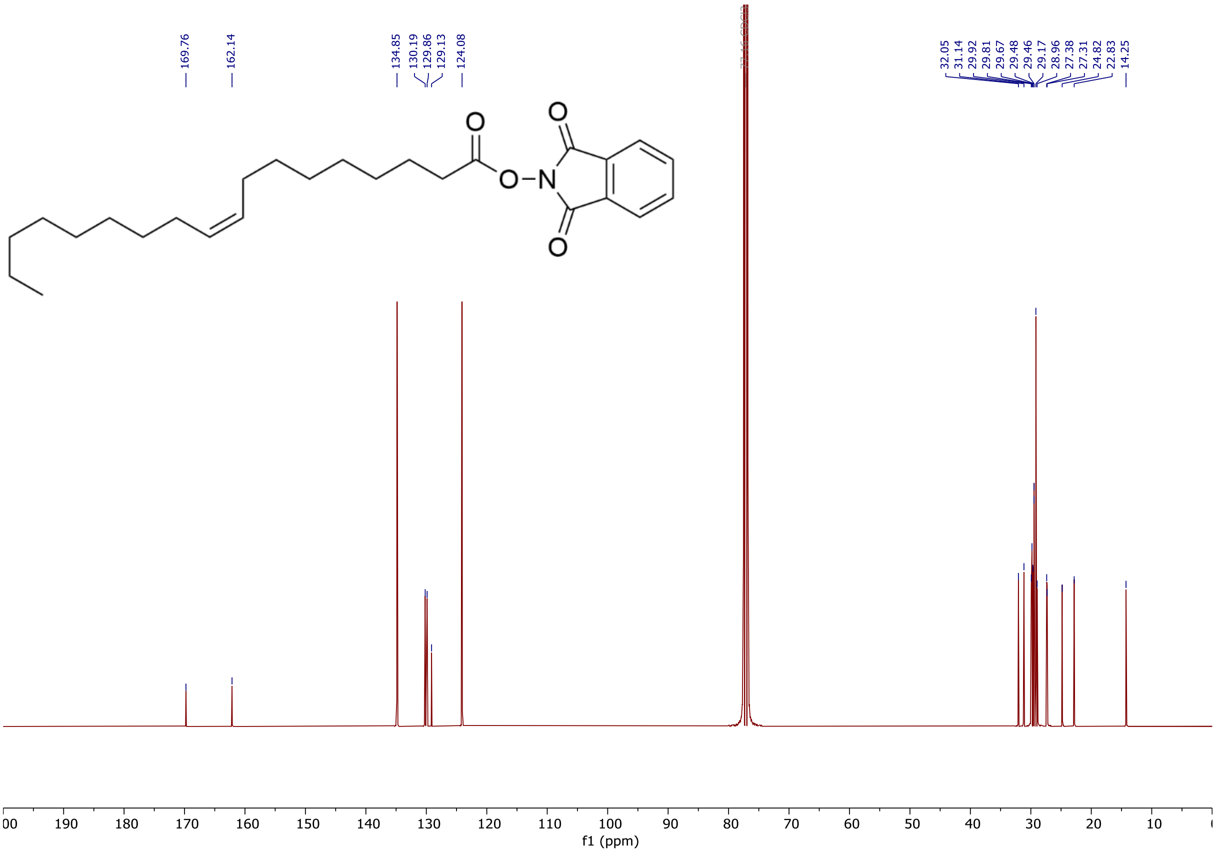

^{1}H NMR (400 MHz, $CDCl_3$): **4ao**

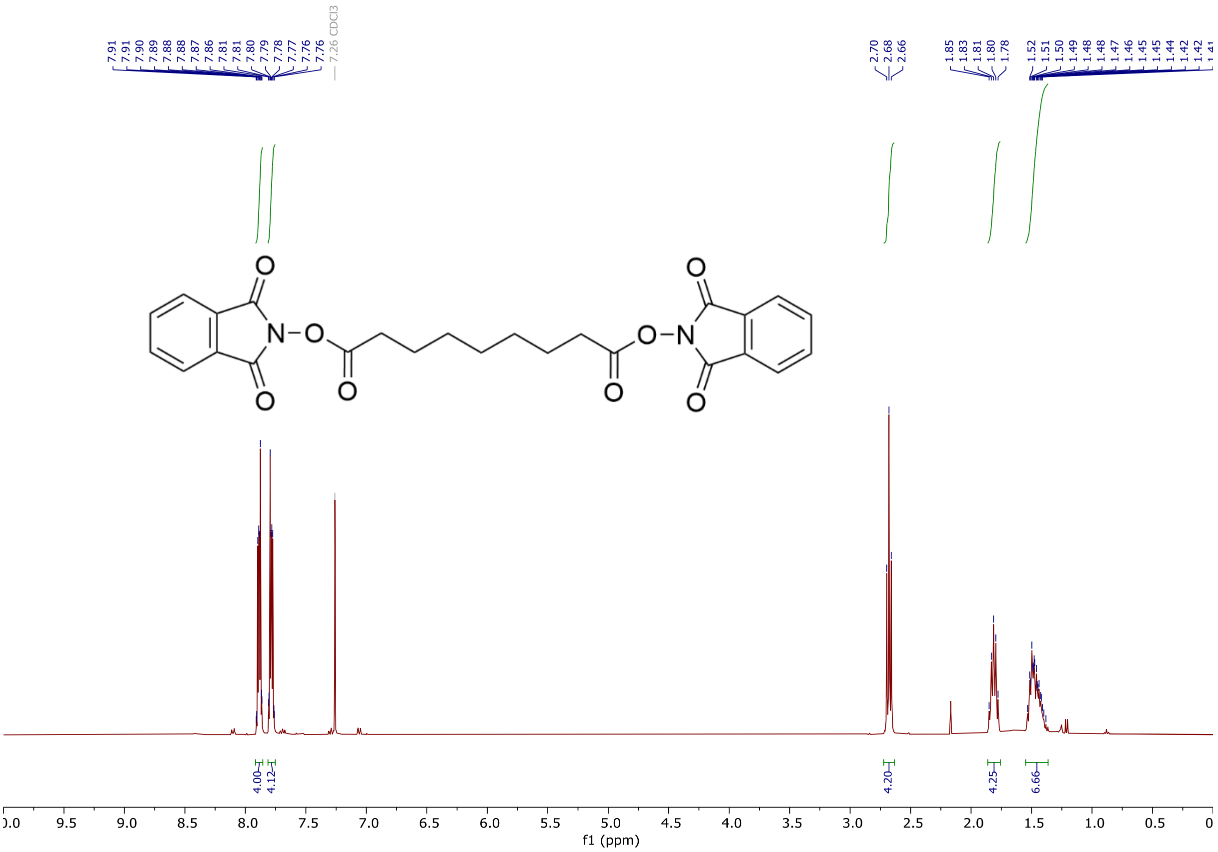

^{13}C NMR (101 MHz, $CDCl_3$): **4ao**

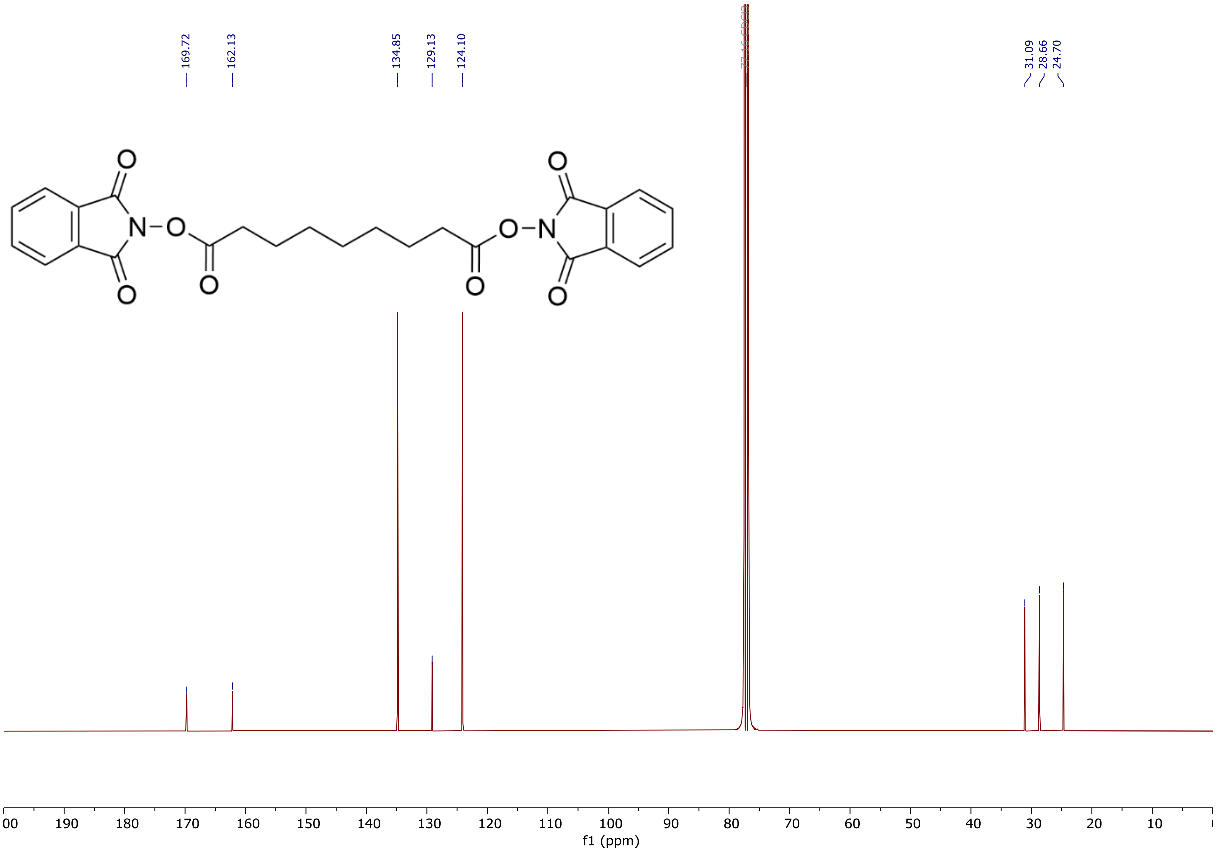

^{1}H NMR (400 MHz, $CDCl_3$): **4ap**

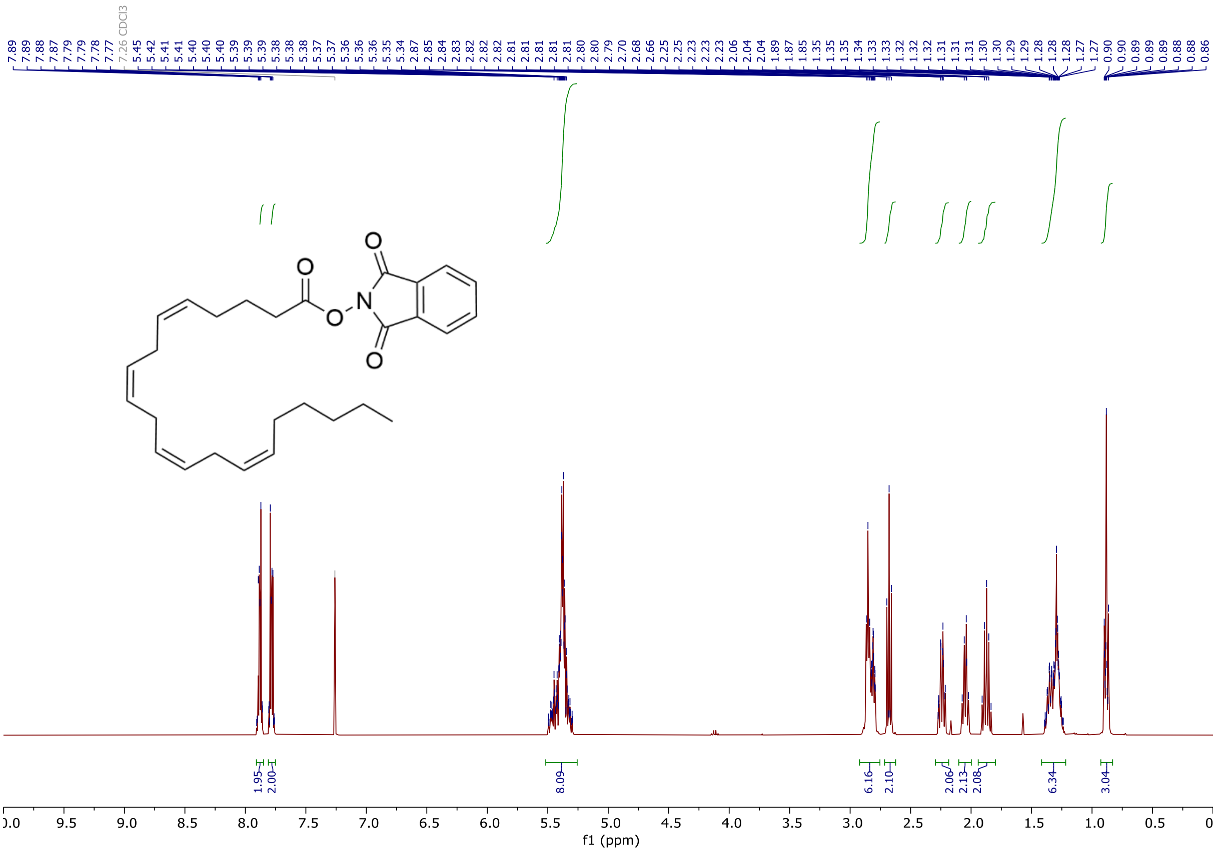

^{13}C NMR (101 MHz, $CDCl_3$): **4ap**

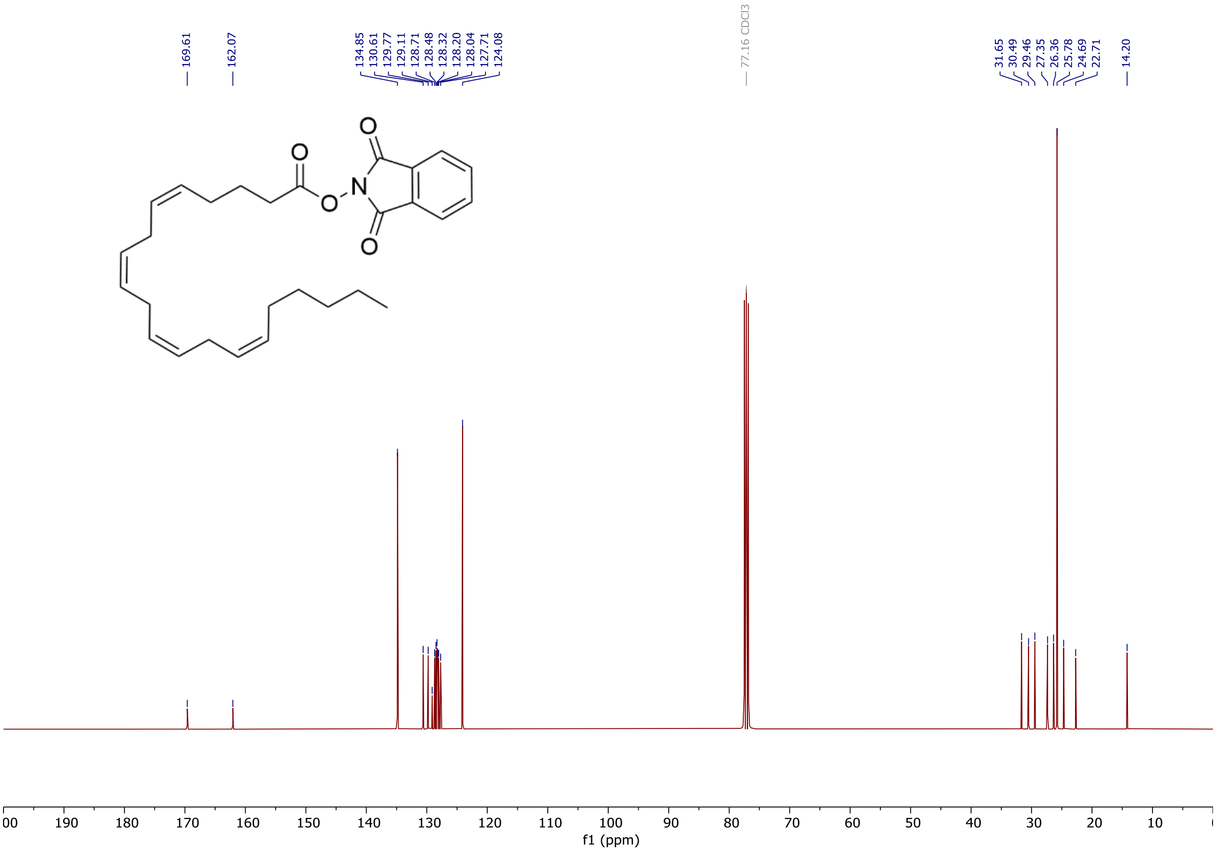

^{1}H NMR (400 MHz, $CDCl_3$): **4aq**

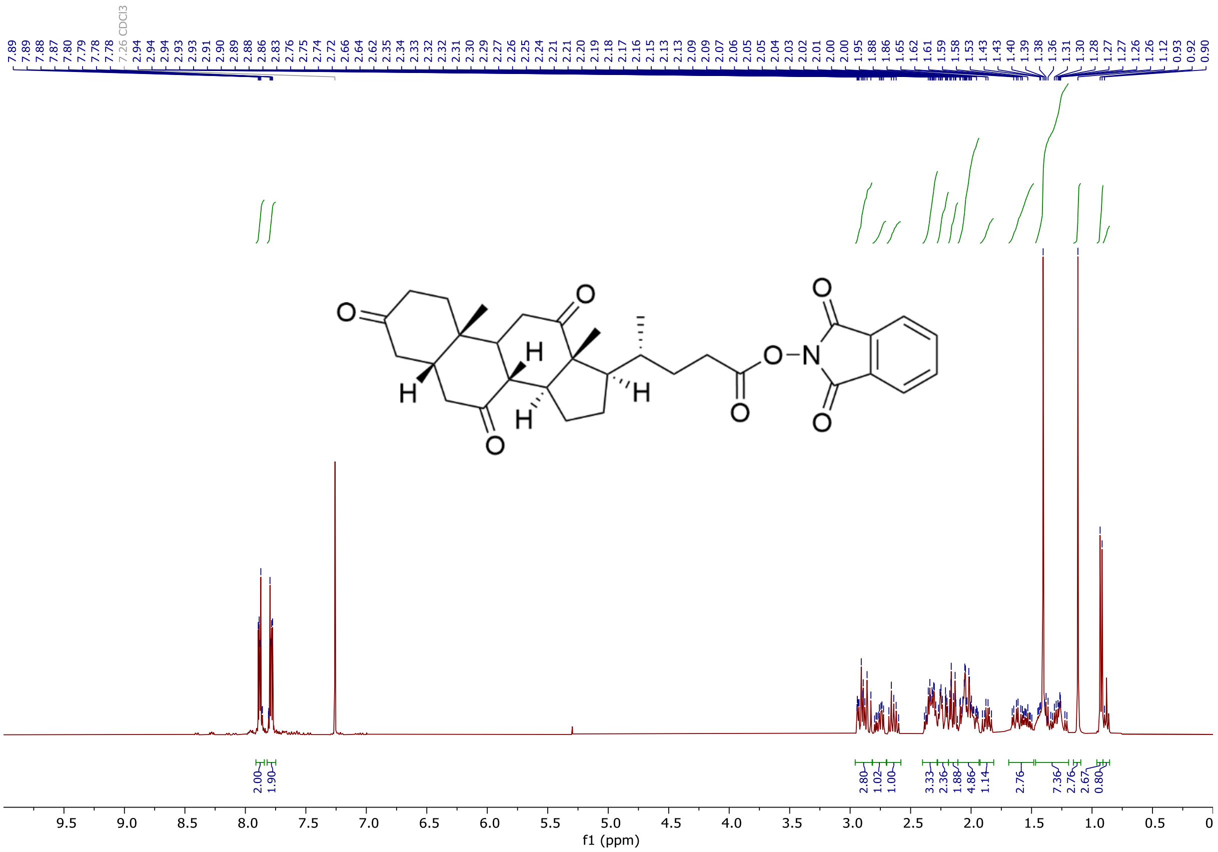

^{13}C NMR (101 MHz, $CDCl_3$): **4aq**

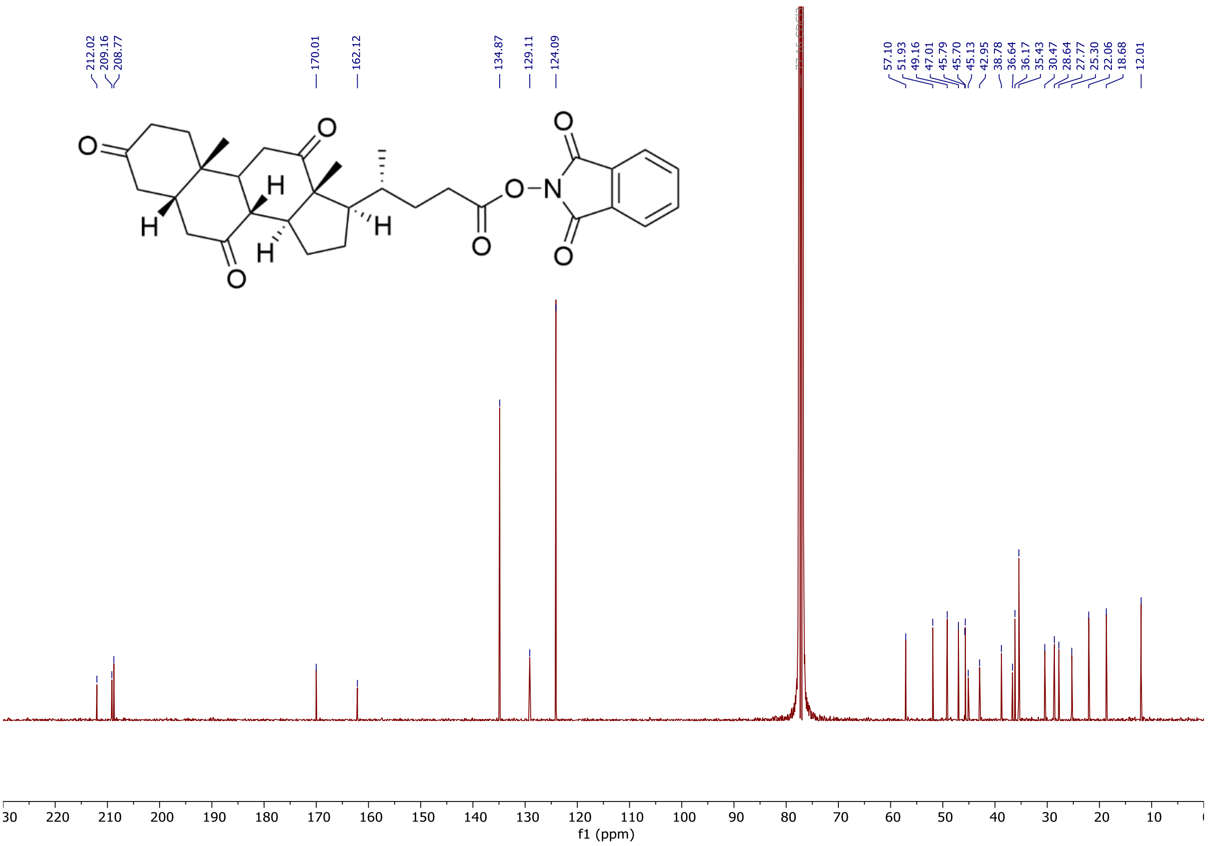

^{1}H NMR (400 MHz, $CDCl_3$): **4ar**

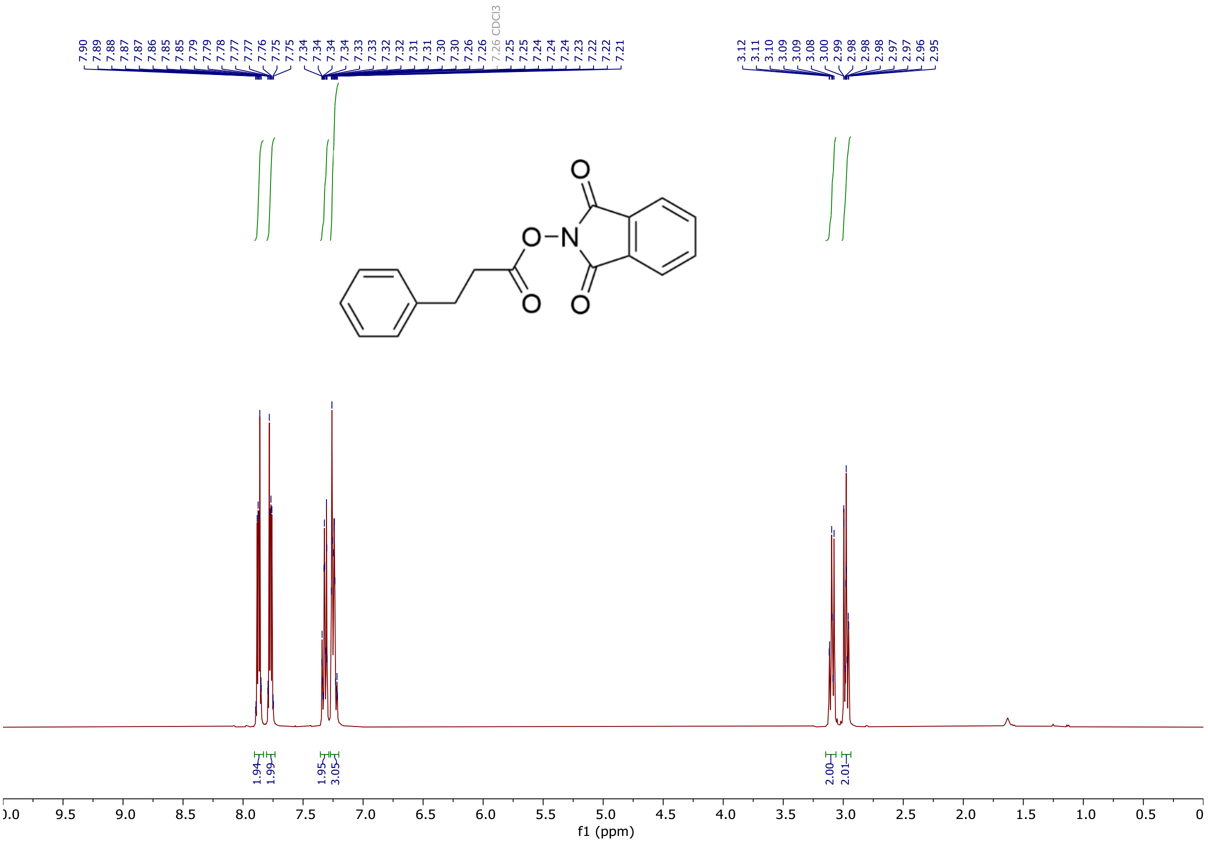

^{13}C NMR (101 MHz, $CDCl_3$): **4ar**

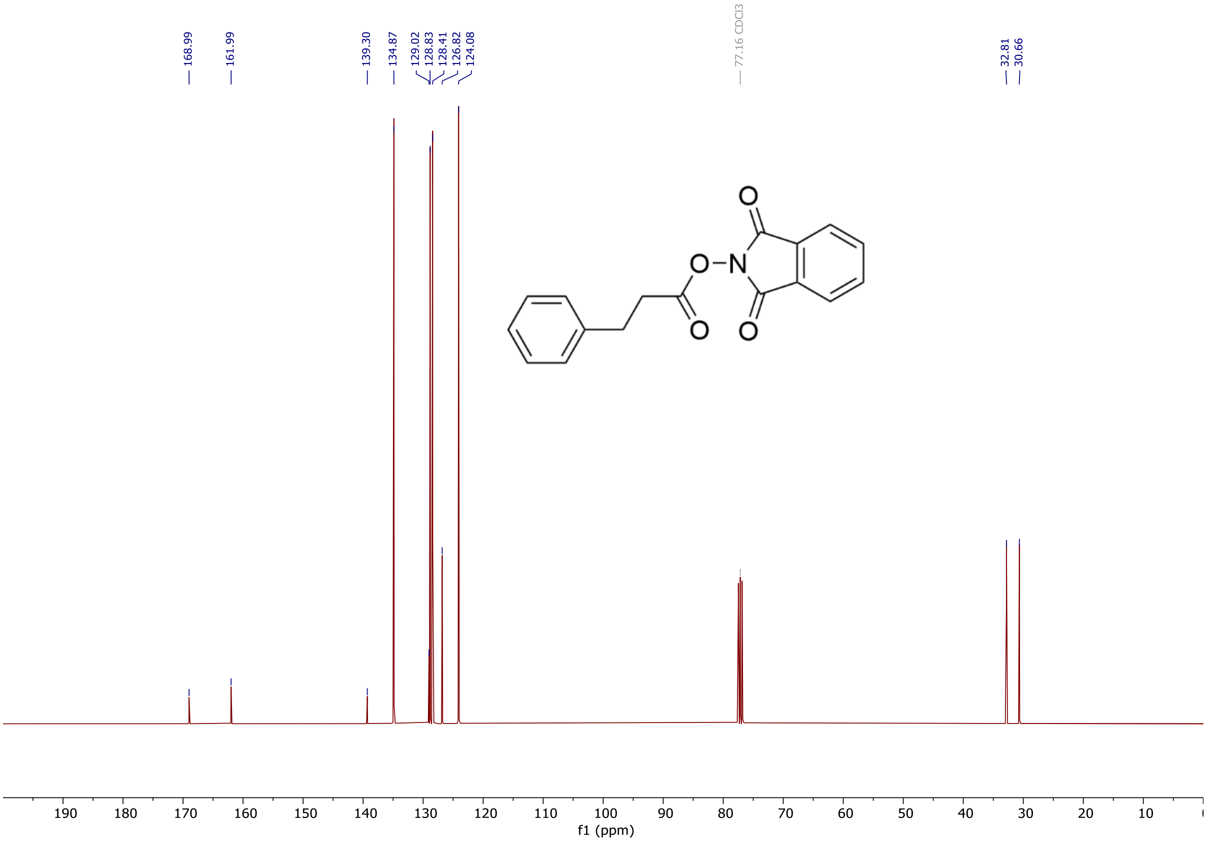

^{1}H NMR (400 MHz, CDCl$_3$): **4as**

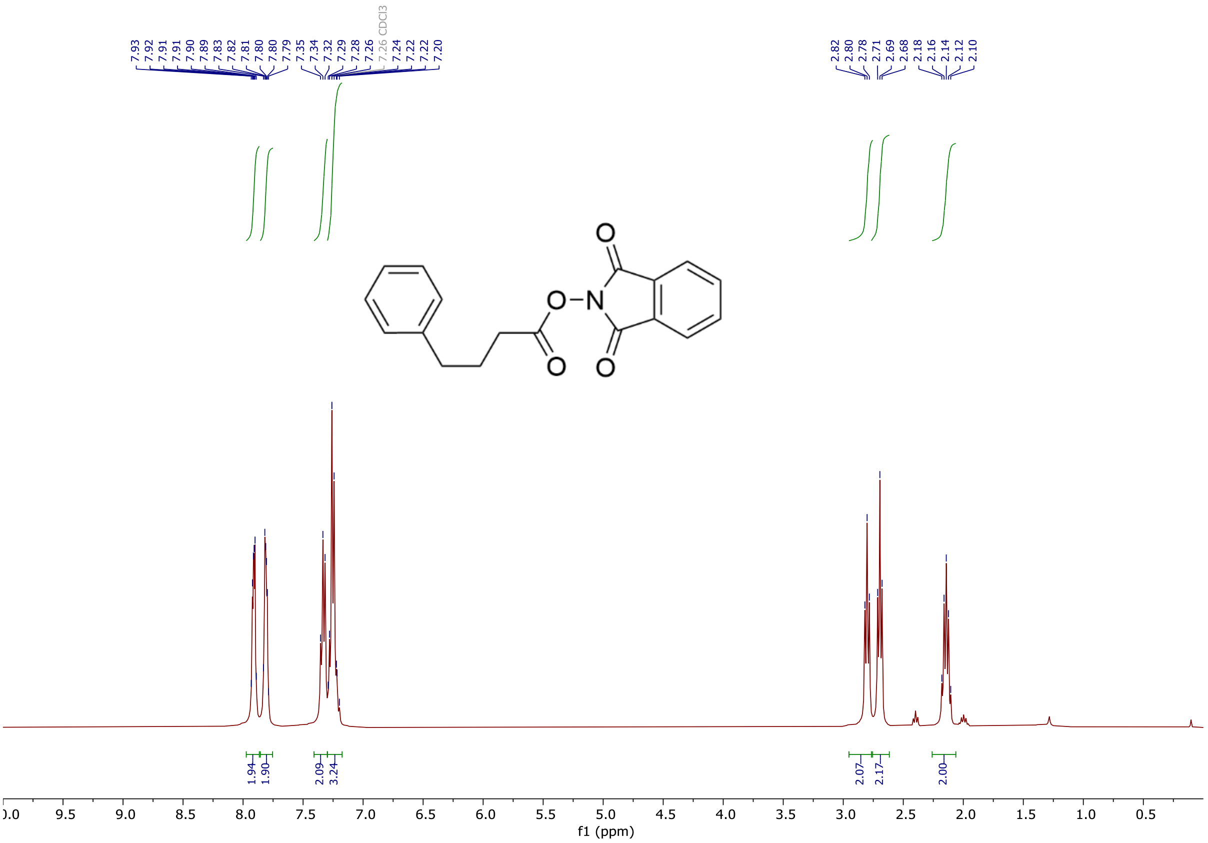

^{13}C NMR (101 MHz, CDCl$_3$): **4as**

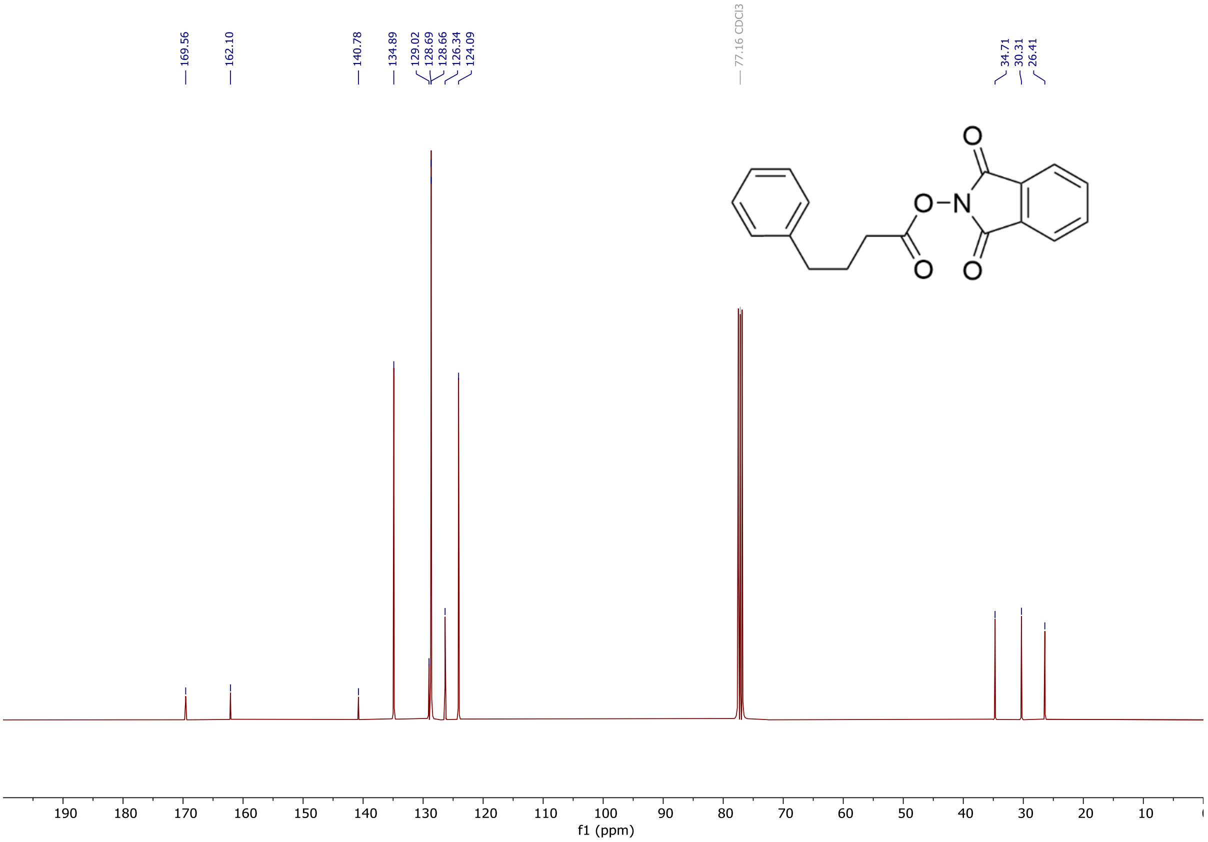

^{1}H NMR (400 MHz, $CDCl_3$): **4at**

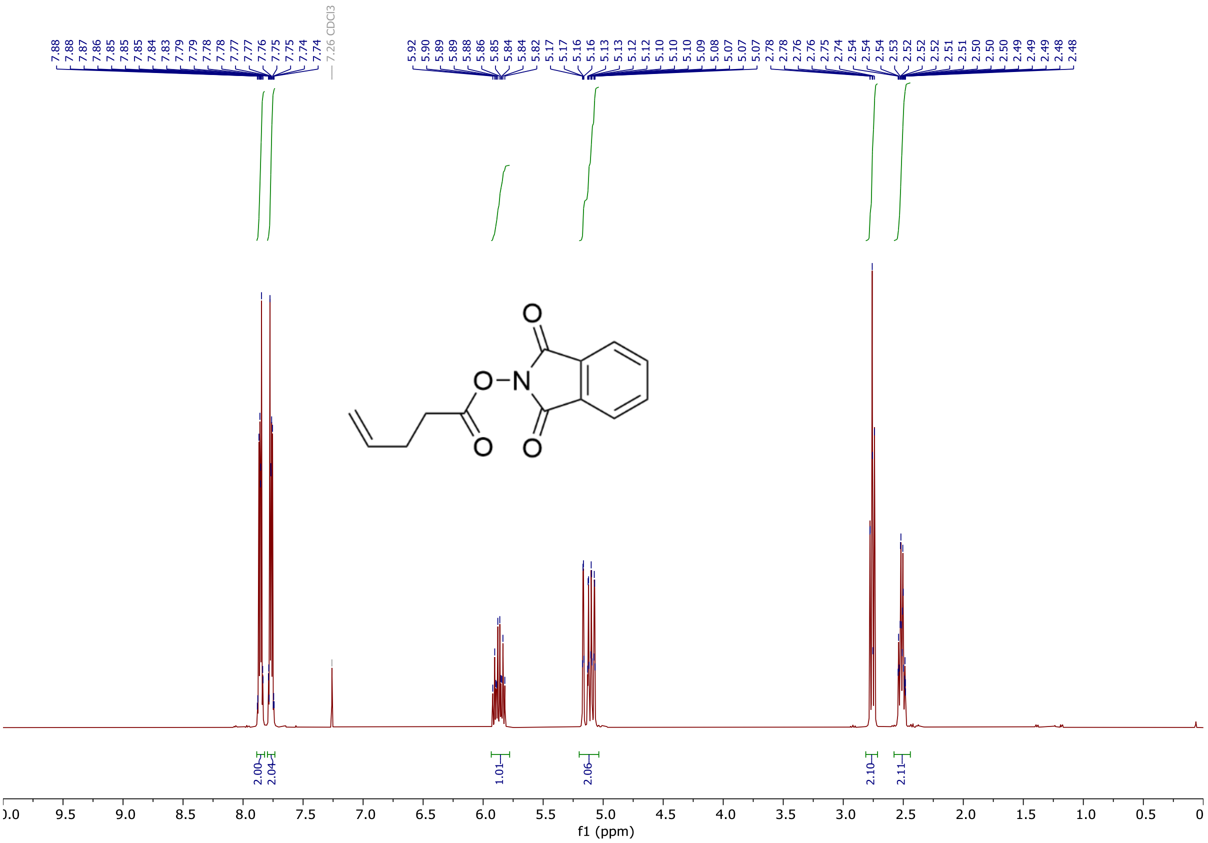

^{13}C NMR (101 MHz, $CDCl_3$): **4at**

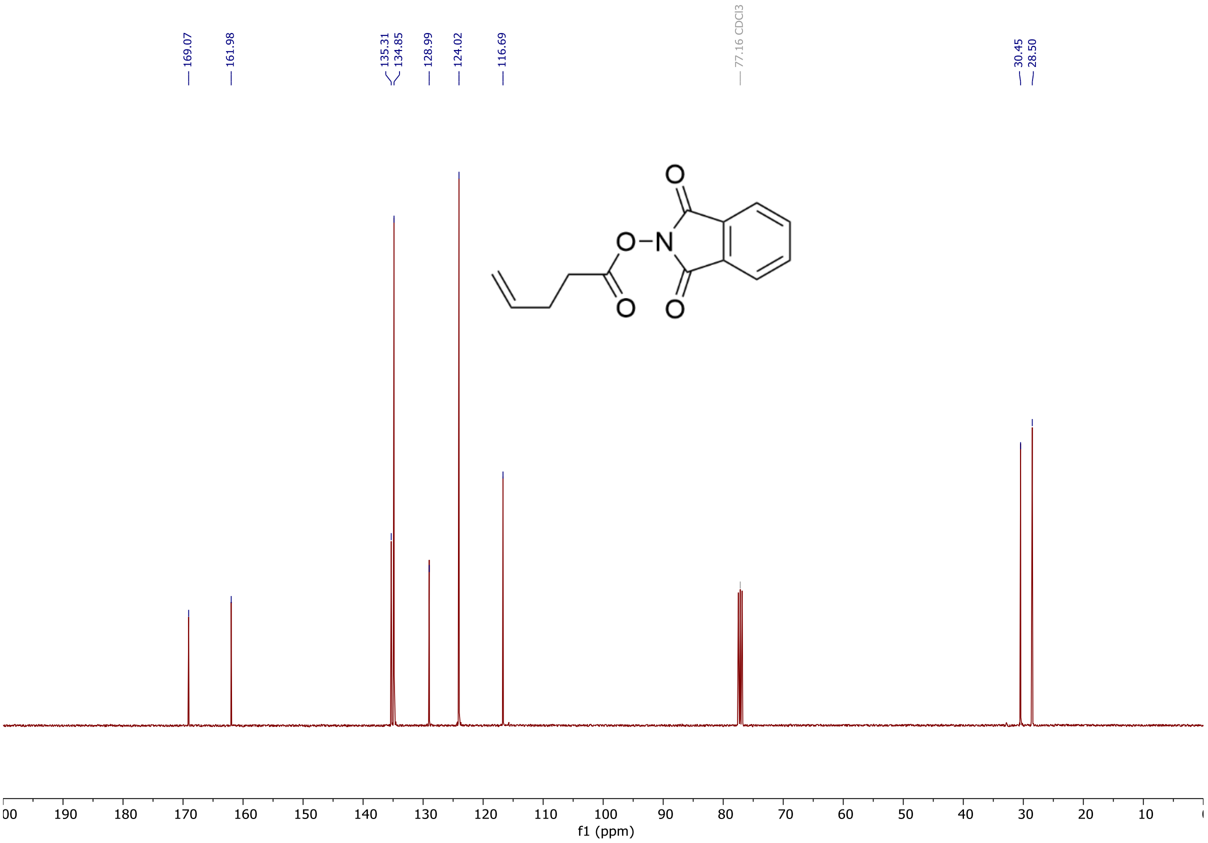

^{1}H NMR (400 MHz, CDCl3): **4au**

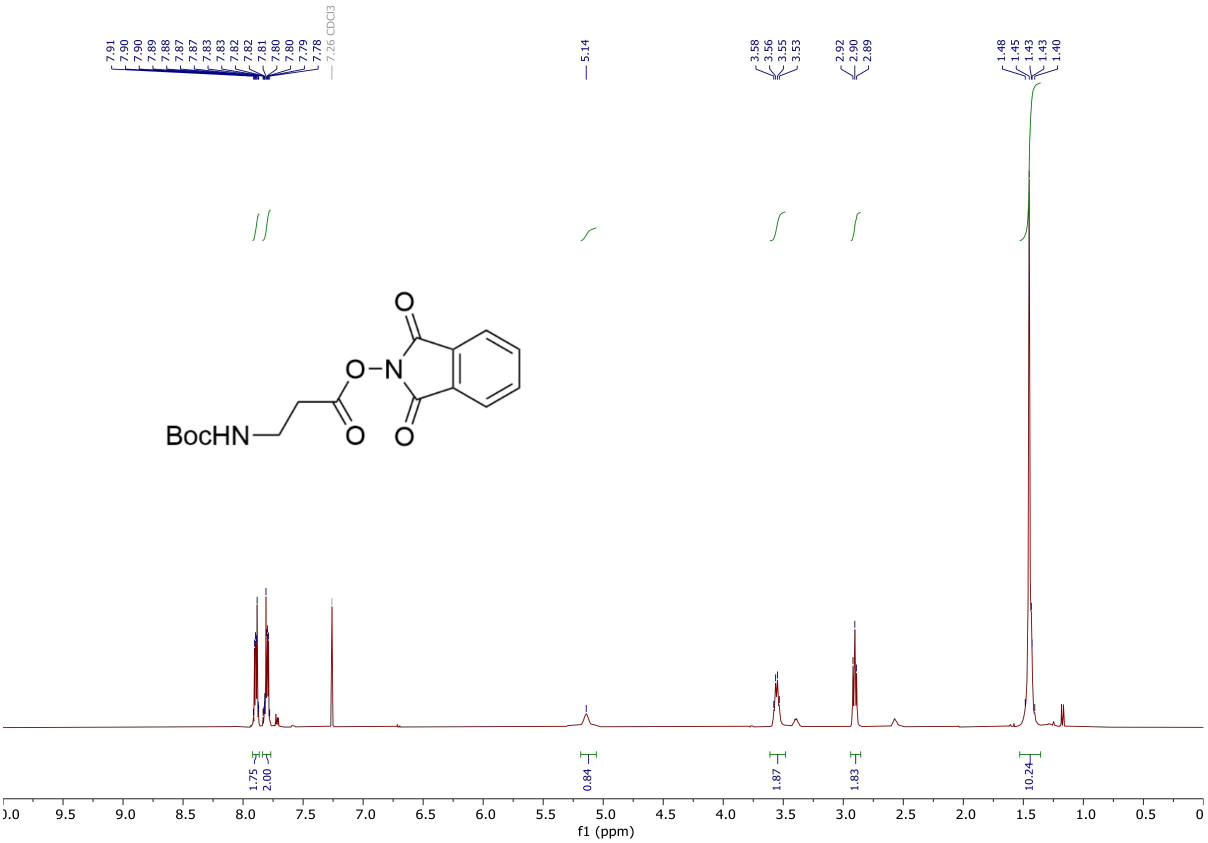

^{13}C NMR (101 MHz, CDCl3): **4au**

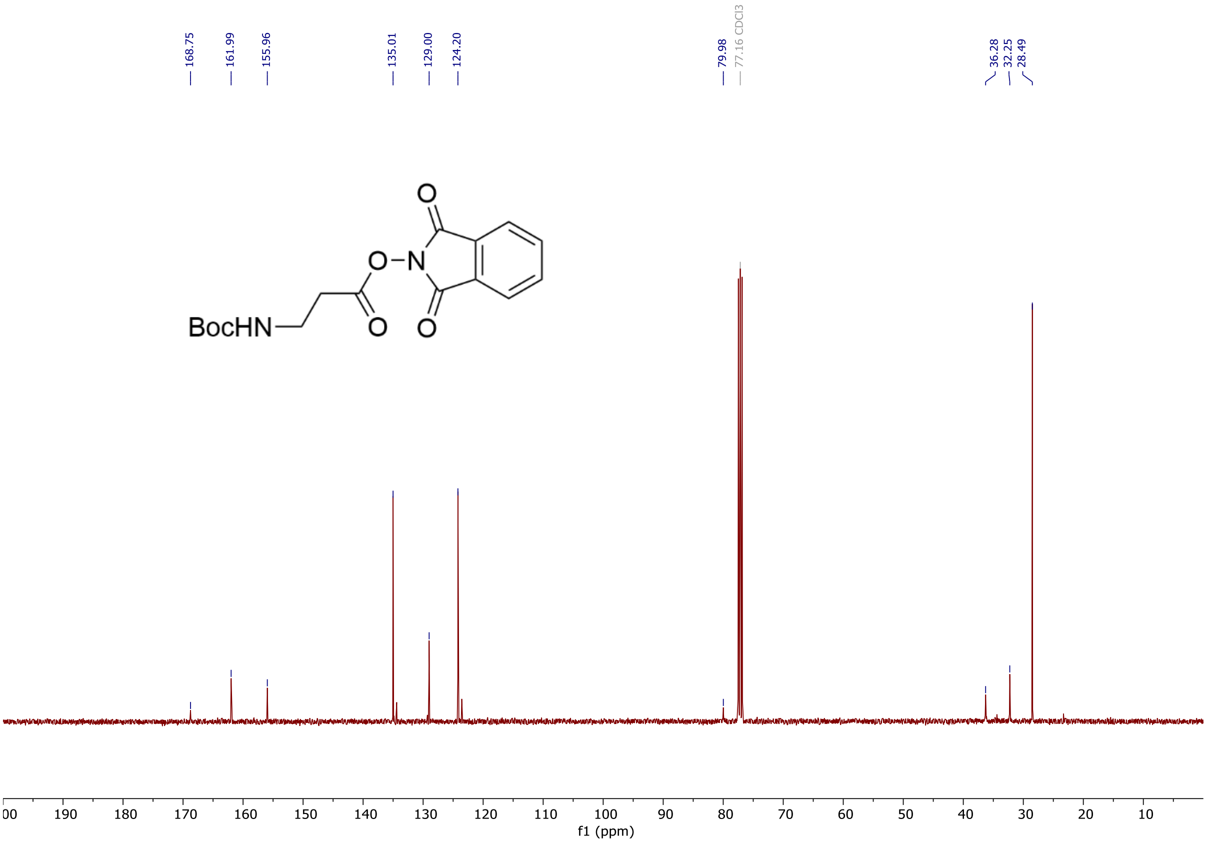

^{1}H NMR (400 MHz, $CDCl_3$): **4av**

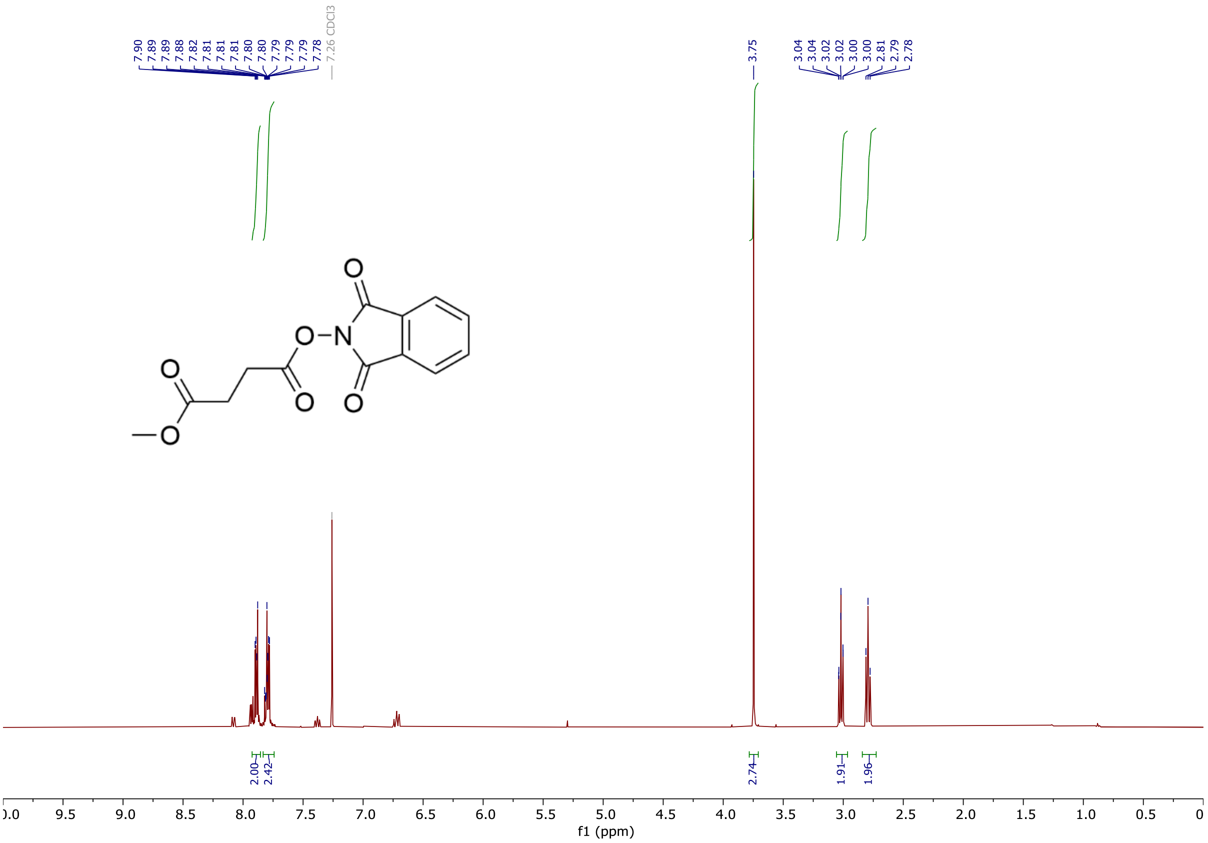

^{13}C NMR (101 MHz, $CDCl_3$): **4av**

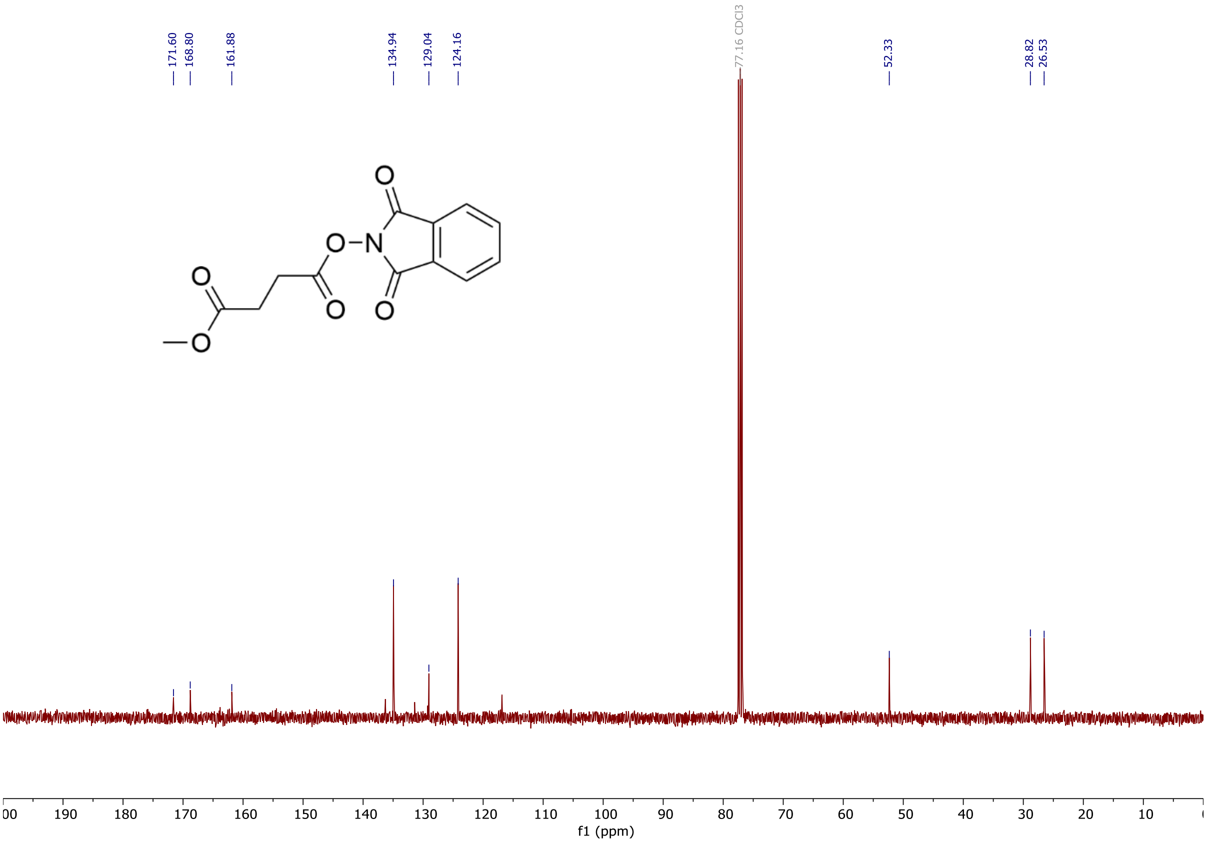

^{1}H NMR (400 MHz, $CDCl_3$): **4az**

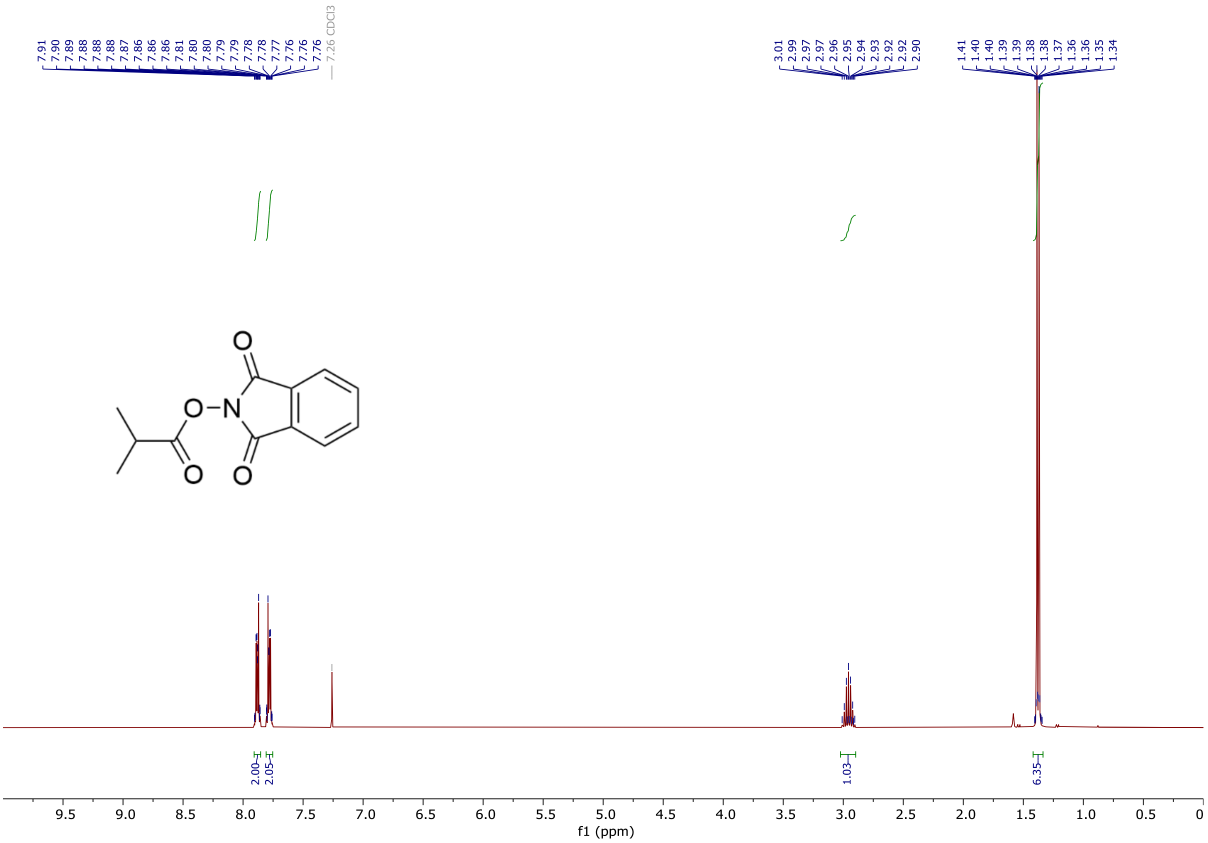

^{13}C NMR (101 MHz, $CDCl_3$): **4az**

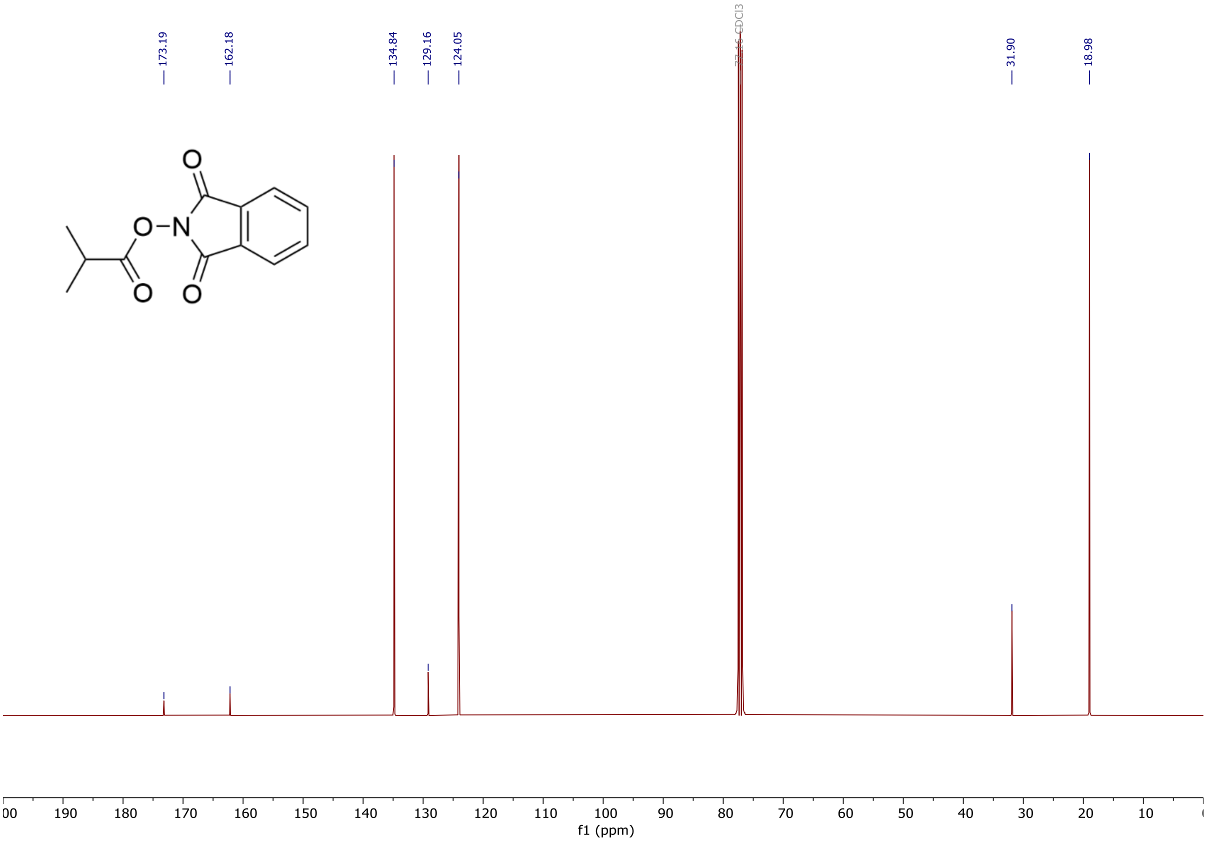

^{1}H NMR (400 MHz, $CDCl_3$): **4ba**

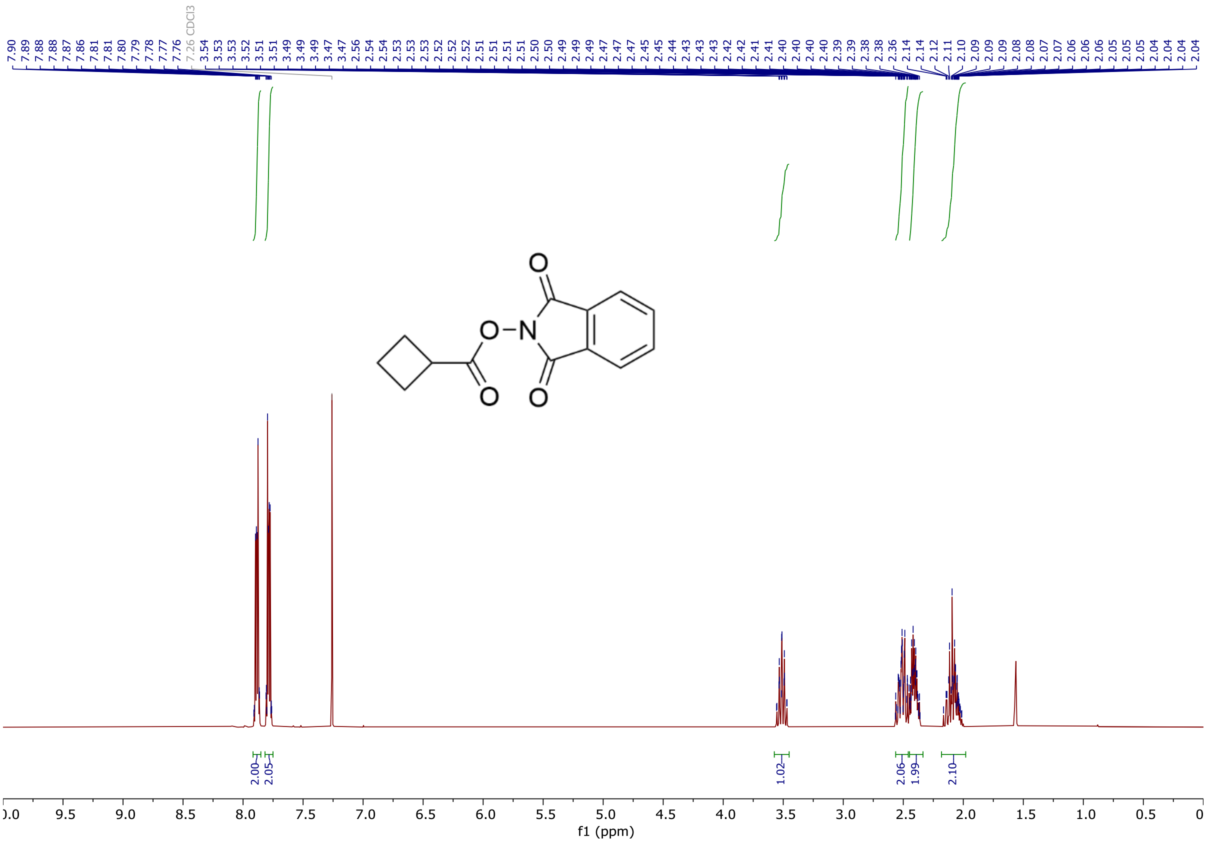

^{13}C NMR (101 MHz, $CDCl_3$): **4ba**

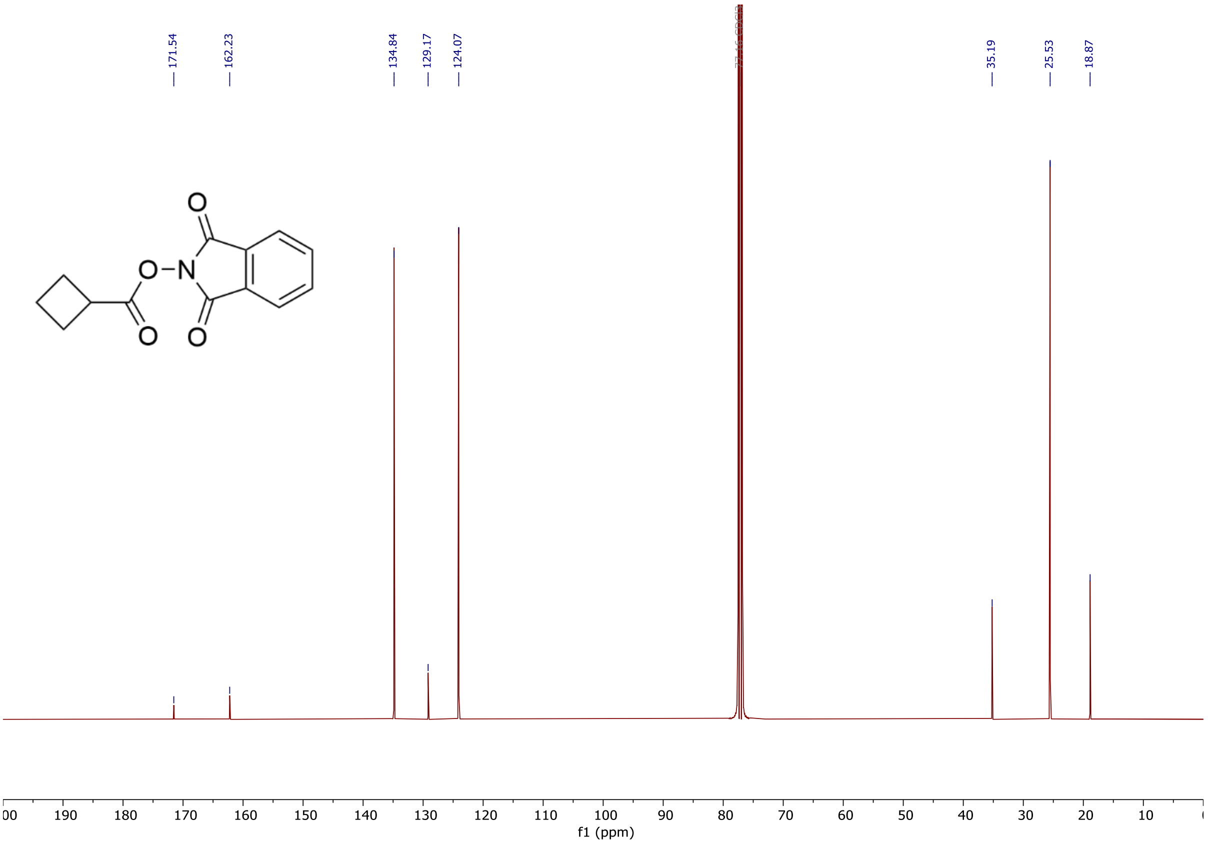

^{1}H NMR (400 MHz, CDCl$_3$): **4bb**

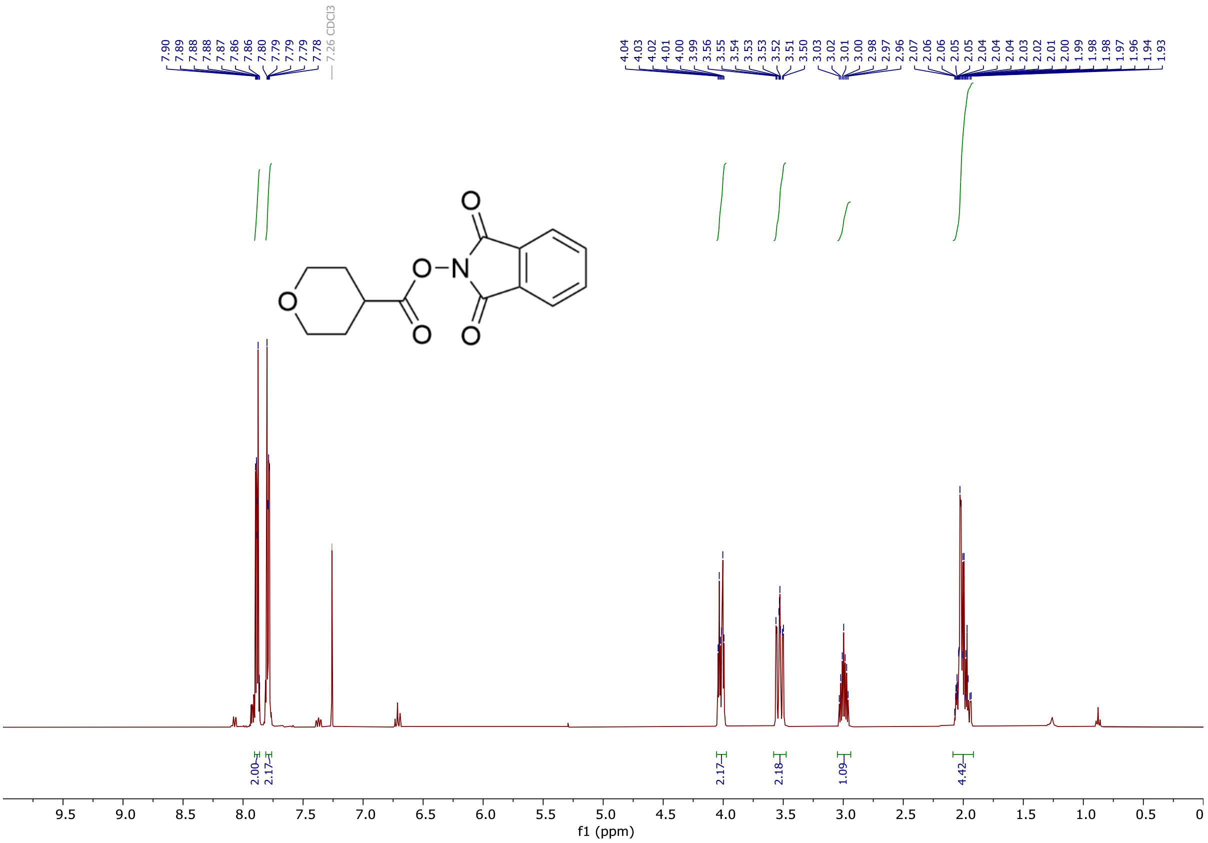

^{13}C NMR (101 MHz, CDCl$_3$): **4bb**

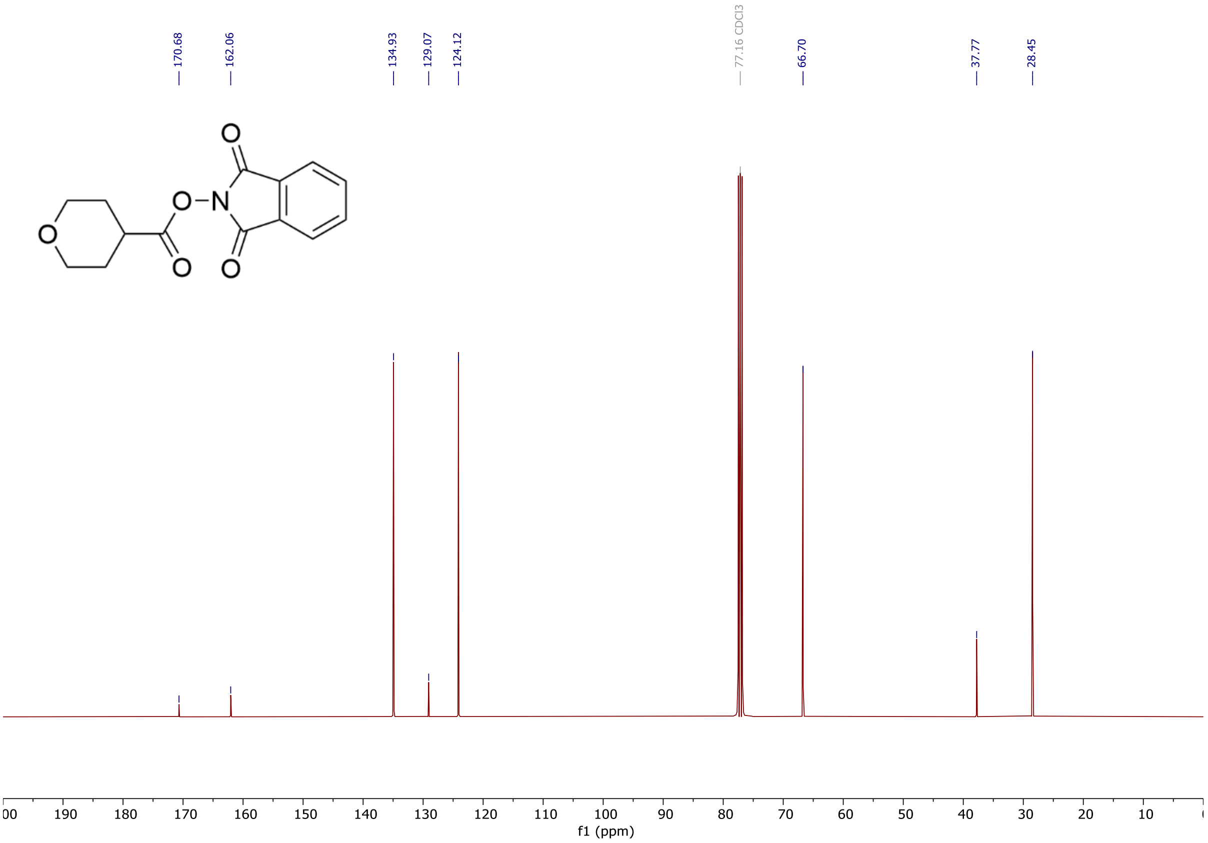

^{1}H NMR (400 MHz, $CDCl_3$): **4bc**

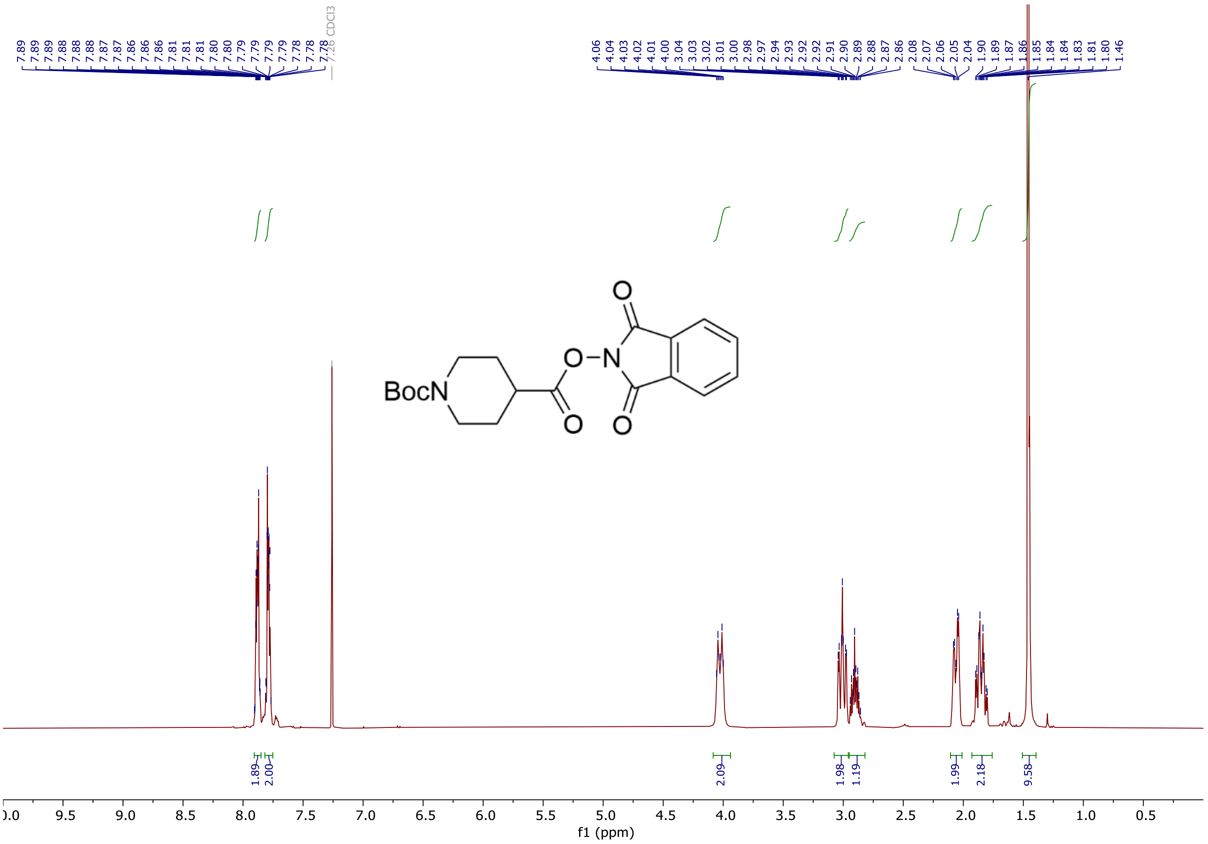

^{13}C NMR (101 MHz, $CDCl_3$): **4bc**

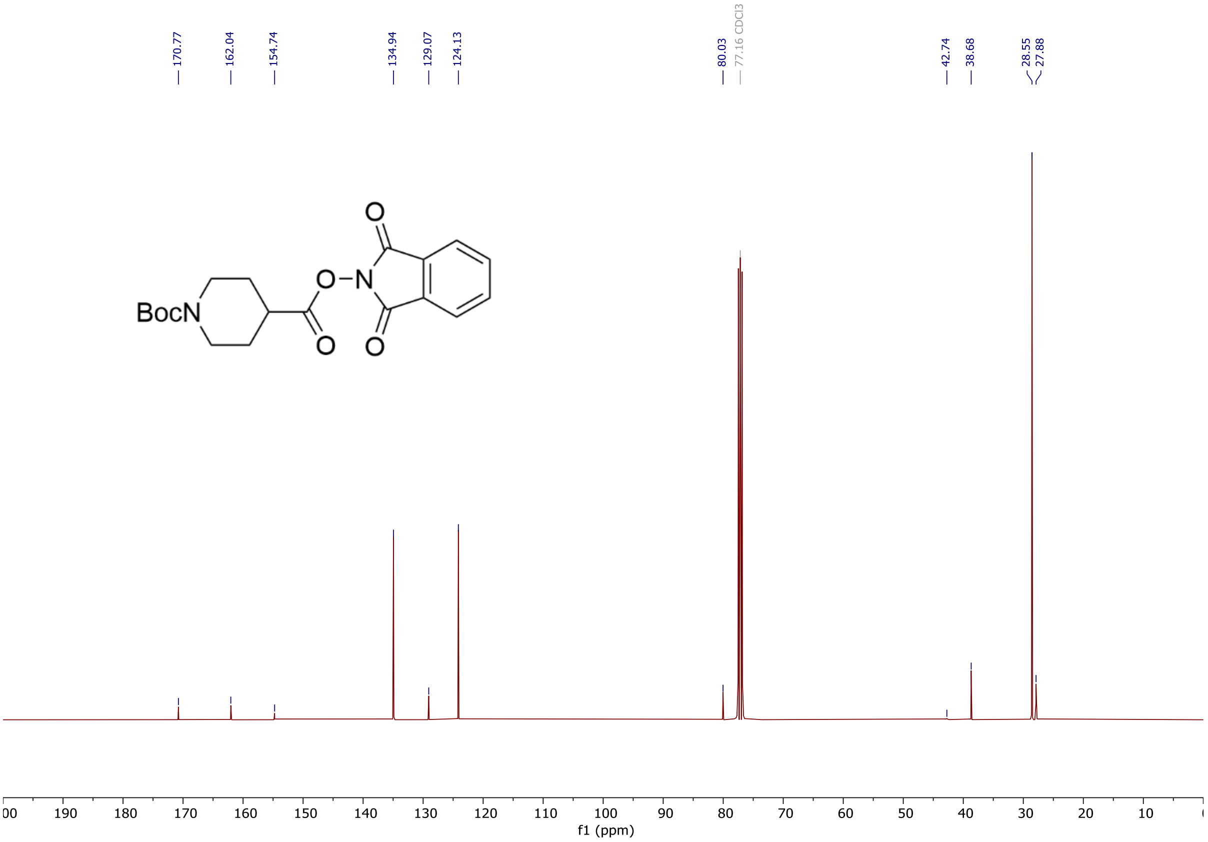

^{1}H NMR (400 MHz, $CDCl_3$): **4bd**

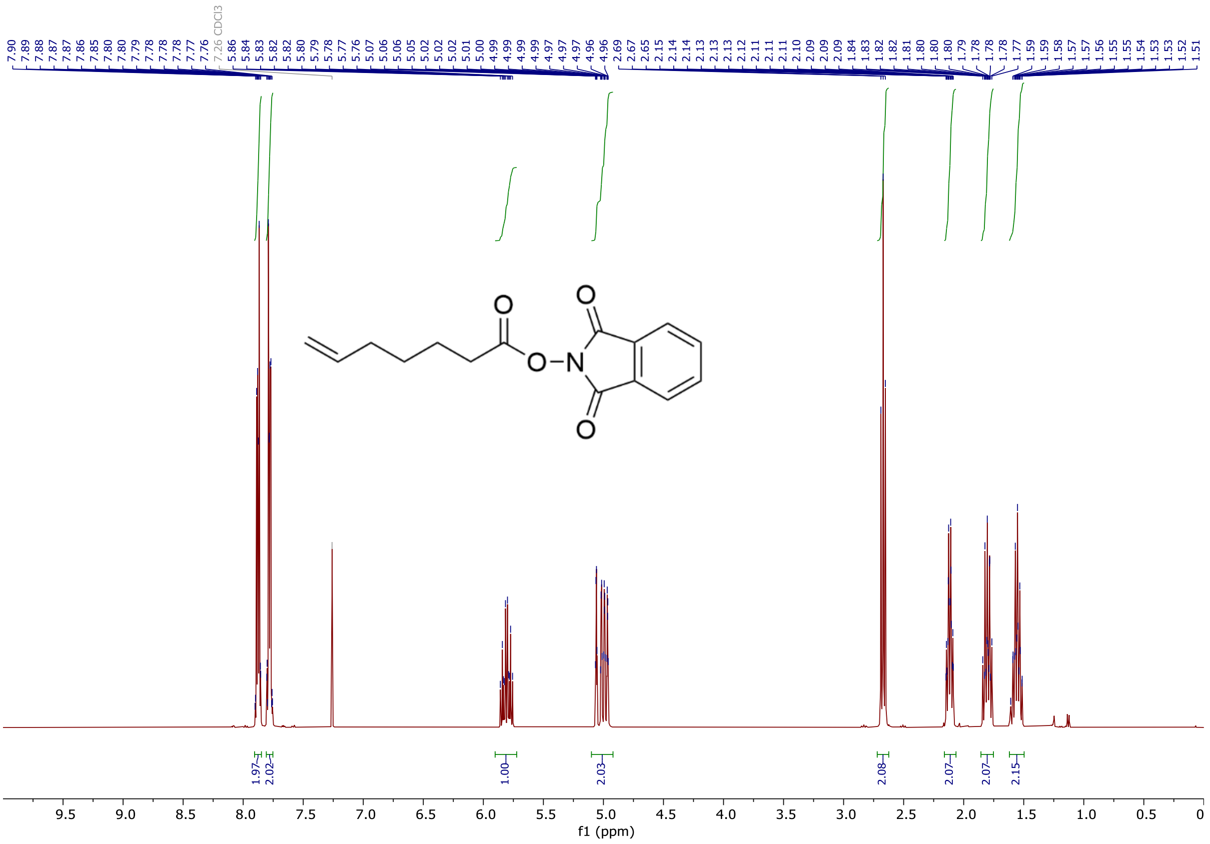

^{13}C NMR (101 MHz, $CDCl_3$): **4bd**

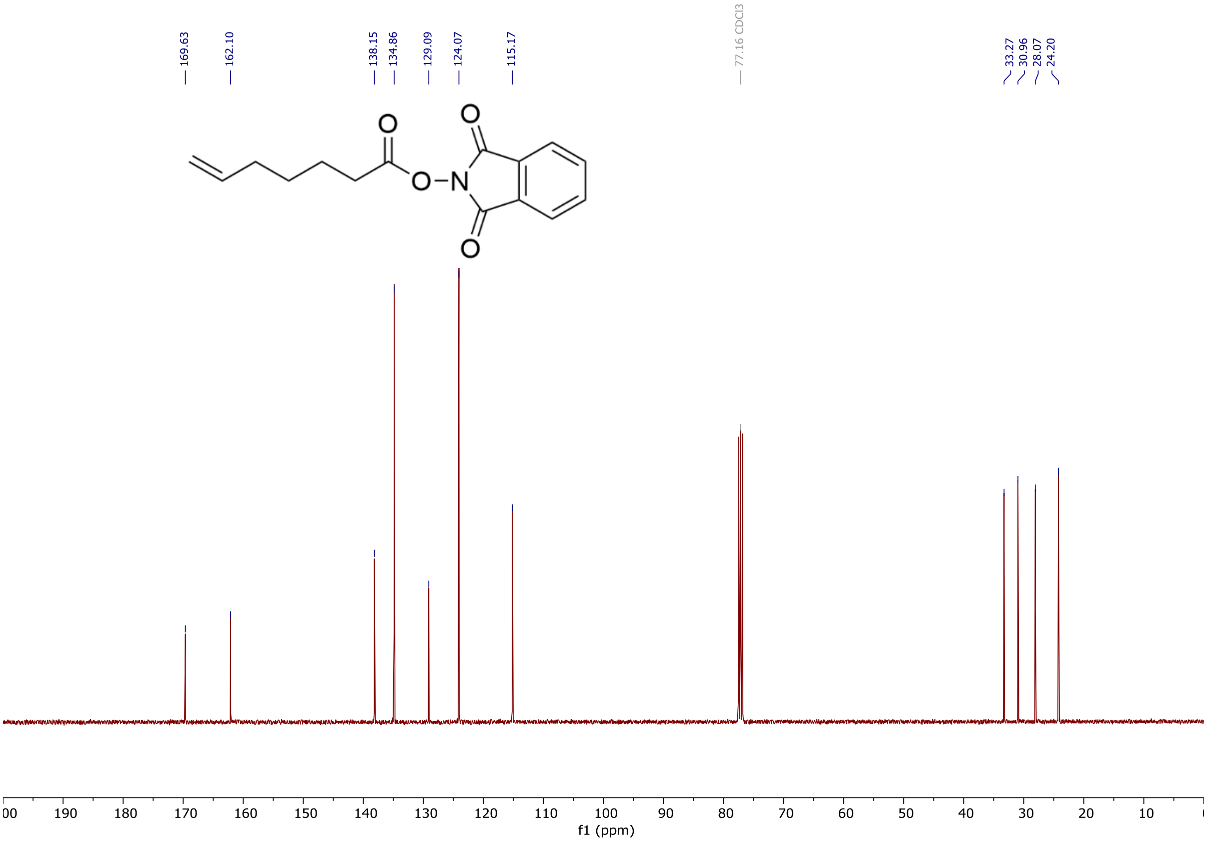

^{1}H NMR (400 MHz, $CDCl_3$): **2a**

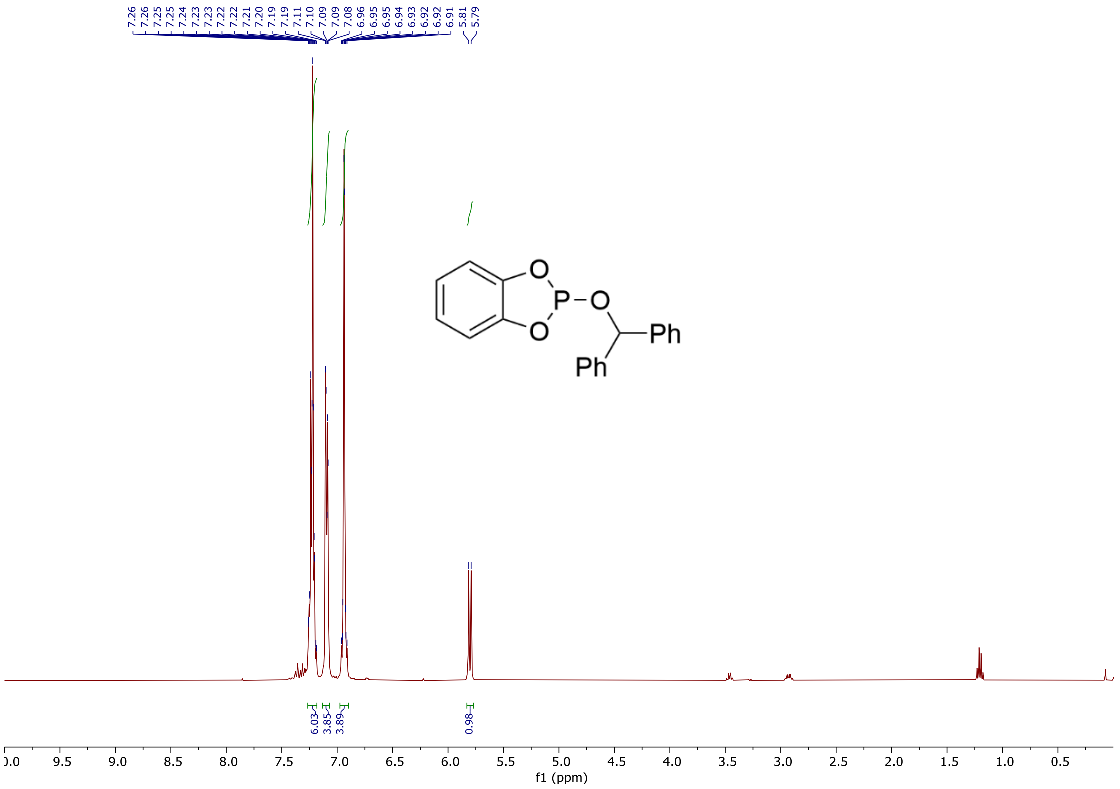

^{13}C NMR (101 MHz, $CDCl_3$): **2a**

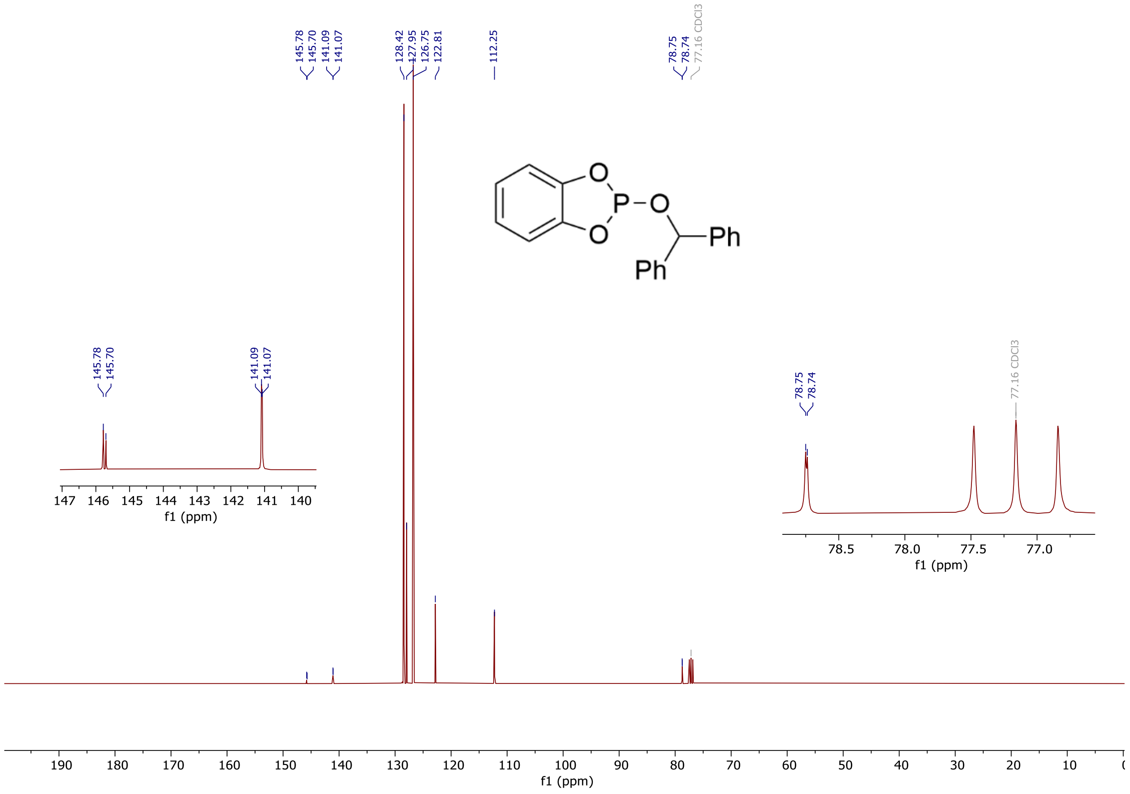

^{31}P NMR (162 MHz, $CDCl_3$): **2a**

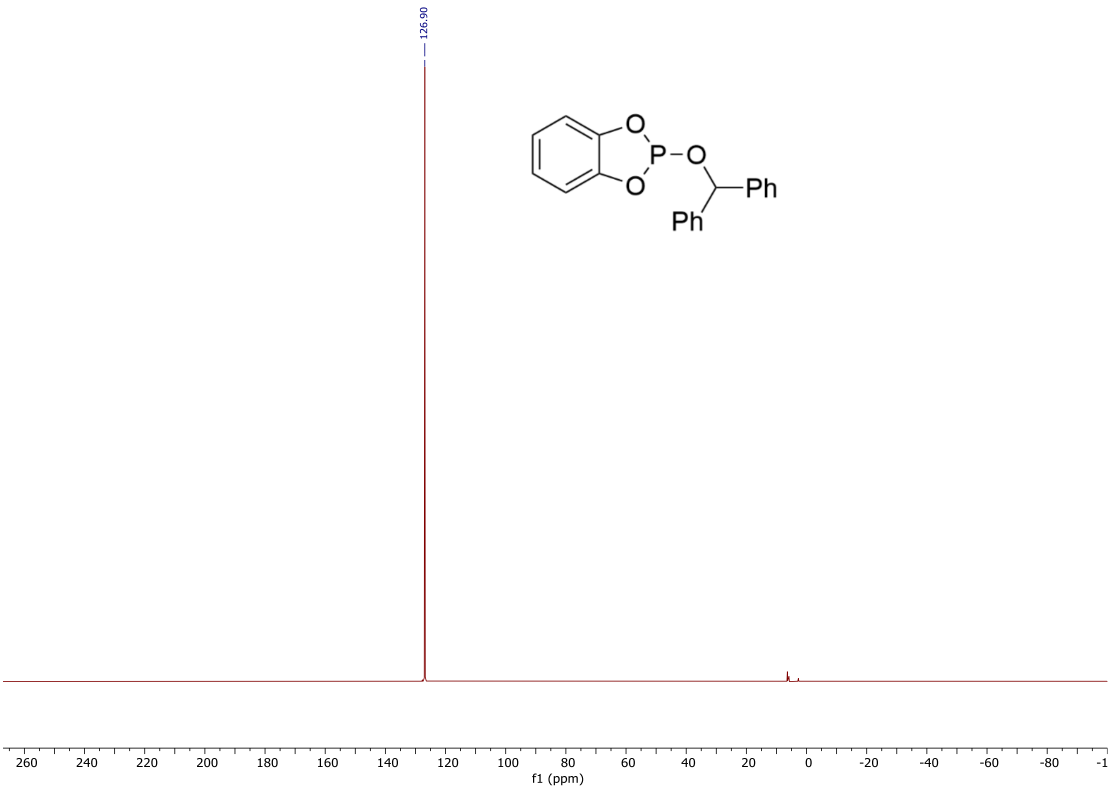

^{1}H NMR (400 MHz, $CDCl_3$): **2e**

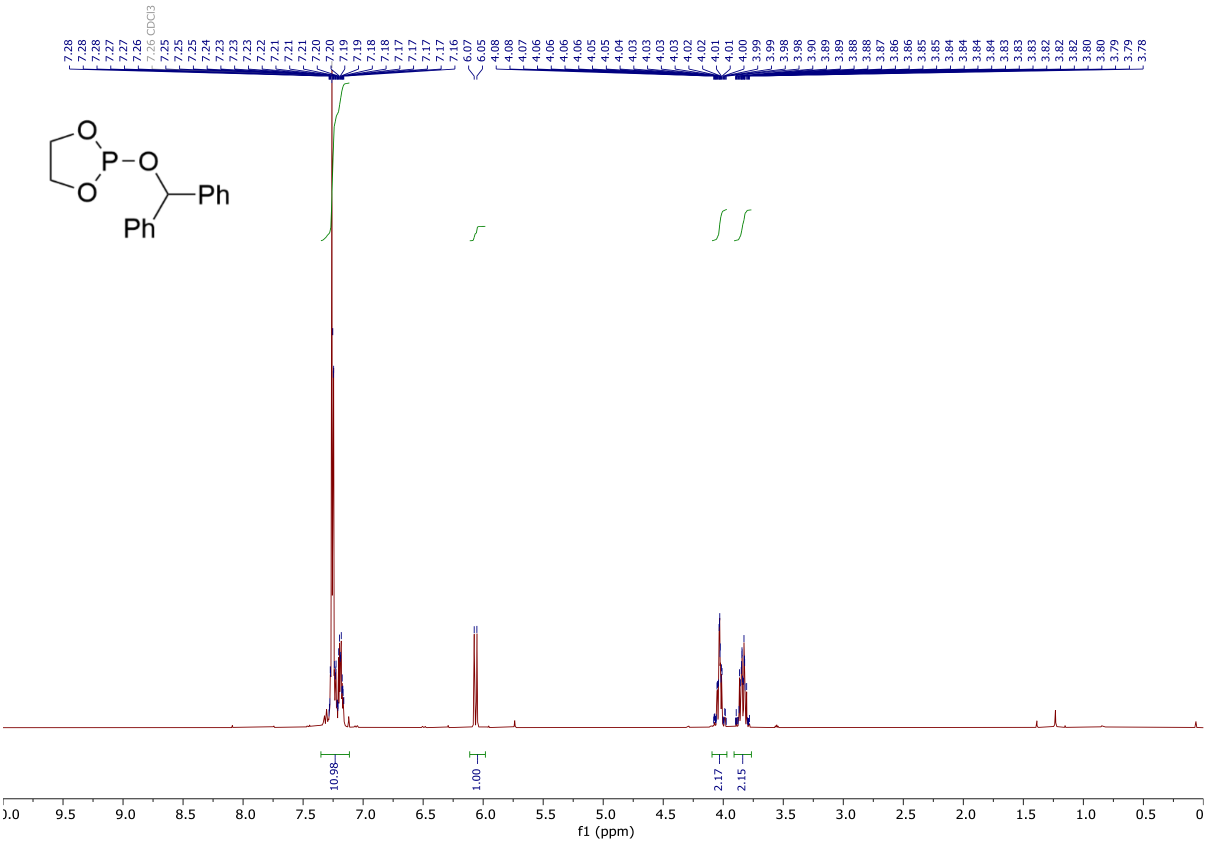

^{13}C NMR (101 MHz, $CDCl_3$): **2e**

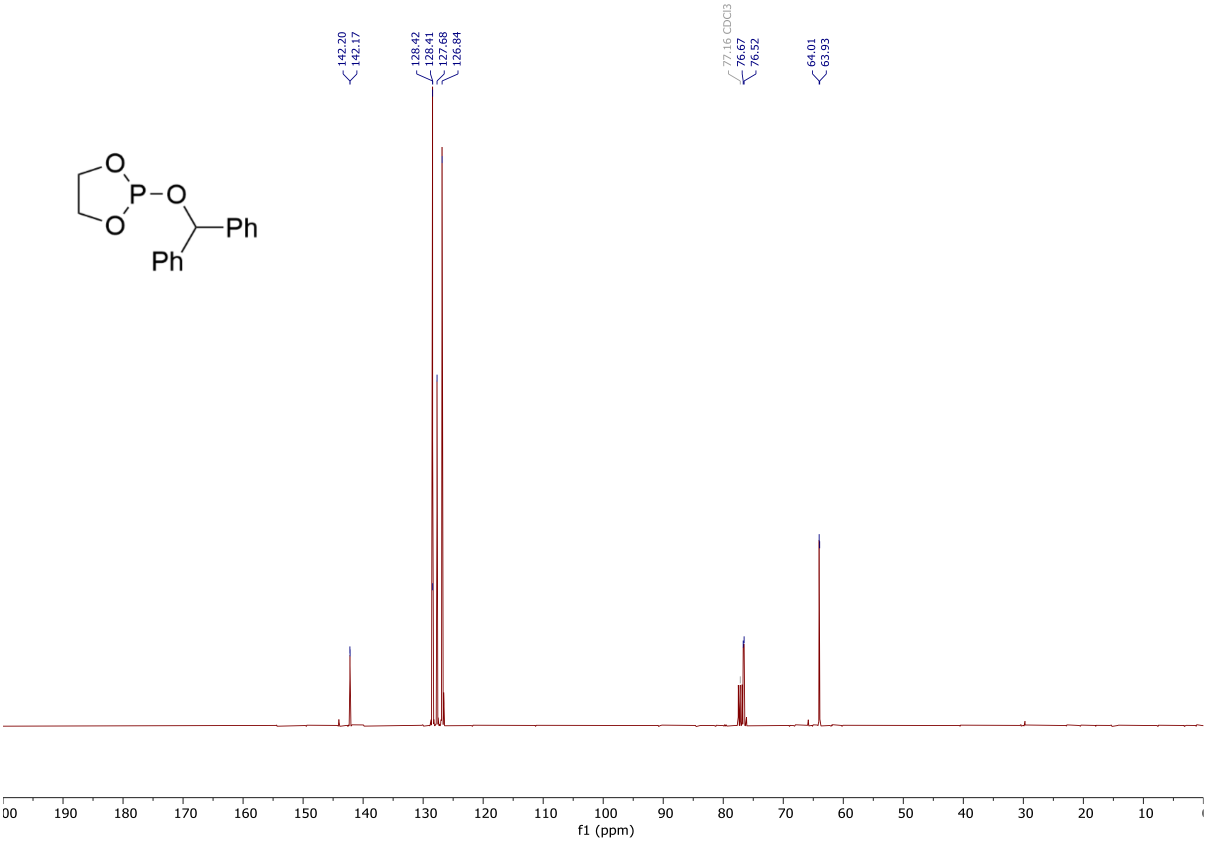

^{31}**P NMR** (162 MHz, $CDCl_3$): **2e**

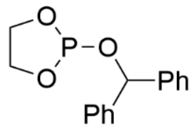

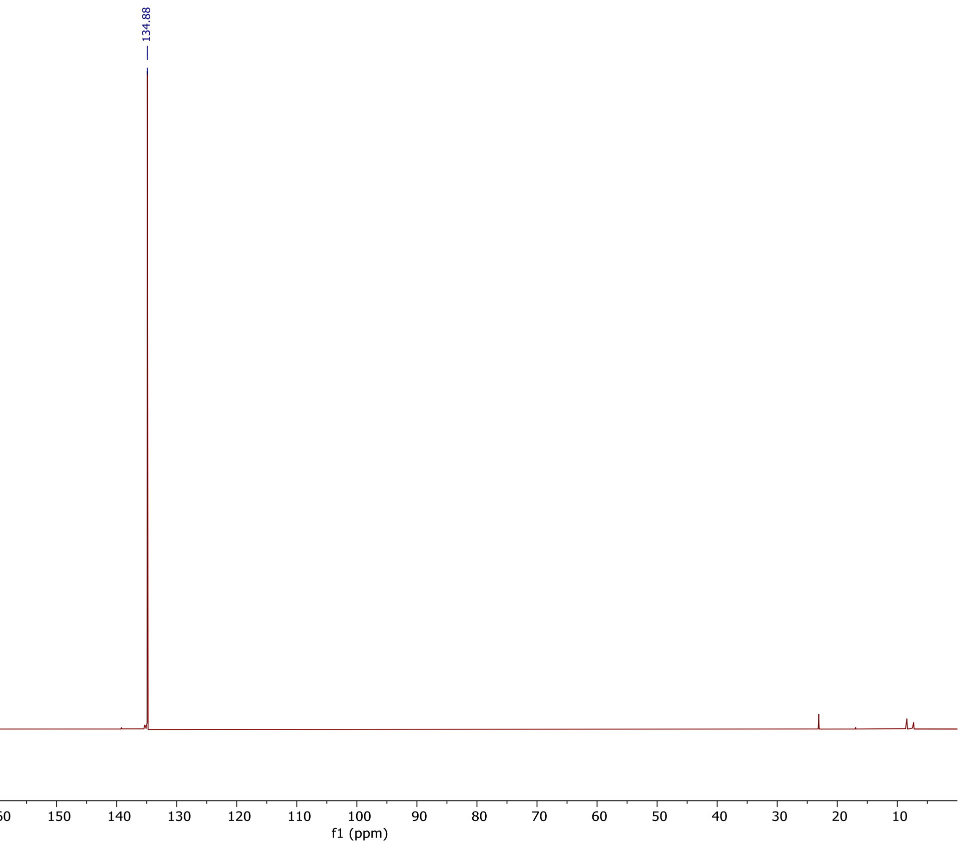

^{1}H NMR (400 MHz, $CDCl_3$): **2f**

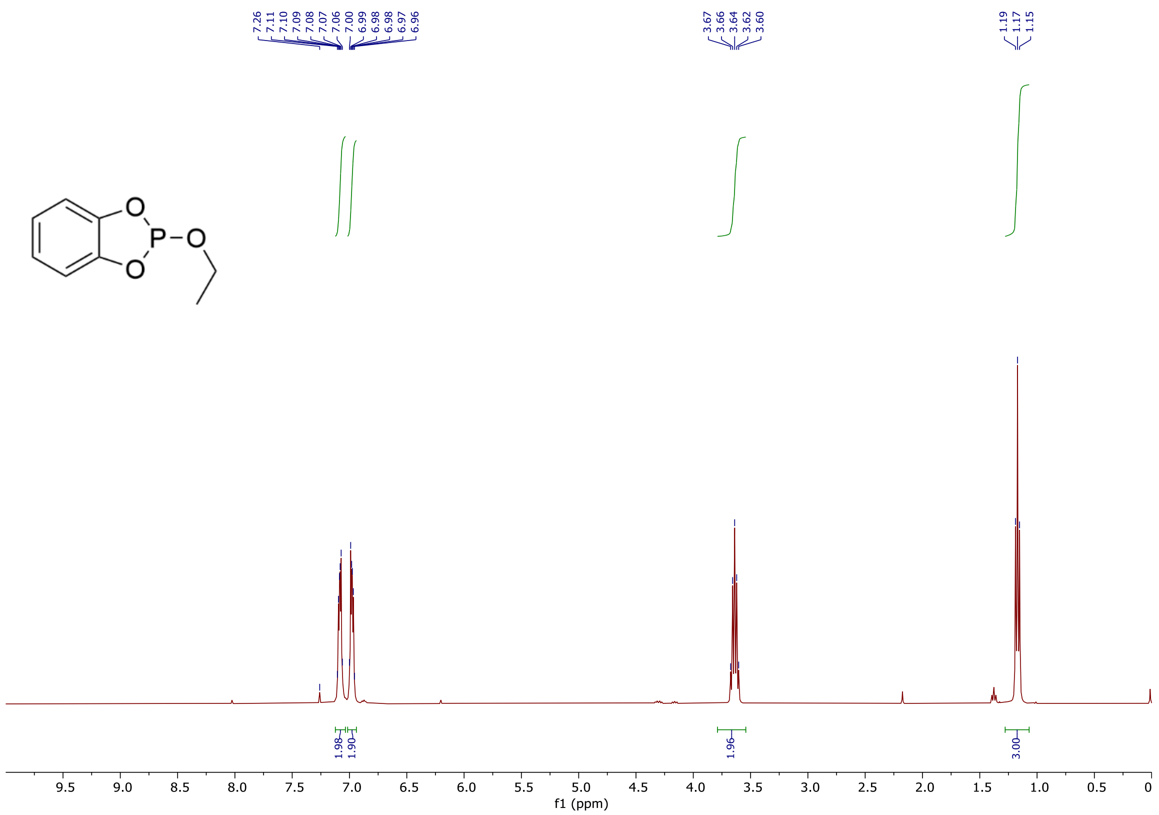

^{13}C NMR (101 MHz, $CDCl_3$): **2f**

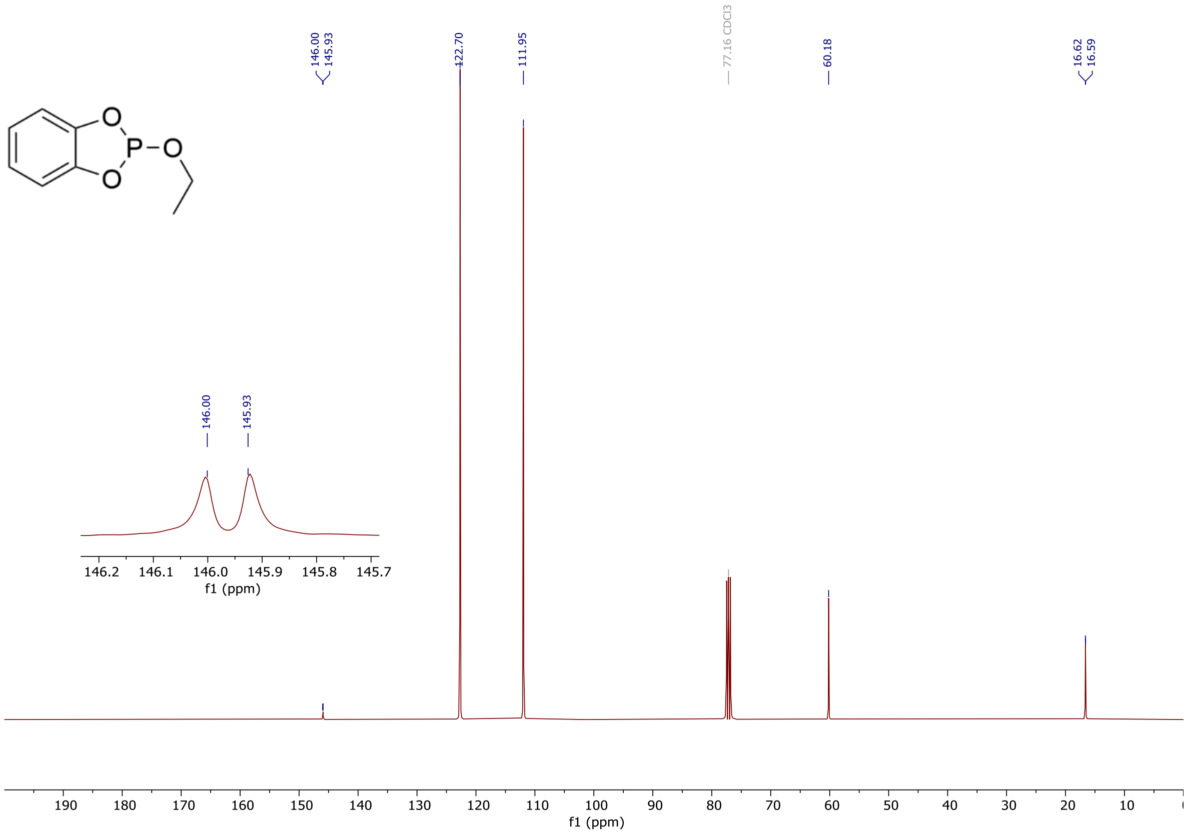

^{31}P NMR (162 MHz, $CDCl_3$): **2f**

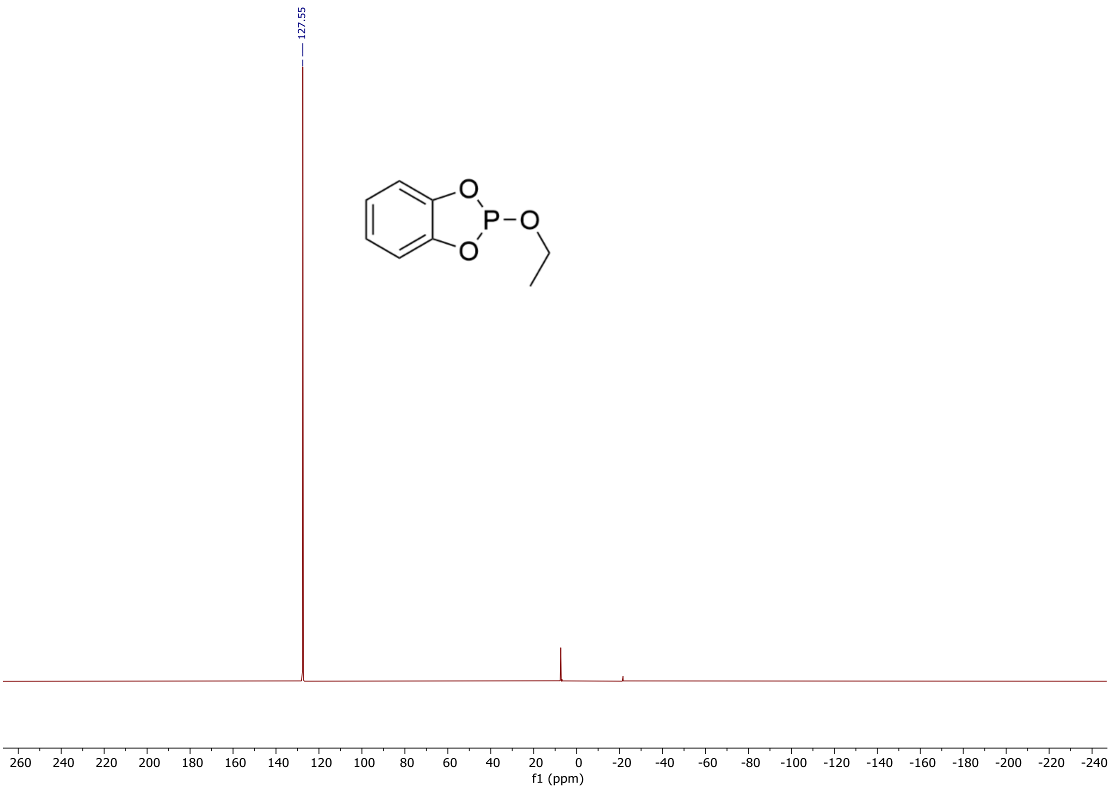

¹H NMR (400 MHz, $CDCl_3$): **2g**

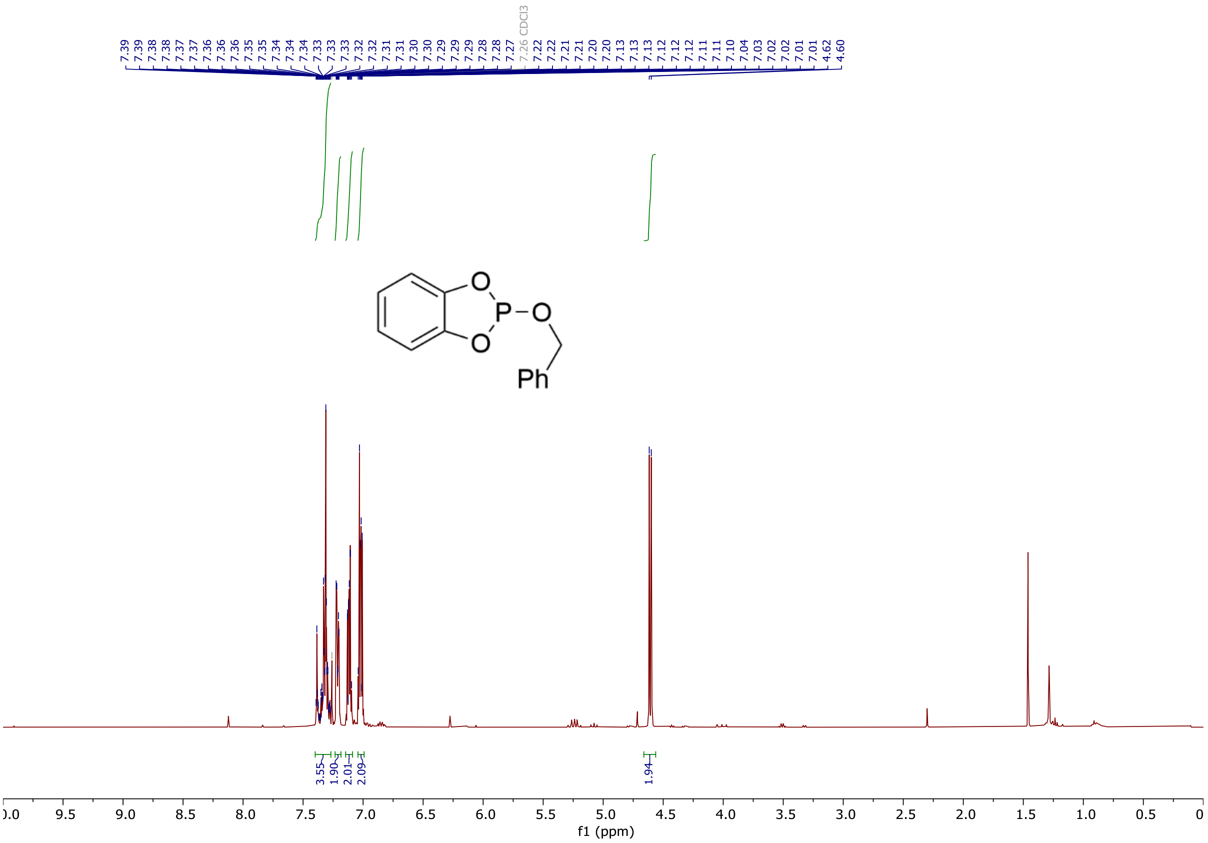

¹³C NMR (101 MHz, $CDCl_3$): **2g**

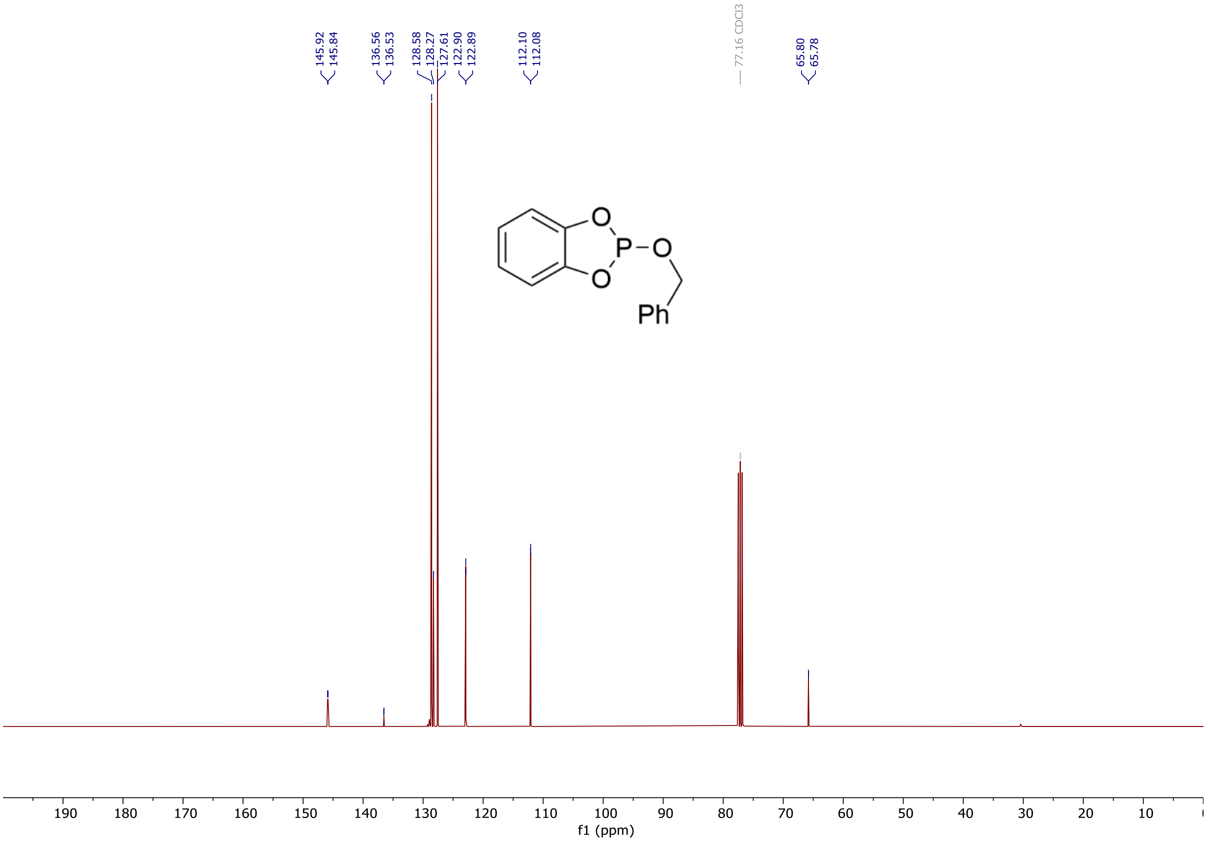

^{31}P NMR (162 MHz, CDCl$_3$): **2g**

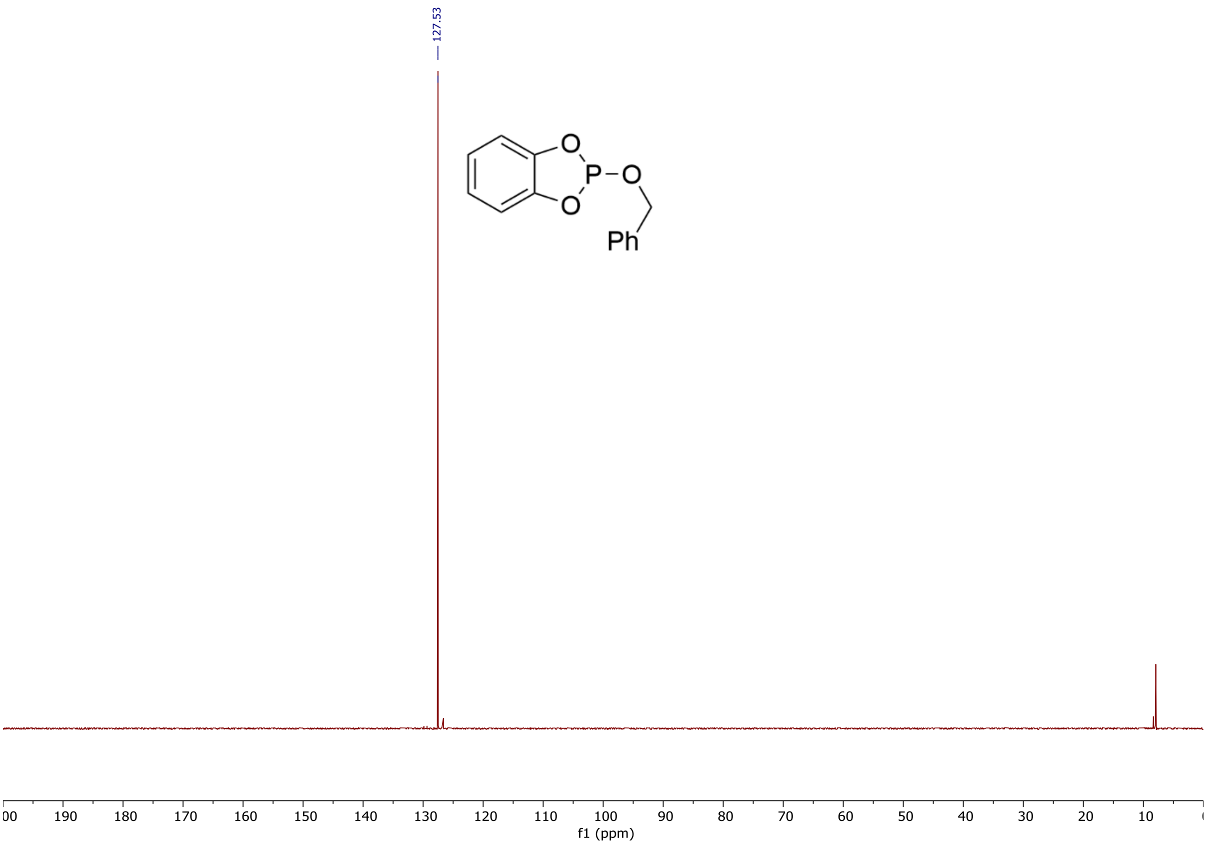

^{1}H NMR (400 MHz, $CDCl_3$): **2i**

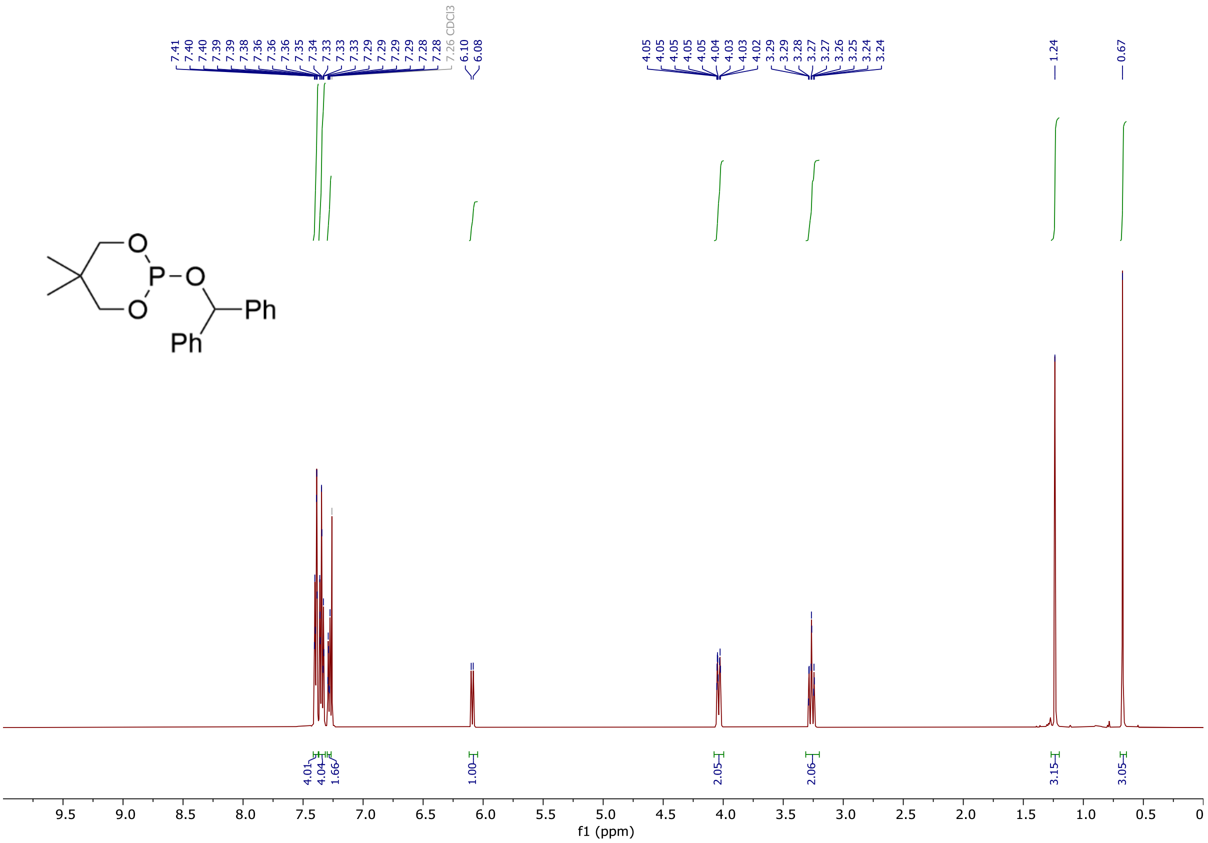

^{13}C NMR (101 MHz, $CDCl_3$): **2i**

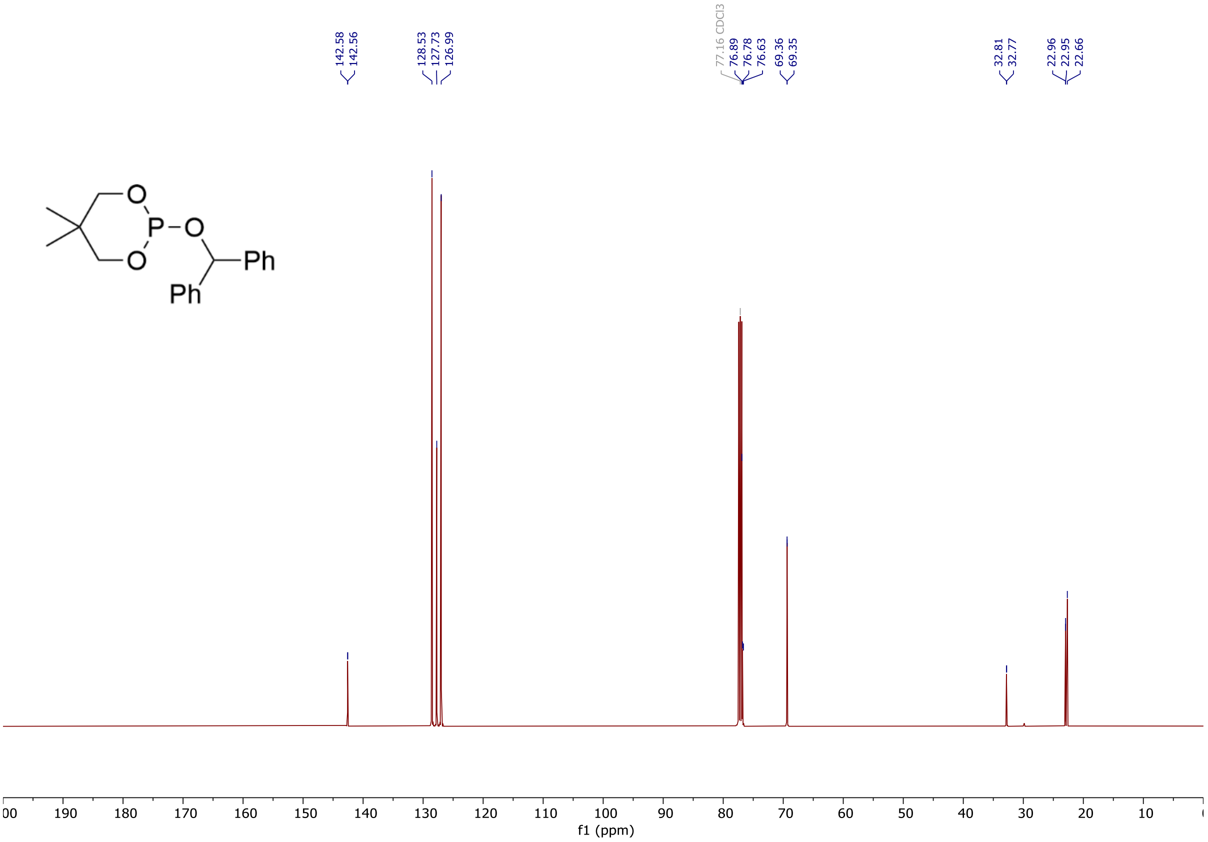

^{31}P NMR (162 MHz, $CDCl_3$): **2i**

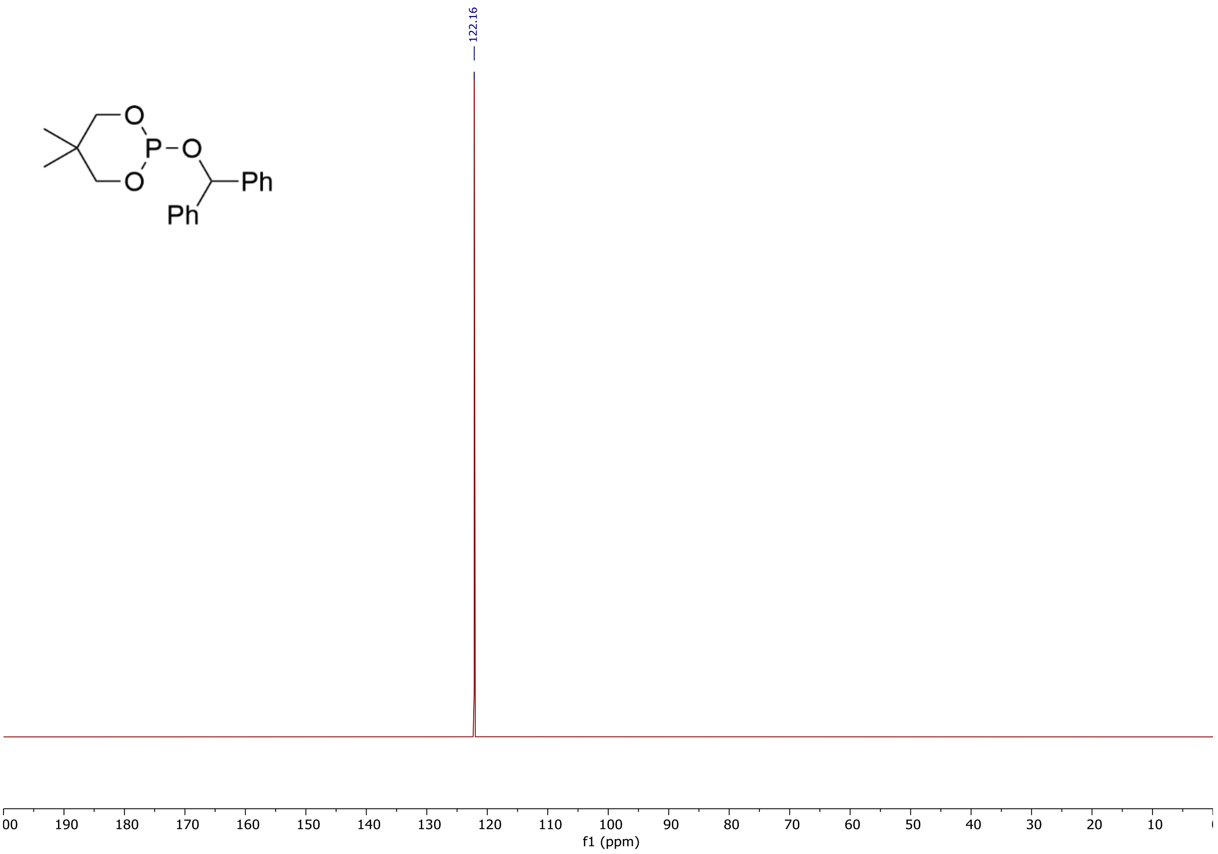

^{1}H NMR (400 MHz, $CDCl_3$): **2j**

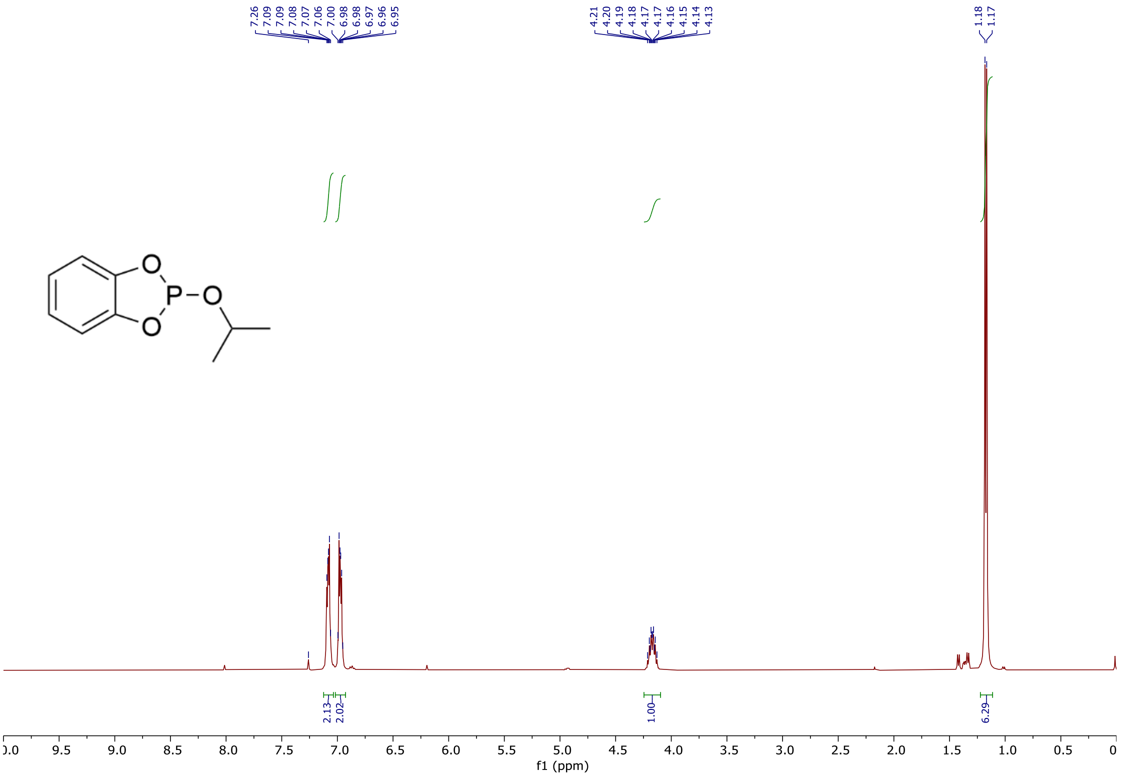

^{13}C NMR (101 MHz, $CDCl_3$): **2j**

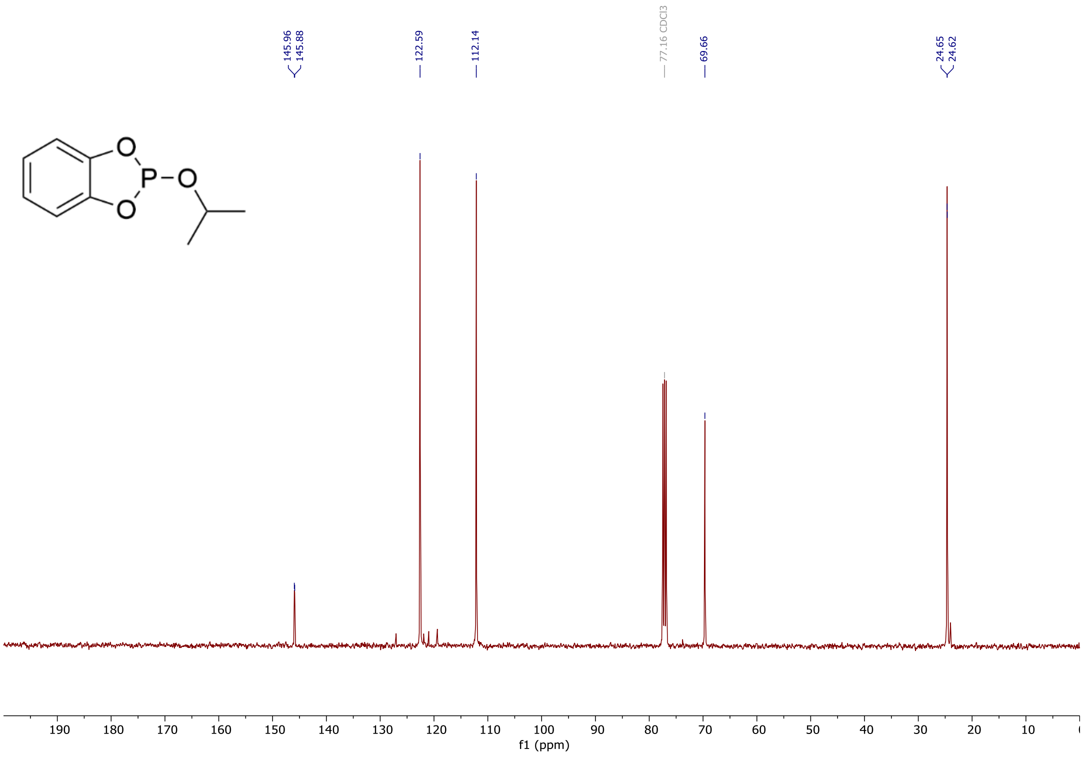

^{31}P NMR (162 MHz, $CDCl_3$): **2j**

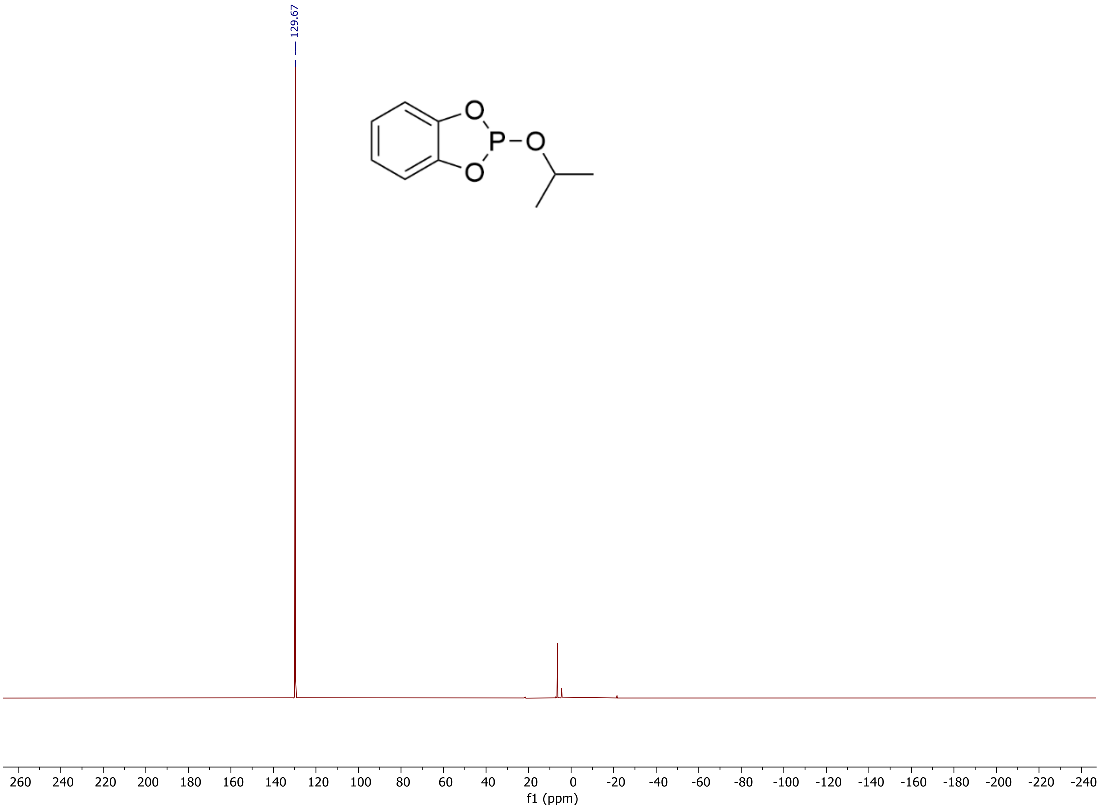

^{1}H NMR (400 MHz, CDCl$_3$): **2k**

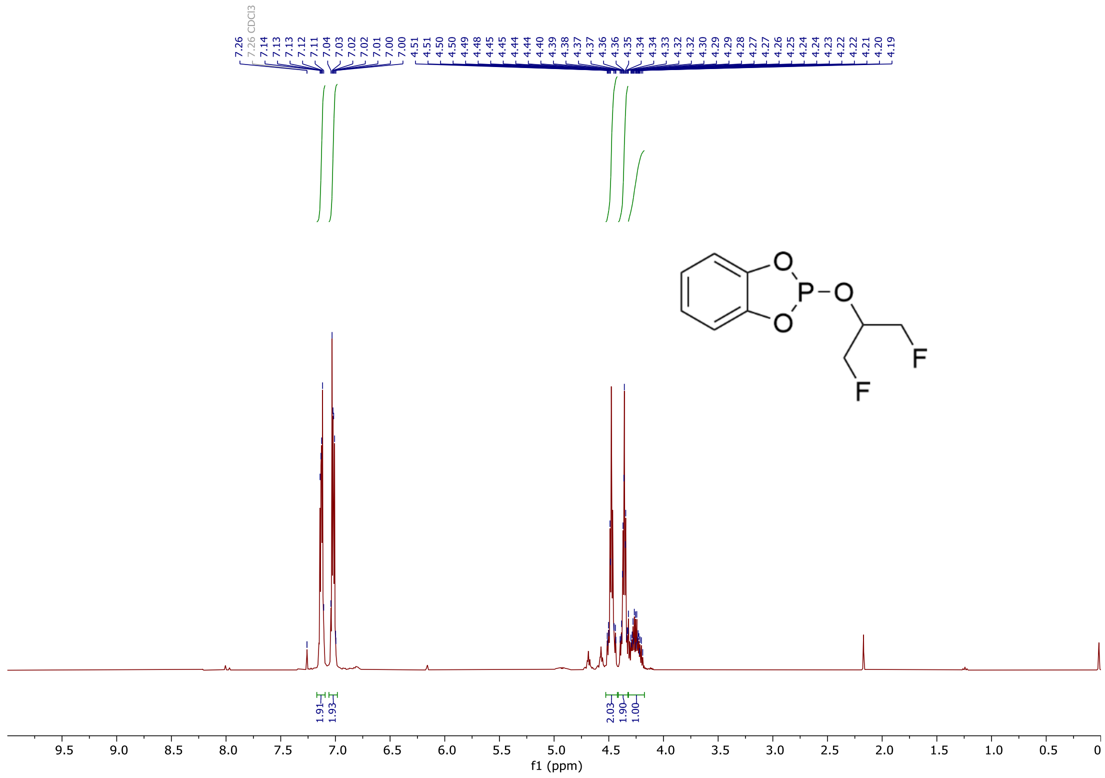

^{13}C NMR (101 MHz, CDCl$_3$): **2k**

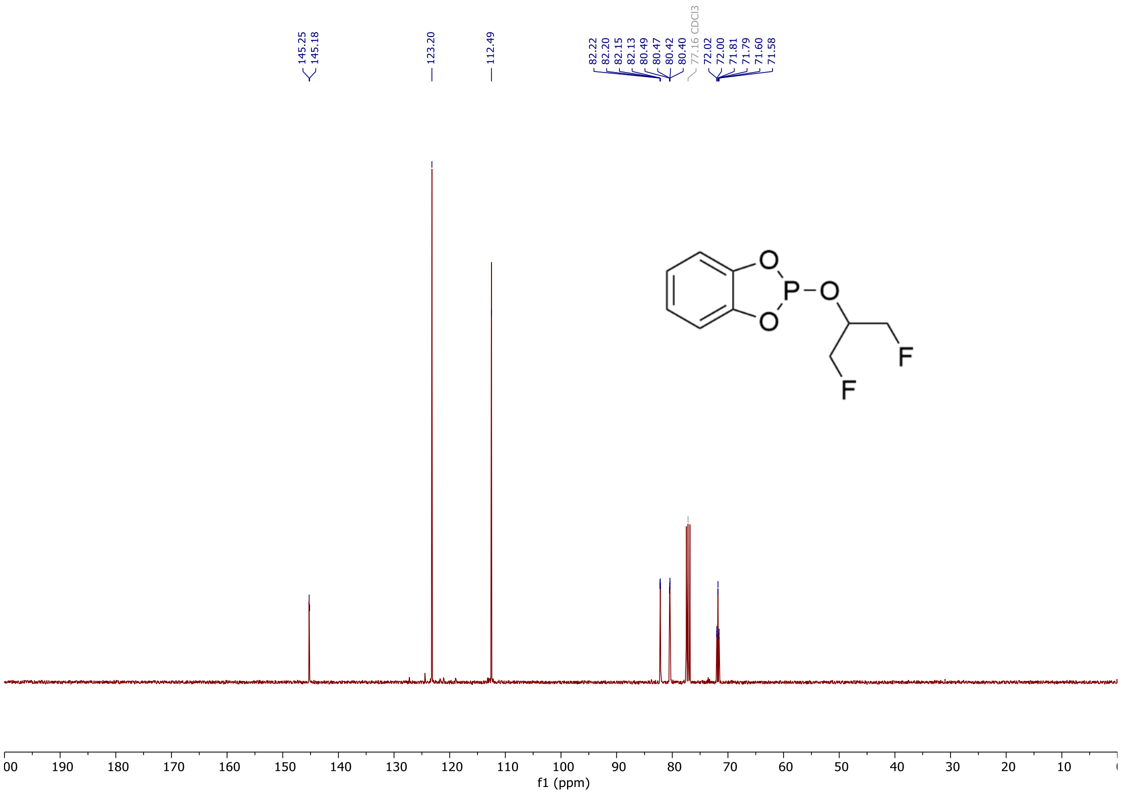

^{31}P NMR (162 MHz, $CDCl_3$): **2k**

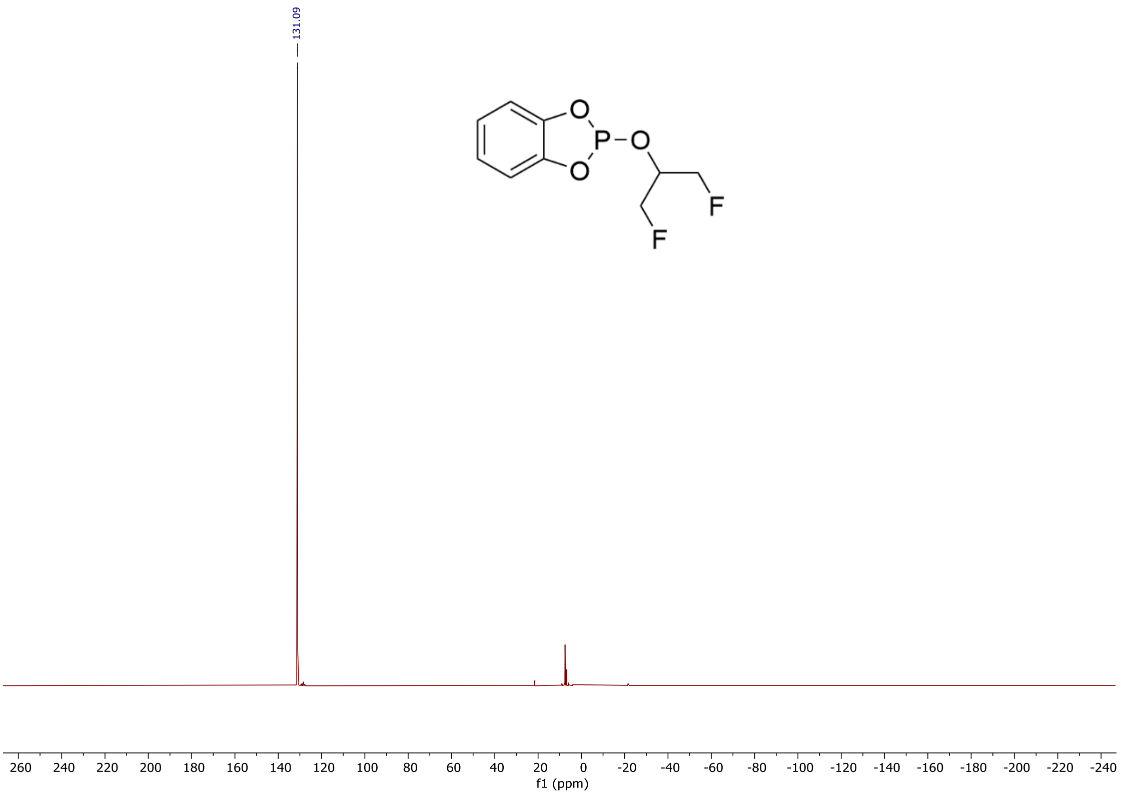

^{19}F NMR (377 MHz, $CDCl_3$: **2k**

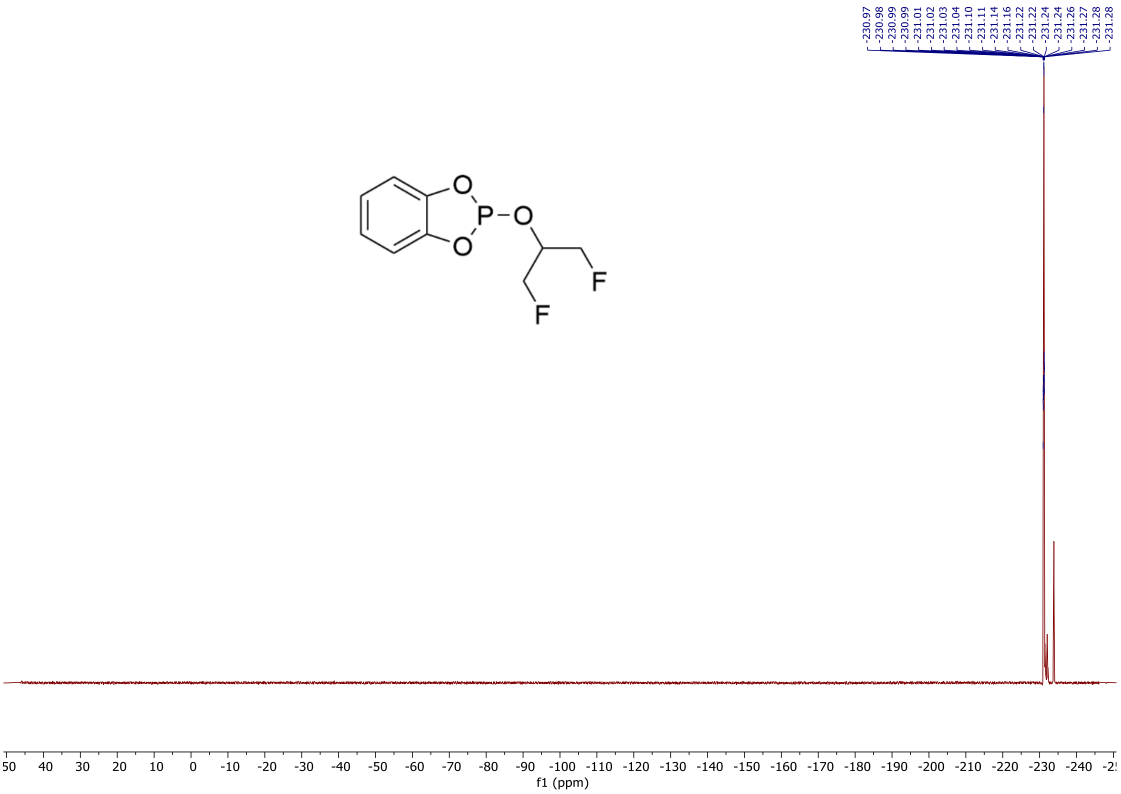

^{1}H NMR (400 MHz, CDCl3): **2l**

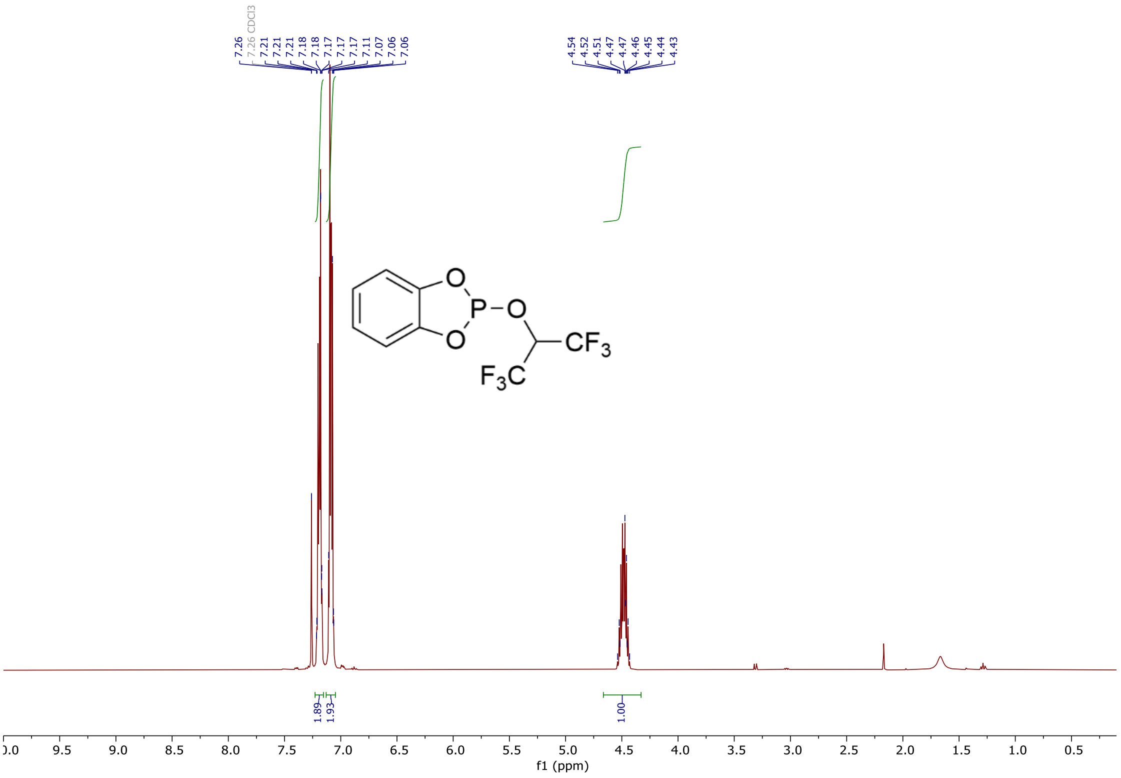

^{13}C NMR (101 MHz, CDCl3): **2l**

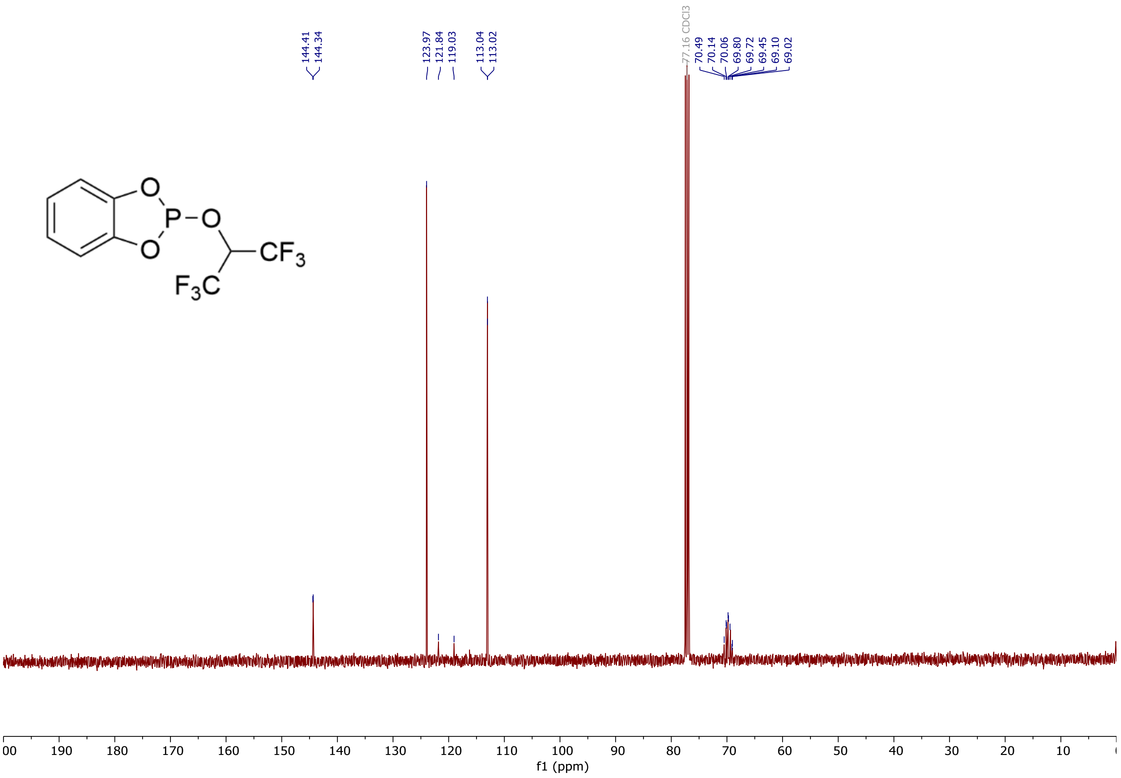

^{31}P NMR (162 MHz, $CDCl_3$): **2l**

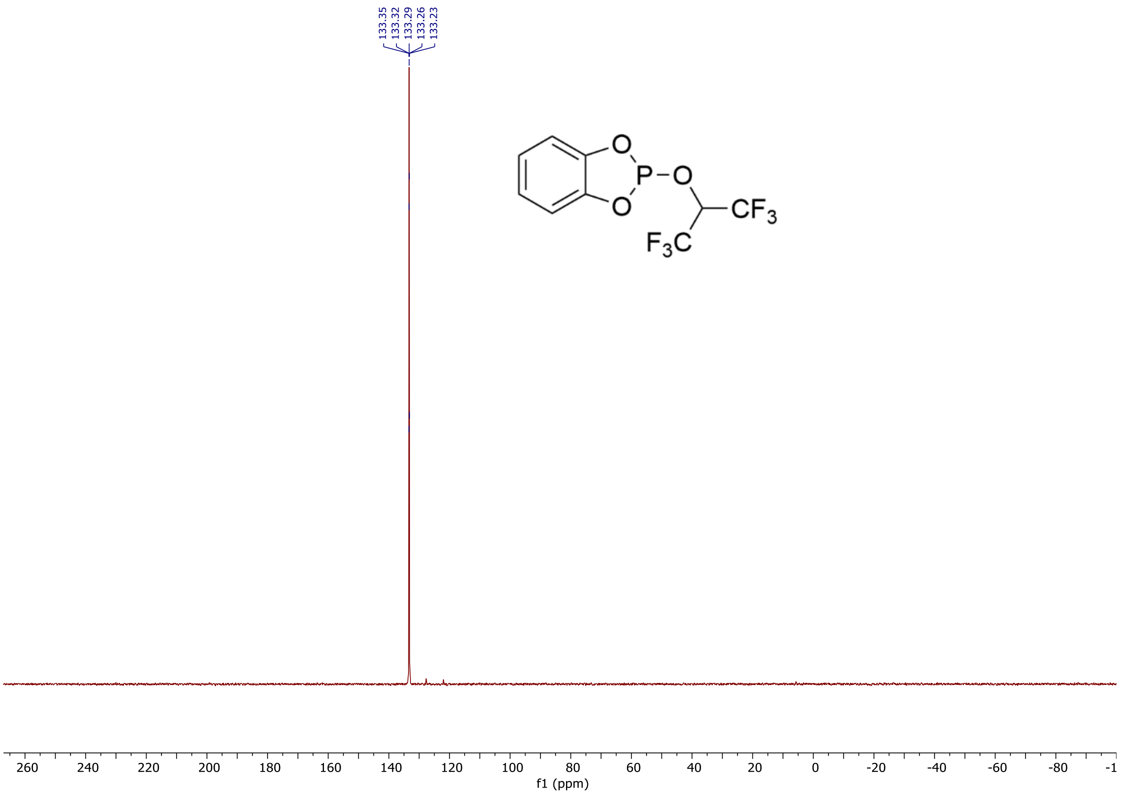

^{19}F NMR (377 MHz, $CDCl_3$): **2l**

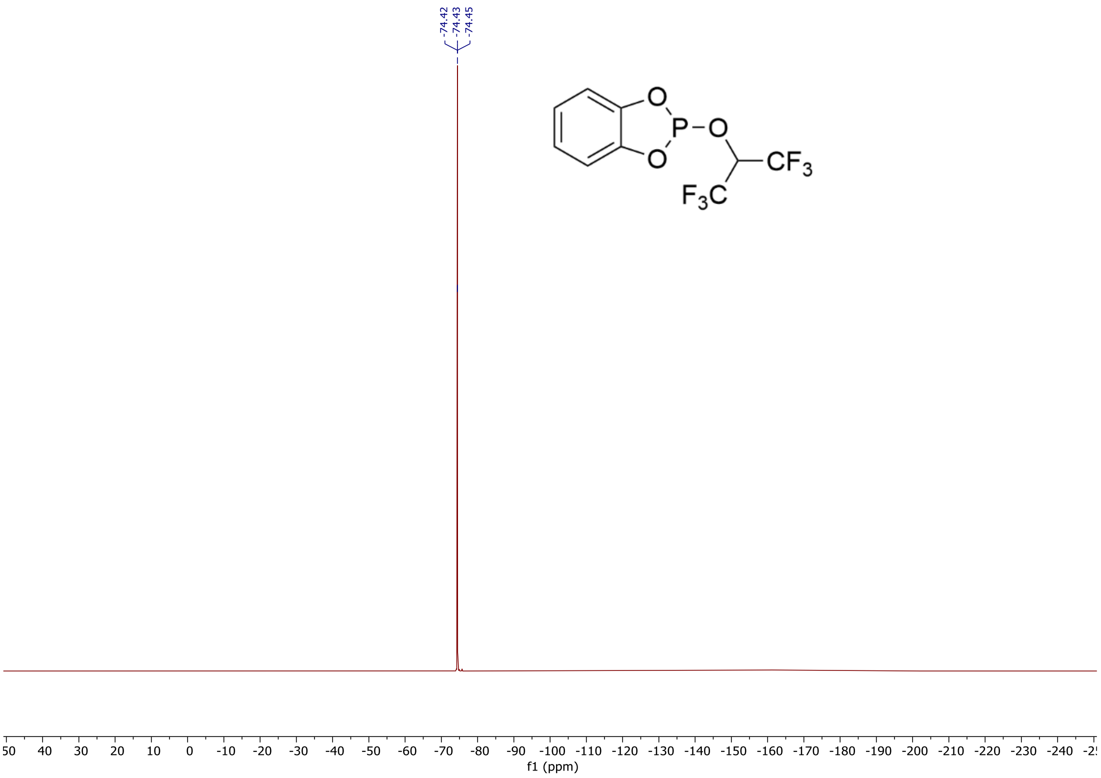

^{1}H NMR (400 MHz, $CDCl_3$): **2m**

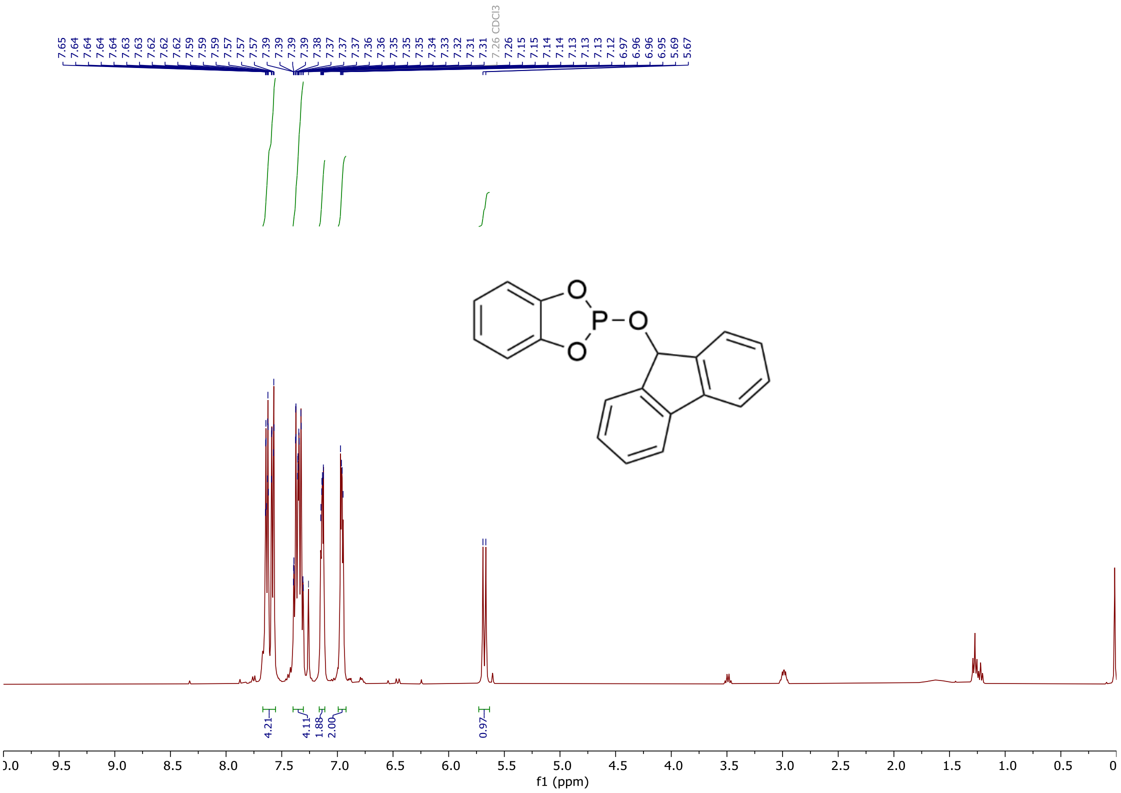

^{13}C NMR (101 MHz, $CDCl_3$): **2m**

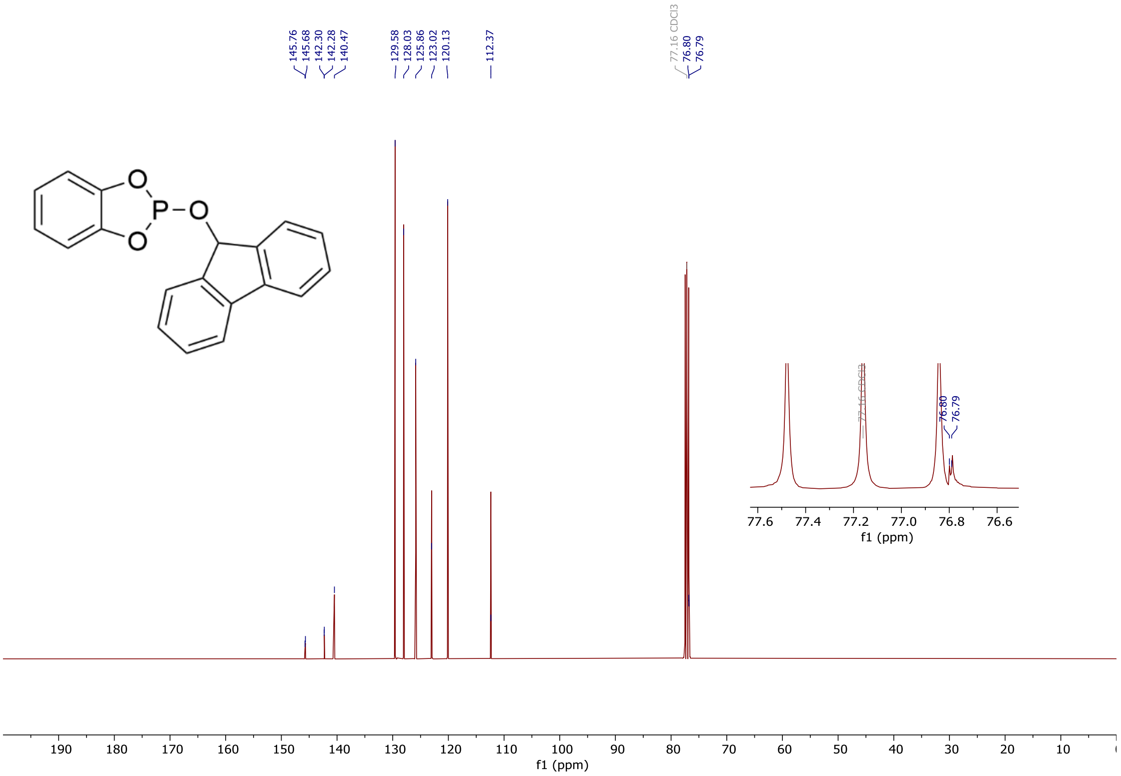

^{31}**P NMR** (162 MHz, $CDCl_3$): **2m**

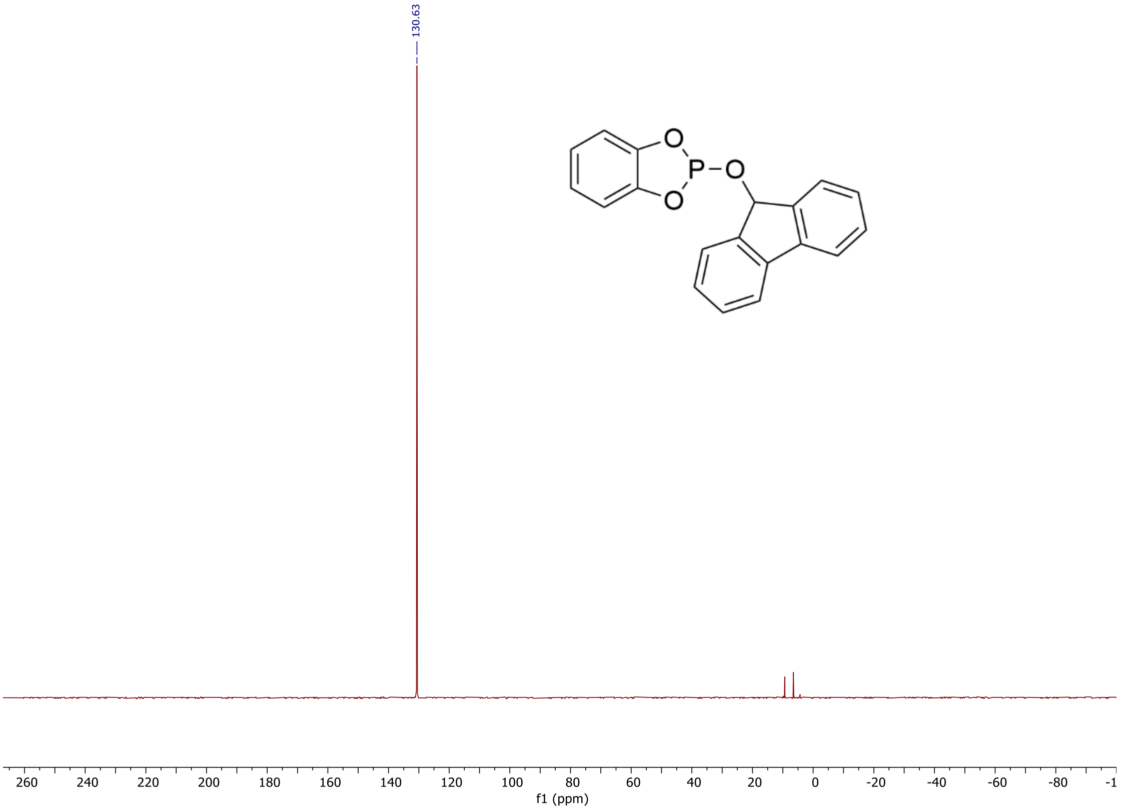

^{1}H NMR (400 MHz, $CDCl_3$): **3b**

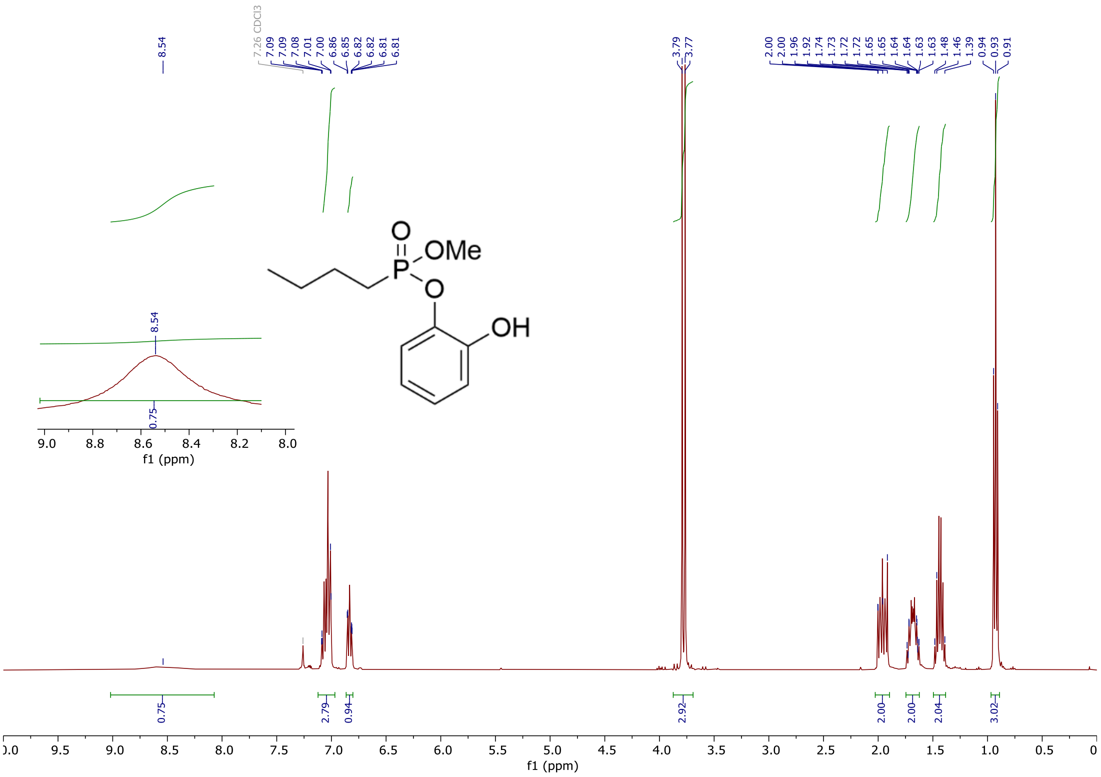

^{13}C NMR (101 MHz, $CDCl_3$): **3b**

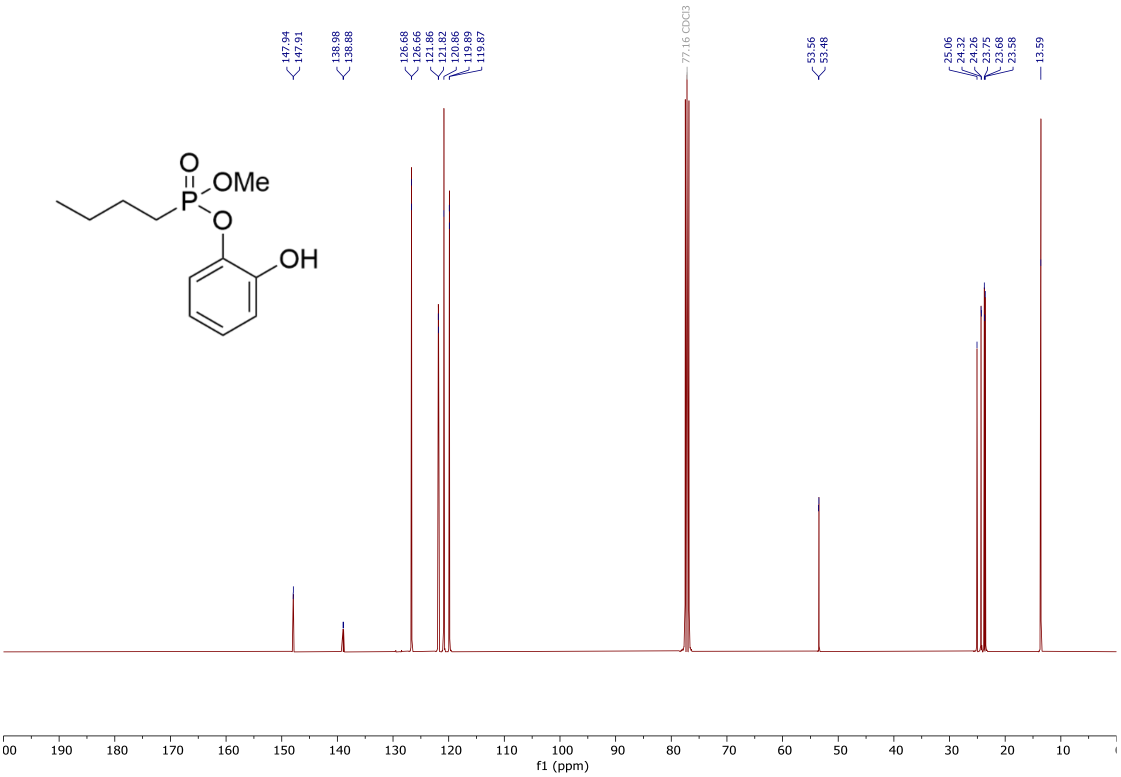

^{31}**P NMR** (162 MHz, $CDCl_3$): **3b**

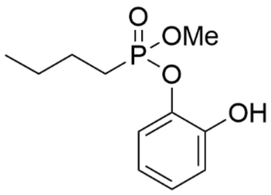

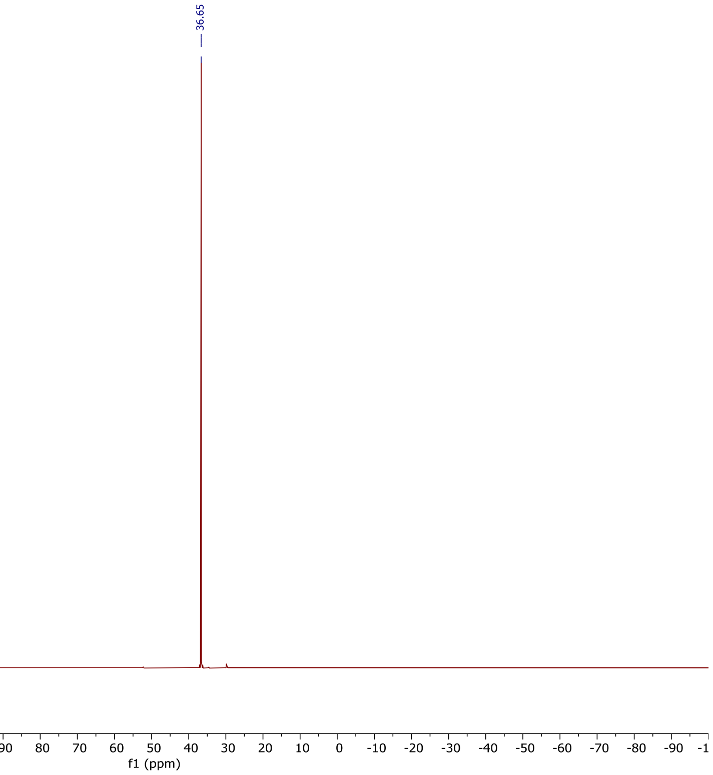

^{1}H NMR (400 MHz, $CDCl_3$): **3c**

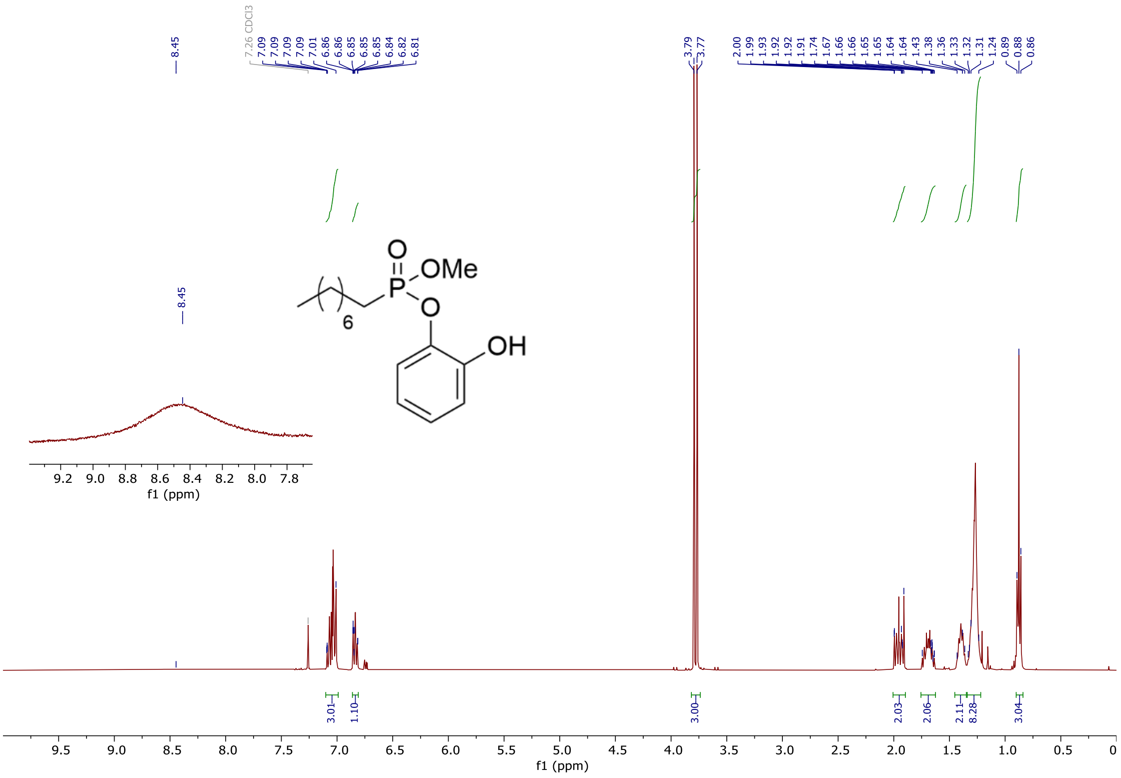

^{13}C NMR (101 MHz, $CDCl_3$): **3c**

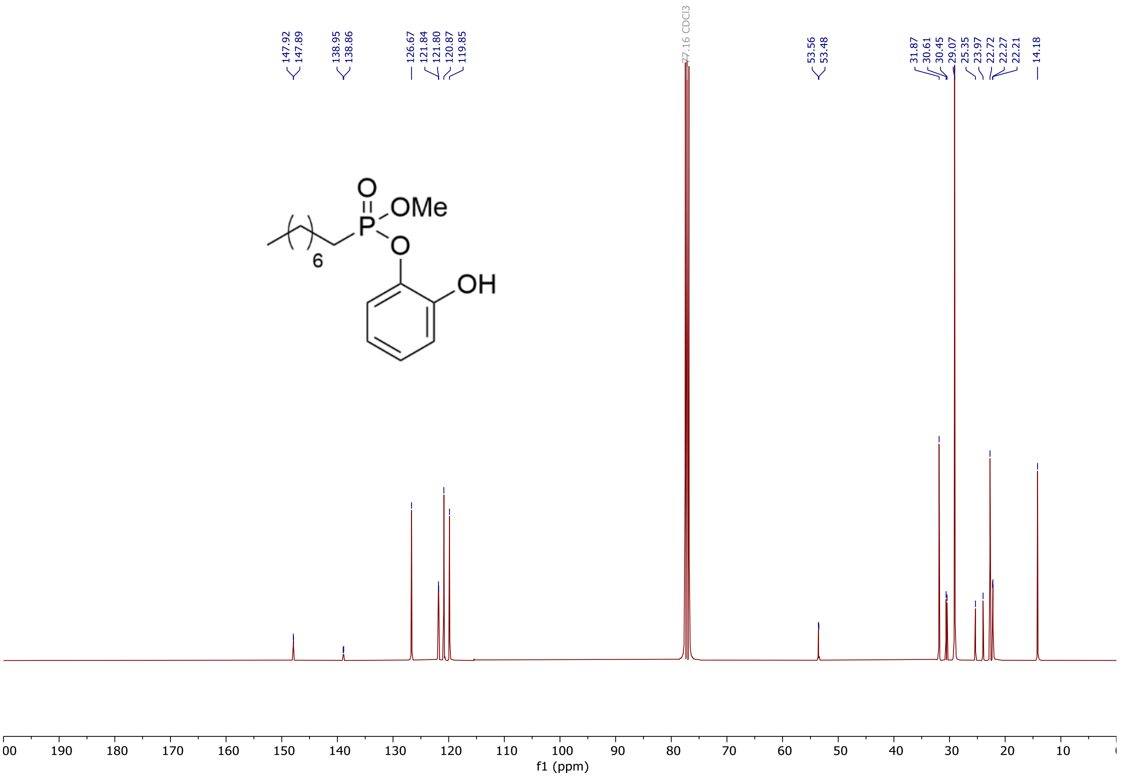

^{31}P NMR (162 MHz, $CDCl_3$): **3c**

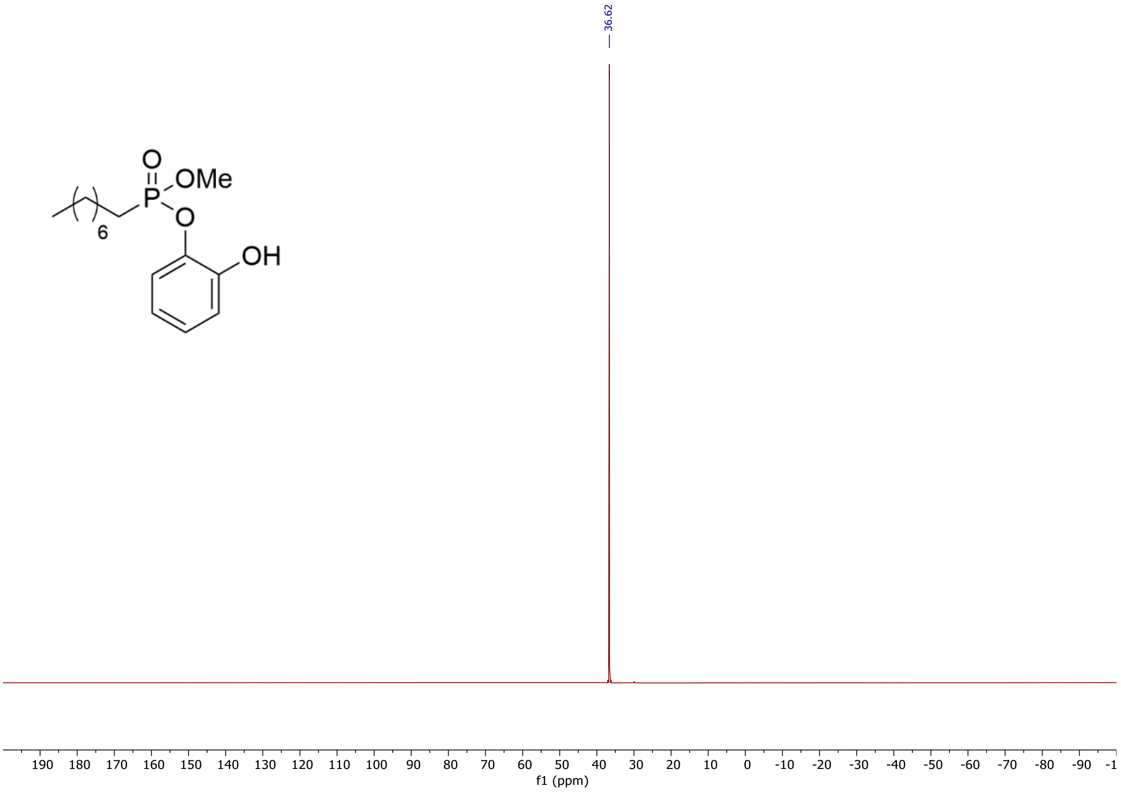

^{1}H NMR (400 MHz, $CDCl_3$): **3d**

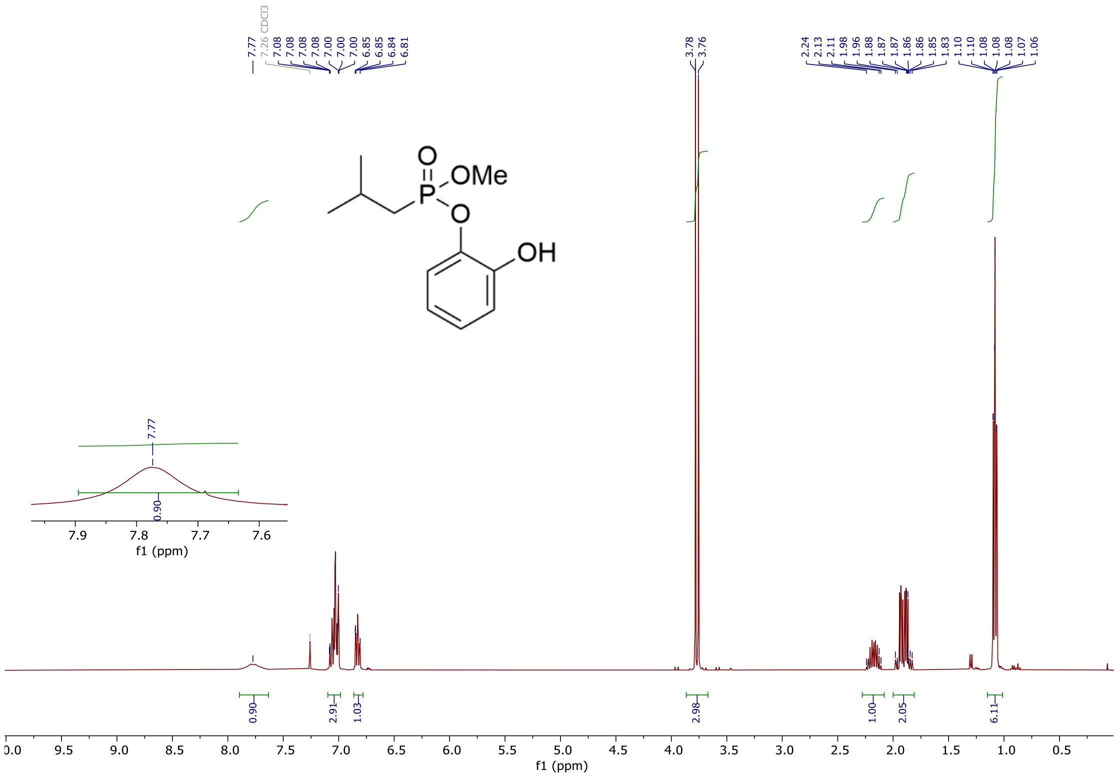

^{13}C NMR (101 MHz, $CDCl_3$): **3d**

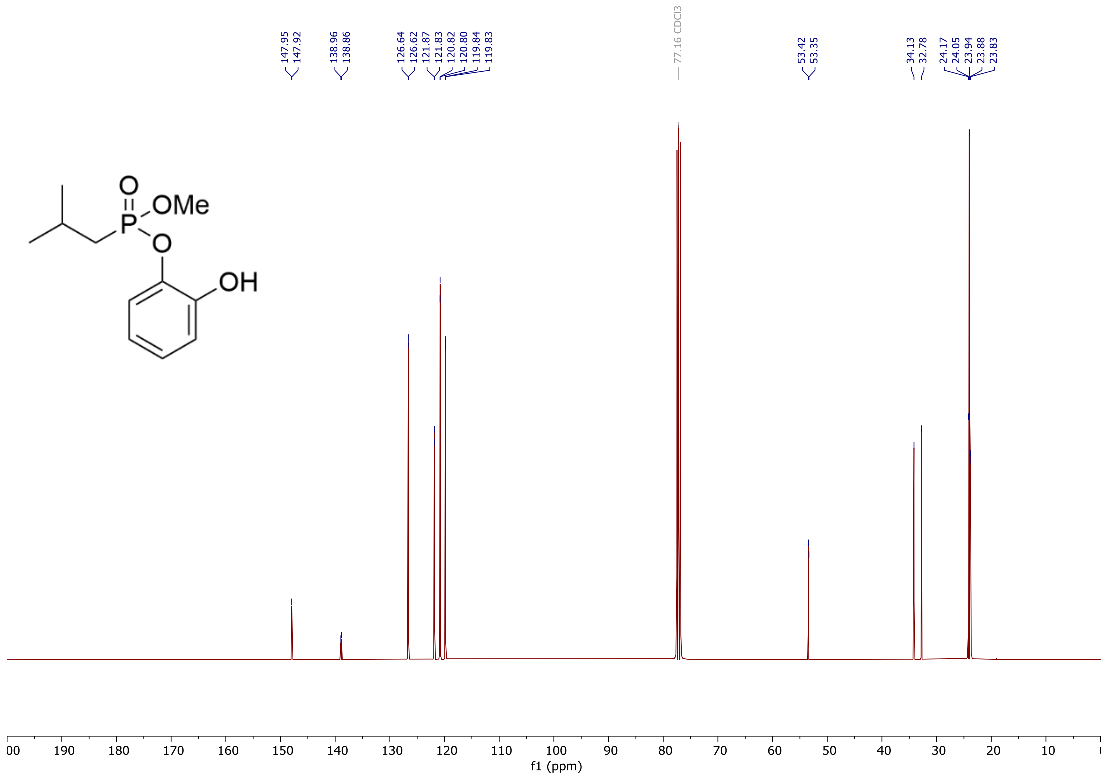

^{31}P NMR (162 MHz, $CDCl_3$): **3d**

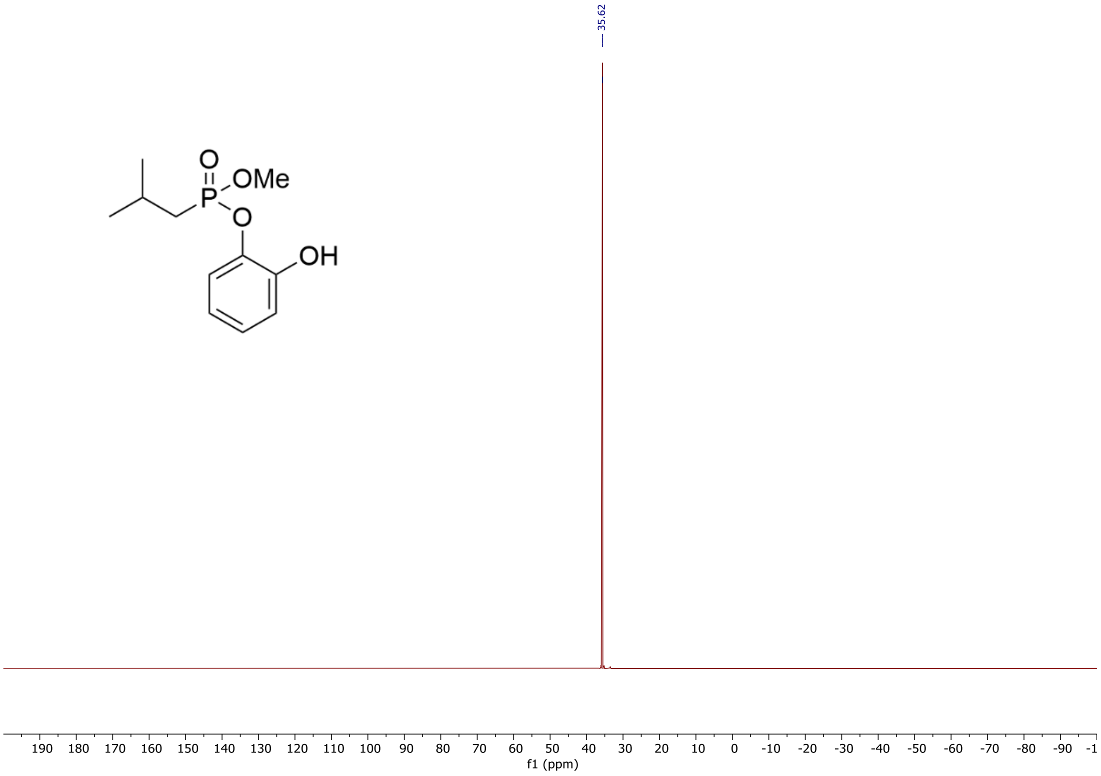

¹H NMR (400 MHz, $CDCl_3$): **3e**

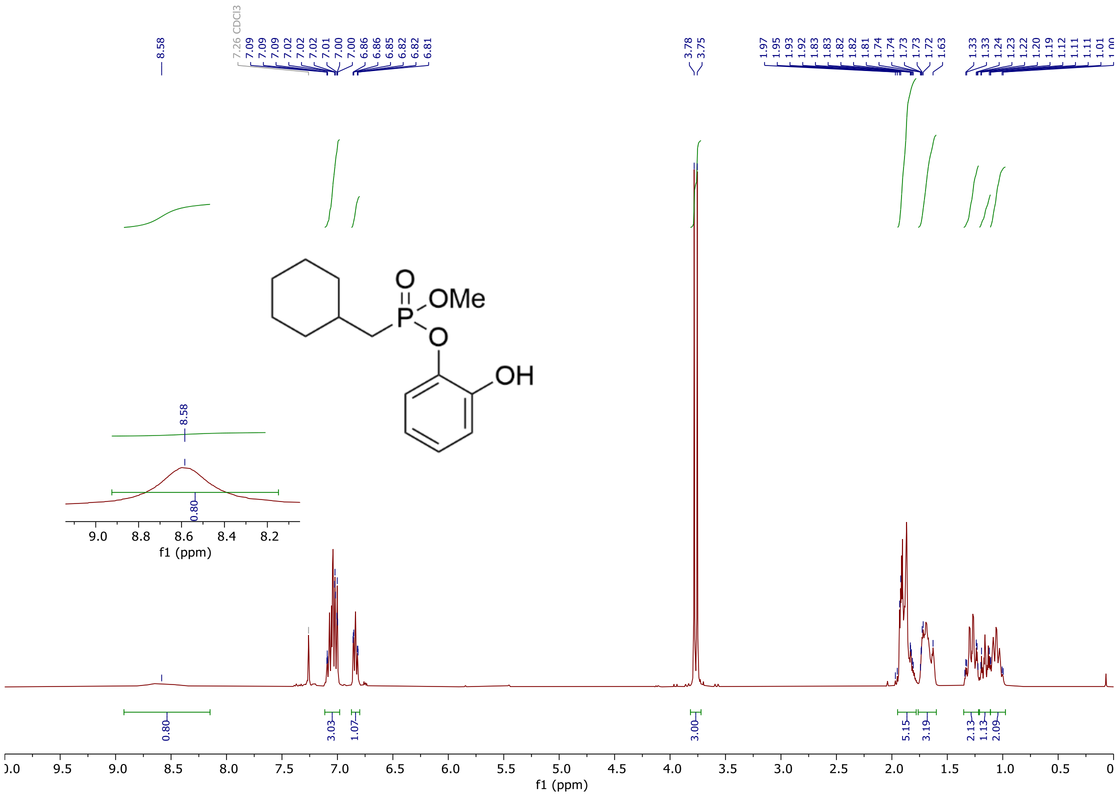

¹³C NMR (101 MHz, $CDCl_3$): **3e**

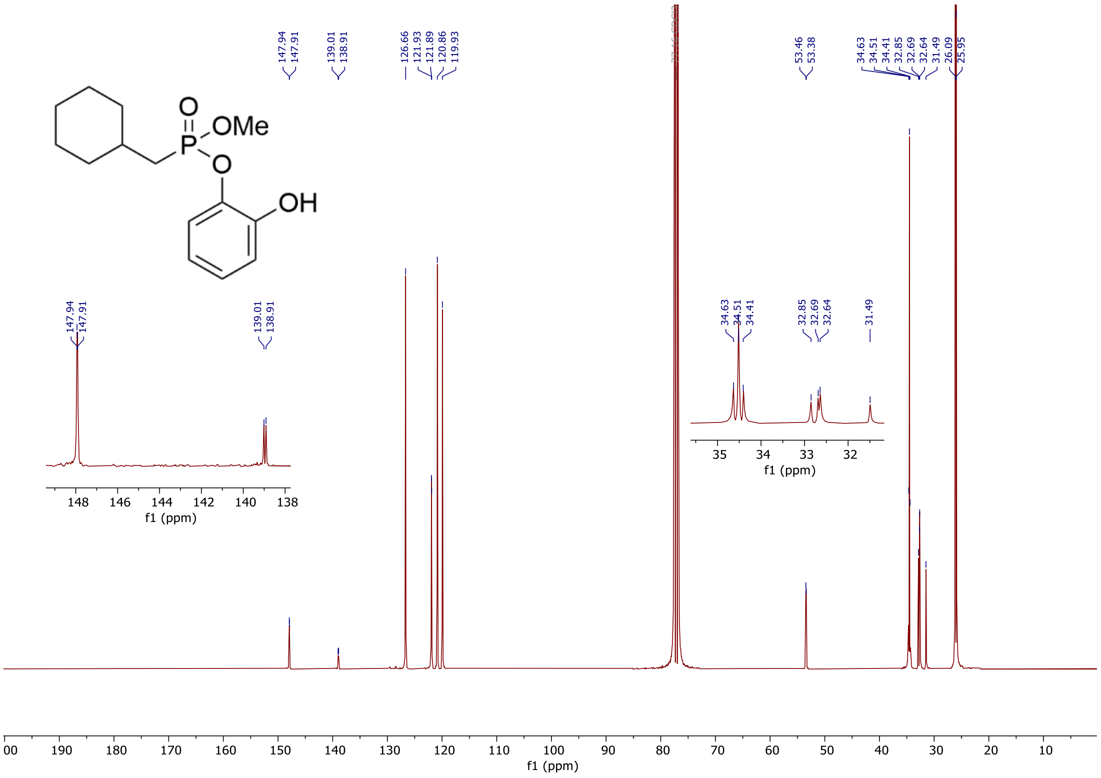

^{31}**P NMR** (162 MHz, $CDCl_3$): **3e**

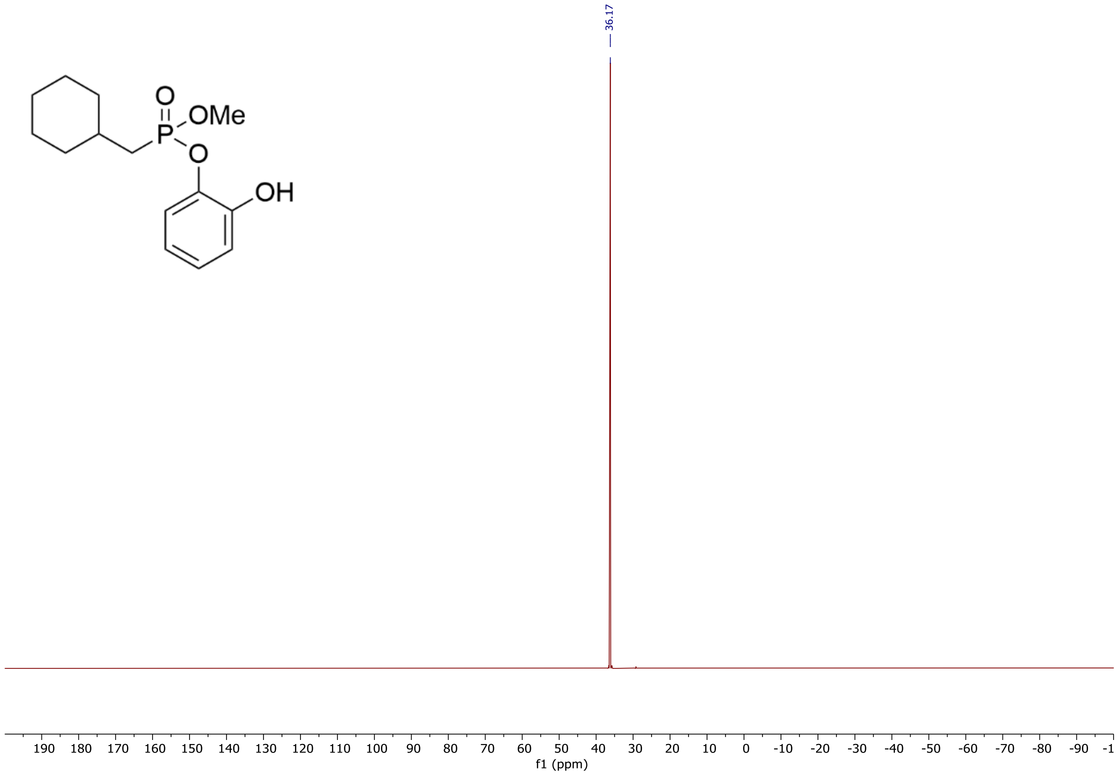

^{1}H NMR (400 MHz, $CDCl_3$): **3f**

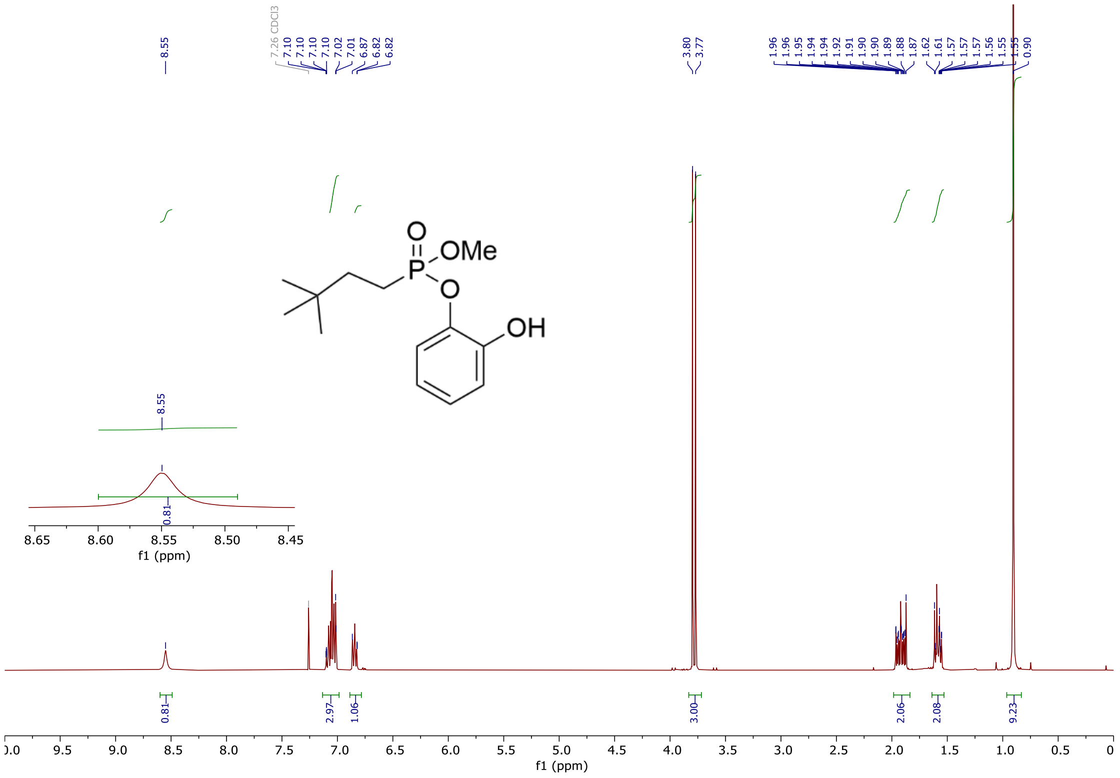

^{13}C NMR (101 MHz, $CDCl_3$): **3f**

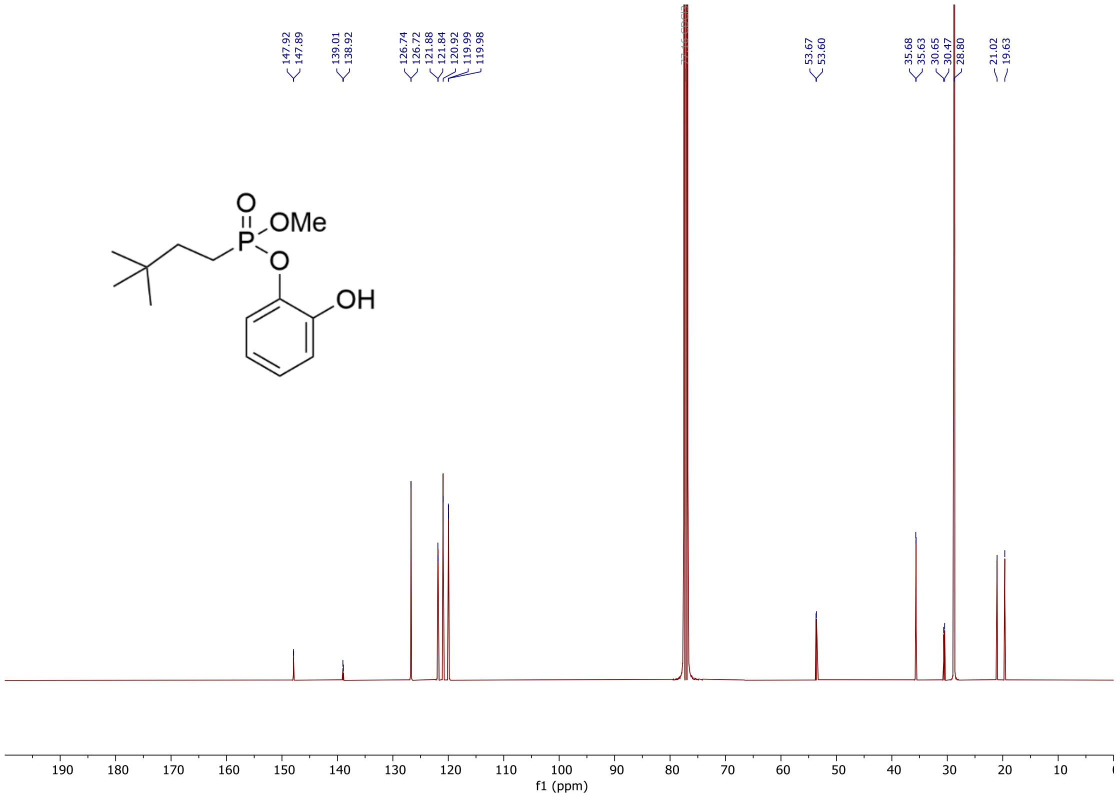

^{31}**P NMR** (162 MHz, $CDCl_3$): **3f**

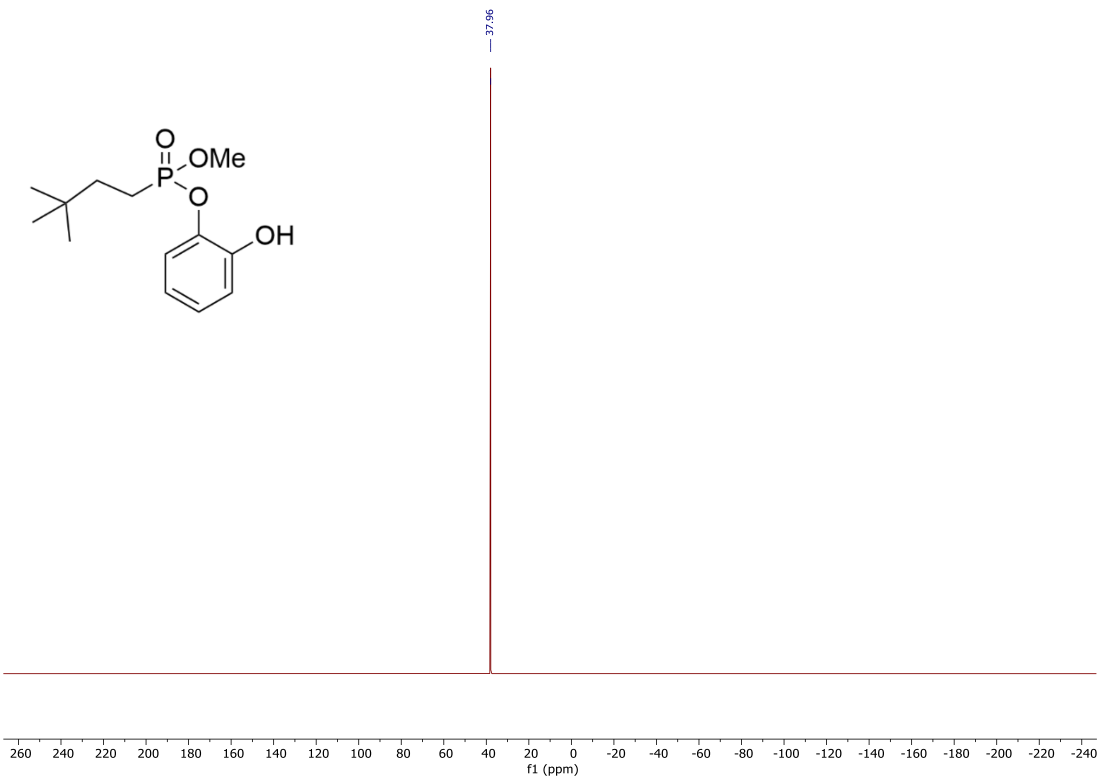

^{1}H NMR (400 MHz, $CDCl_3$): **3g**

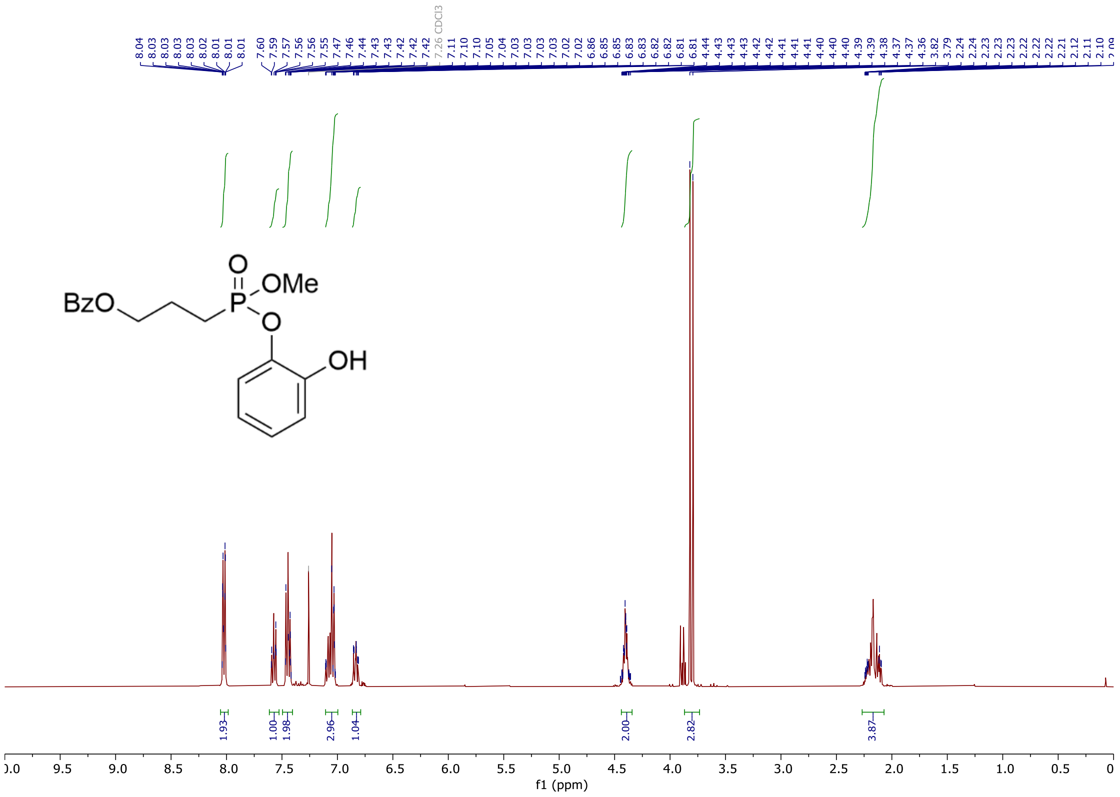

^{13}C NMR (101 MHz, $CDCl_3$): **3g**

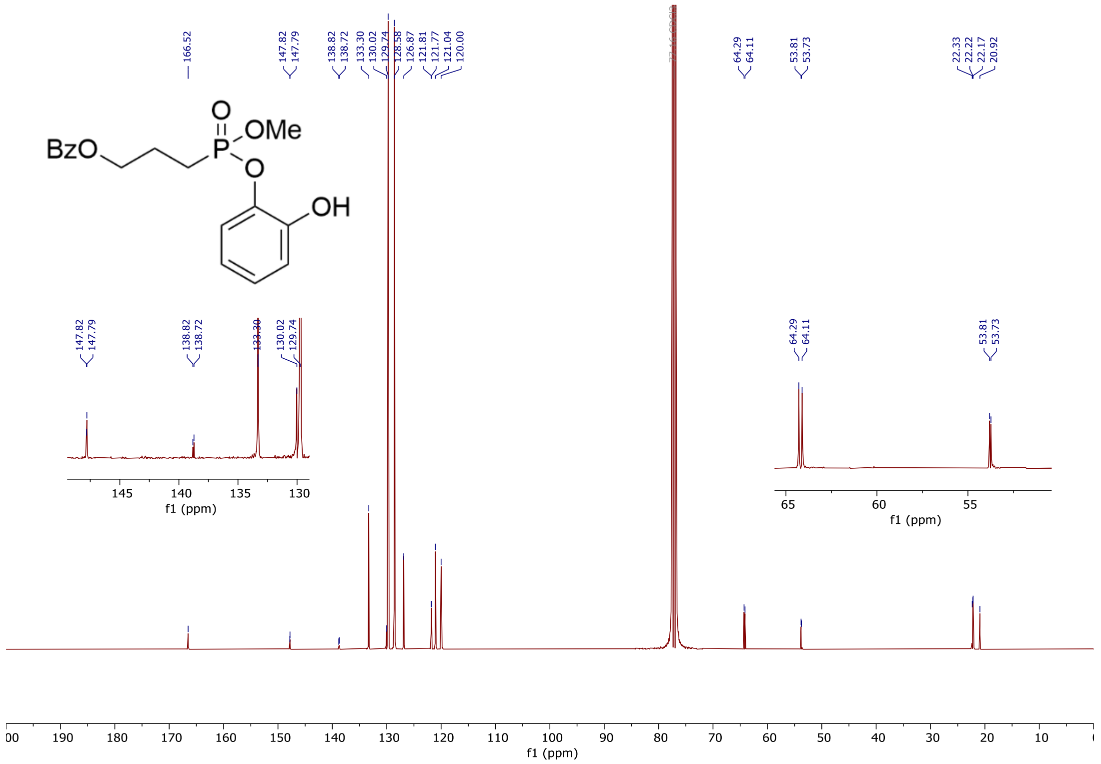

^{31}**P NMR** (162 MHz, $CDCl_3$): **3g**

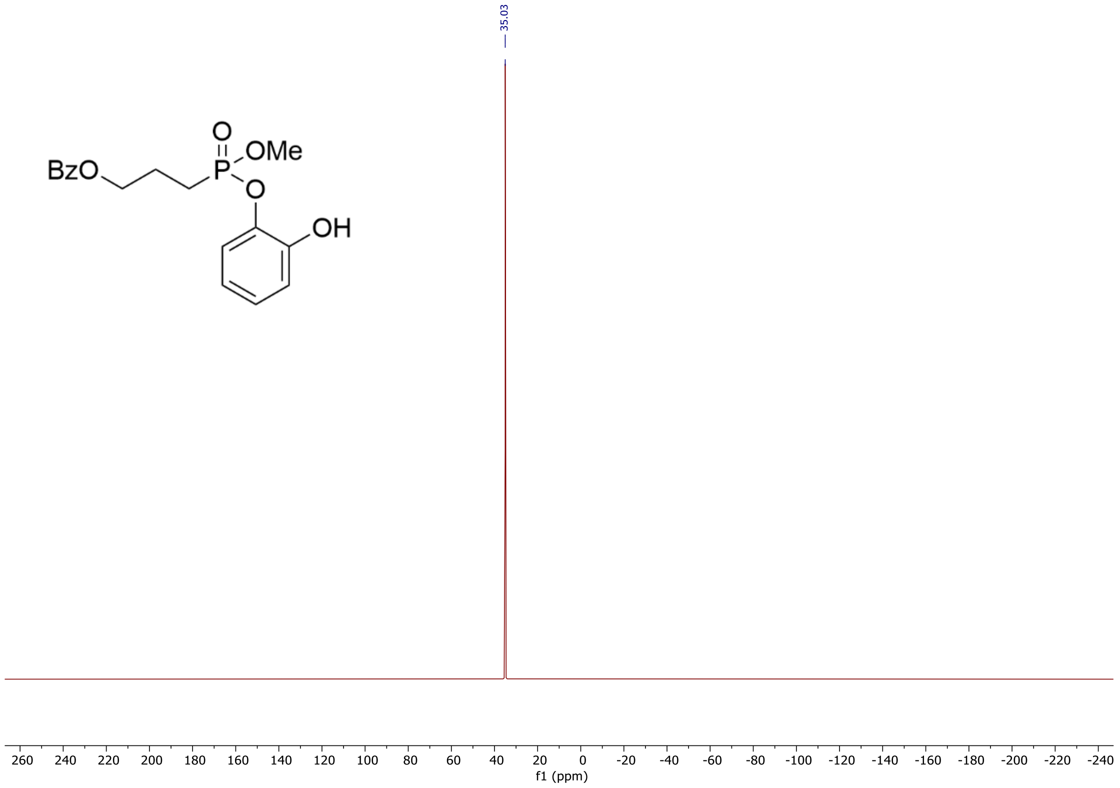

^{1}H NMR (400 MHz, $CDCl_3$): **3h**

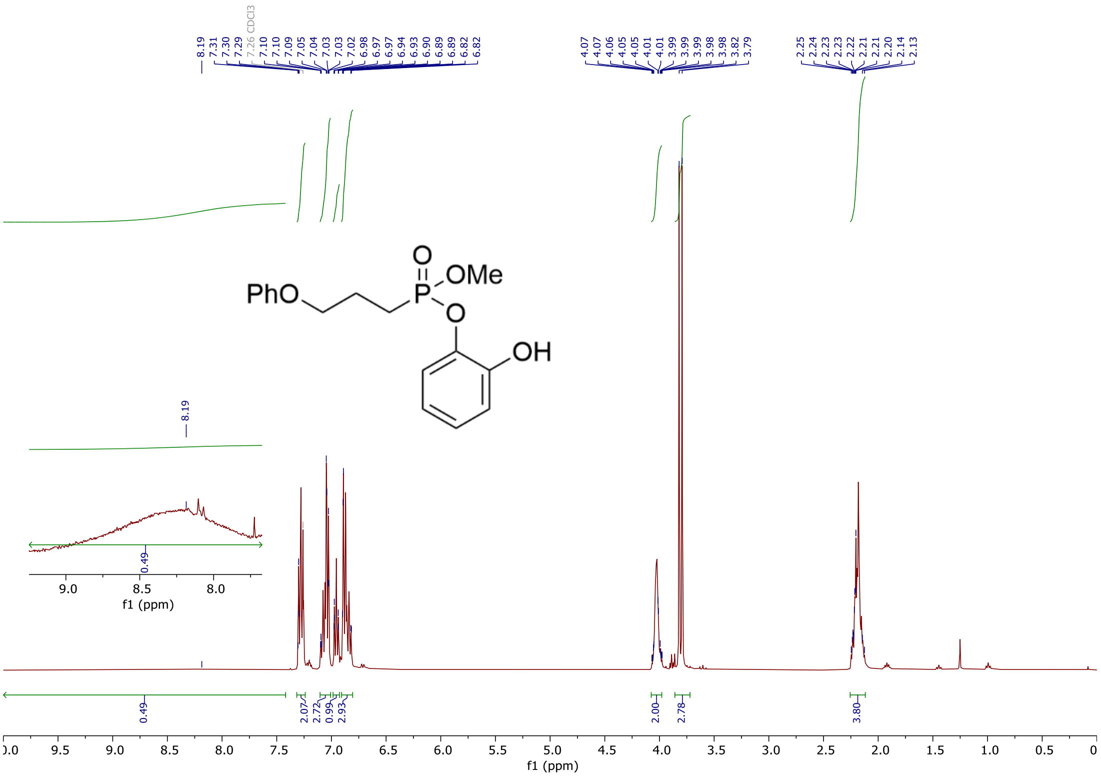

^{13}C NMR (101 MHz, $CDCl_3$): **3h**

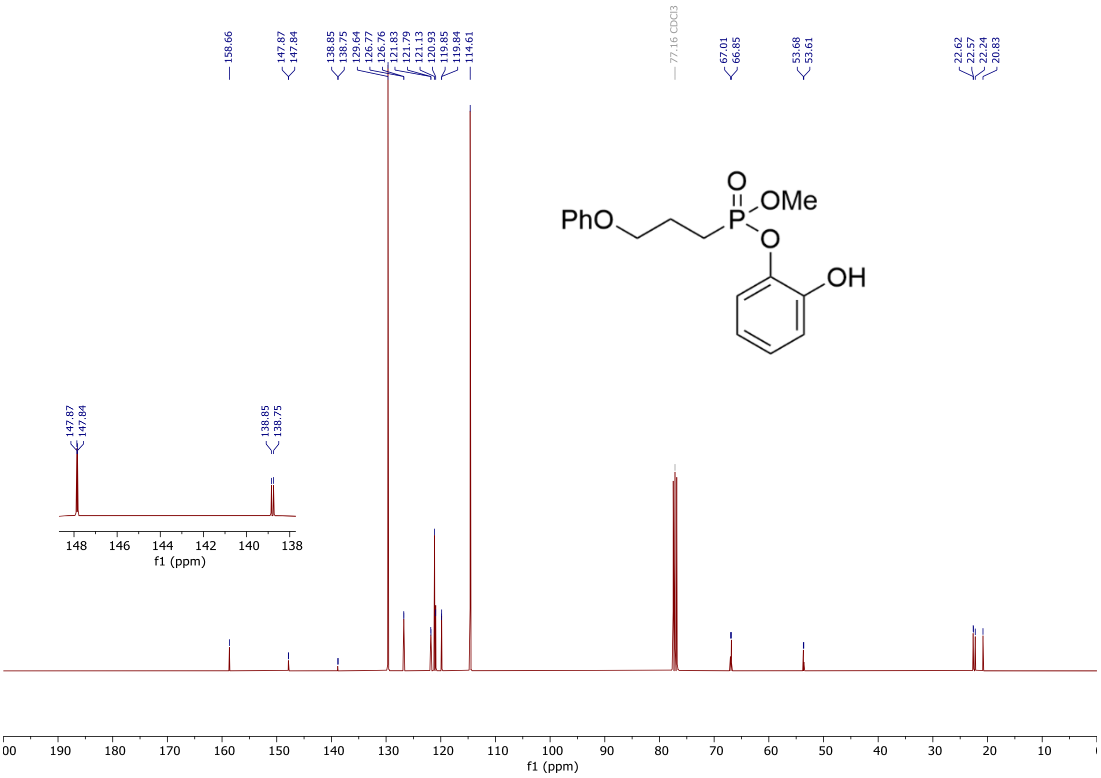

^{31}P NMR (162 MHz, $CDCl_3$): **3h**

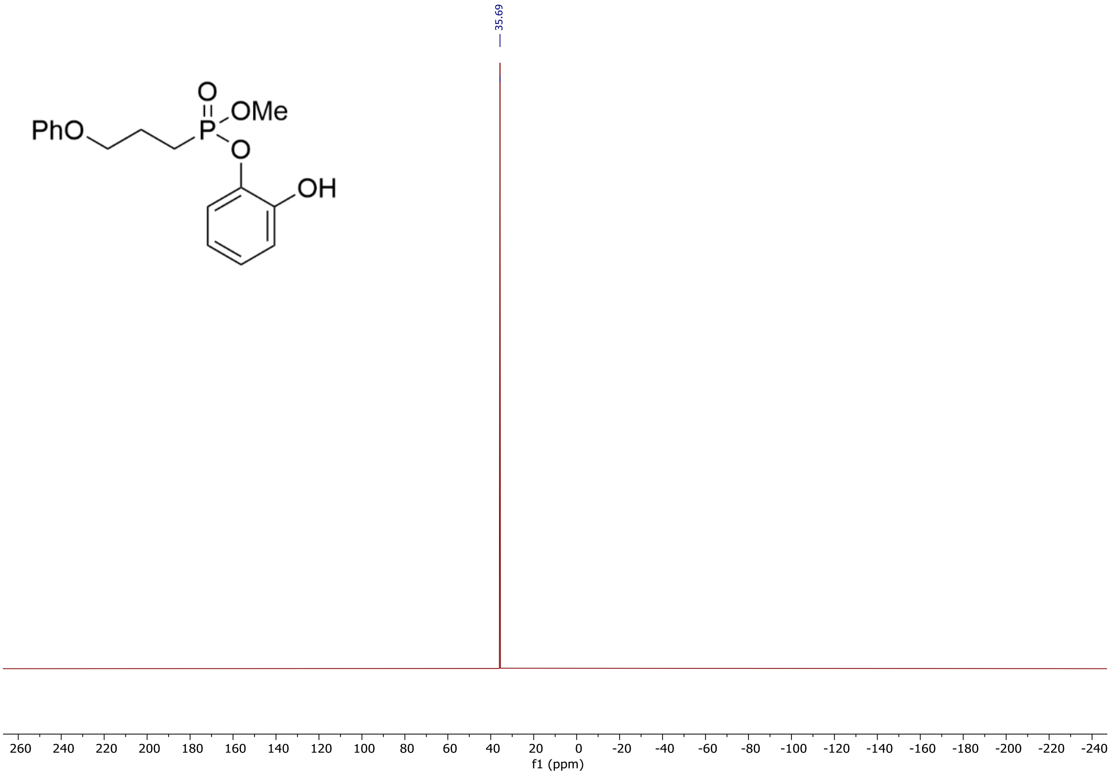

^{1}H NMR (400 MHz, $CDCl_3$): **3i**

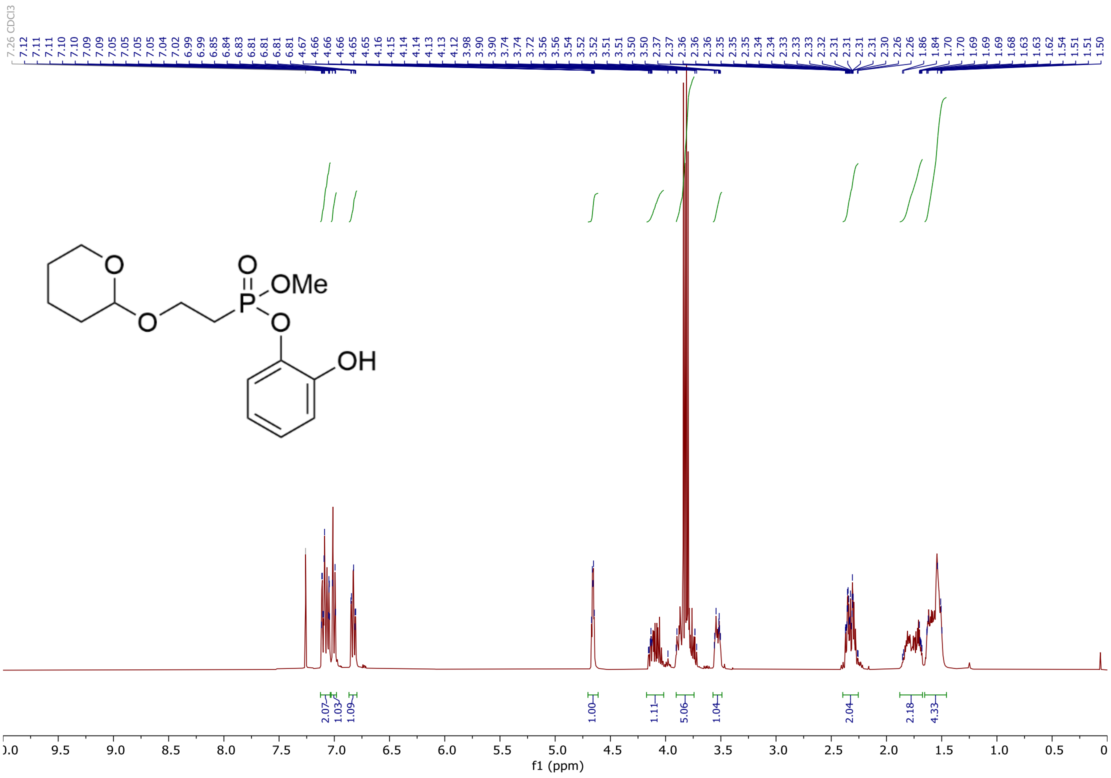

^{13}C NMR (101 MHz, $CDCl_3$): **3i**

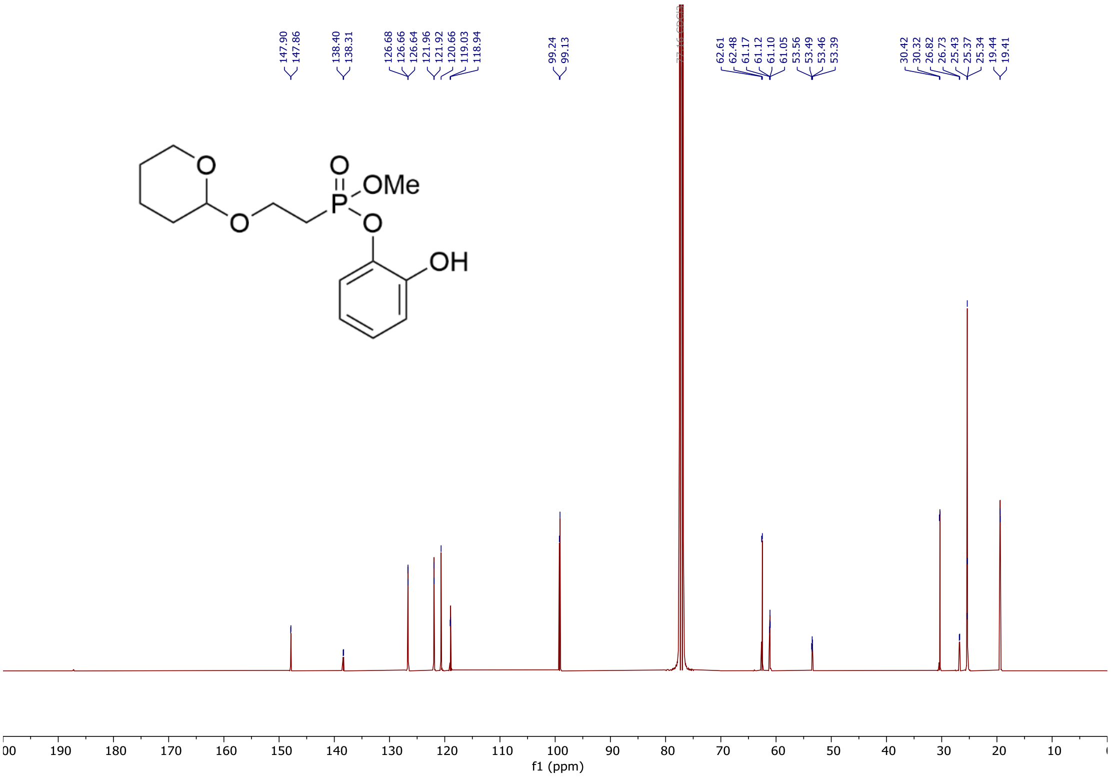

^{31}P NMR (162 MHz, $CDCl_3$): **3i**

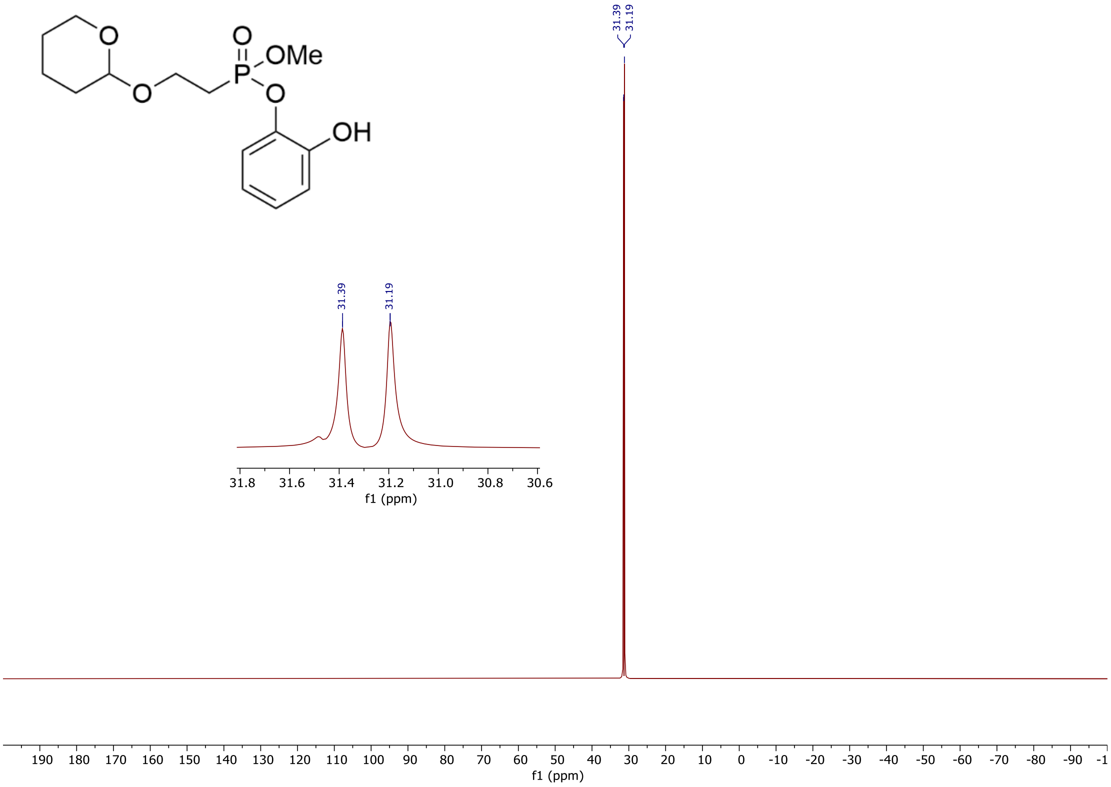

^{1}H NMR (400 MHz, $CDCl_3$): **3j**

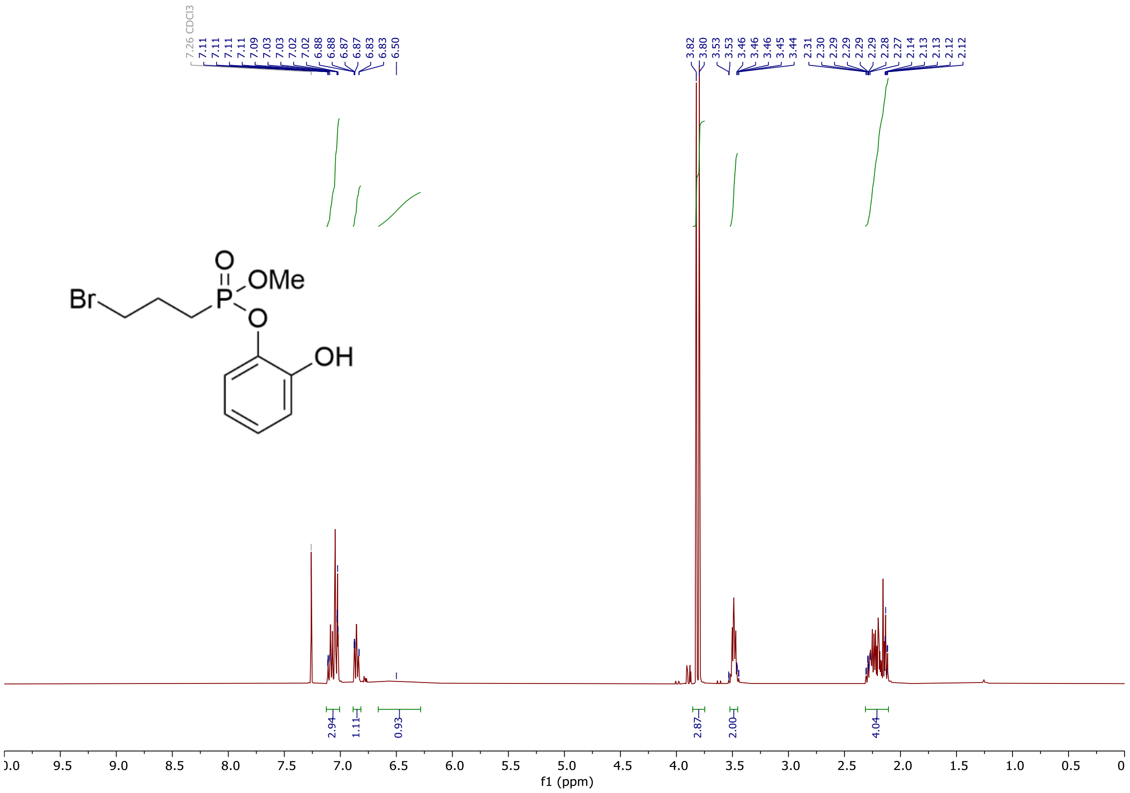

^{13}C NMR (101 MHz, $CDCl_3$): **3j**

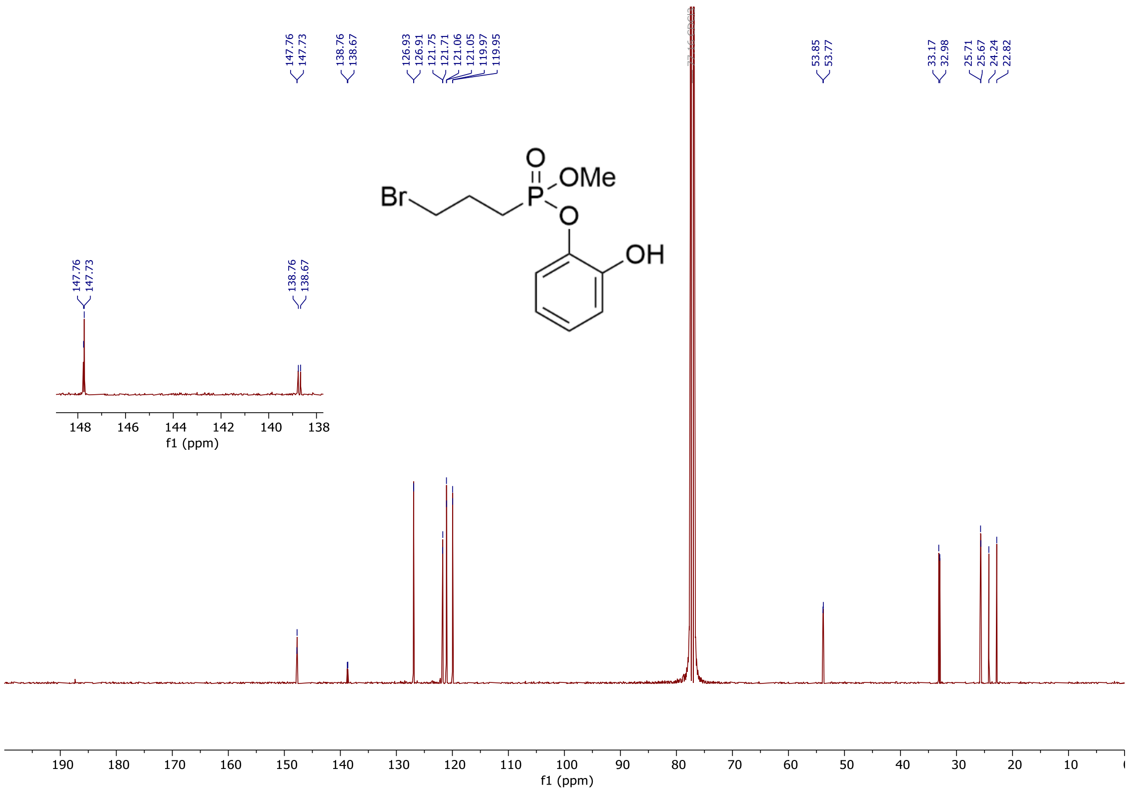

^{31}P NMR (162 MHz, $CDCl_3$): **3j**

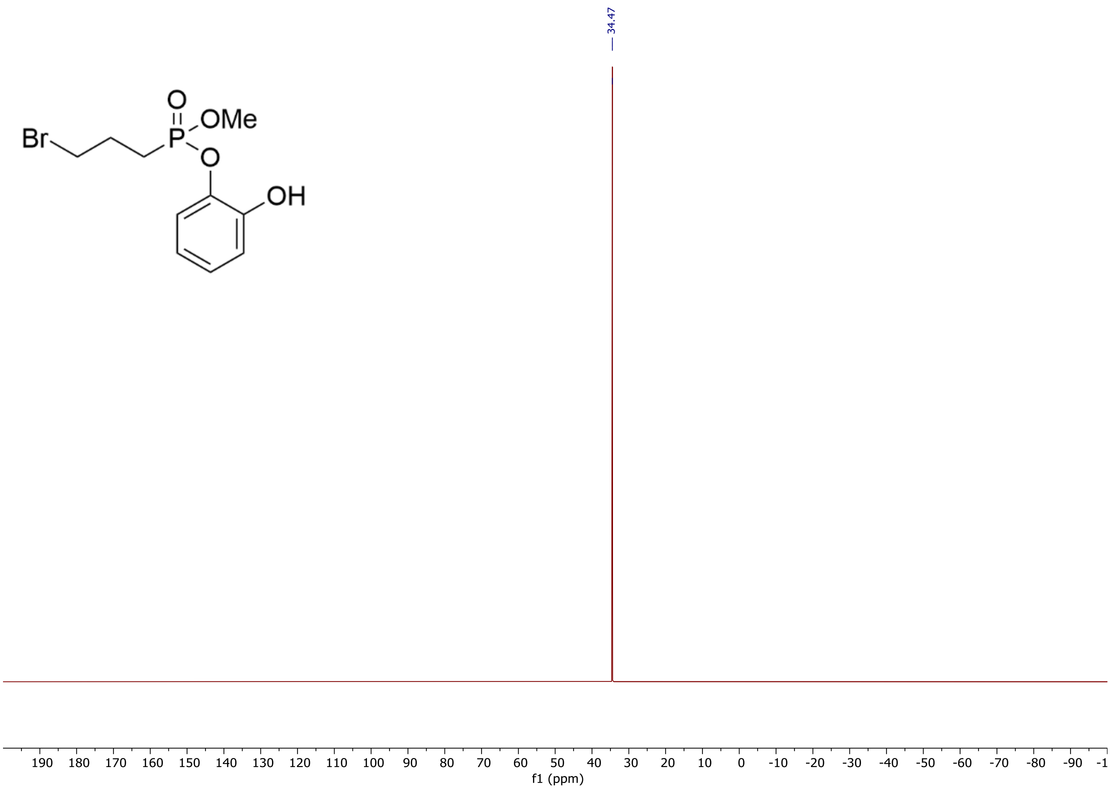

^{1}**H NMR** (400 MHz, $CDCl_3$): **3k**

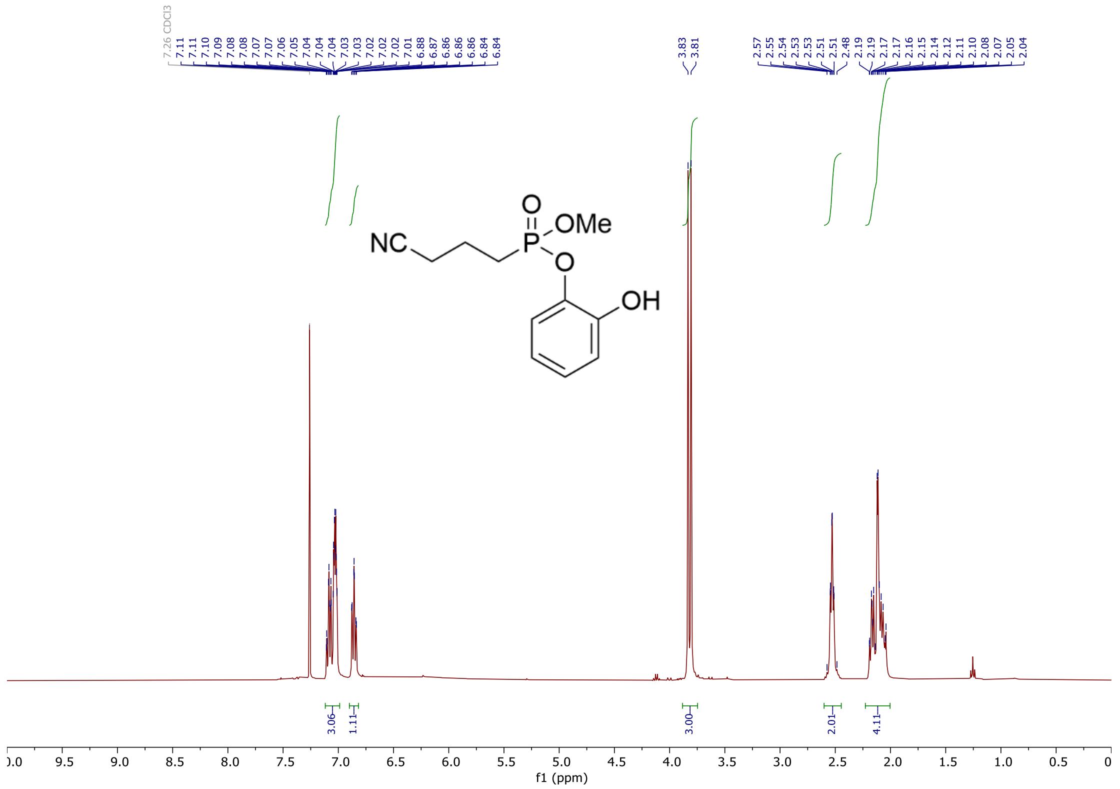

^{13}**C NMR** (101 MHz, $CDCl_3$): **3k**

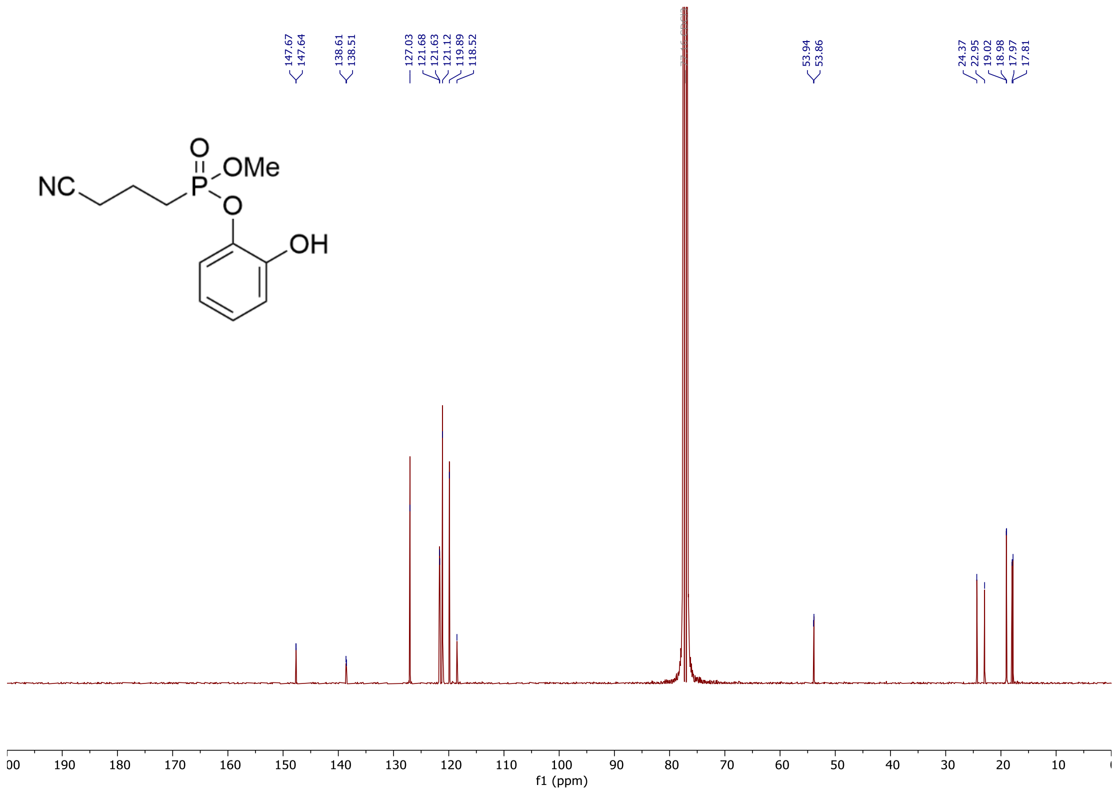

^{31}P NMR (162 MHz, $CDCl_3$): **3k**

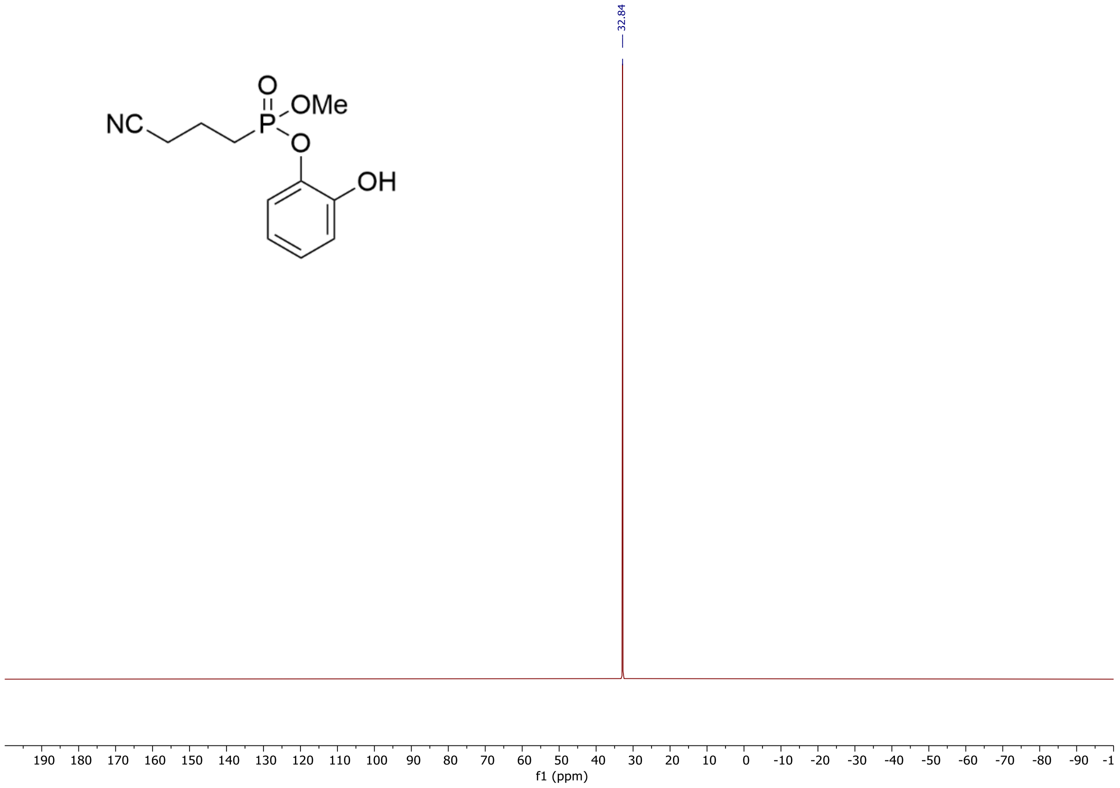

^{1}H NMR (400 MHz, $CDCl_3$): **3l**

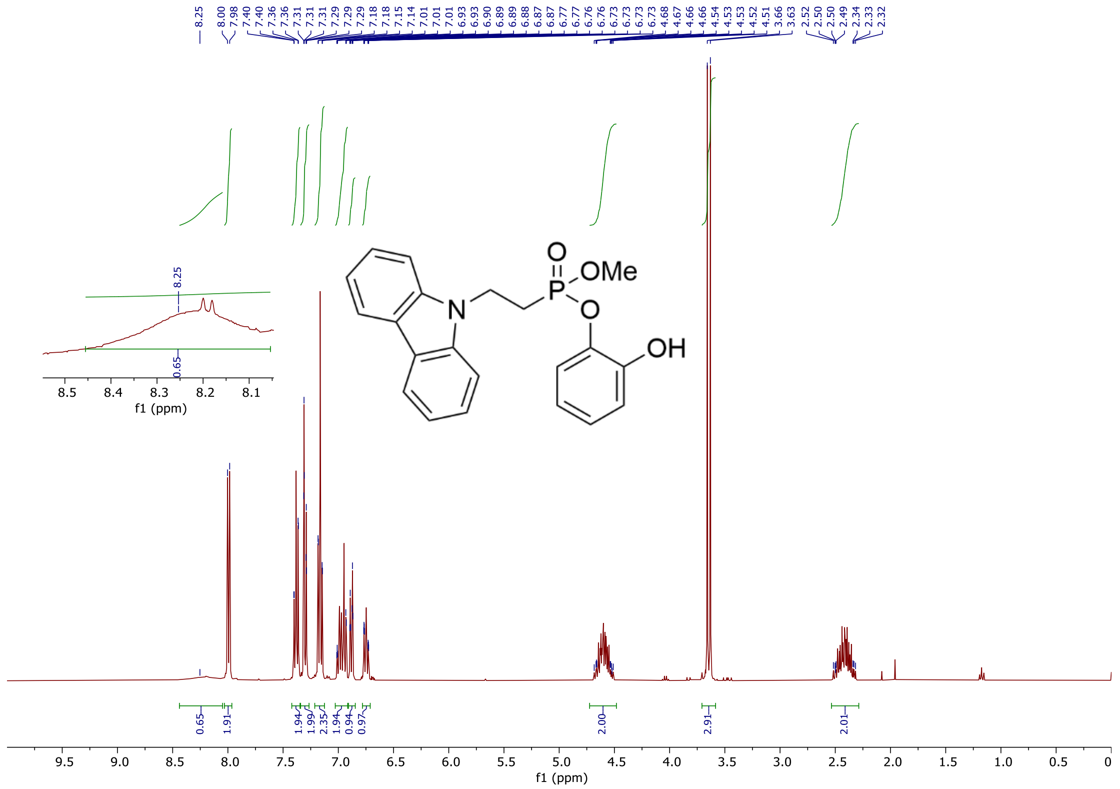

^{13}C NMR (101 MHz, $CDCl_3$): **3l**

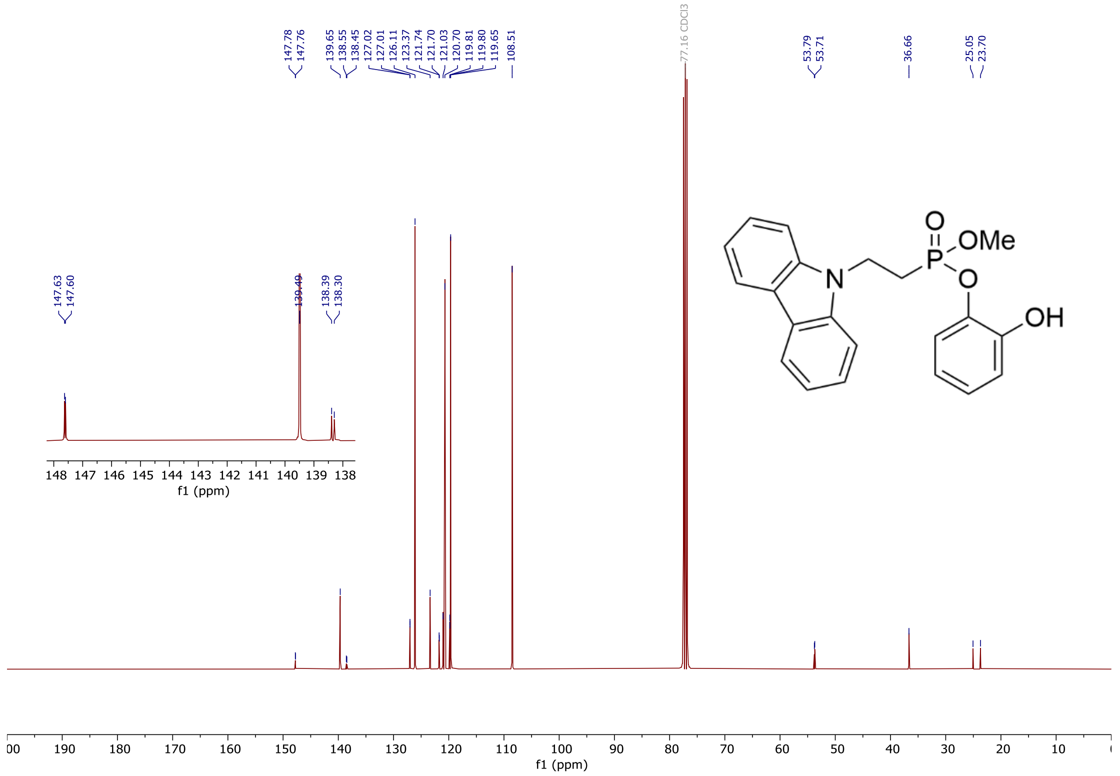

^{31}P NMR (162 MHz, $CDCl_3$): **3l**

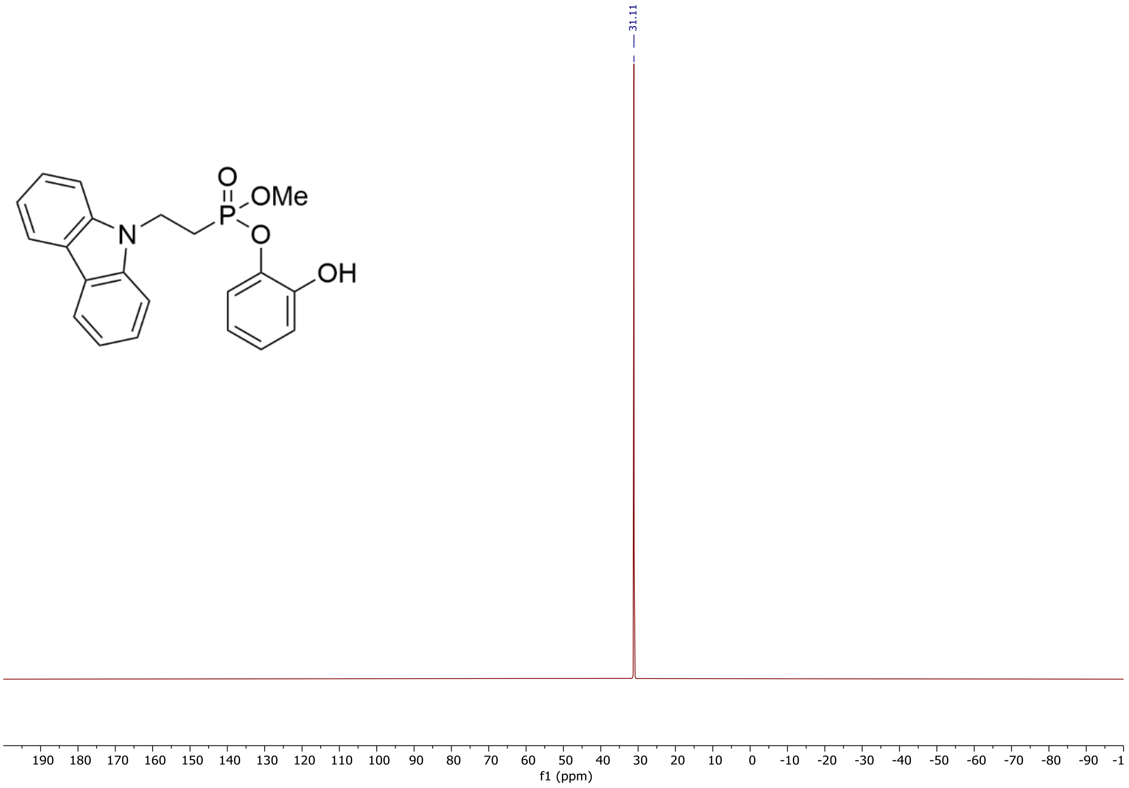

^{1}H NMR (400 MHz, $CDCl_3$): **3m**

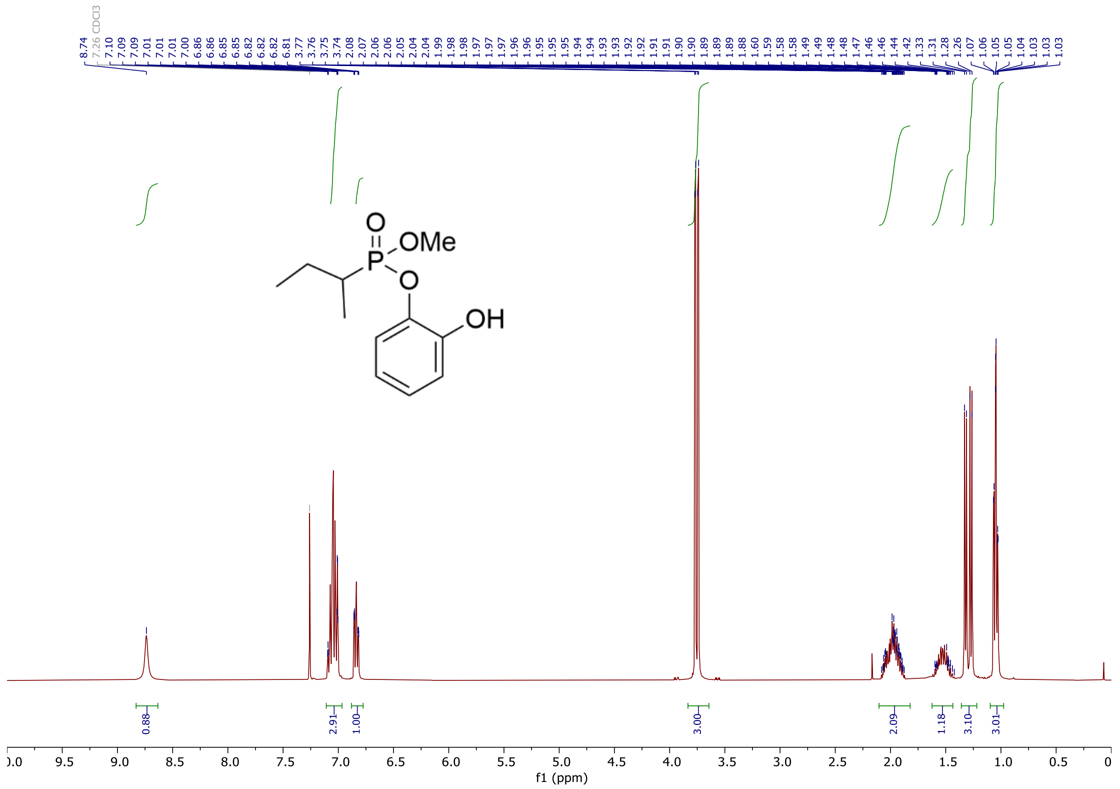

^{13}C NMR (101 MHz, $CDCl_3$): **3m**

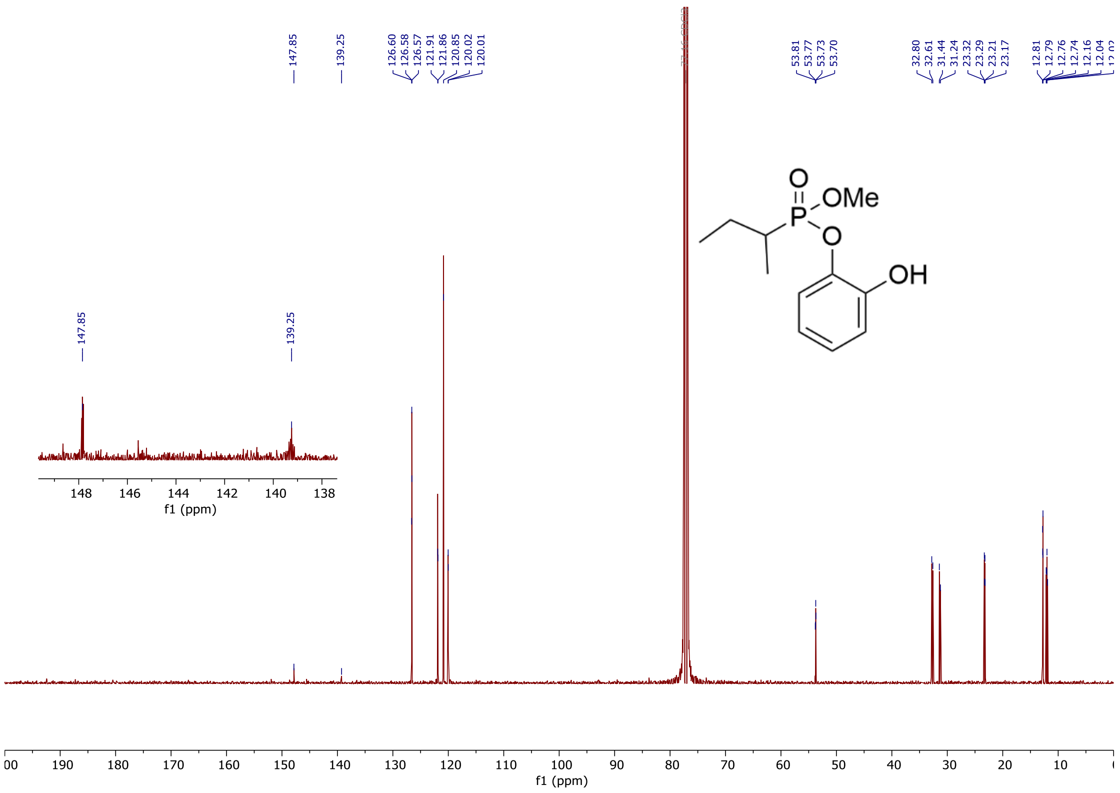

^{31}**P NMR** (162 MHz, $CDCl_3$): **3m**

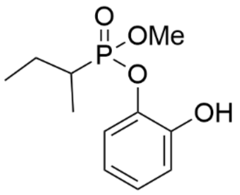

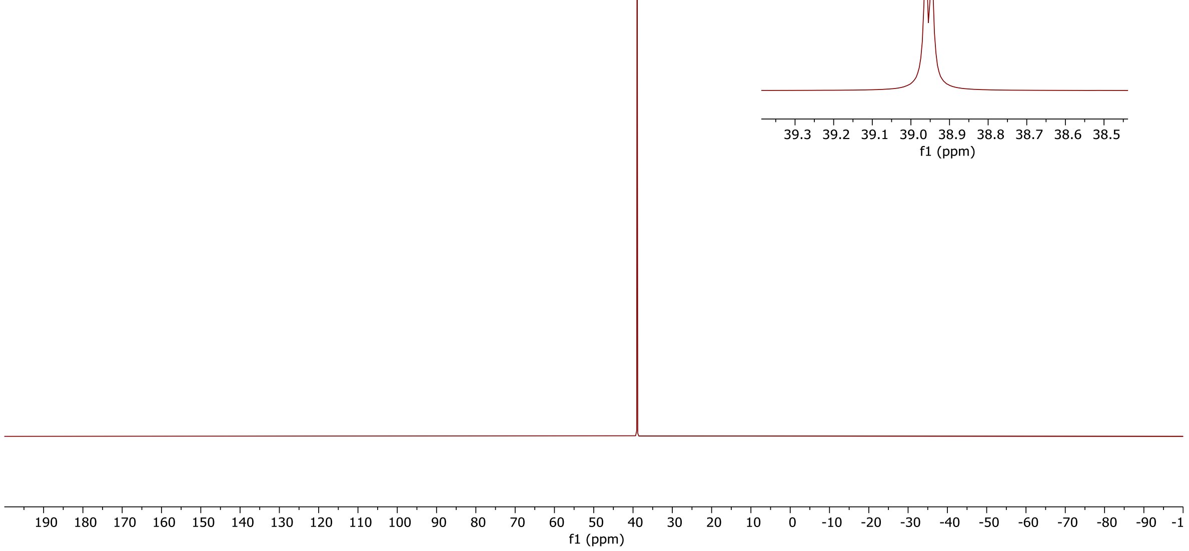

¹H NMR (400 MHz, CDCl3): **3n**

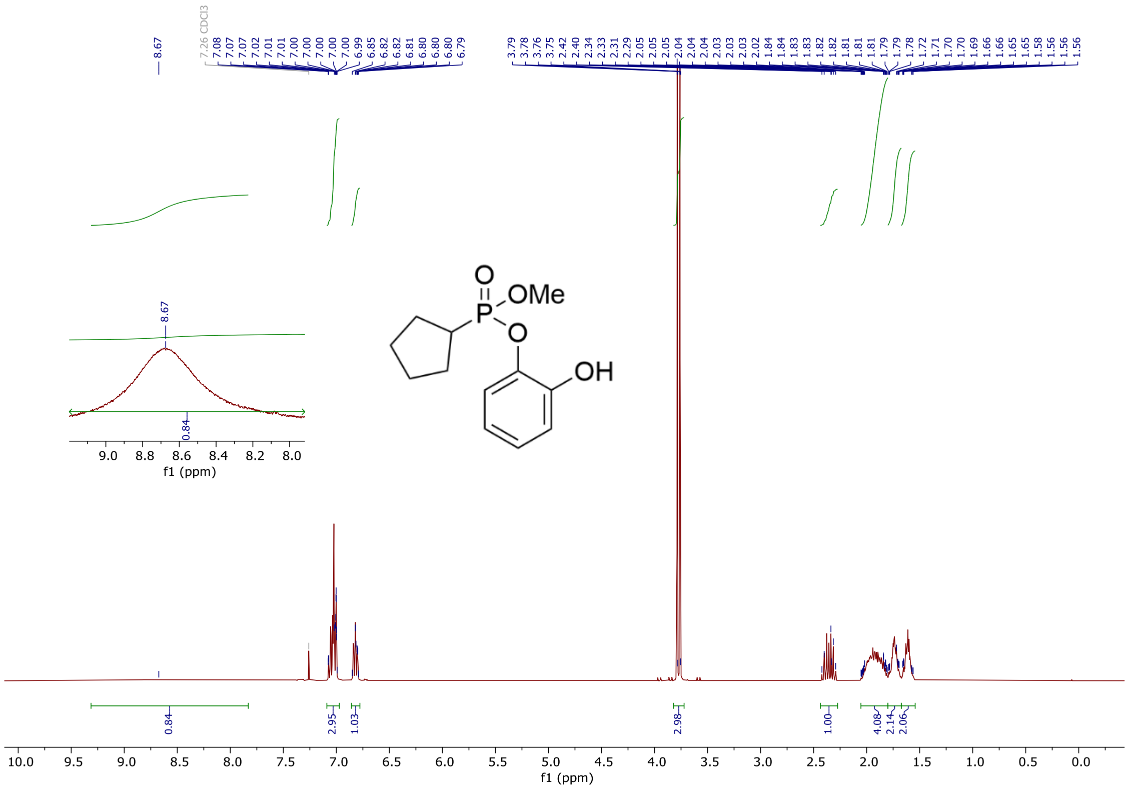

¹³C NMR (101 MHz, CDCl3): **3n**

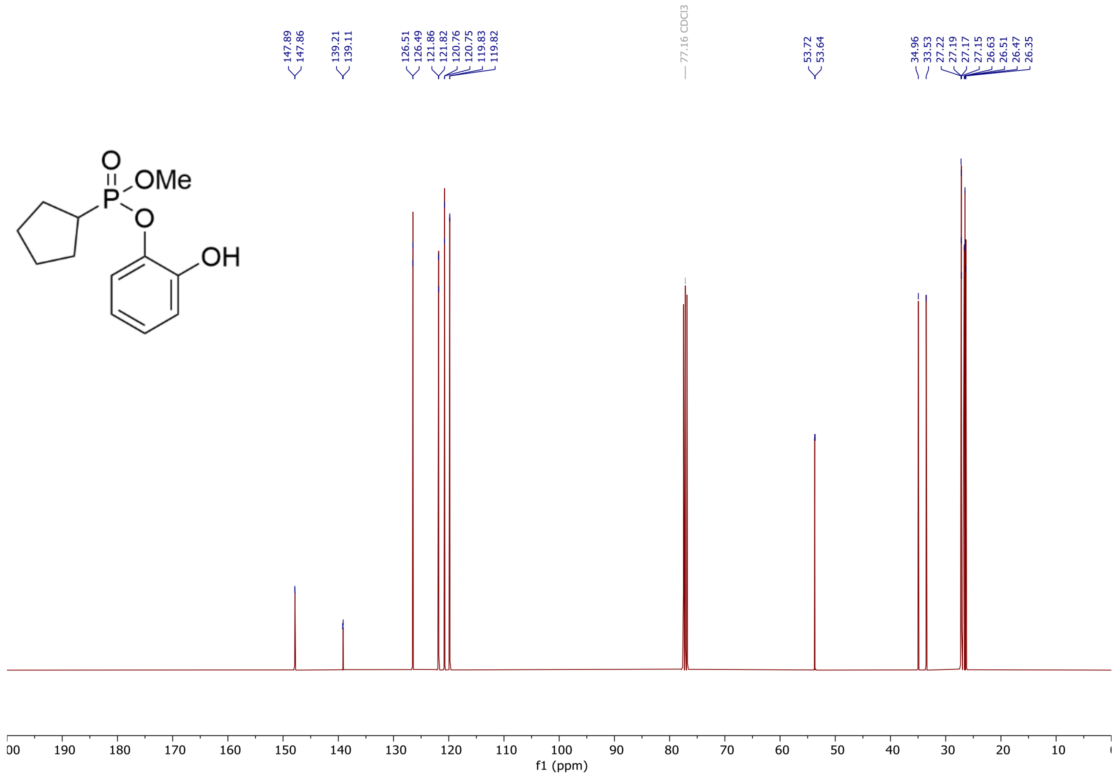

^{31}P NMR (162 MHz, $CDCl_3$): **3n**

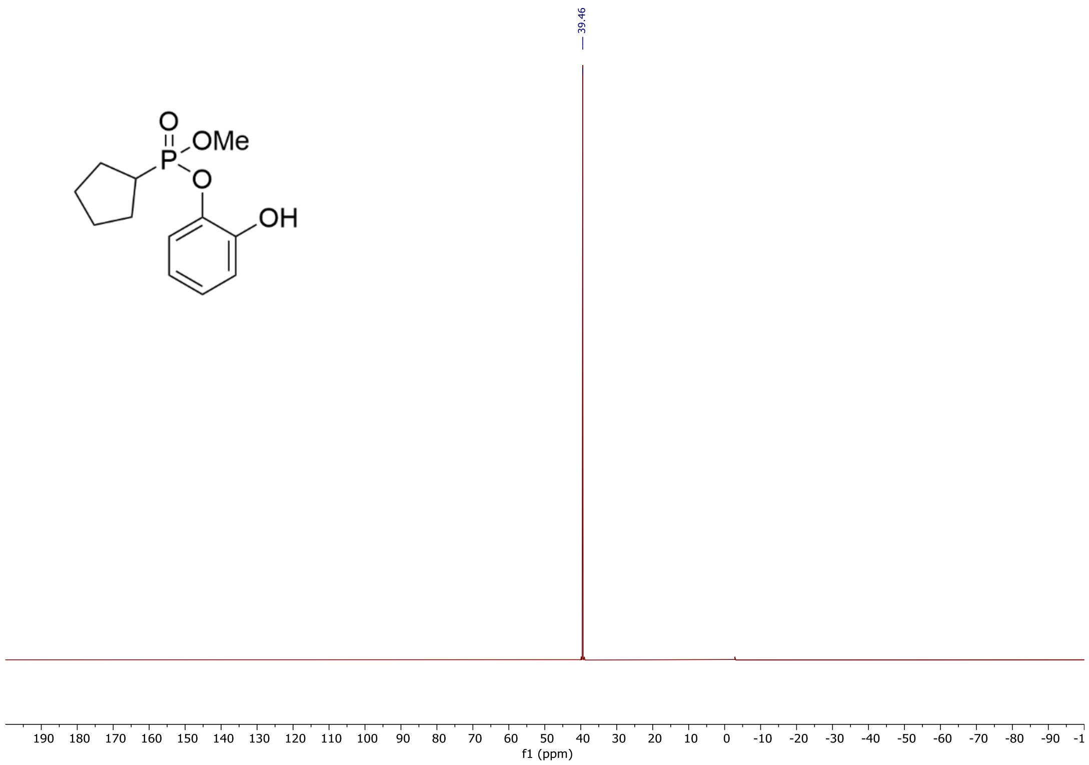

^{1}H NMR (400 MHz, $CDCl_3$): **3o**

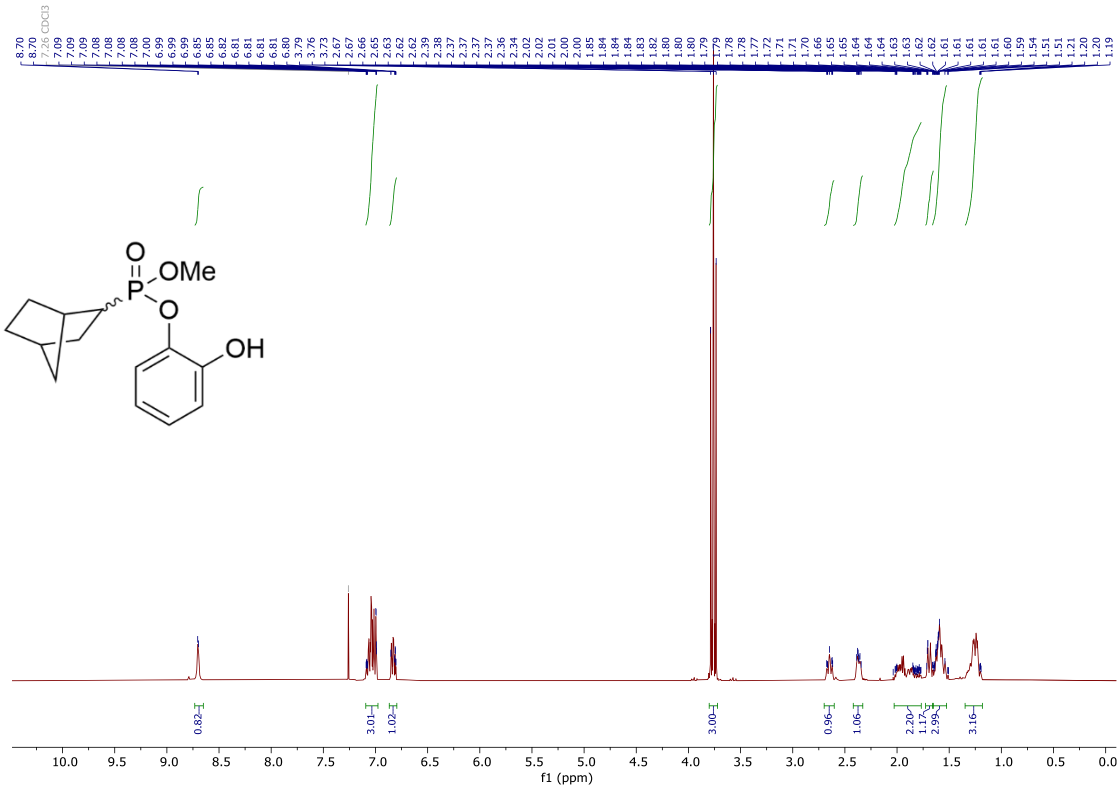

^{13}C NMR (101 MHz, $CDCl_3$): **3o**

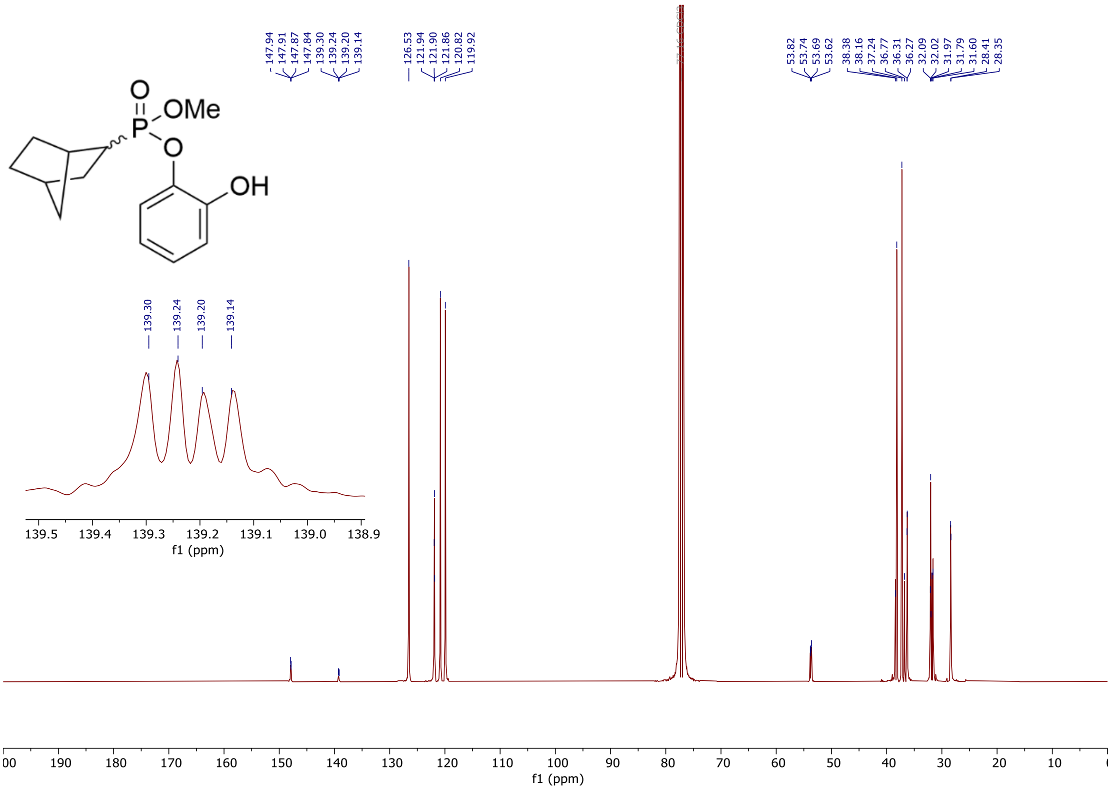

^{31}P NMR (162 MHz, $CDCl_3$): **3o**

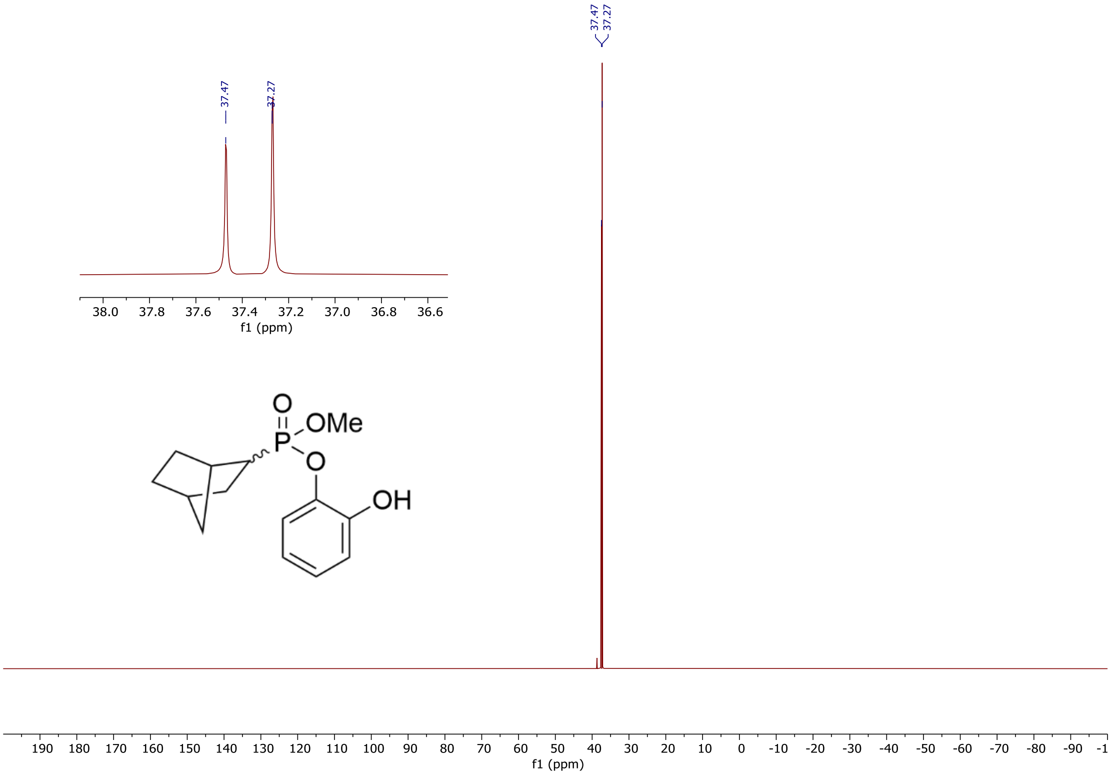

^{1}**H NMR** (400 MHz, $CDCl_3$): **3p**

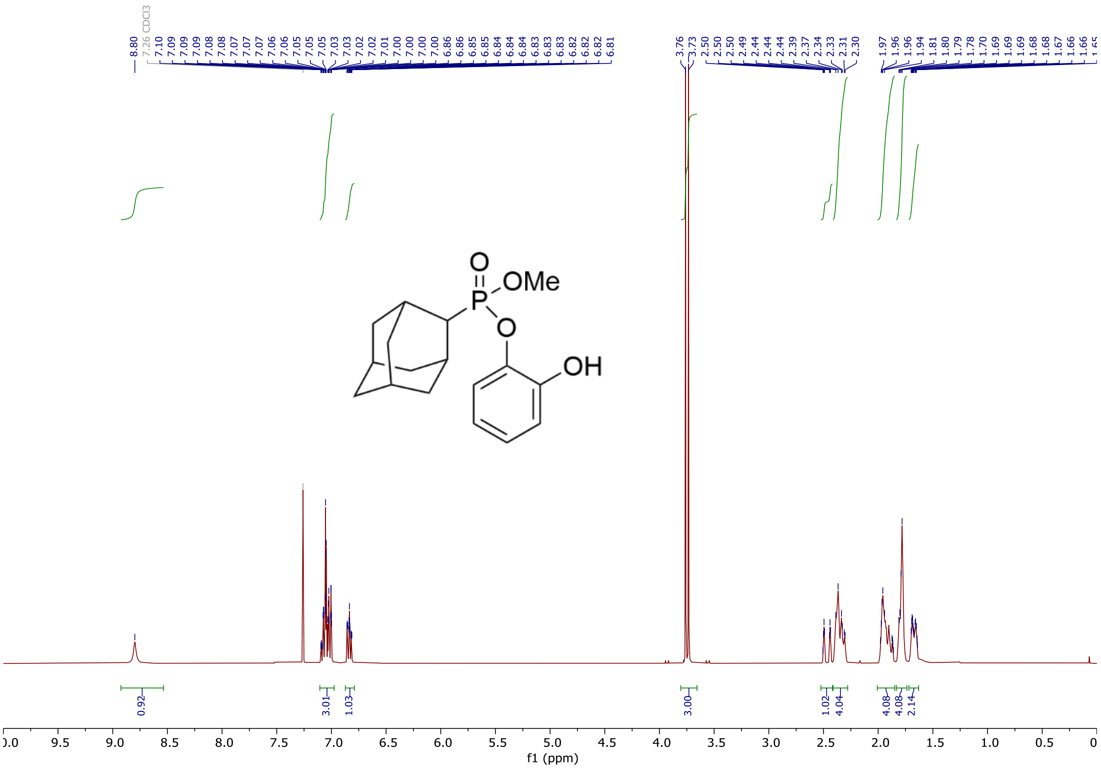

^{13}**C NMR** (101 MHz, $CDCl_3$): **3p**

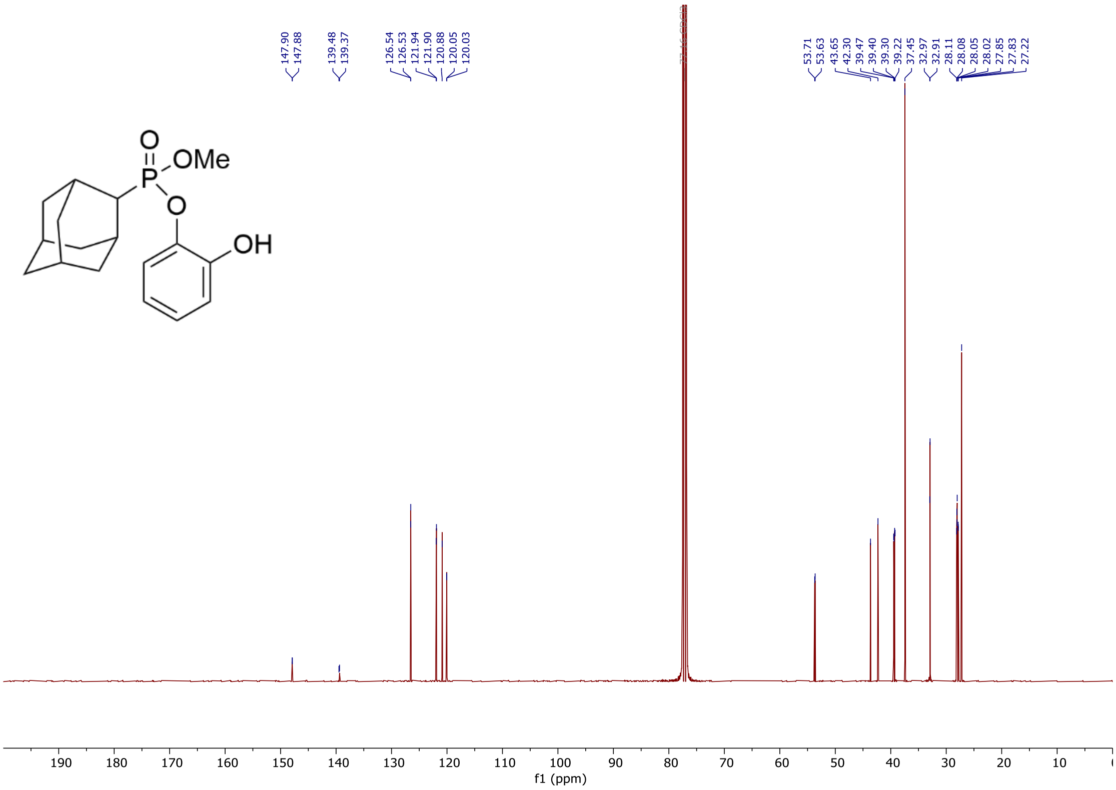

^{31}P NMR (162 MHz, $CDCl_3$): **3p**

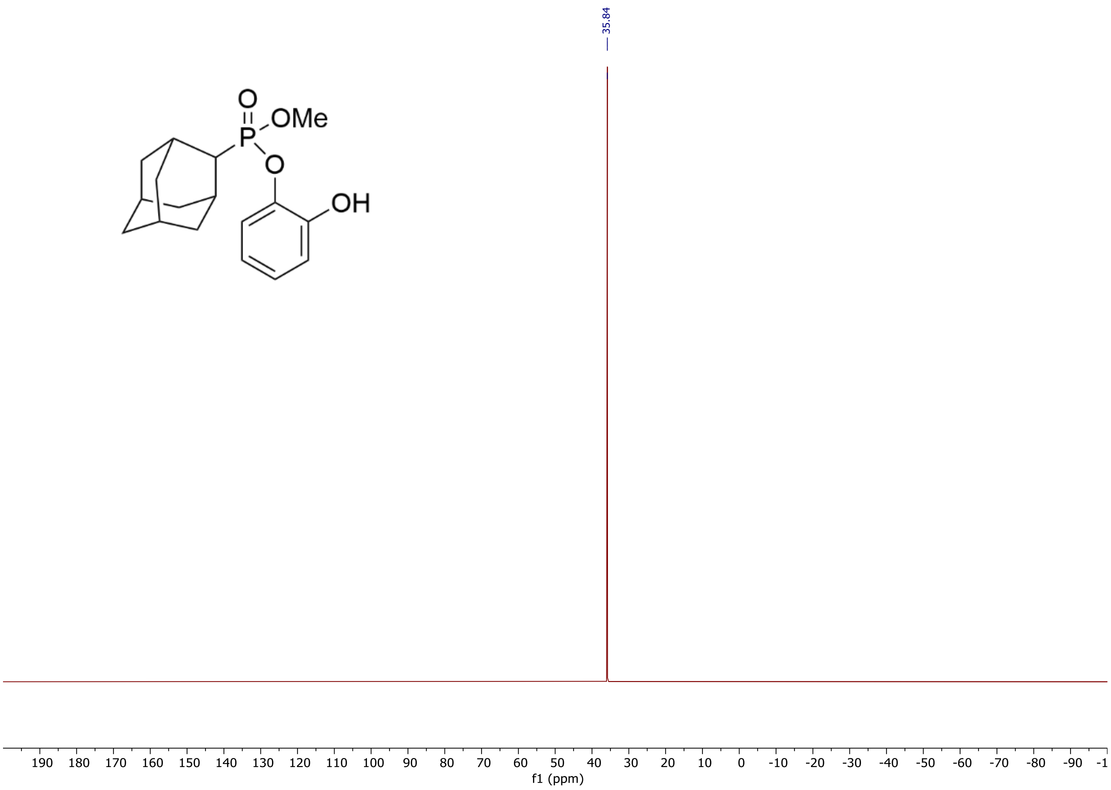

^{1}H NMR (400 MHz, CDCl$_3$): **3r**

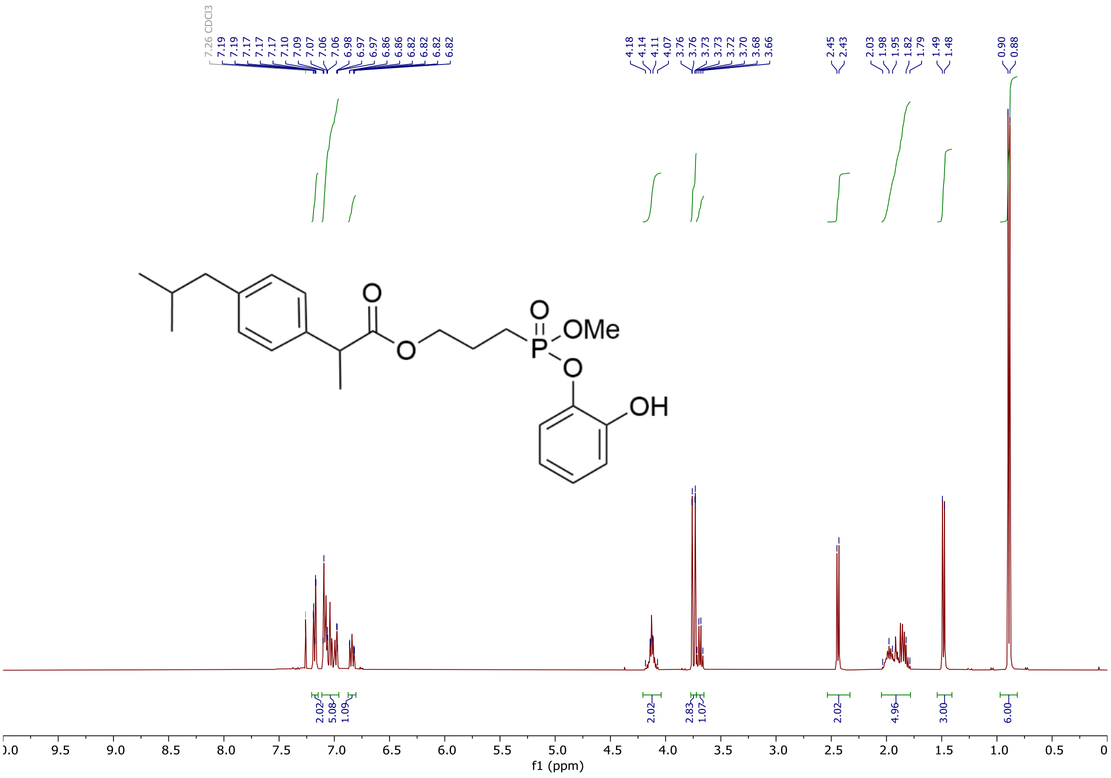

^{13}C NMR (101 MHz, CDCl$_3$): **3r**

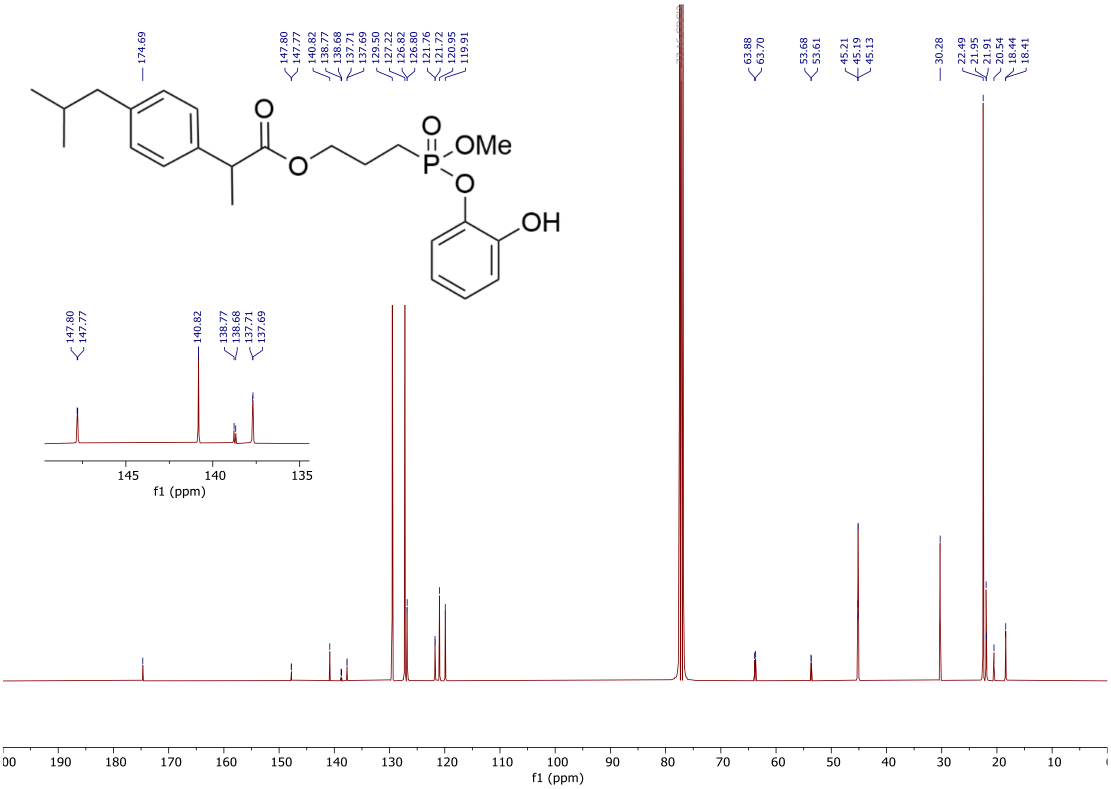

^{31}**P NMR** (162 MHz, $CDCl_3$): **3r**

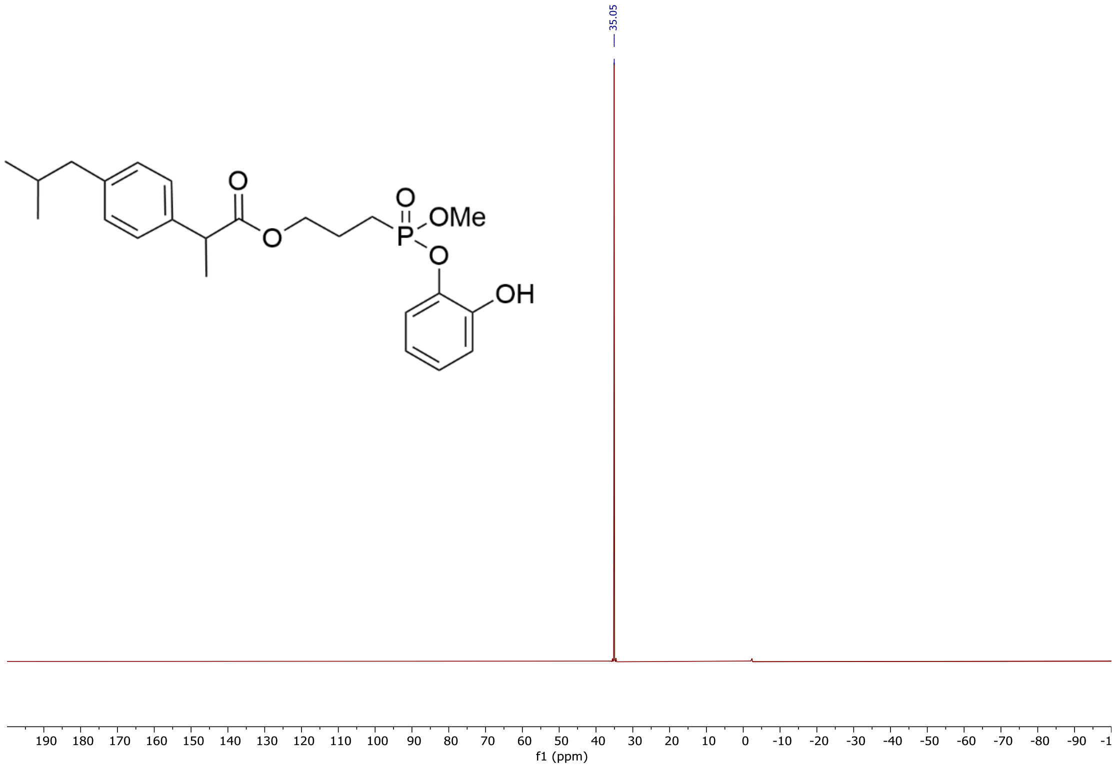

^{1}H NMR (400 MHz, $CDCl_3$): **3s**

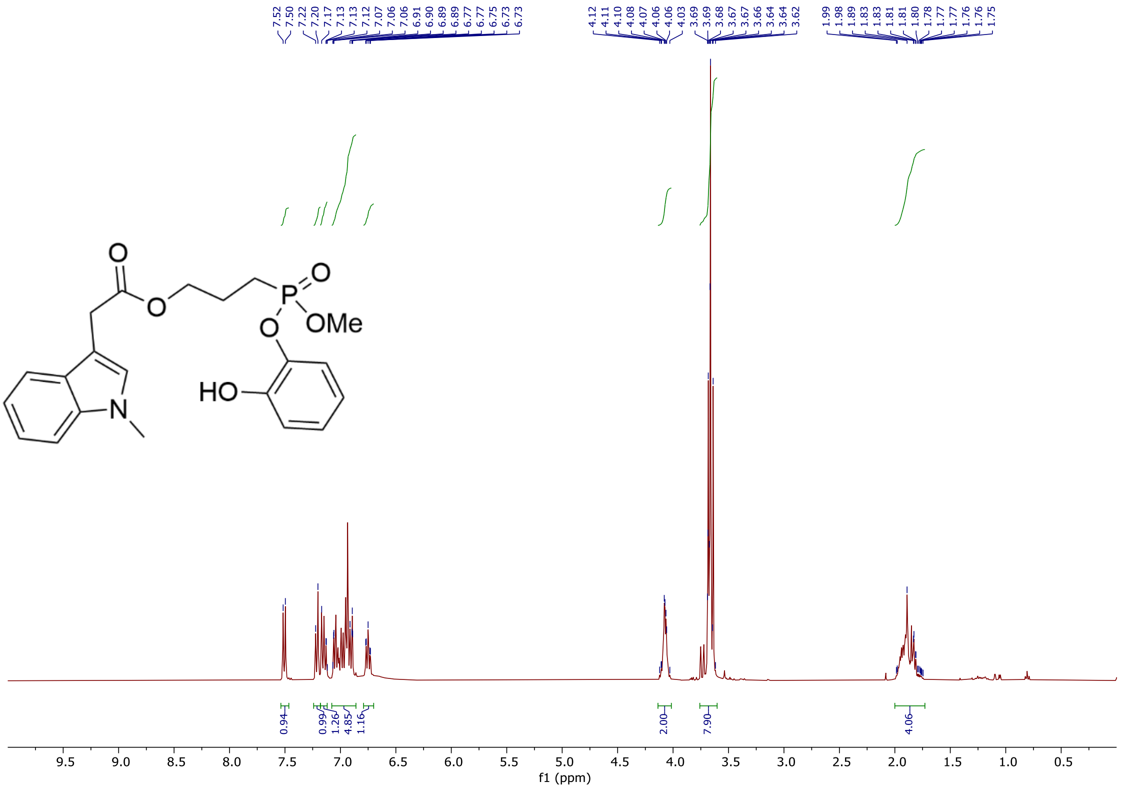

^{13}C NMR (101 MHz, $CDCl_3$): **3s**

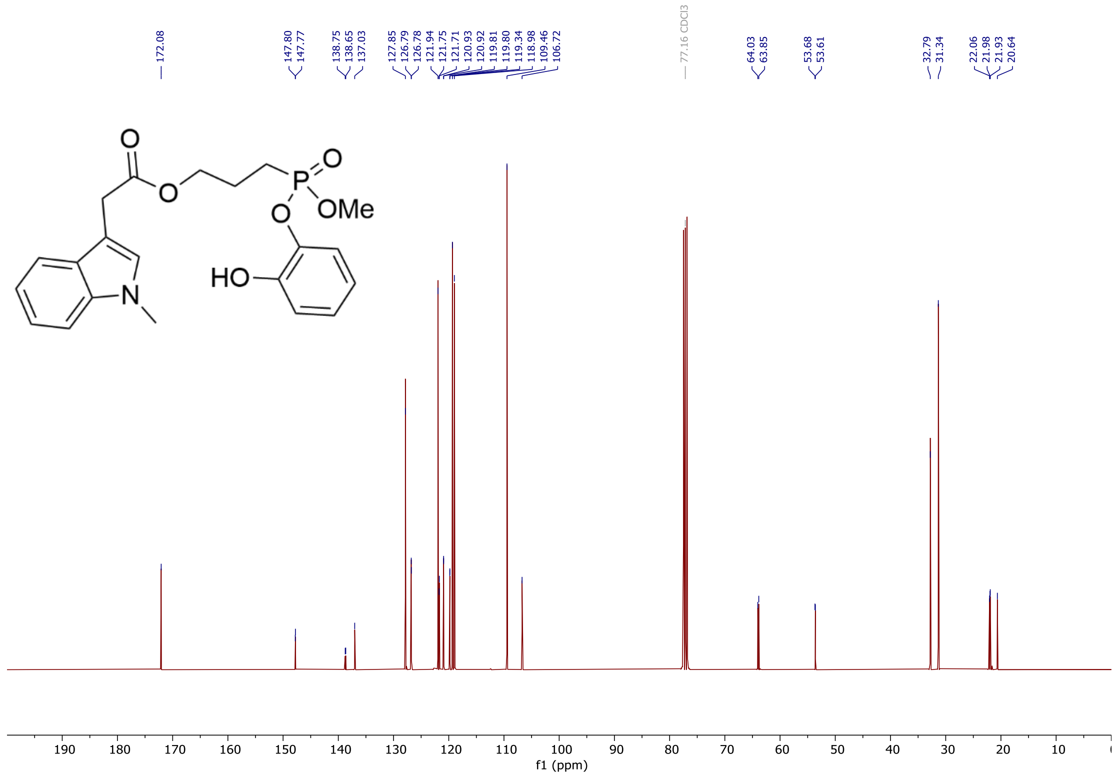

^{31}P NMR (162 MHz, $CDCl_3$): **3s**

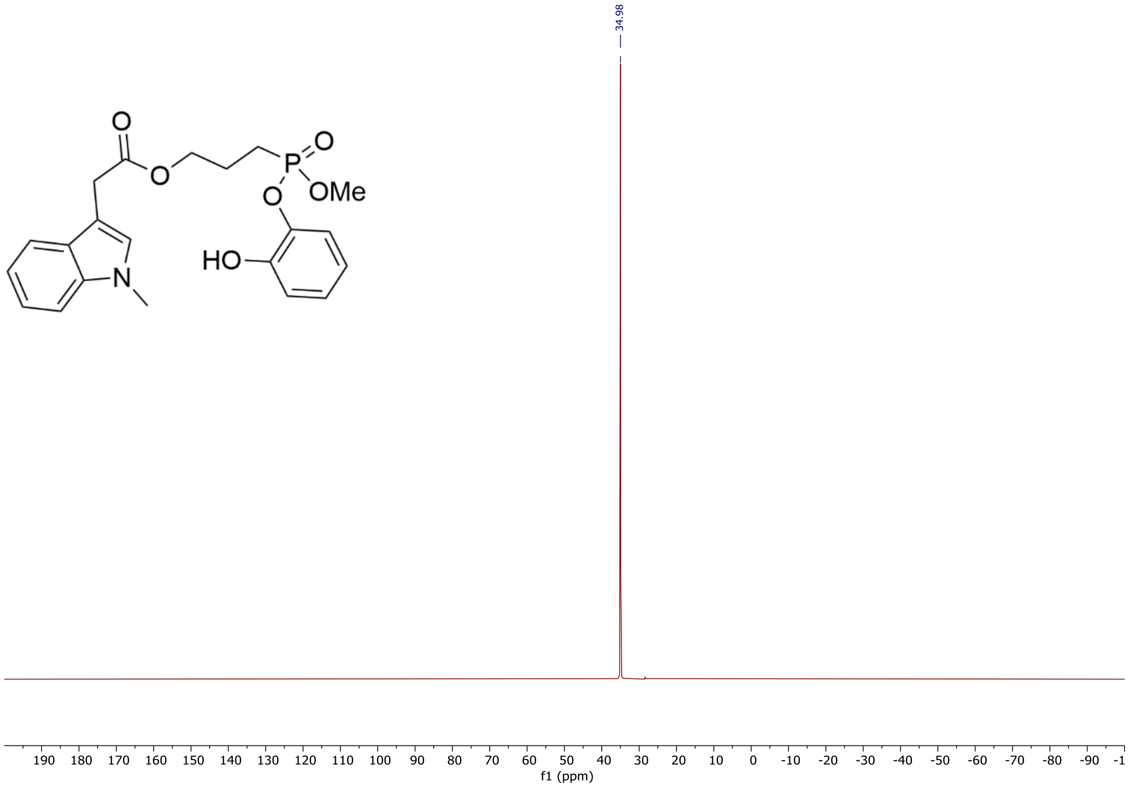

^{1}H NMR (400 MHz, $CDCl_3$): **3t**

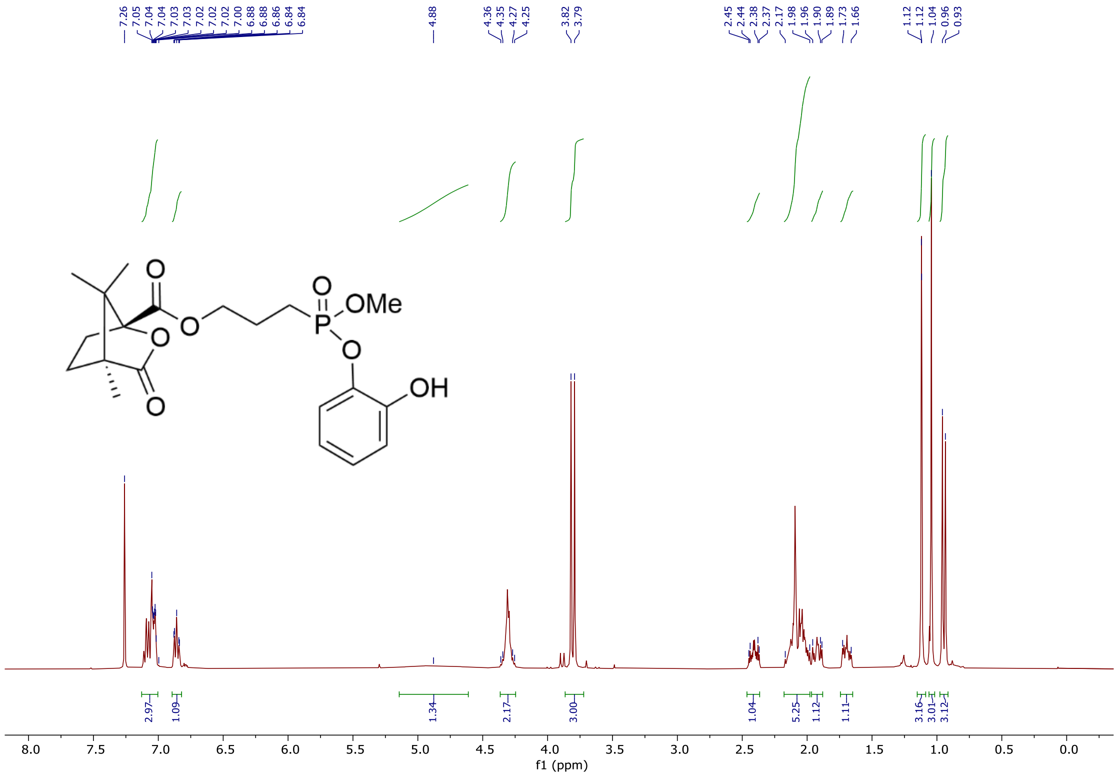

^{13}C NMR (101 MHz, $CDCl_3$): **3t**

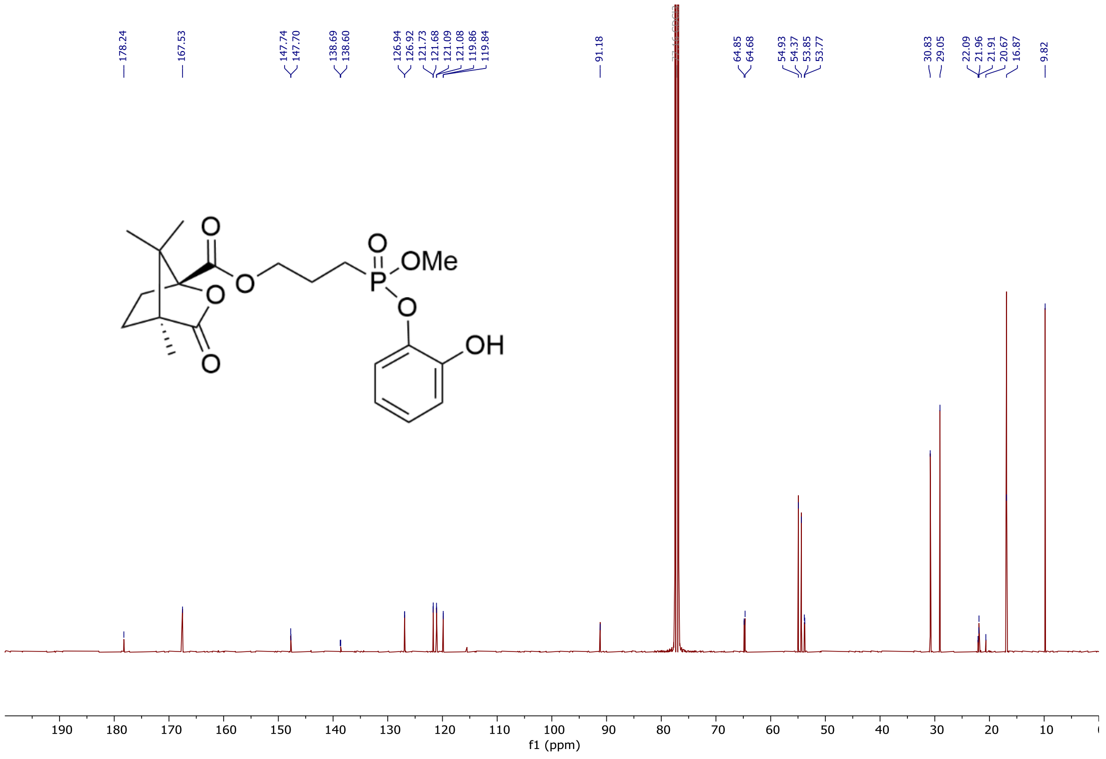

^{31}P NMR (162 MHz, $CDCl_3$): **3t**

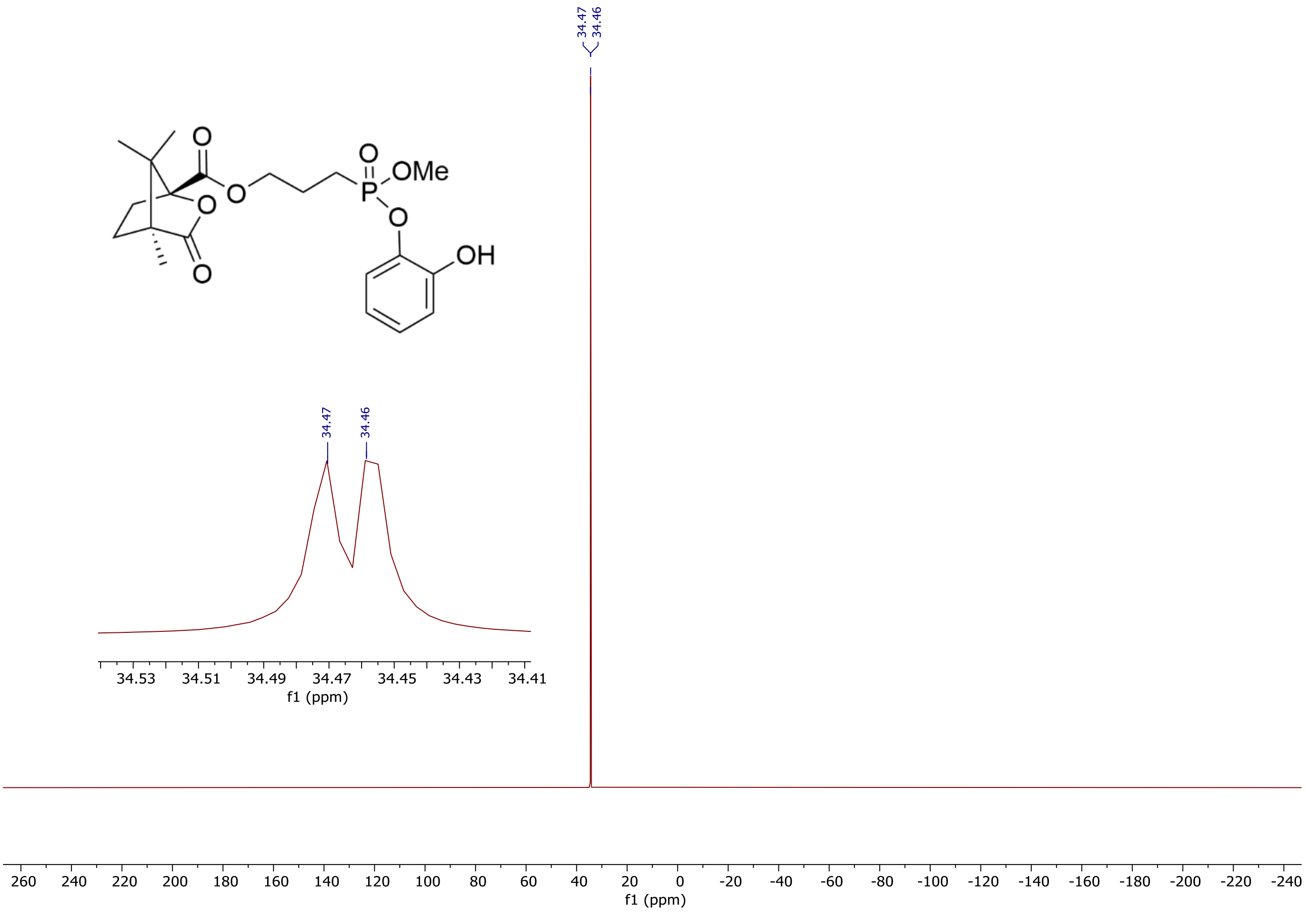

^{1}H NMR (400 MHz, CDCl3): **3u**

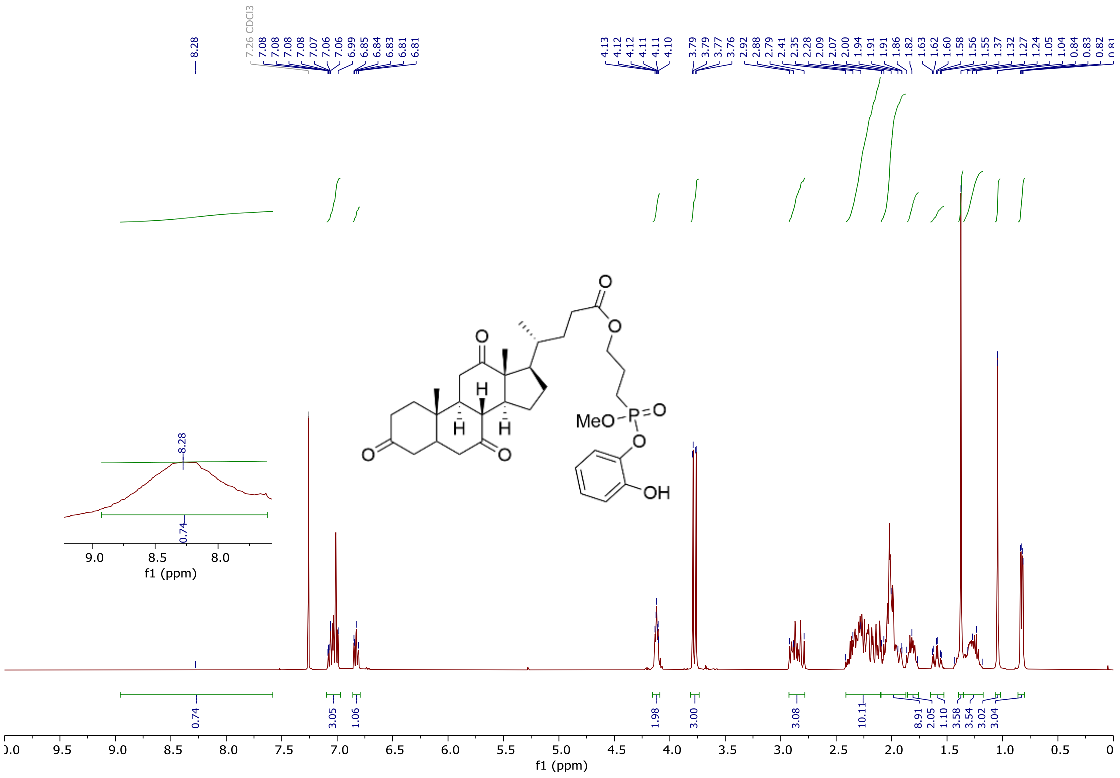

^{13}C NMR (101 MHz, CDCl3): **3u**

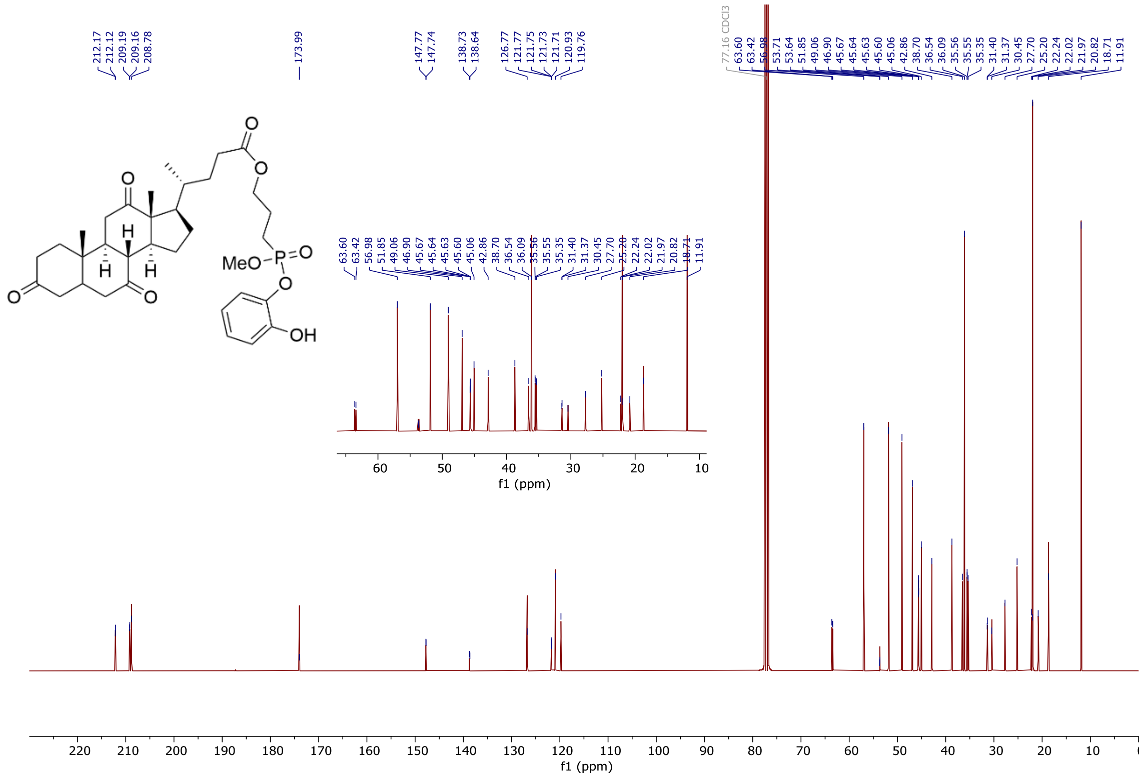

^{31}**P NMR** (162 MHz, $CDCl_3$): **3u**

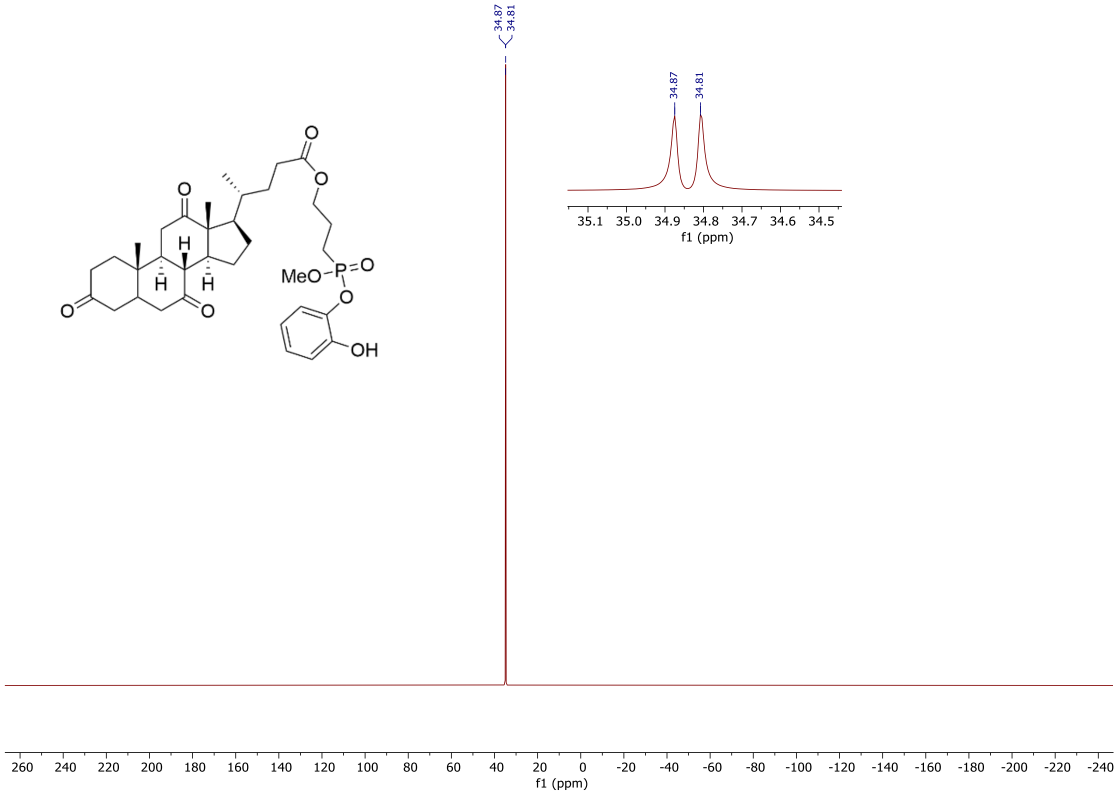

^{1}H NMR (400 MHz, $CDCl_3$): **3v**

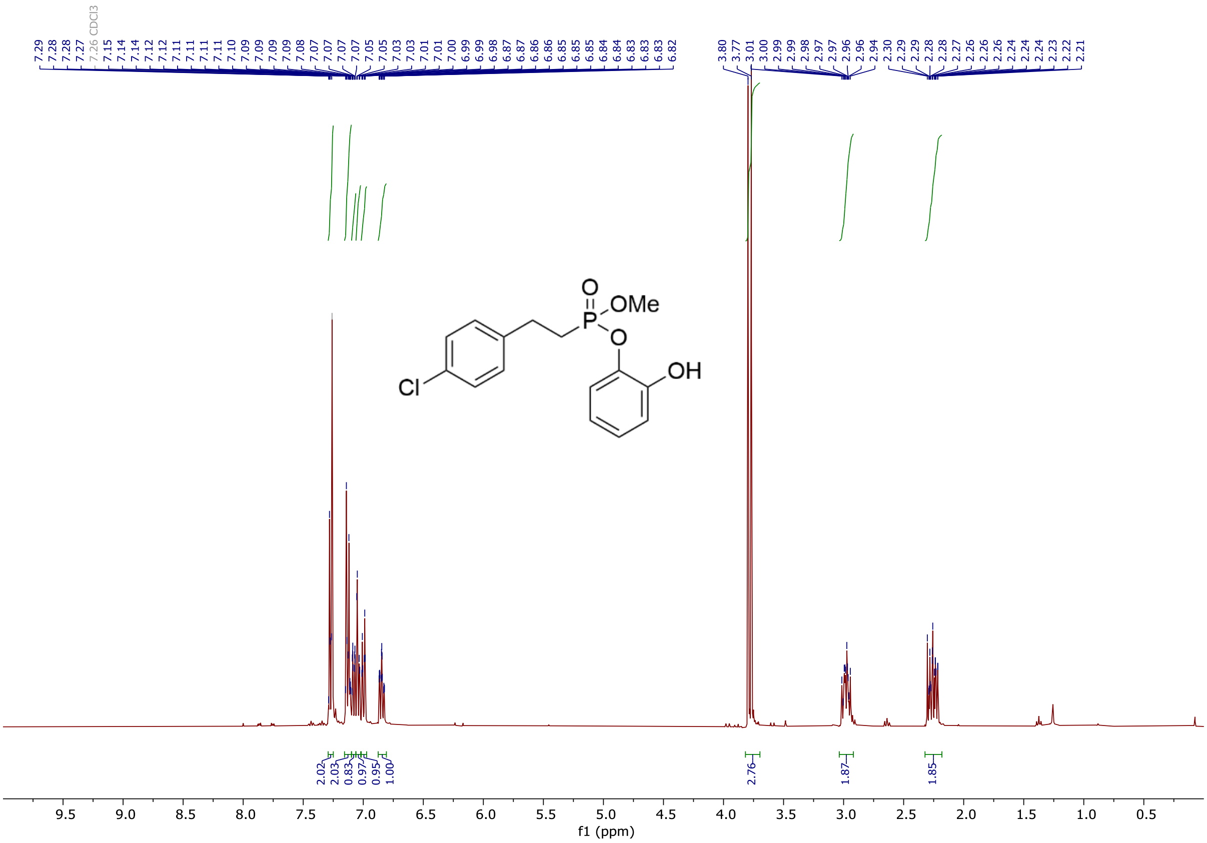

^{13}C NMR (101 MHz, $CDCl_3$): **3v**

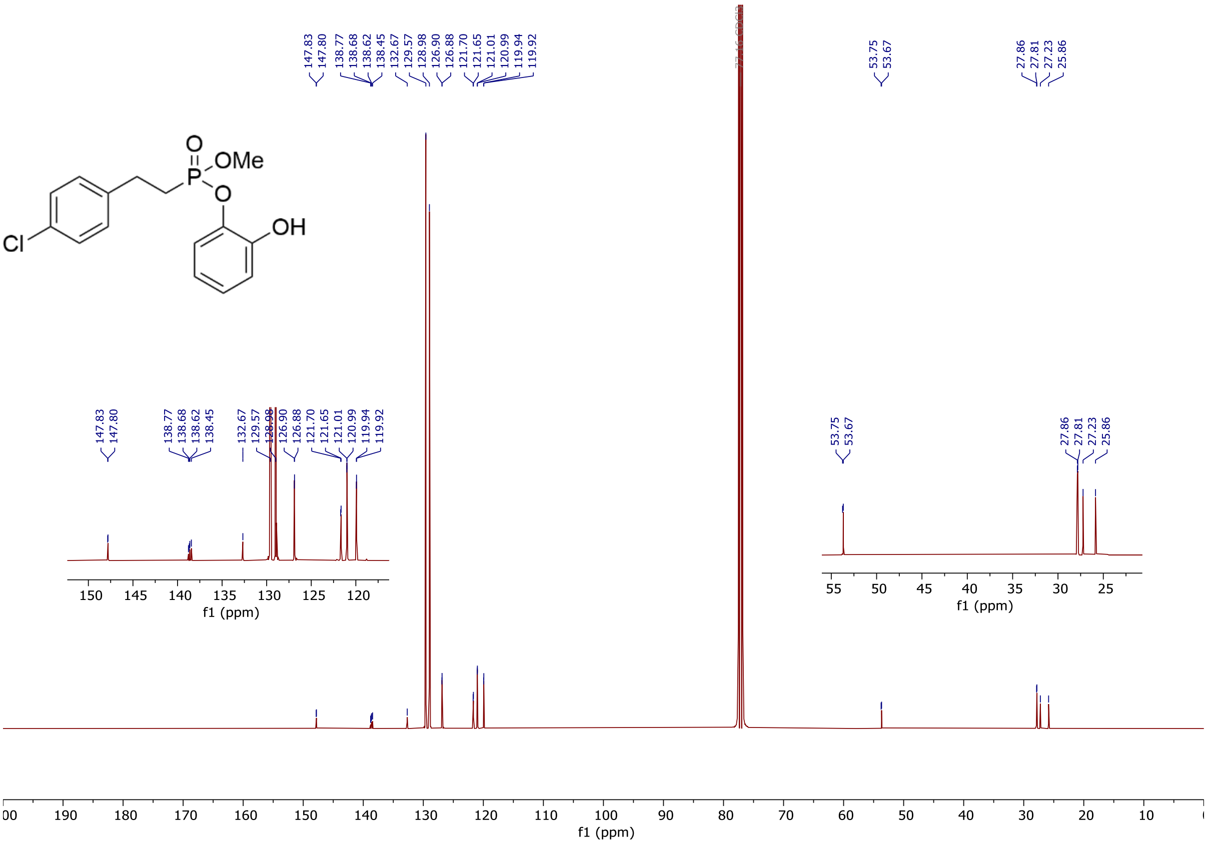

^{31}P NMR (162 MHz, $CDCl_3$): **3v**

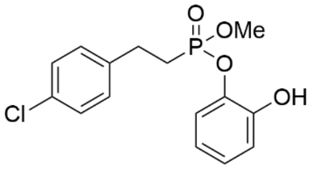

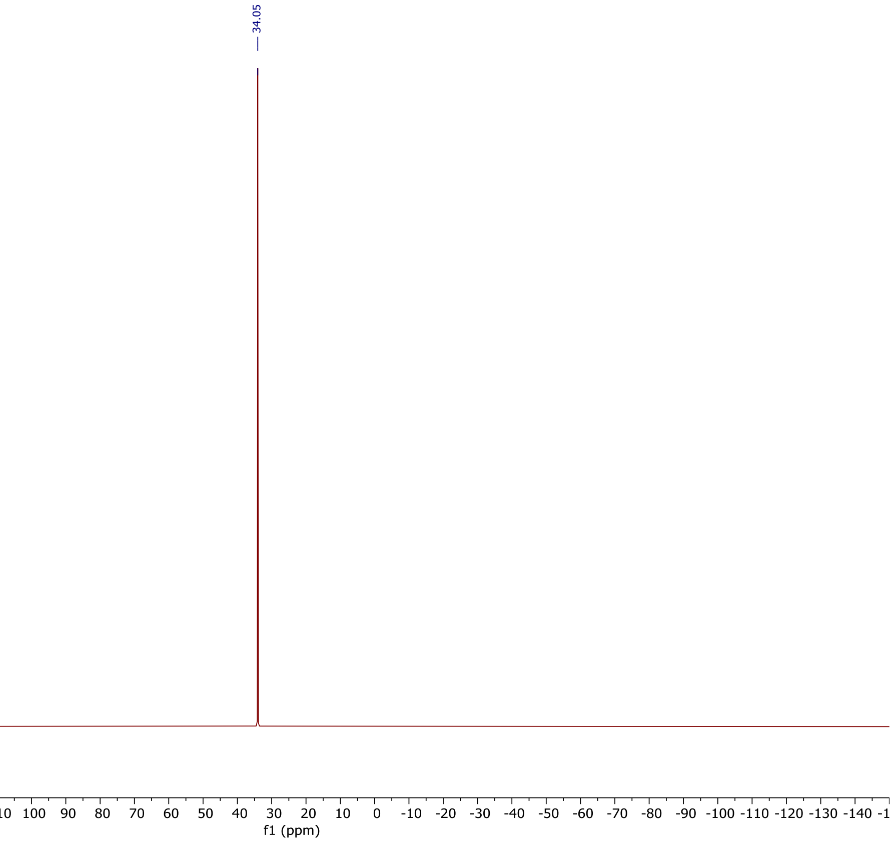

^{1}H NMR (400 MHz, $CDCl_3$): **3w**

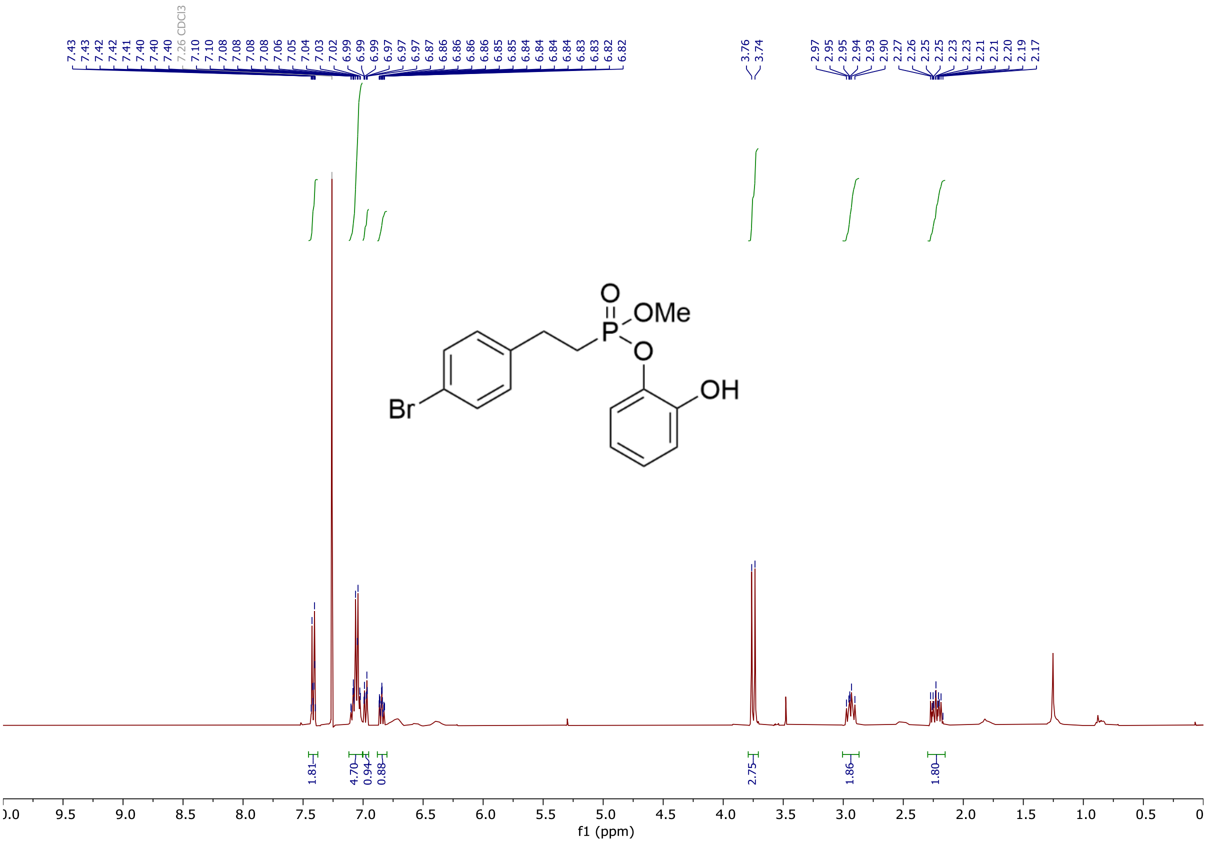

^{13}C NMR (101 MHz, $CDCl_3$): **3w**

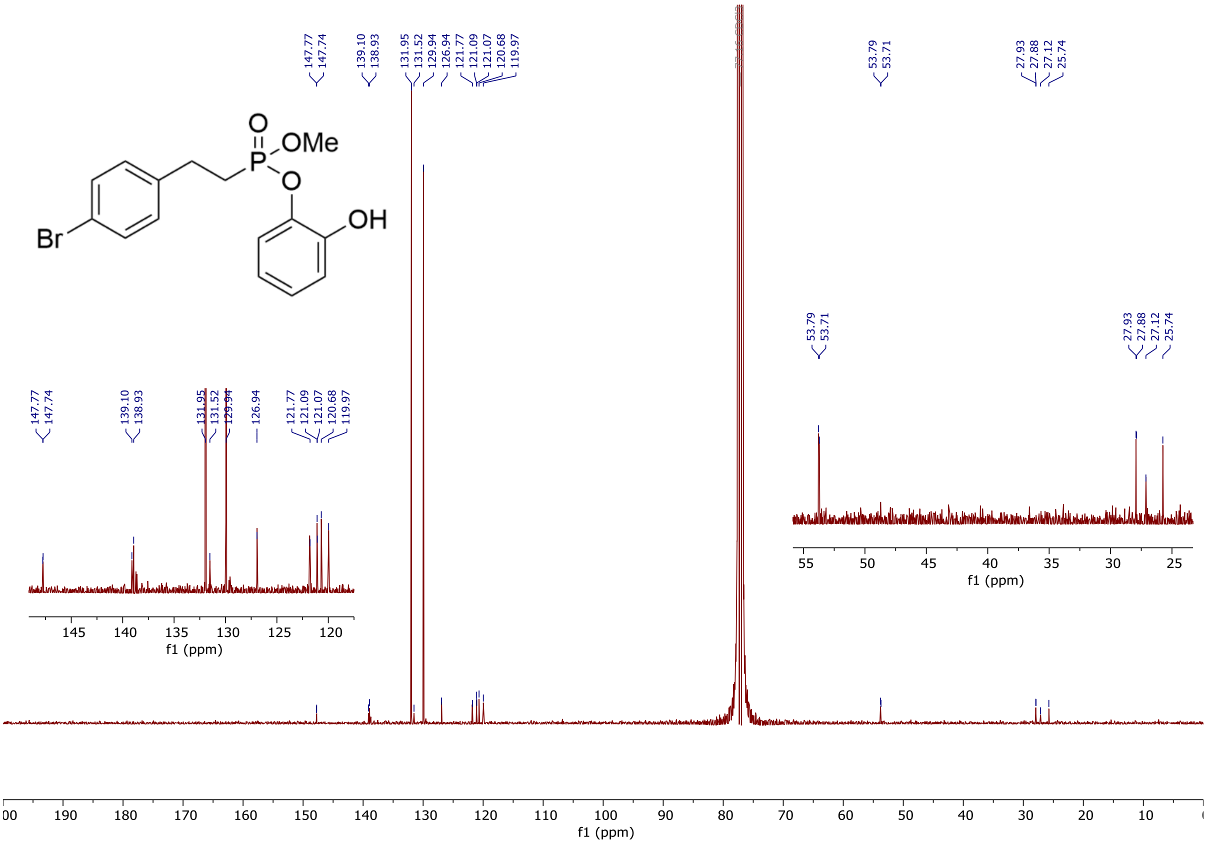

^{31}P NMR (162 MHz, $CDCl_3$): **3w**

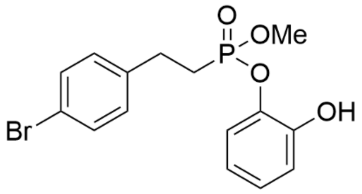

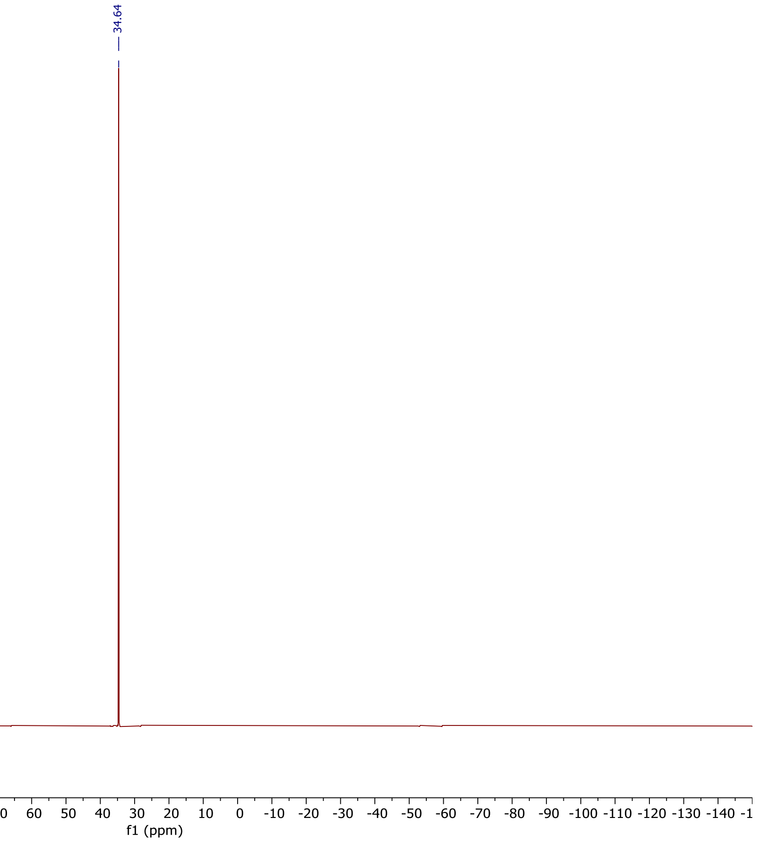

¹H NMR (400 MHz, CDCl$_3$): **3x**

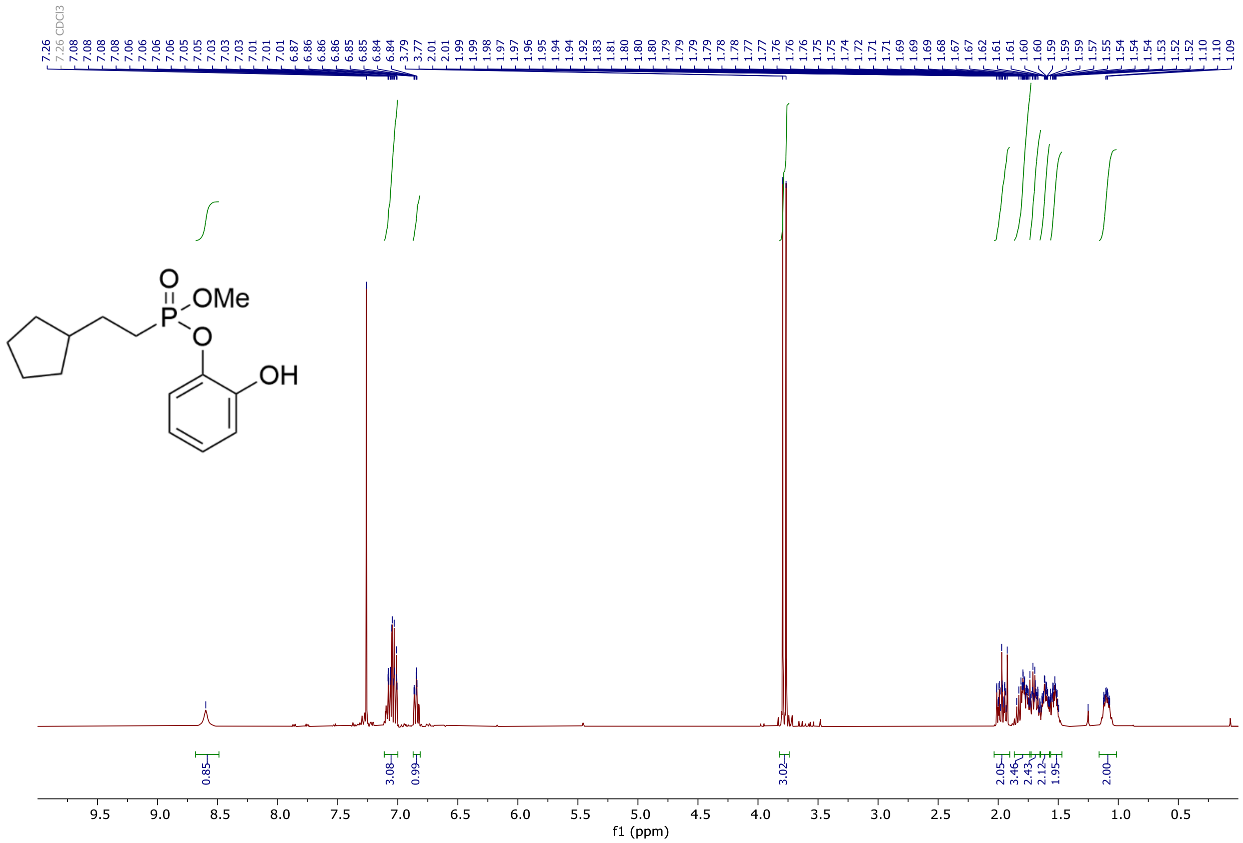

¹³C NMR (101 MHz, CDCl$_3$): **3x**

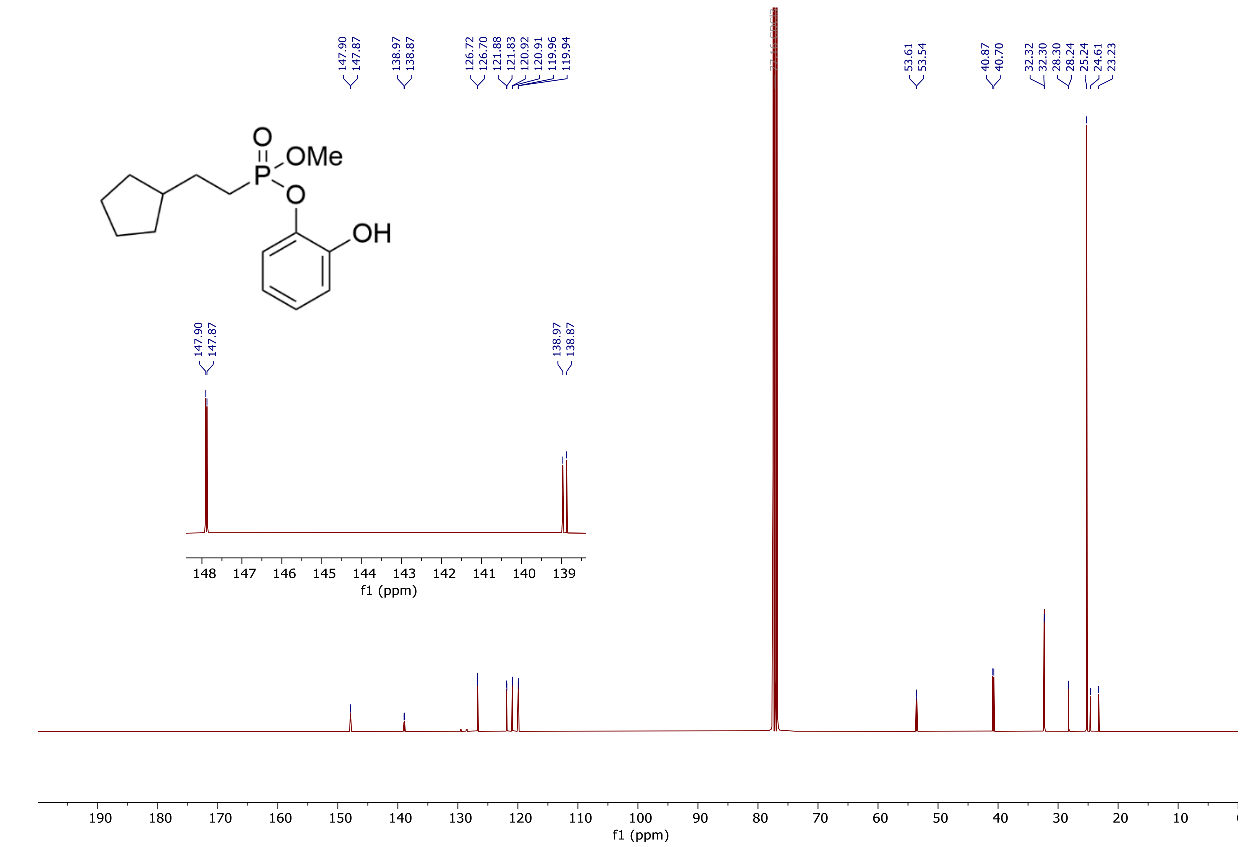

^{31}P NMR (162 MHz, $CDCl_3$): **3x**

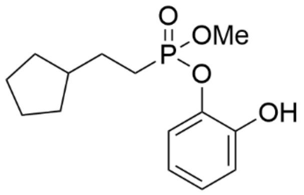

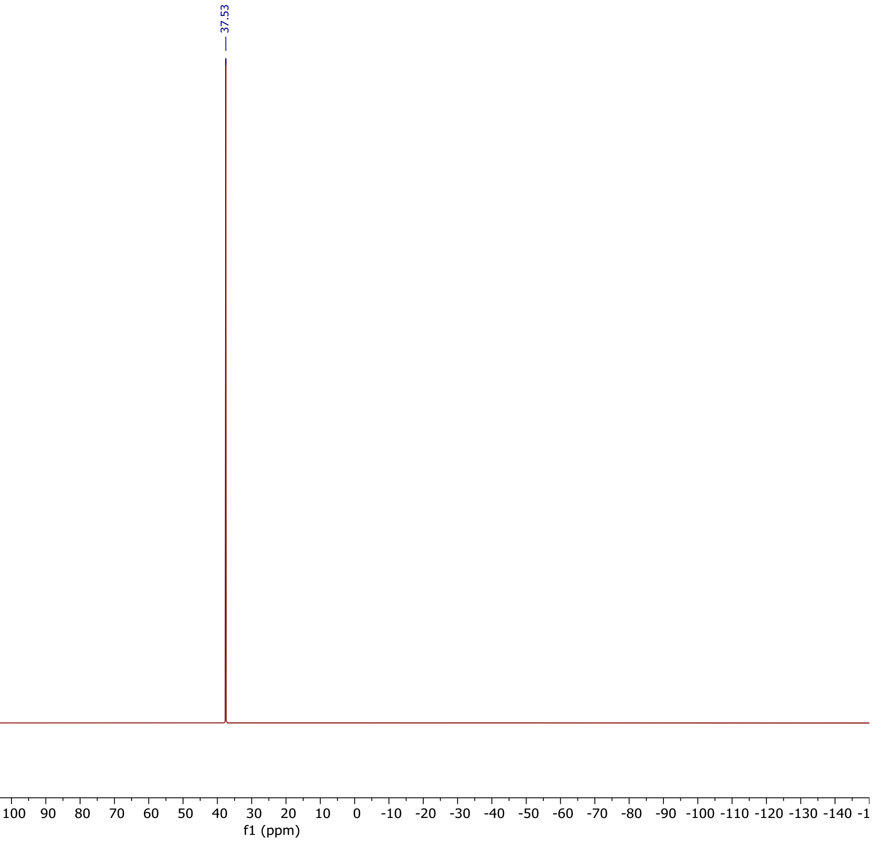

^{1}H NMR (500 MHz, $CDCl_3$): **3y**

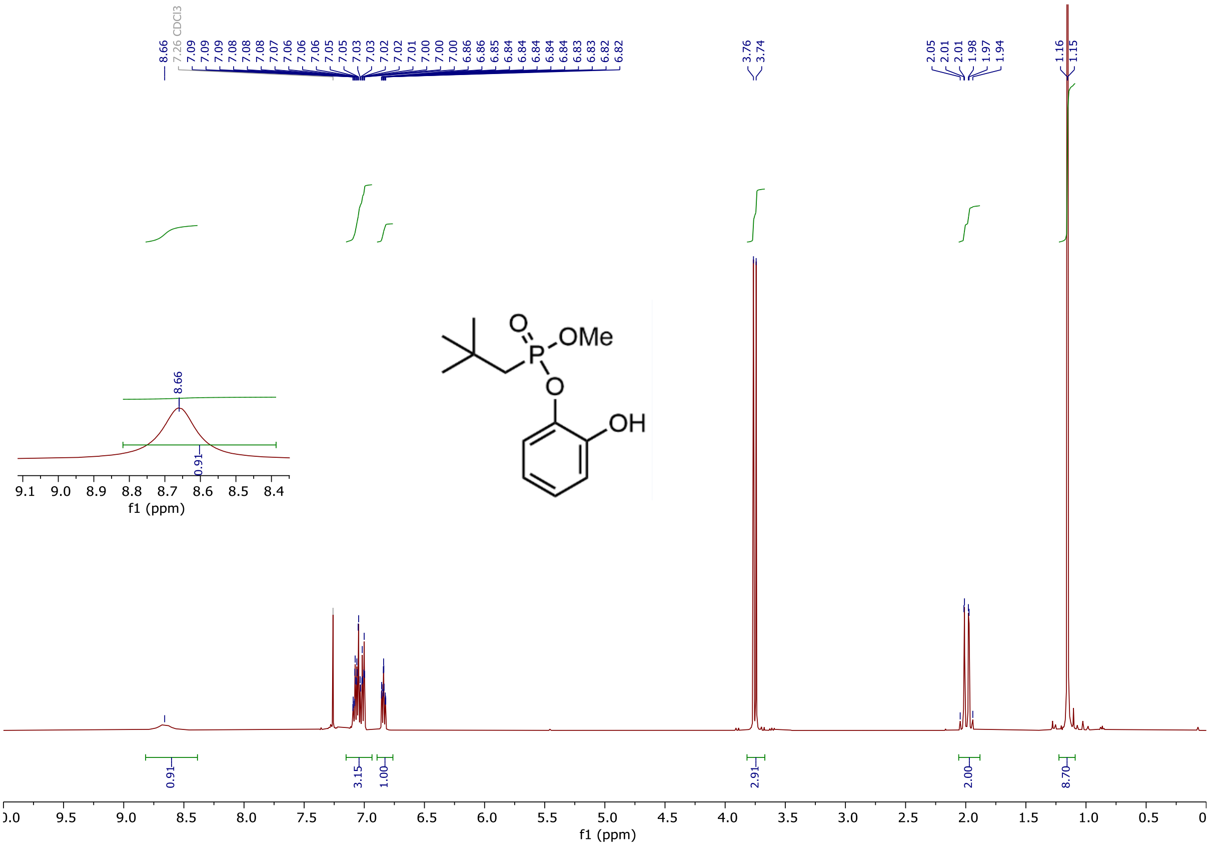

^{13}C NMR (126 MHz, $CDCl_3$): **3y**

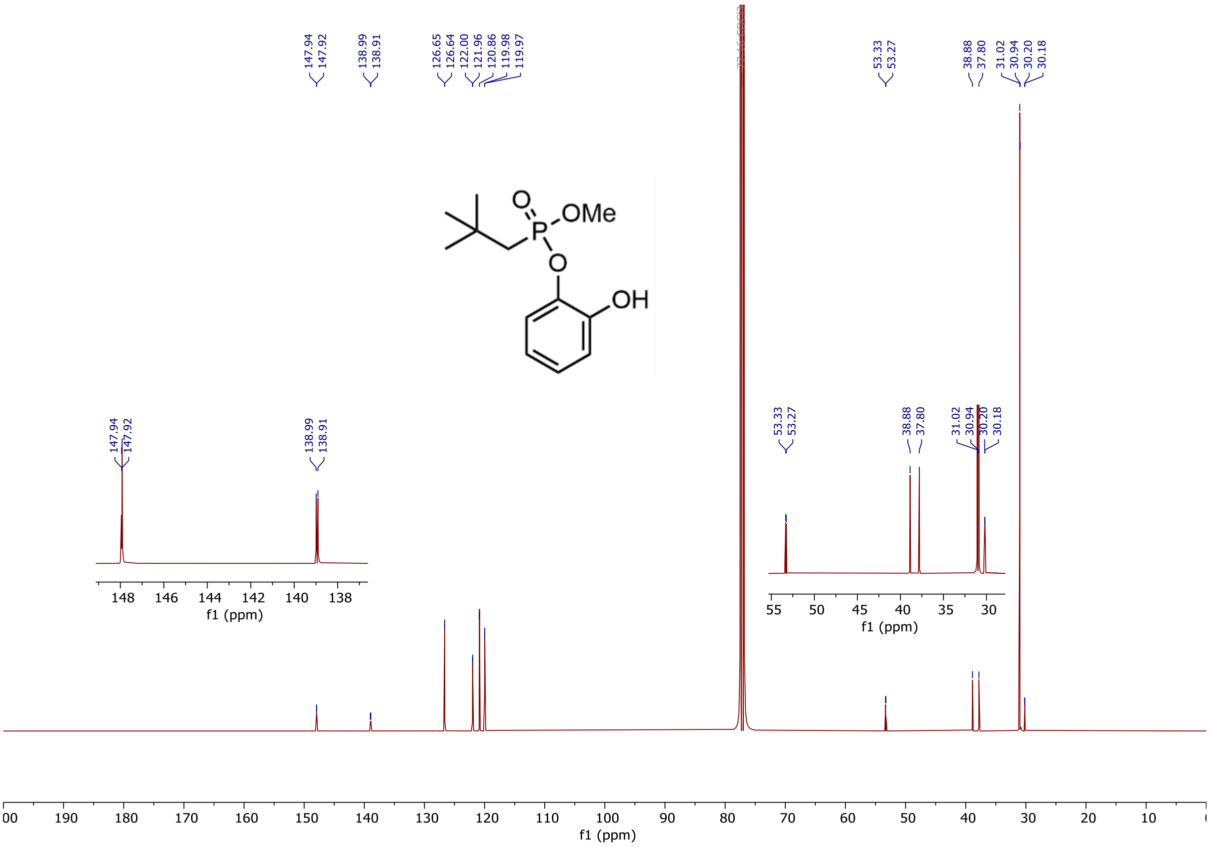

^{31}P NMR (162 MHz, $CDCl_3$): **3y**

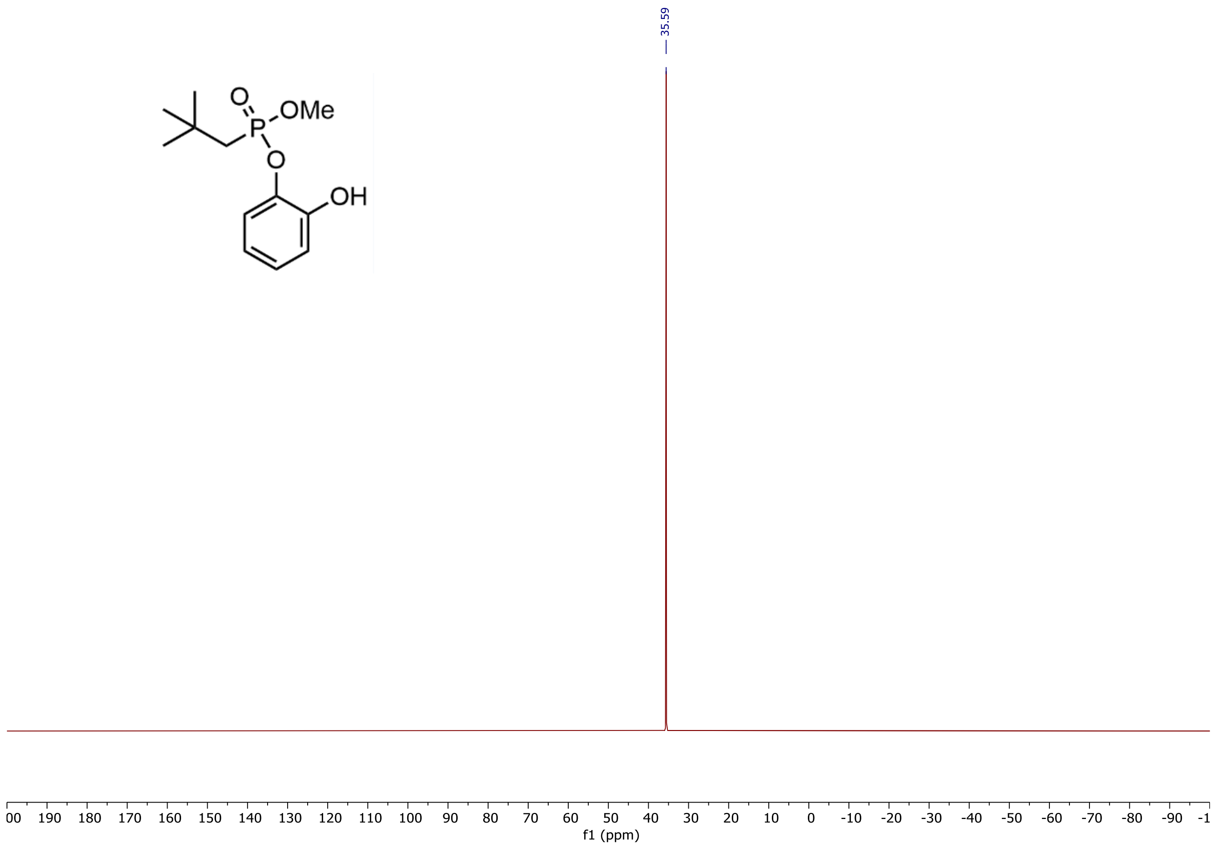

^{1}H NMR (400 MHz, $CDCl_3$): **3z**

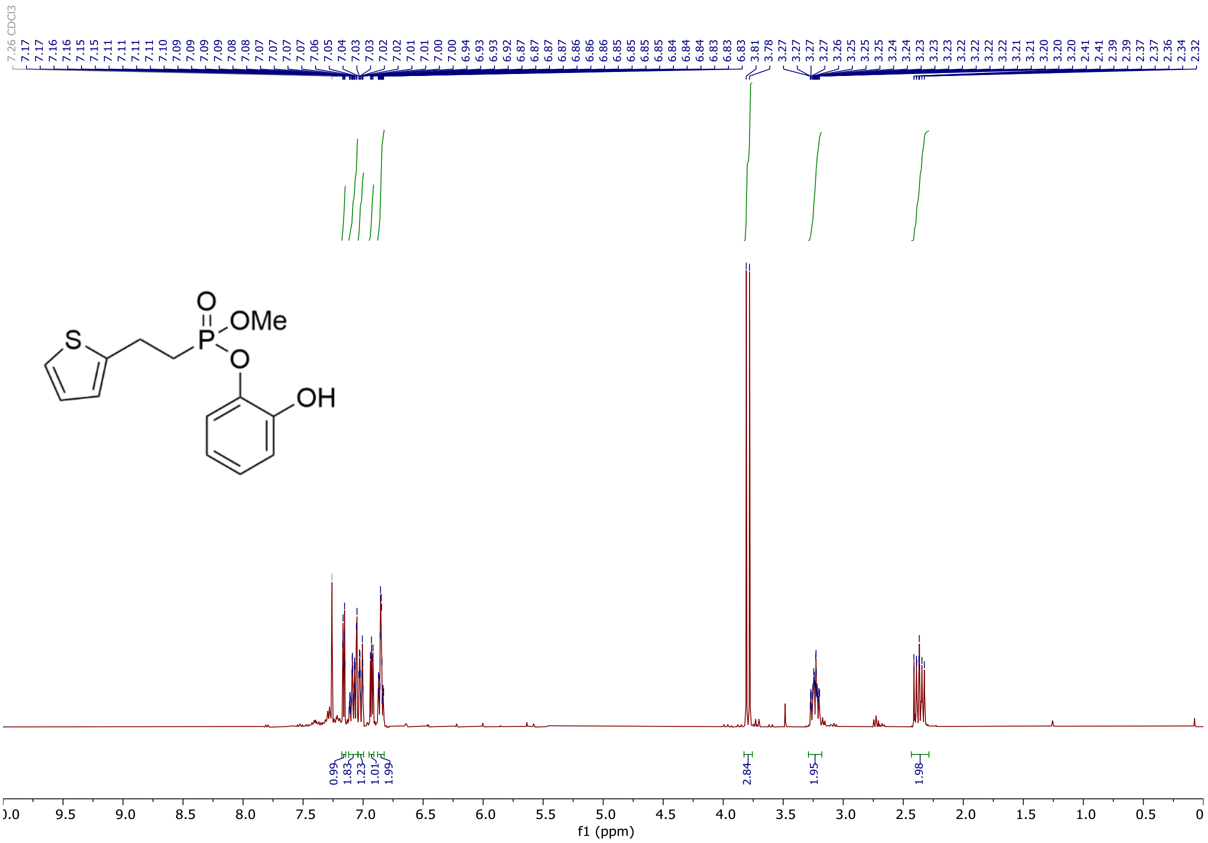

^{13}C NMR (101 MHz, $CDCl_3$): **3z**

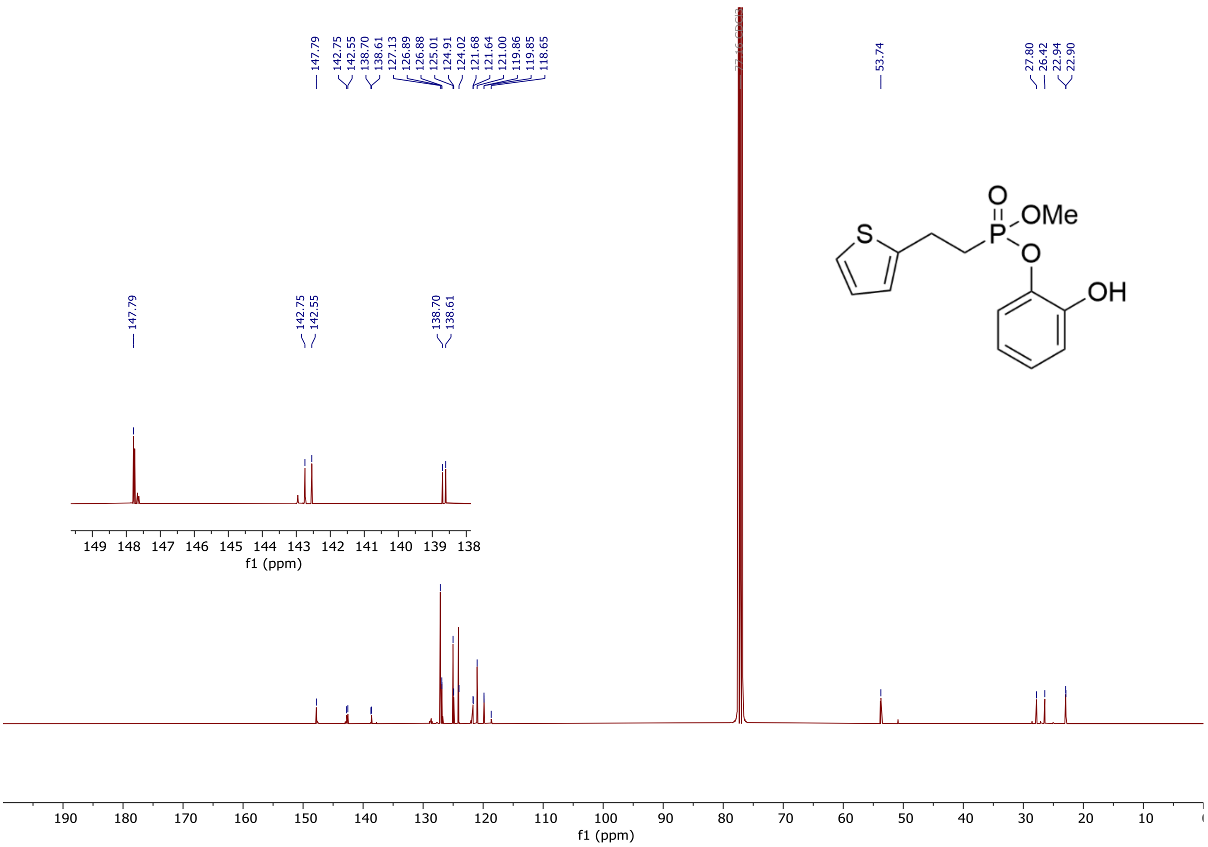

^{31}**P NMR** (162 MHz, $CDCl_3$): **3z**

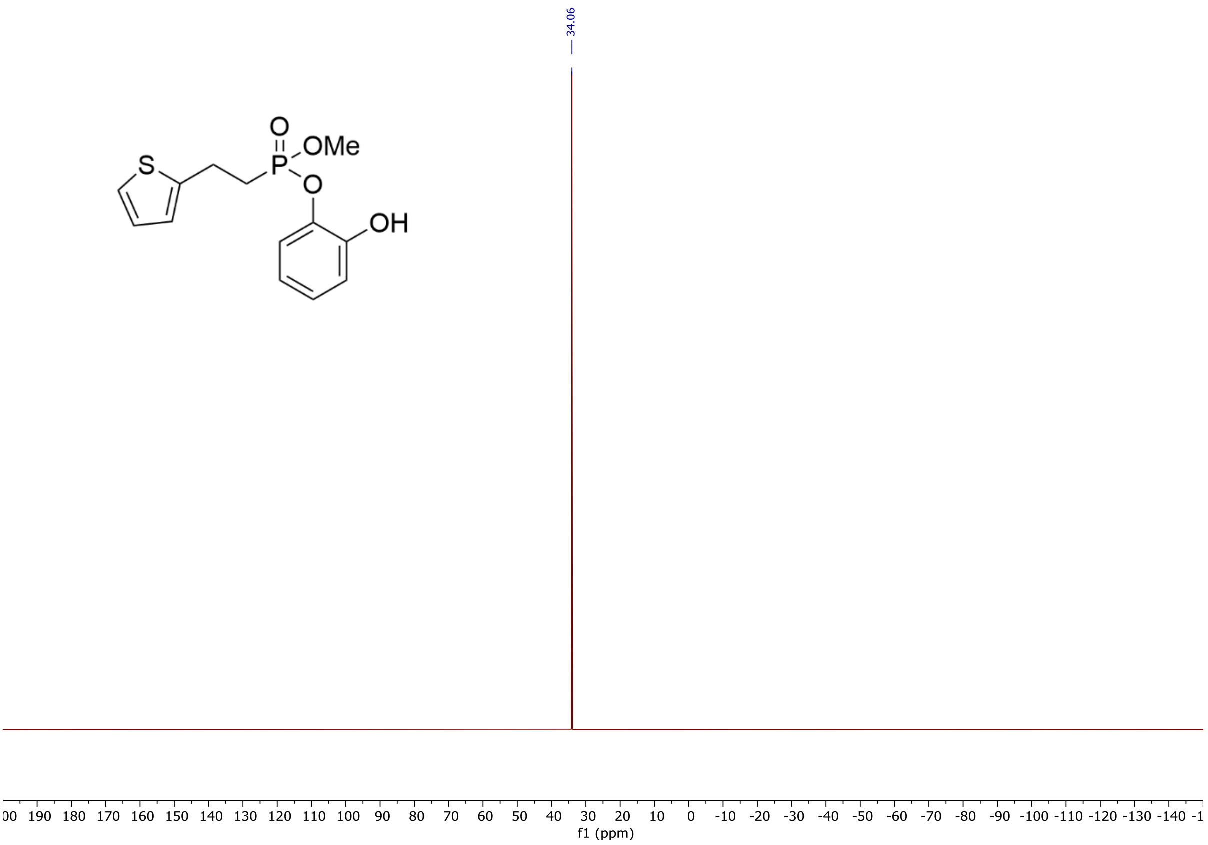

¹H NMR (400 MHz, CDCl₃): **3aa**

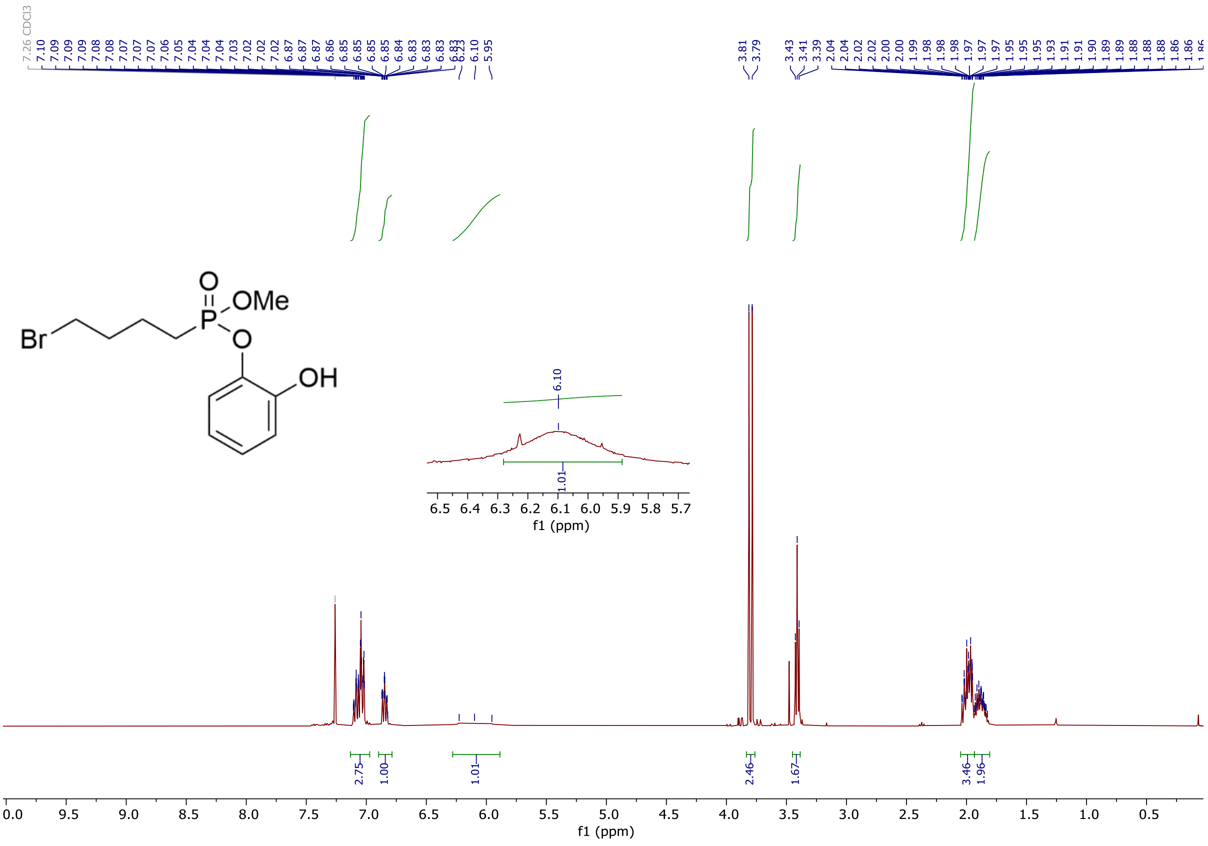

¹³C NMR (101 MHz, CDCl₃): **3aa**

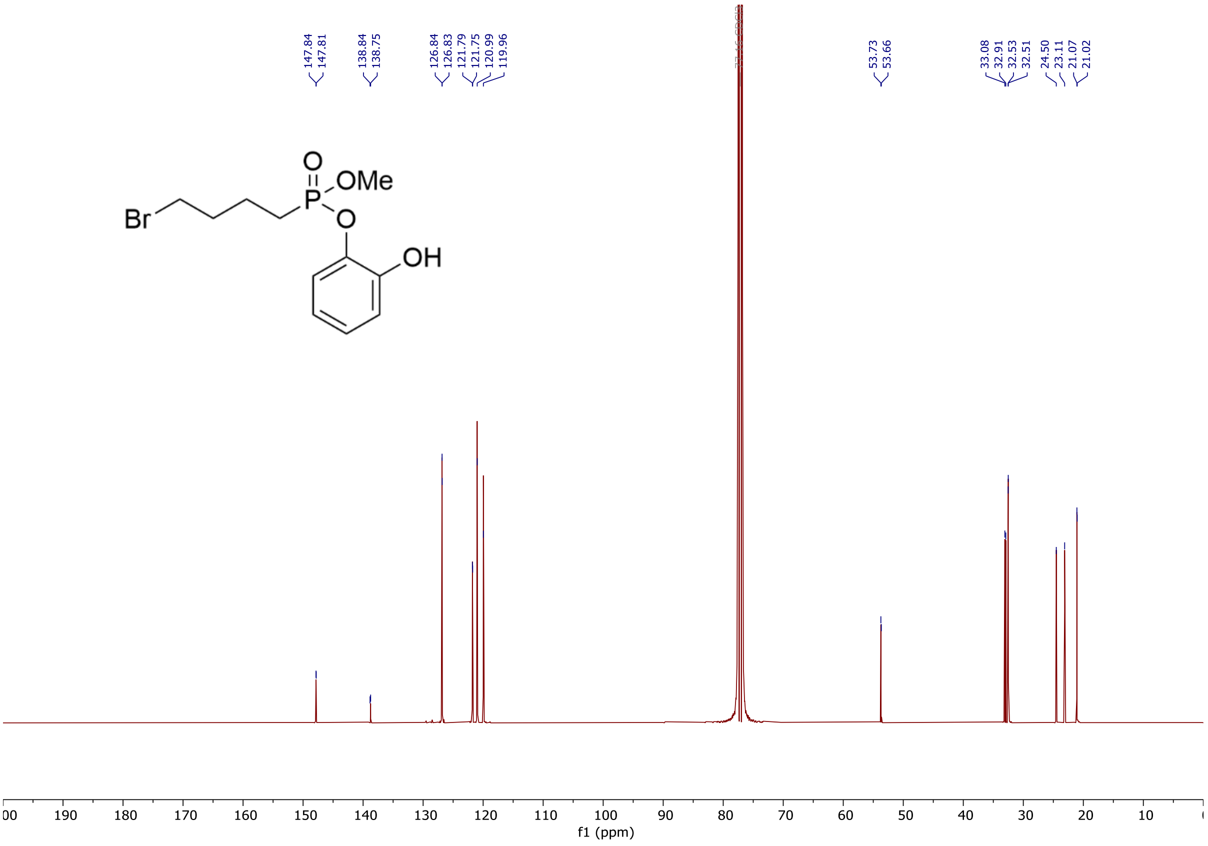

^{31}P NMR (162 MHz, $CDCl_3$): **3aa**

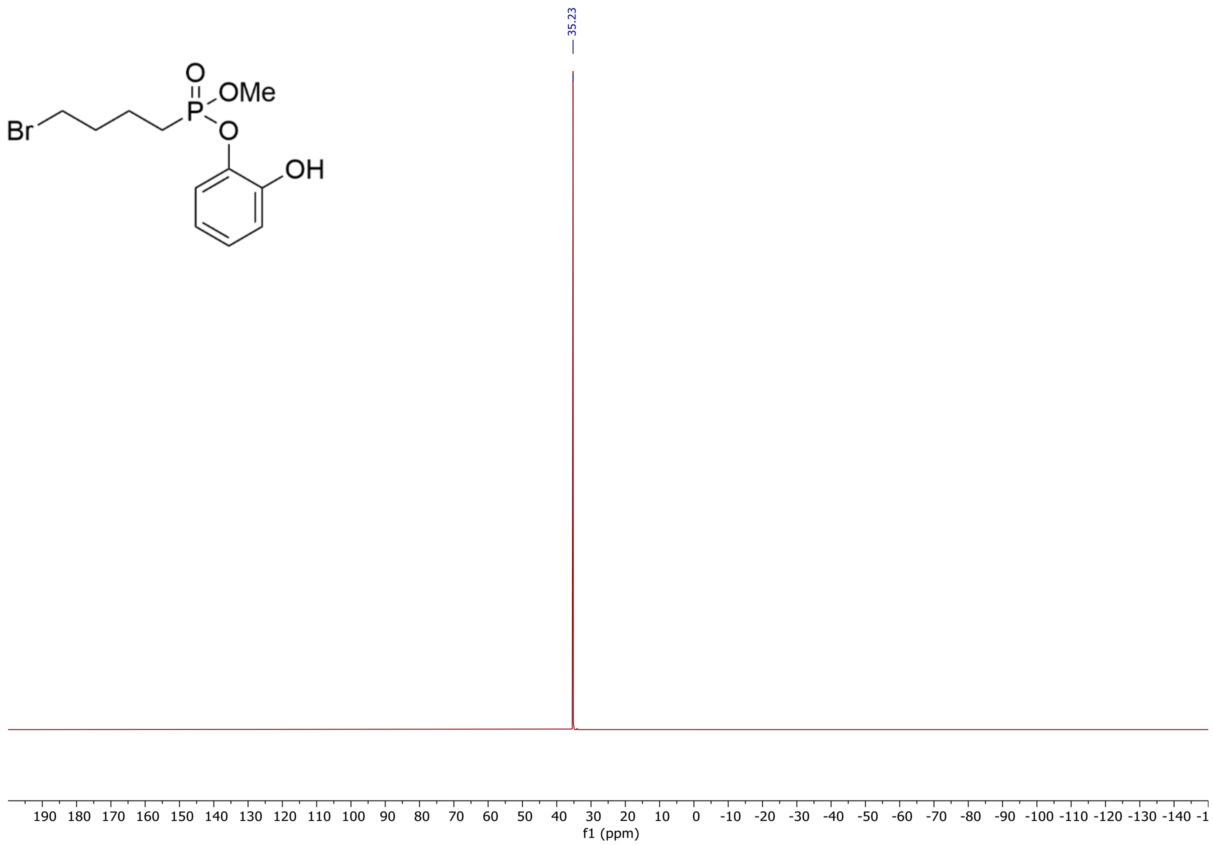

^{1}H NMR (400 MHz, $CDCl_3$): **3ab**

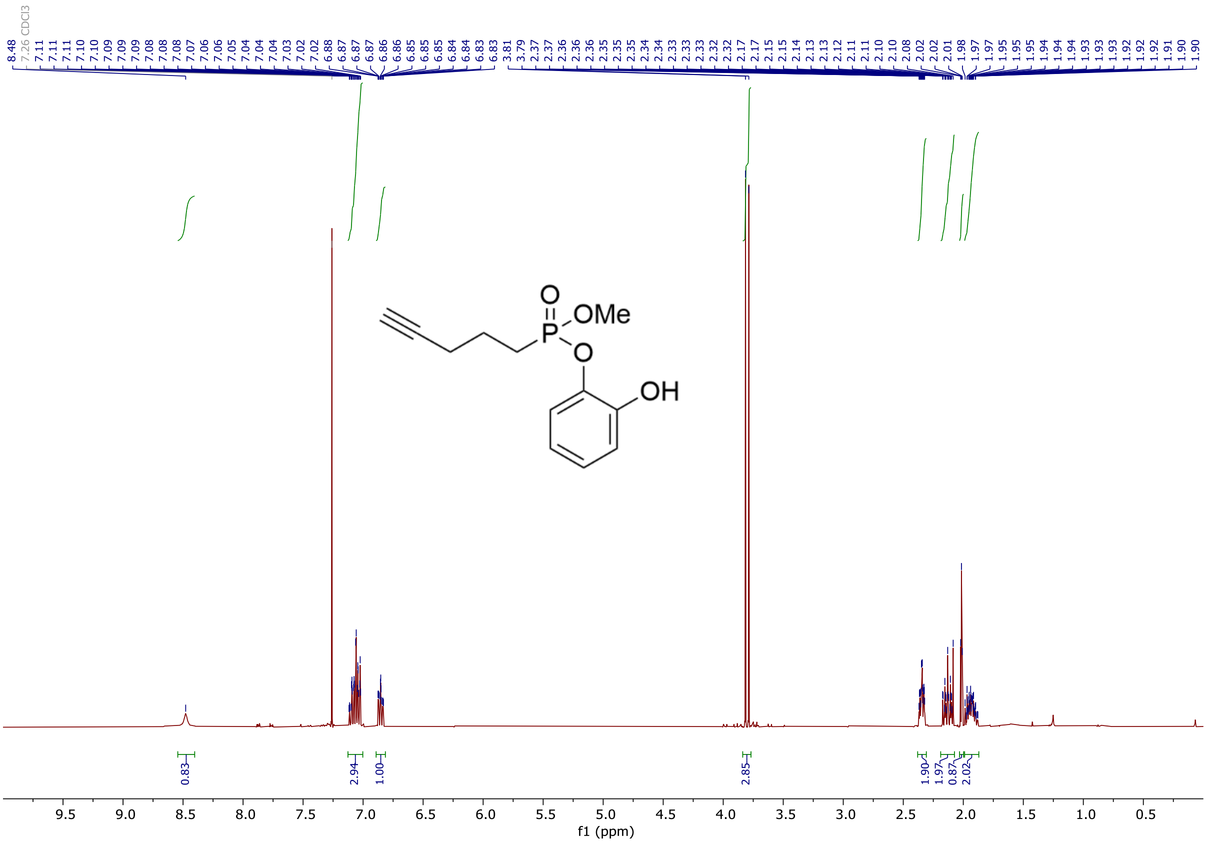

^{13}C NMR (101 MHz, $CDCl_3$): **3ab**

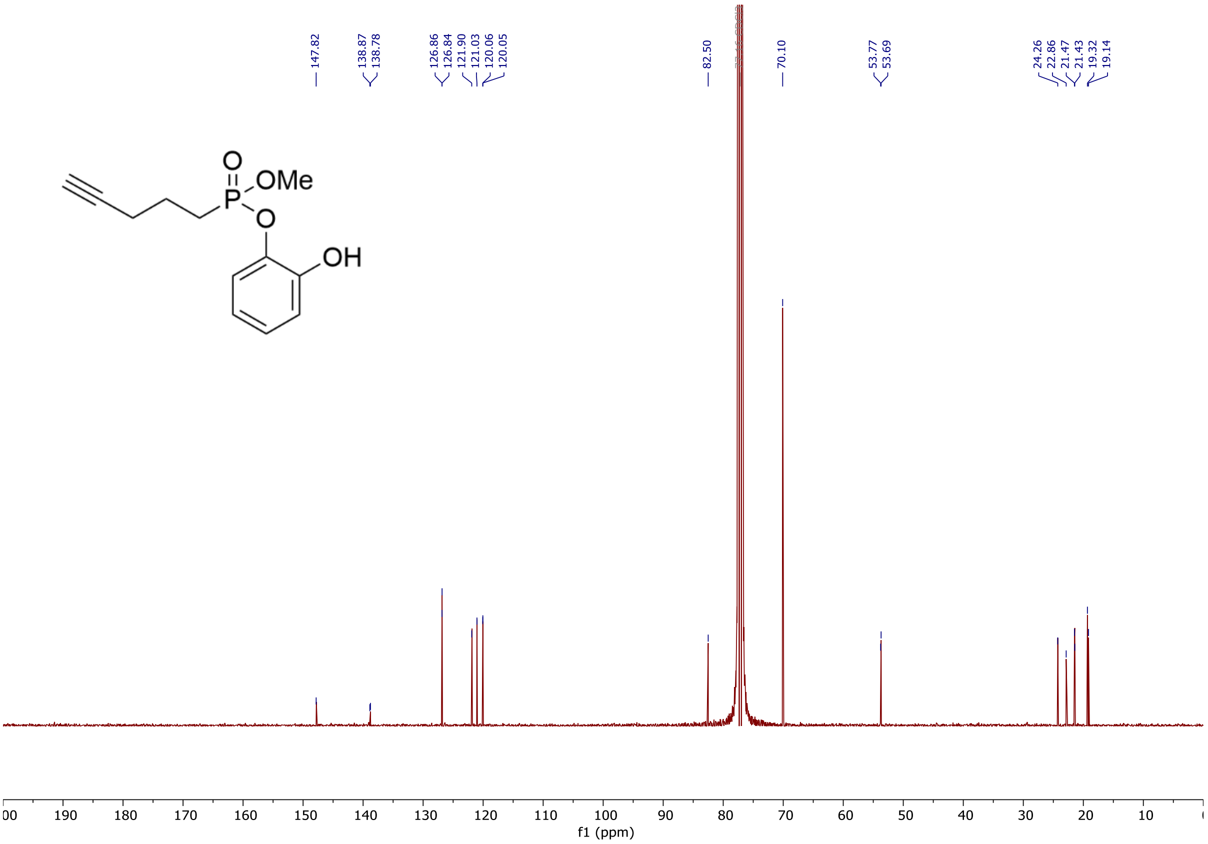

^{31}**P NMR** (162 MHz, $CDCl_3$): **3ab**

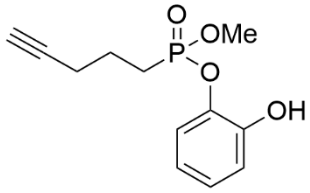

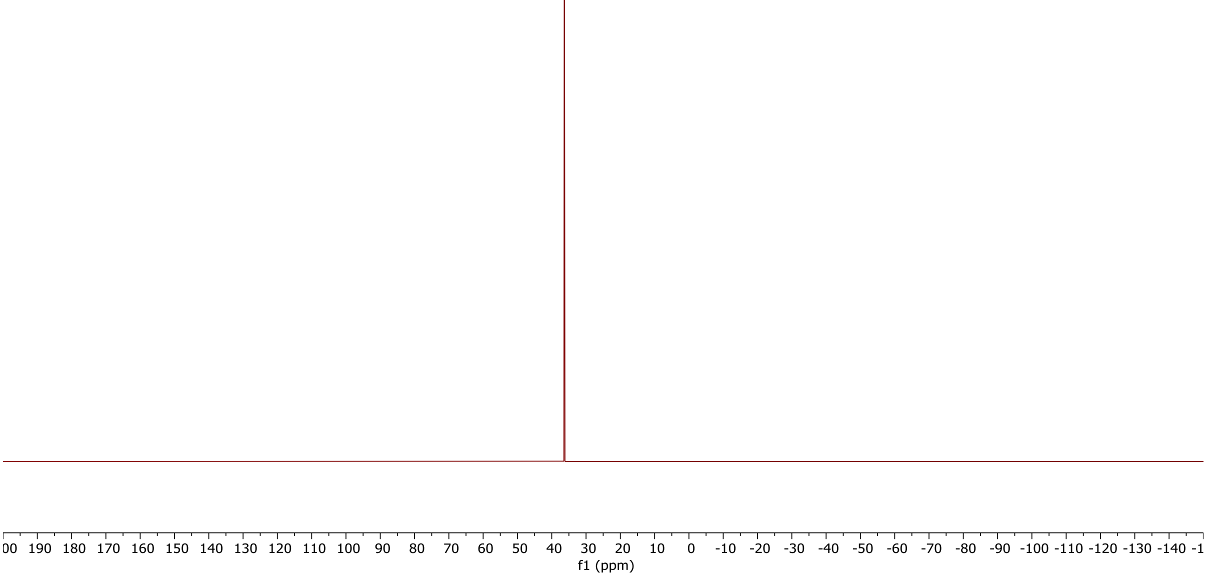

¹H NMR (400 MHz, $CDCl_3$): **3ac**

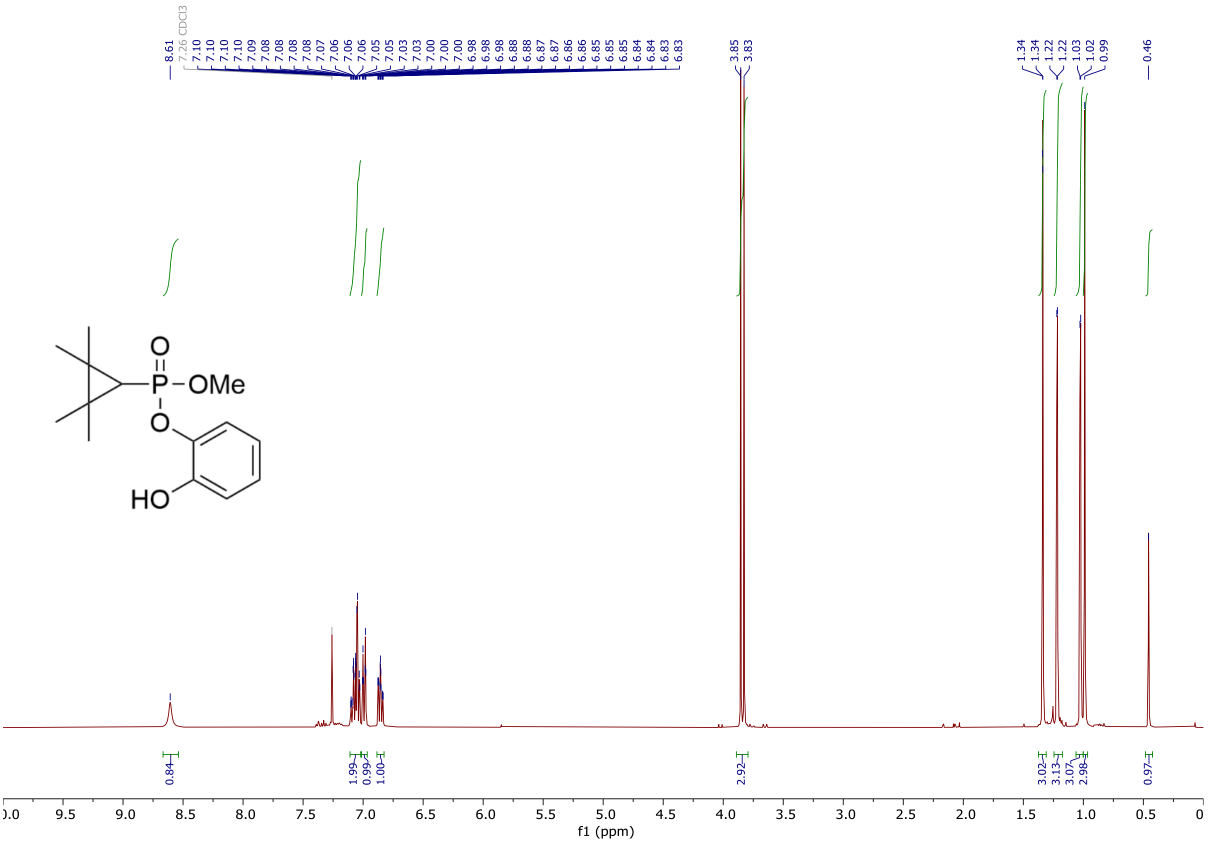

¹³C NMR (126 MHz, $CDCl_3$): **3ac**

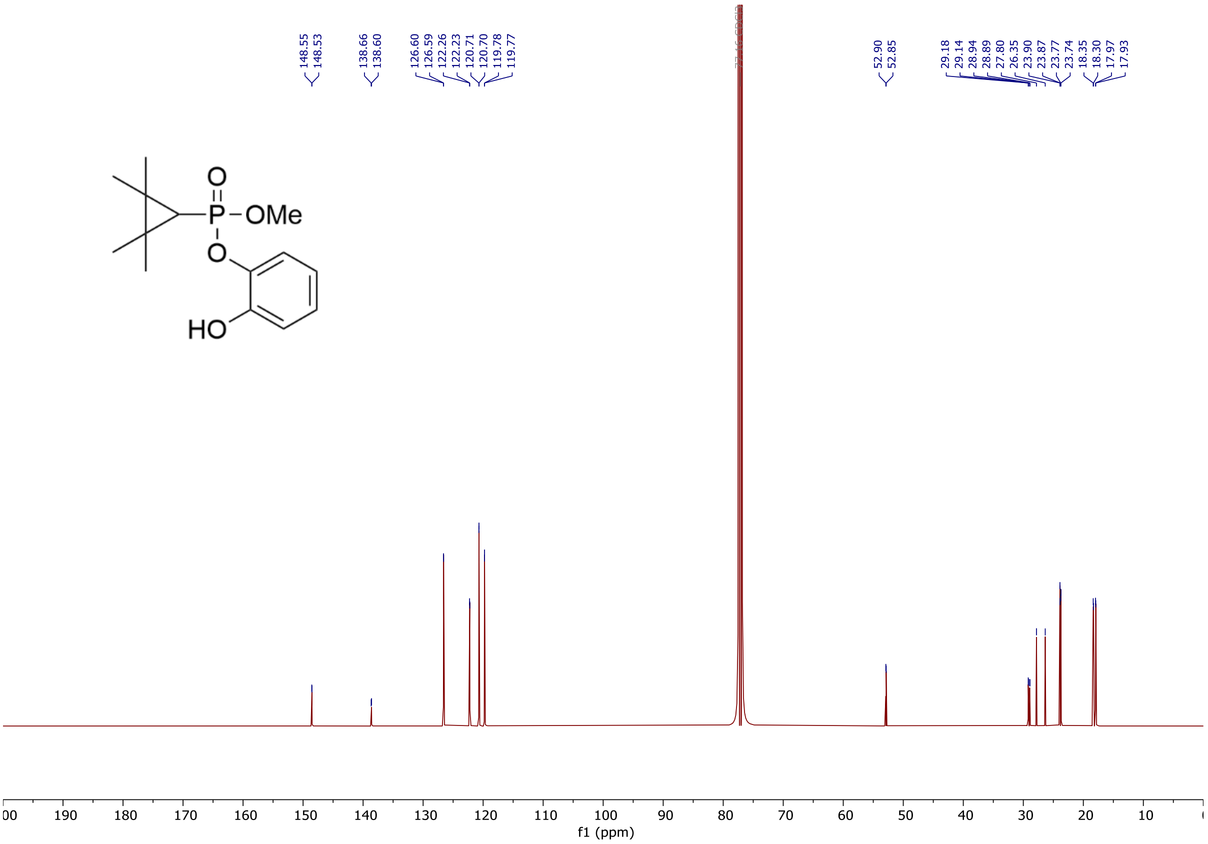

^{31}P NMR (162 MHz, $CDCl_3$): **3ac**

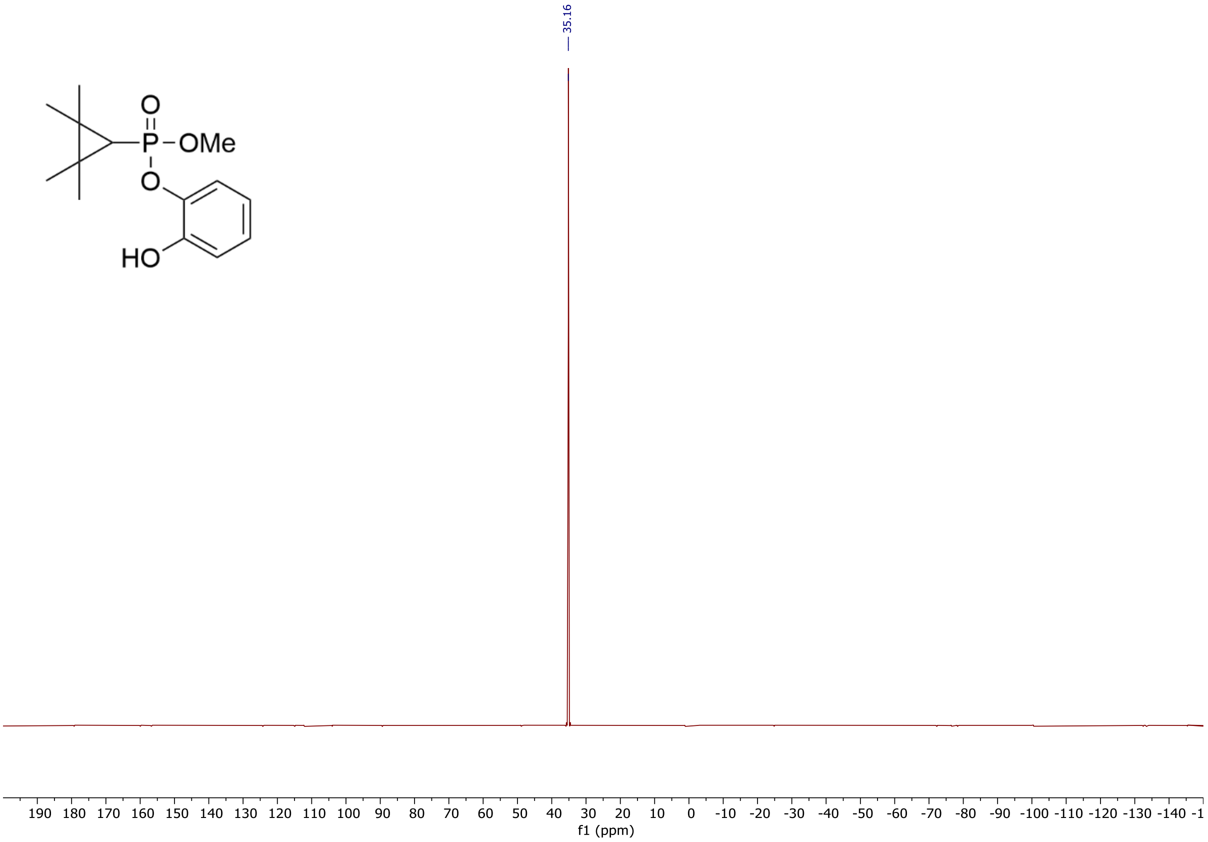

^{1}H NMR (400 MHz, $CDCl_3$): **3ad**

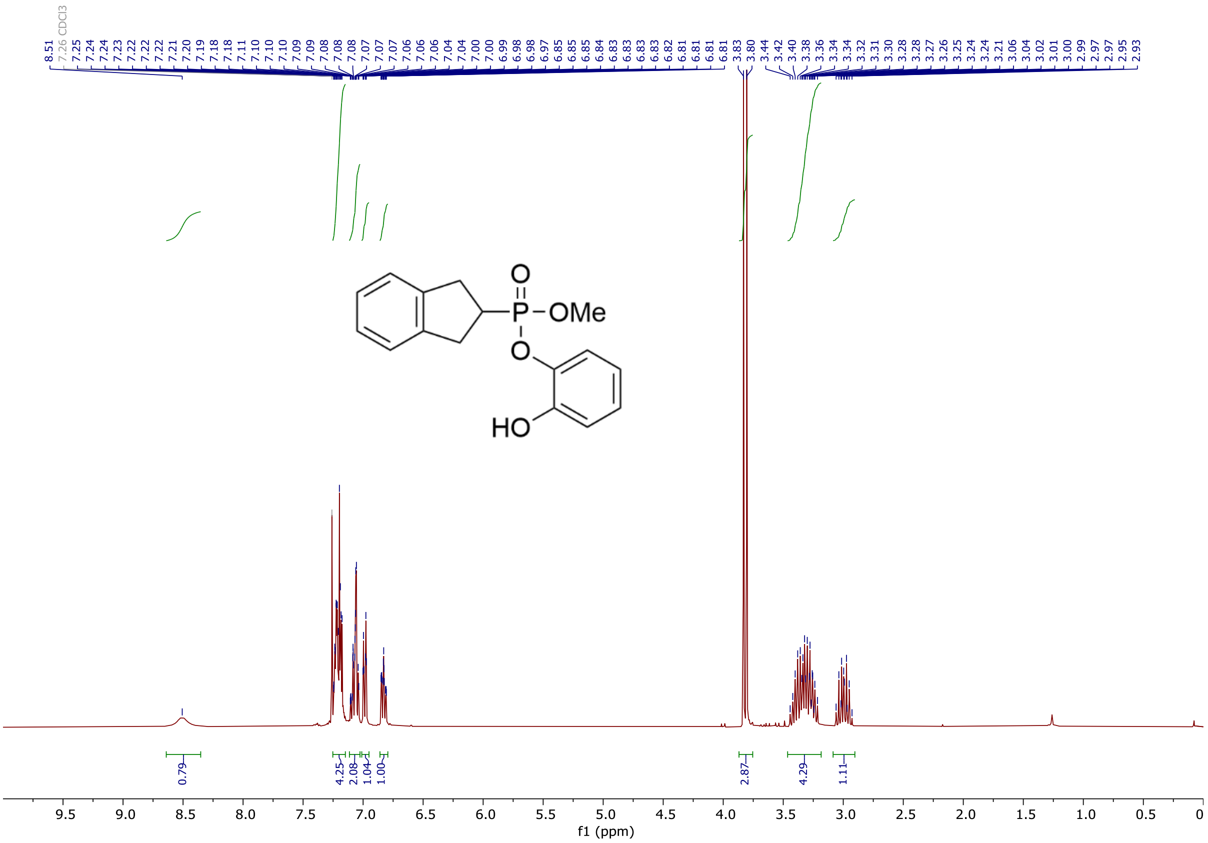

^{13}C NMR (101 MHz, $CDCl_3$): **3ad**

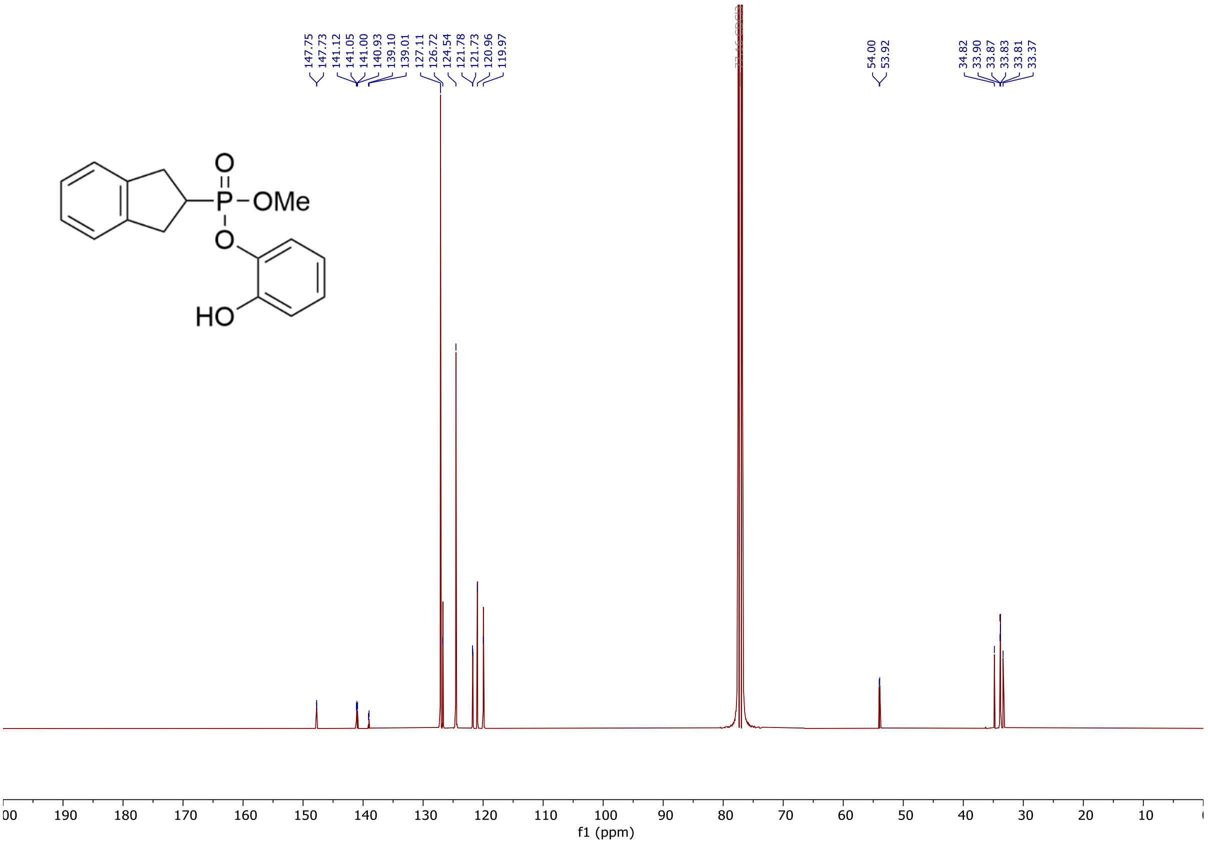

^{31}**P NMR** (162 MHz, $CDCl_3$): **3ad**

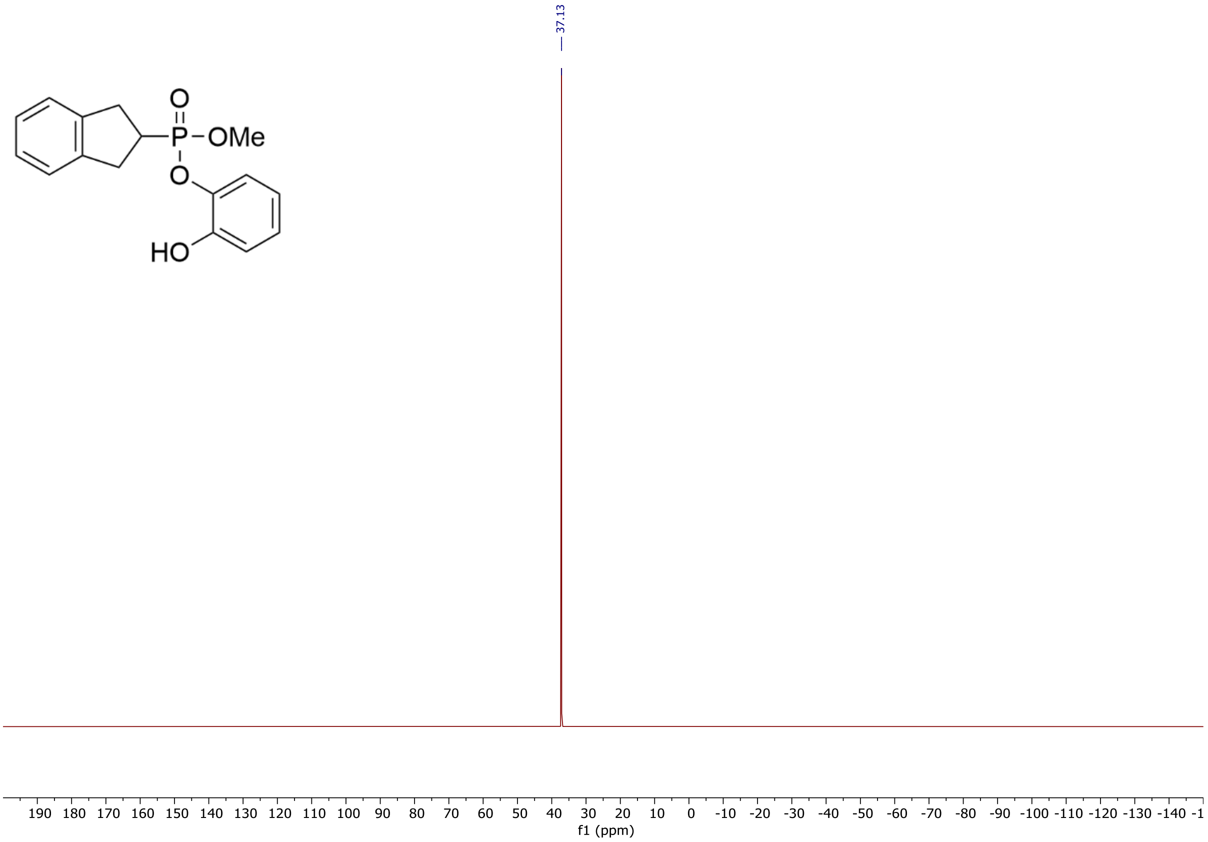

¹H NMR (400 MHz, CDCl₃): **3af**

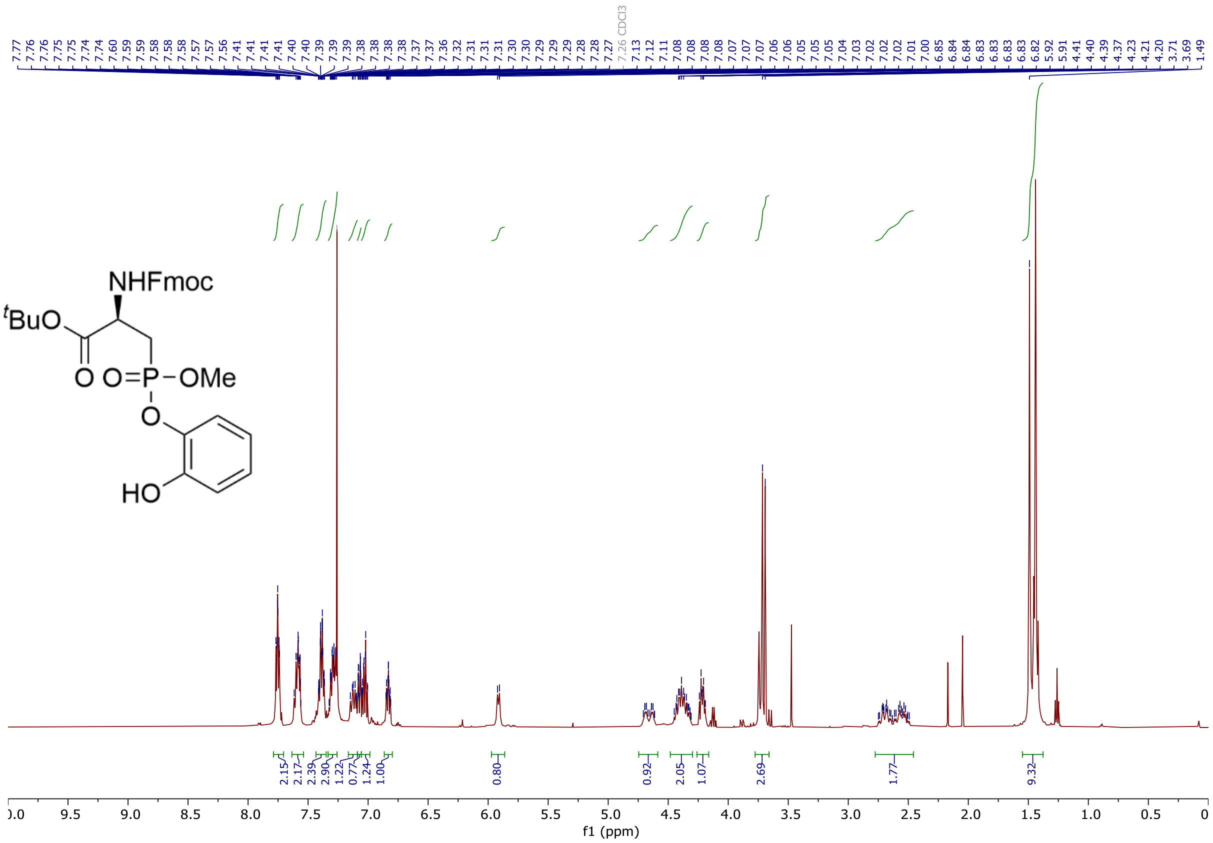

¹³C NMR (101 MHz, CDCl₃): **3af**

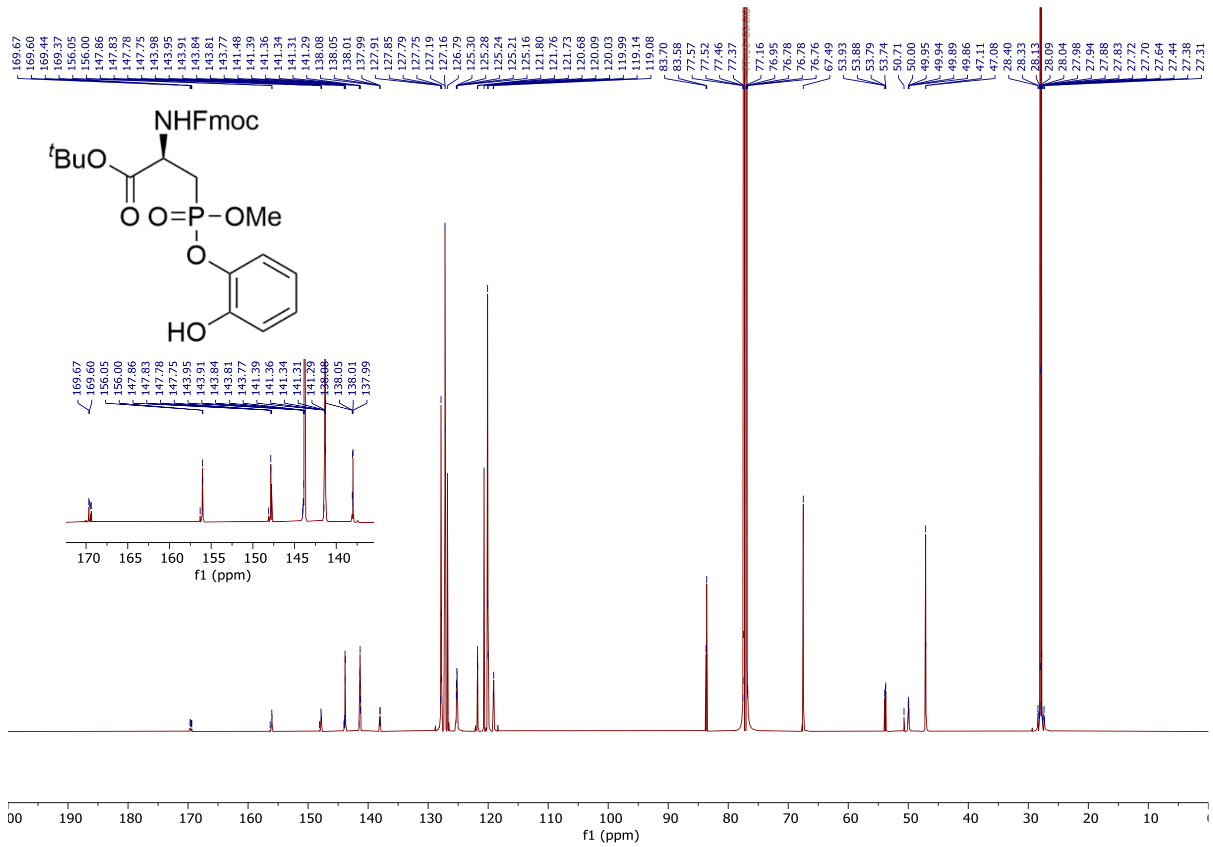

^{31}P NMR (162 MHz, $CDCl_3$): **3af**

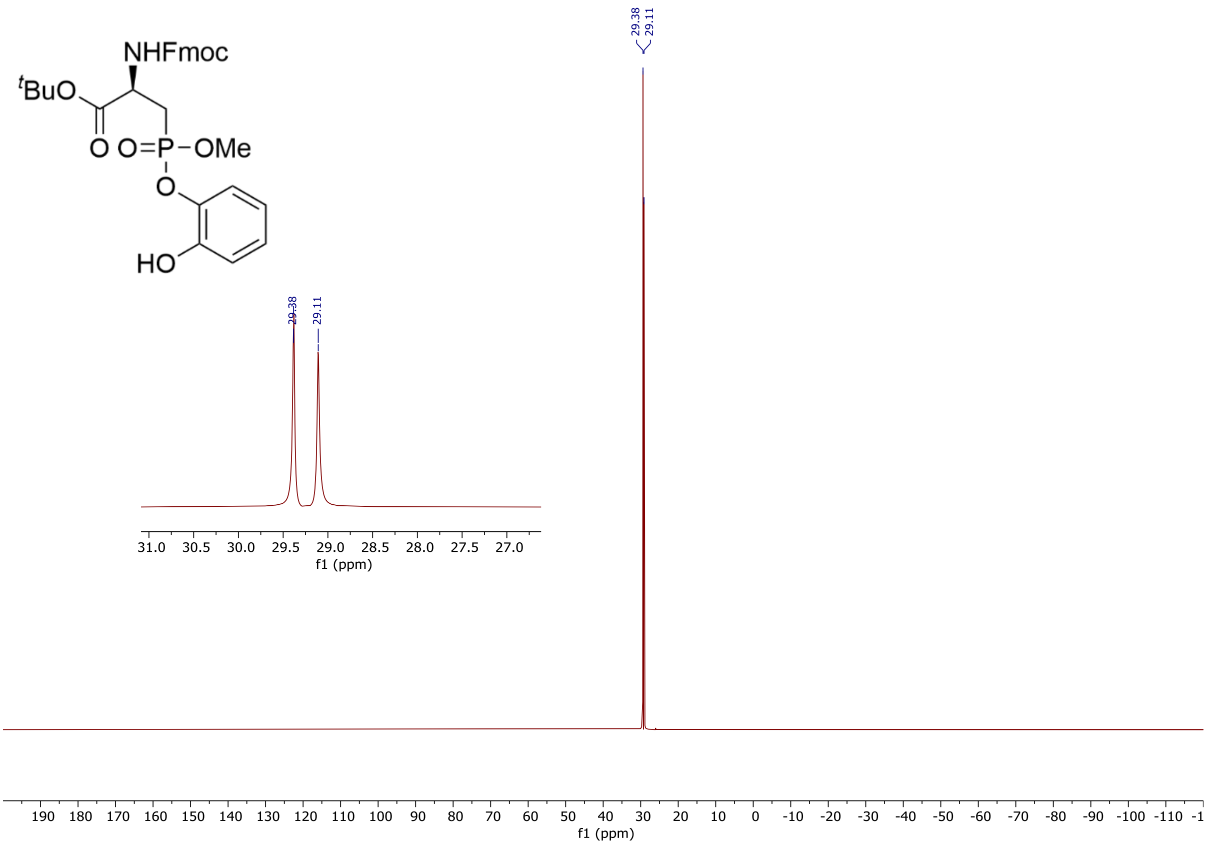

^{1}H NMR (400 MHz, $CDCl_3$): **3ag**

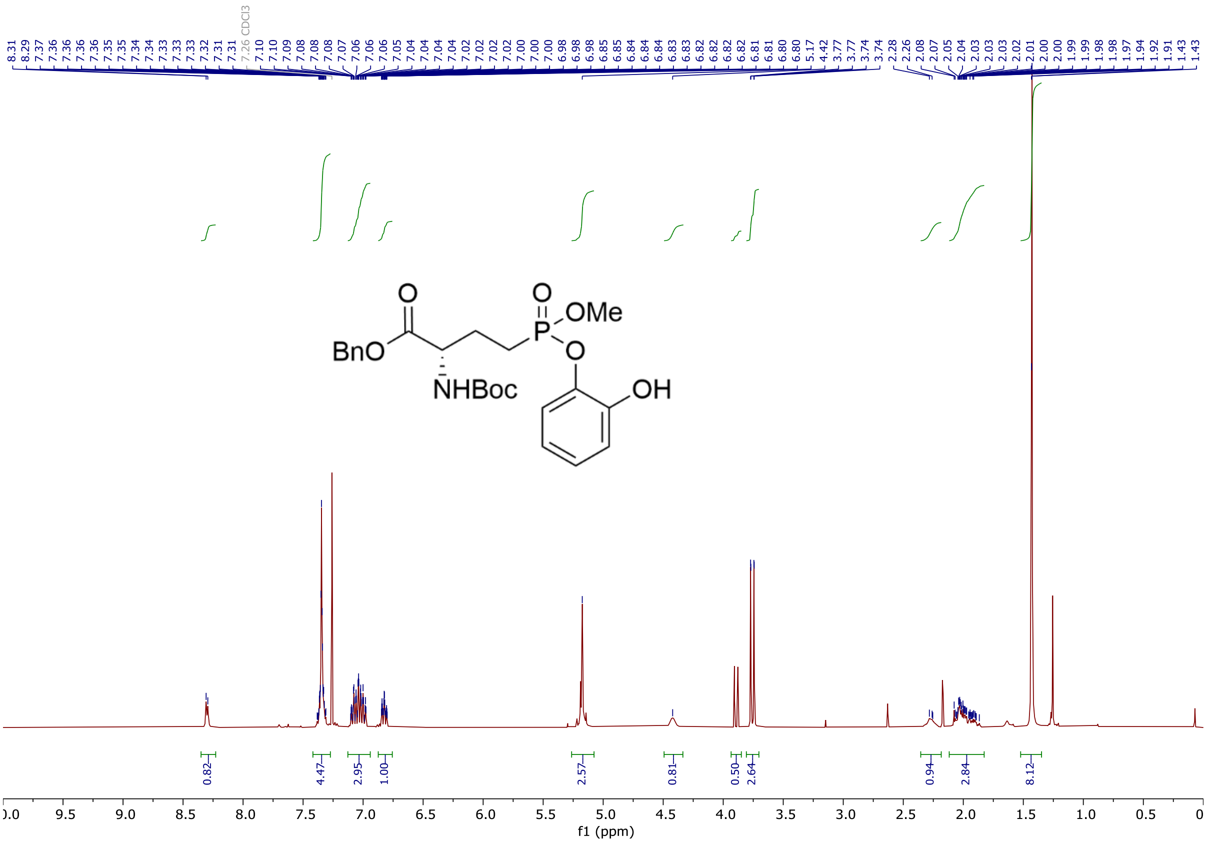

^{13}C NMR (101 MHz, $CDCl_3$): **3ag**

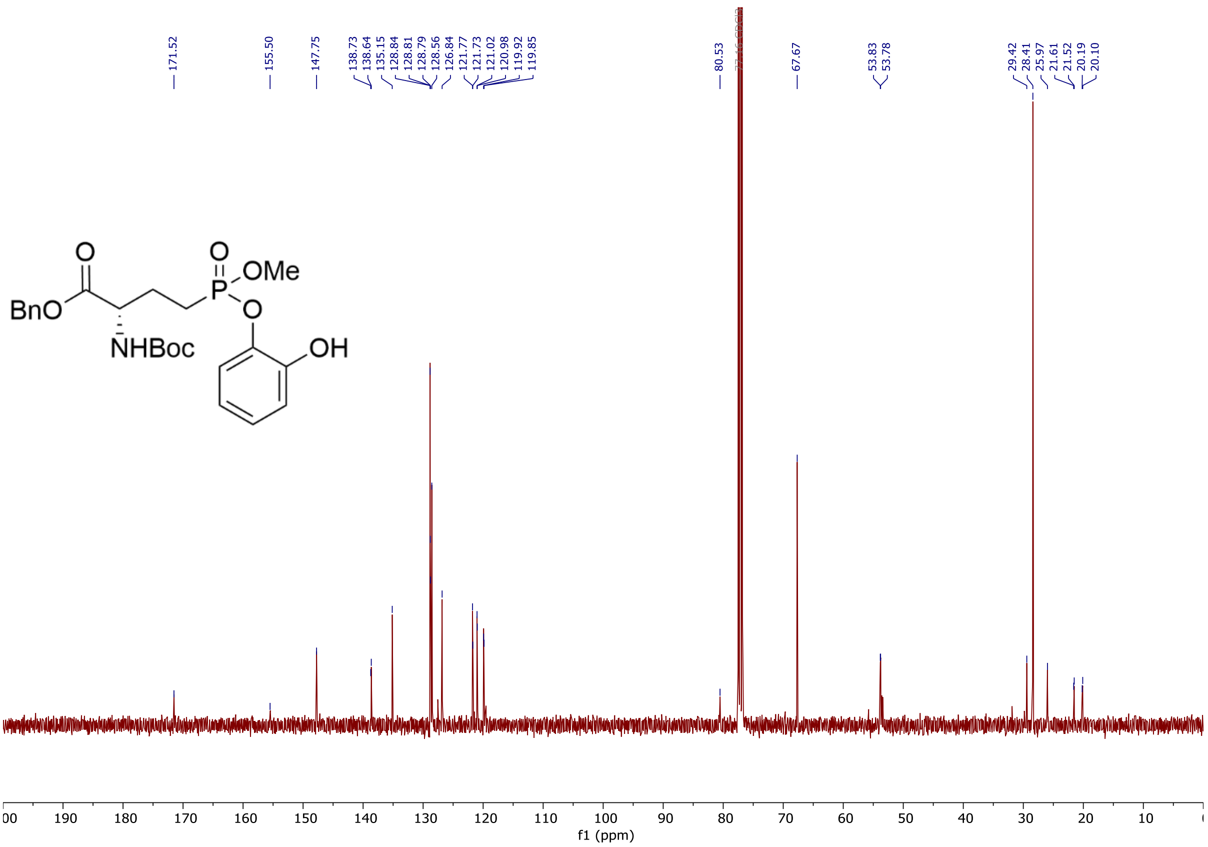

^{31}P NMR (162 MHz, $CDCl_3$): **3ag**

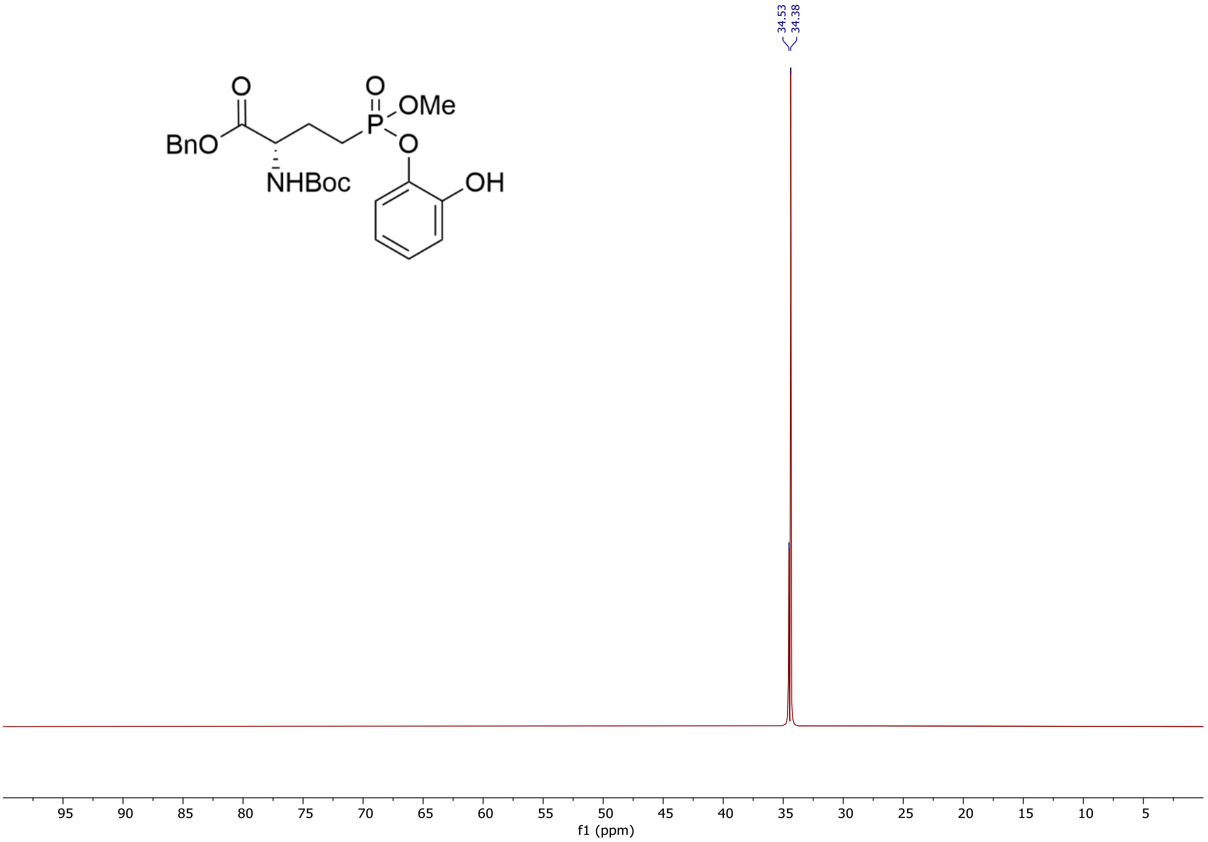

^{1}H NMR (400 MHz, CDCl3): **3ah**

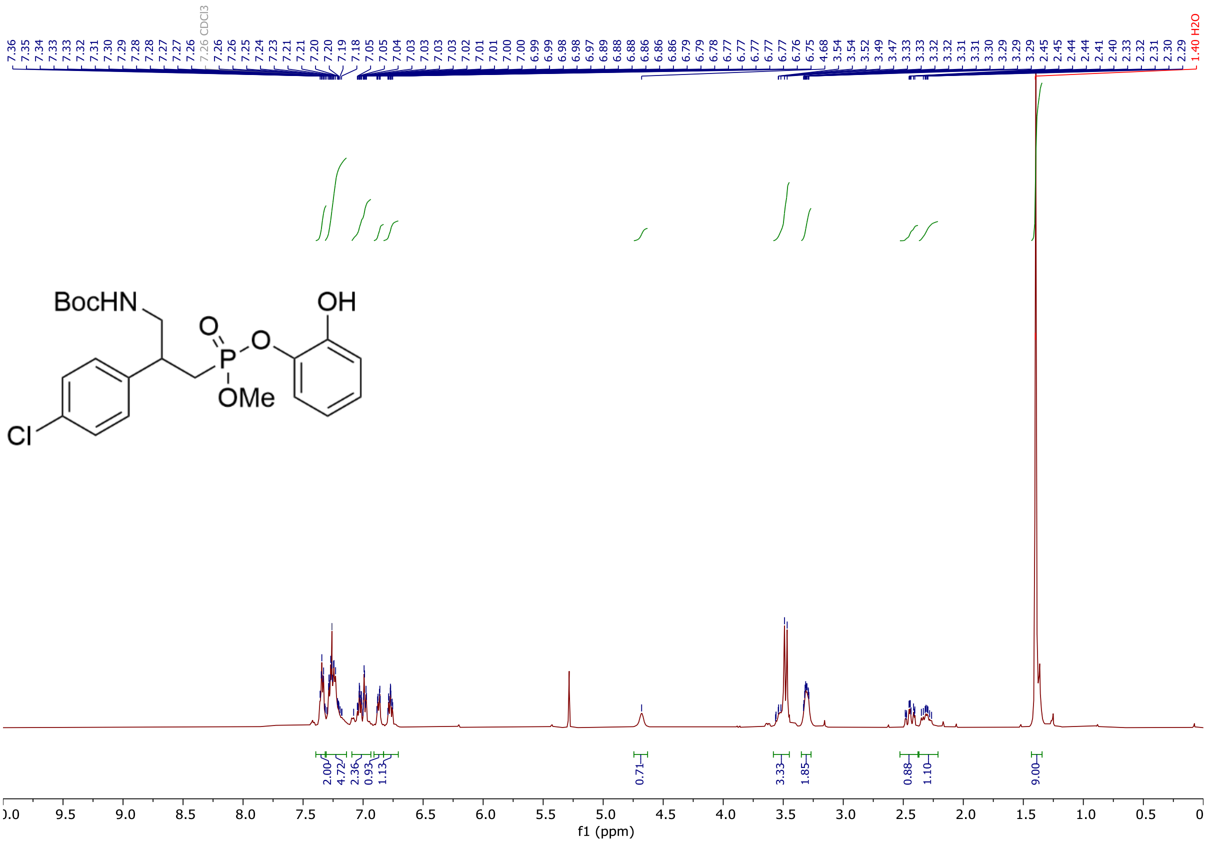

^{13}C NMR (101 MHz, CDCl3): **3ah**

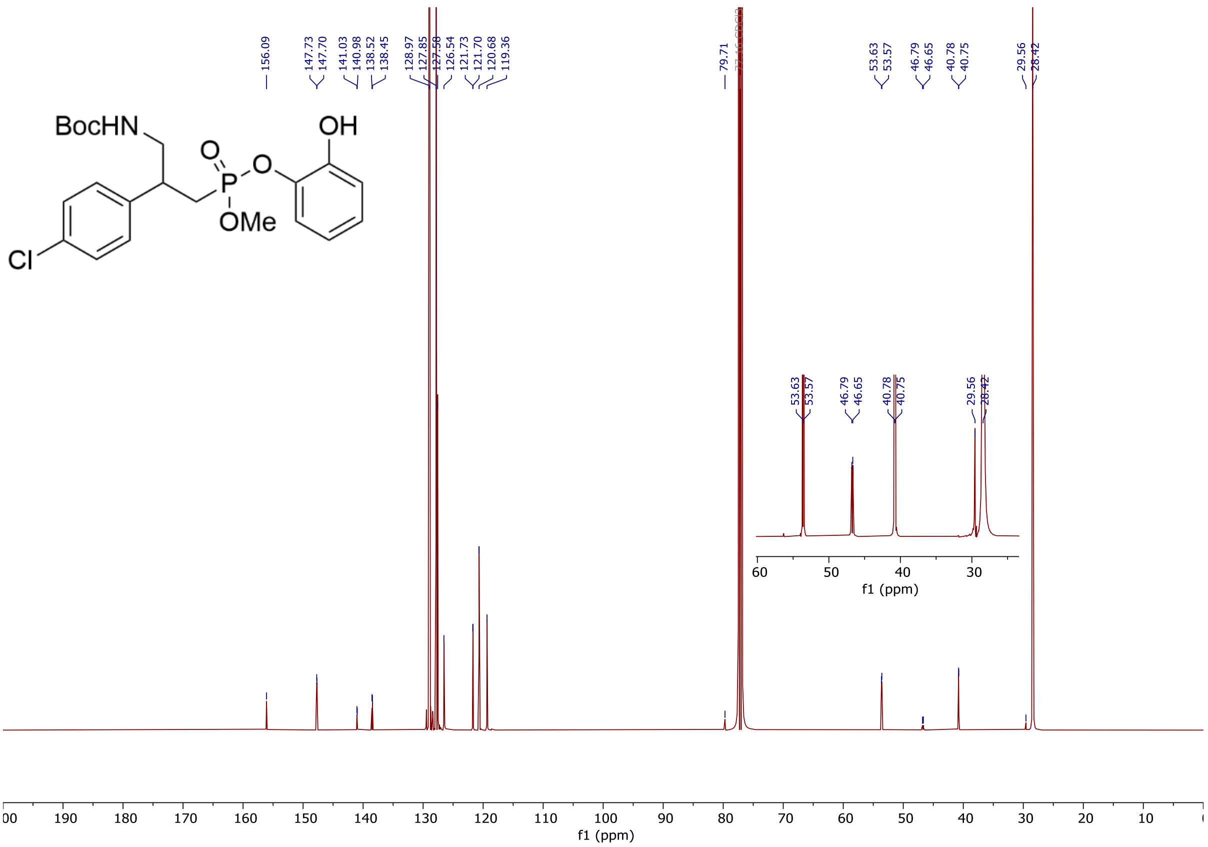

^{31}P NMR (162 MHz, $CDCl_3$): **3ah**

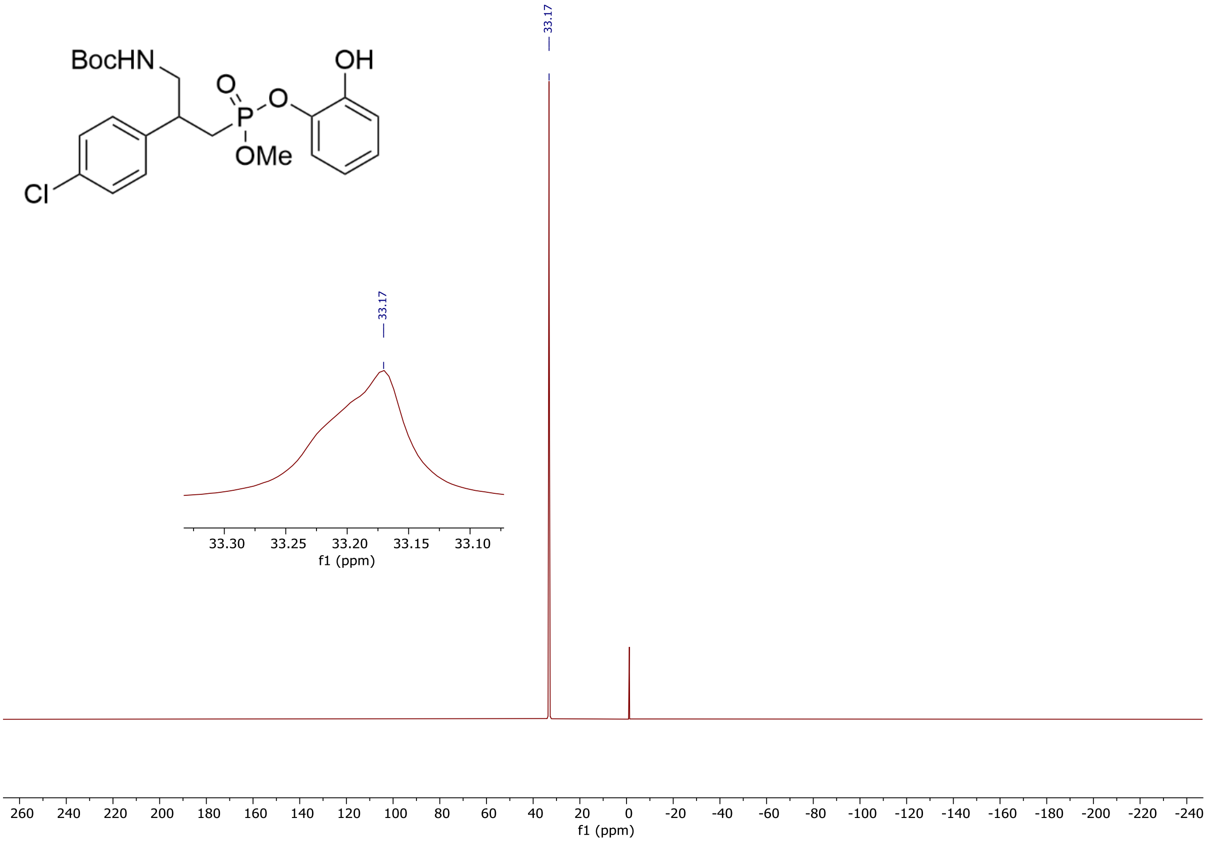

^{1}H NMR (400 MHz, $CDCl_3$): **3ai**

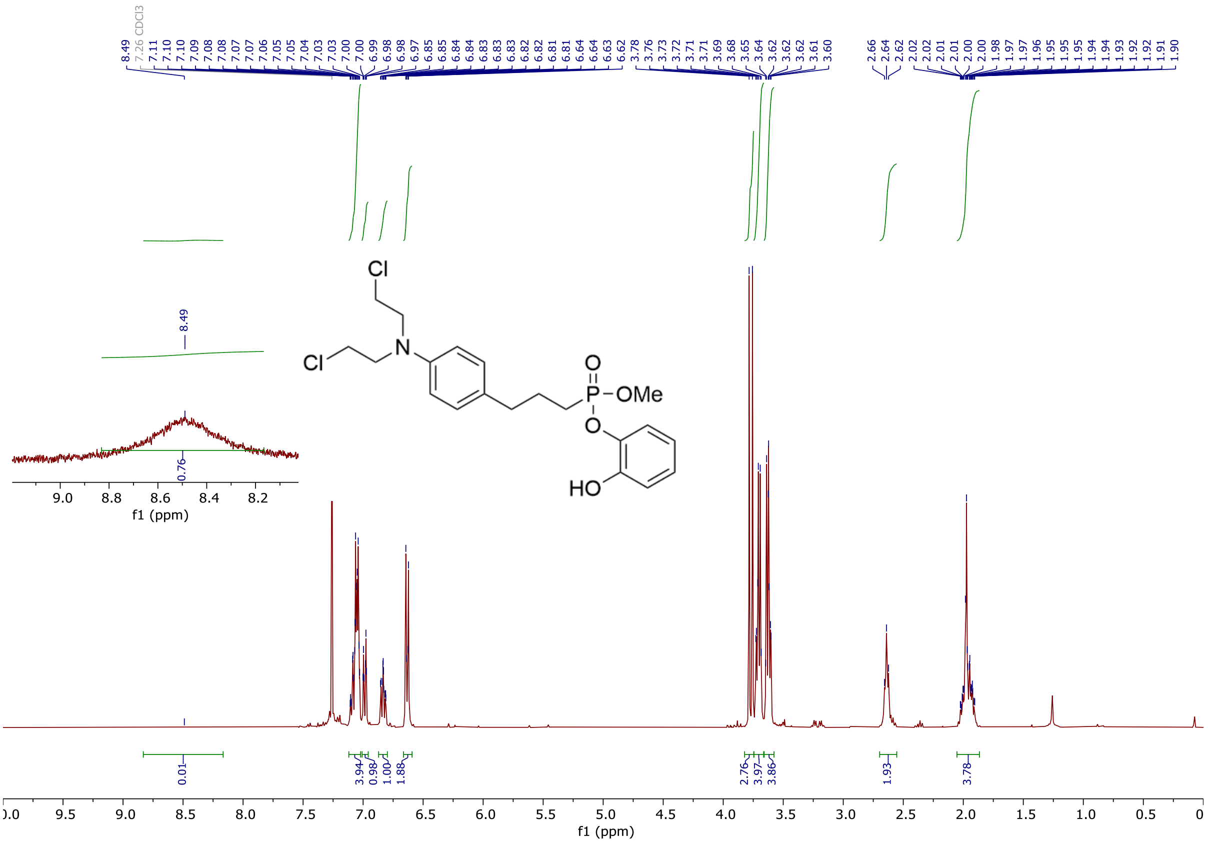

^{13}C NMR (101 MHz, $CDCl_3$): **3ai**

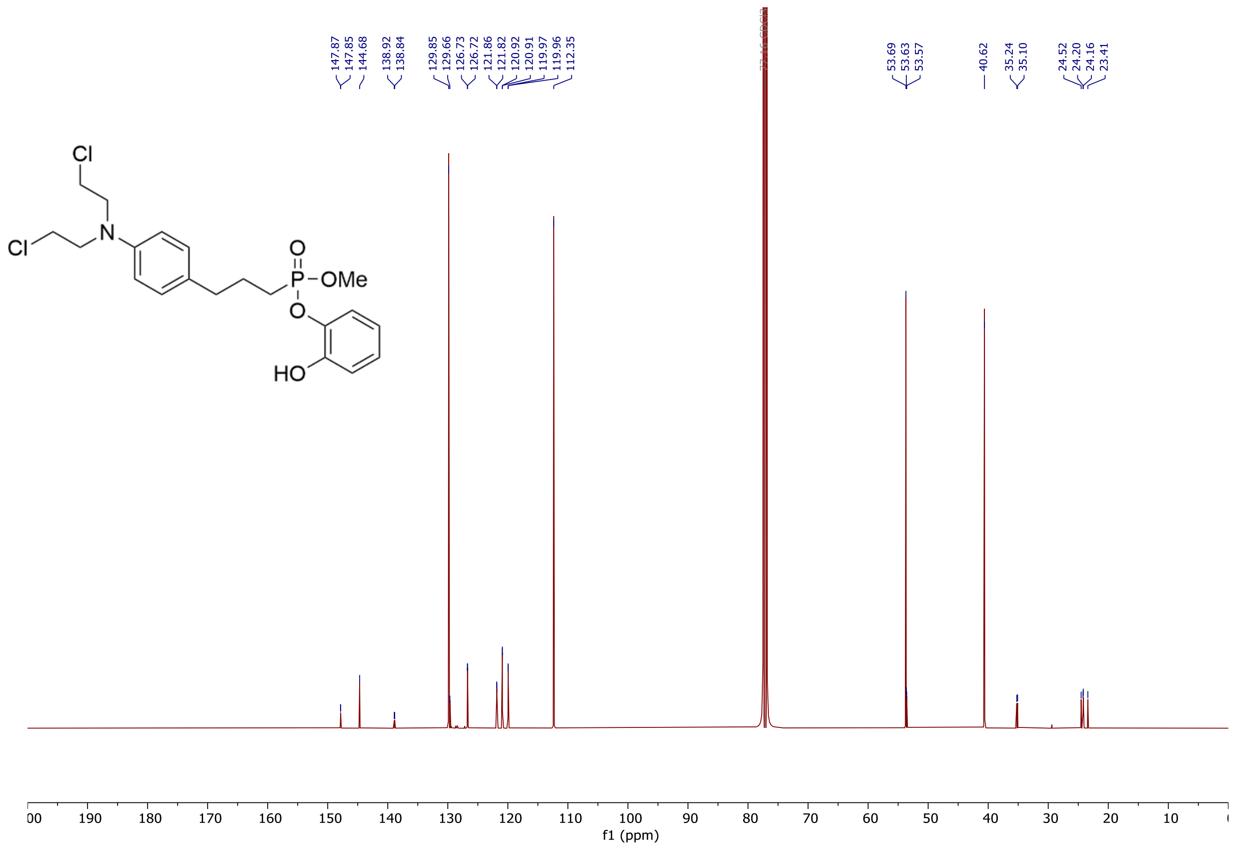

^{31}P NMR (162 MHz, $CDCl_3$): **3ai**

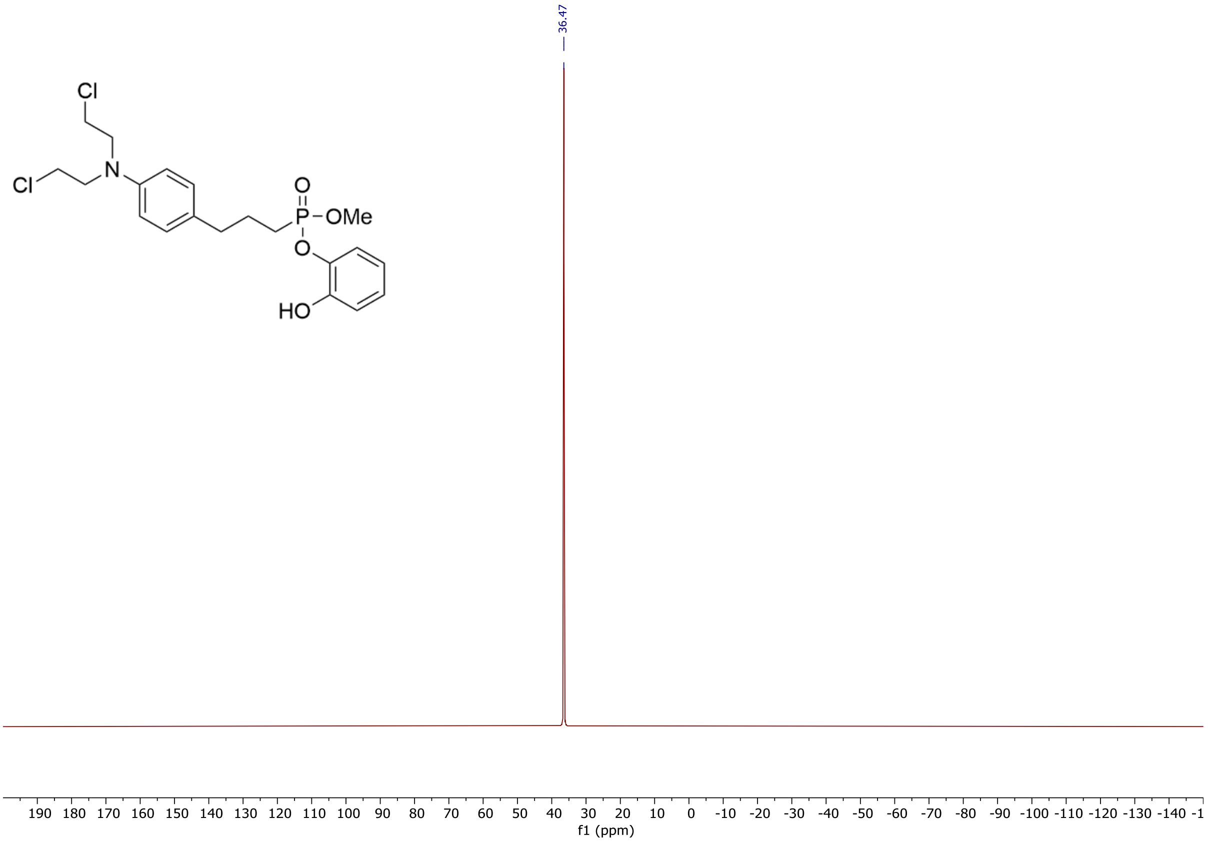

^{1}H NMR (400 MHz, $CDCl_3$): **3aj**

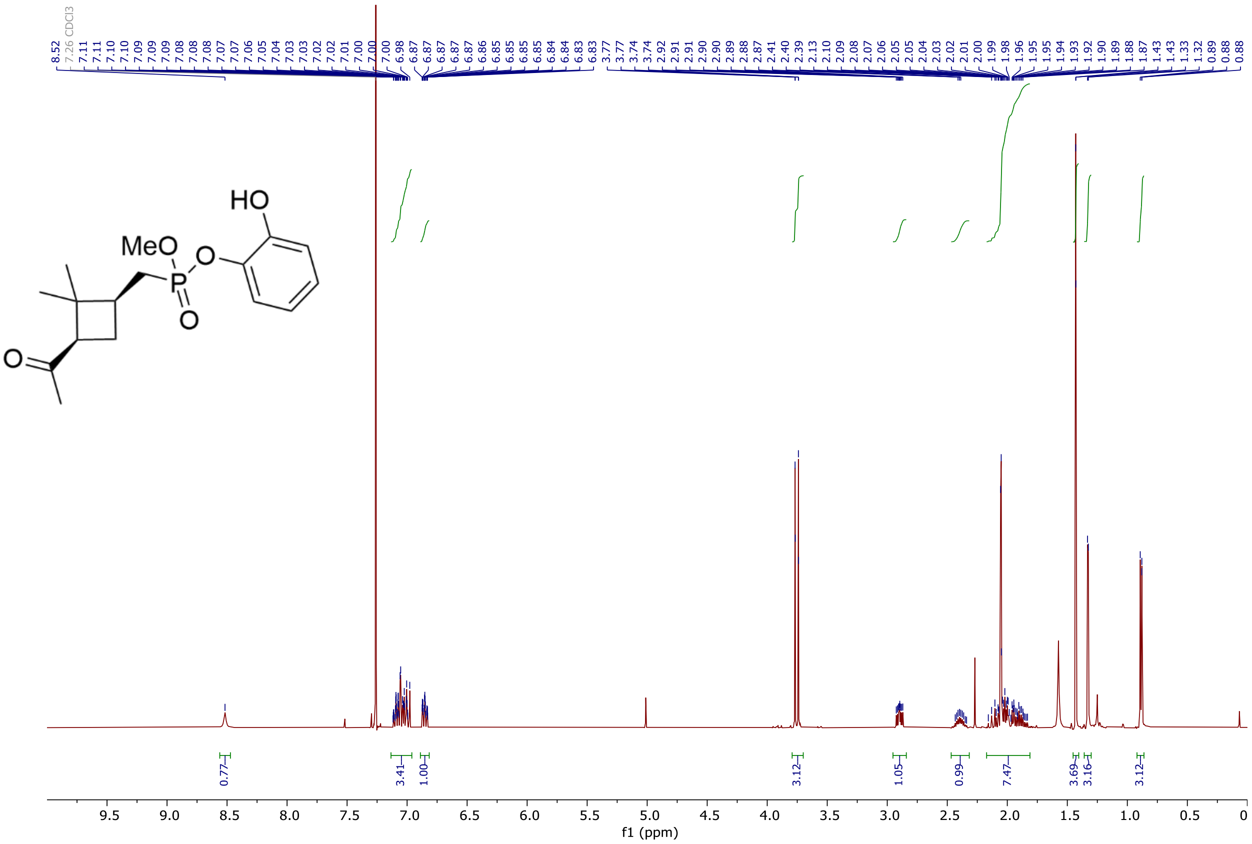

^{13}C NMR (126 MHz, $CDCl_3$): **3aj**

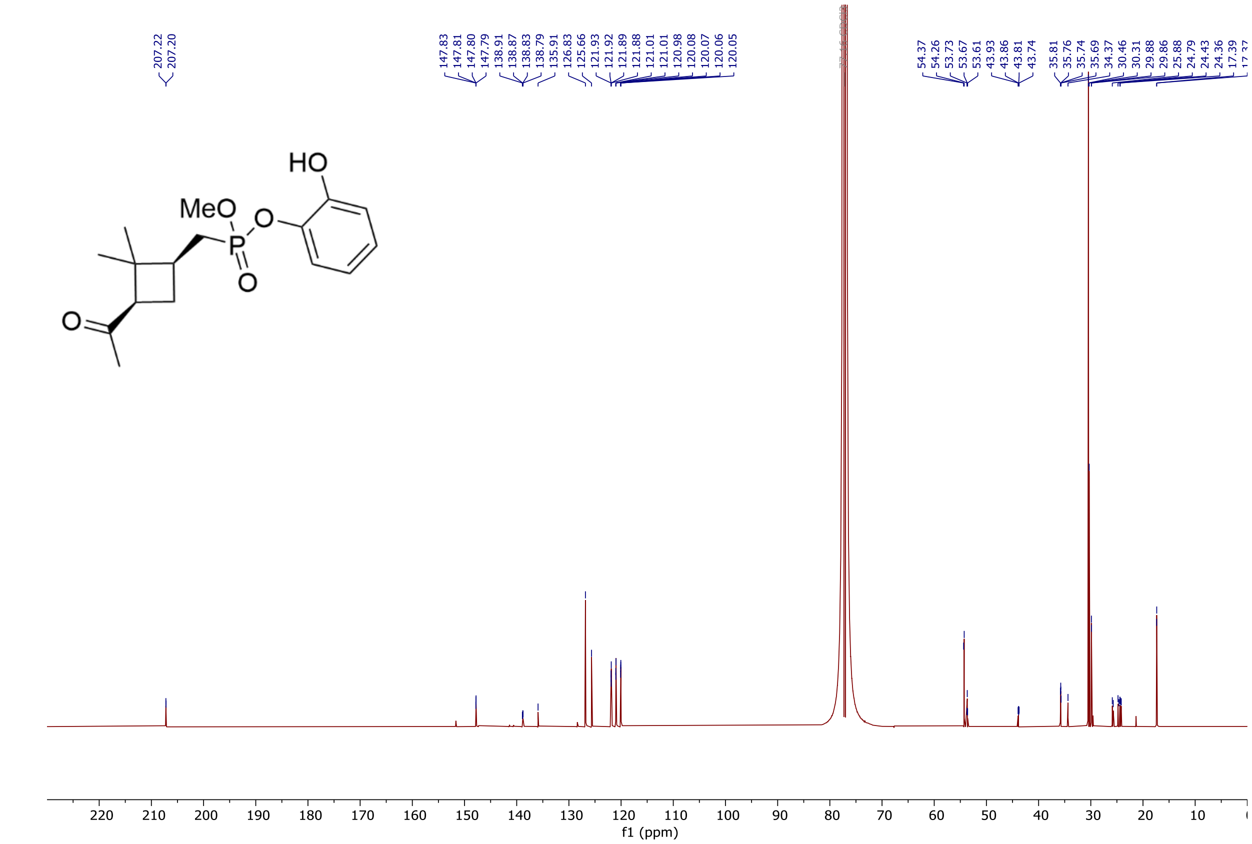

^{31}P NMR (162 MHz, $CDCl_3$): **3aj**

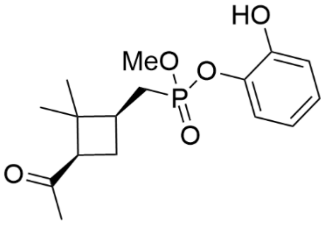

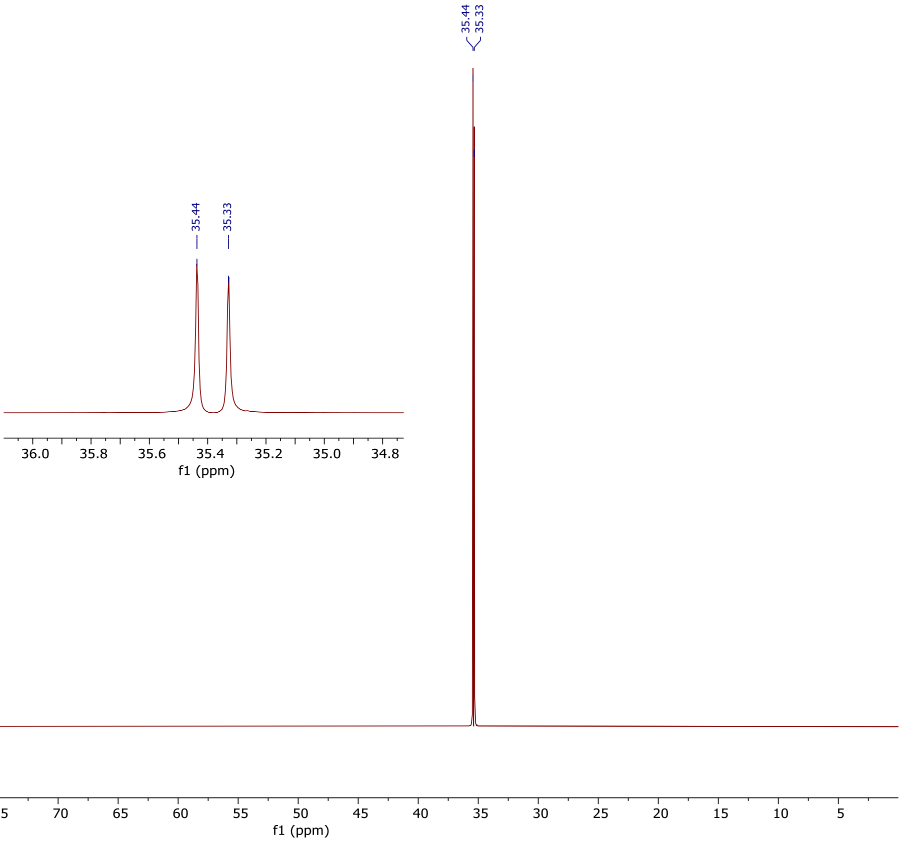

^{1}H NMR (400 MHz, $CDCl_3$): **3ak**

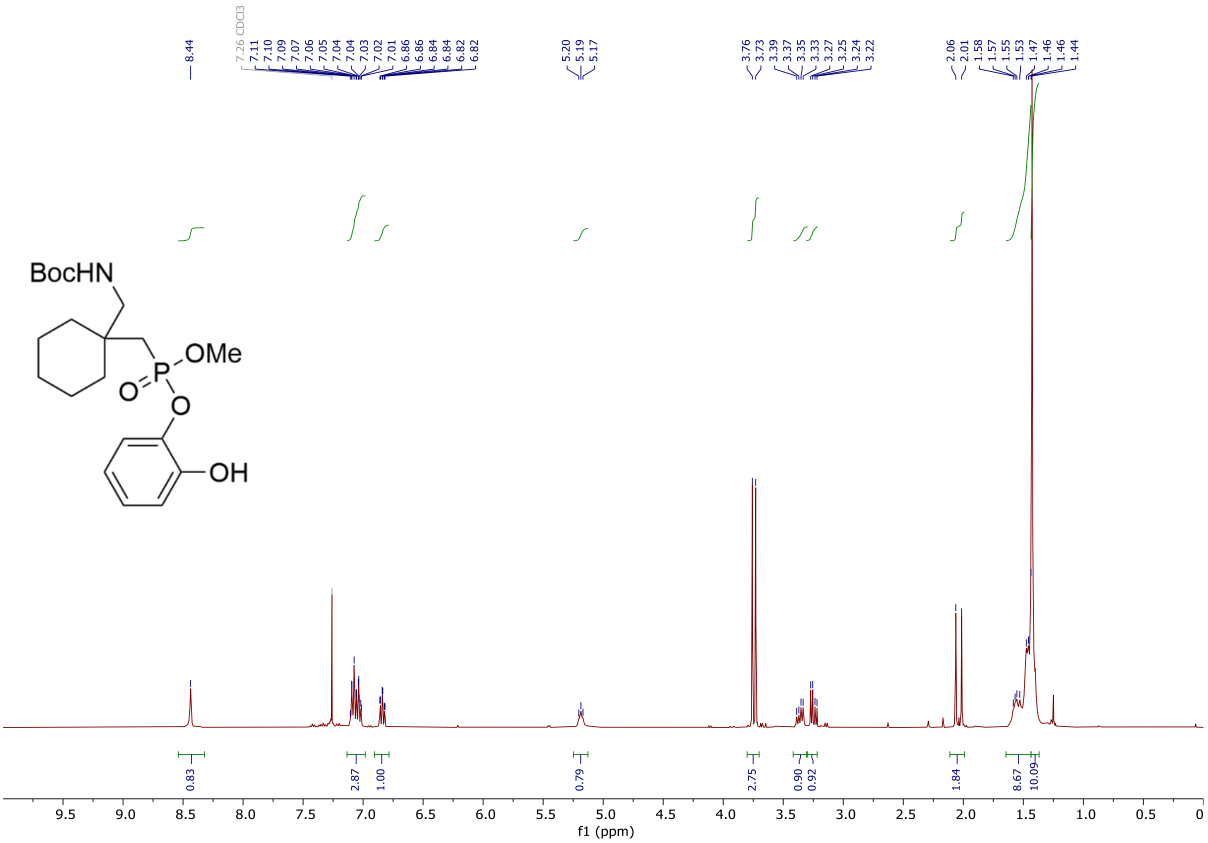

^{13}C NMR (101 MHz, $CDCl_3$): **3ak**

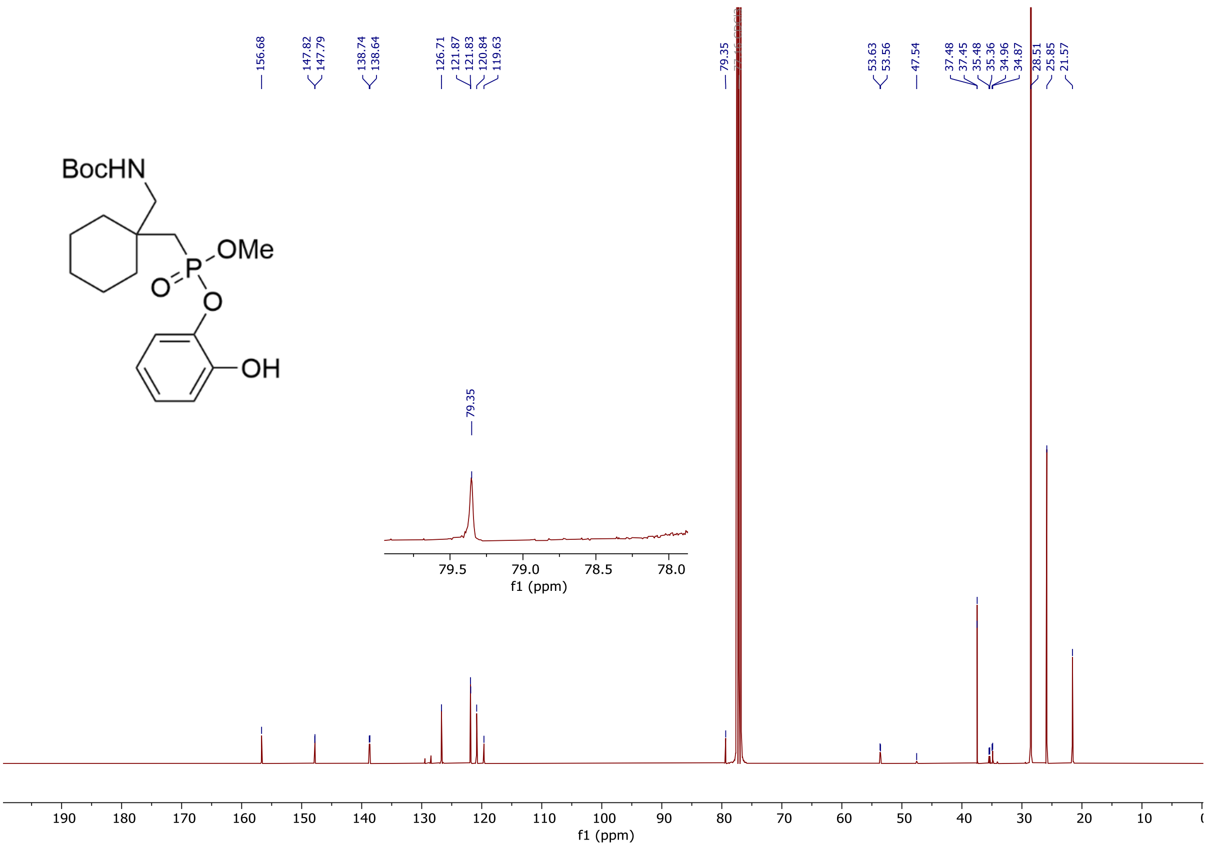

^{31}**P NMR** (162 MHz, $CDCl_3$): **3ak**

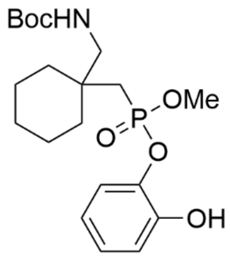

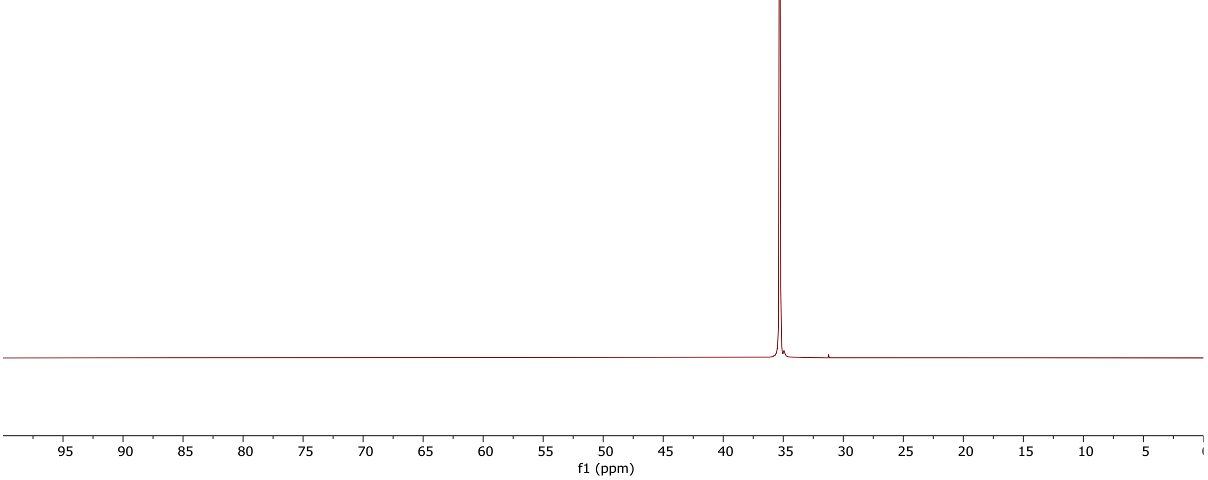

^{1}H NMR (400 MHz, $CDCl_3$): **3al**

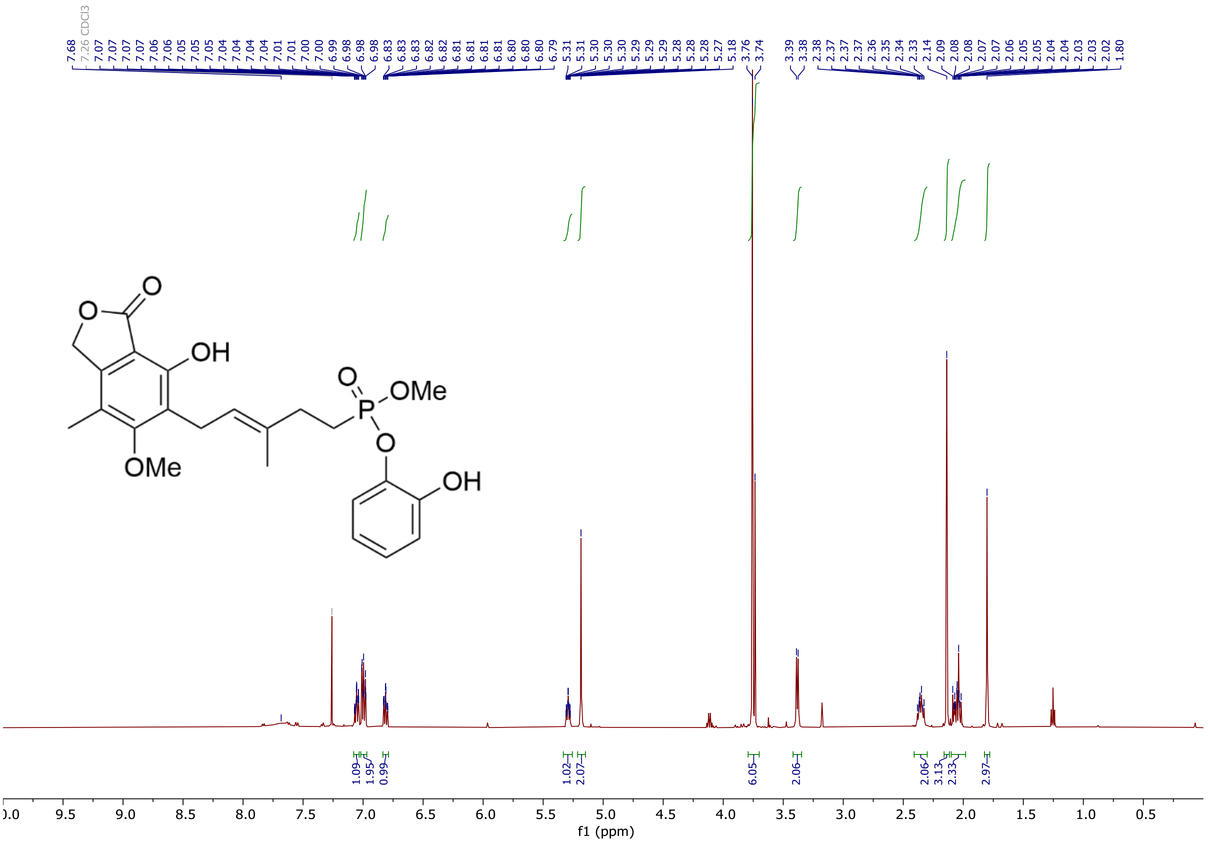

^{13}C NMR (101 MHz, $CDCl_3$): **3al**

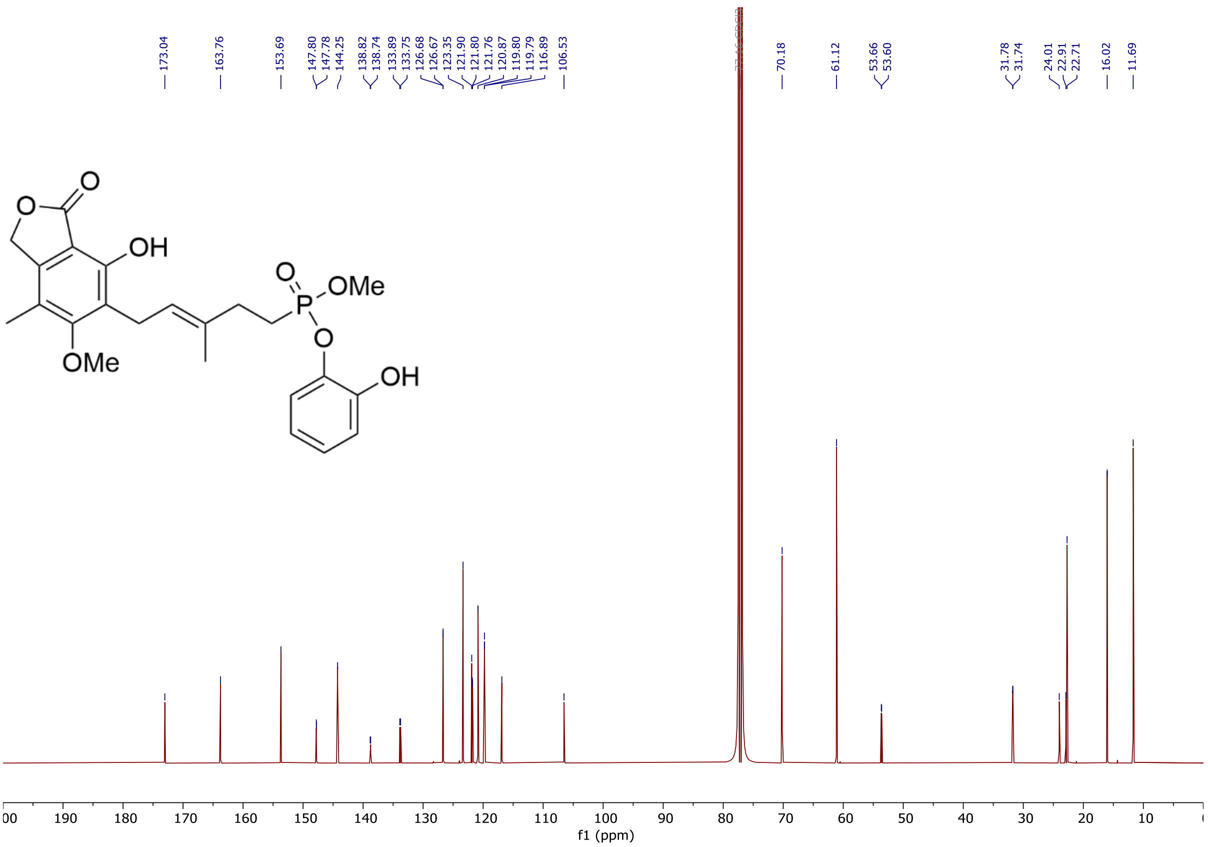

^{31}P NMR (162 MHz, $CDCl_3$): **3al**

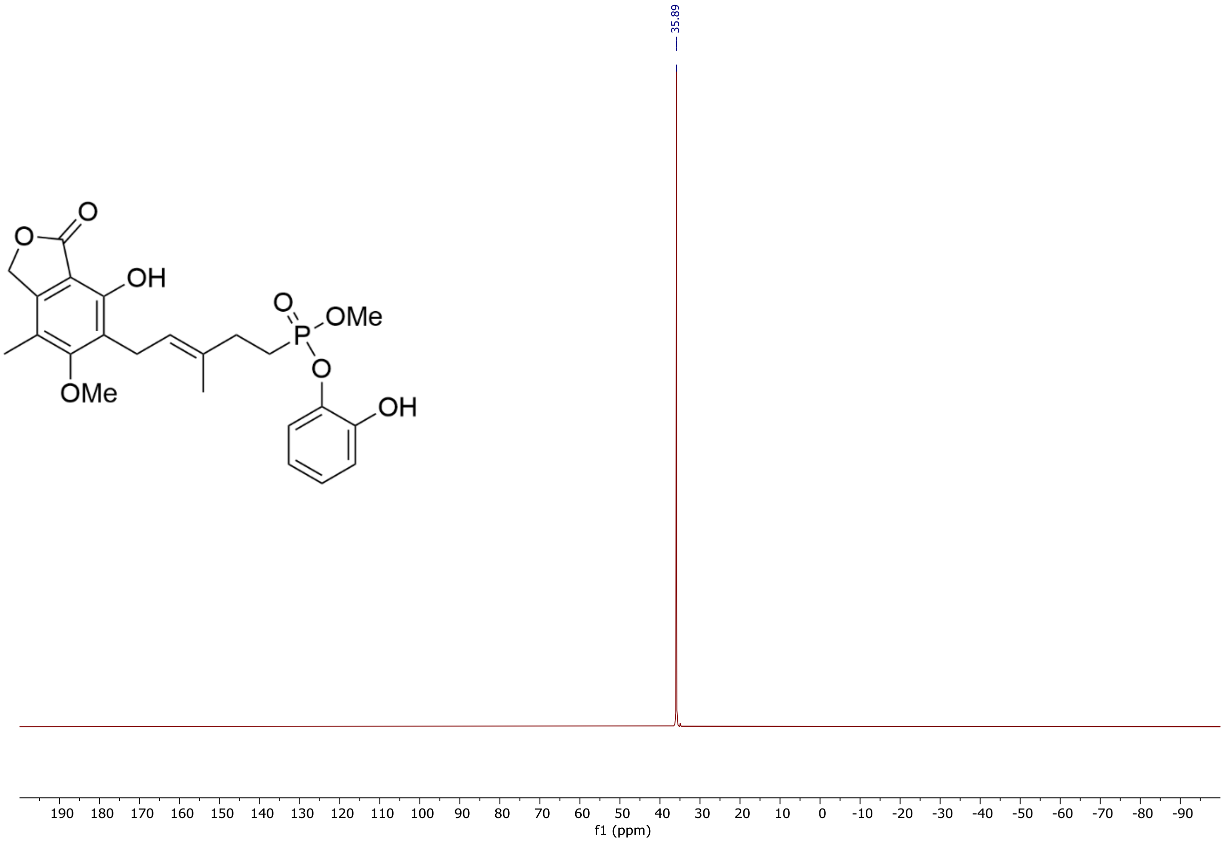

^{1}H NMR (400 MHz, $CDCl_3$): **3am**

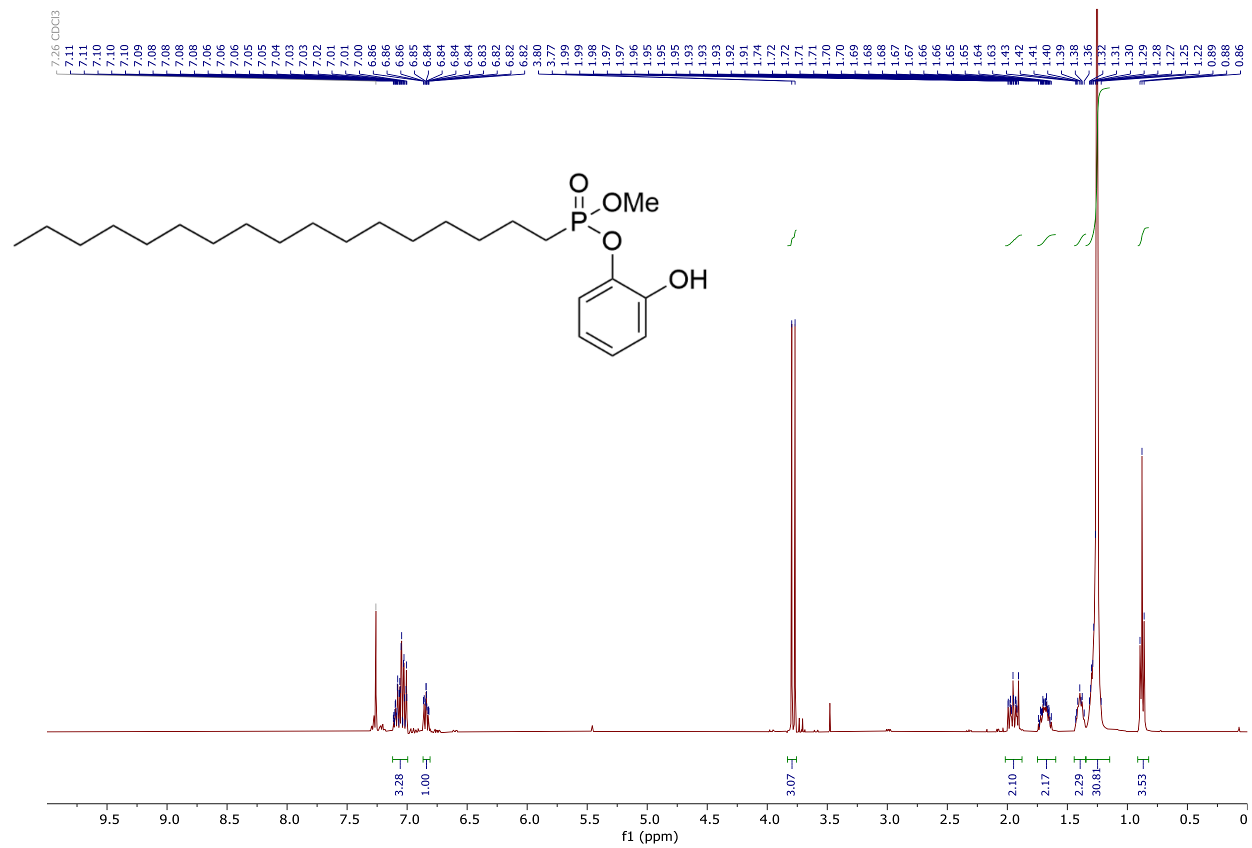

^{13}C NMR (101 MHz, $CDCl_3$): **3am**

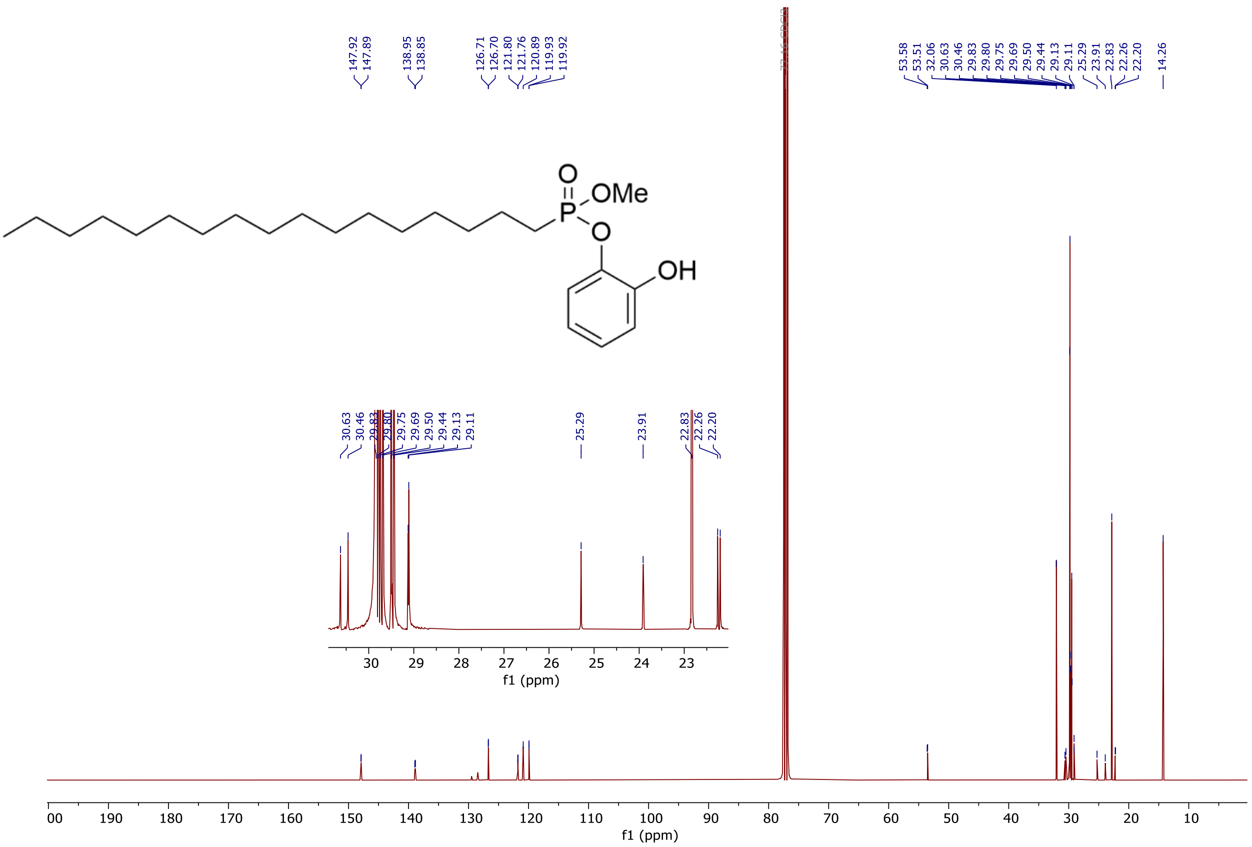

^{31}P NMR (162 MHz, $CDCl_3$): **3am**

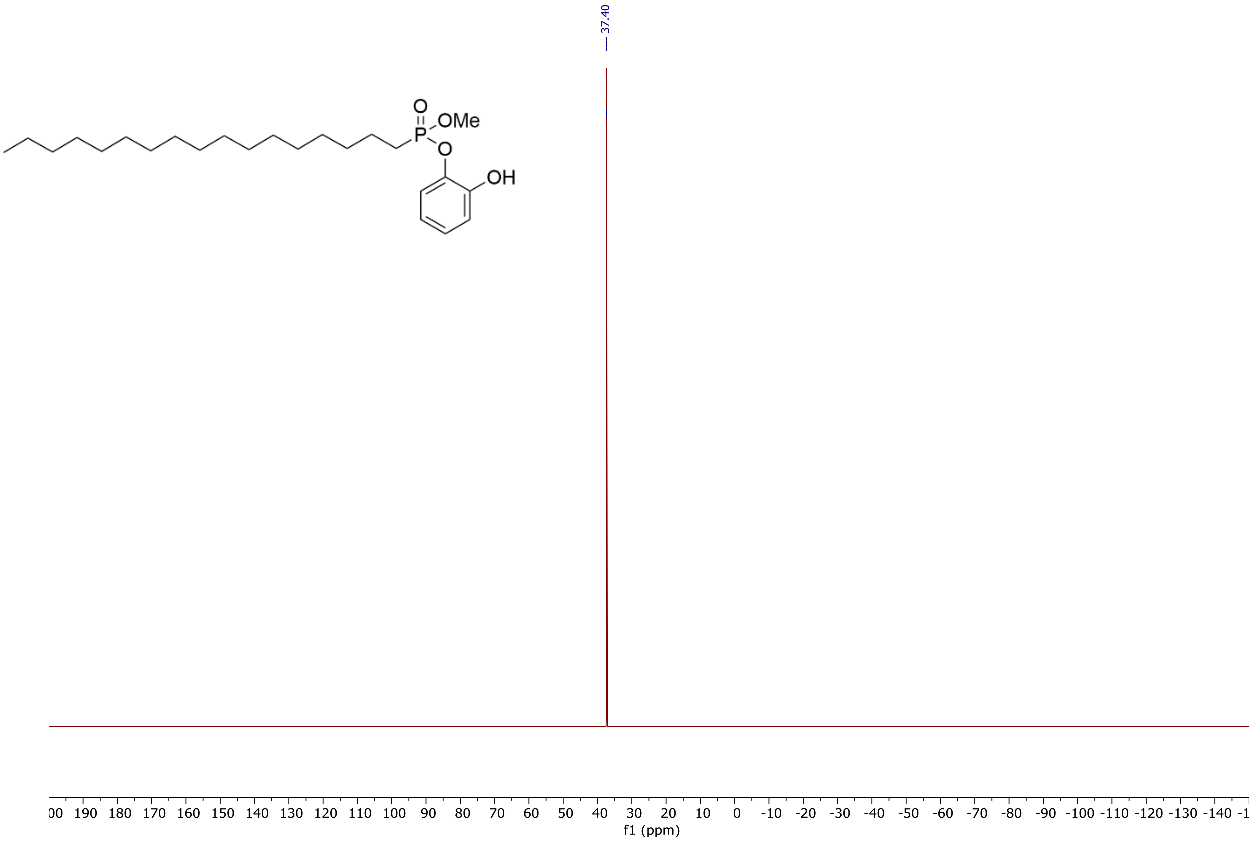

^{1}H NMR (600 MHz, $CDCl_3$): **3an**

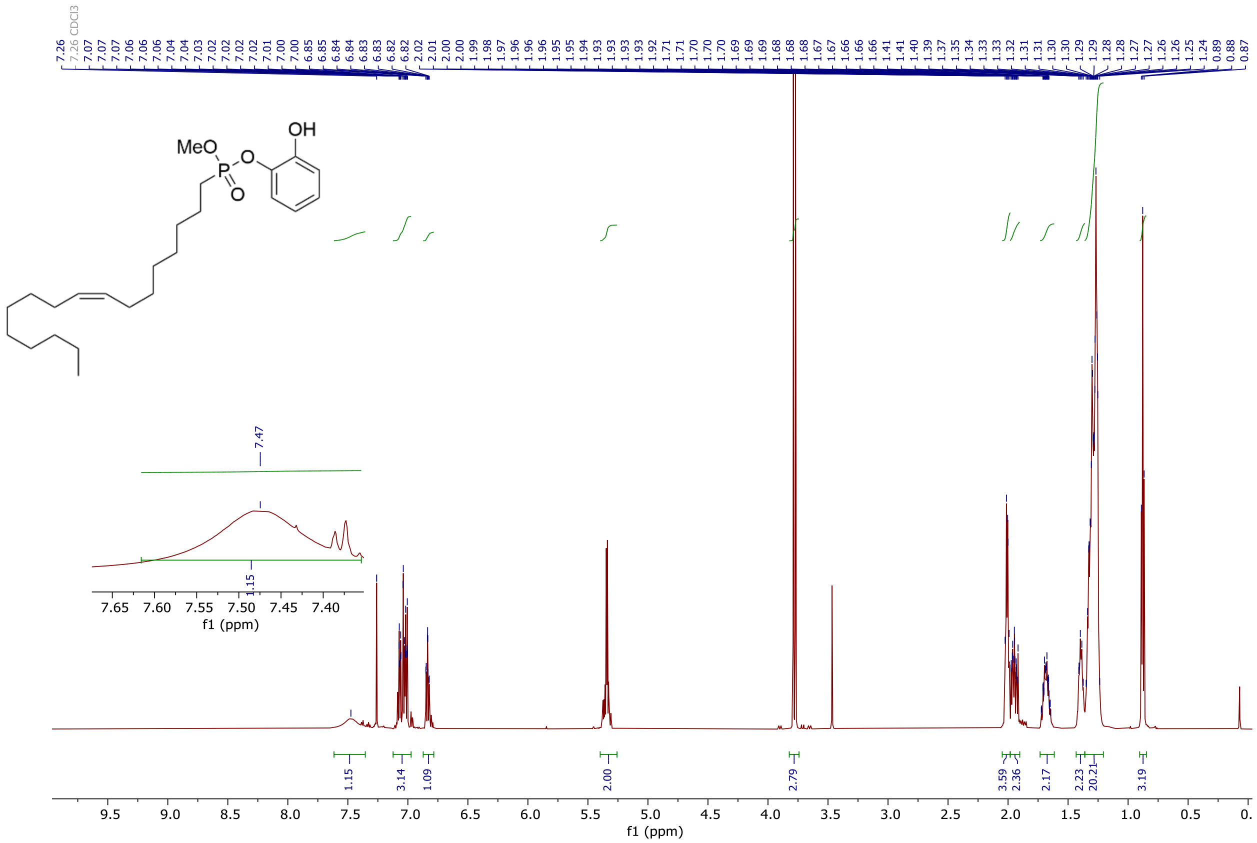

^{13}C NMR (151 MHz, $CDCl_3$): **3an**

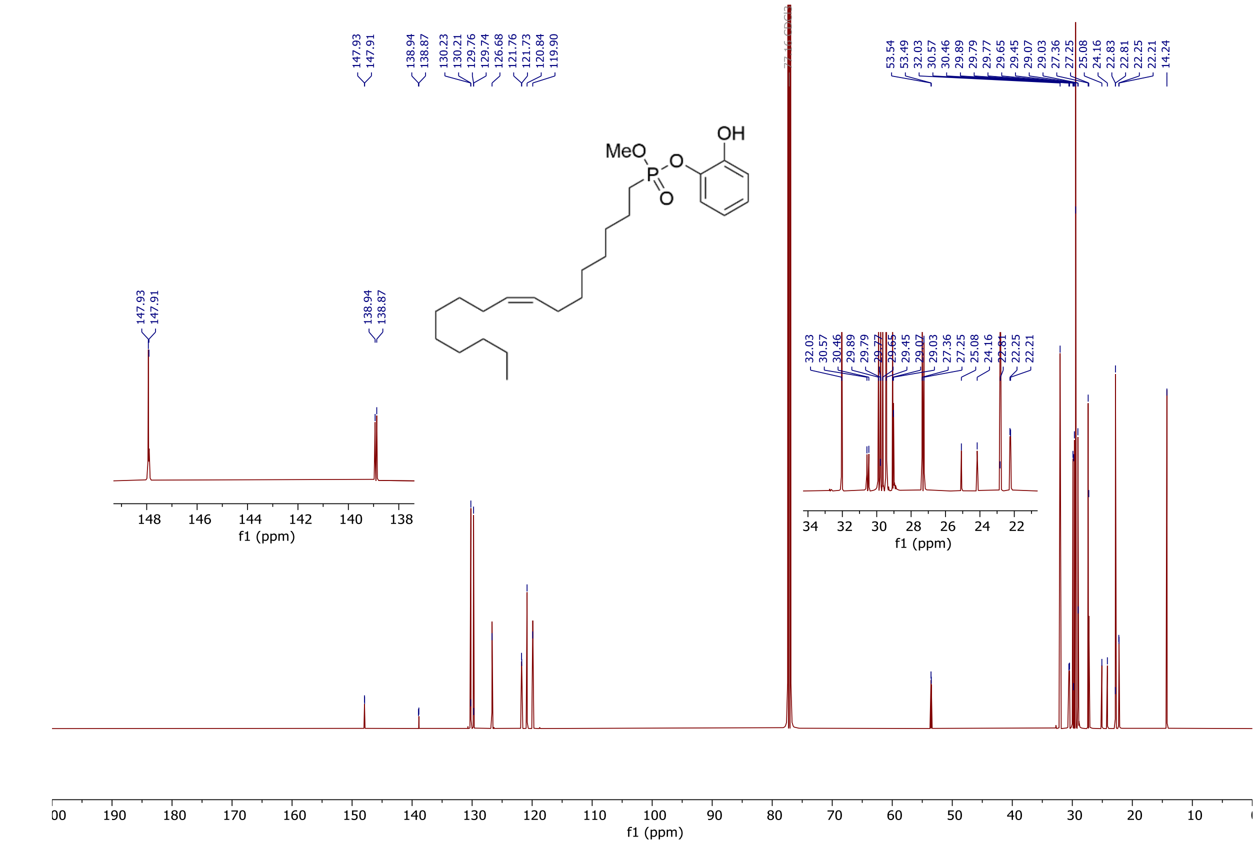

^{31}**P NMR** (162 MHz, $CDCl_3$): **3an**

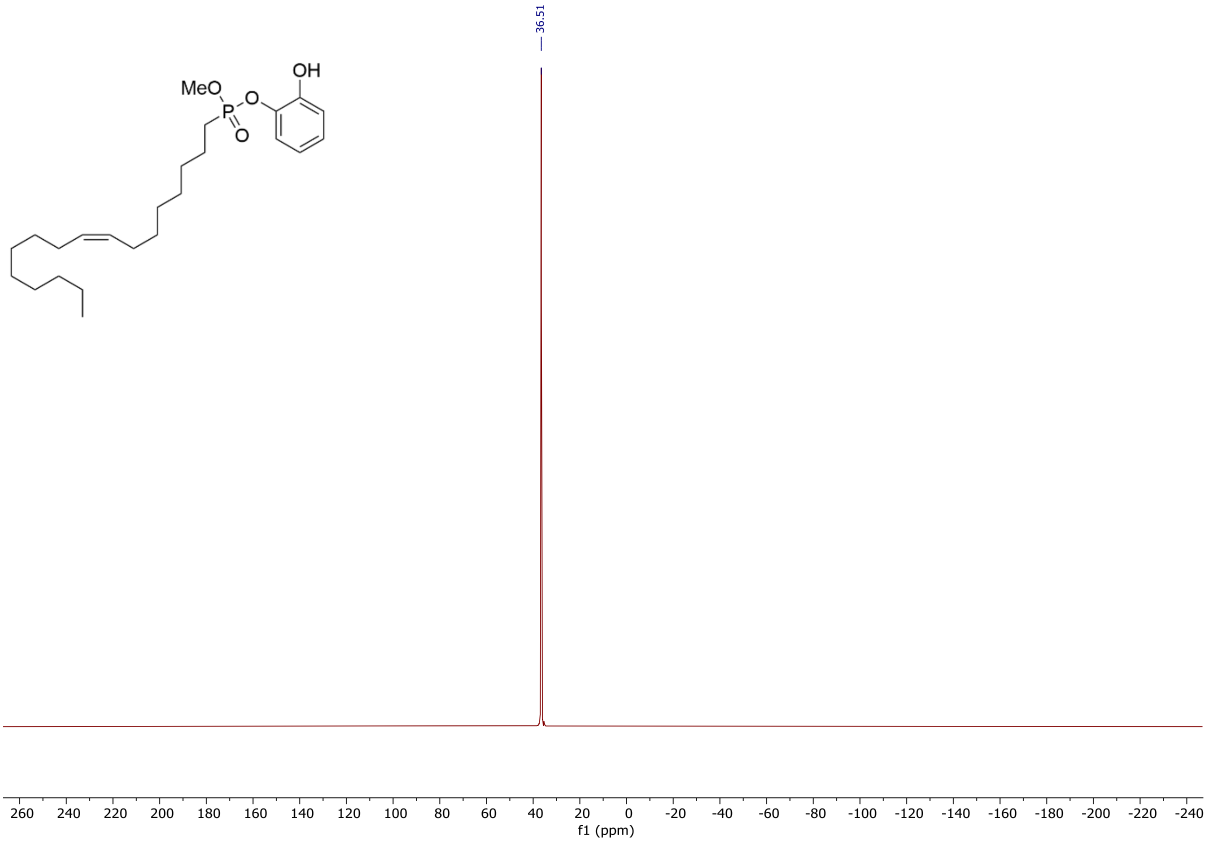

^{1}H NMR (600 MHz, CDCl3): **3ao**

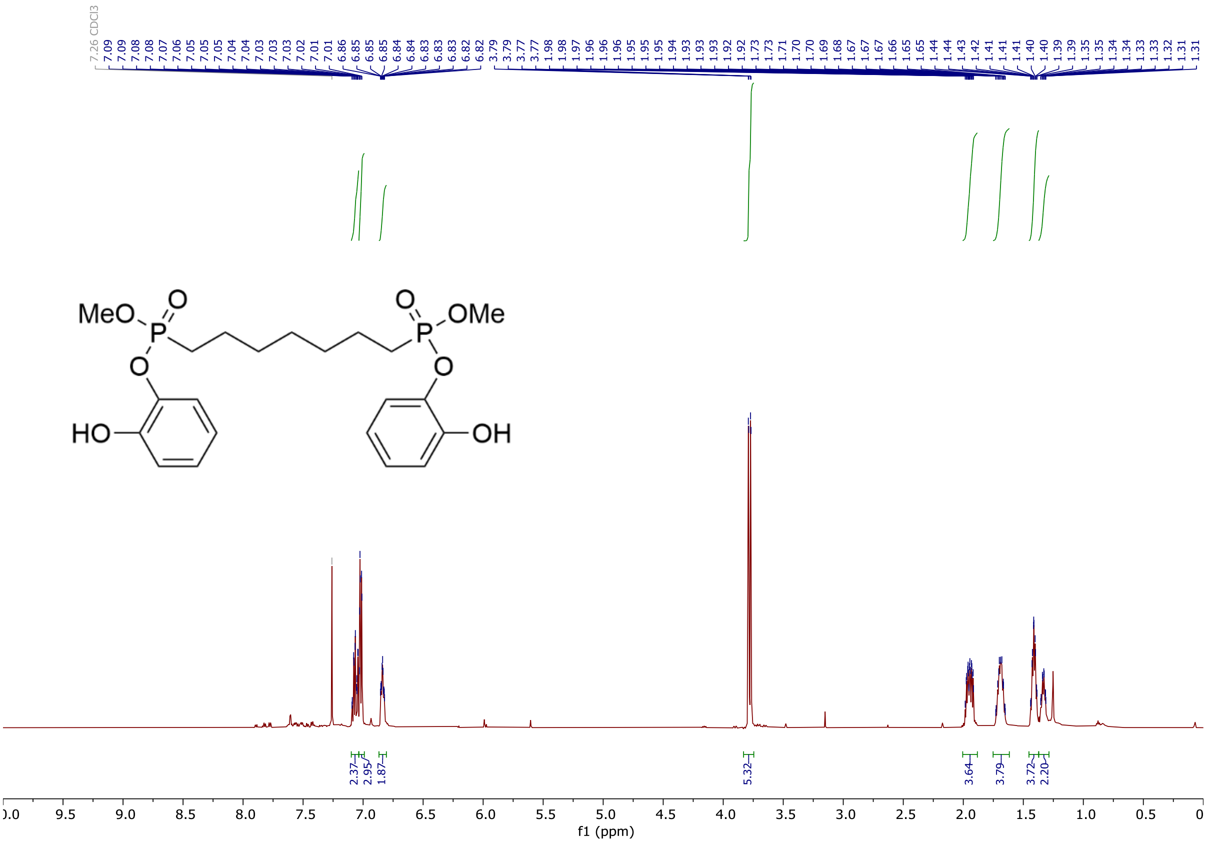

^{13}C NMR (151 MHz, CDCl3): **3ao**

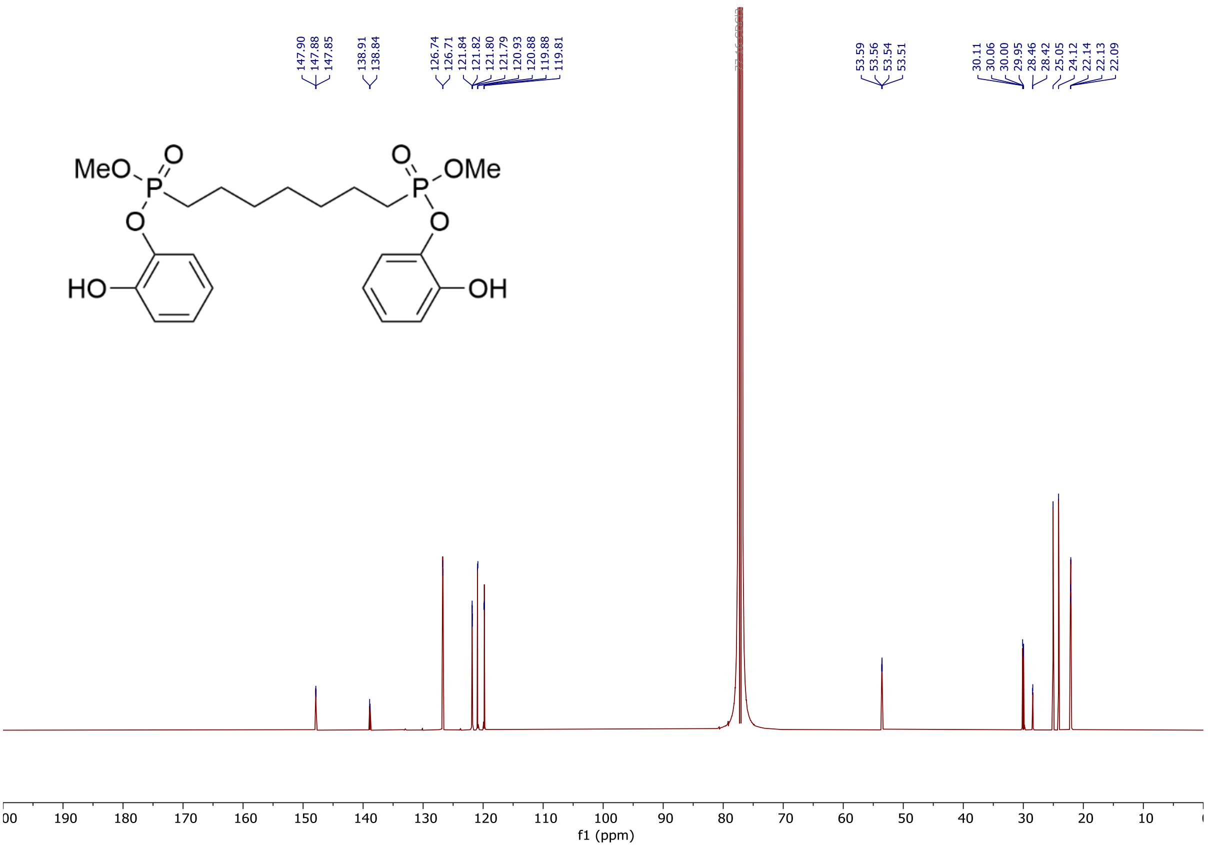

^{31}P NMR (162 MHz, $CDCl_3$): **3ao**

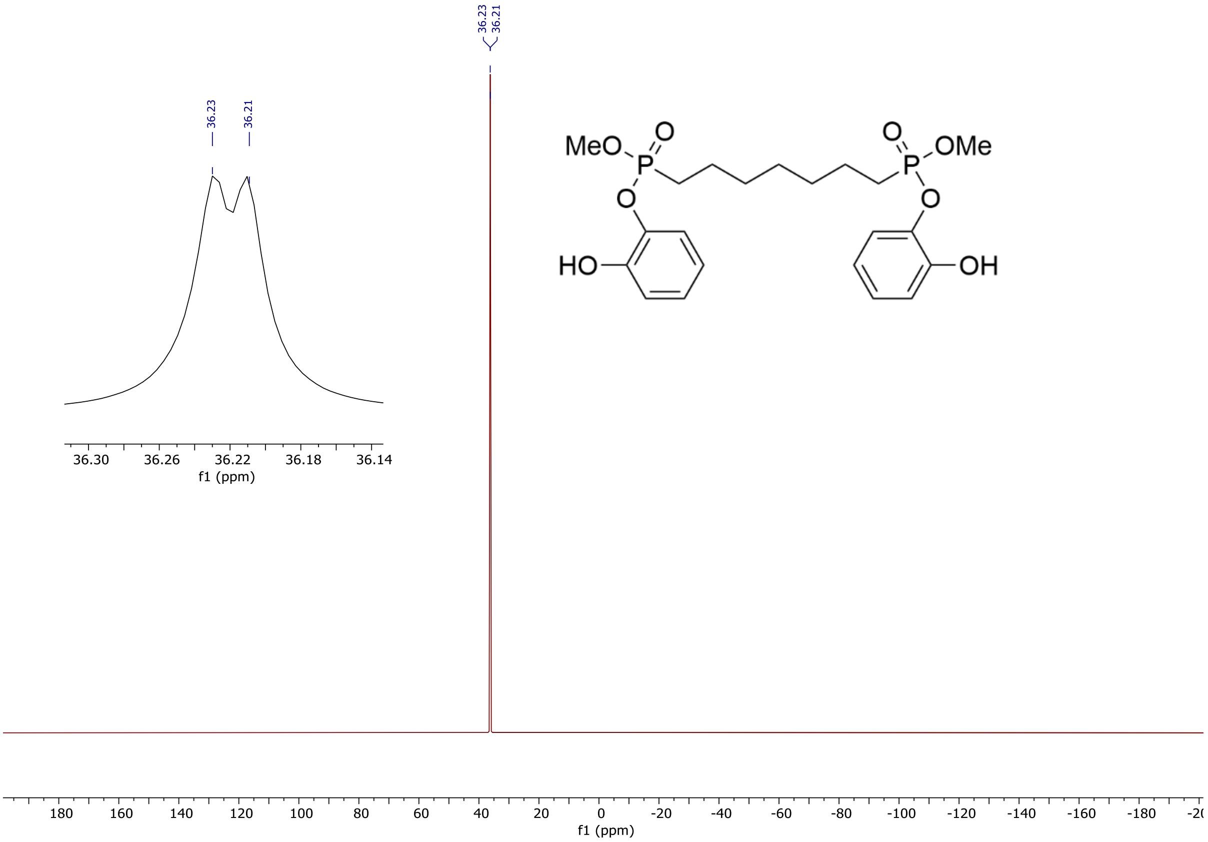

^{1}H NMR (400 MHz, $CDCl_3$): **3ap**

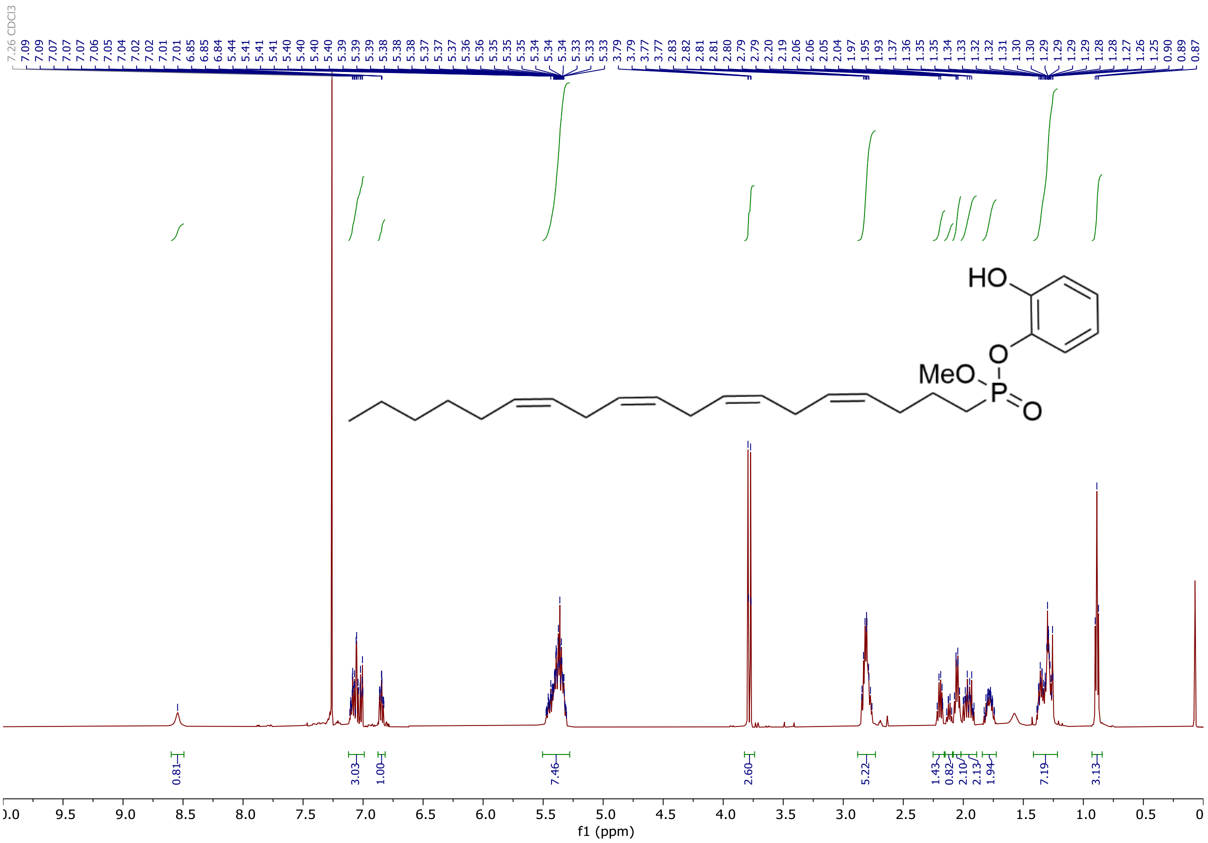

^{13}C NMR (101 MHz, $CDCl_3$): **3ap**

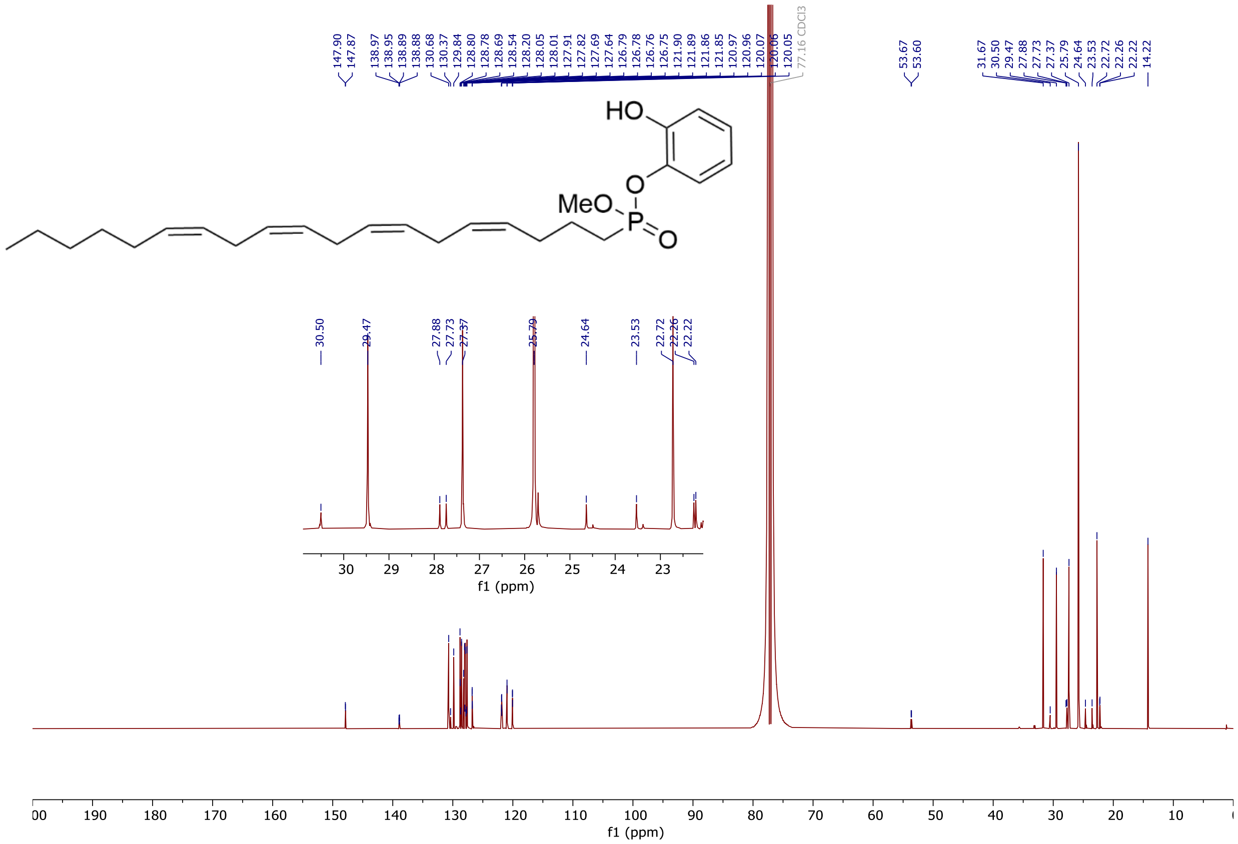

^{31}P NMR (162 MHz, $CDCl_3$): **3ap**

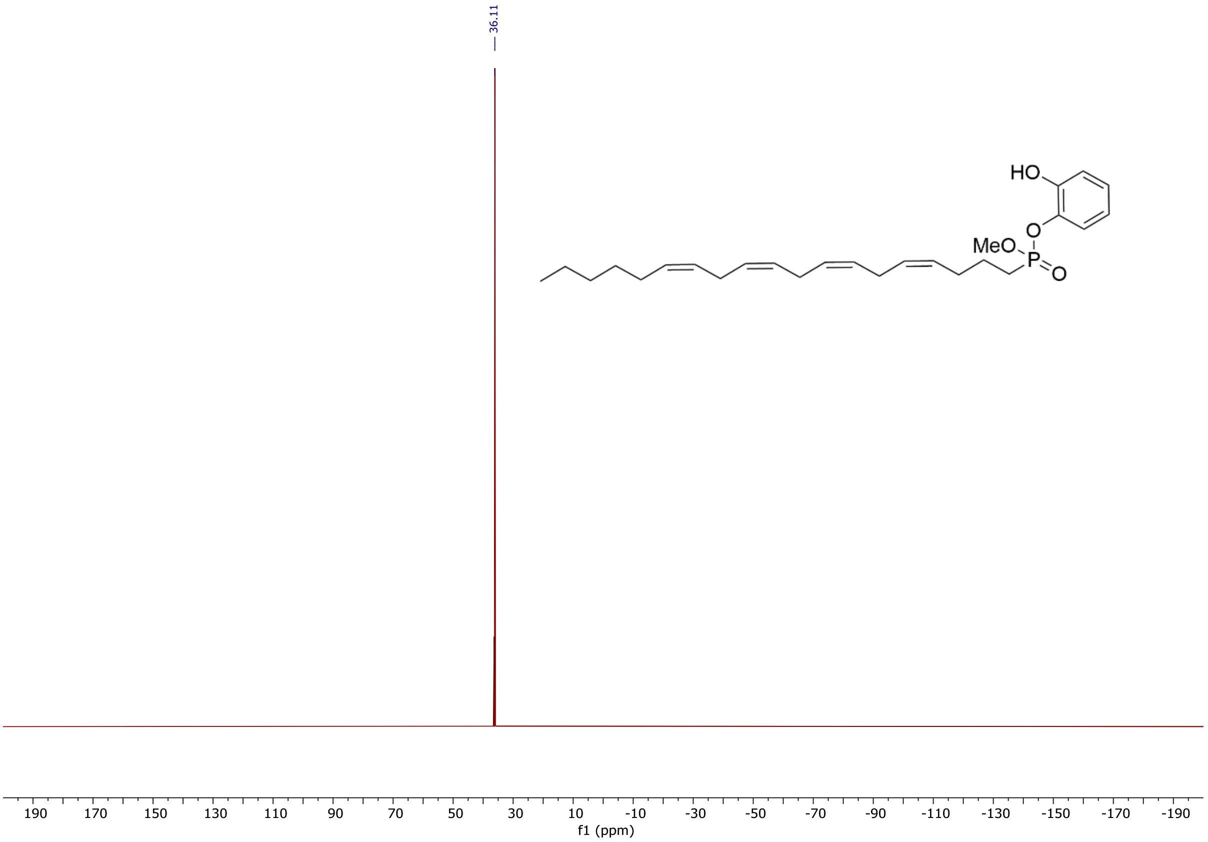

^{1}H NMR (400 MHz, $CDCl_3$): **3aq**

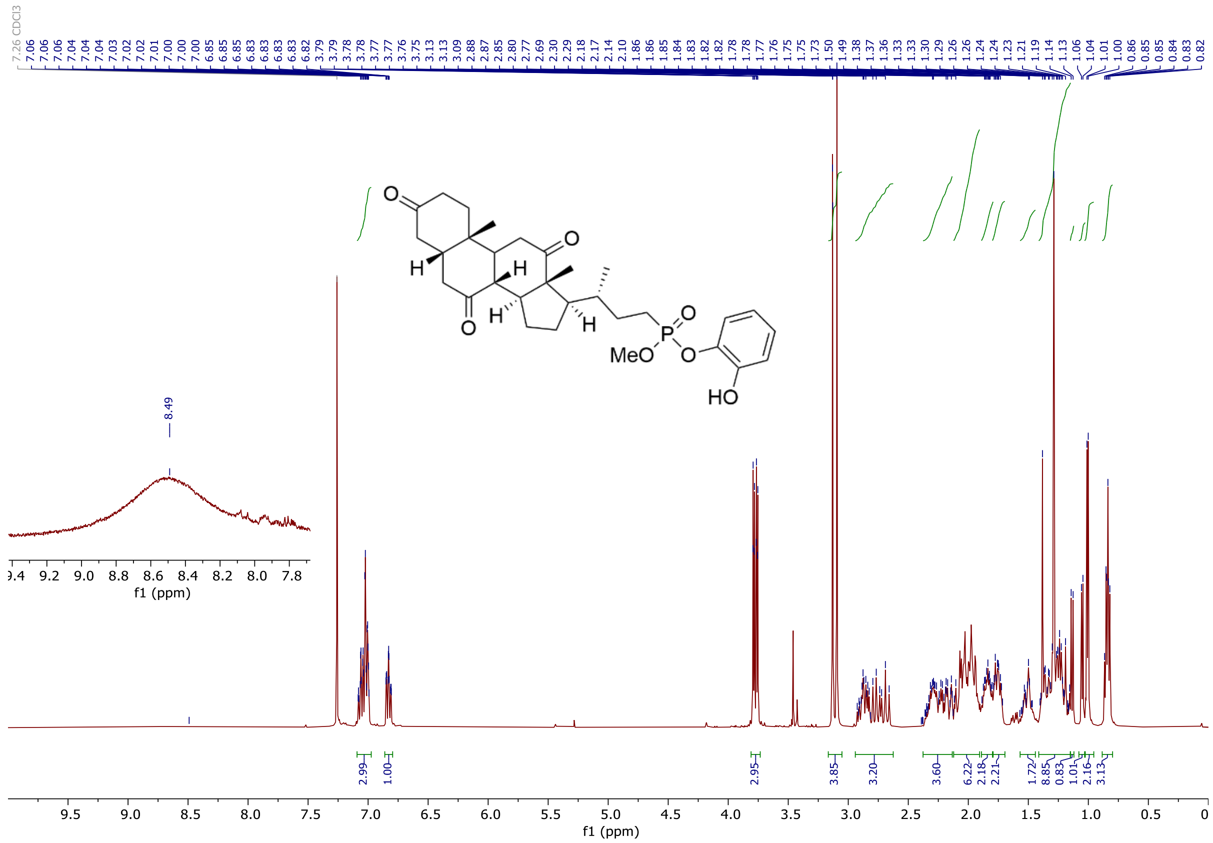

^{13}C NMR (101 MHz, $CDCl_3$): **3aq**

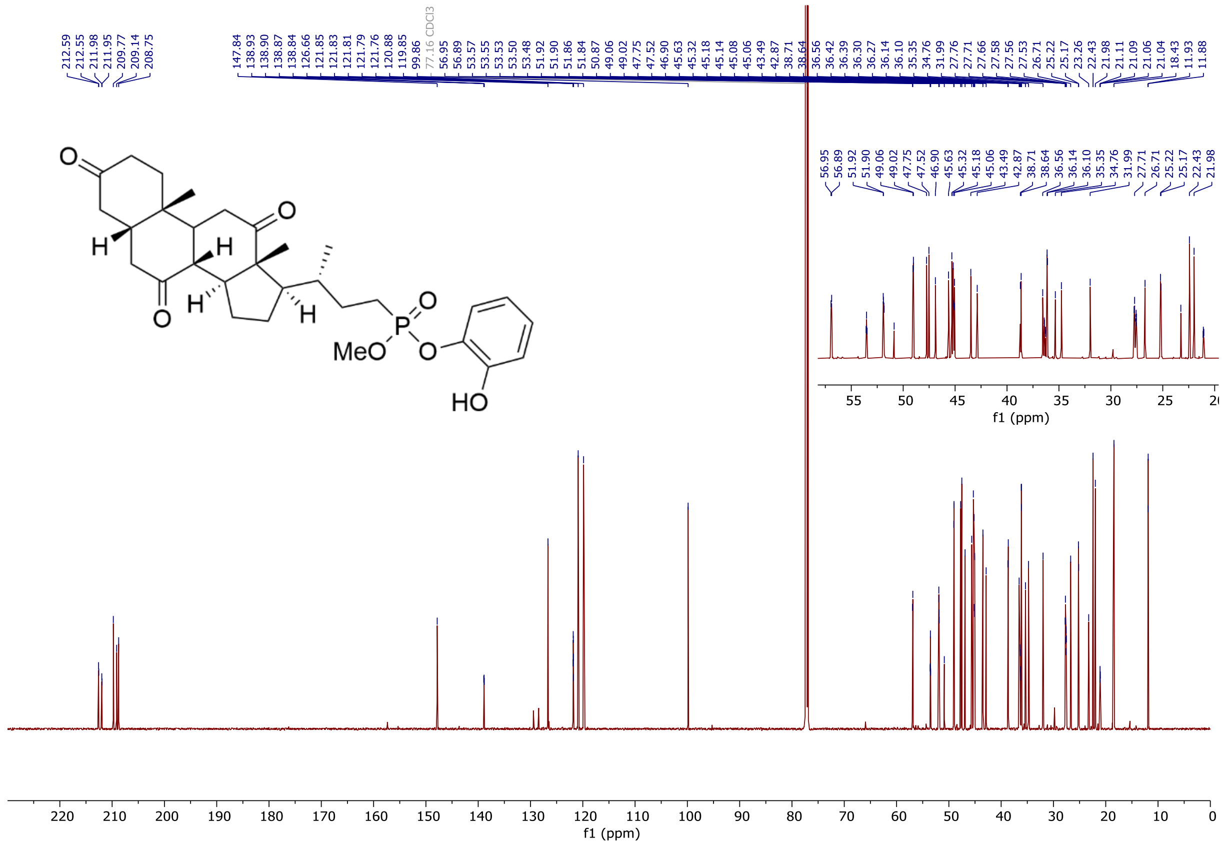

^{31}P NMR (162 MHz, $CDCl_3$): **3aq**

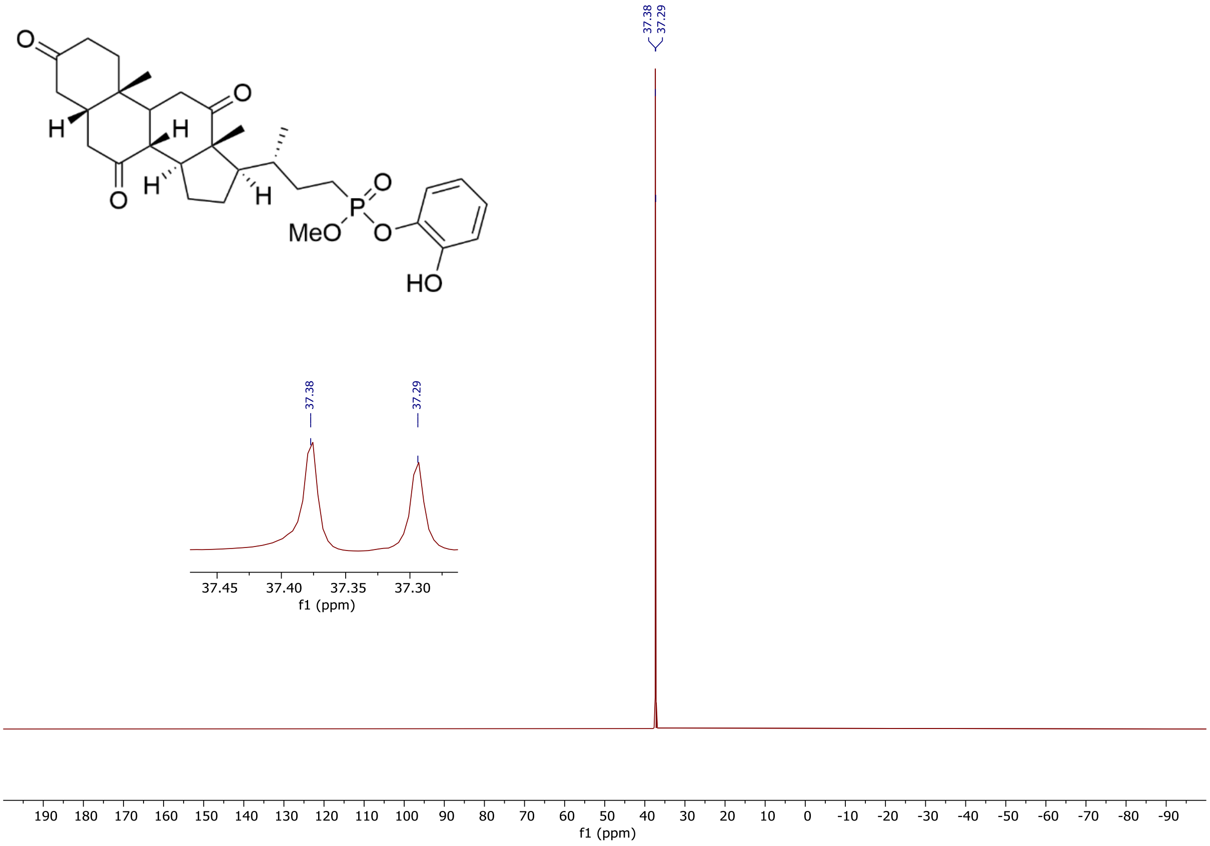

¹H NMR (400 MHz, $CDCl_3$): **3ar**

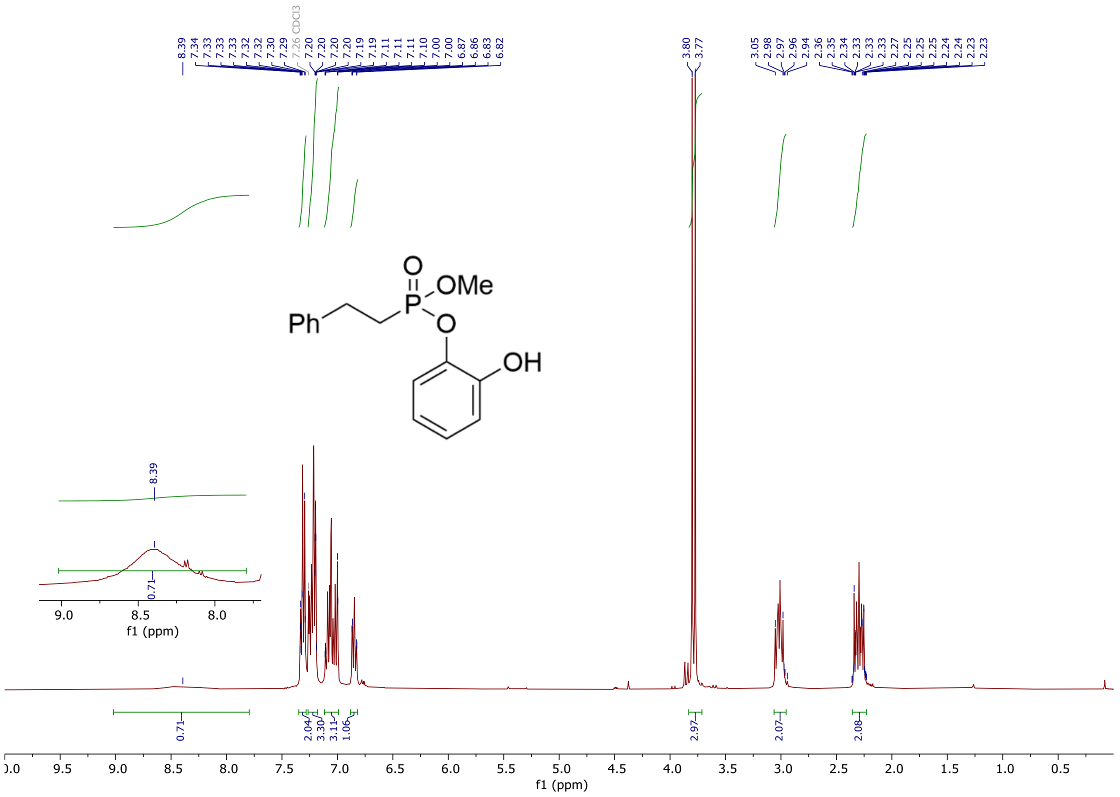

¹³C NMR (101 MHz, $CDCl_3$): **3ar**

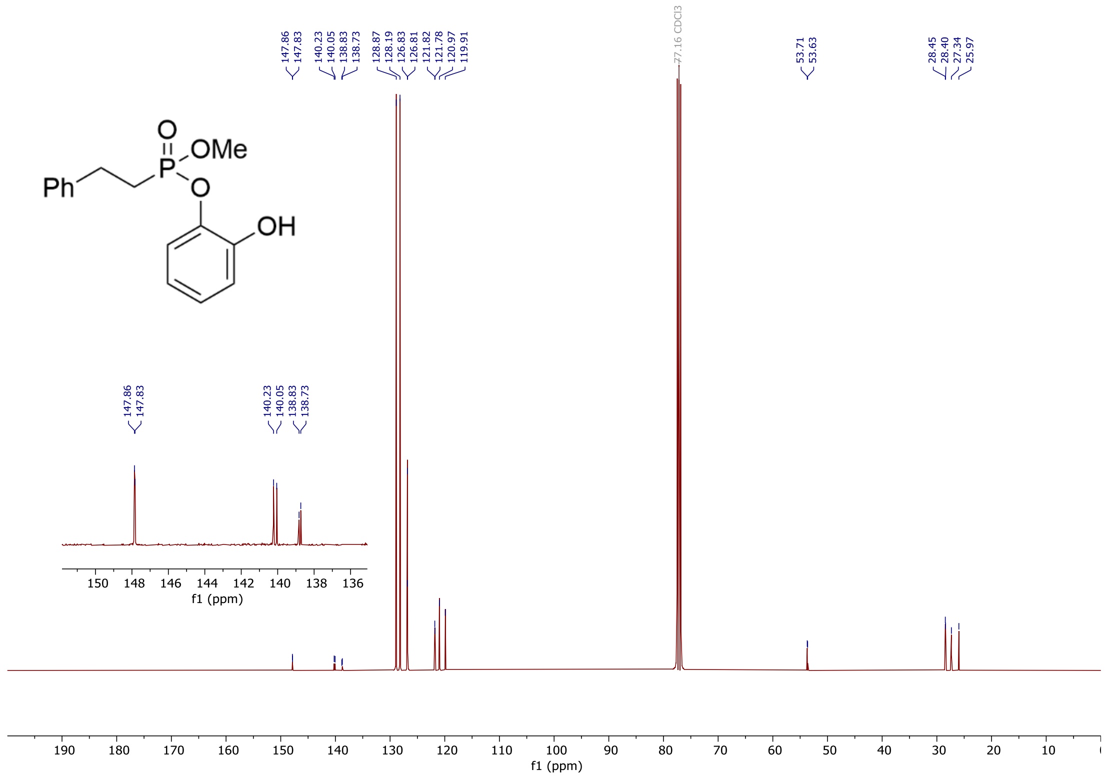

^{31}P NMR (162 MHz, $CDCl_3$): **3ar**

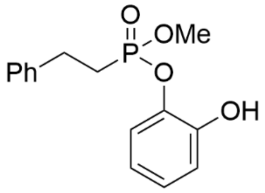

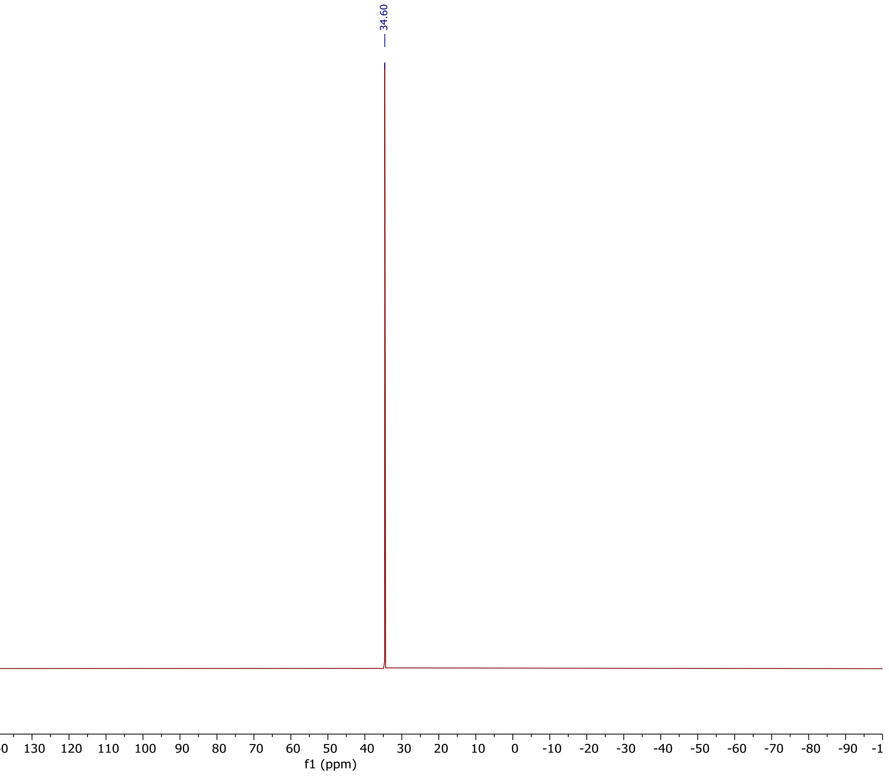

^{1}H NMR (400 MHz, CDCl3): **3as**

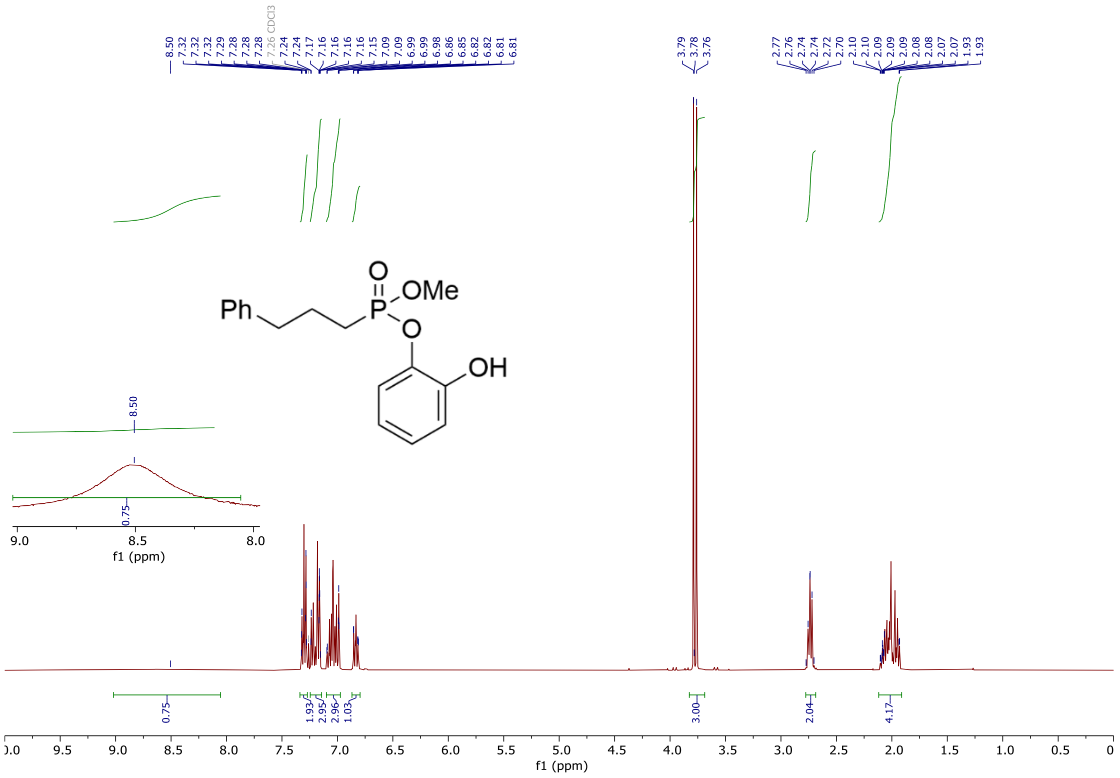

^{13}C NMR (101 MHz, CDCl3): **3as**

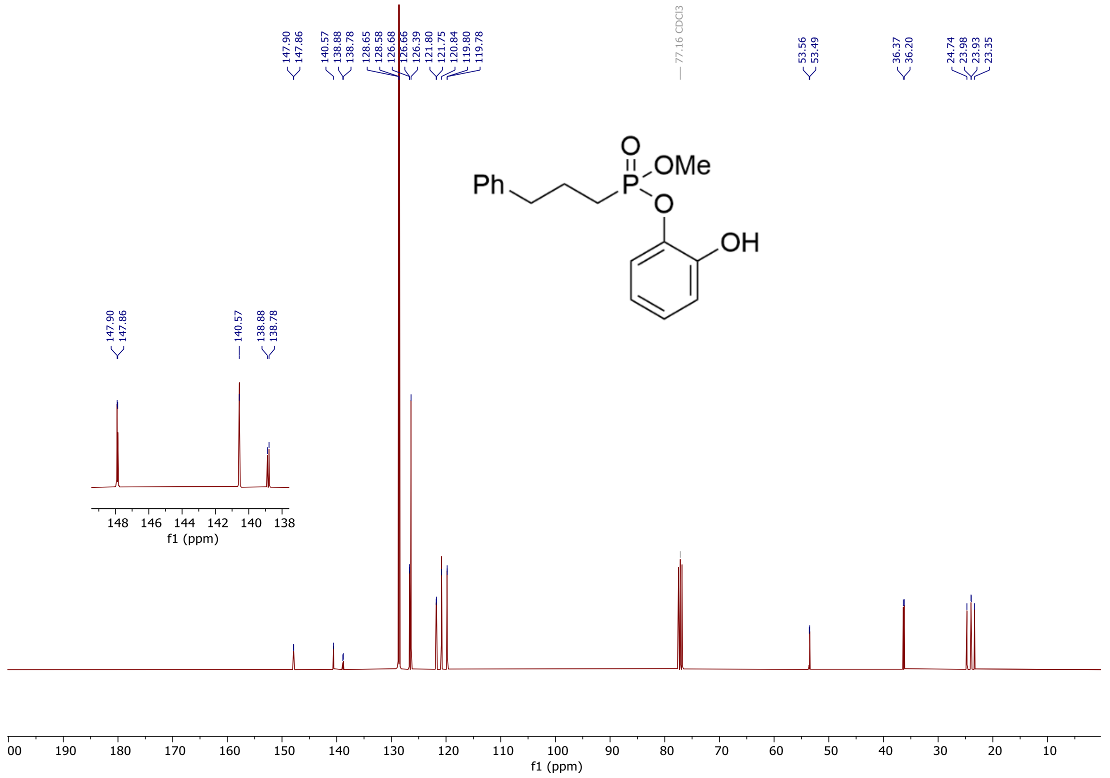

^{31}P NMR (162 MHz, $CDCl_3$): **3as**

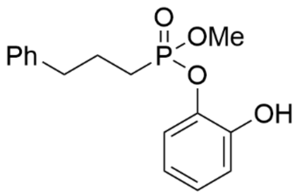

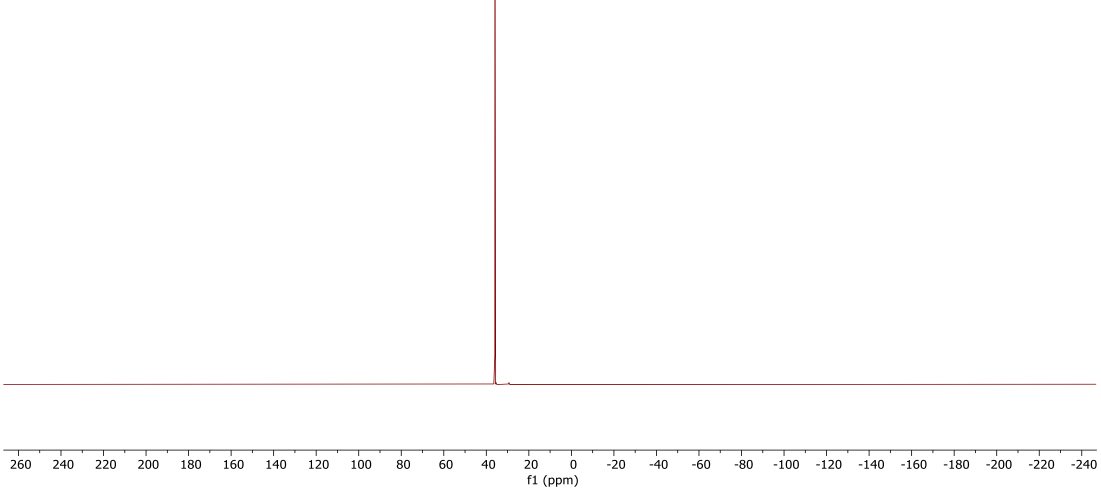

^{1}H NMR (400 MHz, $CDCl_3$): **3at**

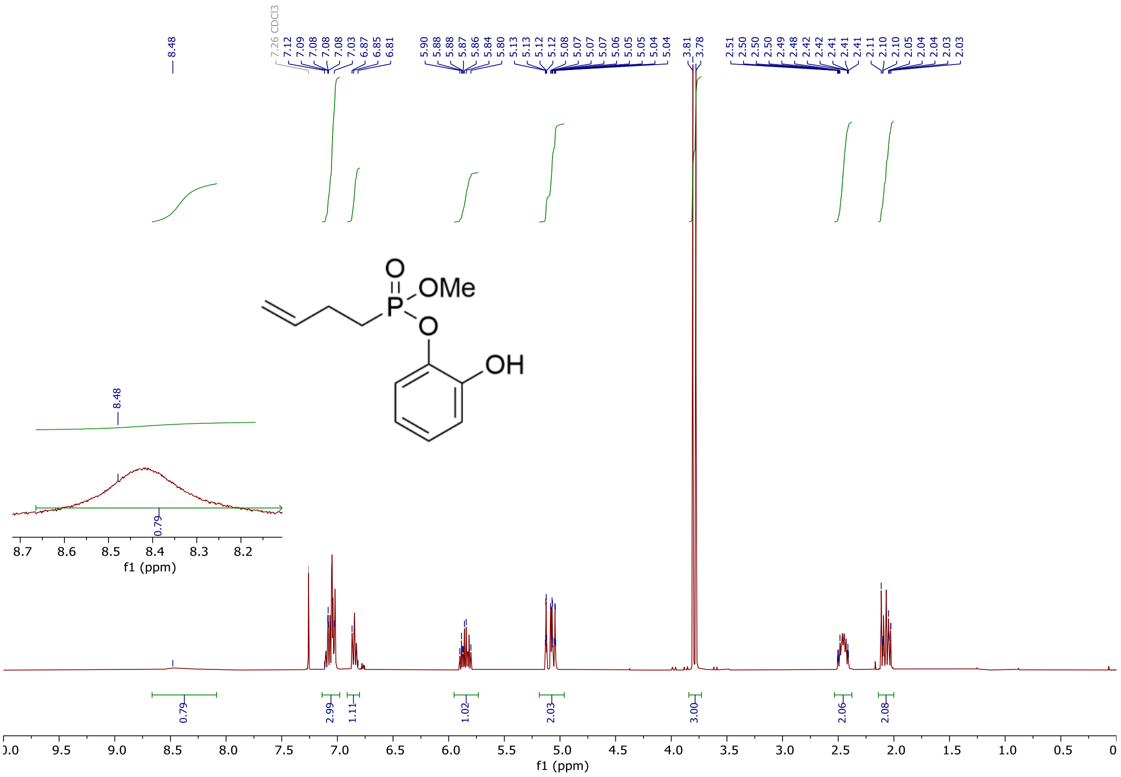

^{13}C NMR (101 MHz, $CDCl_3$): **3at**

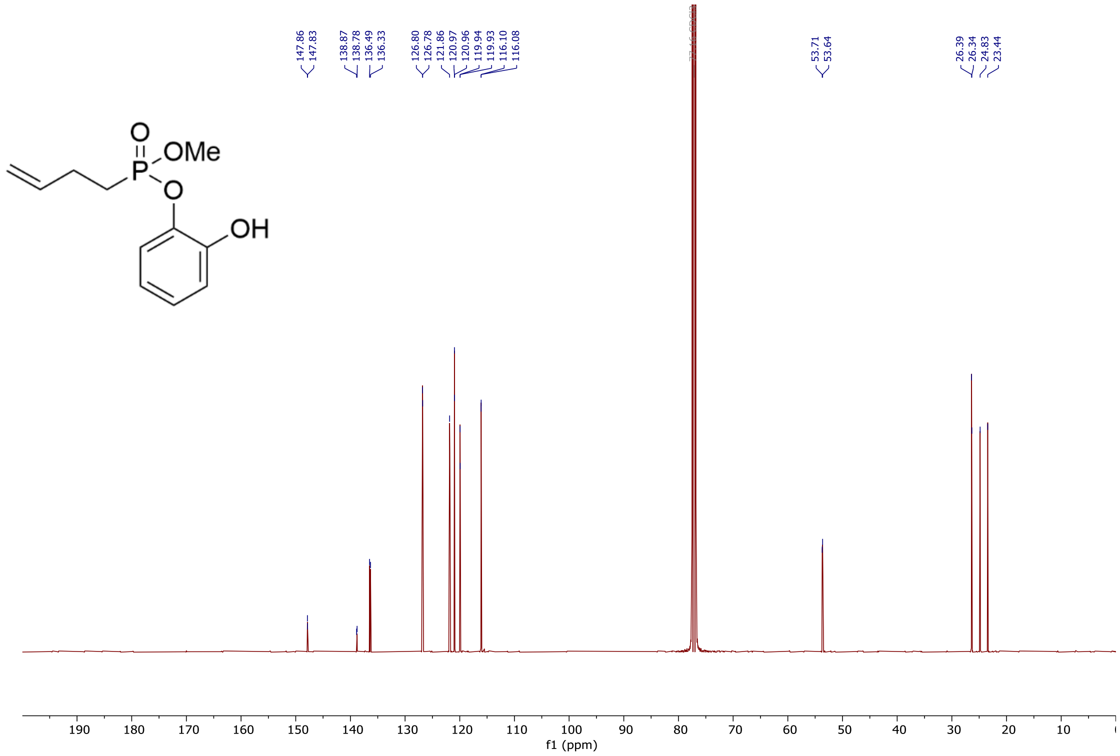

^{31}P NMR (162 MHz, $CDCl_3$): **3at**

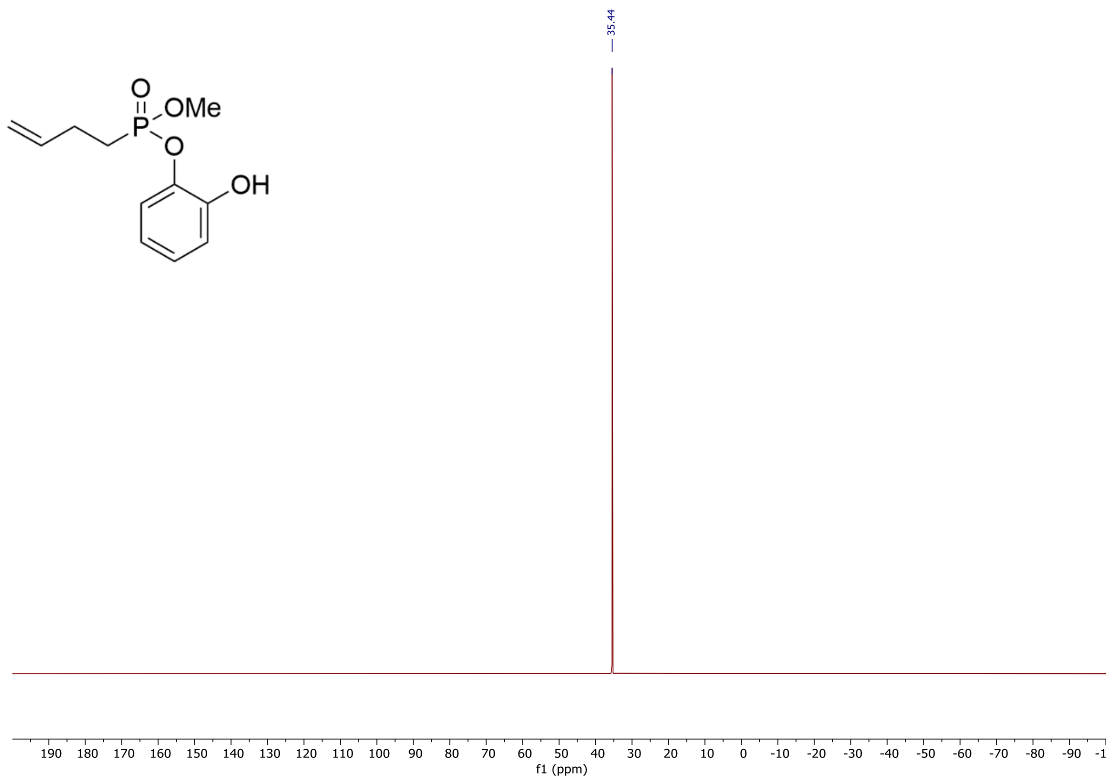

^{1}H NMR (400 MHz, $CDCl_3$): **3au**

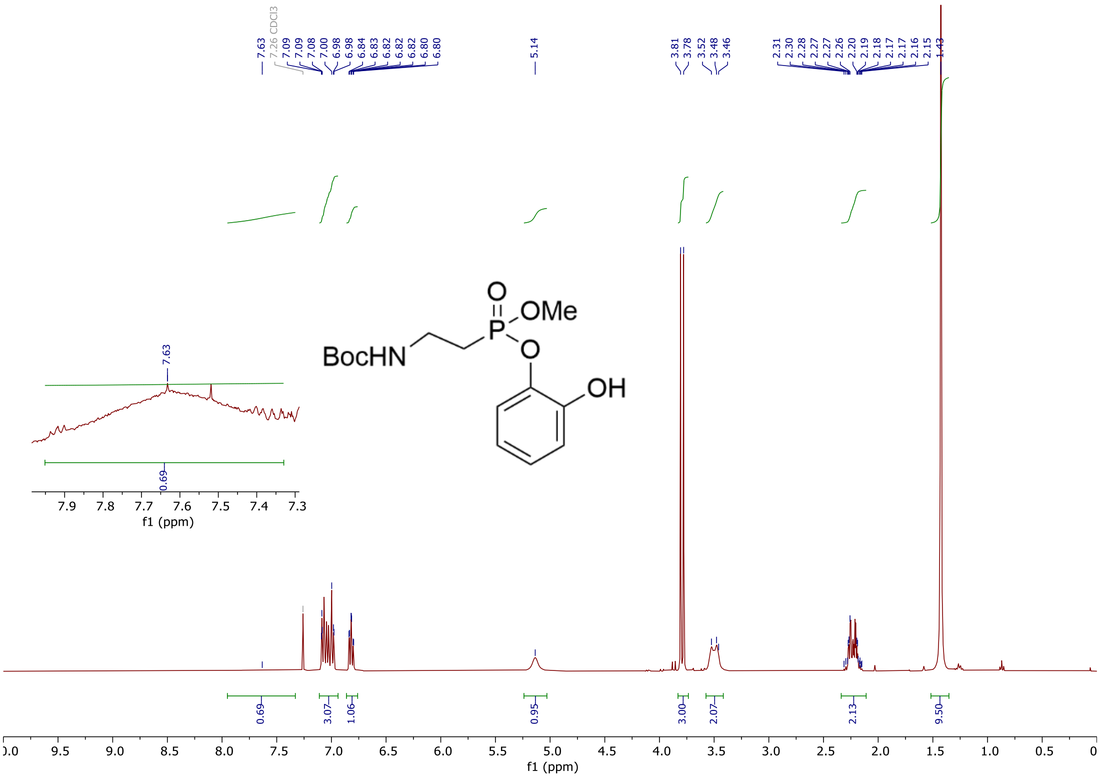

^{13}C NMR (101 MHz, $CDCl_3$): **3au**

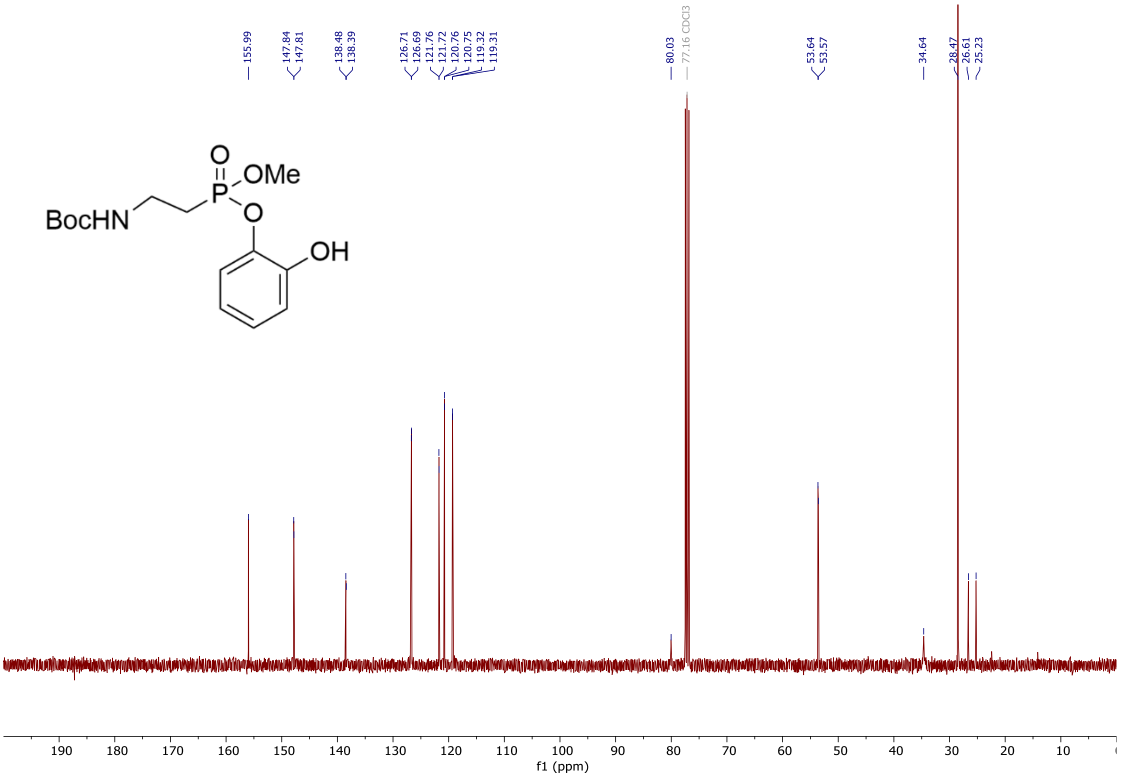

^{31}P NMR (162 MHz, $CDCl_3$): **3au**

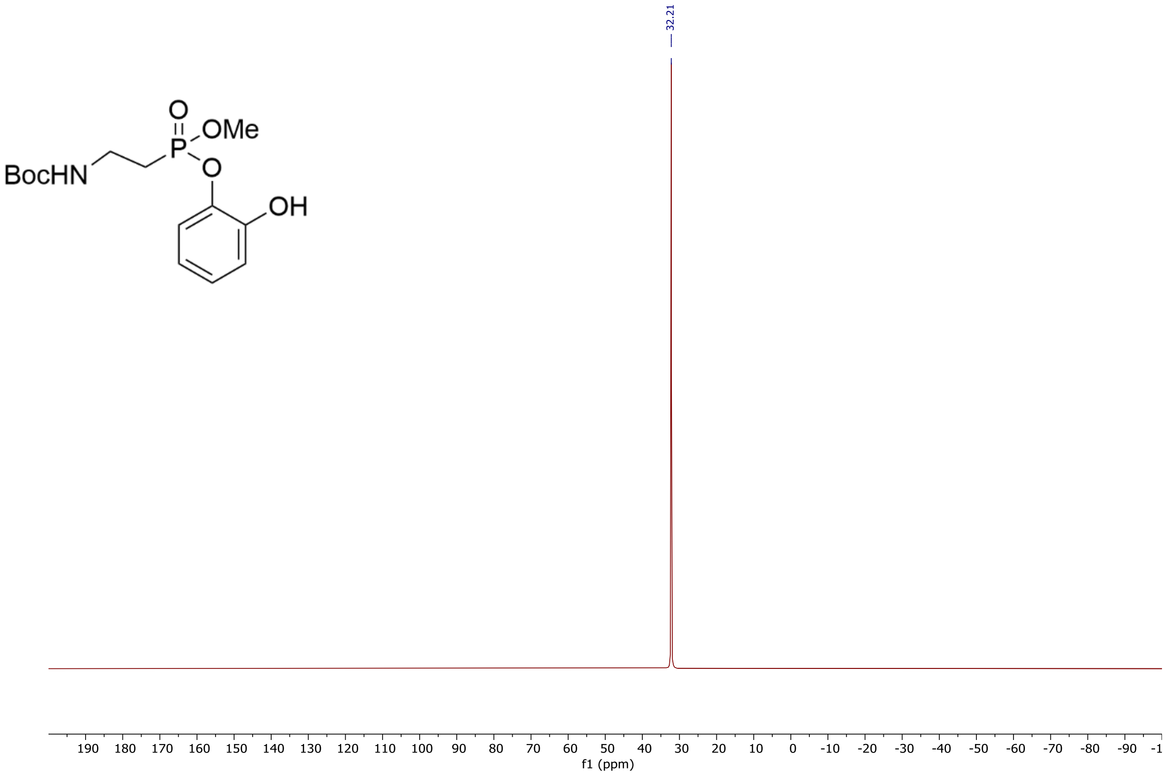

^{1}H NMR (400 MHz, $CDCl_3$): **3av**

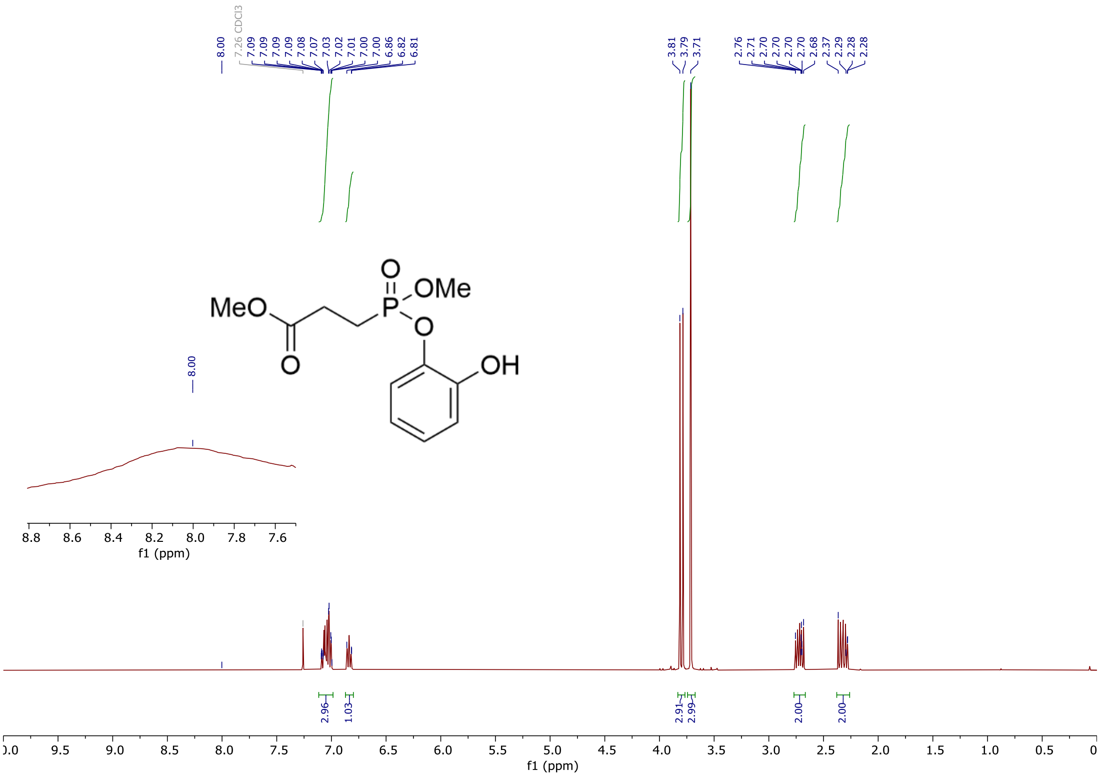

^{13}C NMR (101 MHz, $CDCl_3$): **3av**

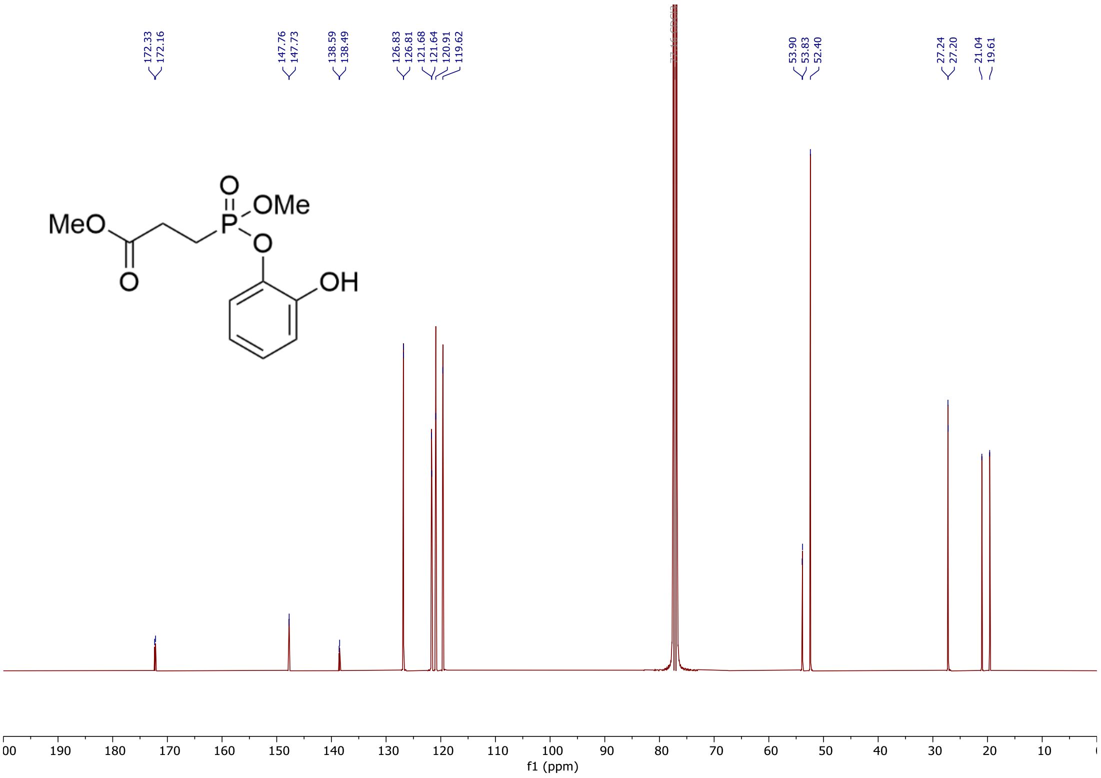

^{31}P NMR (162 MHz, $CDCl_3$): **3av**

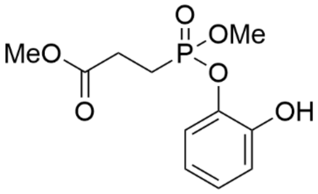

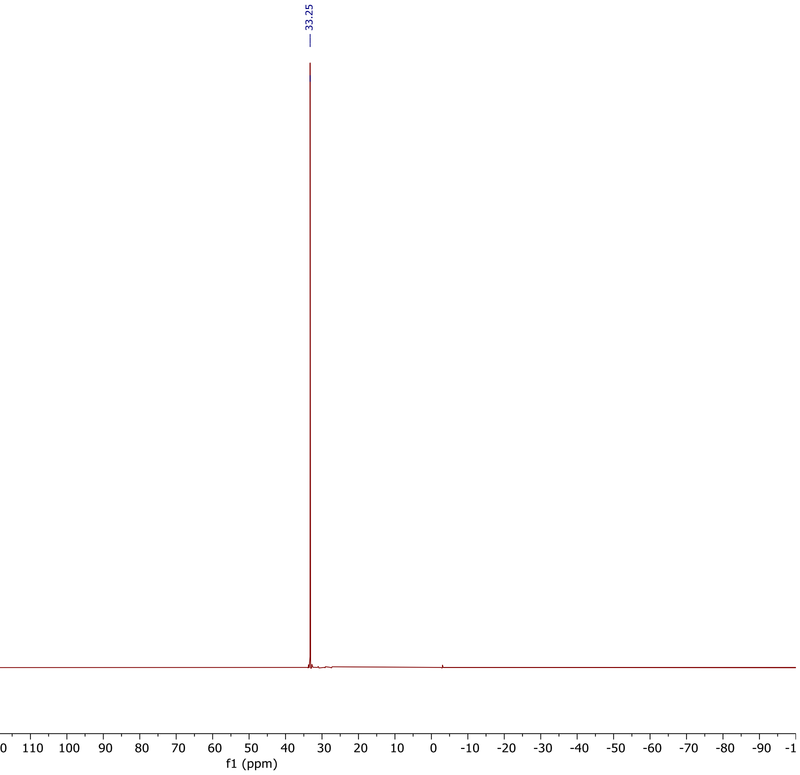

^{1}H NMR (400 MHz, $CDCl_3$): **3a**

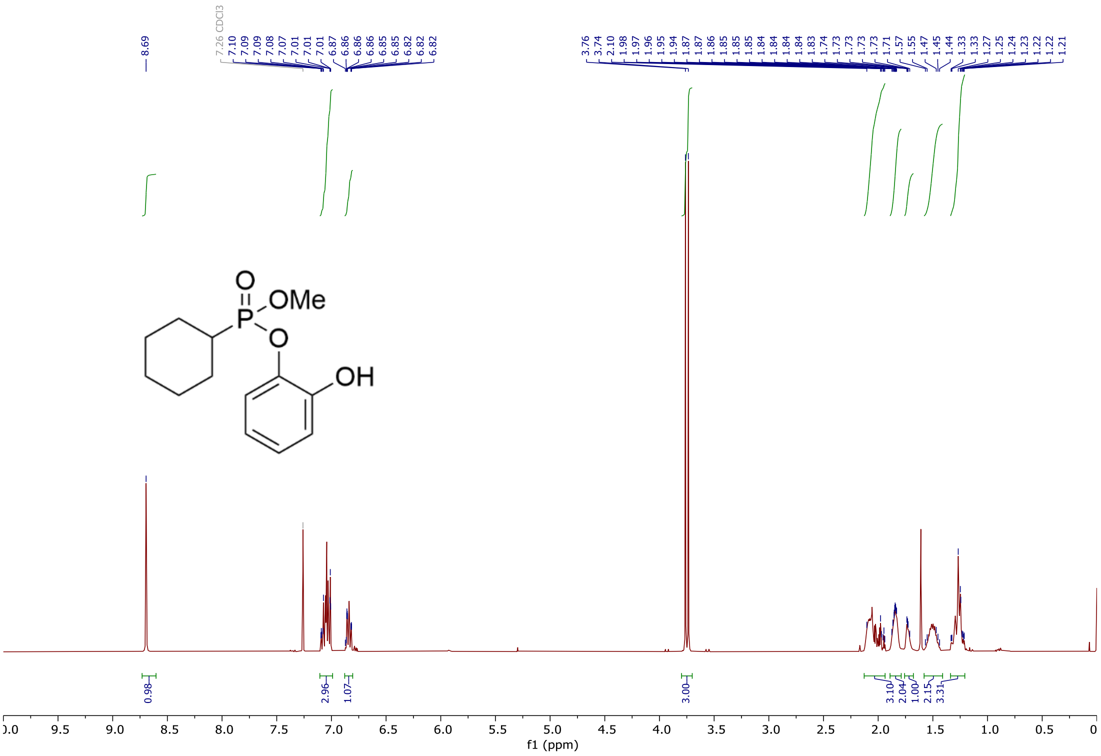

^{13}C NMR (101 MHz, $CDCl_3$): **3a**

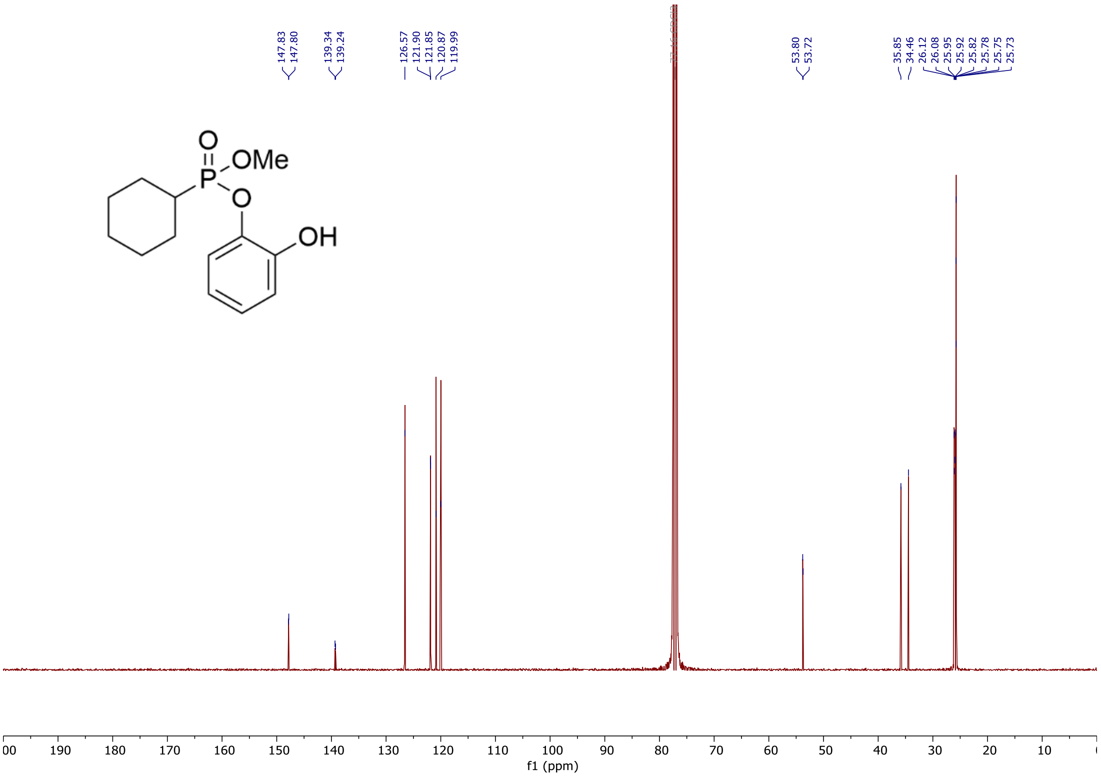

^{31}**P NMR** (162 MHz, $CDCl_3$): **3a**

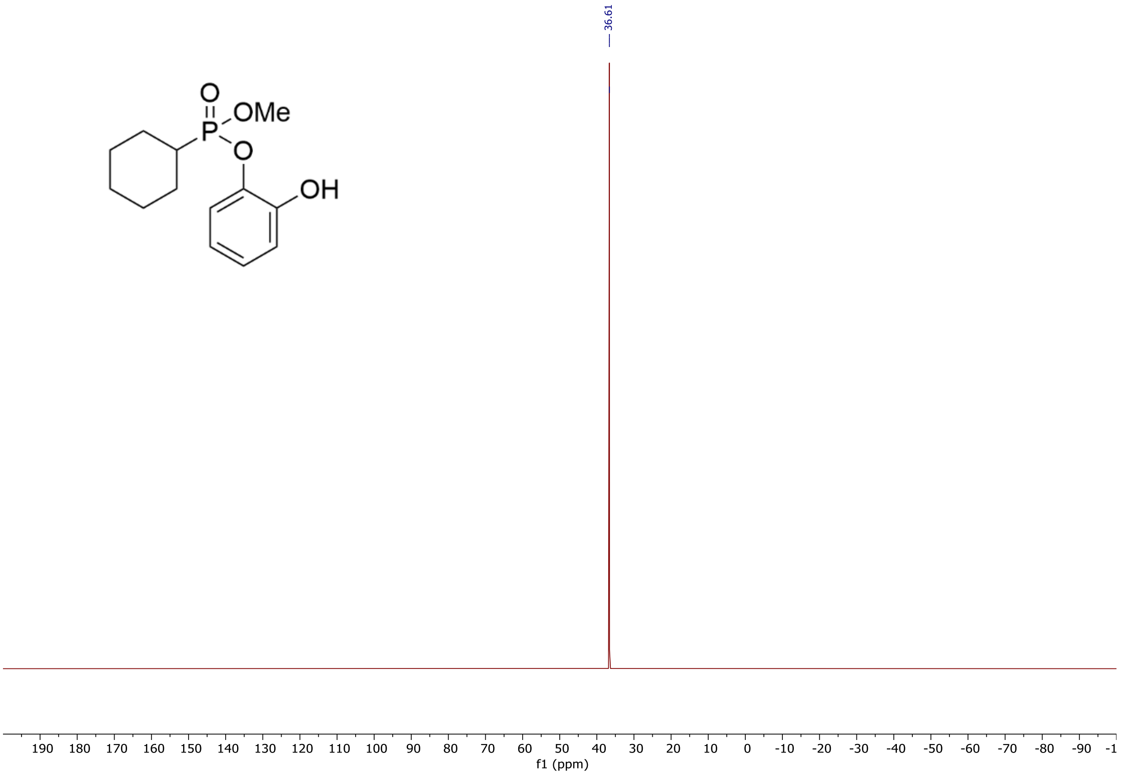

^{1}H NMR (500 MHz, CDCl$_3$): **3aw**

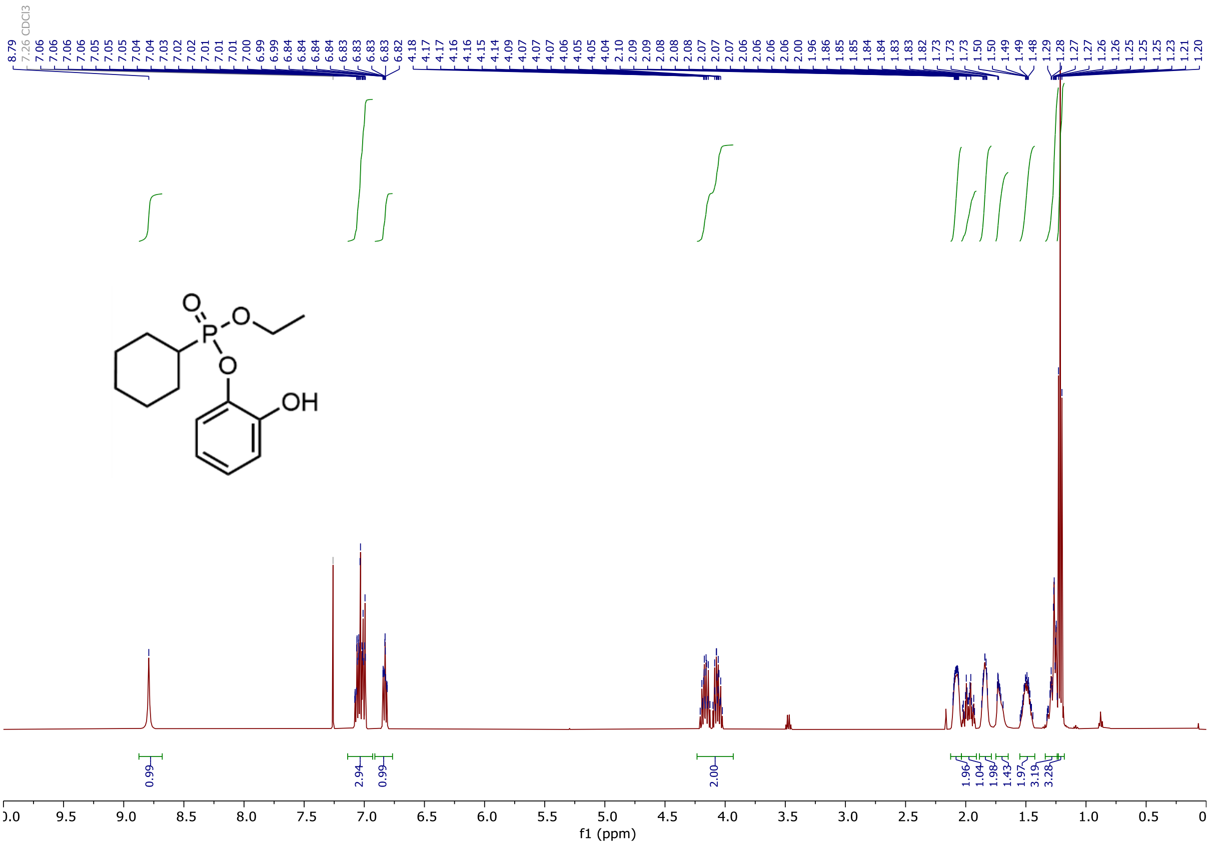

^{13}C NMR (126 MHz, CDCl$_3$): **3aw**

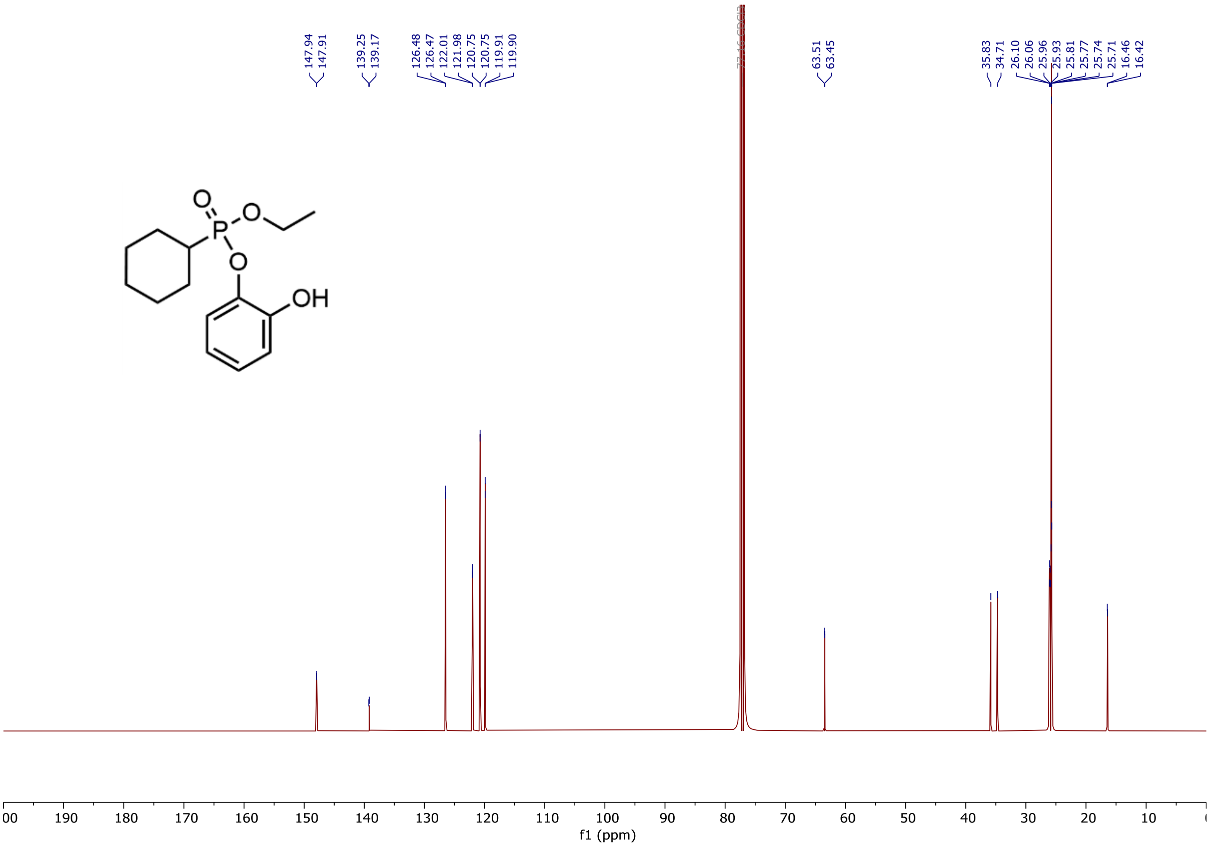

^{31}P NMR (162 MHz, $CDCl_3$): **3aw**

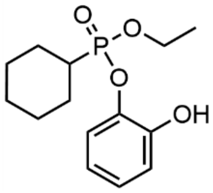

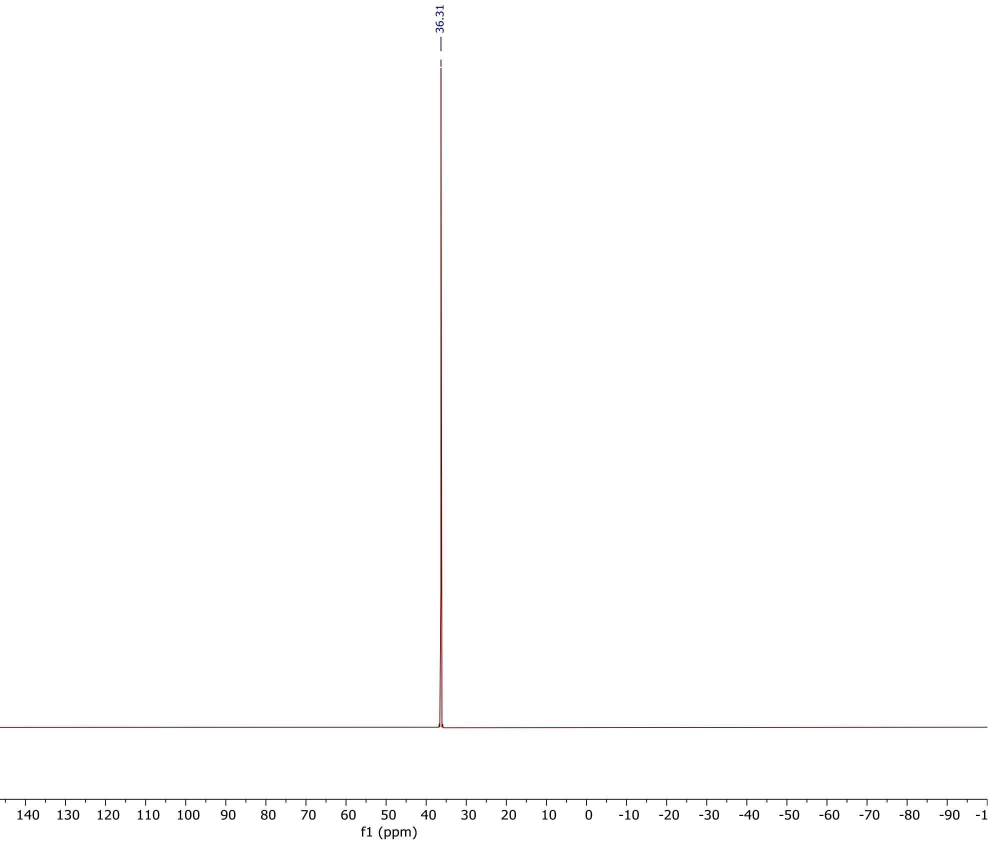

^{1}H NMR (500 MHz, $CDCl_3$): **3ax**

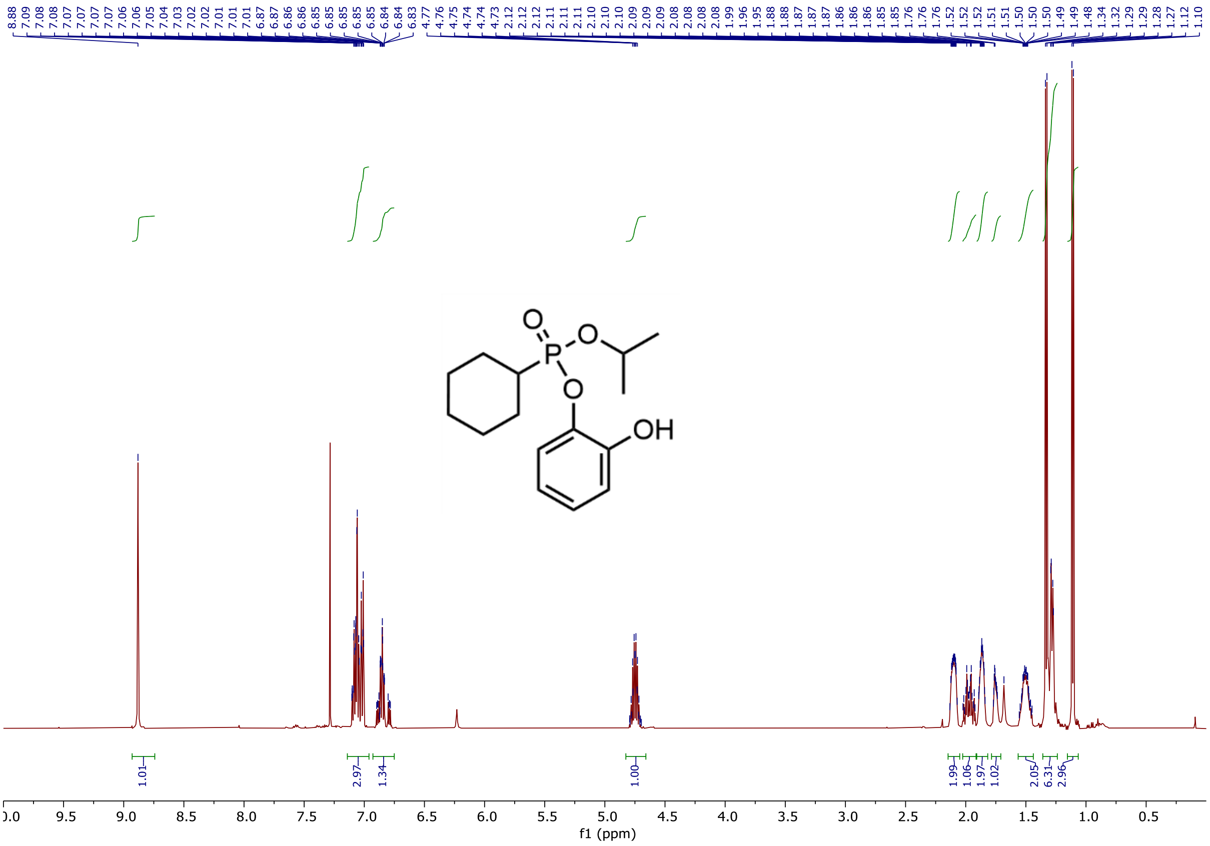

^{13}C NMR (126 MHz, $CDCl_3$): **3ax**

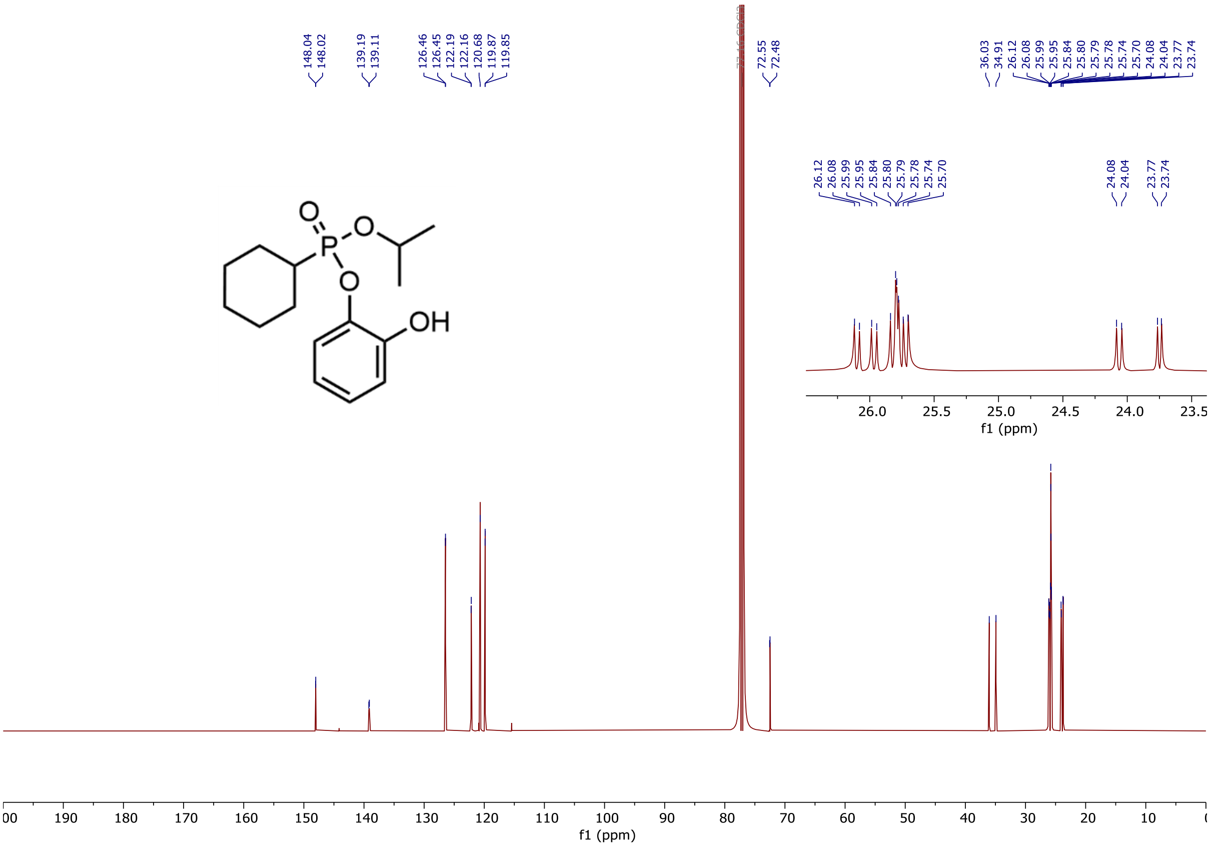

^{31}**P NMR** (162 MHz, $CDCl_3$): **3ax**

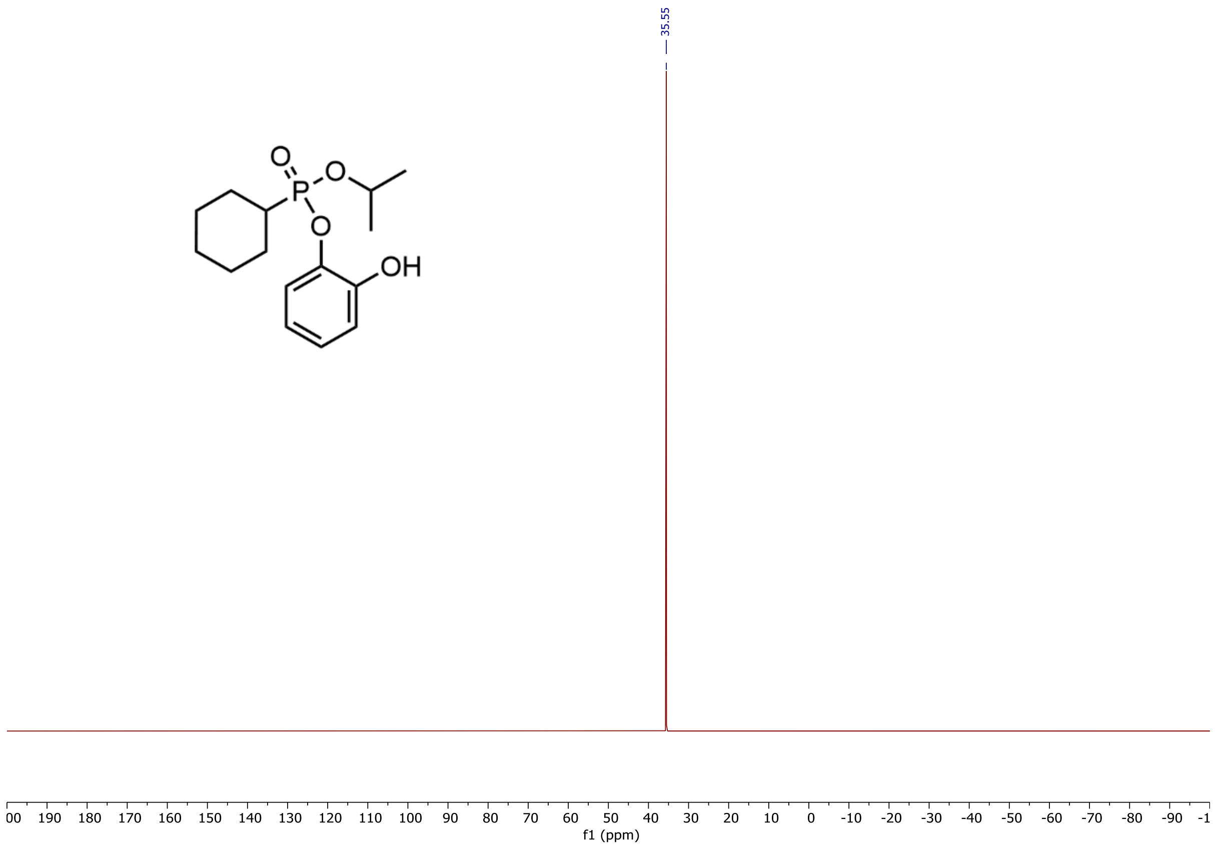

^{1}H NMR (500 MHz, $CDCl_3$): **3ay**

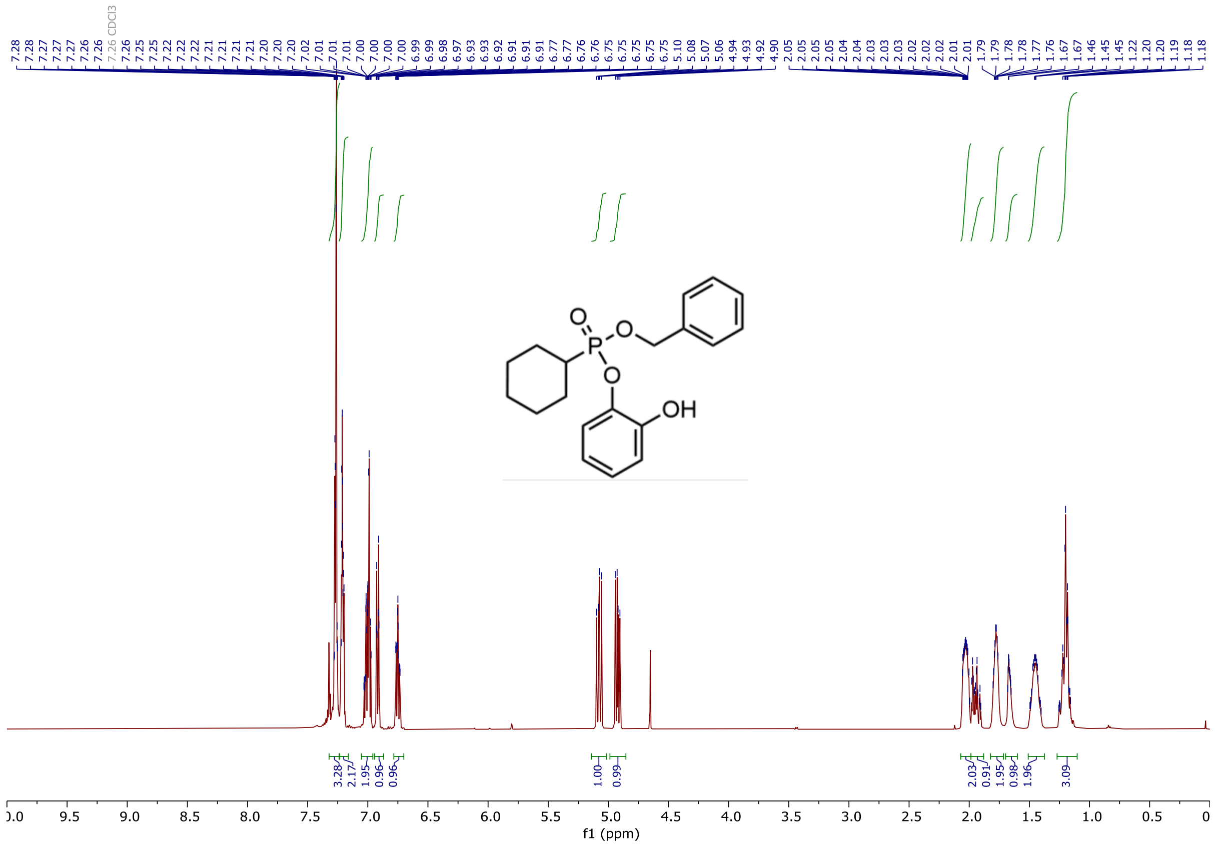

^{13}C NMR (126 MHz, $CDCl_3$): **3ay**

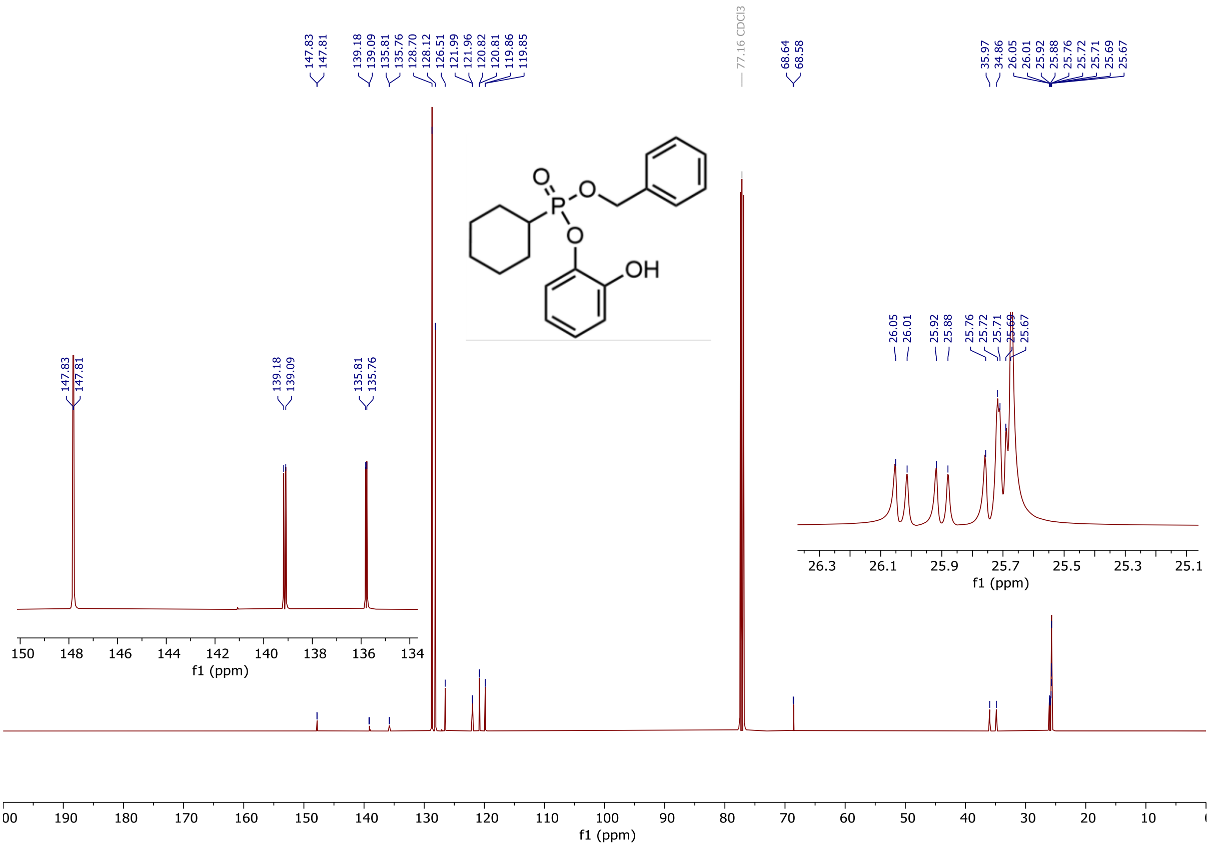

^{31}P NMR (162 MHz, $CDCl_3$): **3ay**

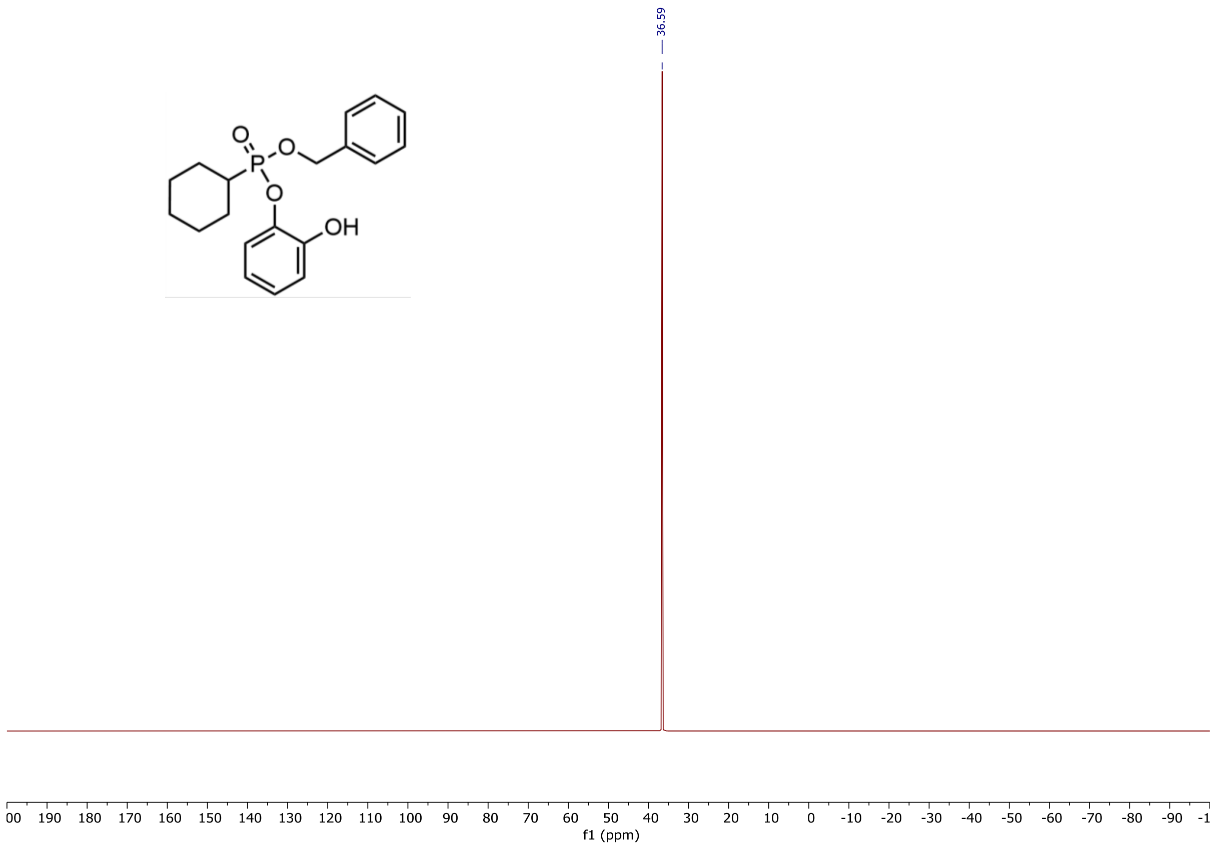

^{1}H NMR (400 MHz, $CDCl_3$): **3az**

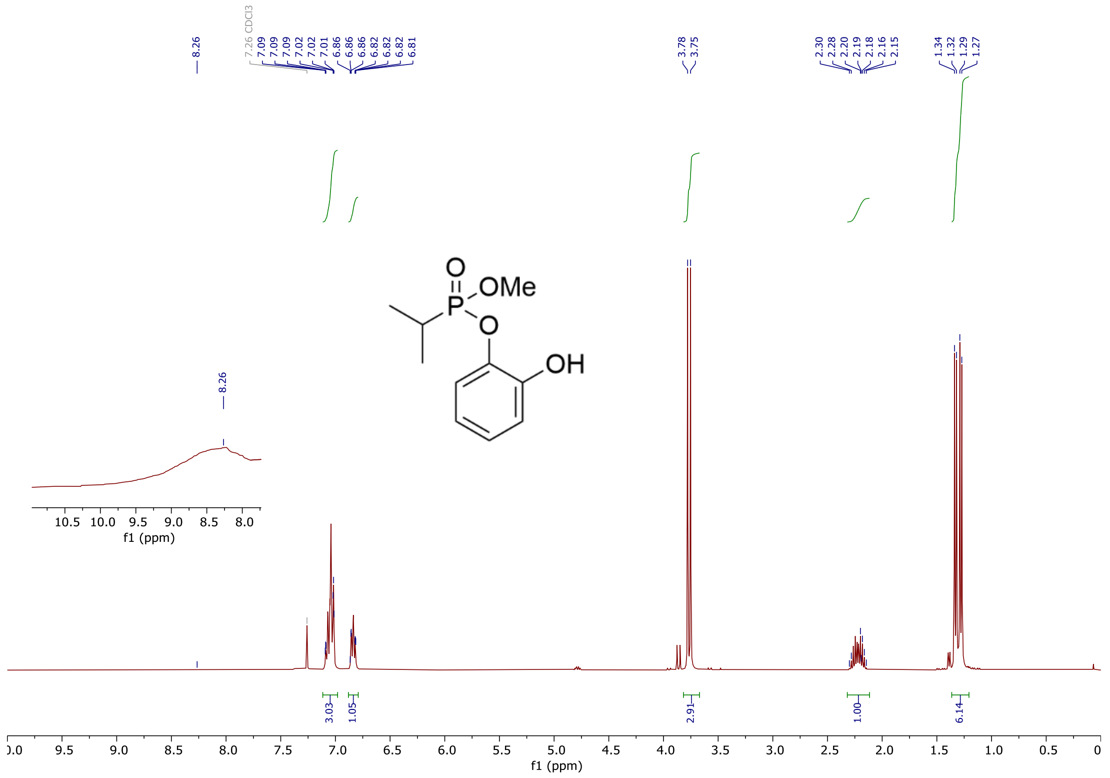

^{13}C NMR (101 MHz, $CDCl_3$): **3az**

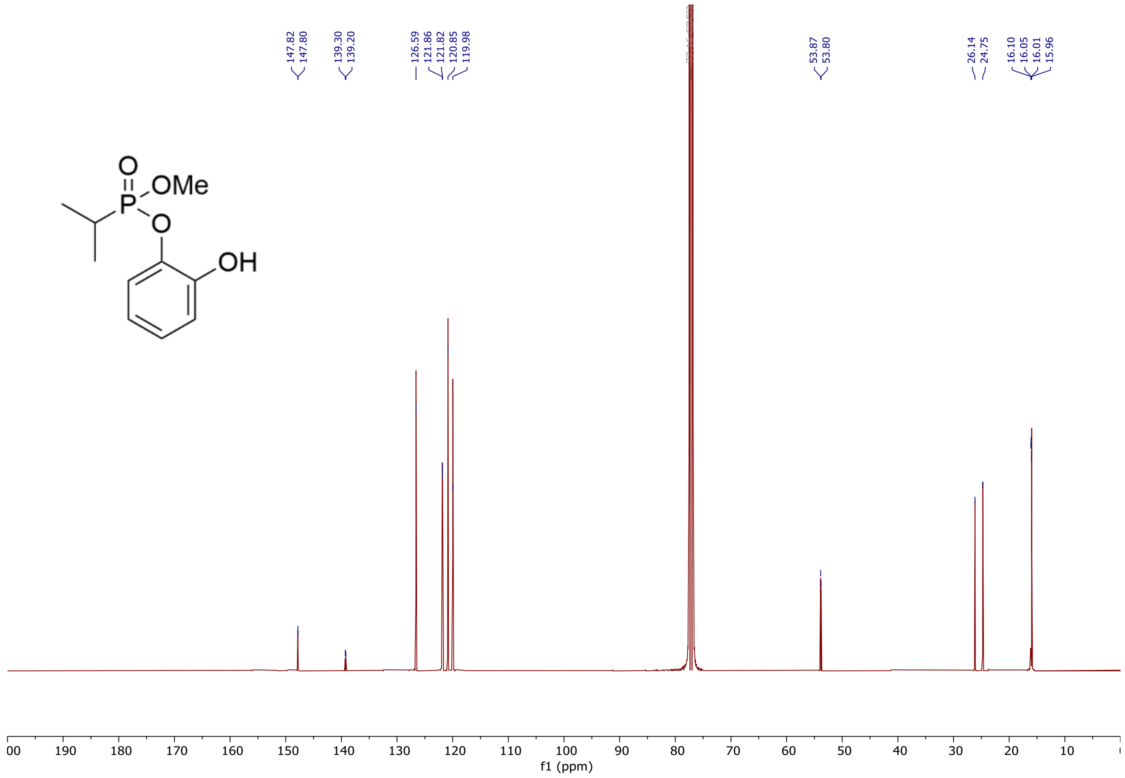

^{31}**P NMR** (162 MHz, $CDCl_3$): **3az**

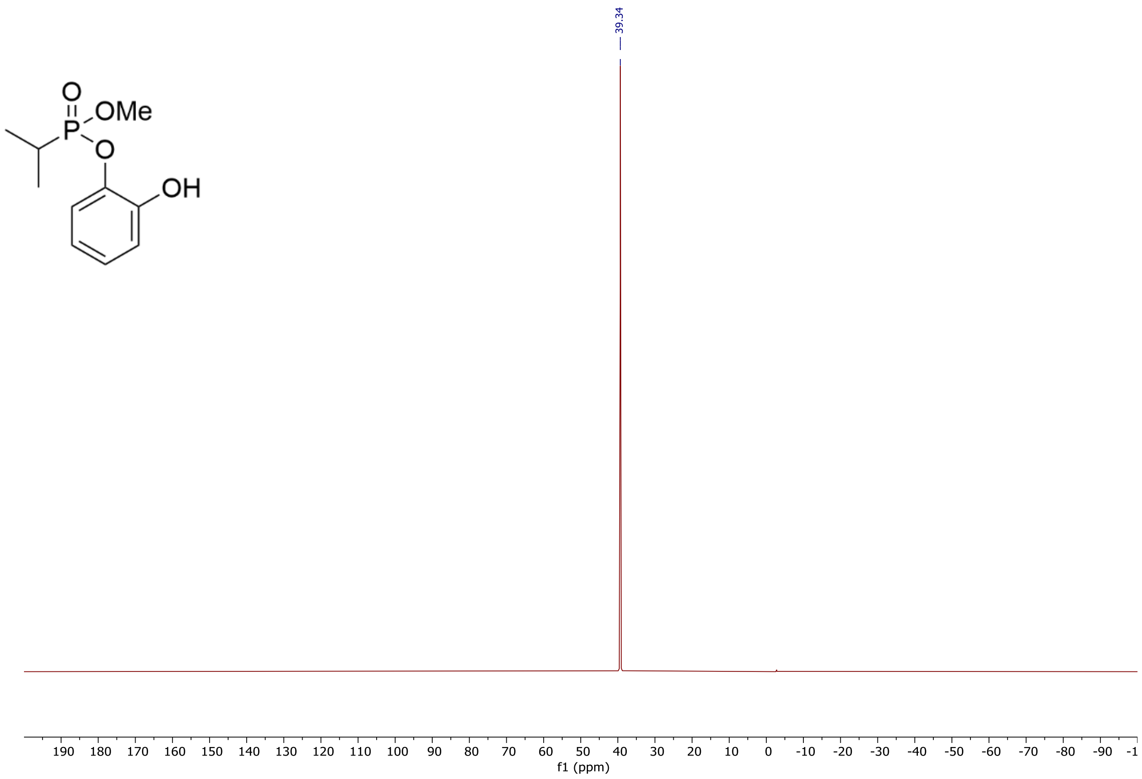

^{1}H NMR (400 MHz, $CDCl_3$): **3ba**

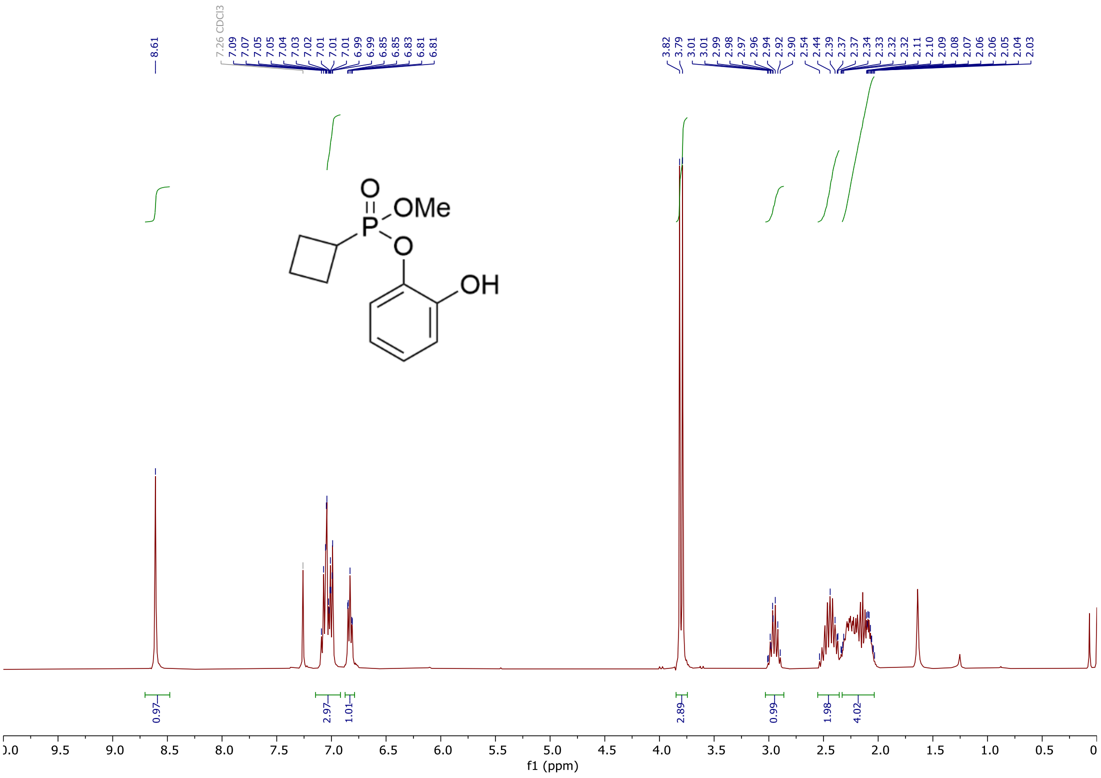

^{13}C NMR (101 MHz, $CDCl_3$): **3ba**

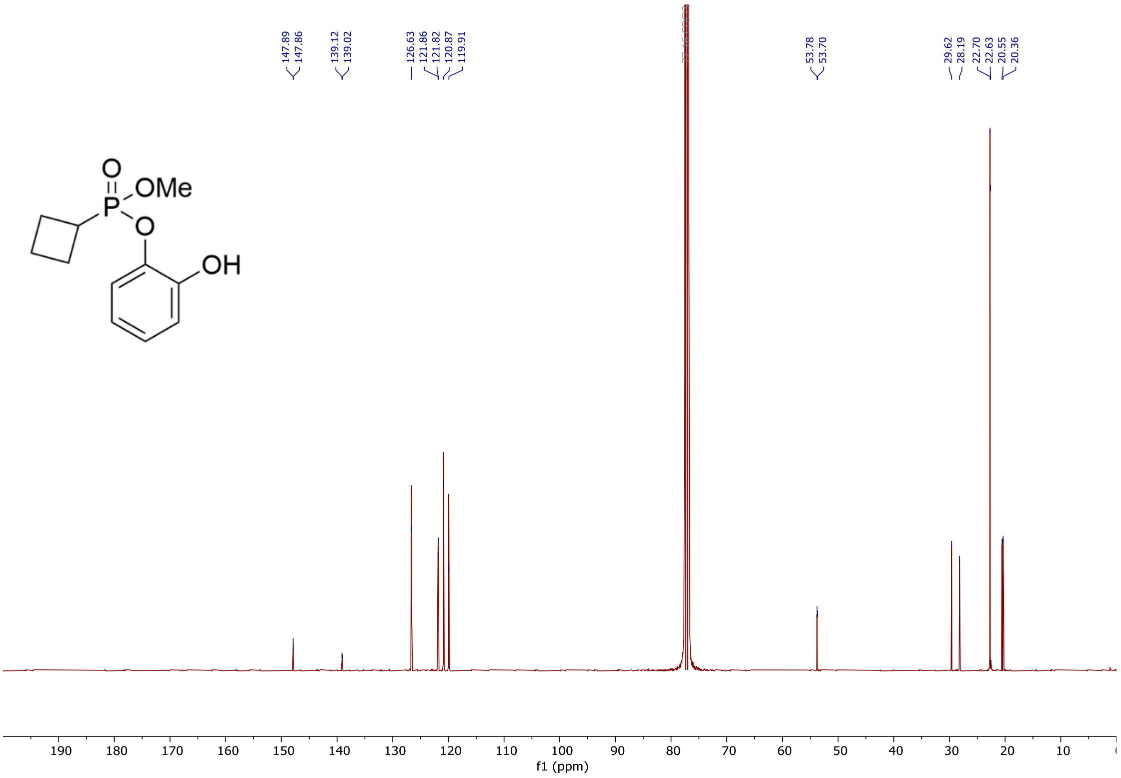

^{31}P NMR (162 MHz, $CDCl_3$): **3ba**

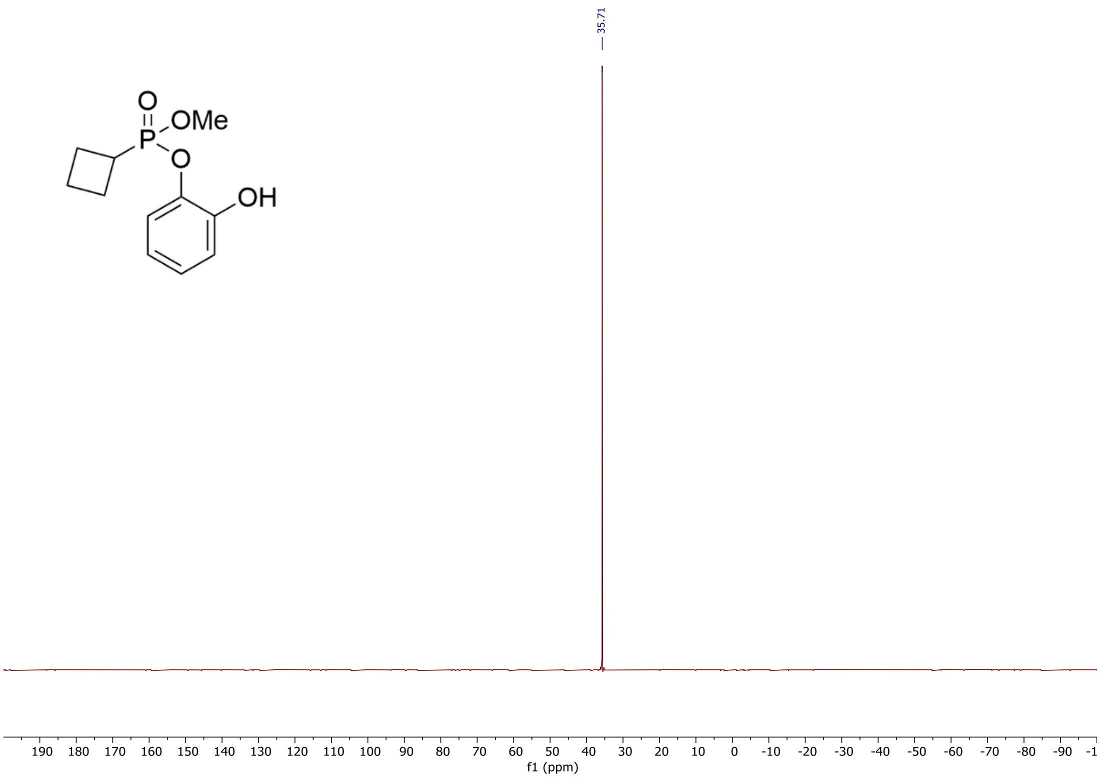

^{1}H NMR (400 MHz, CDCl3): **3bb**

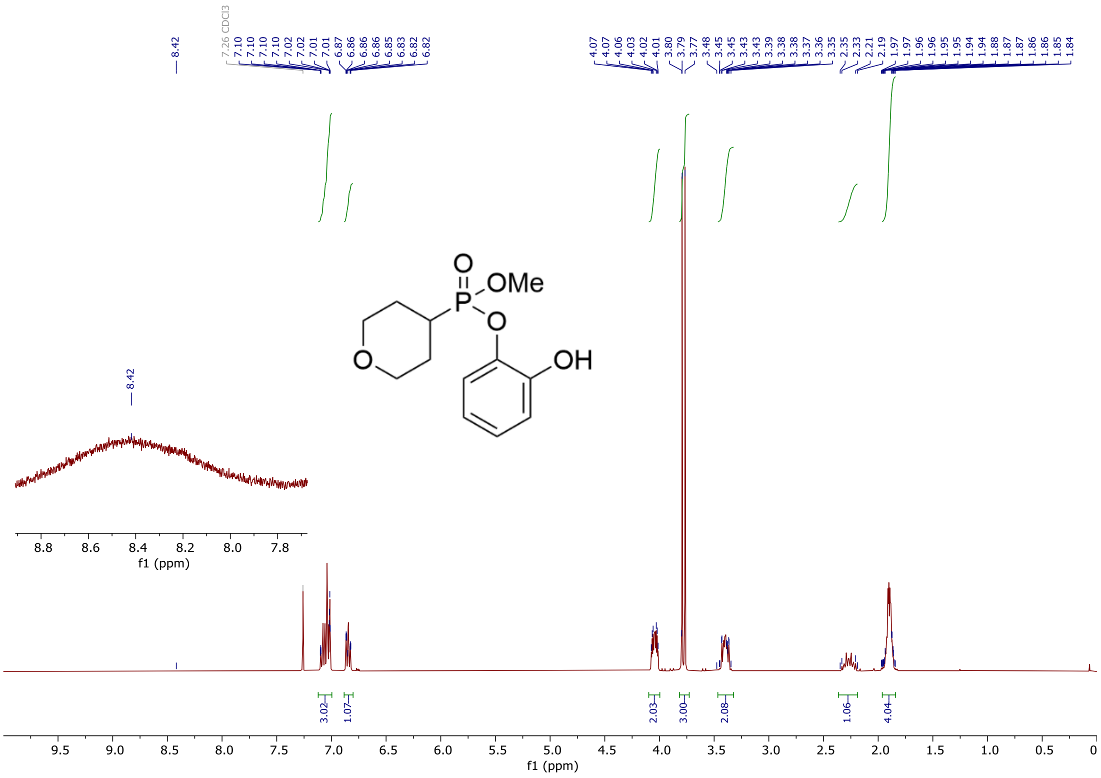

^{13}C NMR (101 MHz, CDCl3): **3bb**

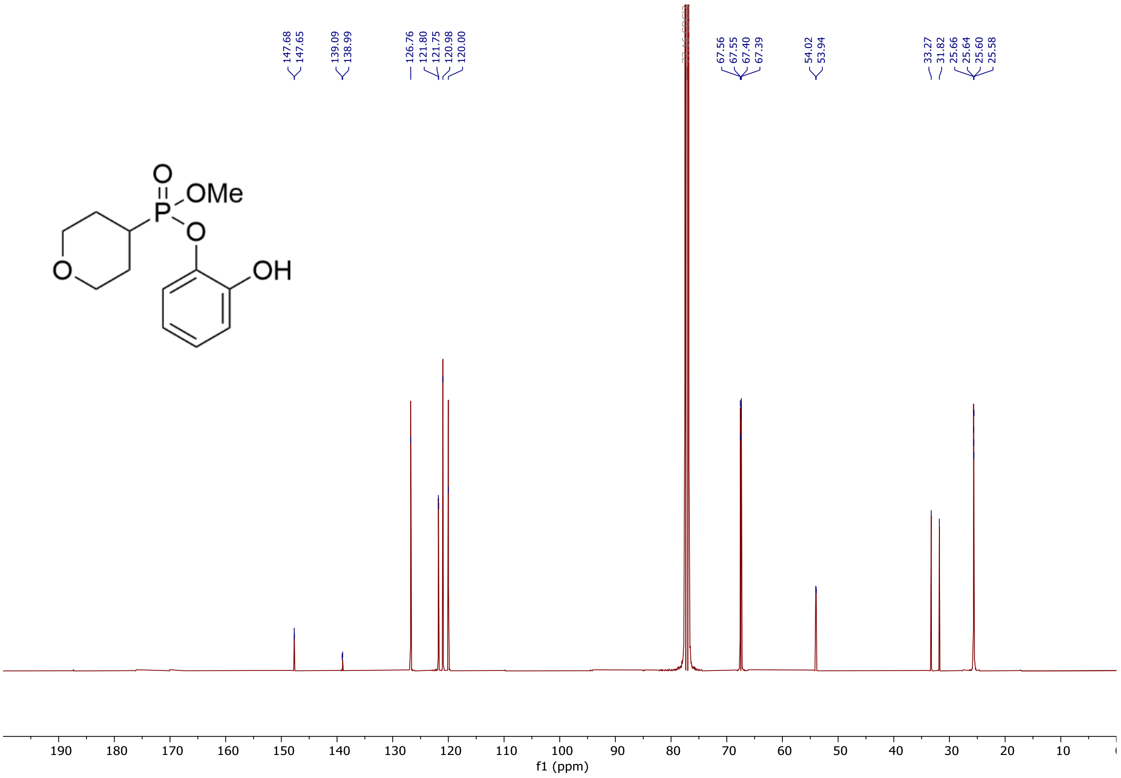

[31]**P NMR** (162 MHz, $CDCl_3$): **3bb**

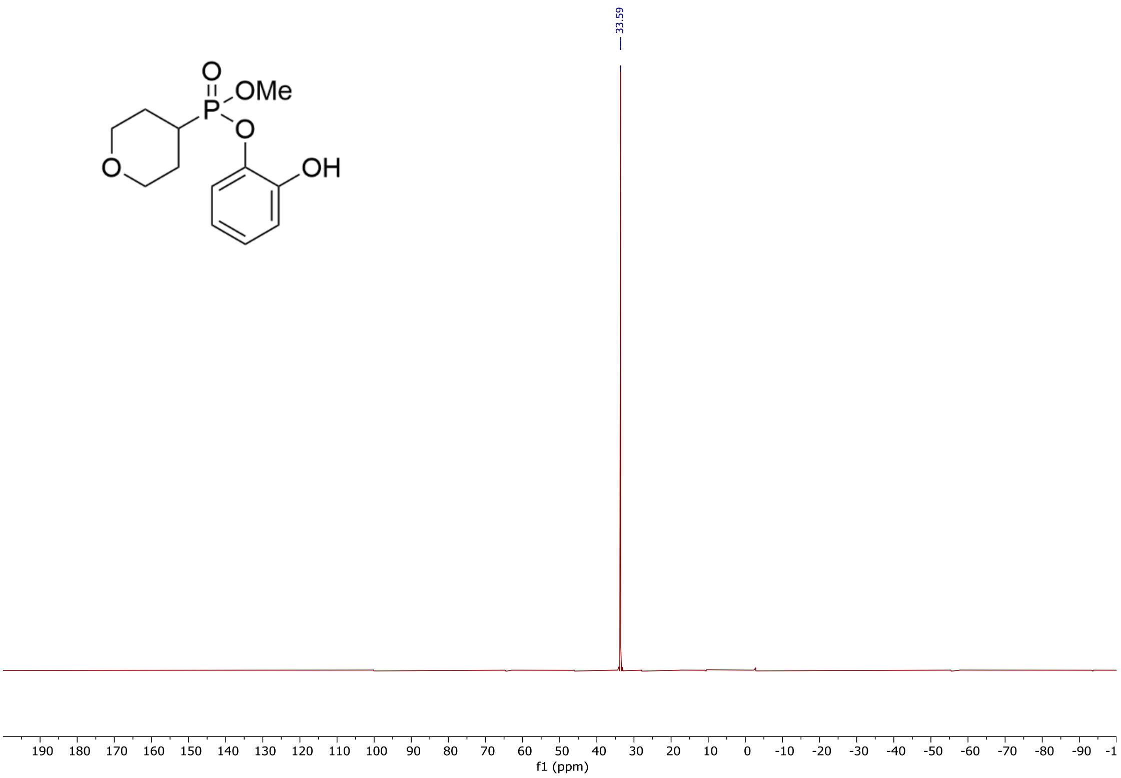

^{1}H NMR (400 MHz, $CDCl_3$): **3bc**

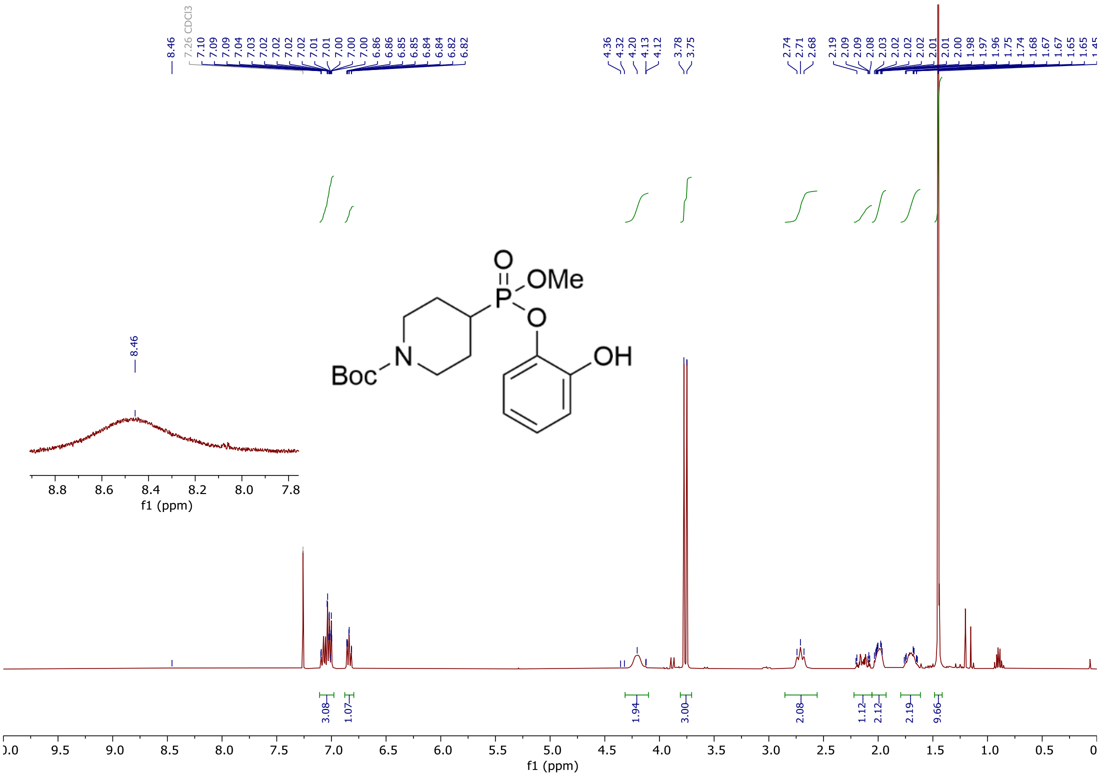

^{13}C NMR (101 MHz, $CDCl_3$): **3bc**

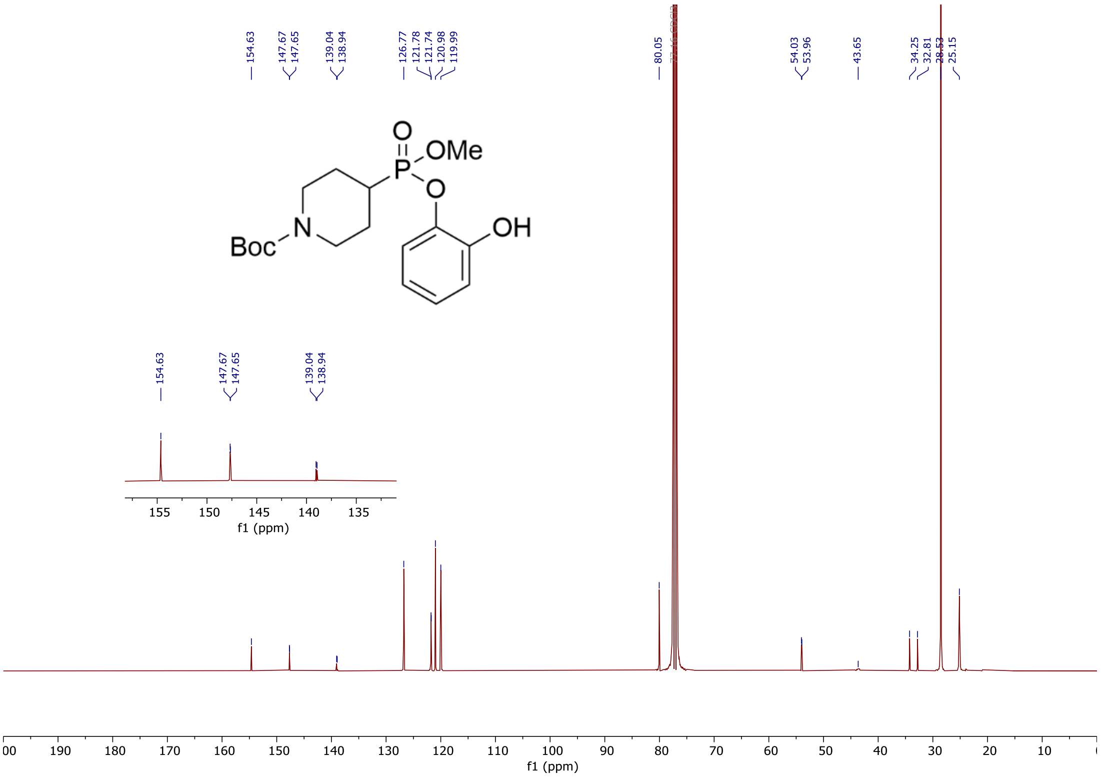

^{31}**P NMR** (162 MHz, $CDCl_3$): **3bc**

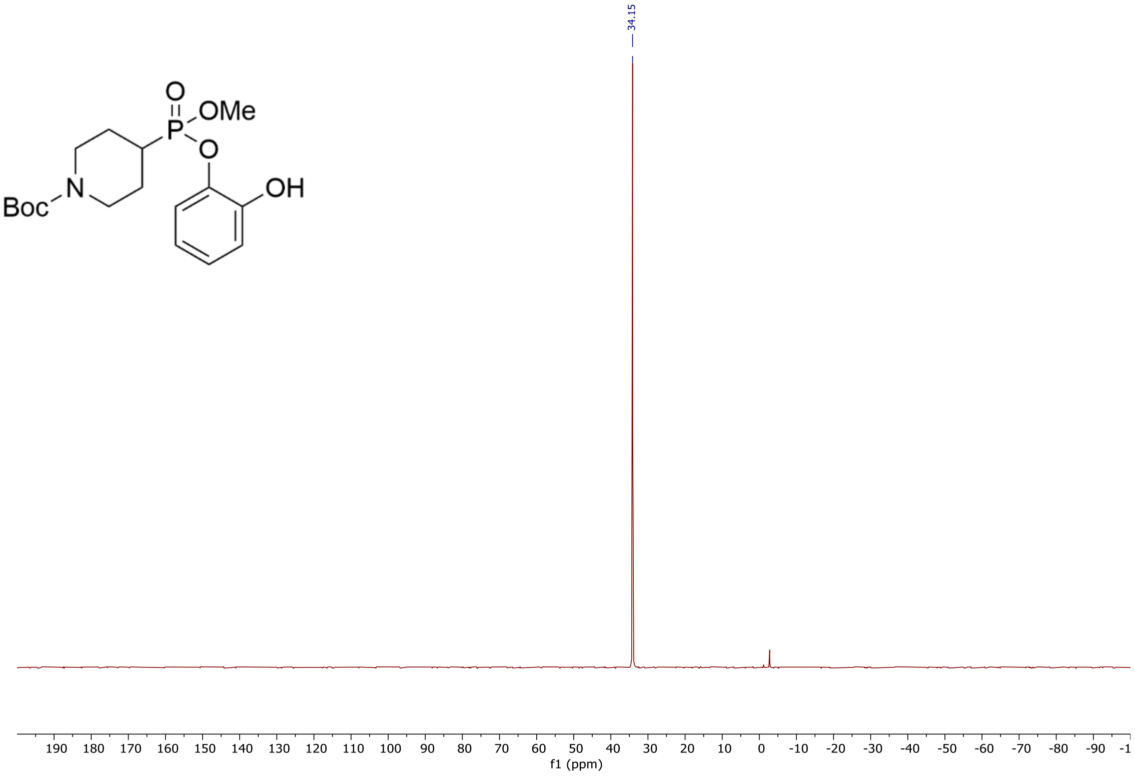

^{1}H NMR (400 MHz, $CDCl_3$): **3bd'**

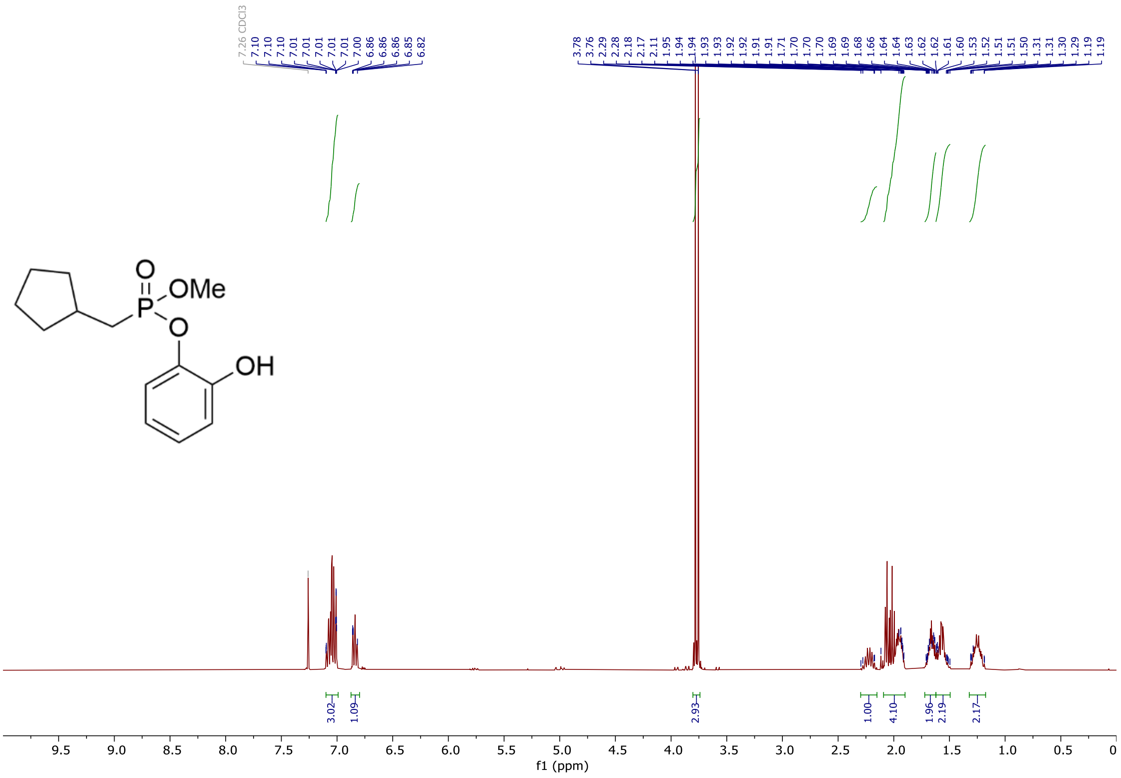

^{13}C NMR (101 MHz, $CDCl_3$): **3bd'**

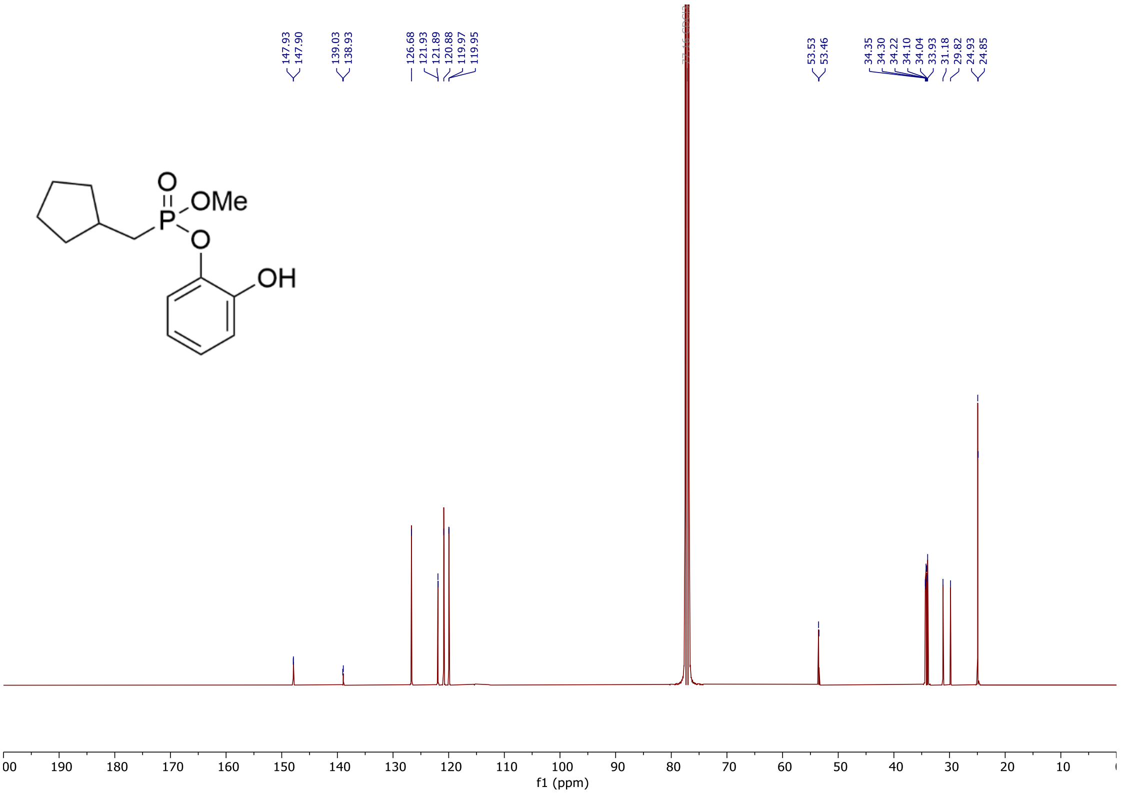

^{31}**P NMR** (162 MHz, $CDCl_3$): **3bd'**

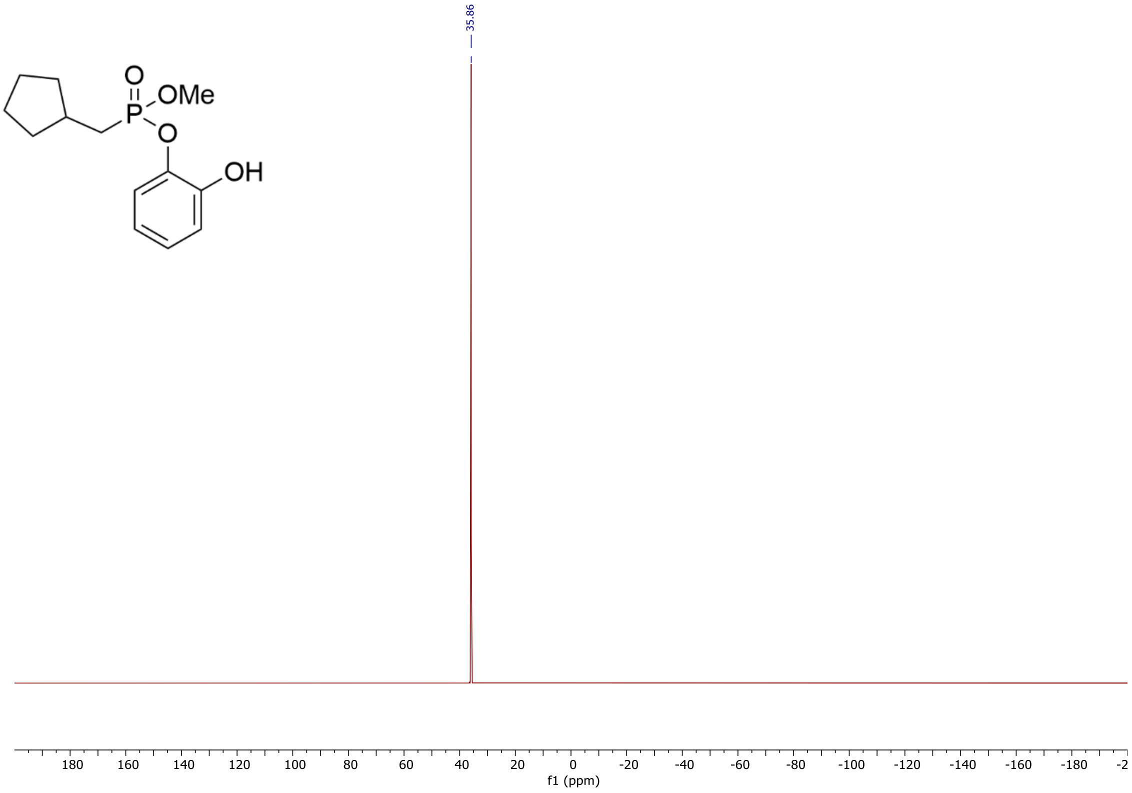

^{1}H NMR (400 MHz, $CDCl_3$): **3bd**

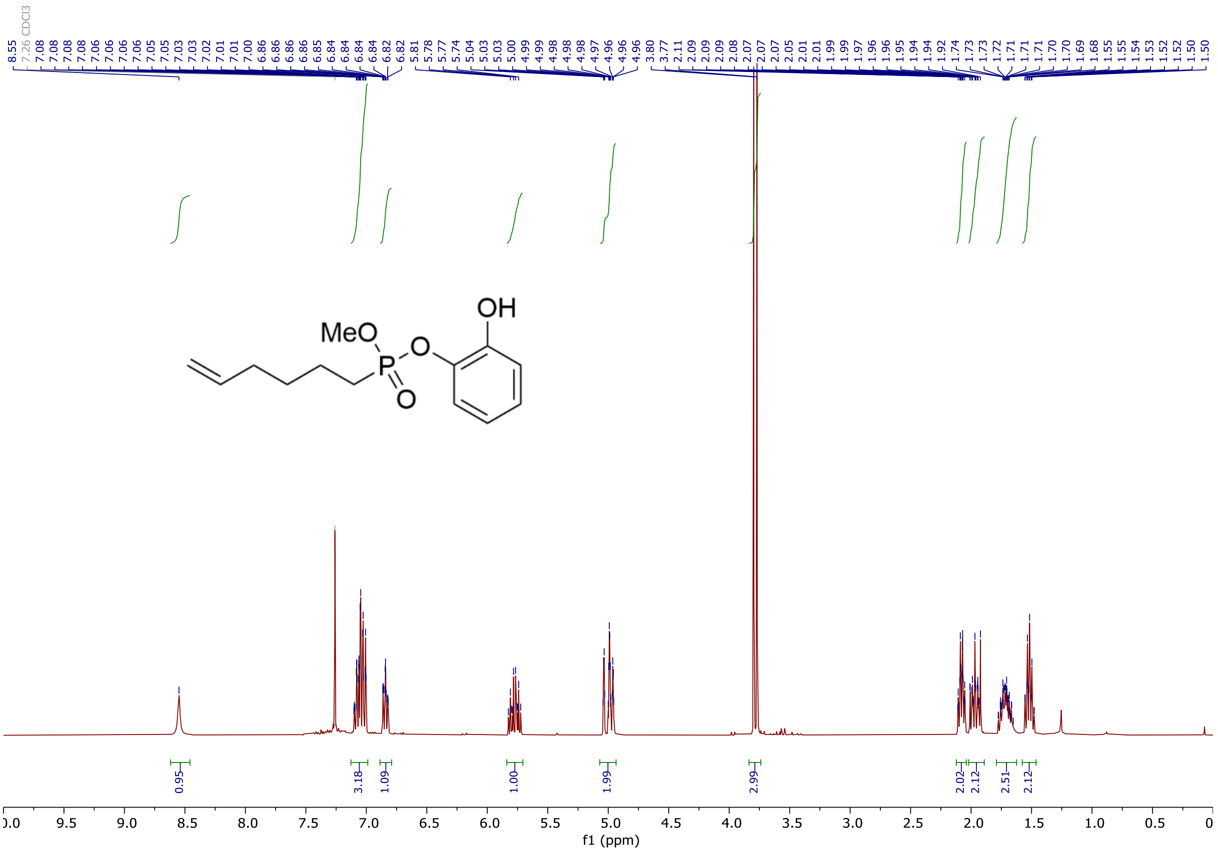

^{13}C NMR (101 MHz, $CDCl_3$): **3bd**

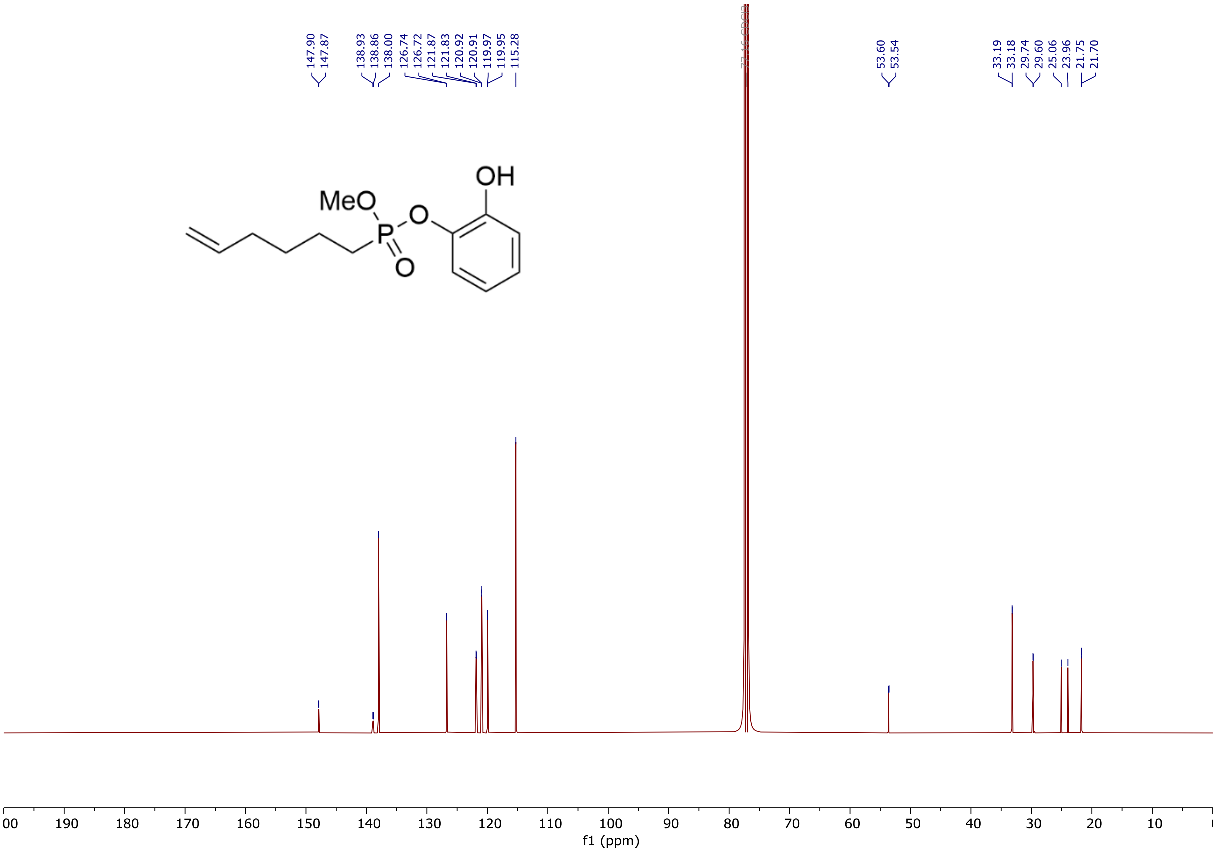

^{31}**P NMR** (162 MHz, $CDCl_3$): **3bd**

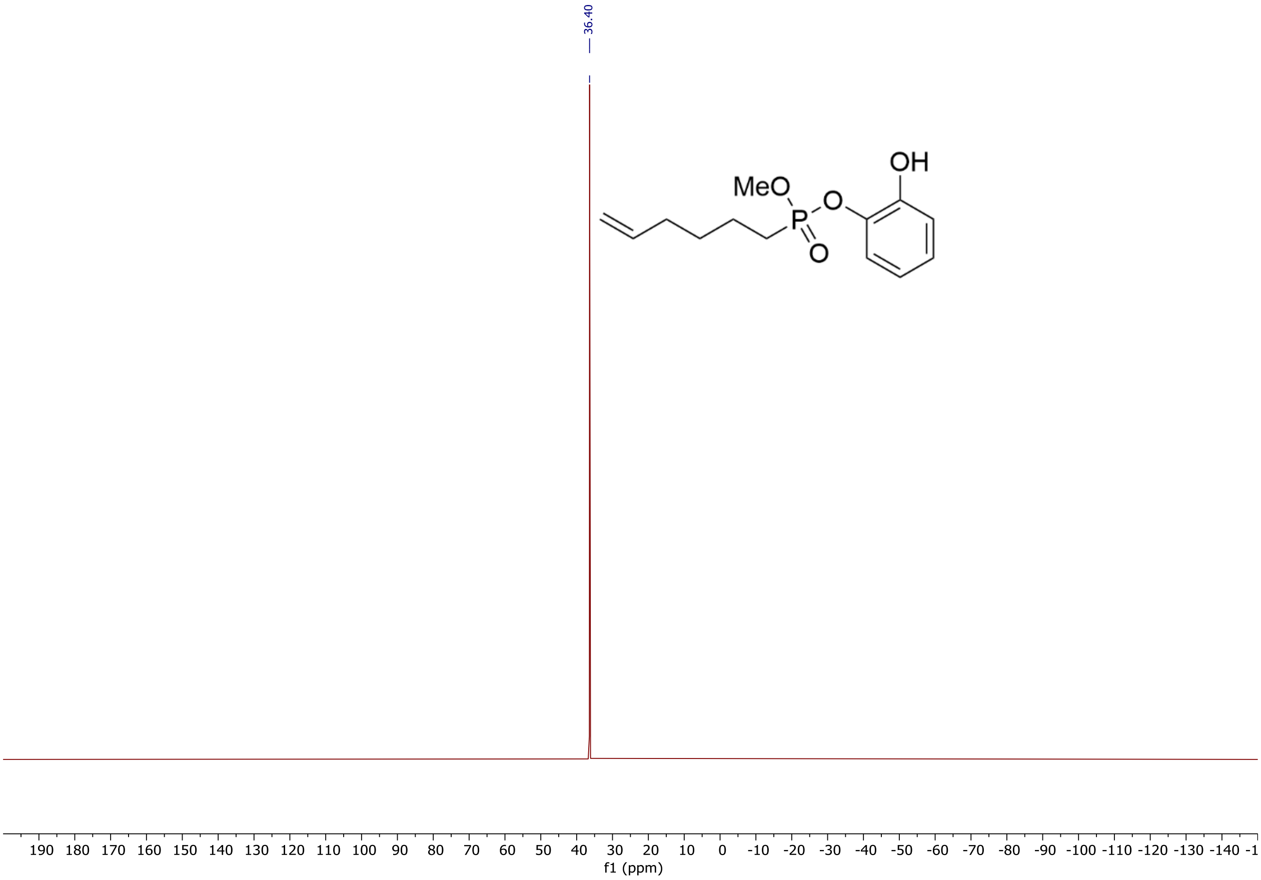

^{1}H NMR (400 MHz, $CDCl_3$): **6**

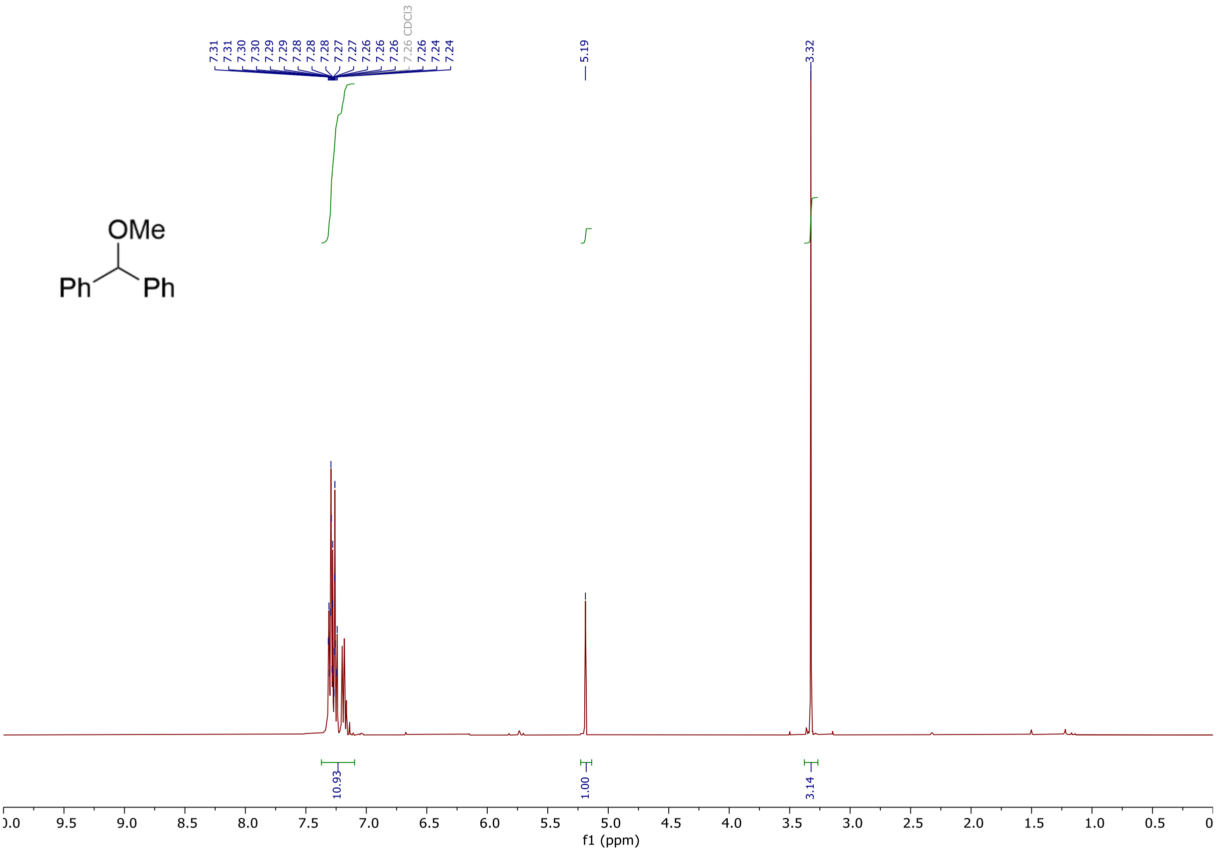

^{13}C NMR (101 MHz, $CDCl_3$): **6**

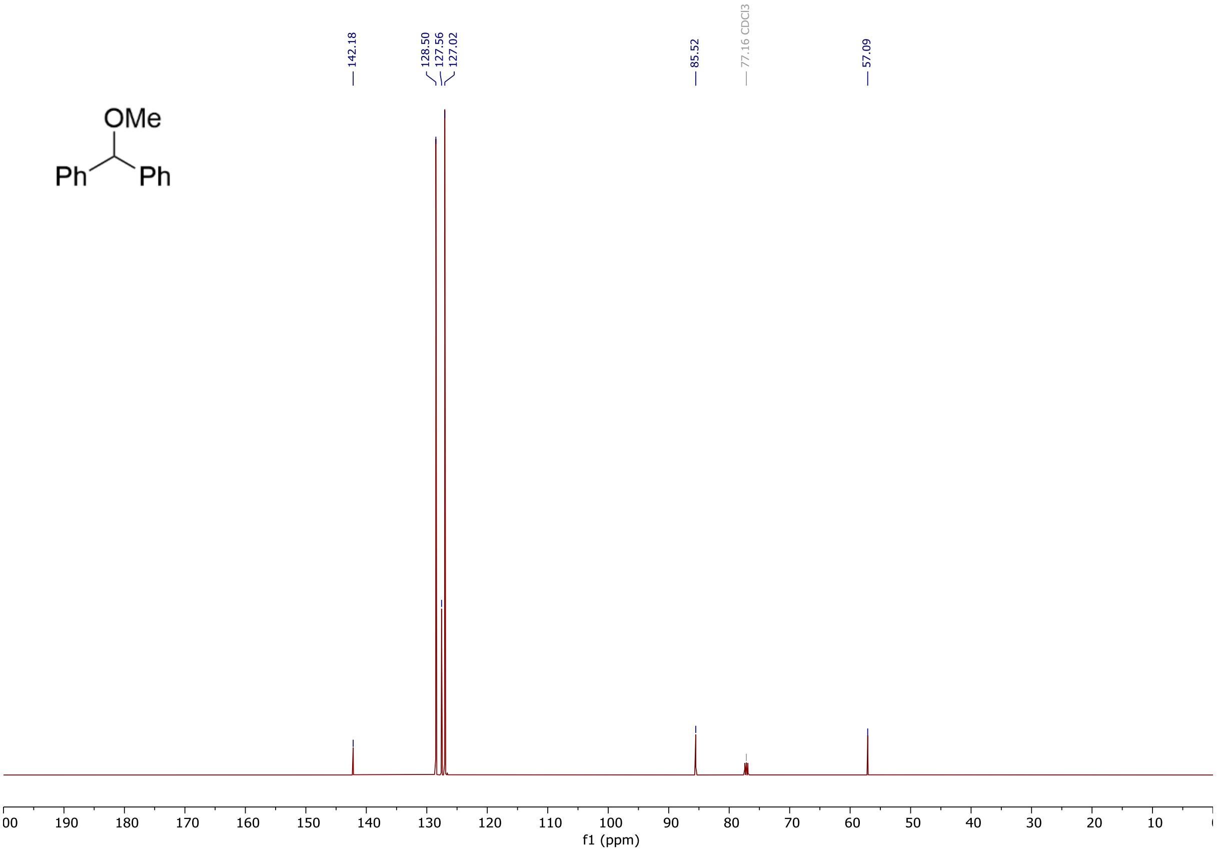

^{1}H NMR (400 MHz, $CDCl_3$): **8a**

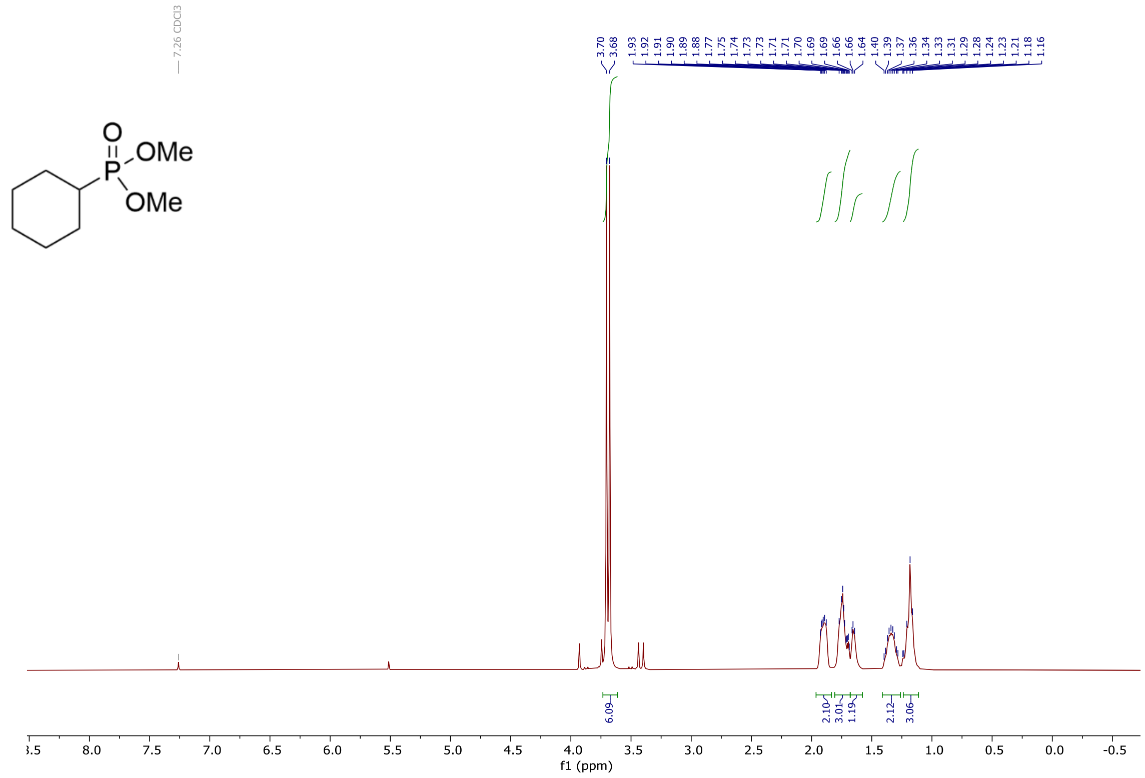

^{13}C NMR (101 MHz, $CDCl_3$): **8a**

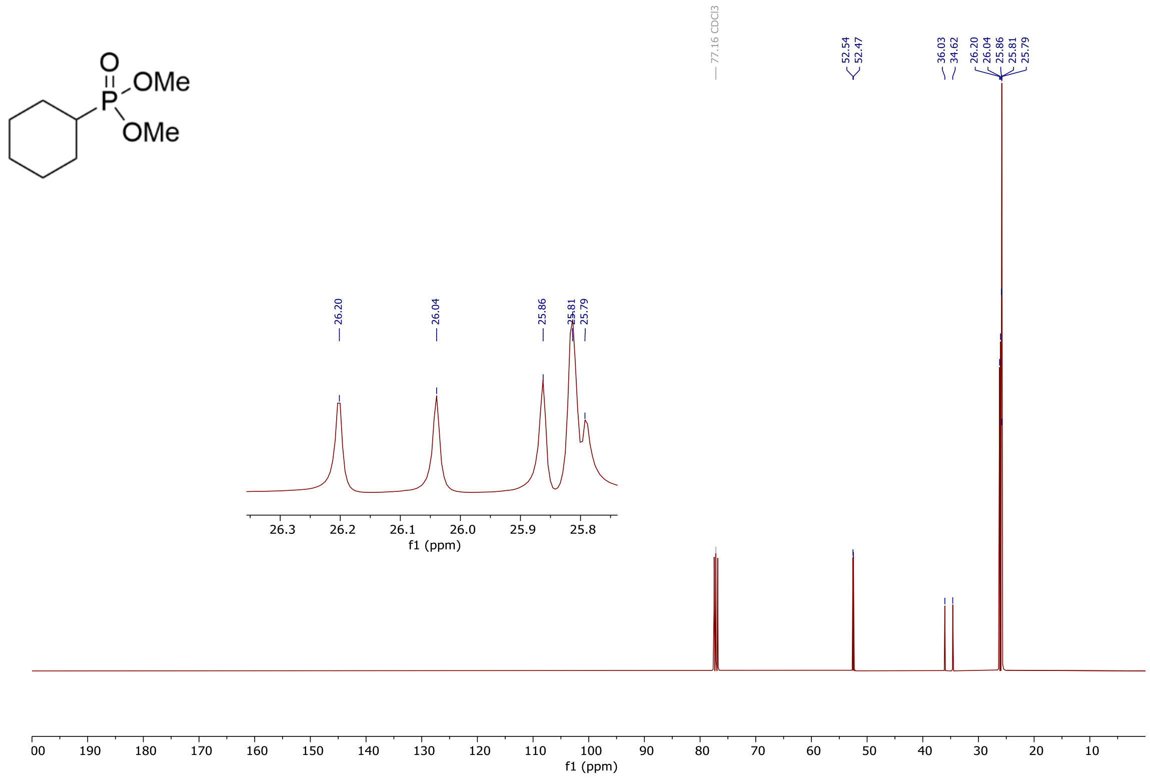

^{1}H NMR (400 MHz, $CDCl_3$): **9a**

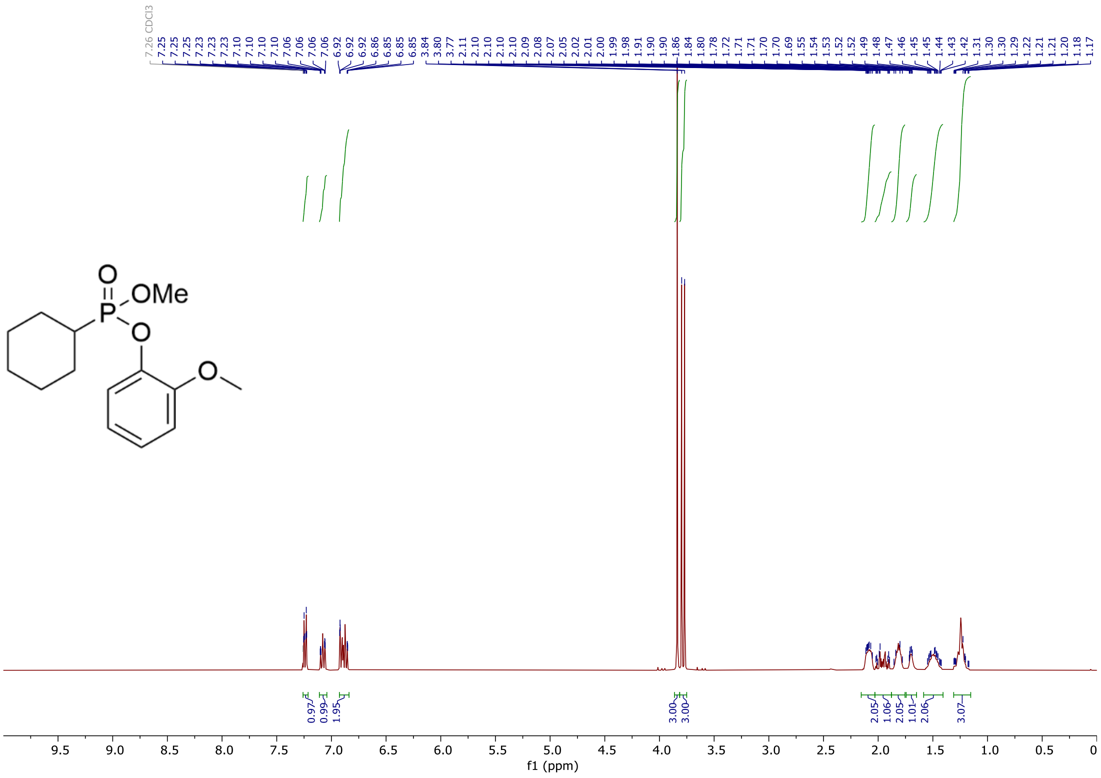

^{13}C NMR (101 MHz, $CDCl_3$): **9a**

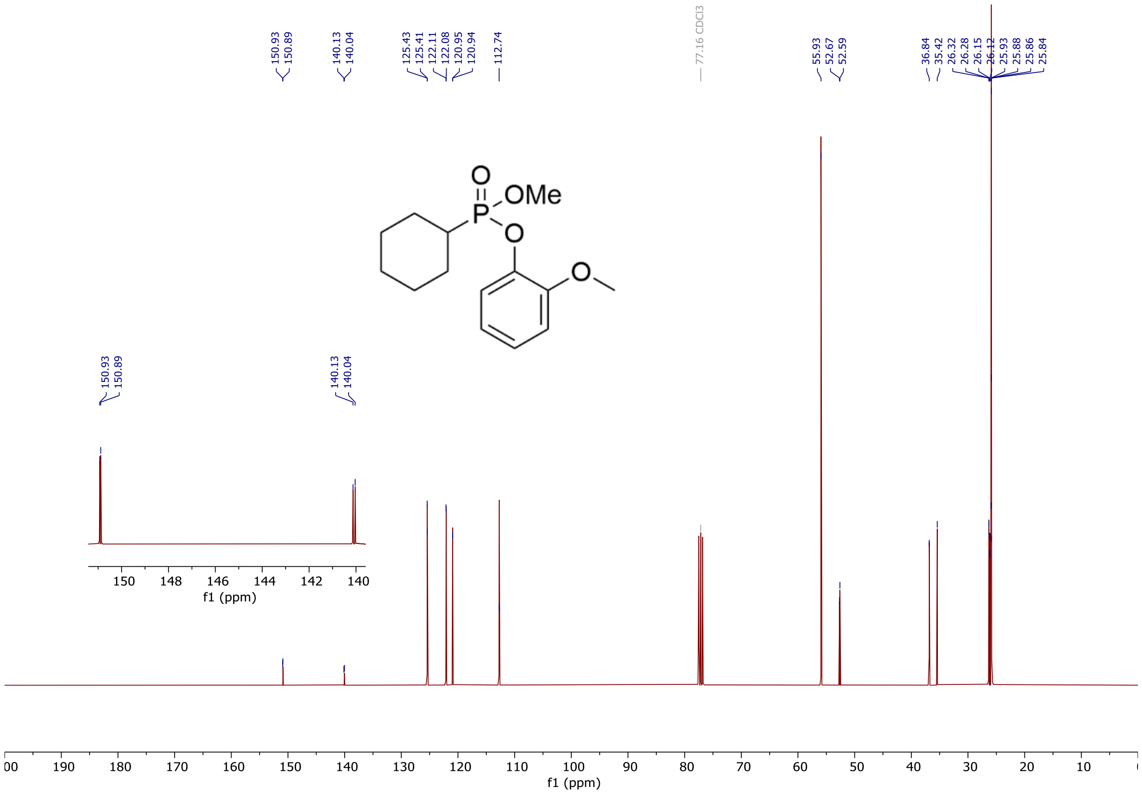

^{31}**P NMR** (162 MHz, $CDCl_3$): **9a**

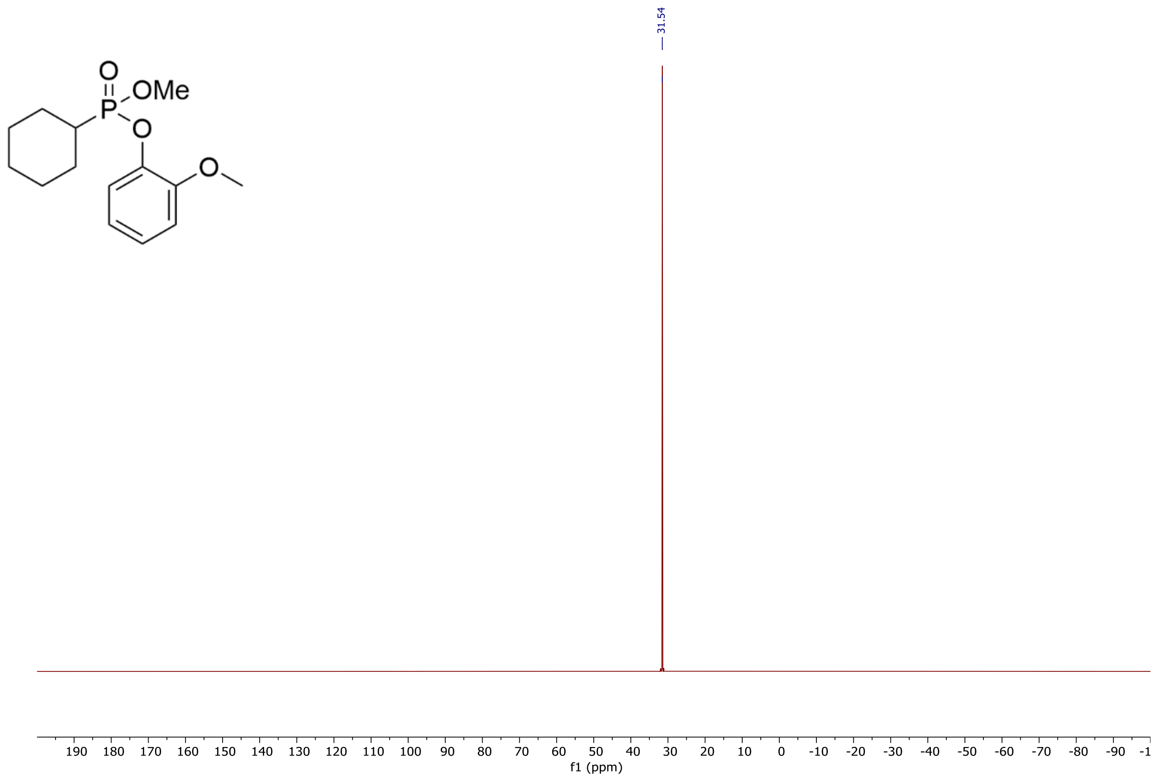

^{1}H NMR (400 MHz, D_2O): **10**

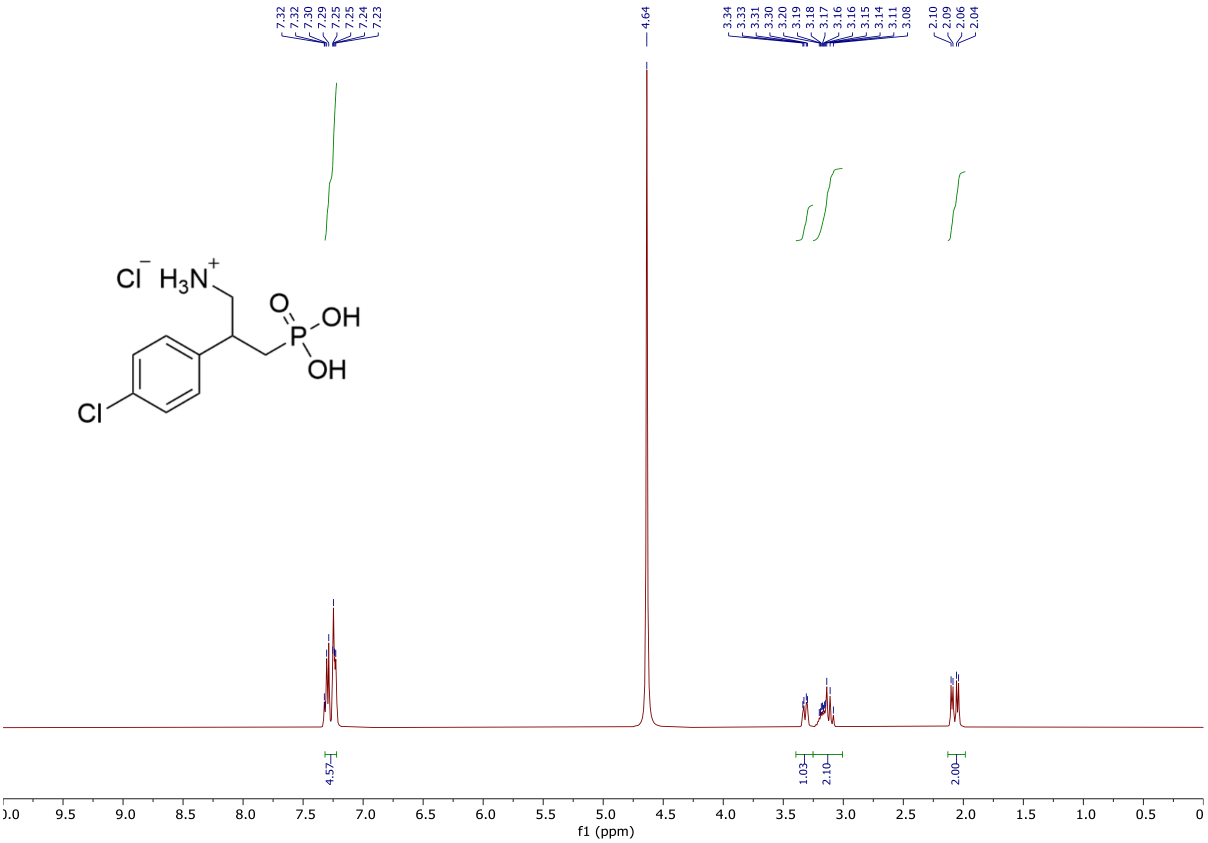

^{13}C NMR (101 MHz, D_2O): **10**

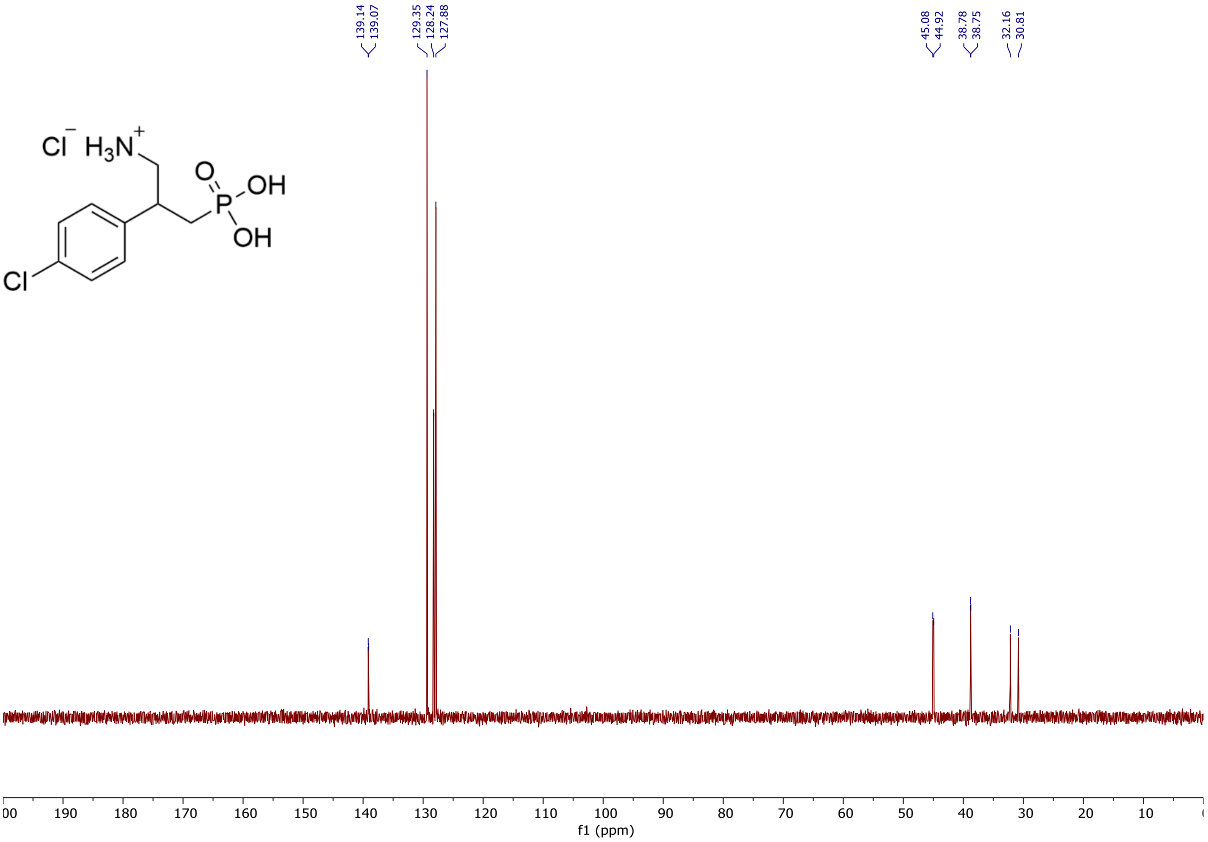

^{31}P NMR (162 MHz, D_2O): **10**

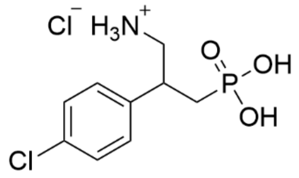

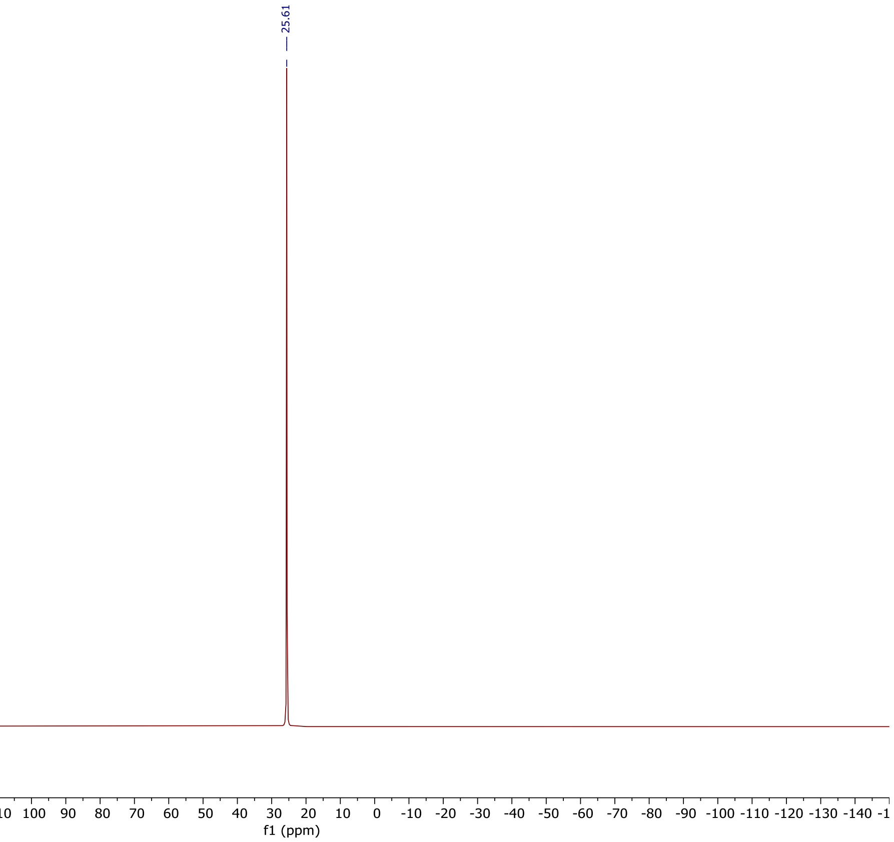

¹H NMR (400 MHz, CDCl₃): **12**

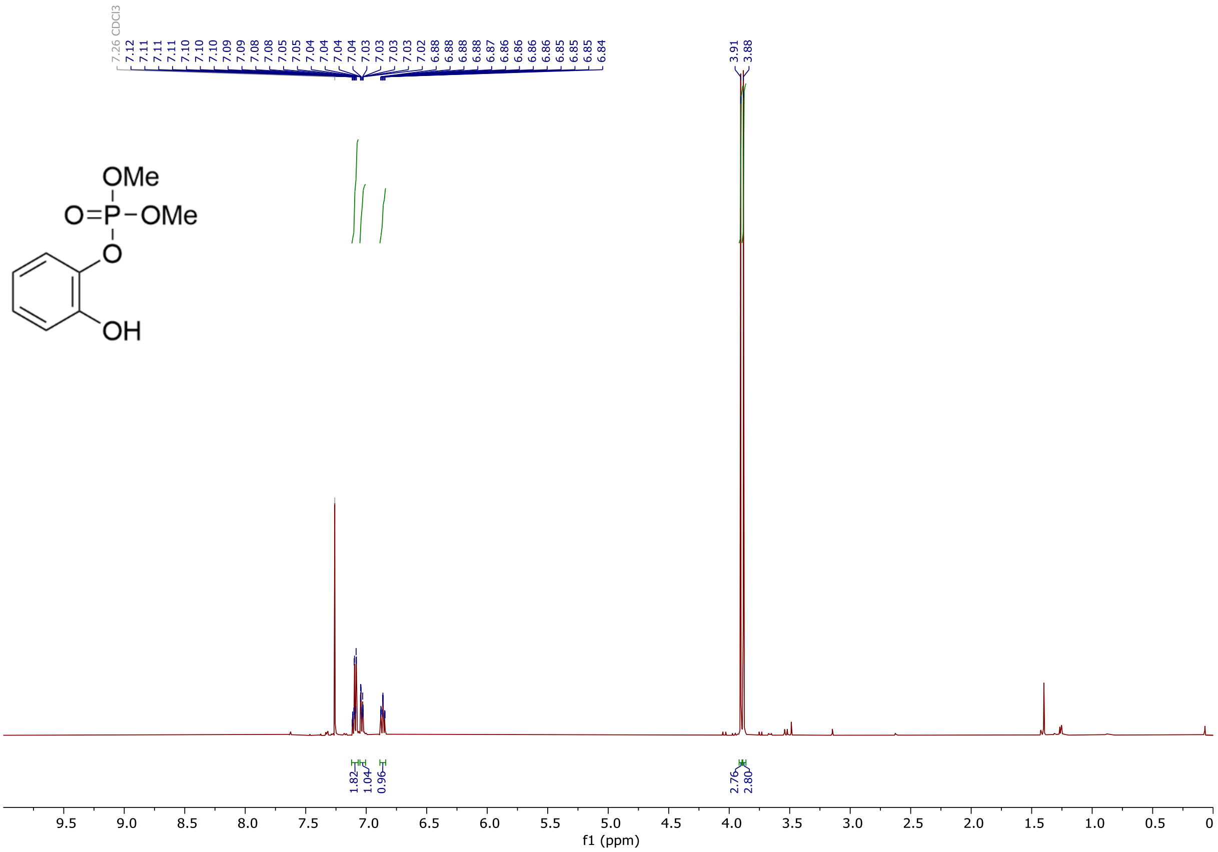

¹³C NMR (101 MHz, CDCl₃): **12**

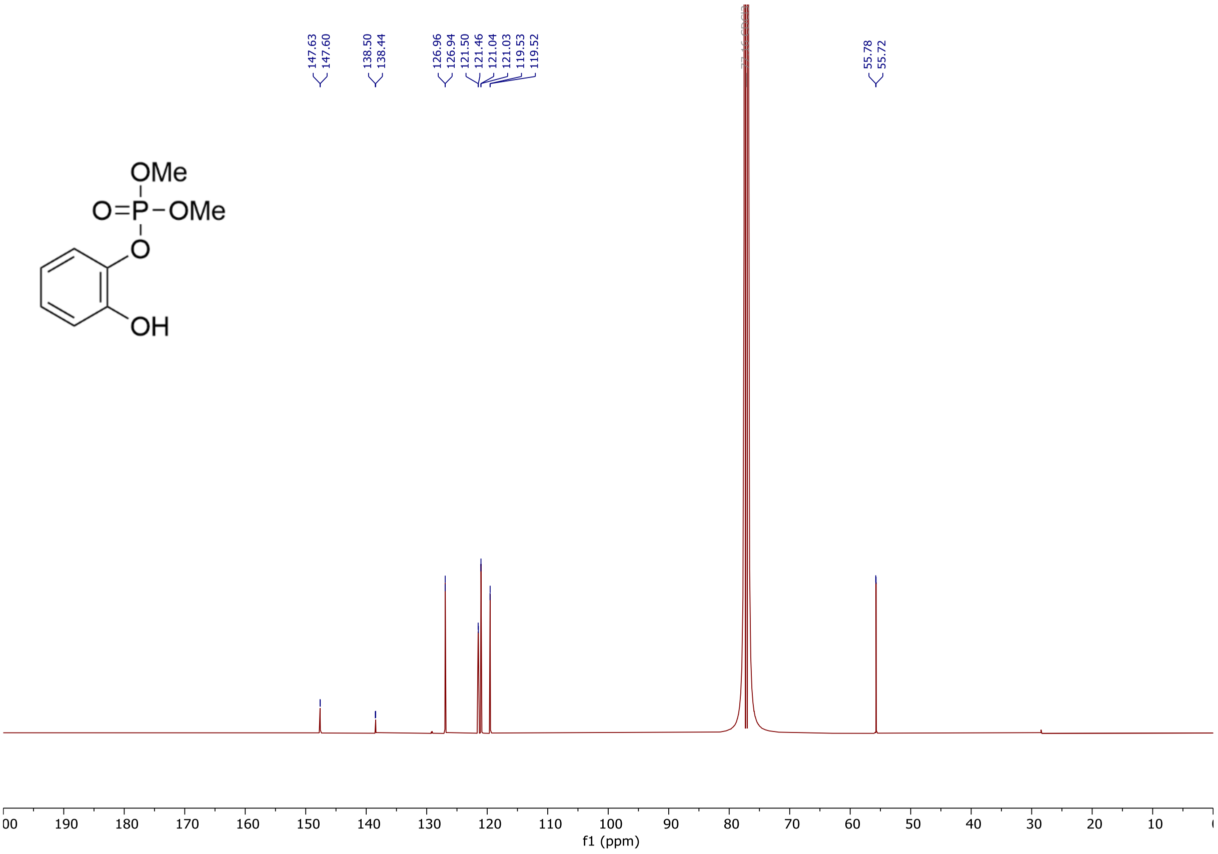

^{31}P NMR (162 MHz, $CDCl_3$): **12**

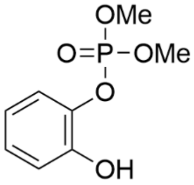

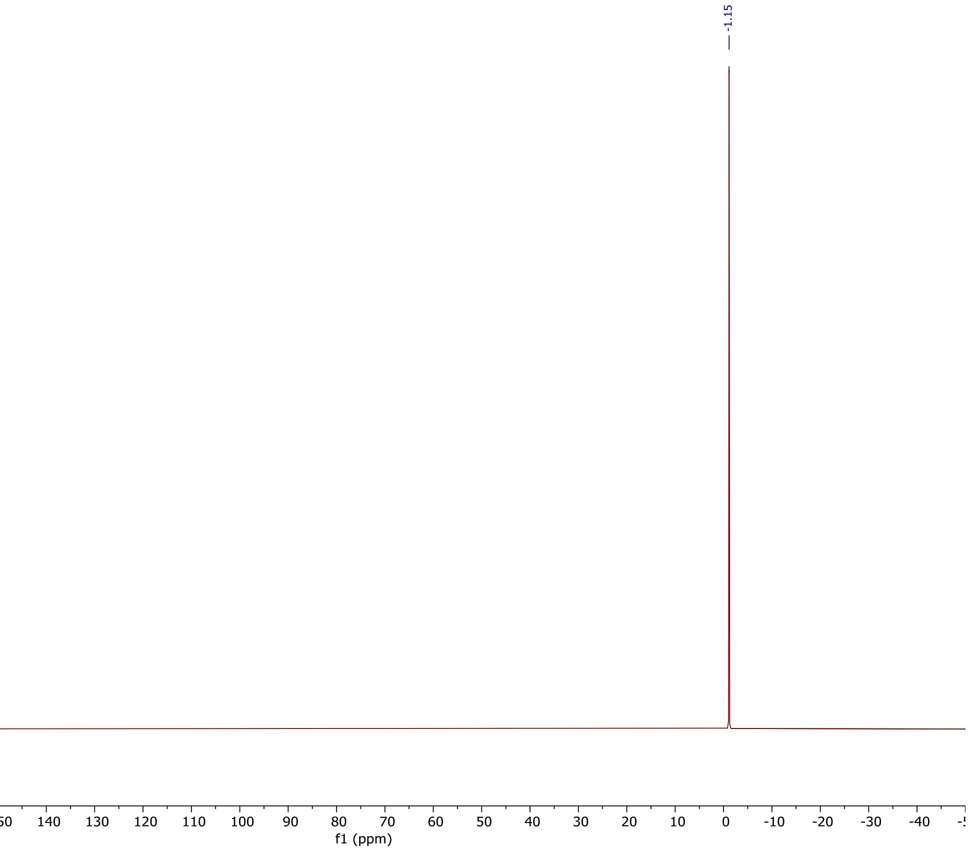

Supplement: Supplementary file 1 — ja3c06524_si_001.pdf [file ja3c06524_si_001.pdf]
